# Supplementary material for: Asymmetric synthesis of stereogenic-at-iridium(III) complexes through Pd-catalyzed kinetic resolution
Source: Nat Commun. 2025 Jan 30;16:1177. doi: 10.1038/s41467-024-55341-4 (PMC11782547; doi:10.1038/s41467-024-55341-4)
Supplement: Supplementary file 1 — Supplementary Information [file 41467_2024_55341_MOESM1_ESM.pdf]

Supplementary Information for

Asymmetric Synthesis of Stereogenic-at-Iridium(III) Complexes through Pd-Catalyzed Kinetic Resolution

Yun-Peng Chu[1], Xue-Lin Yue[1], De-Hai Liu[1], Chuanyong Wang[2] and Jiajia Ma*,[1]

[1]Frontiers Science Center for Transformative Molecules, Shanghai Key Laboratory for Molecular Engineering of Chiral Drugs, School of Chemistry and Chemical Engineering, Zhangjiang Institute for Advanced Study, Shanghai Jiao Tong University, Shanghai 200240, P. R. China

[2]College of Chemistry and Chemical Engineering, Yangzhou University, Yangzhou 225002, China

*Corresponding author: majj@sjtu.edu.cn

Content

1. General Information **2**

2. Reaction Optimization **3**

3. Synthesis of Starting Materials **9**

4.General Procedure for Kinetic Resolution and Characterization Data **41**

5. Synthetic Transformations **157**

6. High Temperature NMR Experiment of Compound 31 .... **176**

7. Study of Coordination Stability of Rh-25 .... **178**

8. Kinetic Experiments **180**

9. Circular Dichroism **188**

10. Attempting to Enhance Enantiopurity of Product via Recrystallization **209**

11. Configuration Stability Test of Iridium complex **210**

12. Single Crystal X-Ray Diffraction **211**

13. Reference **213**

1. General Information

General remarks

Unless otherwise noted, all reactions were carried out under an atmosphere of argon or nitrogen in dried glassware. Reaction temperatures are referred to the ones of the heating/cooling media (heating block, cryogenic bath), unless otherwise stated. Dry solvents were either purchased from Adamas (H_2O < 50 ppm), stored under activated molecular sieves, withdrawn under positive argon pressure Solvents for flash column chromatography (Acetone, petroleum ether, EtOAc, CH_2Cl_2) were purchased of technical grade and purified by atmospheric pressure distillation or purchased of reagent grade (MeOH) and used without additional purification. Reagents were purchased from TCI, Adamas, Bidepharmand used without additional purification, unless otherwise stated.

Purification techniques

Flash chromatography was performed on silica gel (200-300 mesh) under a slight positive pressure. Thin layer chromatography (TLC) was carried out on YANTAI XINNUO silica gel 60F254 pre-coated glass sheets and were visualized using UV light (254 nm/365 nm). Analytical techniques NMR-spectra were recorded on a Bruker Avance II 400 MHz or 500 MHz spectrometers. Chemicals shifts (δ) are quoted in ppm downfield of tetramethyl silane (0.00 ppm). The residual solvent signals were used as references for ^{1}H and ^{13}C NMR spectra ($CDCl_3$: δH = 7.26 ppm, δC = 77.16 ppm), A testing method that concurrently decouples hydrogen and fluorine was utilized to acquire ^{13}C NMR spectra of fluorine-containing compounds. ^{19}F NMR spectra were calibrated using absolute referencing to the ^{1}H NMR spectrum, as suggested by IUPAC. (Harris et al., 2001) Coupling constants (*J*) are quoted in Hz and rounded to the nearest 0.1 Hz. The multiplicity abbreviations used (or combinations thereof) are: s = singlet, d = doublet, t = triplet, q = quartet, hept = heptet, m = multiplet. Enantiomeric excess values were determined by HPLC with Daicel Chirapak column on Agilent 1260 series with *i*-PrOH and *n*-hexane. The CDs were examined by Circular Dichroism J815. High Resolution Mass Spectrometry (HRMS) analysis was obtained using Electrospray Ionization (ESI) and reported as m/z (relative intensity).

2. Reaction Optimization

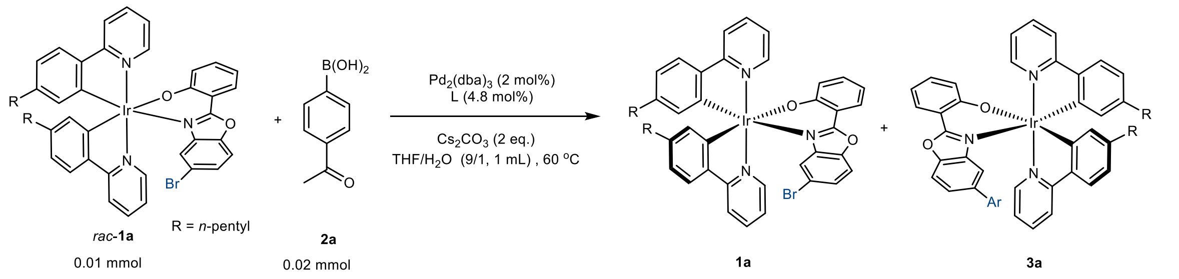

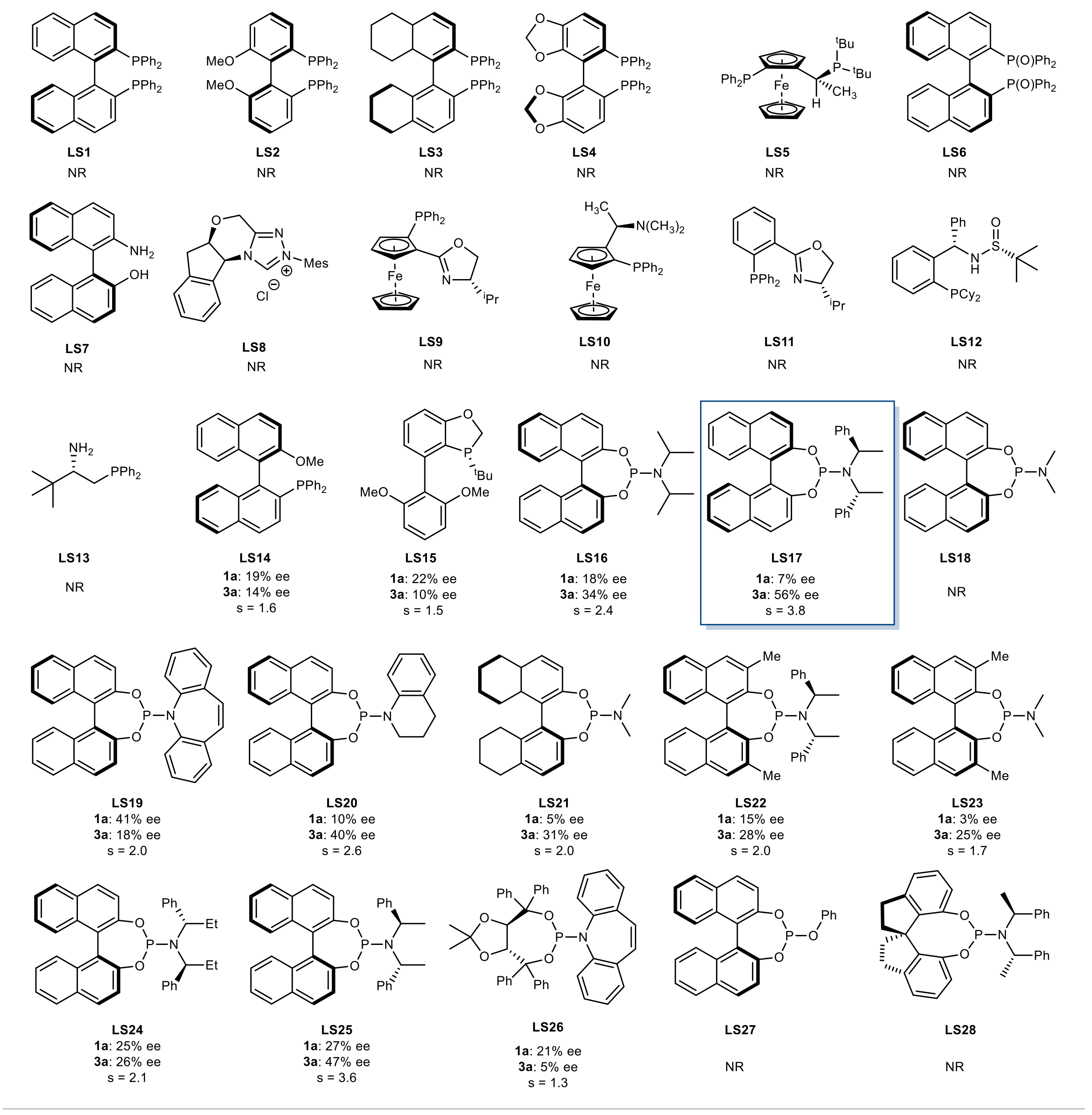

Figure S1. **Optimization of chiral ligand**. Conditions: *Rac*-**1a** (0.01 mmol), **2a** (0.02 mmol), $Pd_2(dba)_3$ (2 mol%), ligand (4.8 mol%) and Cs_2CO_3 (0.02 mmol) in THF/H_2O (v/v=9/1, 1 mL) were stirred at 60 °C under N_2. Ee values were determined by HPLC analysis on a chiral stationary phase and the absolute values are displayed.

Table S1. Optimization of base and solvent. [a]

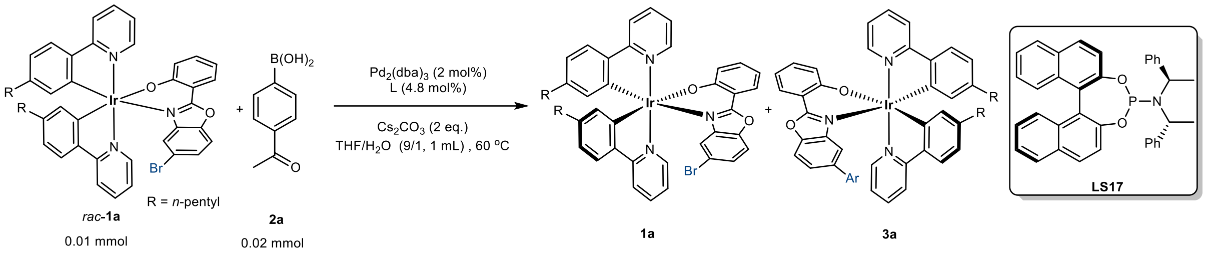

Entry	T/°C	base	solvent	**1a**/ee[b] (%)	**3a**/ee[b] (%)
1	60	Cs_2CO_3	THF/H_2O	7	56
2	50	KF	THF/H_2O	-	63
3	50	CsF	THF/H_2O	-	64
4	70	NaF	THF/H_2O	-	-
5	60	K_2CO_3	THF/H_2O	12	46
6	60	$K_3PO_4\cdot 3H_2O$	THF/H_2O	3	47
7	70	Na_2CO_3	THF/H_2O	-	-
8	70	Cs_2CO_3	DMF	-	-
9	50	Cs_2CO_3	MeCN	10	49

[a]Conditions: *Rac*-**1a** (0.01 mmol), **2a** (0.02 mmol), $Pd_2(dba)_3$ (2 mol%), ligand (4.8 mol%) and Cs_2CO_3 (0.02 mmol) in THF/H_2O (v/v=9/1, 1 mL) were stirred at designated temperatures under N_2. [b]Ee values were determined by HPLC analysis on a chiral stationary phase and the absolute values are displayed.

A series of chiral ligands were evaluated in the kinetic resolution of ***rac-1a****, the Feringa's phosphonamidite LS17 was found to be applicable for the reaction, thus giving the desired cross-coupling product* ***3a*** *with 56% ee (Figure S1). Subsequently, screenings were conducted for solvents and bases, which indicated that the reaction could proceed at 50 °C using CsF as the base, and the ee values were confirmed to be 64% for* ***3a*** *(Table S1).*

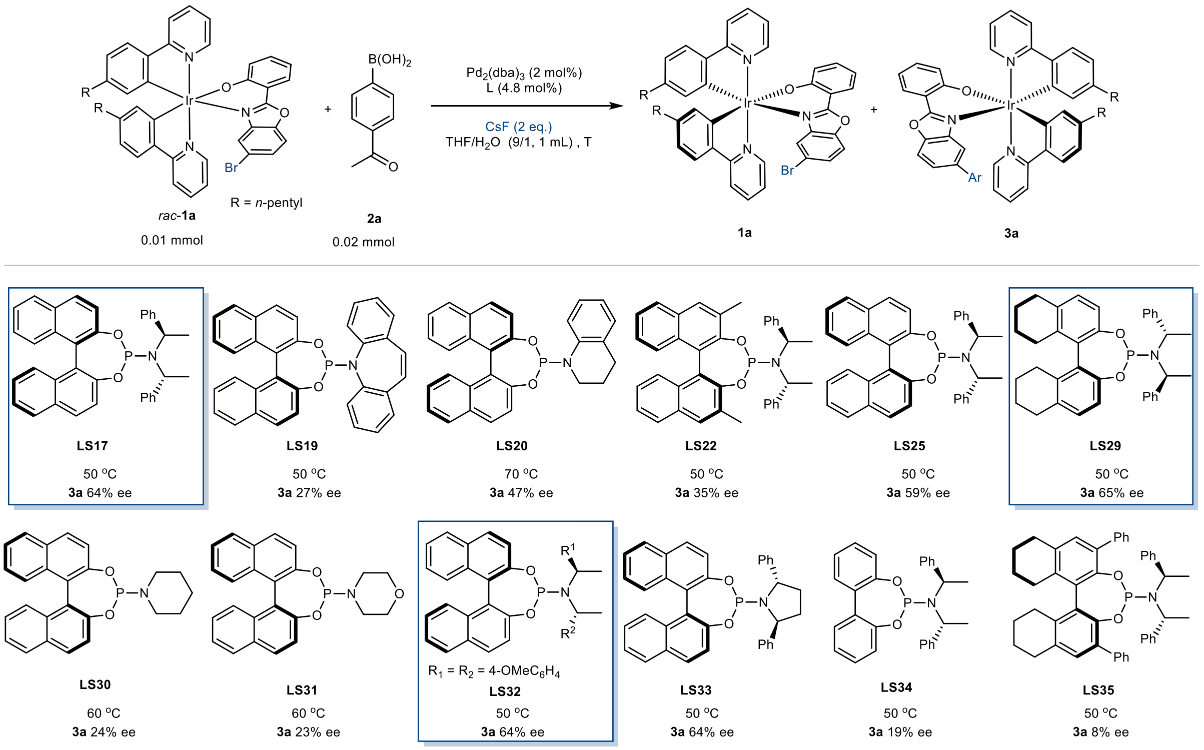

Figure S2. **Optimization of chiral ligand**. Conditions: *Rac*-**1a** (0.01 mmol), **2a** (0.02 mmol), $Pd_2(dba)_3$ (2 mol%), ligand (5 mol%) and CsF (0.02 mmol) in THF/H_2O (v/v=9/1, 1 mL) were stirred at designated temperatures under N_2. When the conversions of reactions were less than 10%, the ee values of **3a** were measured. Ee values of **3a** were determined by HPLC analysis on a chiral stationary phase and the absolute values are displayed.

After establishing CsF as the base, further screenings were conducted on additional phosphonamidite ligands (Figure S2). The results indicated that ligands LS17, LS29 and LS32 exhibited similar enantioselectivity for the kinetic resolution, with the ee values of the obtained product ***3a*** *ranging from 64-65%. (Notably, the diastereomer LS25 of LS17, when participating in the catalysis of the reaction, exhibits poor enantioselectivity. Homochiral phosphoramidite ligands (R,R,R or S,S,S) are more suitable for this reaction. Additionally, the experimental results indicate that the axial chirality of the binaphthyl unit plays a dominant role in controlling the enantioselectivity of the reaction.)*

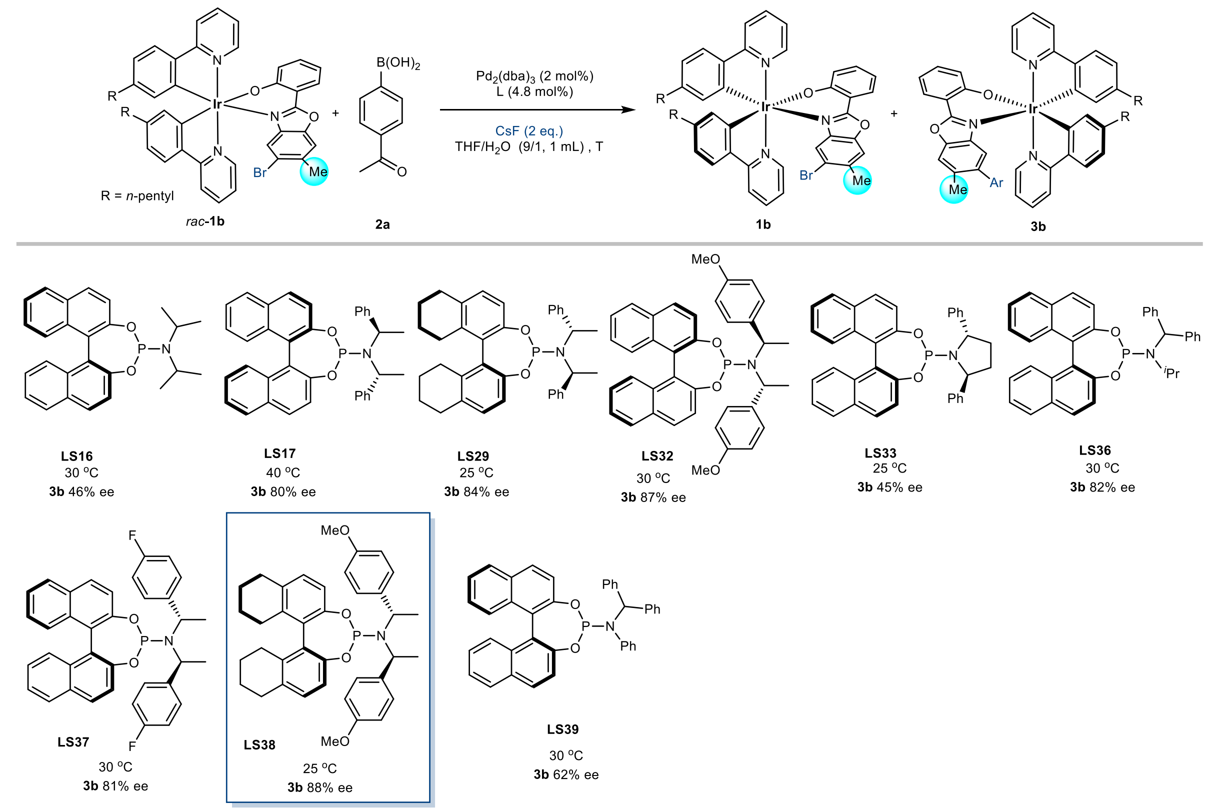

Figure S3. **Optimization of chiral ligand**. Conditions: *Rac*-**1b** (0.01 mmol), **2a** (0.02 mmol), $Pd_2(dba)_3$ (2 mol%), ligand (4.8 mol%) and CsF (0.02 mmol) in THF/H_2O (v/v=9/1, 1 mL) were stirred at designated temperatures under N_2. When conversion of reactions were less than 10%, the ee values of **3b** were measured. Ee values of **3b** were determined by HPLC analysis on a chiral stationary phase and the absolute values are displayed.

*Next, the substrate was adjusted by introducing substituents to ortho position of the bromo in rac-***1a***. Using iridium complex* **1b** *as a template substrate, a series of phosphonamidite ligands were screened (Figure S3). The reaction could proceed at 25 °C using LS38 as the chiral ligand, and the ee values were confirmed to be 88% for* **3b***.*

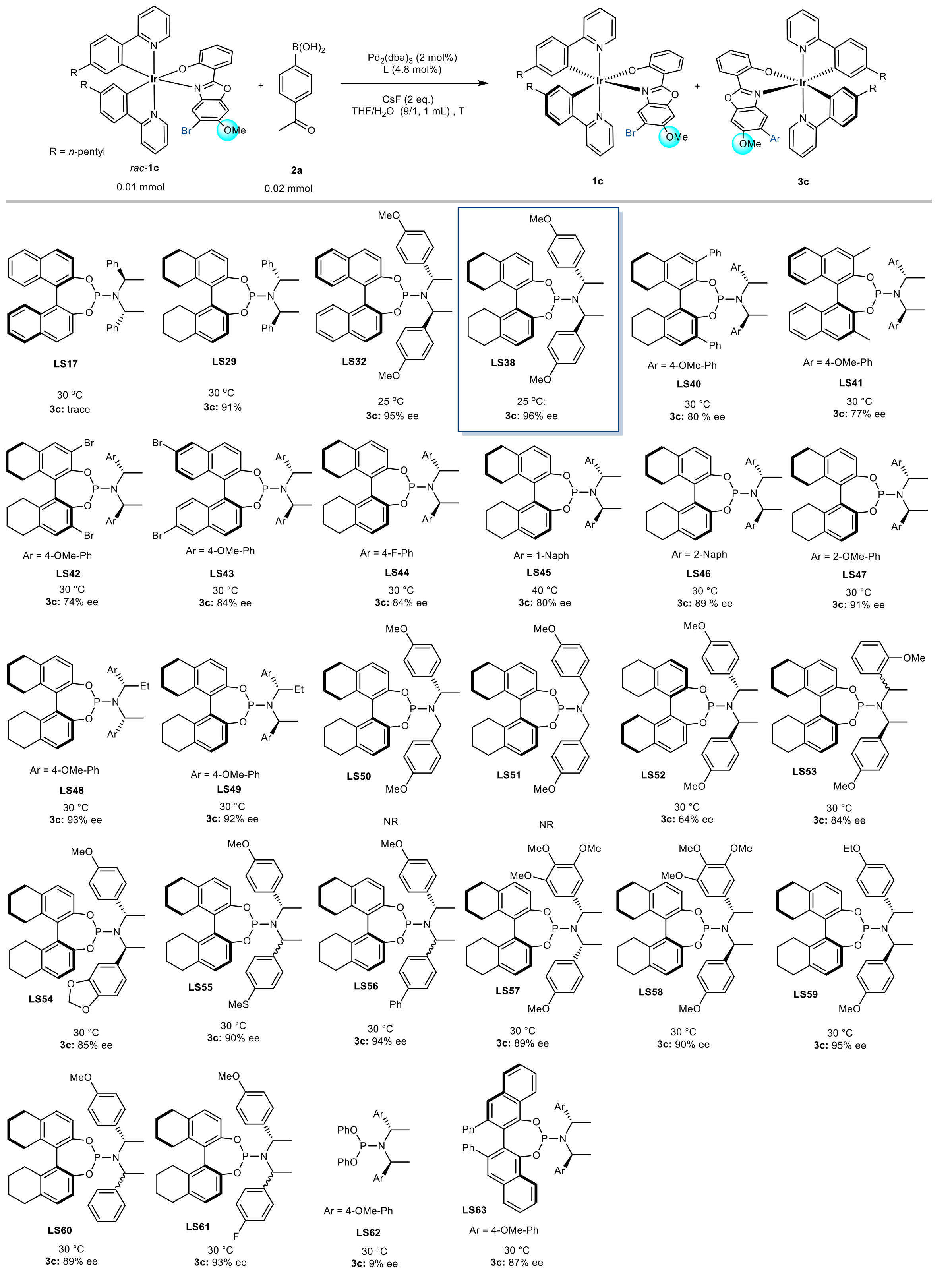

Figure S4. **Optimization of chiral ligand**. [a]Conditions: *Rac*-**1c** (0.01 mmol), **2a** (0.02 mmol), $Pd_2(dba)_3$ (5 mol%), ligand (12 mol%) and CsF (0.02 mmol) in THF/H_2O (v/v=9/1, 1 mL) were stirred at designated temperatures under N_2. When the conversions of reactions were less than 10%, the ee values of **3c** were measured. Ee values of **3c** were determined by HPLC analysis on a chiral stationary phase and the absolute values are displayed.

Table S2. Optimization of catalyst concentration and reaction temperature.

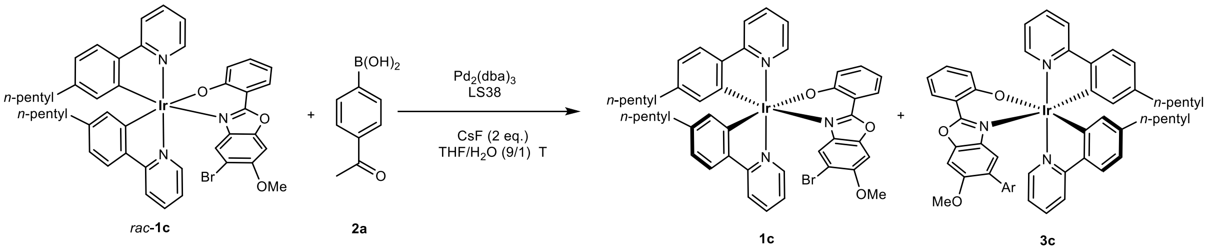

Entry	T/°C	$Pd_2(dba)_3$	L	t/h	Concentration (mol/L)	**1c**/ee (%)[b]	**3c**/ee (%)[b]	C[c](%)	S[d]
1	25	2 mol%	4.8 mol%	24	0.01	5	96	5	46
2	25	2 mol%	4.8 mol%	36	0.1	19	95	17	47
3	30	2 mol%	4.8 mol%	70	0.1	48	91	35	34
4	35	2 mol%	4.8 mol%	12	0.1	12	93	11	31
5	40	2 mol%	4.8 mol%	12	0.1	15	92	14	28
6	30	5 mol%	12 mol%	70	0.1	93	87	52	49
7[e]	30	5 mol%	12 mol%	84	0.1	90	90	50	58

[a]Conditions: *Rac*-**1c** (0.01 mmol), **2a** (0.02 mmol), $Pd_2(dba)_3$, ligand and CsF (0.02 mmol) in THF/H_2O (v/v=9/1) were stirred at designated temperatures under N_2. [b]Determined by HPLC analysis on a chiral stationary phase and the absolute values are displayed. [c]Conversion (C) = $ee_s/(ee_s+ee_p)$. [d]$s = \ln[(1-C)(1-ee_s)]/\ln[(1-C)(1+ee_s)]$. [e]Reaction was performed on 0.05 mmol scale.

To further modify the substrate, a methoxy group was introduced at the ortho position of the bromo in ***rac-1a****, which was then fixed as the template substrate for screening a series of phosphonamidite ligands. The results indicated that ligand LS38 was the optimal ligand (Figure S4). Subsequently, the reaction conditions such as temperature and catalyst concentration were optimized (Table S2). Finally, after conducting a 0.05 mmol scale reaction at 30 °C for 84 h under the optimized conditions, the ee values were confirmed to be 90% for* ***1c*** *and 90% for* ***3c****.*

3. Synthesis of Starting Materials

*Cyclometalated ligands (**S3-S8**) were prepared according to the modified procedure*[1]*, other cyclometalated ligands were prepared according to the corresponding procedures*[2–6].

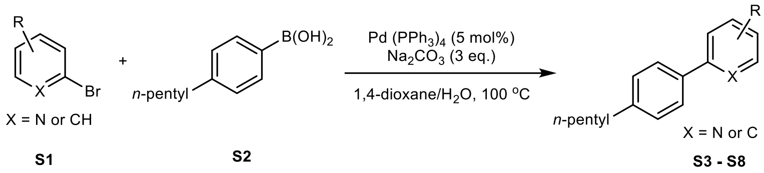

A mixture of **S1** (5 mmol, 1.0 equiv.), **S2** (6 mmol, 1.2 equiv.) and $Pd(PPh_3)_4$ (5 mol%) in 1,4-dioxane/H_2O (20 mL/5 mL) was stirred at reflux under N_2 for 12 h in a 50 mL two-necked flask, then cooled to room temperature. Aqueous NH_4Cl (15 mL) was added to the reaction mixture, and the resulting mixture was extracted with EtOAc three times. The organic layers were collected and dried over $MgSO_4$, then evaporated in vacuum to yield the crude product, which was further purified by flash column chromatography on silica gel.

2-(4-pentylphenyl)pyridine (S3)

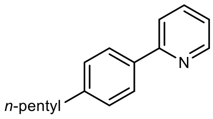

Purification by column chromatography (petroleum ether/EtOAc, 20:1) afforded the title compound (1.01 g, 89%) as a colorless oil.

^1H NMR (500 MHz, Chloroform-*d*) δ 8.64 (d, *J* = 4.5 Hz, 1H), 7.90 (d, *J* = 8.0 Hz, 2H), 7.67 – 7.59 (m, 2H), 7.26 (d, *J* = 8.0 Hz, 2H), 7.15 – 7.08 (m, 1H), 2.67 – 2.59 (m, 2H), 1.63 (p, *J* = 7.5 Hz, 2H), 1.36 – 1.28 (m, 4H), 0.88 (t, *J* = 7.0 Hz, 3H).

^{13}C NMR (126 MHz, $CDCl_3$) δ 157.4, 149.6, 143.9, 136.8, 136.6, 128.8, 126.8, 121.7, 120.2, 35.7, 31.5, 31.1, 22.6, 14.1.

HRMS (ESI) for $C_{16}H_{20}N$ $[M+H]^+$ calcd. 226.1590, found 226.1595;

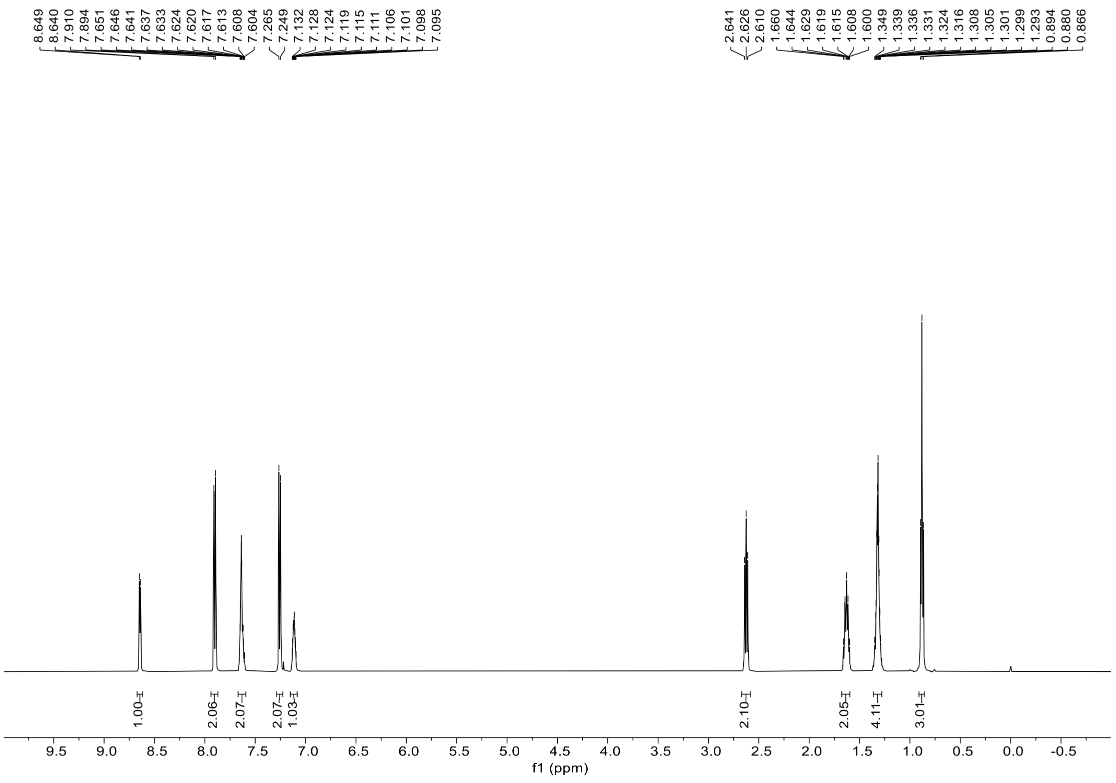

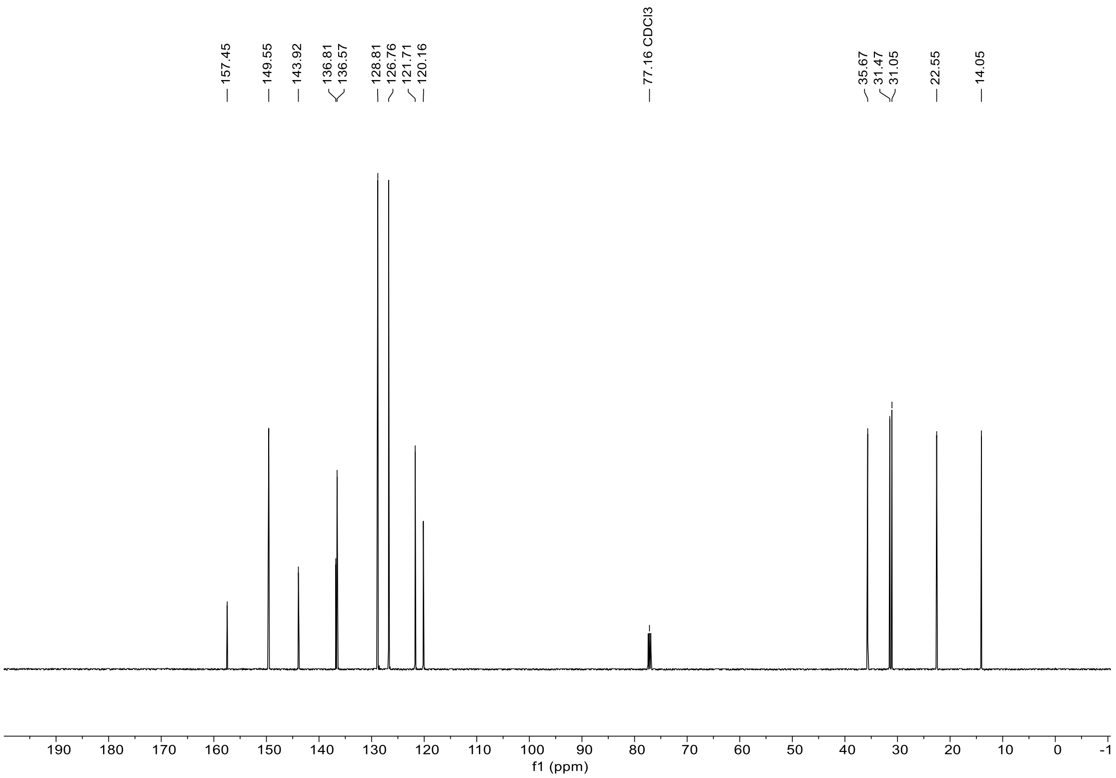

3-(4-pentylphenyl)isoquinoline (S4)

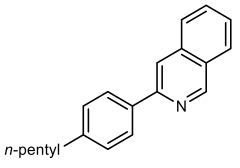

Purification by column chromatography (petroleum ether/EtOAc, 10:1) afforded the title

compound (1.10 g, 80%) as a white solid.

^{1}H NMR (500 MHz, Chloroform-*d*) δ 9.32 (s, 1H), 8.07 – 8.02 (m, 3H), 7.97 (d, *J* = 8.0 Hz, 1H), 7.85 (d, *J* = 8.0 Hz, 1H), 7.71 – 7.65 (m, 1H), 7.59 – 7.53 (m, 1H), 7.32 (d, *J* = 8.0 Hz, 2H), 2.67 (t, *J* = 7.5 Hz, 2H), 1.73 – 1.64 (m, 2H), 1.40 – 1.33 (m, 4H), 0.94 – 0.88 (m, 3H).

^{13}C NMR (126 MHz, $CDCl_3$) δ 152.5, 151.5, 143.7, 137.1, 136.8, 130.6, 129.1, 127.8, 127.7, 127.0, 126.98, 116.2, 35.8, 31.6, 31.3, 22.7, 14.2.

HRMS (ESI) for $C_{20}H_{22}N$ $[M+H]^+$ calcd. 276.1747, found 276.1740;

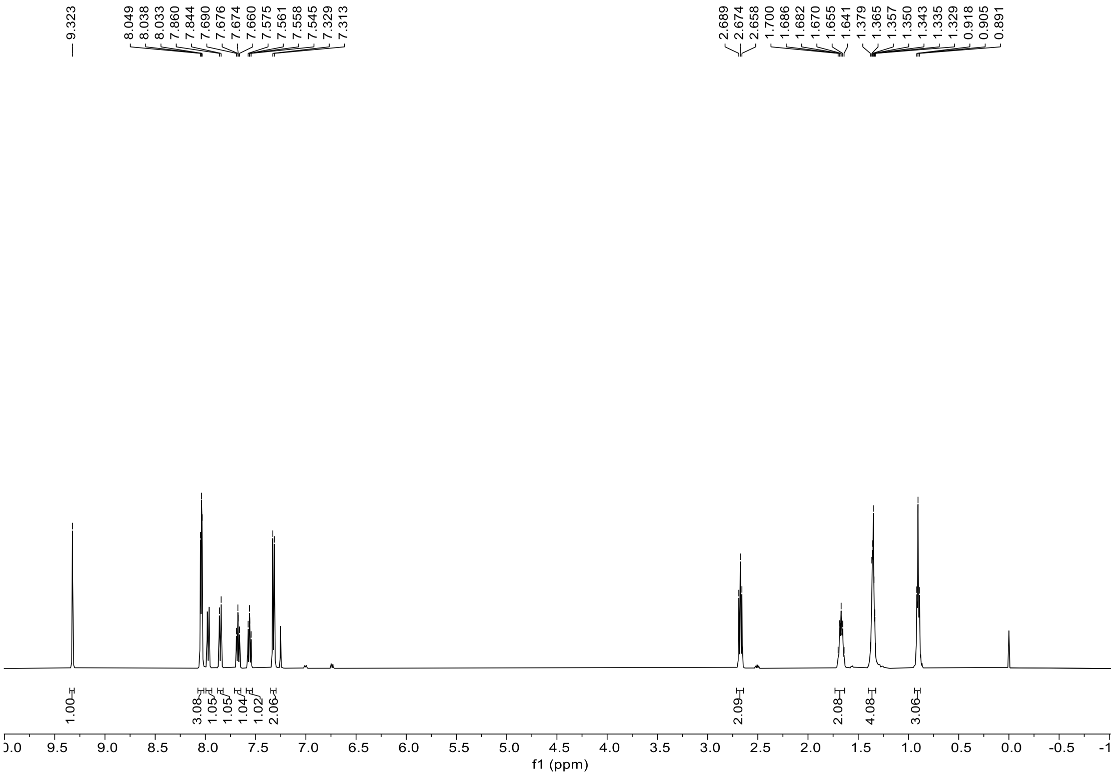

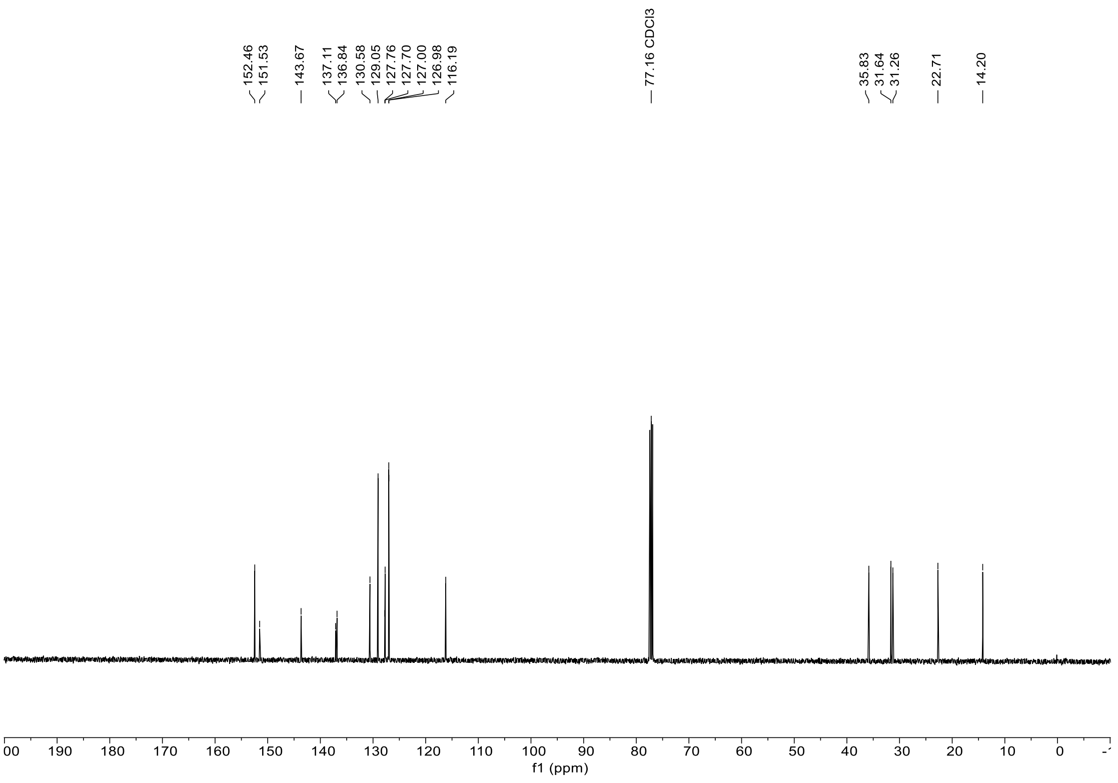

5-methyl-2-(4-pentylphenyl)pyridine (S5)

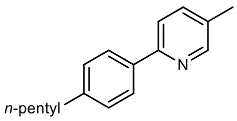

Purification by column chromatography (petroleum ether/EtOAc, 20:1) afforded the title compound (812 mg, 68%) as a colorless oil.

^{1}H NMR (500 MHz, Chloroform-*d*) δ 8.48 (s, 1H), 7.90 – 7.85 (m, 2H), 7.58 – 7.52 (m, 1H), 7.49 – 7.43 (m, 1H), 7.25 (d, *J* = 8.0 Hz, 2H), 2.66 – 2.60 (m, 2H), 2.30 (d, *J* = 3.5 Hz, 3H), 1.64 (p, *J* = 7.0 Hz, 2H), 1.37 – 1.28 (m, 4H), 0.92 – 0.86 (m, 3H).

^{13}C NMR (126 MHz, $CDCl_3$) δ 154.9, 150.0, 143.6, 137.2, 136.9, 131.2, 128.8, 126.6, 119.73, 119.71, 35.7, 31.5, 31.1, 22.6, 18.1, 14.1.

HRMS (ESI) for $C_{17}H_{22}N$ $[M+H]^+$ calcd. 240.1747, found 240.1745;

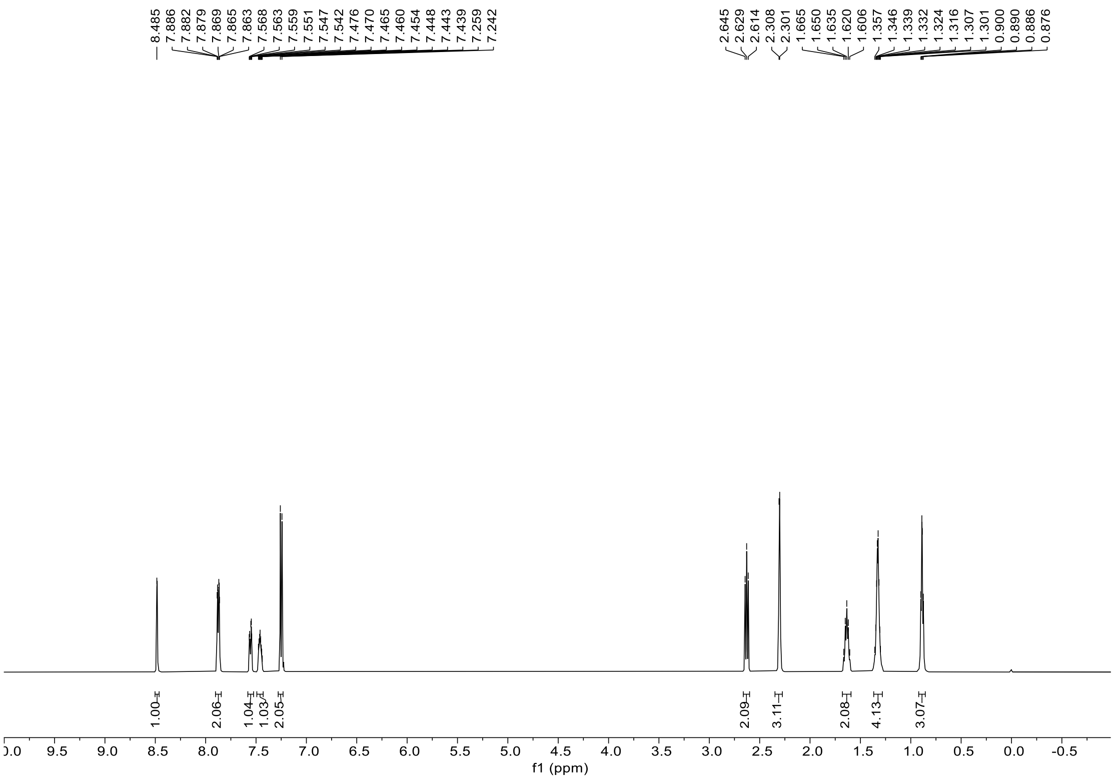

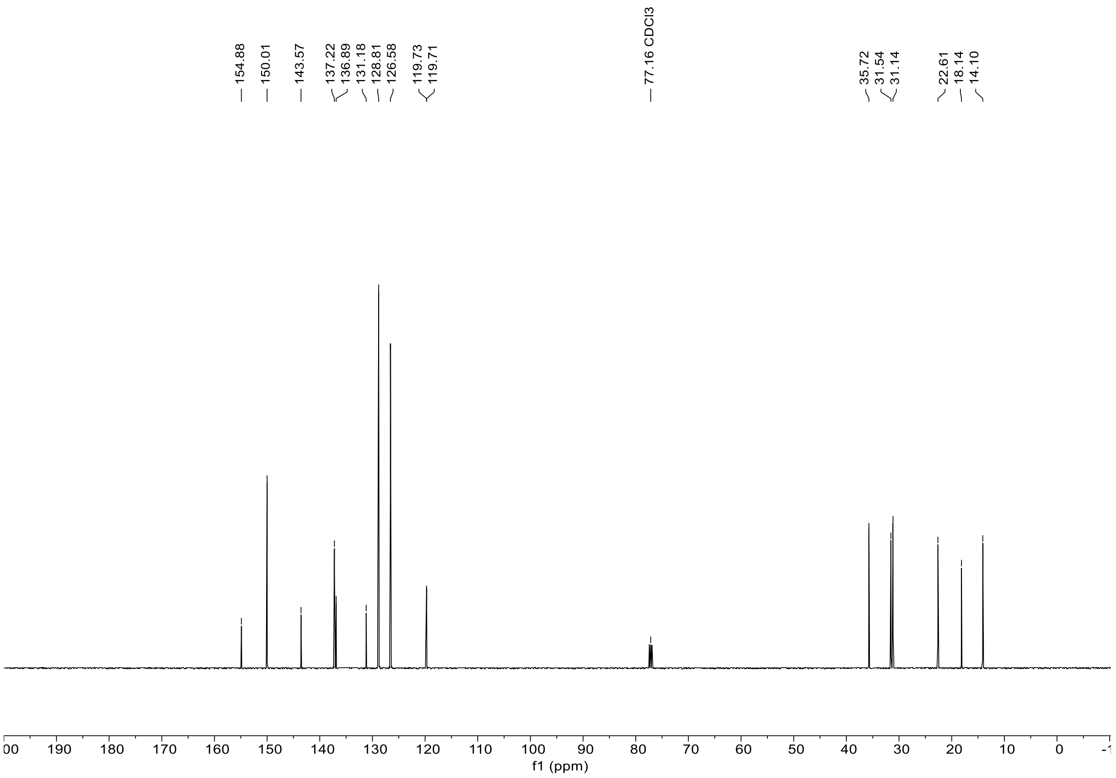

4-methyl-2-(4-pentylphenyl)pyridine (S6)

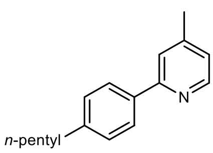

Purification by column chromatography (petroleum ether/EtOAc, 20:1) afforded the title

compound (1.08 g, 90%) as a colorless oil.

^{1}H NMR (500 MHz, Chloroform-*d*) δ 8.50 (d, *J* = 5.0 Hz, 1H), 7.89 (d, *J* = 8.0 Hz, 2H), 7.48 (s, 1H), 7.25 (d, *J* = 8.0 Hz, 2H), 6.97 (d, *J* = 5.0 Hz, 1H), 2.66 – 2.60 (m, 2H), 2.34 (s, 3H), 1.63 (p, *J* = 7.0 Hz, 2H), 1.38 – 1.27 (m, 4H), 0.88 (t, *J* = 7.0 Hz, 3H).

^{13}C NMR (126 MHz, $CDCl_3$) δ 157.4, 149.4, 147.6, 143.8, 137.0, 128.8, 126.8, 122.8, 121.2, 35.7, 31.5, 31.1, 22.6, 21.2, 14.1.

HRMS (ESI) for $C_{17}H_{22}N$ $[M+H]^+$ calcd. 240.1747, found 240.1743;

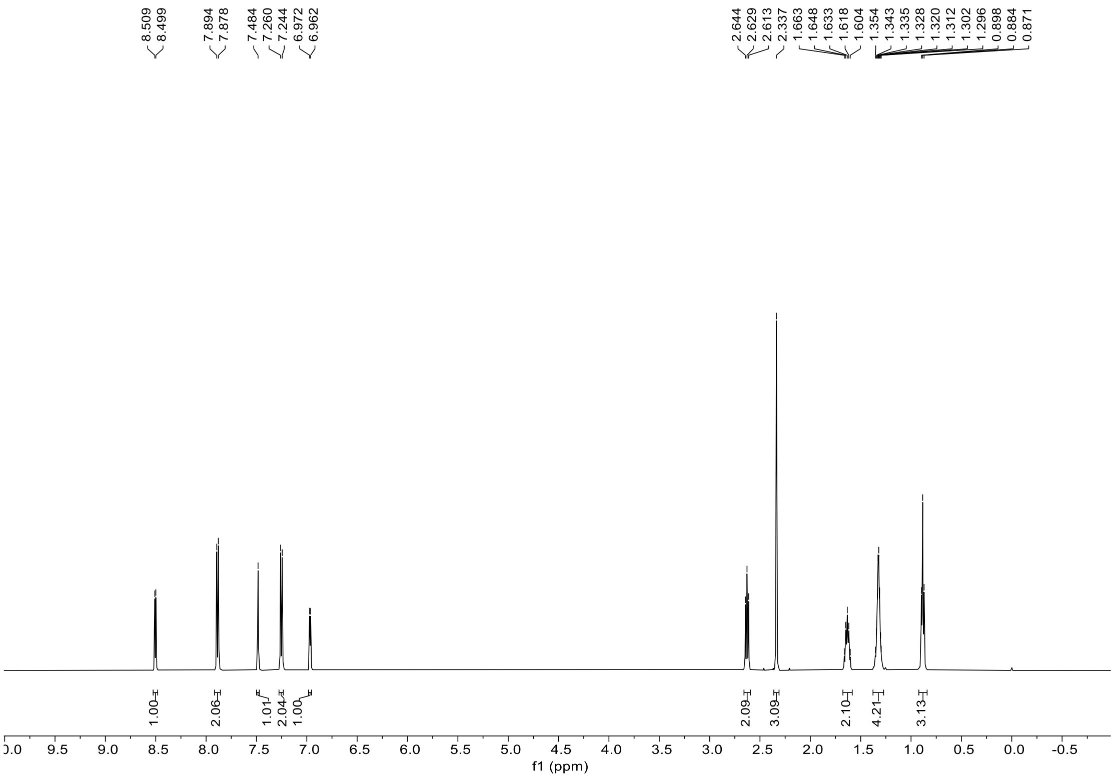

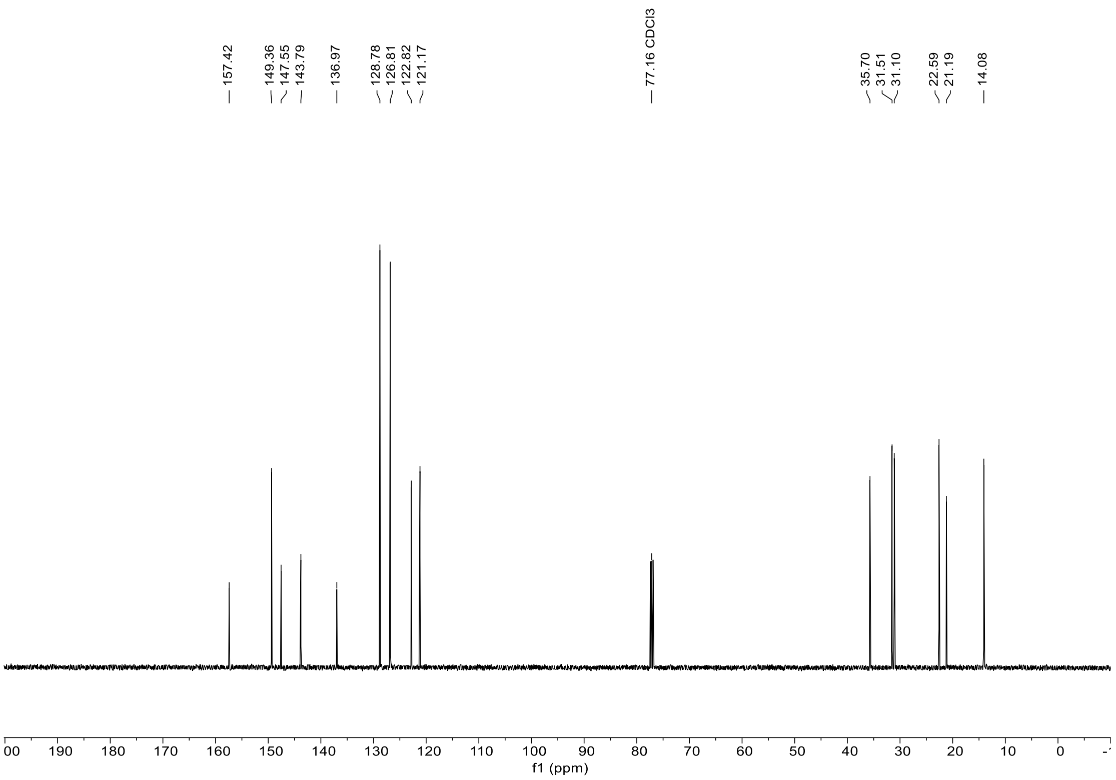

2-(4'-pentyl-[1,1'-biphenyl]-4-yl)pyridine (S7)

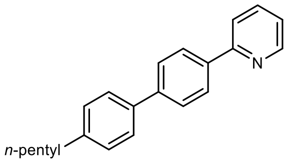

Purification by column chromatography (petroleum ether/EtOAc, 20:1) afforded the title compound (1. 10 g, 73%) as a white solid.

^{1}H NMR (500 MHz, Chloroform-*d*) δ 8.70 (d, *J* = 5.0 Hz, 1H), 8.06 (d, *J* = 8.5 Hz, 2H), 7.78 – 7.72 (m, 2H), 7.72 – 7.68 (m, 2H), 7.57 (d, *J* = 8.0 Hz, 2H), 7.29 – 7.25 (m, 2H), 7.24 – 7.20 (m, 1H), 2.69 – 2.62 (m, 2H), 1.70 – 1.62 (m, 2H), 1.39 – 1.32 (m, 4H), 0.93 – 0.89 (m, 3H).

^{13}C NMR (126 MHz, $CDCl_3$) δ 157.2, 149.8, 142.6, 141.8, 138.1, 138.0, 136.9, 129.0, 127.39, 127.38, 127.0, 122.2, 120.5, 35.7, 31.7, 31.3, 22.7, 14.2.

HRMS (ESI) for $C_{22}H_{24}N$ $[M+H]^+$ calcd. 302.1903, found 302.1898;

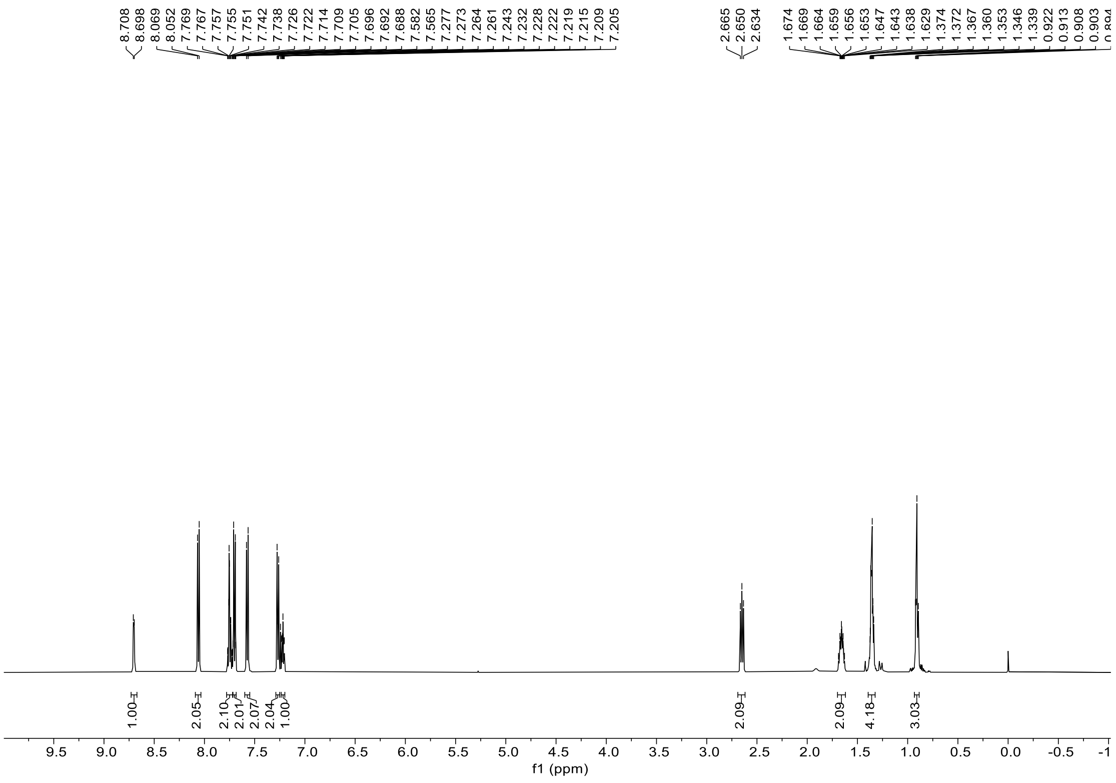

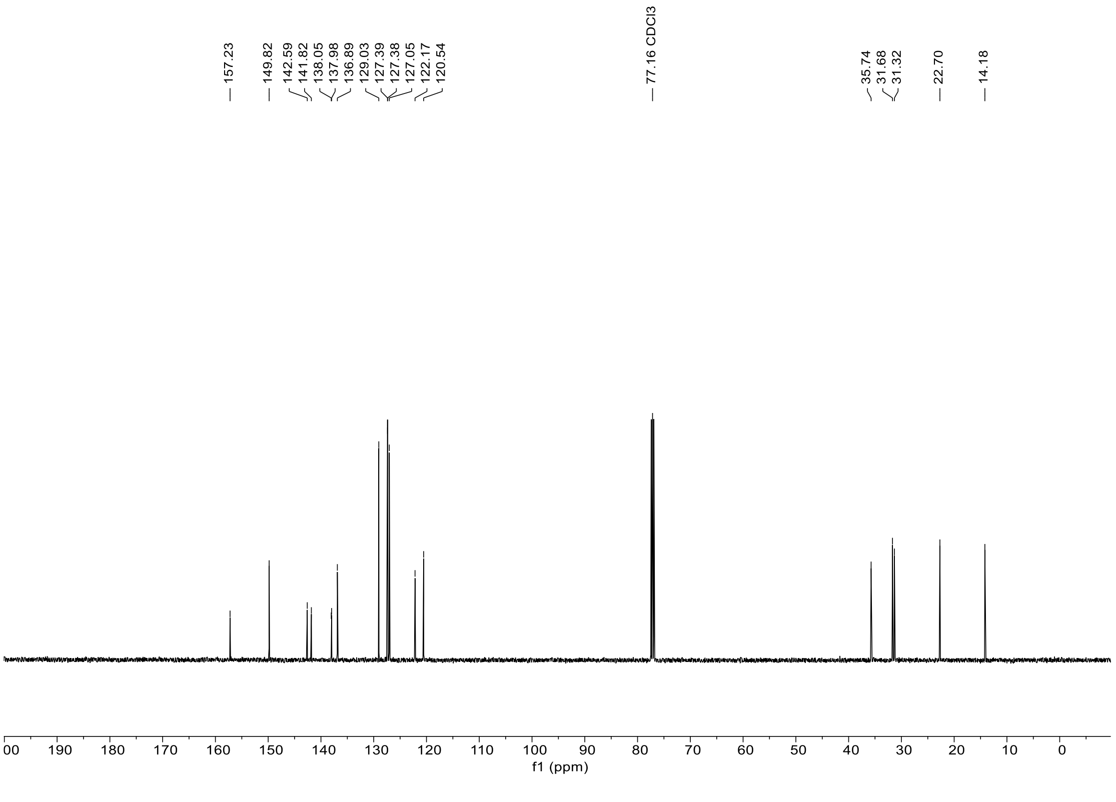

2-(4-pentylphenyl)-5-phenylpyridine (S8)

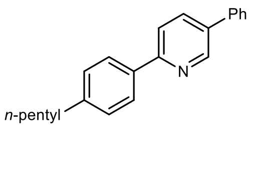

Purification by column chromatography (petroleum ether/EtOAc, 20:1) afforded the title compound (1.35 g, 90%) as a white solid.

^{1}H NMR (500 MHz, Chloroform-*d*) δ 8.96 – 8.89 (m, 1H), 7.99 – 7.93 (m, 2H), 7.93 – 7.87 (m, 1H), 7.76 (d, *J* = 8.5 Hz, 1H), 7.62 (d, *J* = 7.5 Hz, 2H), 7.48 (t, *J* = 7.5 Hz, 2H), 7.42 – 7.37 (m, 1H), 7.33 – 7.28 (m, 2H), 2.70 – 2.62 (m, 2H), 1.72 – 1.62 (m, 2H), 1.40 – 1.31 (m, 4H), 0.96 – 0.86 (m, 3H).

^{13}C NMR (126 MHz, $CDCl_3$) δ 156.3, 148.1, 144.2, 137.8, 136.5, 135.1, 134.7, 129.2, 129.0, 128.1, 127.0, 126.8, 120.2, 35.8, 31.6, 31.2, 22.7, 14.2.

HRMS (ESI) for $C_{22}H_{24}N$ $[M+H]^+$ calcd. 302.1903, found 302.1899;

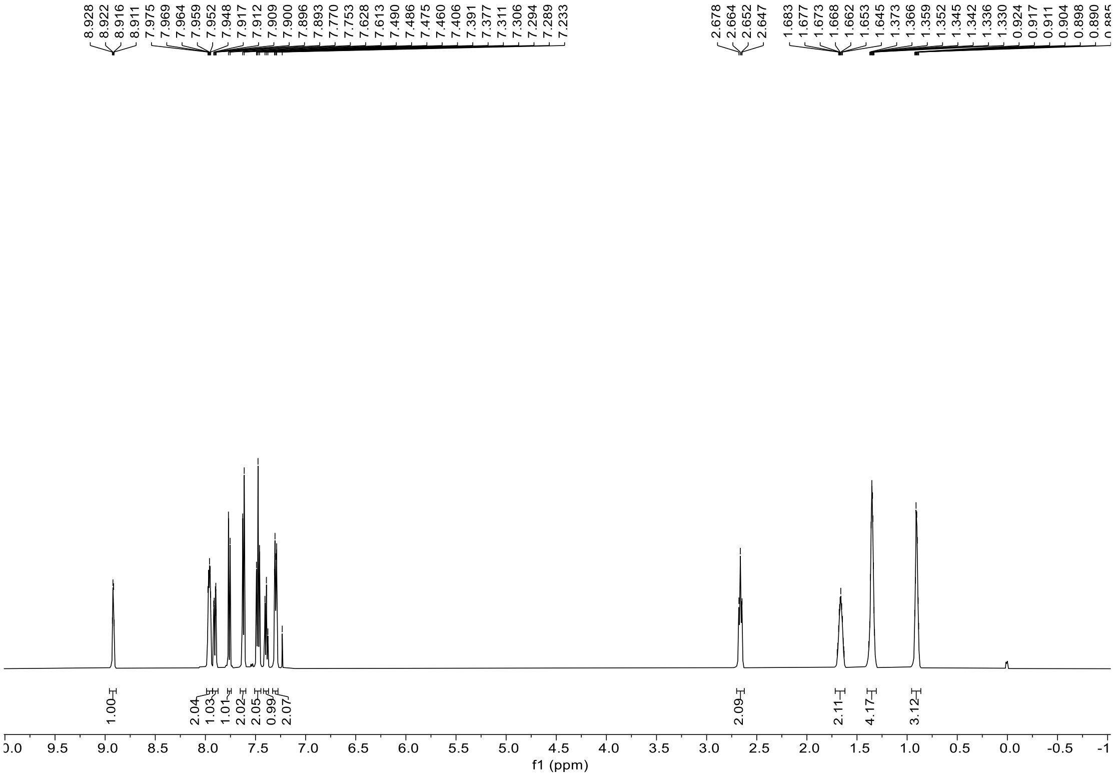

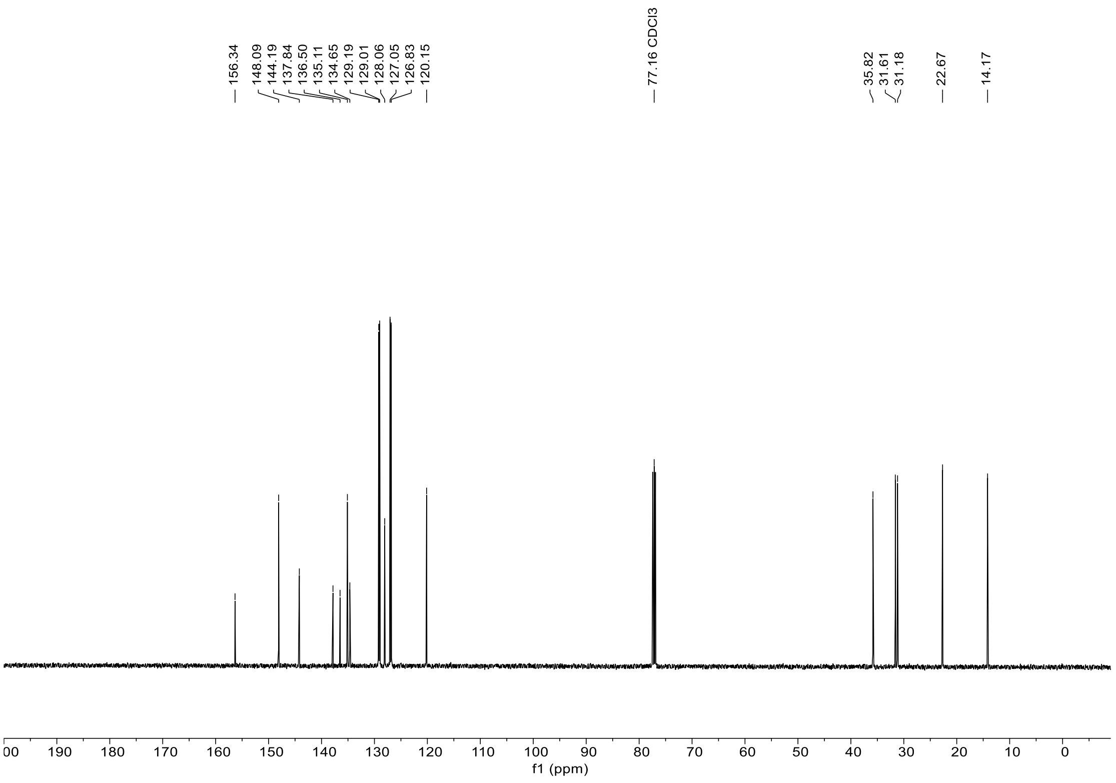

*Ligand (**S11-S12**) was prepared according to the reported procedure*[7]

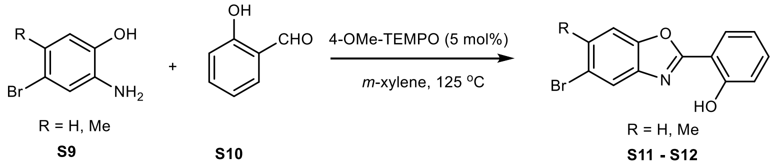

A mixture of **S9** (3.0 mmol) and salicylaldehyde **S10** (3.0 mmol) in *m*-xylene (9 mL) was heated at 120 °C for 0.5 h. 4-Methoxy-TEMPO (5 mol%) was then added to the mixture which was stirred for additional 5 h under an oxygen atmosphere. The reaction mixture was cooled to room temperature and concentrated to dryness. The product (**S11 – S12**) was purified by flash column chromatography on silica gel.

2-(5-bromobenzo[d]oxazol-2-yl)phenol (S11)

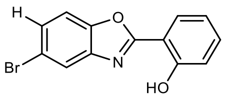

Purification by column chromatography (petroleum ether/EtOAc, 20:1) afforded the title compound (825 mg, 95%) as a white solid.

^{1}H NMR (500 MHz, Chloroform-*d*) δ 11.20 (s, 1H), 7.99 (dt, *J* = 8.0, 1.5 Hz, 1H), 7.88 – 7.84 (m, 1H), 7.51 – 7.42 (m, 3H), 7.12 (dd, *J* = 8.5, 1.0 Hz, 1H), 7.03 – 6.98 (m, 1H).

^{13}C NMR (126 MHz, $CDCl_3$) δ 164.1, 159.0, 148.3, 141.8, 134.2, 128.5, 127.4, 122.4, 119.8, 117.9, 117.7, 112.0, 110.2.

HRMS (ESI) for $C_{13}H_9BrNO_2$ $[M+H]^+$ calcd. 289.9811, found 289.9808;

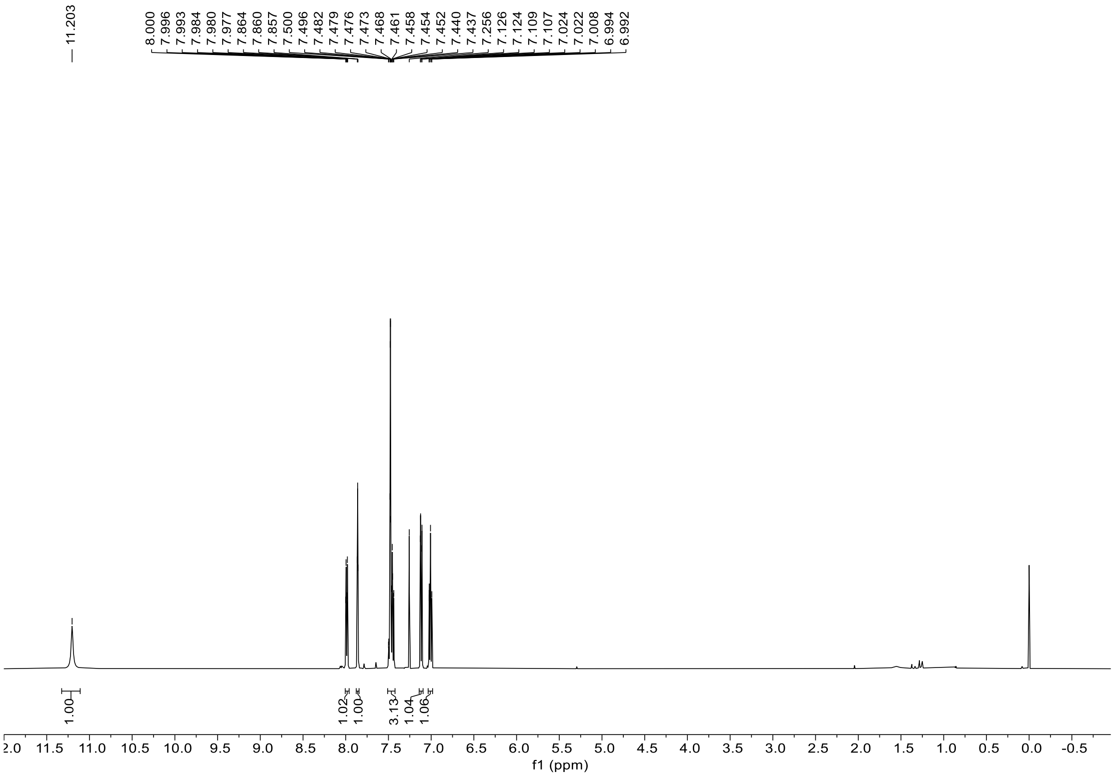

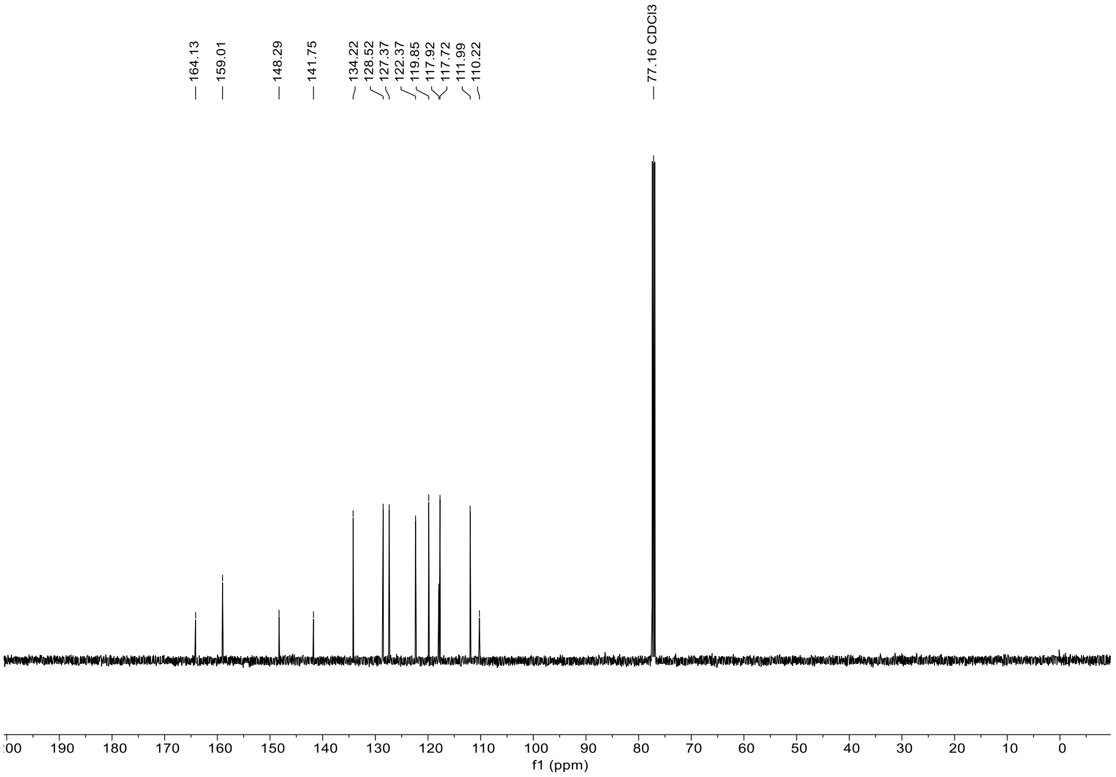

2-(5-bromo-6-methylbenzo[d]oxazol-2-yl)phenol (S12)

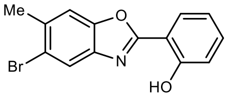

Purification by column chromatography (petroleum ether/EtOAc, 20:1) afforded the title compound (821 mg, 90%) as a white solid.

^{1}H NMR (500 MHz, Chloroform-*d*) δ 11.24 (s, 1H), 7.97 (dd, *J* = 8.0, 1.5 Hz, 1H), 7.89 (s, 1H), 7.48 (s, 1H), 7.46 – 7.42 (m, 1H), 7.11 (dd, *J* = 8.5, 1.0 Hz, 1H), 7.02 – 6.98 (m, 1H), 2.53 (s, 3H).

^{13}C NMR (126 MHz, $CDCl_3$) δ 163.5, 158.9, 148.7, 139.6, 135.4, 133.9, 127.2, 122.5, 120.7, 119.8, 117.6, 112.1, 110.4, 23.9.

HRMS (ESI) for $C_{14}H_{11}BrNO_2$ $[M+H]^+$ calcd. 303.9968, found 303.9967;

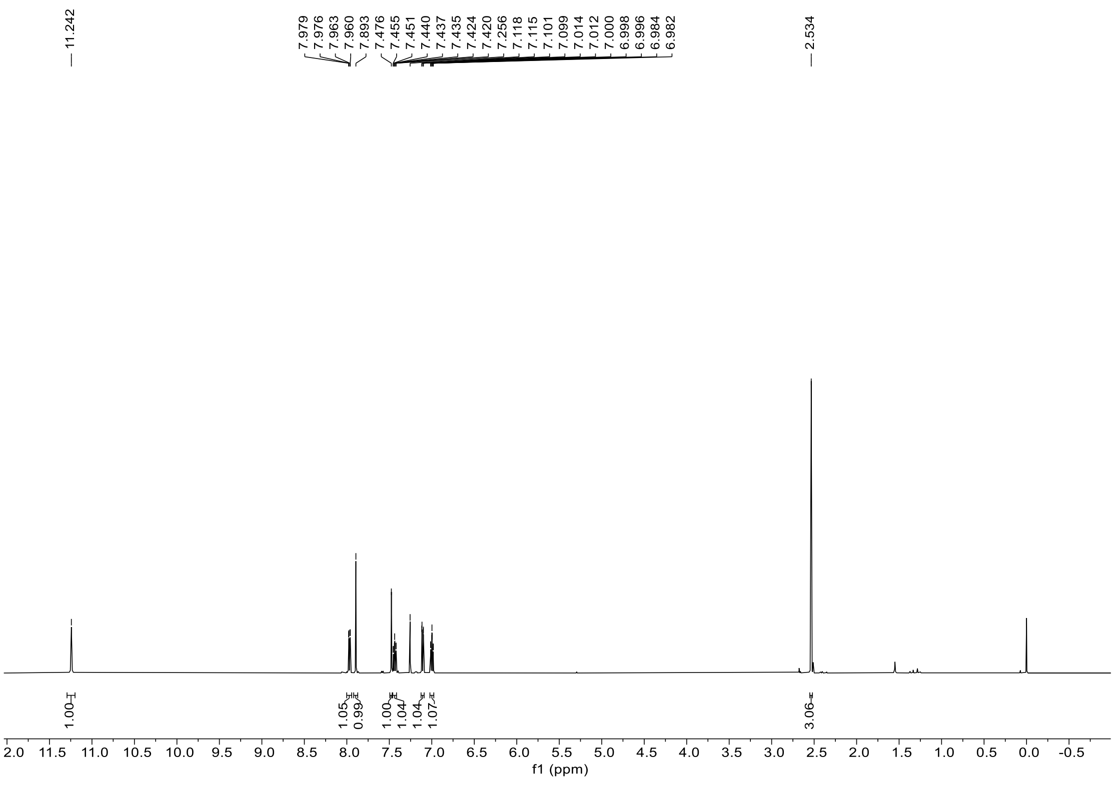

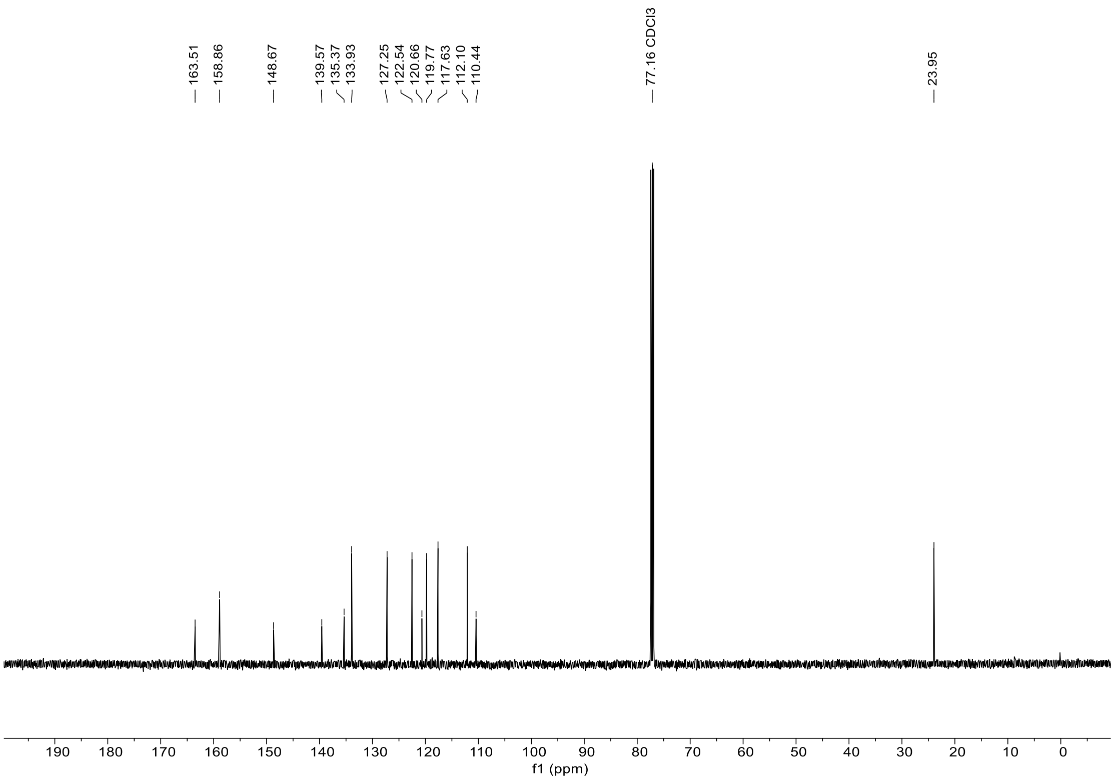

*Experimental Procedure for synthesizing **S14***

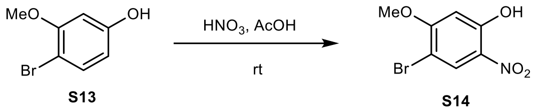

To a solution of 4-bromo-3-methoxyphenol **S13** (10 mmol, 2.03g) in AcOH (30 mL) was added conc. HNO_3 (1.7 g) dropwise. The temperature was maintained at 15-20 °C and the mixture was stirred for 1 h and then poured into water (20 mL) and extracted with EtOAc. The organic layer was washed with brine, dried over Na_2SO_4 and concentrated. The crude product was purified by flash column chromatography on silica gel (petroleum ether/EtOAc = 10:1) to give 4-bromo-5-methoxy-2-nitrophenol **S14** (1.14 g, 46% yield, yellow solid).

^{1}H NMR (400 MHz, $CDCl_3$) δ 10.94 (s, 1H), 8.31 (s, 1H), 6.57 (s, 1H), 3.98 (s, 3H).

^{13}C NMR (101 MHz, $CDCl_3$) δ 163.1, 157.1, 129.4, 127.8, 103.4, 101.0, 57.3.

HRMS (ESI) for $C_7H_5BrNO_4$ $[M-H]^-$ calcd. 245.9407, found 245.9410;

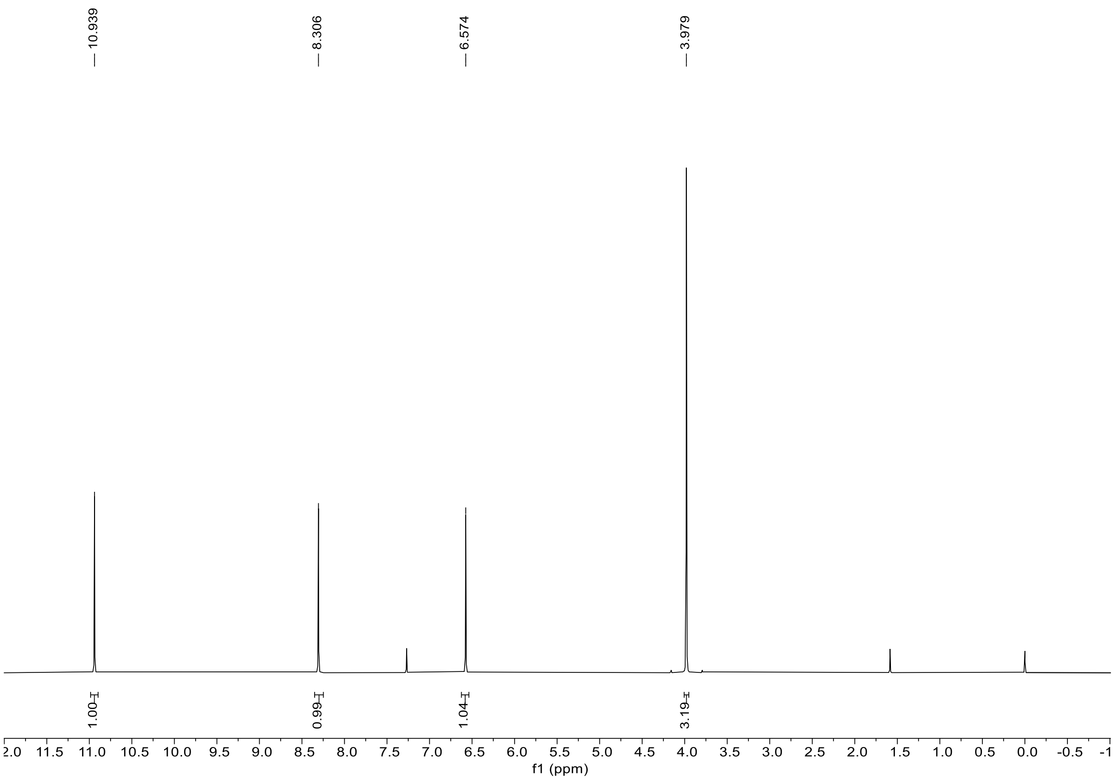

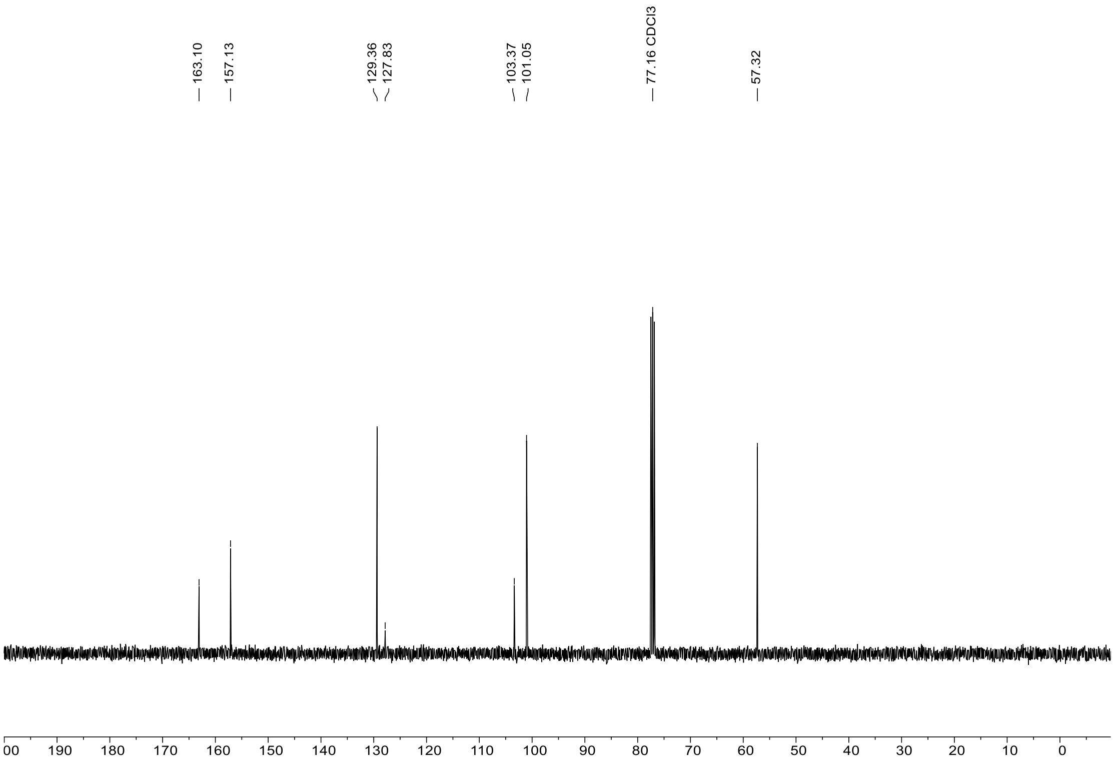

*Experimental Procedure for synthesizing **26, S17, S19, 27***

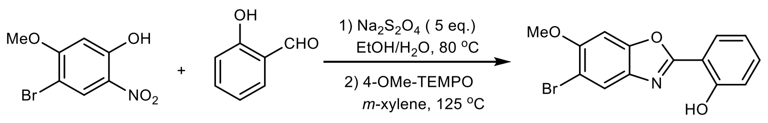

S14 **S10** **26**

A solution of 4-bromo-5-methoxy-2-nitrophenol **S14** (1.0 mmol) and salicylaldehyde **S10** (1.0 mmol) in EtOH/H_2O (7 mL/3 mL) was heated at 80 °C for 10 min. $Na_2S_2O_4$ (5 equiv.) was then added to the mixture which was stirred for additional 20 min, and the progress of the reaction was monitored by using TLC. After the reaction was completed, EtOAc was added and the solution was filtered. The organic layer was washed with brine, dried over Na_2SO_4 and concentrated. Added 4-Methoxy-TEMPO (5 mol%) and *m*-xylene (10 mL) to the obtained dark red crude product, and heat under reflux for 12 h in oxygen atmosphere. The reaction mixture was cooled to room temperature and concentrated to dryness. The product was purified by flash column chromatography on silica gel (petroleum ether/EtOAc = 10:1), which afforded the compound **26** (141 mg, 44% yield, white solid).

^{1}H NMR (400 MHz, $CDCl_3$) δ 11.15 (s, 1H), 7.93 (dd, *J* = 8.0, 1.6 Hz, 1H), 7.89 (s, 1H), 7.44 – 7.40 (m, 1H), 7.15 (s, 1H), 7.10 (dd, *J* = 8.4, 1.2 Hz, 1H), 7.01 – 6.97 (m, 1 H), 3.97 (s, 3H).

^{13}C NMR (101 MHz, $CDCl_3$) δ 162.8, 158.4, 154.5, 149.3, 134.4, 133.6, 126.9, 123.1, 119.8, 117.6, 110.6, 108.9, 94.9, 57.0.

HRMS (ESI) for $C_{14}H_{11}BrNO_3$ $[M+H]^+$ calcd. 319.9917, found 319.9912;

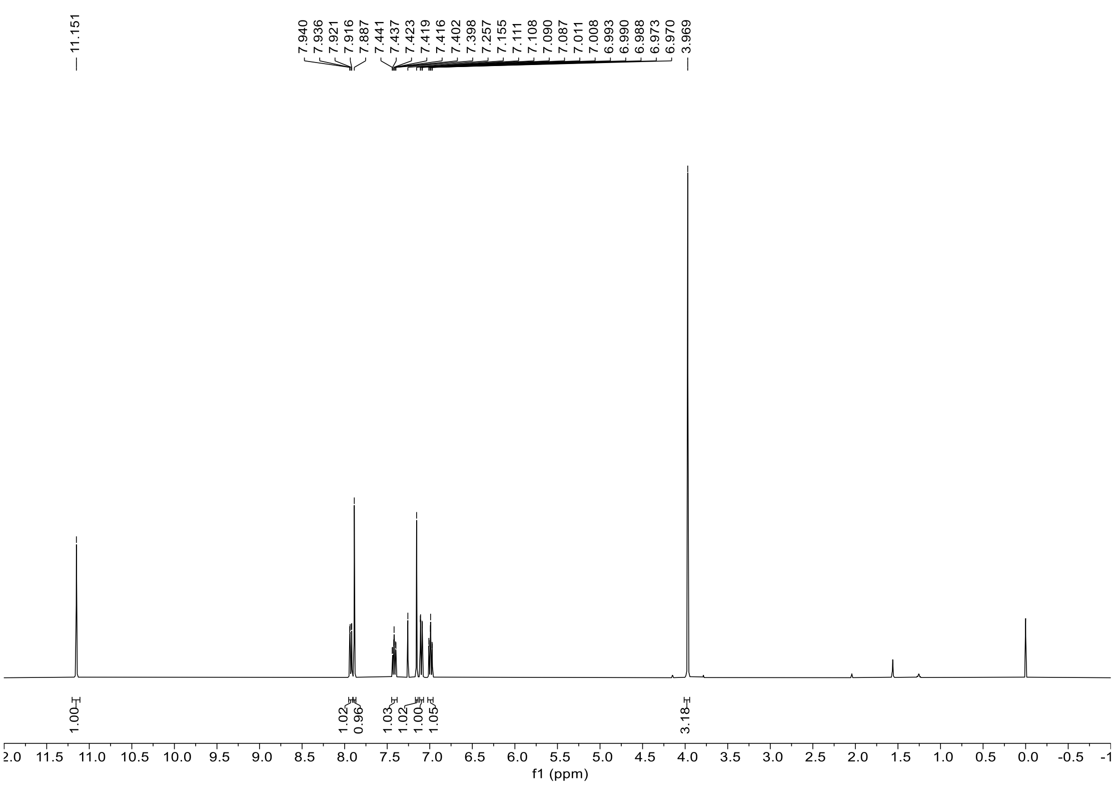

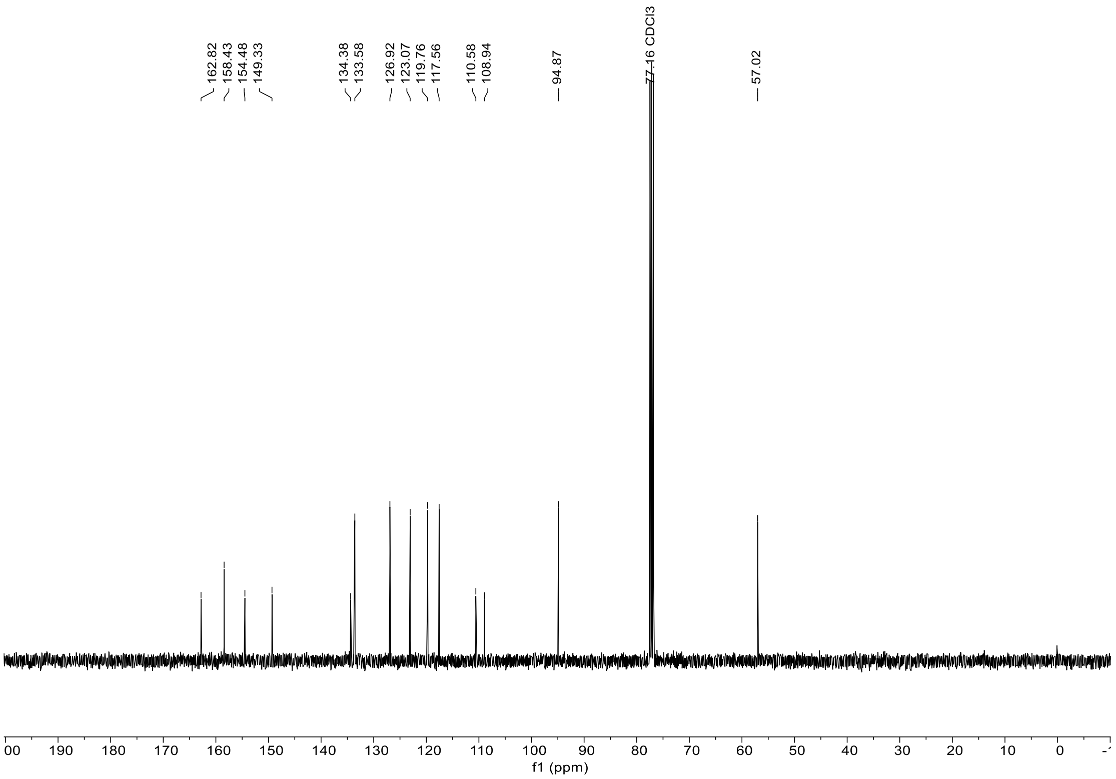

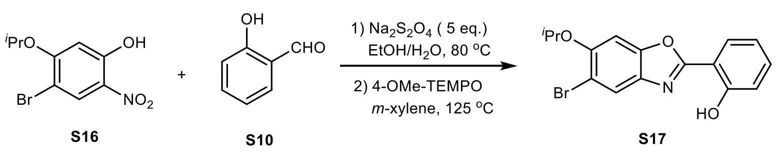

A solution of 4-bromo-5-isopropoxy-2-nitrophenol **S16**[8] (1.0 mmol) and salicylaldehyde **S10**

(1.0 mmol) in EtOH/H_2O (7 mL/3 mL) was heated at 80 °C for 10 min. $Na_2S_2O_4$ (5 equiv.) was then added to the mixture which was stirred for additional 20 min, and the progress of the reaction was monitored by using TLC. After the reaction was completed, EtOAc was added and the solution was filtered. The organic layer was washed with brine, dried over Na_2SO_4 and concentrated. Added 4-Methoxy-TEMPO (5 mol%) and *m*-xylene (10 mL) to the obtained dark red crude product, and heat under reflux for 12 h in oxygen atmosphere. The reaction mixture was cooled to room temperature and concentrated to dryness. The product was purified by flash column chromatography on silica gel (petroleum ether/EtOAc = 10:1), which afforded the compound **S17** (167 mg, 48% yield, white solid).

^{1}H NMR (500 MHz, Chloroform-*d*) δ 11.18 (s, 1H), 7.93 (dd, *J* = 7.5, 1.5 Hz, 1H), 7.88 (s, 1H), 7.46 – 7.38 (m, 1H), 7.17 (s, 1H), 7.10 (d, *J* = 8.5 Hz, 1H), 6.99 (t, *J* = 7.5 Hz, 1H), 4.59 (hept, *J* = 6.0 Hz, 1H), 1.44 (d, *J* = 6.0 Hz, 6H).

^{13}C NMR (126 MHz, $CDCl_3$) δ 162.9, 158.5, 152.9, 149.2, 134.6, 133.6, 126.9, 123.0, 119.7, 117.5, 111.0, 110.6, 98.3, 73.4, 22.1.

HRMS (ESI) for $C_{16}H_{15}BrNO_3$ $[M+H]^+$ calcd. 348.0230, found 348.0227;

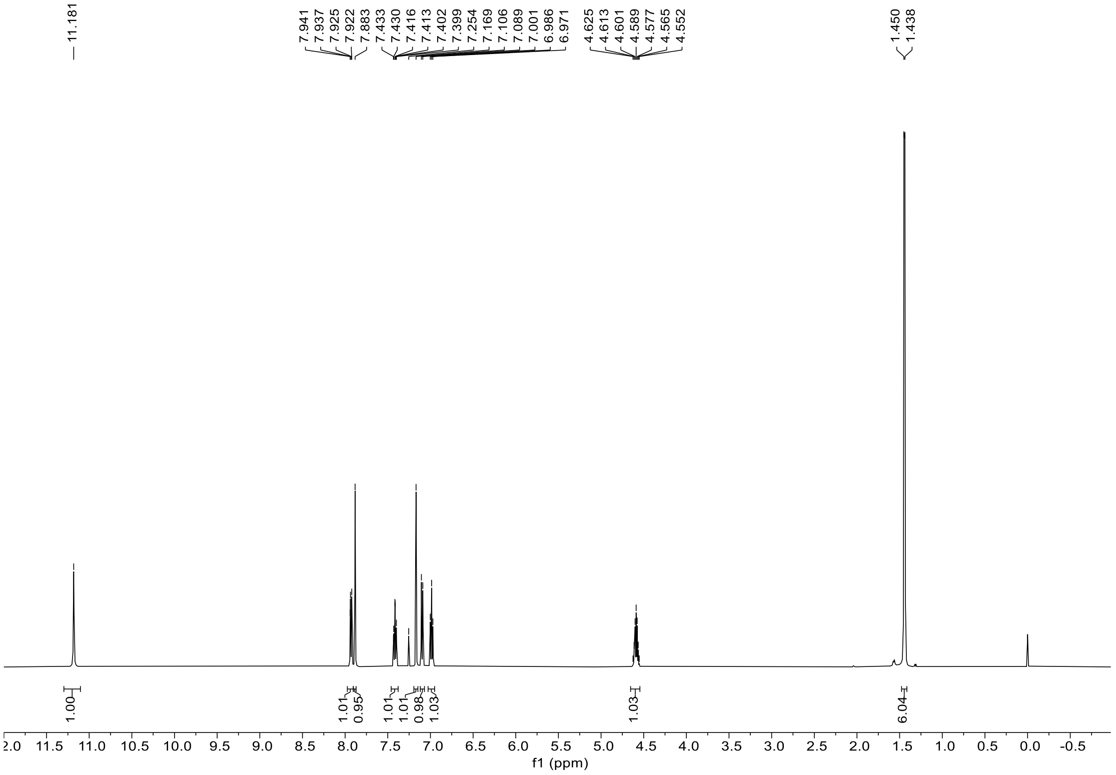

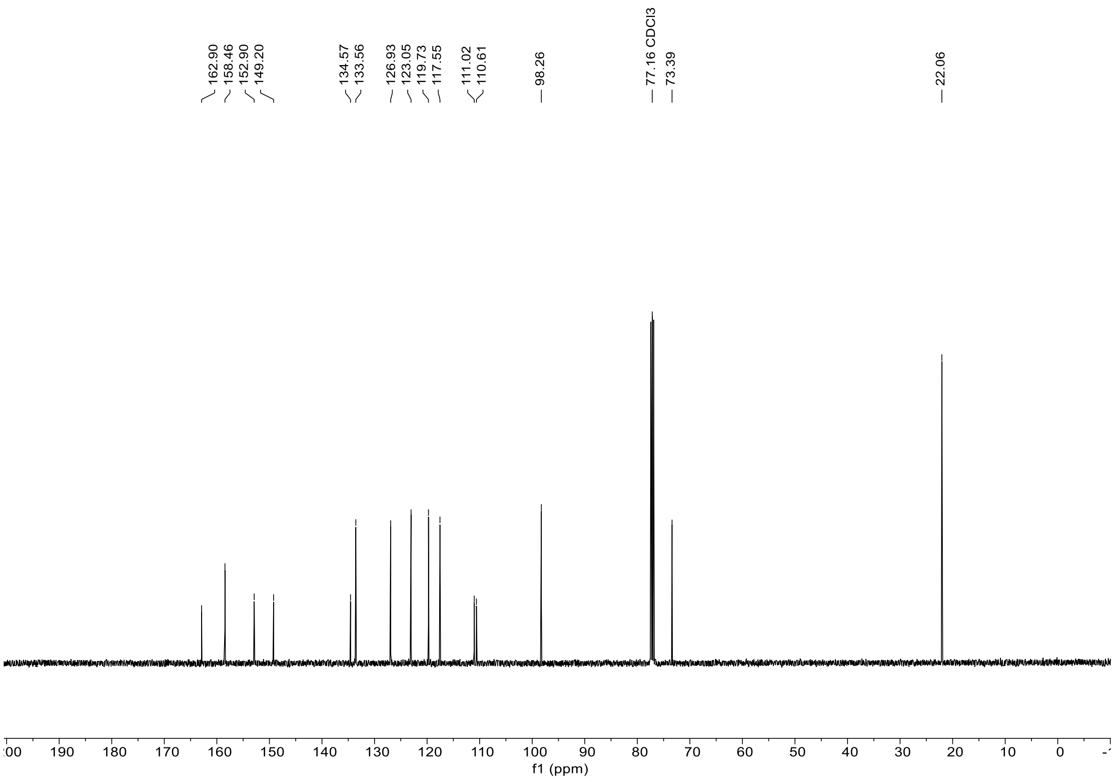

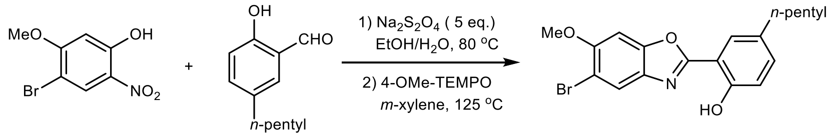

S14 **S18** **S19**

A solution of 4-bromo-5-methoxy-2-nitrophenol **S14** (1.0 mmol) and 2-hydroxy-5-pentylbenzaldehyde **S18**[9] (1.0 mmol) in EtOH/H_2O (7 mL/3 mL) was heated at 80 °C for 10 min. $Na_2S_2O_4$ (5 equiv.) was then added to the mixture which was stirred for additional 20 min, and the progress of the reaction was monitored by TLC. After the reaction was completed, EtOAc was added and the mixture was filtered. The organic layer was washed with brine, dried over Na_2SO_4 and concentrated. Added 4-Methoxy-TEMPO (5 mol%) and *m*-xylene (10 mL) to the obtained dark red crude product, and heat under reflux for 12 h in oxygen atmosphere. The reaction mixture was cooled to room temperature and concentrated to dryness. The product was purified by flash column chromatography on silica gel (petroleum ether/EtOAc = 10:1), which afforded the compound **S19** (230 mg, 59% yield, white solid).

^{1}H NMR (500 MHz, $CDCl_3$) δ 10.98 (s, 1H), 7.90 (s, 1H), 7.75 (d, *J* = 2.0 Hz, 1H), 7.24 (dd, *J* = 8.5, 2.5 Hz, 1H), 7.18 (s, 1H), 7.02 (d, *J* = 8.5 Hz, 1H), 3.98 (s, 3H), 2.61 (t, *J* = 7.5 Hz, 2H), 1.64 (p, *J* = 7.5 Hz, 2H), 1.38 – 1.32 (m, 4H), 0.91 (t, *J* = 6.5 Hz, 3H).

^{13}C NMR (126 MHz, $CDCl_3$) δ 163.1, 156.6, 154.4, 149.3, 134.6, 134.2, 134.0, 126.2, 123.1, 117.4, 110.2, 108.9, 94.9, 57.0, 35.1, 31.5, 31.4, 22.7, 14.2.

HRMS (ESI) for $C_{19}H_{21}BrNO_3$ $[M+H]^+$ calcd. 390.0700, found 390.0699;

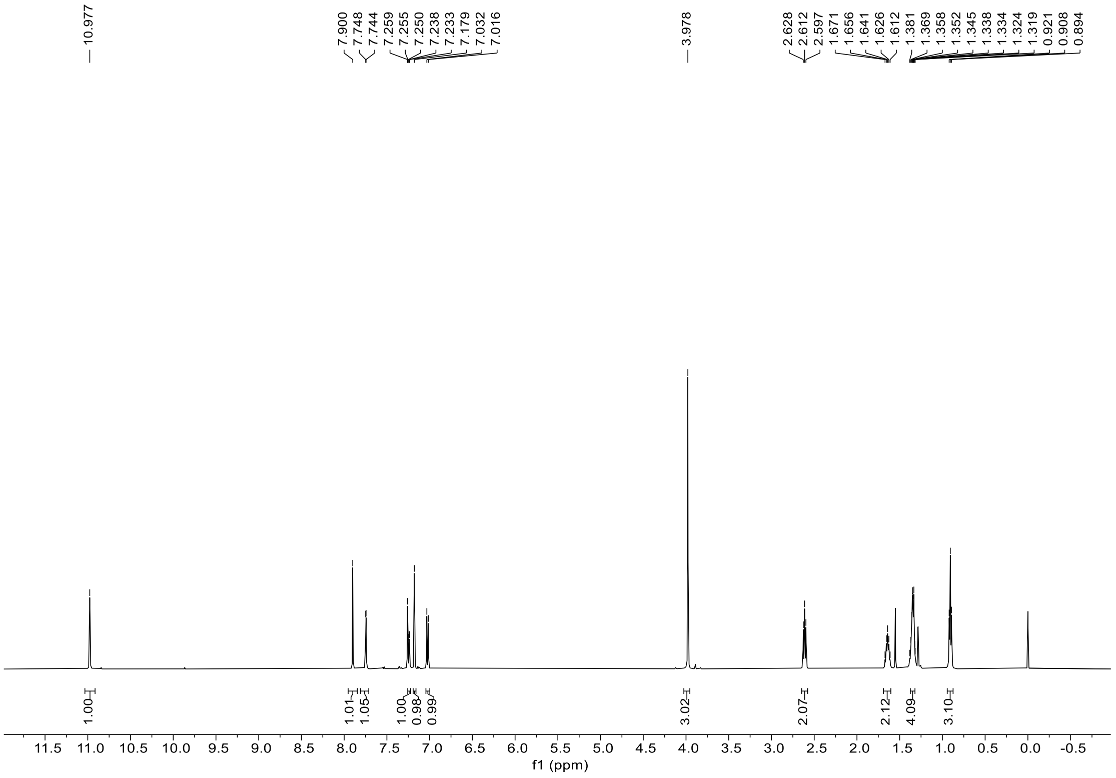

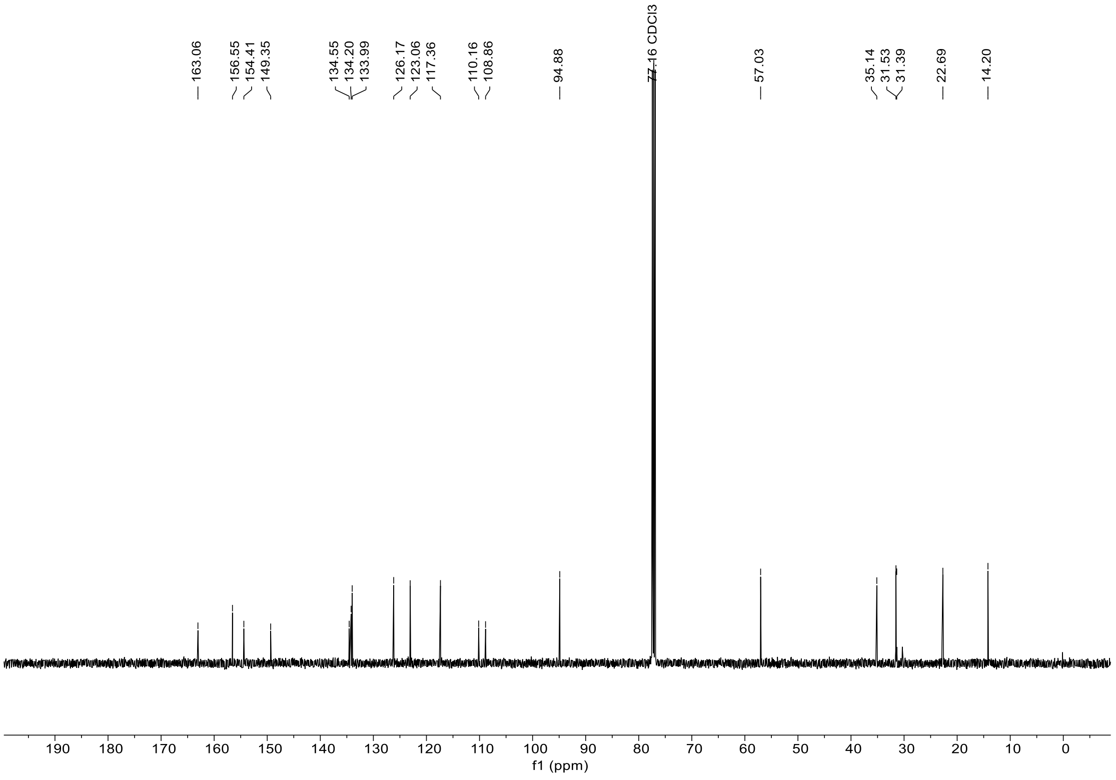

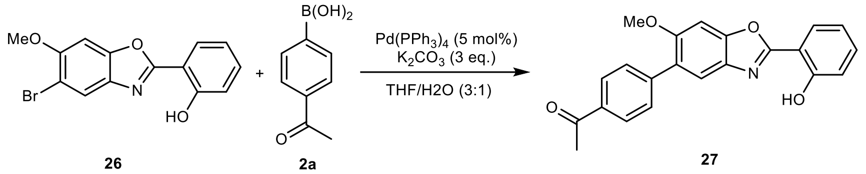

A dried 10 mL Schlenk tube was charged with the **26** (0.1 mmol, 1.0 equiv.), (4-acetylphenyl)boronic acid (0.2 mmol, 2 equiv.), $Pd(PPh_3)_4$ (5 mol%), K_2CO_3 (0.06 mmol) and THF/H_2O (v/v, 3:1, 1 mL) in glovebox. The mixture was stirred at 90°C for 12 h. After achieving appropriate conversion, the mixture was concentrated under vacuum to give a residue, which was purified by flash column chromatography on silica gel to give the **27** (32.1 mg, 90% yield, white solid).

^{1}H NMR (500 MHz, Chloroform-*d*) δ 11.28 (s, 1H), 8.10 – 7.90 (m, 3H), 7.75 – 7.56 (m, 3H), 7.43 (t, *J* = 8.0 Hz, 1H), 7.27 – 7.18 (m, 1H), 7.12 (d, *J* = 8.0 Hz, 1H), 7.01 (t, *J* = 7.5 Hz, 1H), 3.89 (s, 3H), 2.65 (s, 3H).

^{13}C NMR (126 MHz, $CDCl_3$) δ 197.8, 162.4, 158.3, 155.5, 149.9, 143.1, 135.8, 133.7, 133.3, 130.0, 129.0, 128.2, 128.0, 127.5, 126.8, 120.4, 119.6, 117.4, 110.7, 94.2, 56.3, 26.7.

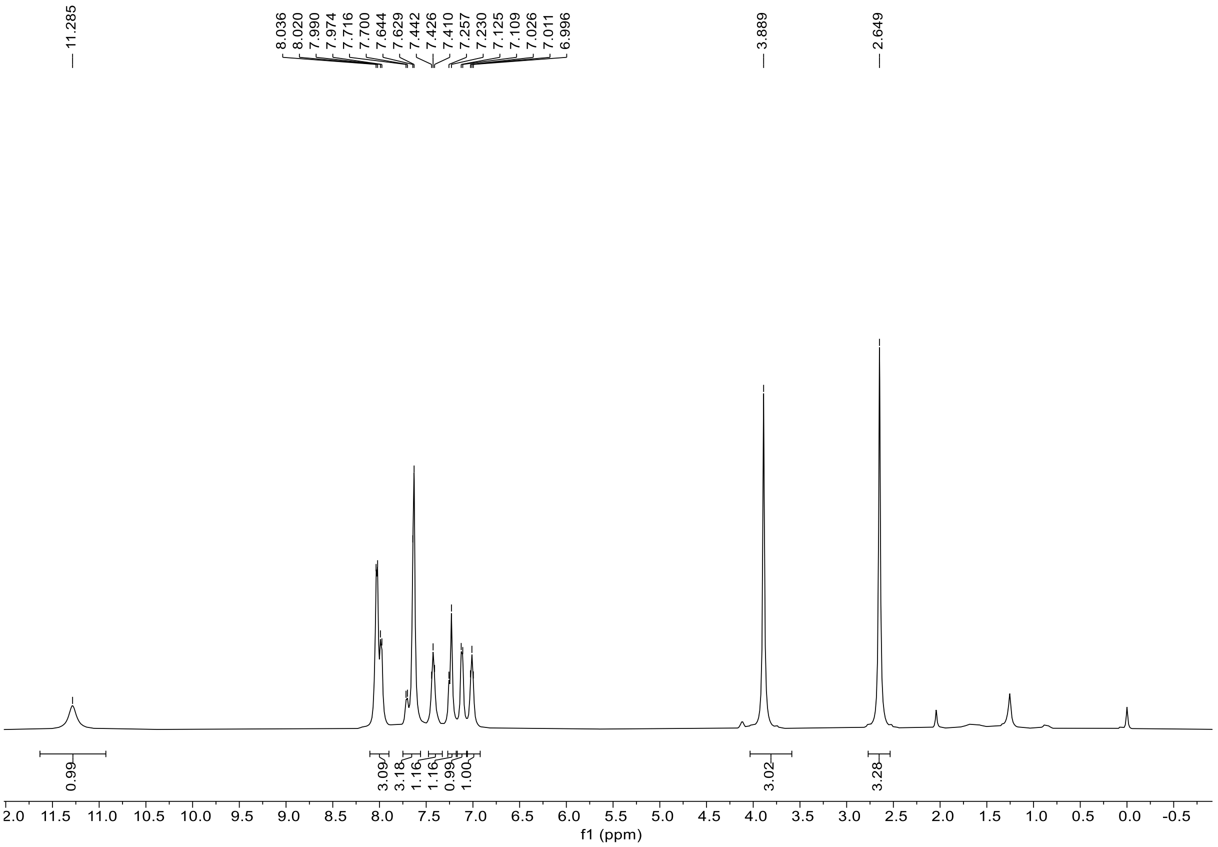

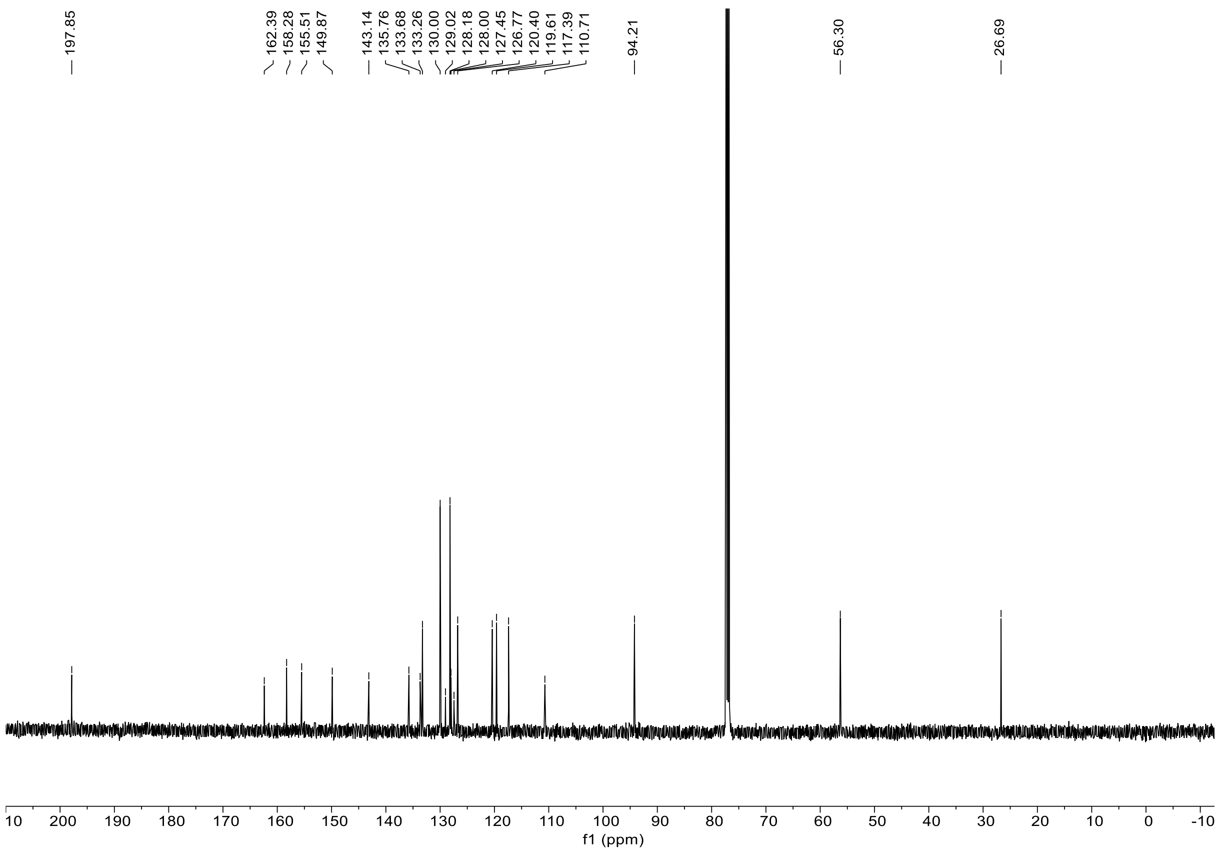

***Ir complexes** was prepared according to the modified procedure*[10]

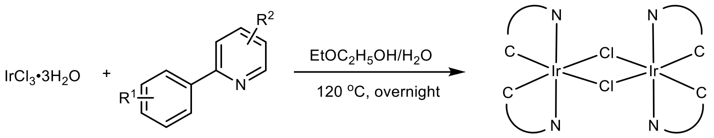

Under air, a three-neck round-bottom flask was charged with $IrCl_3 \cdot 3H_2O$ (1 mmol, 1.0 equiv.) and mixture of 2-ethoxyethanol/water (2/1 v/v, 10 mL, 0.1 M), followed by addition of cyclometalated ligands (2 equiv.). The reaction mixture was sparged with nitrogen for 30 minutes and heated to 120 °C for 12 hours. The reaction mixture was cooled to room temperature and then concentrated under vacuum. The crude solid was taken to the next step without further purification.

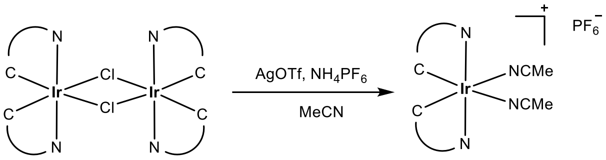

Under air, a round-bottom flask was charged with crude product (dimer-Ir), AgOTf (1 mmol), and MeCN (30 mL). The reaction was placed under nitrogen, protected from light and stirred at 60 °C for 4 hours, NH_4PF_6 (10 equiv.) was then added to the mixture which was stirred for

additional 30 minutes. The suspension was filtered over Celite to remove AgCl, and the filtrate was concentrated to give a pale-yellow solid. This crude solid was taken to the next step without further purification.

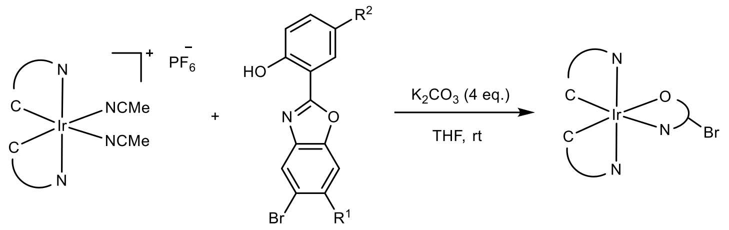

Under air, a round-bottom flask was charged with Iridium acetonitrile complex (0.1 mmol, 1 equiv.), phenol benzoxazole ligand (1 equiv.) and THF (5 mL). The reaction solution was stirred at room temperature for 8 hours. The solvent was removed under vacuum, the crude product was purified by flash column chromatography on silica gel (petroleum ether/EtOAc).

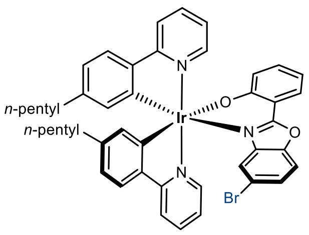

Analytical data of **1a:**

^{1}H NMR (500 MHz, Chloroform-*d*) δ 8.82 – 8.76 (m, 1H), 8.02 (d, *J* = 5.5 Hz, 1H), 7.90 (d, *J* = 8.0 Hz, 1H), 7.69 (dd, *J* = 14.5, 8.0 Hz, 2H), 7.59 – 7.46 (m, 3H), 7.44 – 7.37 (m, 1H), 7.22 – 7.11 (m, 3H), 6.93 (t, *J* = 6.5 Hz, 1H), 6.80 – 6.70 (m, 3H), 6.63 (d, *J* = 8.0 Hz, 1H), 6.44 – 6.37 (m, 1H), 6.24 – 6.19 (m, 1H), 6.04 – 5.93 (m, 2H), 2.38 – 2.23 (m, 4H), 1.49 – 1.33 (m, 4H), 1.22 – 1.07 (m, 8H), 0.81 – 0.72 (m, 6H).

^{13}C NMR (126 MHz, $CDCl_3$) δ 170.1, 168.9, 168.6, 161.0, 150.9, 148.9, 148.7, 148.3, 148.1, 144.6, 143.9, 142.6, 142.5, 142.0, 136.7, 136.6, 133.9, 133.7, 131.8, 128.8, 127.2, 126.2, 124.5, 123.8, 122.3, 121.7, 121.6, 121.2, 121.1, 118.6, 117.9, 117.2, 113.6, 110.8, 109.9, 36.1, 36.0, 31.7, 31.6, 31.0, 30.3, 22.62, 22.6, 14.2.

HRMS (ESI) for $C_{45}H_{44}BrIrN_3O_2$ $[M+H]^+$ calcd. 930.2241, found 930.2244;

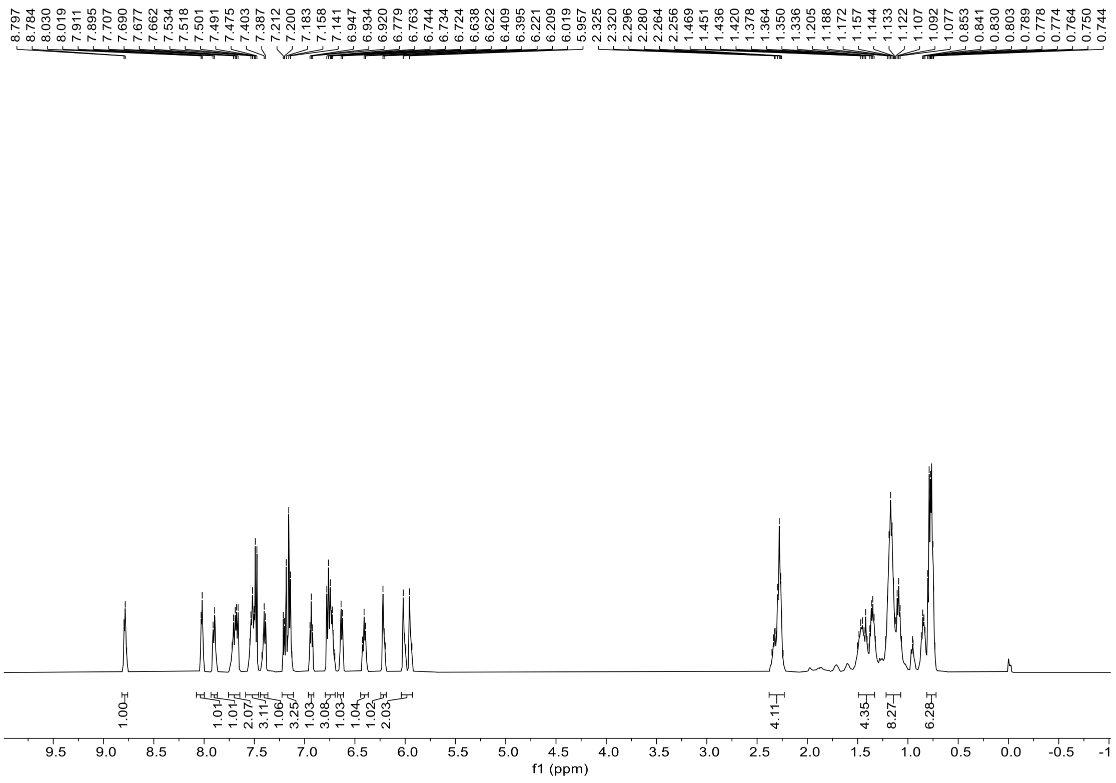
8.797 8.784 8.030 8.019 7.911 7.895 7.707 7.690 7.677 7.662 7.534 7.518 7.501 7.491 7.475 7.403 7.387 7.212 7.200 7.183 7.158 7.141 6.947 6.934 6.920 6.779 6.763 6.744 6.734 6.724 6.638 6.622 6.409 6.395 6.221 6.209 6.019 5.957 2.325 2.320 2.296 2.280 2.264 2.256 1.469 1.451 1.436 1.420 1.378 1.364 1.350 1.336 1.205 1.188 1.172 1.157 1.144 1.133 1.122 1.107 1.092 1.077 0.853 0.841 0.830 0.803 0.789 0.778 0.774 0.764 0.750 0.744
1.00 1.01 1.01 2.07 3.11 1.06 3.25 1.03 3.08 1.03 1.04 1.02 2.03 4.11 4.35 8.27 6.28
9.5 9.0 8.5 8.0 7.5 7.0 6.5 6.0 5.5 5.0 4.5 4.0 3.5 3.0 2.5 2.0 1.5 1.0 0.5 0.0 -0.5 -1
f1 (ppm)

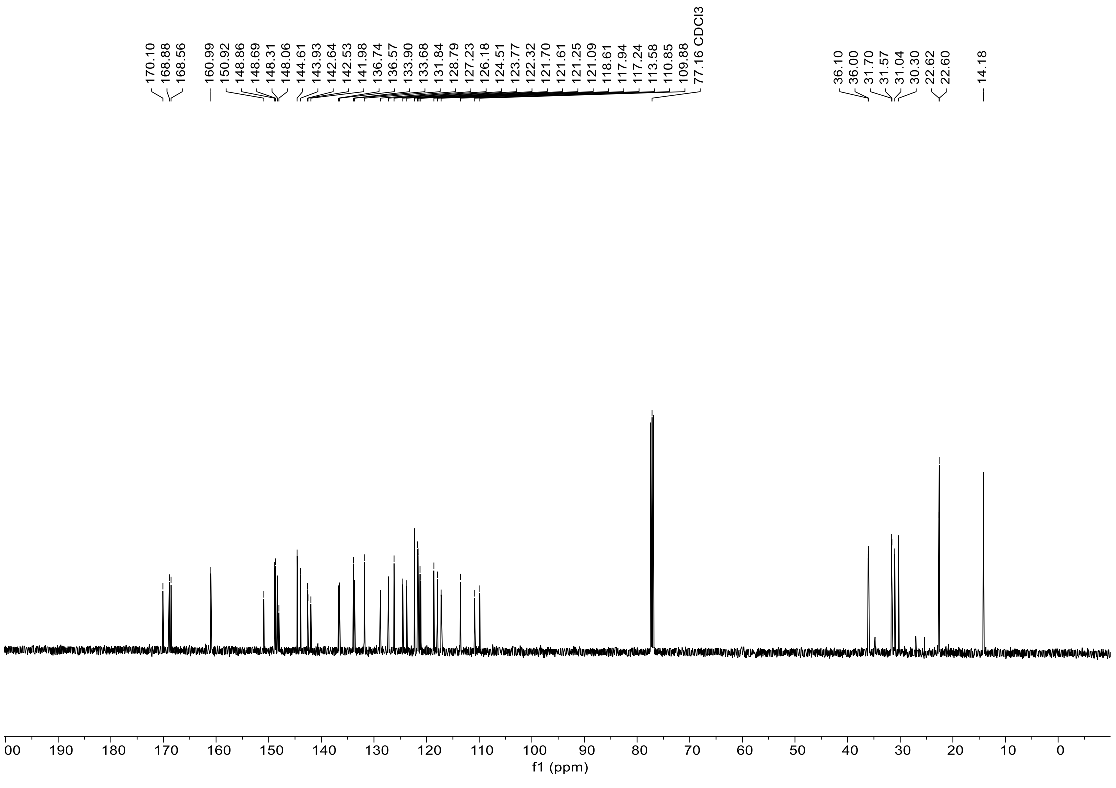
170.10 168.88 168.56 160.99 150.92 148.86 148.69 148.31 148.06 144.61 143.93 142.64 142.53 141.98 136.74 136.57 133.90 133.68 131.84 128.79 127.23 126.18 124.51 123.77 122.32 121.70 121.61 121.25 121.09 118.61 117.94 117.24 113.58 110.85 109.88
77.16 CDCl3
36.10 36.00 31.70 31.57 31.04 30.30 22.62 22.60 14.18
00 190 180 170 160 150 140 130 120 110 100 90 80 70 60 50 40 30 20 10 0
f1 (ppm)

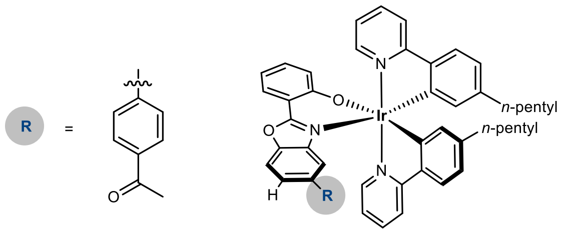

Analytical data of **3a:**

^{1}H NMR (500 MHz, Chloroform-*d*) δ 8.88 (dd, *J* = 6.0, 1.5 Hz, 1H), 8.11 (dd, *J* = 6.0, 1.5 Hz, 1H), 7.97 (dd, *J* = 8.0, 2.0 Hz, 1H), 7.92 – 7.85 (m, 2H), 7.79 – 7.72 (m, 2H), 7.63 – 7.53 (m, 3H), 7.46 (dd, *J* = 15.5, 8.0 Hz, 2H), 7.39 (dd, *J* = 8.5, 1.8 Hz, 1H), 7.23 – 7.13 (m, 3H), 7.04 – 6.97 (m, 1H), 6.82 – 6.72 (m, 3H), 6.69 – 6.62 (m, 2H), 6.49 – 6.42 (m, 1H), 6.24 (d, *J* = 1.5 Hz, 1H), 5.92 (d, *J* = 1.5 Hz, 1H), 2.61 (s, 3H), 2.33 – 2.17 (m, 4H), 1.40 – 1.31 (m, 2H), 1.23 – 0.90 (m, 10H), 0.80 (t, *J* = 7.5 Hz, 3H), 0.65 (t, *J* = 7.0 Hz, 3H).

^{13}C NMR (126 MHz, $CDCl_3$) δ 197.8, 169.9, 169.0, 168.6, 160.9, 151.1, 149.5, 149.03, 148.96, 148.8, 144.8, 144.4, 144.0, 142.7, 142.4, 141.9, 136.8, 136.6, 136.3, 135.7, 133.9, 133.7, 131.7, 128.8, 128.6, 127.6, 125.9, 124.5, 123.7, 121.8, 121.4, 121.24, 121.21, 118.6, 118.0, 117.9, 113.7, 110.3, 110.2, 36.01, 35.99, 31.5, 31.4, 30.5, 30.3, 26.8, 22.6, 22.5, 14.2, 14.1.

HRMS (ESI) for $C_{53}H_{51}IrN_3O_3$ $[M+H]^+$ calcd. 970.3554, found 970.3547;

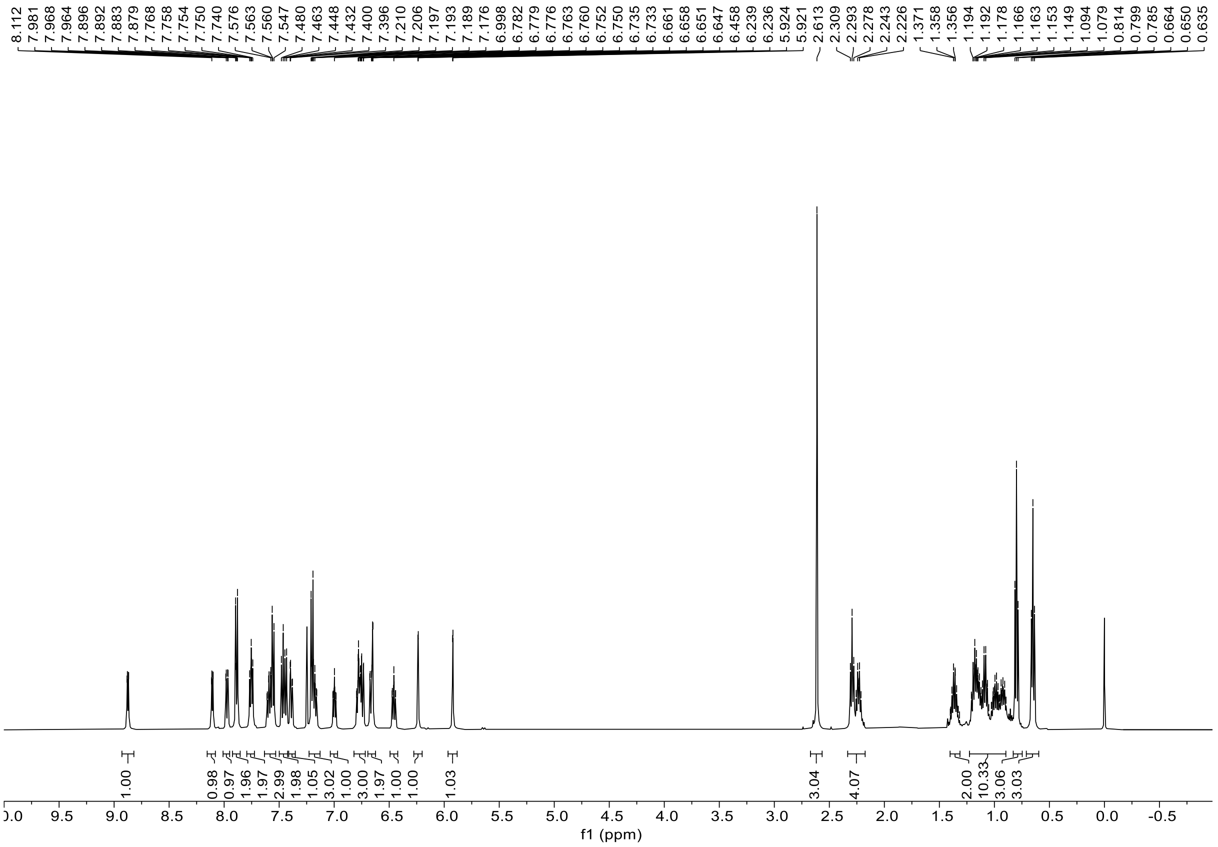

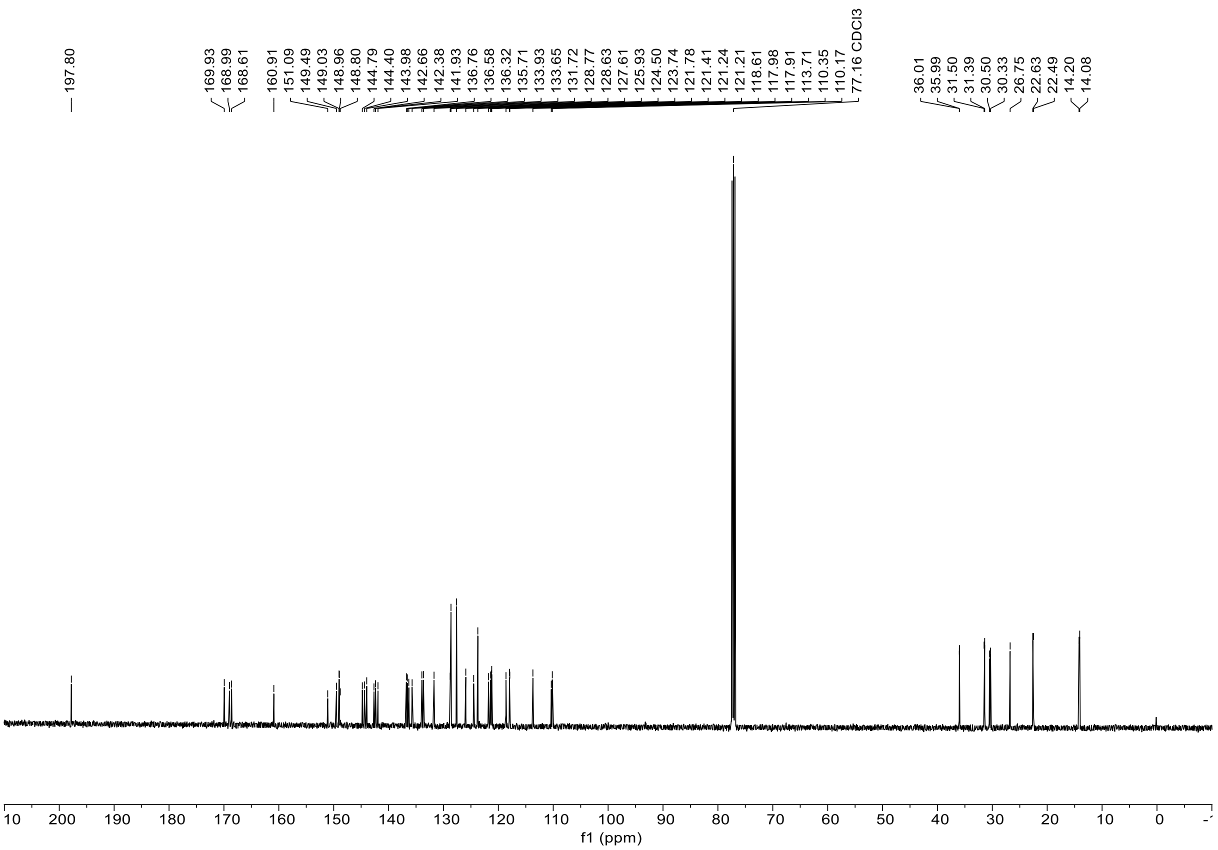

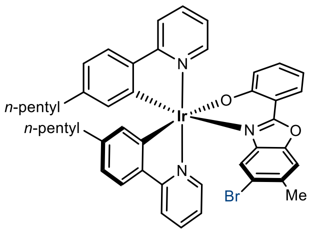

Analytical data of **1b:**

^{1}H NMR (500 MHz, Chloroform-*d*) δ 8.80 (d, *J* = 5.5 Hz, 1H), 8.03 (d, *J* = 6.0 Hz, 1H), 7.89 (dd, *J* = 8.5, 2.0 Hz, 1H), 7.70 (dd, *J* = 19.5, 8.0 Hz, 2H), 7.52 (dt, *J* = 18.0, 8.0 Hz, 3H), 7.41 (d, *J* = 8.0 Hz, 1H), 7.21 (s, 1H), 7.18 – 7.12 (m, 1H), 6.94 (t, *J* = 6.5 Hz, 1H), 6.81 – 6.71 (m, 3H), 6.64 (dd, *J* = 8.0, 1.5 Hz, 1H), 6.41 (t, *J* = 7.5 Hz, 1H), 6.22 (d, *J* = 1.5 Hz, 1H), 6.05 (s, 1H), 5.97 (d, *J* = 2.0 Hz, 1H), 2.35 – 2.25 (m, 7H), 1.50 – 1.35 (m, 4H), 1.22 – 1.09 (m, 8H), 0.78 (dt, *J* = 13.5, 7.0 Hz, 6H).

^{13}C NMR (126 MHz, $CDCl_3$) δ 169.9, 168.9, 168.6, 160.4, 151.2, 148.9, 148.73, 148.71, 148.1, 144.6, 143.9, 142.6, 142.0, 140.4, 136.7, 136.5, 134.1, 133.9, 133.5, 131.9, 128.7, 126.1, 124.4, 123.8, 122.5, 121.7, 121.6, 121.2, 121.1, 119.9, 118.6, 117.9, 113.5, 111.0, 110.1, 36.1, 36.0, 31.7, 31.6, 31.0, 30.3, 23.5, 22.63, 22.61, 14.19, 14.17.

HRMS (ESI) for $C_{46}H_{46}BrIrN_3O_2$ $[M+H]^+$ calcd. 944.2397, found 944.2405;

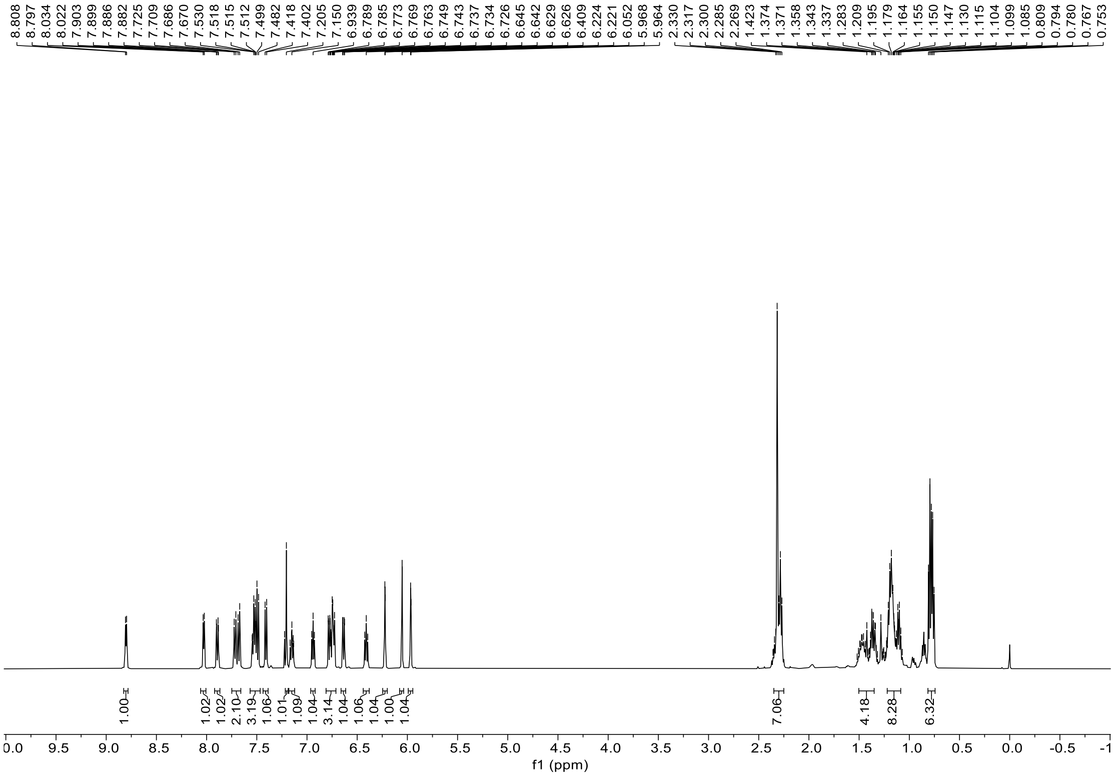

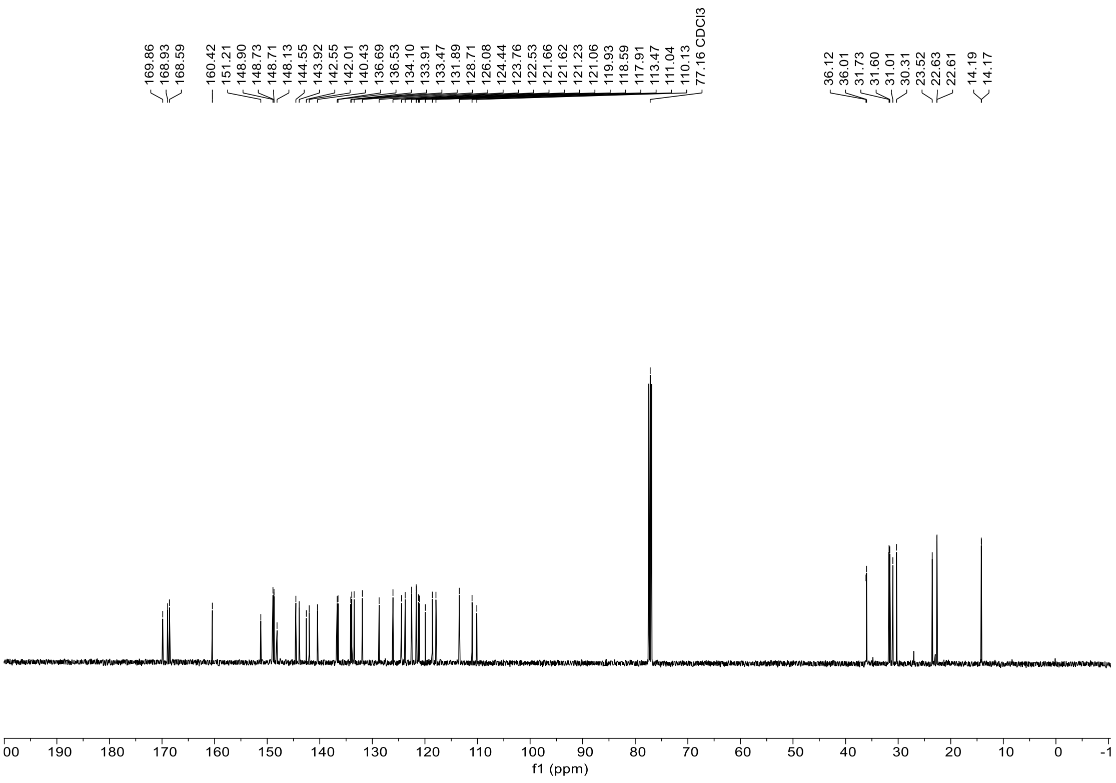

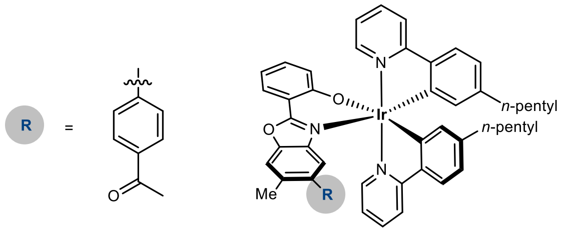

Analytical data of **3b:**

^{1}H NMR (500 MHz, Chloroform-*d*) δ 8.84 (d, *J* = 6.0 Hz, 1H), 8.12 (d, *J* = 5.5 Hz, 1H), 7.96 (d, *J* = 8.0 Hz, 1H), 7.90 (d, *J* = 8.0 Hz, 2H), 7.74 (dd, *J* = 25.5, 8.0 Hz, 2H), 7.63 – 7.55 (m, 2H), 7.44 (dd, *J* = 14.5, 8.0 Hz, 2H), 7.31 (s, 1H), 7.21 – 7.15 (m, 1H), 7.05 – 6.96 (m, 3H), 6.84 – 6.74 (m, 2H), 6.67 (dd, *J* = 8.0, 2.0 Hz, 1H), 6.52 – 6.42 (m, 2H), 6.13 (s, 1H), 6.03 (s, 1H), 5.93 (s, 1H), 2.64 (s, 3H), 2.32 – 2.21 (m, 5H), 2.08 – 1.97 (m, 2H), 1.42 – 1.32 (m, 2H), 1.23 – 1.15 (m, 2H), 1.13 – 1.00 (m, 4H), 0.95 – 0.85 (m, 4H), 0.79 (t, *J* = 7.5 Hz, 3H), 0.71 (t, *J* = 7.5 Hz, 3H).

^{13}C NMR (126 MHz, $CDCl_3$) δ 197.7, 169.5, 168.9, 168.5, 160.1, 151.2, 149.1, 149.0, 148.9, 148.4, 145.8, 144.0, 143.9, 142.5, 141.9, 139.7, 137.2, 136.6, 136.4, 135.4, 133.7, 133.3, 132.2, 131.8, 130.0, 128.6, 127.8, 125.8, 124.0, 123.7, 121.6, 121.4, 121.1, 121.0, 119.9, 118.5, 117.7, 113.5, 111.1, 110.4, 35.9, 35.7, 31.5, 31.4, 30.24, 30.16, 26.6, 22.5, 22.4, 21.0, 14.07, 14.06.

HRMS (ESI) for $C_{54}H_{53}IrN_3O_3$ $[M+H]^+$ calcd. 984.3711, found 984.3695;

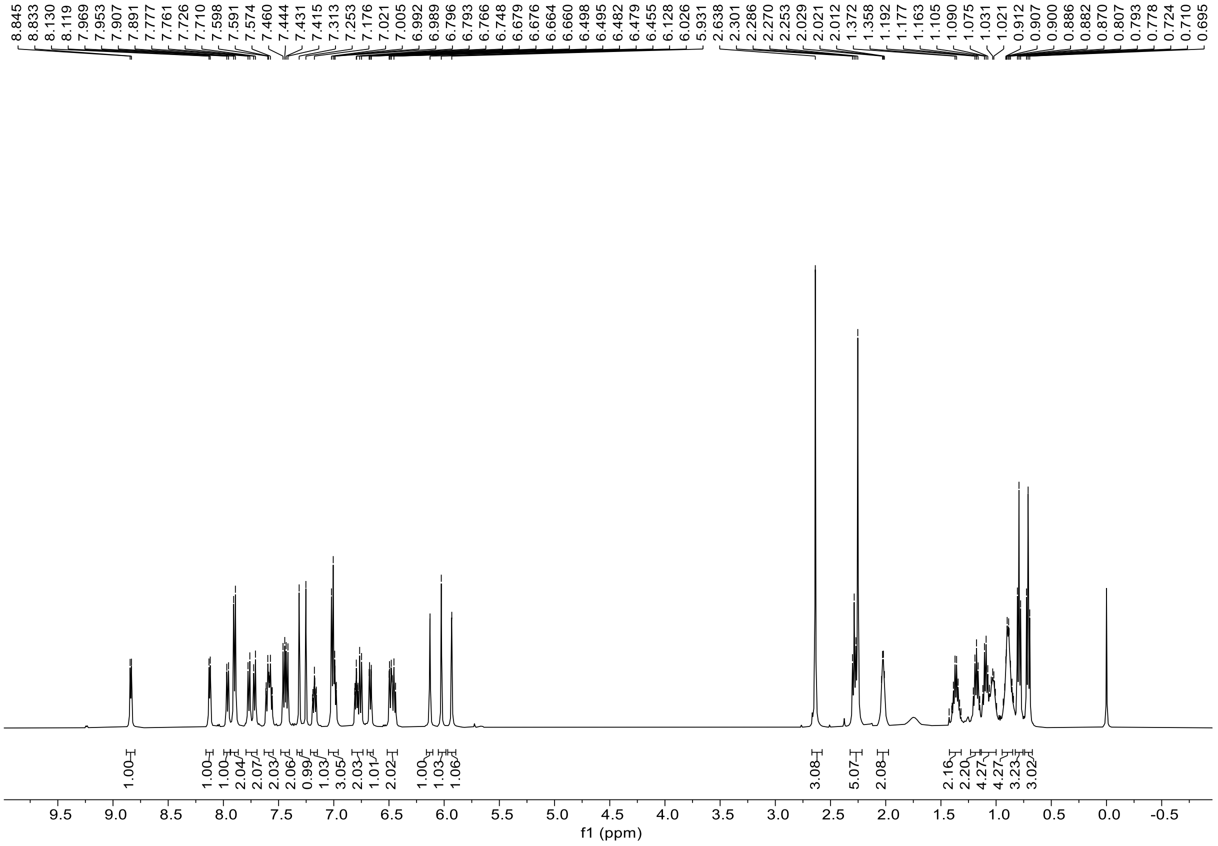

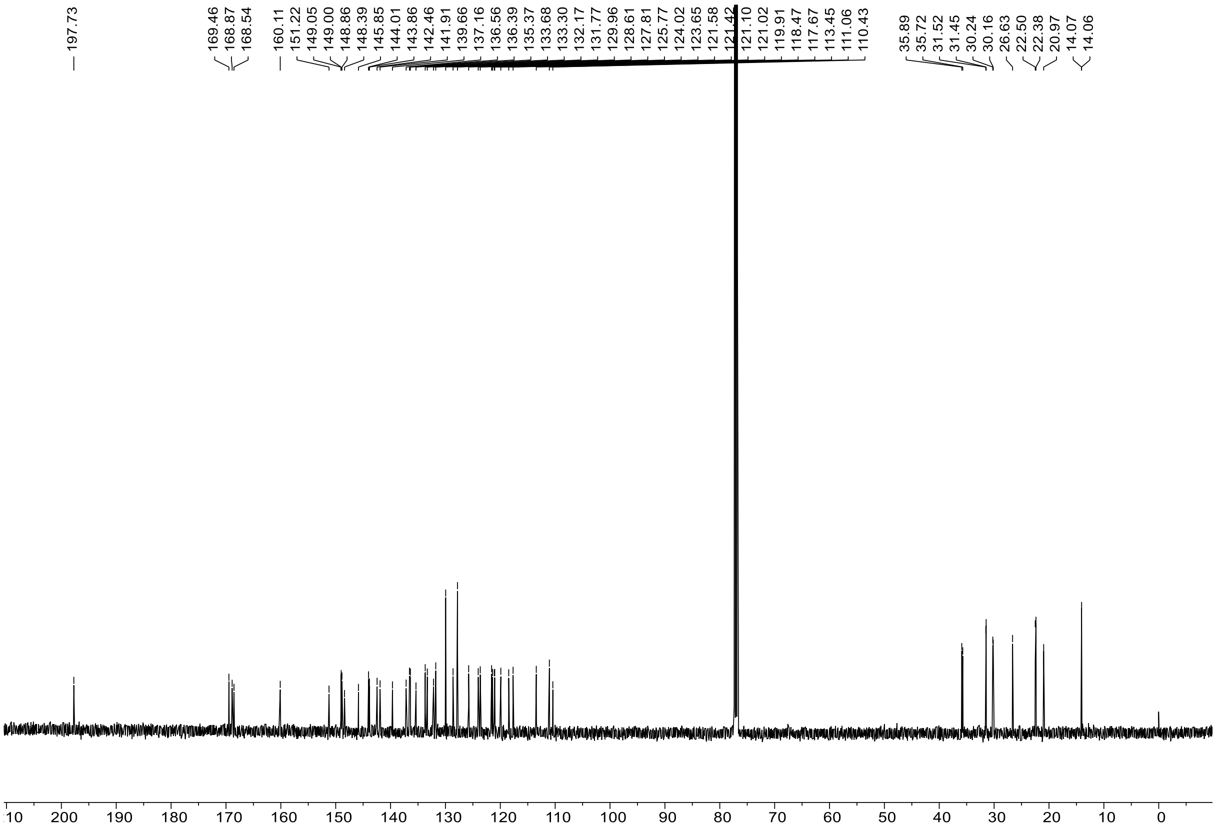

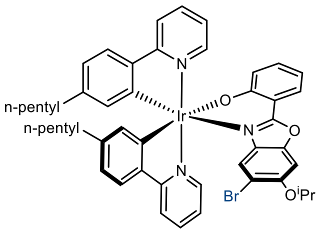

Analytical data of **1d:**

^{1}H NMR (500 MHz, Chloroform-*d*) δ 8.81 (d, *J* = 5.5 Hz, 1H), 8.04 (d, *J* = 6.0 Hz, 1H), 7.87 (d, *J* = 8.0 Hz, 1H), 7.71 (dd, *J* = 22.5, 8.0 Hz, 2H), 7.59 – 7.51 (m, 2H), 7.49 (d, *J* = 8.0 Hz, 1H), 7.45 – 7.40 (m, 1H), 7.14 (dd, *J* = 78.0, 8.0 Hz, 1H), 6.98 – 6.92 (m, 2H), 6.82 – 6.71 (m, 3H), 6.65 (d, *J* = 8.0 Hz, 1H), 6.41 (t, *J* = 7.5 Hz, 1H), 6.22 (s, 1H), 6.02 (s, 1H), 5.97 (s, 1H), 4.41 (p, *J* = 6.0 Hz, 1H), 2.39 – 2.23 (m, *J* = 7.7, 7.0 Hz, 4H), 1.51 – 1.30 (m, 10H), 1.24 – 1.08 (m, 8H), 0.83 – 0.72 (m, 6H).

^{13}C NMR (126 MHz, $CDCl_3$) δ 169.4, 168.9, 168.6, 160.0, 152.0, 151.3, 149.3, 148.9, 148.7, 148.0, 144.6, 143.9, 142.6, 142.0, 136.7, 136.5, 135.4, 133.9, 133.2, 131.9, 128.5, 125.9, 124.5, 123.8, 123.2, 121.67, 121.65, 121.2, 121.1, 118.6, 117.9, 113.4, 110.4, 109.8, 97.2, 72.9, 36.1, 36.0, 31.7, 31.6, 31.0, 30.3, 22.6, 22.0, 14.2.

HRMS (ESI) for $C_{48}H_{50}BrIrN_3O_3$ $[M+H]^+$ calcd. 988.2660, found 988.2661;

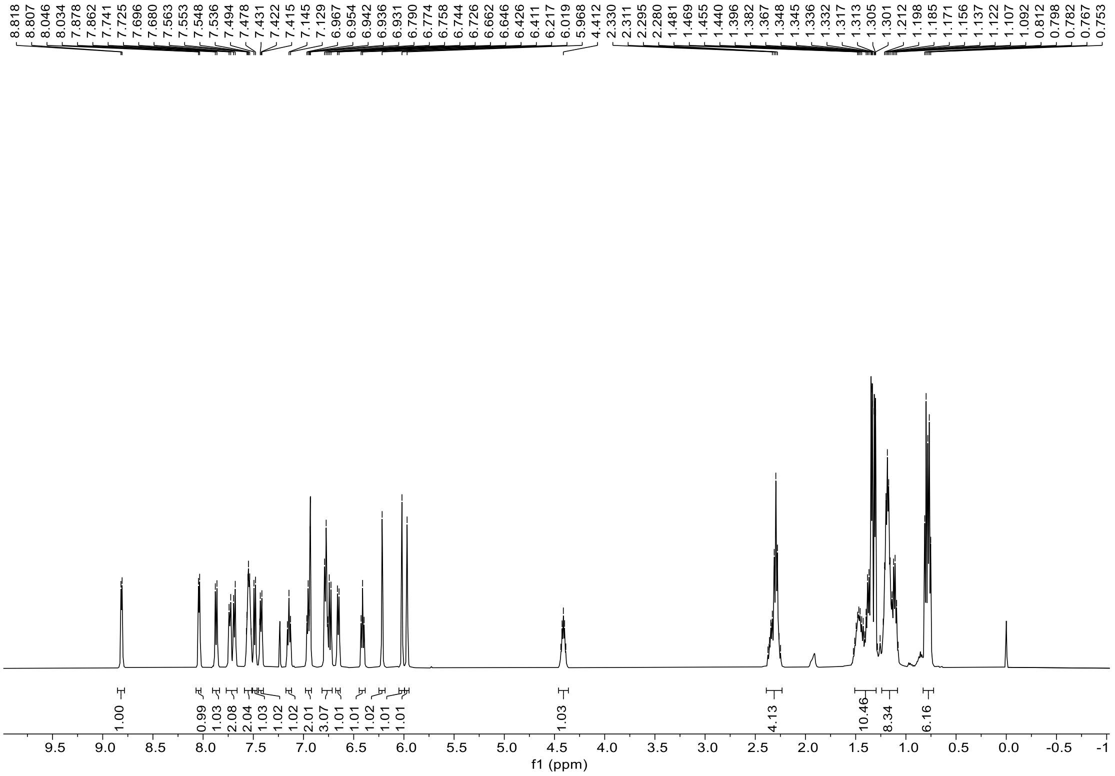

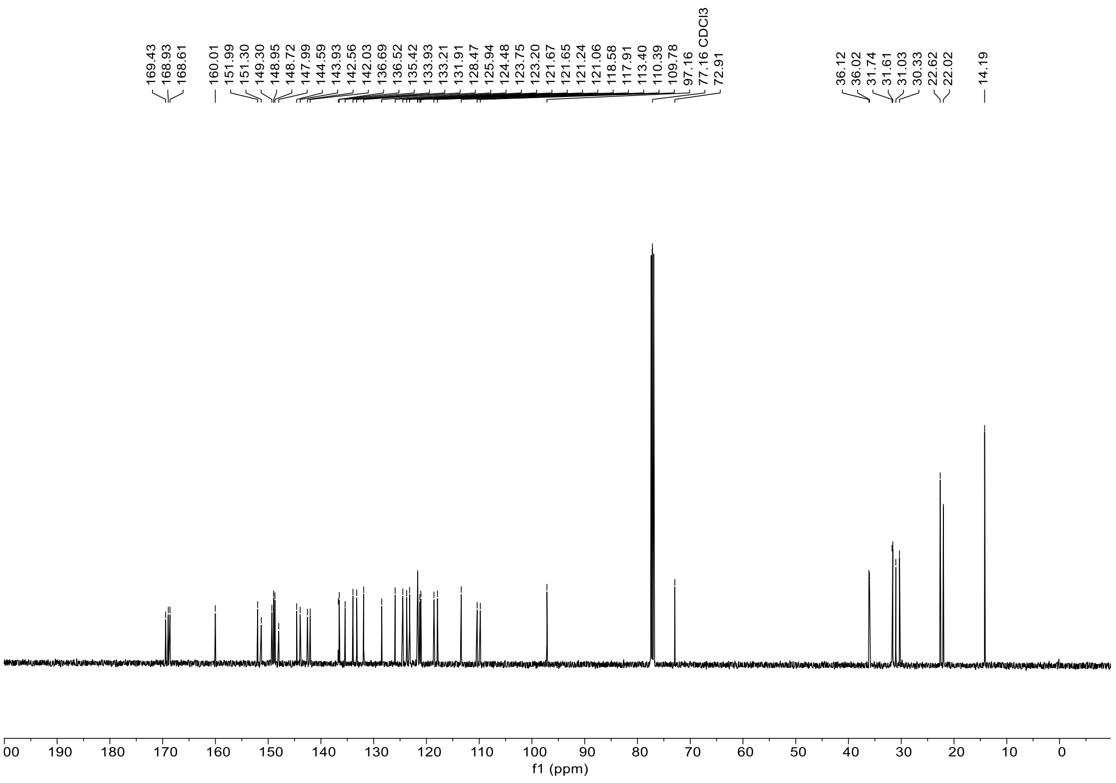

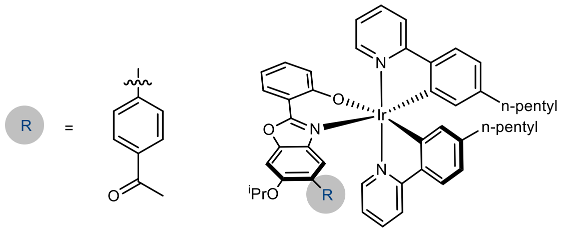

Analytical data of **3d:**

^{1}H NMR (500 MHz, Chloroform-*d*) δ 8.87 (d, *J* = 6.0 Hz, 1H), 8.11 (d, *J* = 6.0 Hz, 1H), 7.93 (d, *J* = 8.0 Hz, 1H), 7.86 (d, *J* = 8.0 Hz, 2H), 7.80 – 7.71 (m, 2H), 7.58 (dt, *J* = 21.5, 8.0 Hz, 2H), 7.49 (d, *J* = 8.0 Hz, 1H), 7.44 (d, *J* = 8.0 Hz, 1H), 7.17 (dd, *J* = 17.5, 8.0 Hz, 3H), 7.04 (s, 1H), 7.00 (t, *J* = 7.0 Hz, 1H), 6.83 – 6.72 (m, 2H), 6.65 (dd, *J* = 15.0, 8.0 Hz, 2H), 6.45 (t, *J* = 7.5 Hz, 1H), 6.22 (s, 1H), 6.16 (s, 1H), 5.92 (s, 1H), 4.45 – 4.34 (m, 1H), 2.62 (s, 3H), 2.29 (t, *J* = 7.5 Hz, 2H), 2.18 – 2.05 (m, 2H), 1.40 – 1.32 (m, 2H), 1.29 (d, *J* = 6.0 Hz, 3H), 1.22 – 1.14 (m, 5H), 1.09 (q, *J* = 7.5 Hz, 2H), 1.04 – 0.94 (m, 4H), 0.92 – 0.84 (m, 2H), 0.80 (t, *J* = 7.5 Hz, 3H), 0.68 (t, *J* = 7.5 Hz, 3H).

^{13}C NMR (126 MHz, $CDCl_3$) δ 197.9, 169.1, 168.9, 168.6, 159.7, 152.8, 151.3, 149.9, 149.0, 148.8, 148.4, 144.1, 143.8, 143.0, 142.5, 141.9, 136.6, 136.4, 135.2, 135.1, 133.8, 133.1, 131.7, 130.2, 128.3, 127.51, 127.49, 125.7, 124.2, 123.6, 121.6, 121.5, 121.1, 121.0, 120.7, 118.5,

117.7, 113.4, 110.6, 96.4, 71.7, 35.9, 35.8, 31.43, 31.41, 30.23, 30.21, 26.6, 22.5, 22.4, 21.9, 21.8, 14.1, 14.0.

HRMS (ESI) for $C_{56}H_{57}IrN_3O_4$ $[M+H]^+$ calcd. 1028.3973, found 1028.3965;

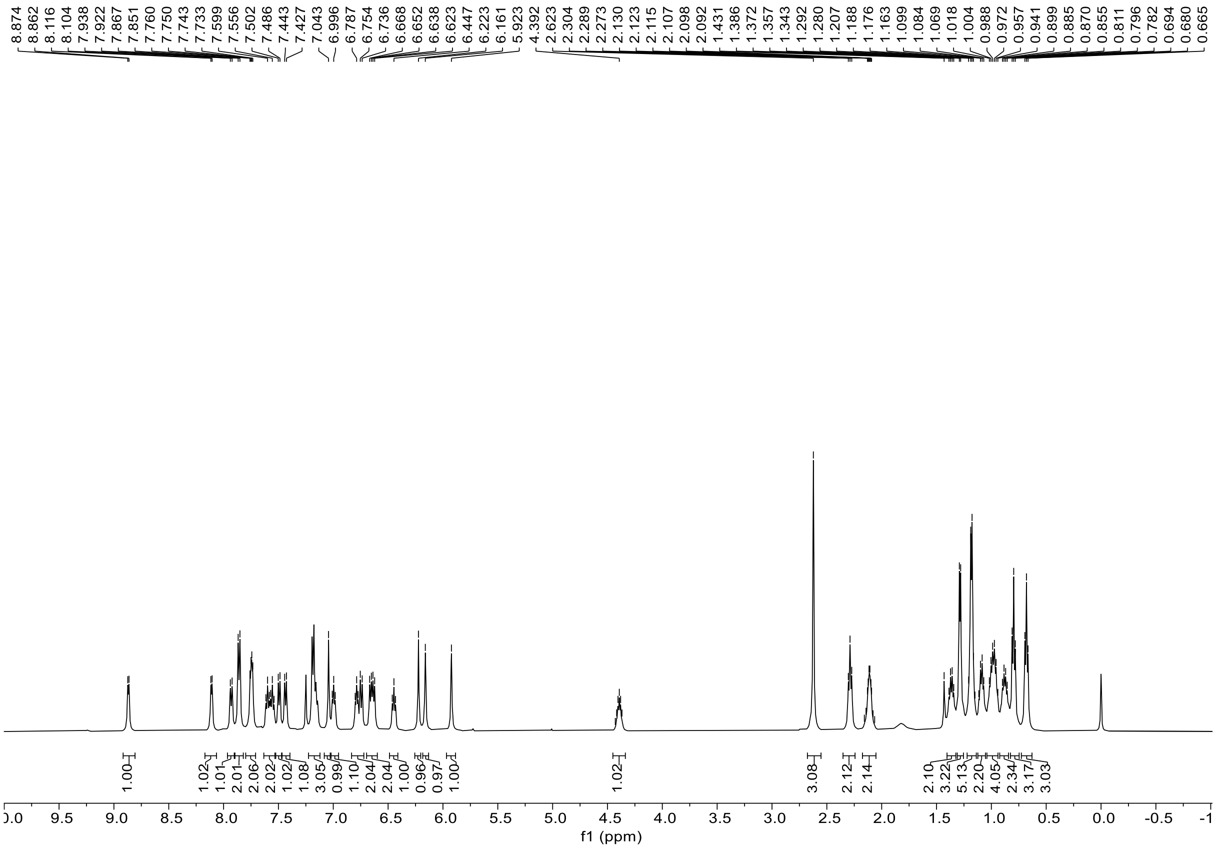

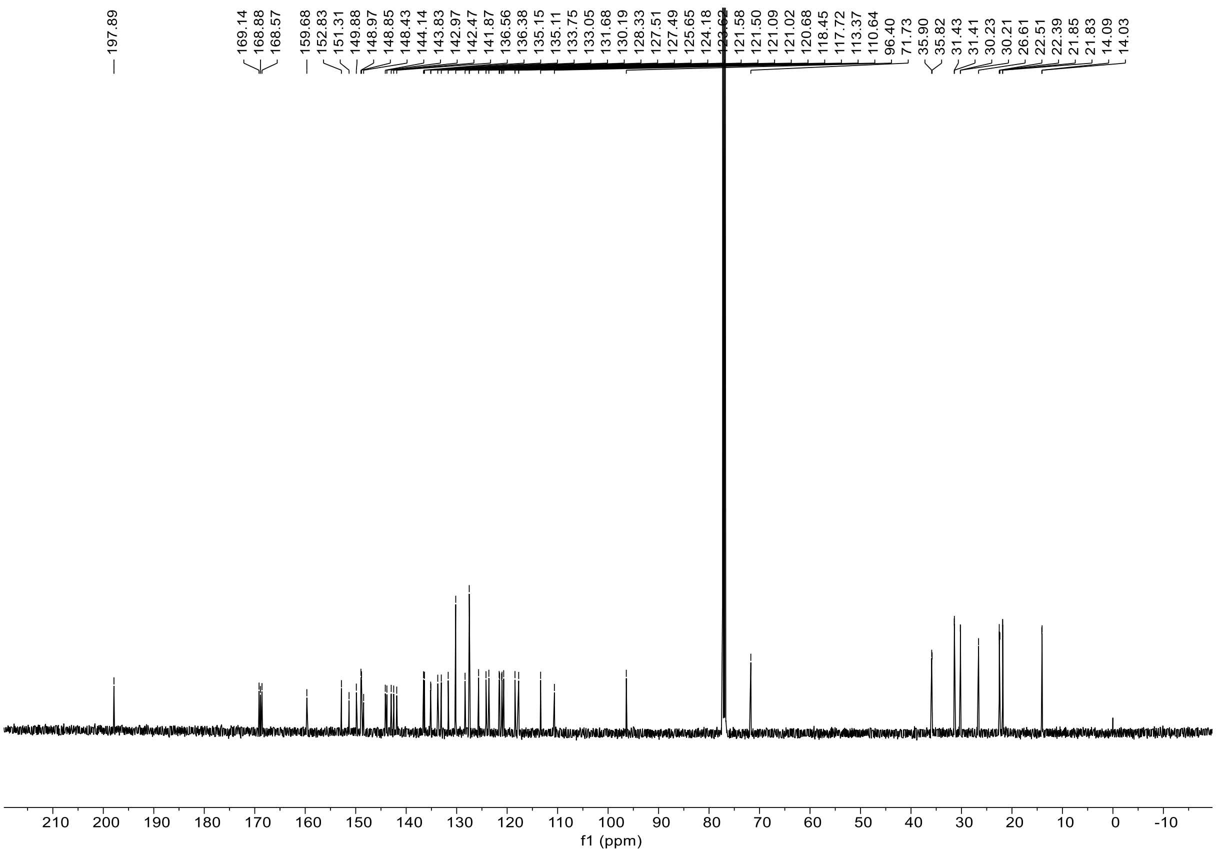
197.89
169.14
168.88
168.57
159.68
152.83
151.31
149.88
148.97
148.85
148.43
144.14
143.83
142.97
142.47
141.87
136.56
136.38
135.15
135.11
133.75
133.05
131.68
130.19
128.33
127.51
127.49
125.65
124.18
121.58
121.50
121.09
121.02
120.68
118.45
117.72
113.37
110.64
96.40
71.73
35.90
35.82
31.43
31.41
30.23
30.21
26.61
22.51
22.39
21.85
21.83
14.09
14.03
210 200 190 180 170 160 150 140 130 120 110 100 90 80 70 60 50 40 30 20 10 0 -10
f1 (ppm)

4.General Procedure for Kinetic Resolution and Characterization Data

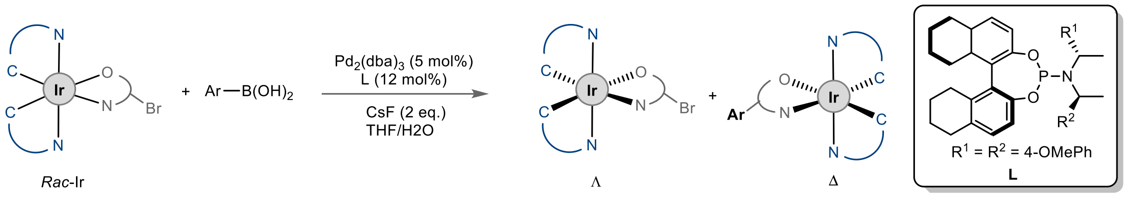

A dried 10 mL Schlenk tube was charged with the *rac*-**Ir** (0.05 mmol, 1.0 equiv.), arylboronic acid (0.1 mmol, 2 equiv.), $Pd_2(dba)_3$ (5 mol%, 2.3 mg), L (12 mol%, 3.7 mg), CsF (0.1 mmol, 15.2 mg, 2 equiv.) and THF/H_2O (v/v, 9:1, 0.5 mL) in glovebox. The mixture was stirred at 30°C and monitored by HPLC analysis. After achieving appropriate conversion, the mixture was concentrated under vacuum to give a residue, which was purified by column chromatography on silica gel to give the recovered Λ-**Ir** and product Δ-**Ir**.

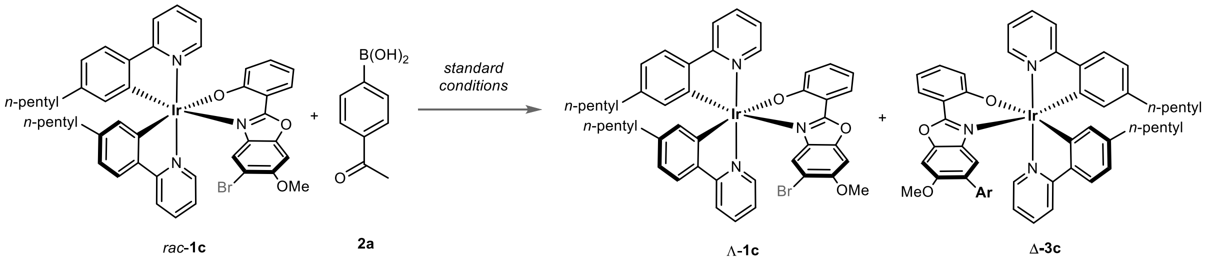

According to the general procedure, the reaction was carried out at 30 °C for 3.5 days to give the recovered Λ-**1c** as yellow solid (21.6 mg, 45% yield) and Δ-**3c** as a yellow solid (23.5 mg, 47% yield).

Purification conditions: petroleum ether/EtOAc = 10:1 to 4:1.

R_f (Λ-**1c**) = 0.6 in petroleum ether/EtOAc (4:1).

R_f (Δ-**3c**) = 0.4 in petroleum ether/EtOAc (4:1).

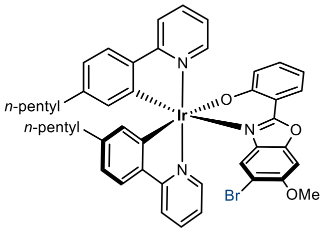

Analytical data of Λ-**1c:**

^{1}H NMR (500 MHz, Chloroform-*d*) δ 8.82 (s, 1H), 8.03 (d, *J* = 5.5 Hz, 1H), 7.87 (dd, *J* = 8.0, 2.0 Hz, 1H), 7.77 – 7.67 (m, 2H), 7.60 – 7.46 (m, 3H), 7.46 – 7.38 (m, 1H), 7.20 – 7.14 (m,

1H), 6.97 (t, J = 6.5 Hz, 1H), 6.94 – 6.86 (m, 1H), 6.82 – 6.72 (m, 3H), 6.69 – 6.63 (m, 1H), 6.43 (t, J = 7.5 Hz, 1H), 6.22 (s, 1H), 6.03 – 5.97 (m, 2H), 3.81 – 3.72 (m, 3H), 2.39 – 2.24 (m, 4H), 1.56 – 1.33 (m, 4H), 1.25 – 1.09 (m, 8H), 0.84 – 0.74 (m, 6H).

^{13}C NMR (126 MHz, $CDCl_3$) δ 169.2, 168.8, 168.5, 159.9, 153.6, 151.2, 149.4, 148.9, 148.7, 147.8, 144.7, 144.0, 142.6, 142.1, 136.8, 136.6, 135.3, 135.2, 133.9, 133.3, 131.9, 128.5, 125.8, 124.5, 123.8, 123.1, 121.8, 121.7, 121.3, 121.2, 118.6, 118.0, 113.6, 110.3, 107.8, 94.2, 56.8, 36.1, 36.0, 31.7, 31.64, 31.62, 31.1, 30.4, 22.6, 14.21, 14.2.

HRMS (ESI) for $C_{46}H_{46}BrIrN_3O_3$ $[M+H]^+$ calcd. 960.2347, found 960.2340;

Enantiomeric excess established by HPLC analysis using a Chiralpak IK-3 column, ee = 90% (HPLC: IK-3, 254 nm, *n*-hexane/isopropanol = 90:10, flow rate 1.0 mL/min, 40 °C, t_r (major) = 10.0 min, t_r (minor) = 12.0 min.)

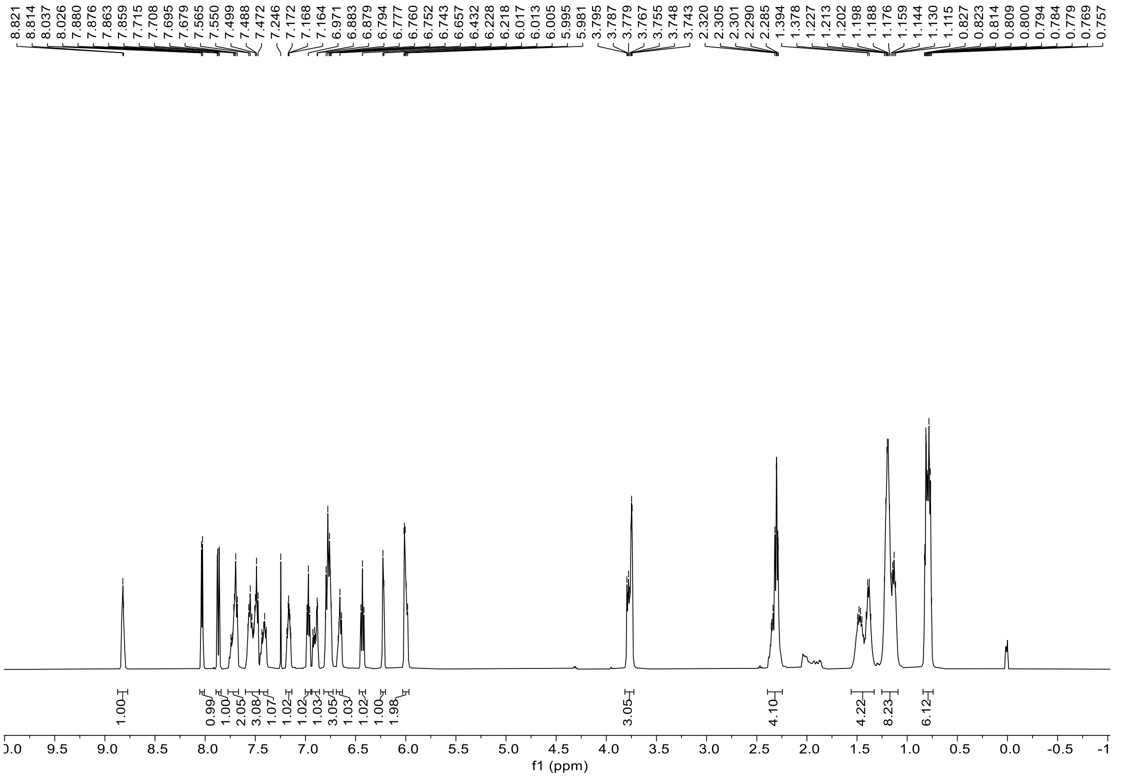

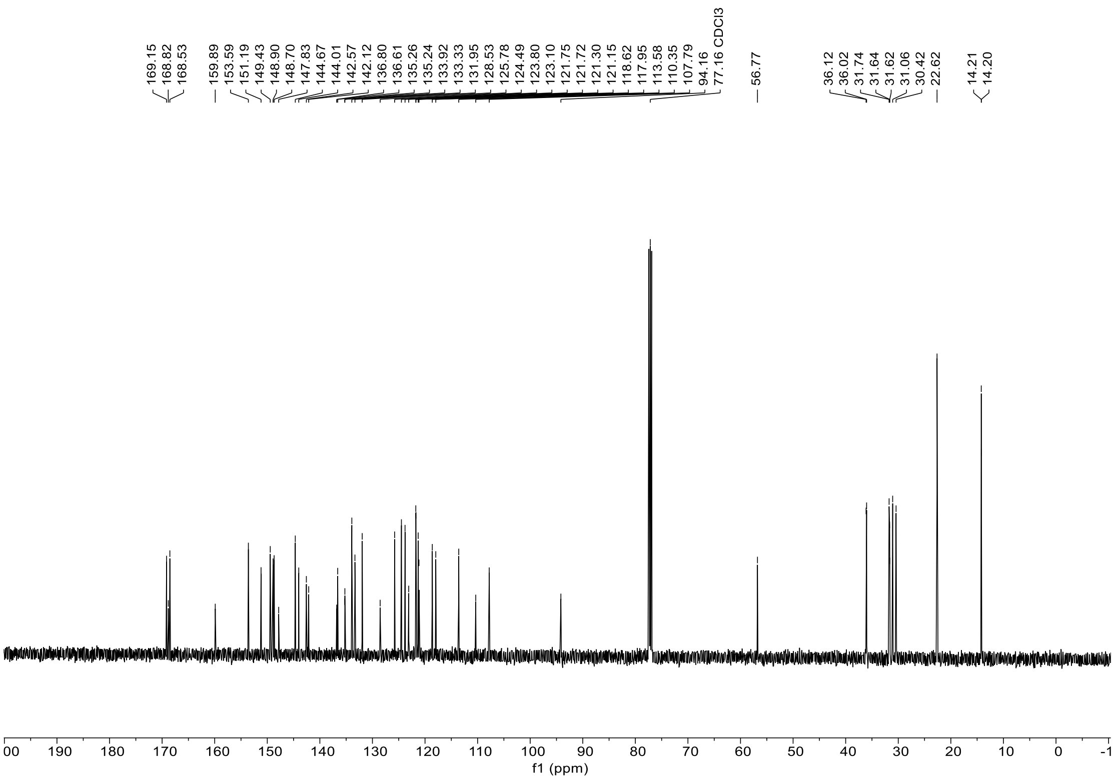

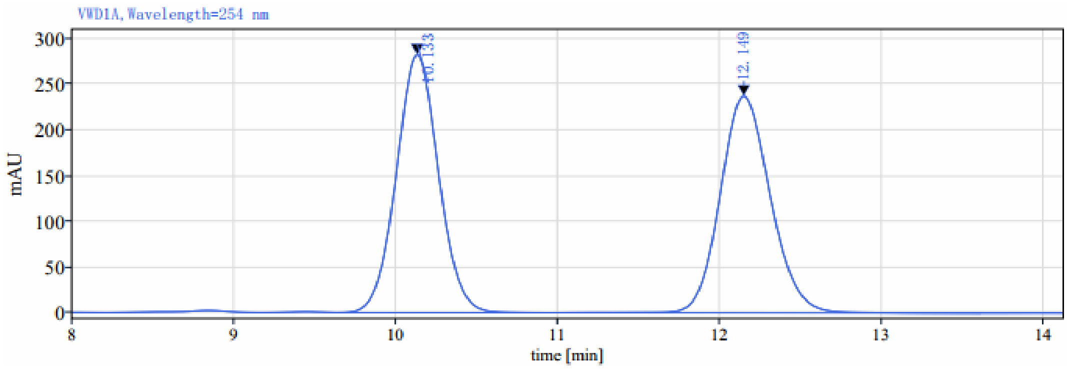

Rettime [min]	Type	Width [min]	Area	Height	Area%
10.133	VB	1.30	5128.08	282.05	50.11
12.149	BB	2.66	5106.14	237.26	49.89

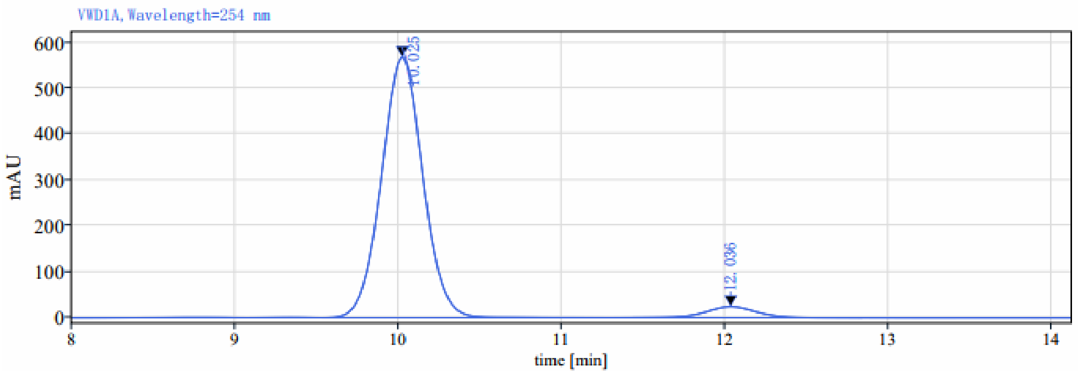

Rettime [min]	Type	Width [min]	Area	Height	Area%
10.025	VB	1.73	10025.23	567.40	95.21
12.036	MM m	1.41	503.98	23.63	4.79

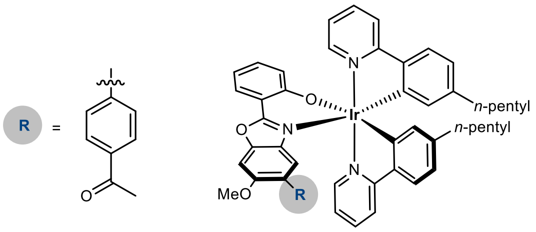

Analytical data of Δ-**3c:**

^{1}H NMR (500 MHz, Chloroform-*d*) δ 8.85 (d, *J* = 6.0 Hz, 1H), 8.11 (d, *J* = 6.0 Hz, 1H), 7.94 (dd, *J* = 8.0, 2.0 Hz, 1H), 7.88 (d, *J* = 8.0 Hz, 2H), 7.74 (t, *J* = 7.5 Hz, 2H), 7.58 (dt, *J* = 19.1, 7.5 Hz, 2H), 7.48 (d, *J* = 8.0 Hz, 1H), 7.42 (d, *J* = 8.0 Hz, 1H), 7.17 (d, *J* = 8.0 Hz, 3H), 7.04 (s, 1H), 7.00 (t, *J* = 6.5 Hz, 1H), 6.82 – 6.74 (m, 2H), 6.63 (dd, *J* = 17.5, 8.0 Hz, 2H), 6.47 (t, *J* = 7.5 Hz, 1H), 6.21 (s, 1H), 6.16 (s, 1H), 5.94 (s, 1H), 3.76 (s, 3H), 2.62 (s, 3H), 2.28 (t, *J* = 7.5 Hz, 2H), 2.15 – 2.06 (m, 2H), 1.41 – 1.31 (m, 2H), 1.21 – 1.15 (m, 2H), 1.12 – 1.08 (m, 2H), 1.03 – 0.92 (m, 4H), 0.91 – 0.85 (m, 2H), 0.80 (t, *J* = 7.0 Hz, 3H), 0.68 (t, *J* = 7.0 Hz, 3H).

^{13}C NMR (126 MHz, $CDCl_3$) δ 197.9, 168.9, 168.8, 168.6, 159.7, 154.8, 151.2, 150.1, 149.0, 148.9, 148.2, 144.3, 144.0, 142.63, 142.56, 142.1, 136.8, 136.6, 135.4, 135.1, 133.8, 133.3, 131.8, 130.2, 128.5, 127.8, 126.6, 125.5, 124.3, 123.8, 121.8, 121.7, 121.3, 121.2, 120.6, 118.6, 117.9, 113.8, 110.7, 93.7, 56.2, 36.0, 35.9, 31.6, 31.5, 30.4, 30.3, 26.7, 22.6, 22.5, 14.2, 14.1.

HRMS (ESI) for $C_{54}H_{53}IrN_3O_4[M+H]^+$ calcd. 1000.3660, found 1000.3659;

Enantiomeric excess established by HPLC analysis using a Chiralpak IM column, ee = 90% (HPLC: IM, 254 nm, *n*-hexane/isopropanol = 80:20, flow rate 1.0 mL/min, 40 °C, t_r (major) =

13.3 min, t_r (minor) = 17.3 min.)

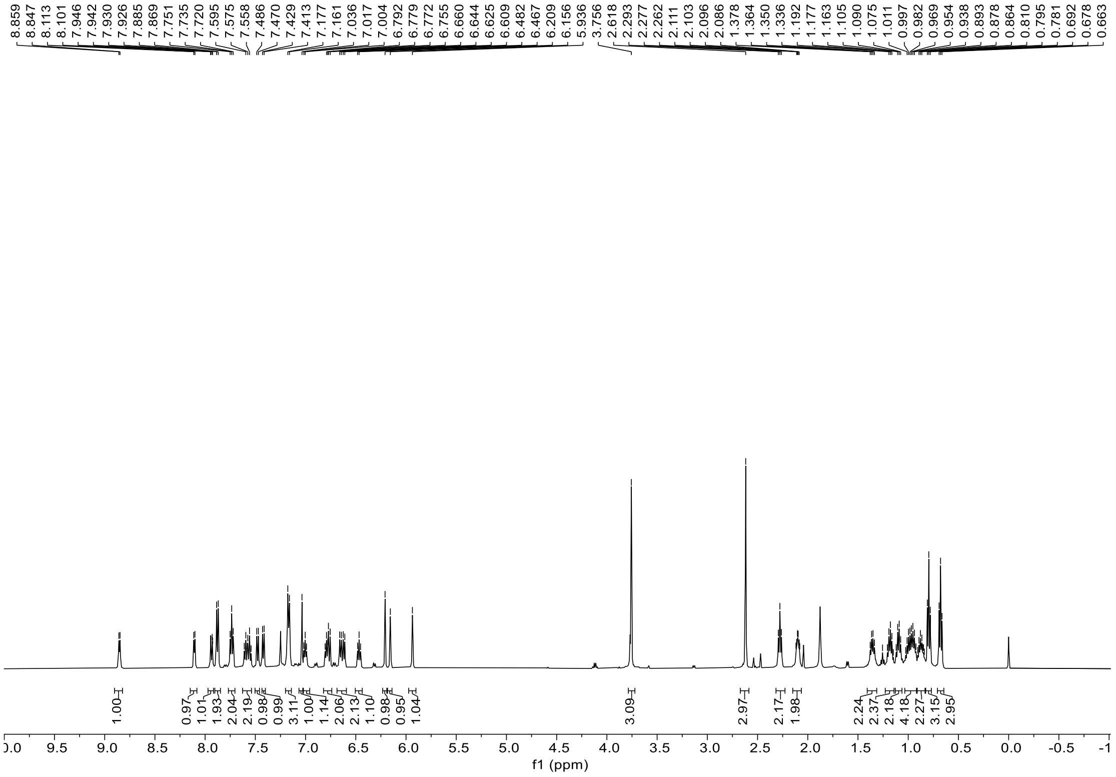

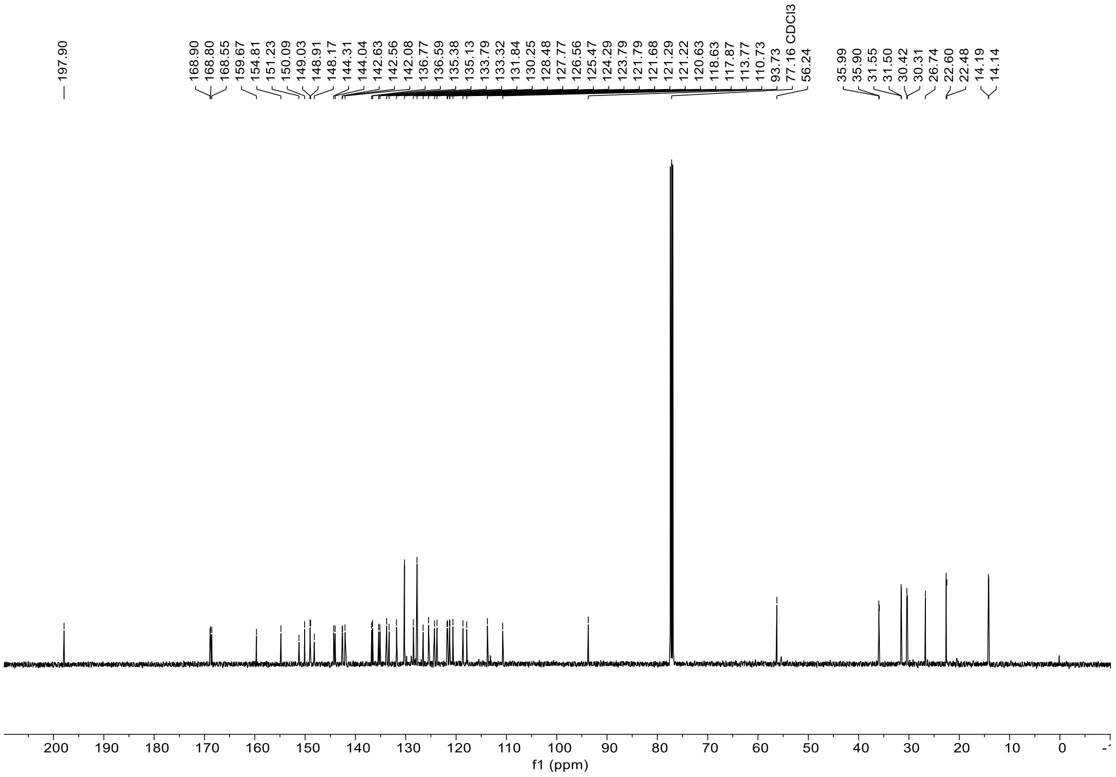

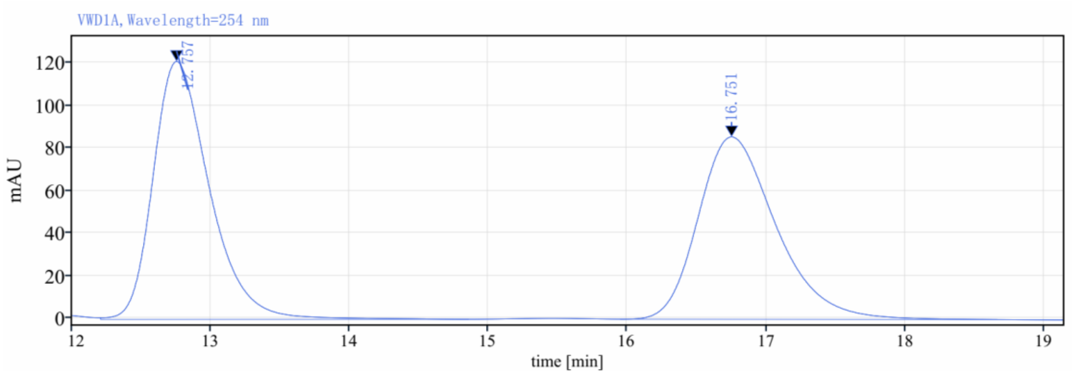

Rettime [min]	Type	Width [min]	Area	Height	Area%
12.757	VB	2.65	3426.06	121.42	50.63
16.751	BM m	2.71	3341.10	85.88	49.37

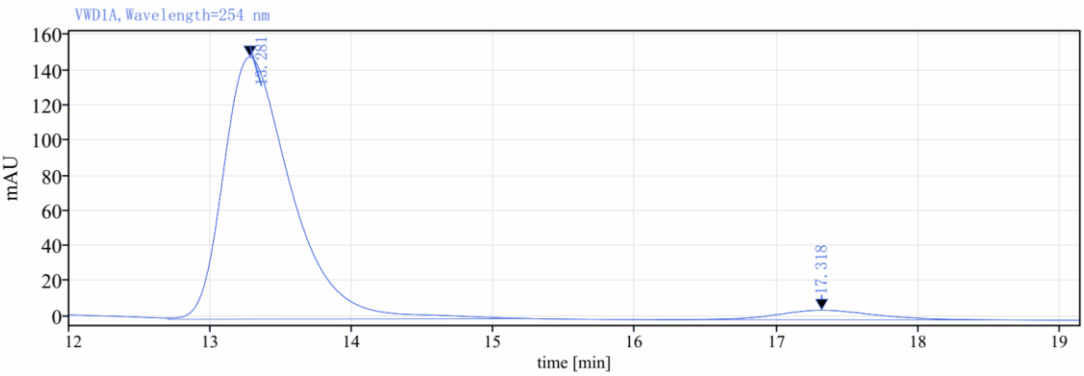

Rettime [min]	Type	Width [min]	Area	Height	Area%
13.281	VM m	2.56	4965.24	149.38	94.89
17.318	BM m	2.52	267.33	5.45	5.11

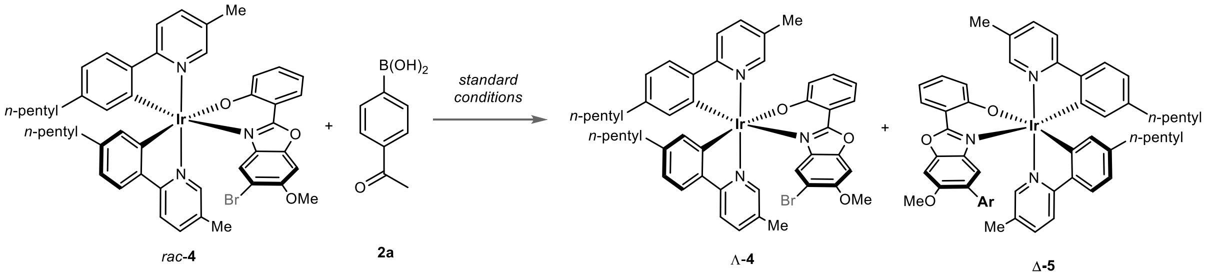

According to the general procedure, the reaction was carried out at 30 ºC for 2.5 days to give the recovered Λ-**4** as yellow solid (18.9 mg, 38% yield) and Δ-**5** as a yellow solid (22.6 mg, 44% yield).

Purification conditions: petroleum ether/EtOAc = 10:1 to 4:1.

R_f (Λ-**4**) = 0.5 in petroleum ether/EtOAc (4:1).

R_f (Δ-**5**) = 0.4 in petroleum ether/EtOAc (4:1).

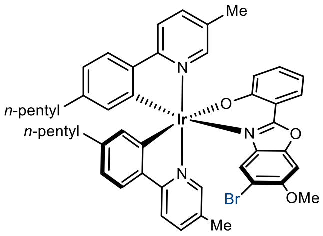

Analytical data of Λ-**4:**

^{1}H NMR (500 MHz, Chloroform-*d*) δ 8.68 (d, *J* = 2.0 Hz, 1H), 7.87 – 7.80 (m, 2H), 7.60 (d, *J* = 8.5 Hz, 2H), 7.43 (d, *J* = 8.0 Hz, 1H), 7.40 – 7.32 (m, 3H), 7.16 – 7.11 (m, 1H), 6.88 (s, 1H), 6.77 – 6.68 (m, 2H), 6.63 (d, *J* = 8.0 Hz, 1H), 6.40 (t, *J* = 7.5 Hz, 1H), 6.21 (s, 1H), 6.02 (s, 1H), 5.96 (s, 1H), 3.76 (s, 3H), 2.37 – 2.25 (m, 4H), 2.21 (s, 3H), 2.08 (s, 3H), 1.50 – 1.35 (m, 4H), 1.24 – 1.10 (m, 8H), 0.80 (t, *J* = 7.0 Hz, 3H), 0.74 (t, *J* = 7.0 Hz, 3H).

^{13}C NMR (126 MHz, $CDCl_3$) δ 169.7, 166.3, 165.9, 160.1, 153.5, 150.3, 149.5, 148.7, 148.4, 146.9, 143.9, 143.3, 142.8, 142.3, 137.7, 137.5, 135.2, 134.0, 133.2, 131.9, 130.9, 130.7, 128.3, 125.9, 124.0, 123.2, 123.1, 121.53, 121.50, 118.0, 117.5, 113.4, 110.9, 107.8, 94.1, 56.7, 36.01, 35.99, 31.64, 31.62, 30.9, 30.2, 22.63, 22.62, 18.5, 18.4, 14.17, 14.15.

HRMS (ESI) for $C_{48}H_{50}BrIrN_3O_3$ $[M+H]^+$ calcd. 988.2660, found 988.2654;

Enantiomeric excess established by HPLC analysis using a Chiralpak IM column, ee = 96% (HPLC: IM, 254 nm, *n*-hexane/isopropanol = 90:10, flow rate 1.0 mL/min, 40 °C, t_r (major) = 11.5 min, t_r (minor) = 10.5 min.)

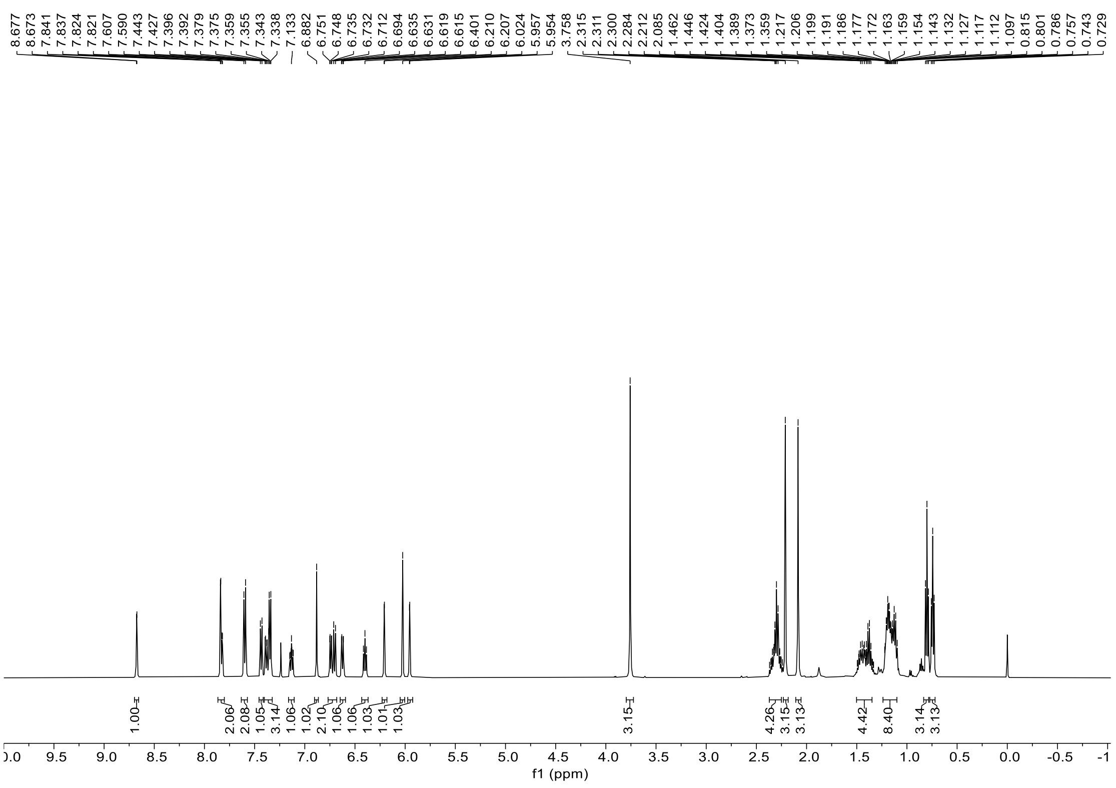

8.677
8.673
7.841
7.837
7.824
7.821
7.607
7.590
7.443
7.427
7.396
7.392
7.379
7.375
7.359
7.355
7.343
7.338
7.133
6.882
6.751
6.748
6.735
6.732
6.712
6.694
6.635
6.631
6.619
6.615
6.401
6.210
6.207
6.024
5.957
5.954
3.758
2.315
2.311
2.300
2.284
2.212
2.085
1.462
1.446
1.424
1.404
1.389
1.373
1.359
1.217
1.206
1.199
1.191
1.186
1.177
1.172
1.163
1.159
1.154
1.143
1.132
1.127
1.117
1.112
1.097
0.815
0.801
0.786
0.757
0.743
0.729
1.00
2.06
2.08
1.05
3.14
1.06
1.02
2.10
1.06
1.06
1.03
1.01
1.03
3.15
4.26
3.15
3.13
4.42
8.40
3.14
3.13
f1 (ppm)

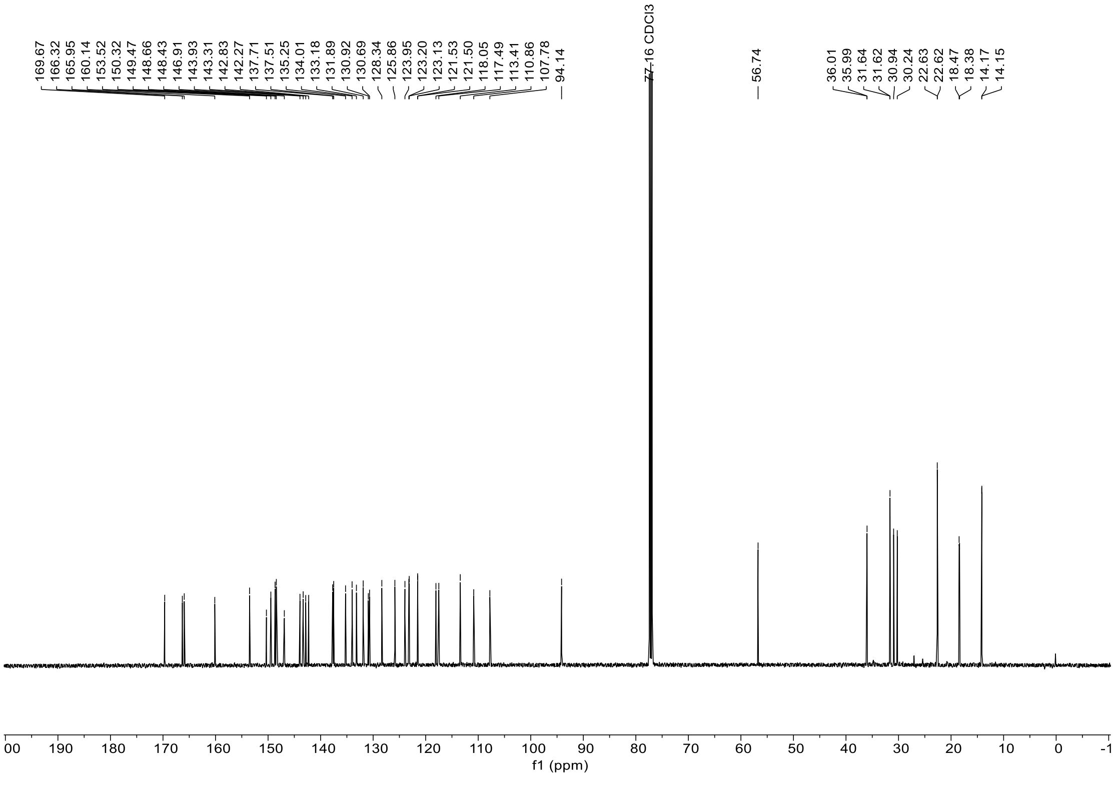

169.67
166.32
165.95
160.14
153.52
150.32
149.47
148.66
148.43
146.91
143.93
143.31
142.83
142.27
137.71
137.51
135.25
134.01
133.18
131.89
130.92
130.69
128.34
125.86
123.95
123.20
123.13
121.53
121.50
118.05
117.49
113.41
110.86
107.78
94.14
77.16 CDCl3
56.74
36.01
35.99
31.64
31.62
30.94
30.24
22.63
22.62
18.47
18.38
14.17
14.15
f1 (ppm)

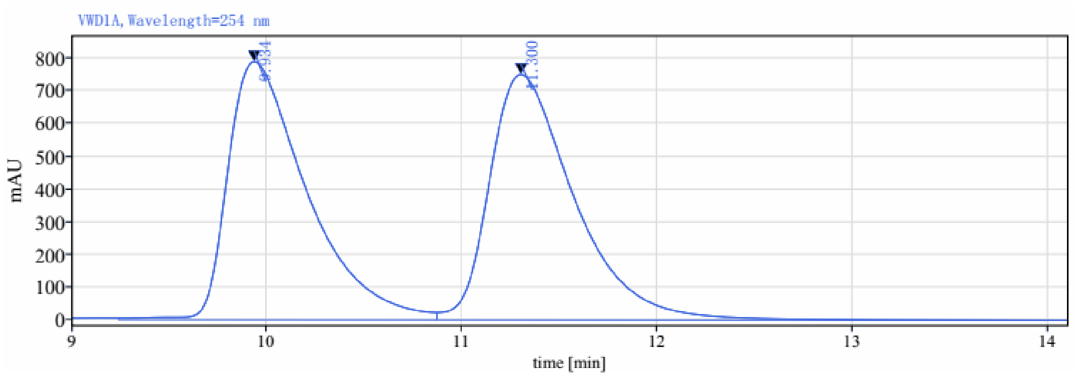

Rettime [min]	Type	Width [min]	Area	Height	Area%
9.934	VV	1.63	22221.84	787.45	49.53
11.300	VBA	3.70	22643.97	749.09	50.47

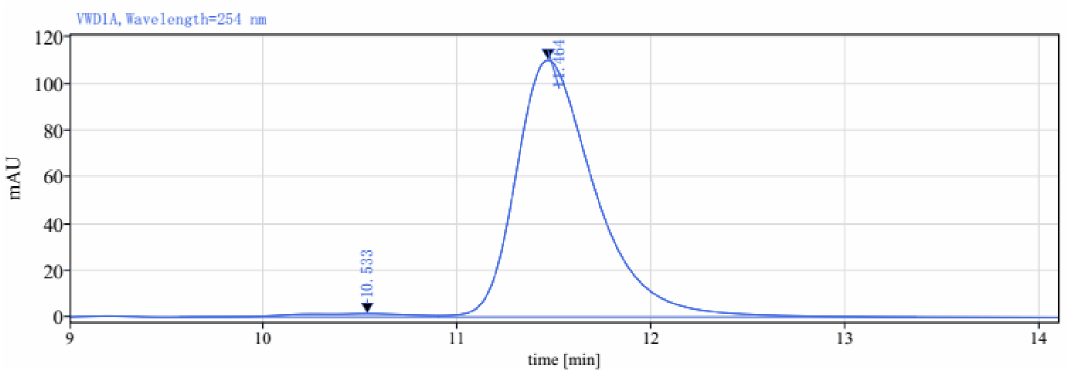

Rettime [min]	Type	Width [min]	Area	Height	Area%
10.533	MM m	1.34	60.56	1.40	1.91
11.464	MM m	2.47	3107.78	109.94	98.09

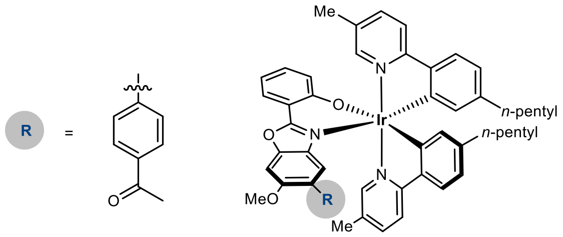

Analytical data of Δ-**5:**

^{1}H NMR (500 MHz, Chloroform-*d*) δ 8.73 (s, 1H), 7.95 – 7.85 (m, 4H), 7.62 (d, *J* = 7.5 Hz, 2H), 7.45 – 7.39 (m, 2H), 7.39 – 7.34 (m, 2H), 7.19 – 7.12 (m, 3H), 7.07 – 7.03 (m, 1H), 6.70 (d, *J* = 8.5 Hz, 1H), 6.61 (dd, *J* = 15.5, 8.0 Hz, 2H), 6.45 (t, *J* = 7.5 Hz, 1H), 6.24 (s, 1H), 6.17

(s, 1H), 5.90 (d, J = 2.5 Hz, 1H), 3.79 – 3.75 (m, 3H), 2.62 (s, 3H), 2.28 (t, J = 7.5 Hz, 2H), 2.24 (s, 3H), 2.17 – 2.11 (m, 2H), 2.10 (s, 3H), 1.39 – 1.32 (m, 2H), 1.22 – 1.15 (m, 2H), 1.12 – 1.00 (m, 6H), 0.91 – 0.85 (m, 2H), 0.80 (t, J = 7.0 Hz, 3H), 0.68 (t, J = 7.5 Hz, 3H).

^{13}C NMR (126 MHz, $CDCl_3$) δ 197.9, 169.52, 169.49, 166.4, 166.0, 160.0, 154.8, 150.4, 150.2, 148.8, 148.7, 147.3, 143.6, 143.3, 142.9, 142.7, 142.2, 137.7, 137.5, 135.4, 135.2, 133.9, 133.2, 131.8, 131.0, 130.6, 130.2, 128.3, 127.8, 126.5, 125.6, 123.8, 123.2, 121.5, 121.4, 120.7, 118.0, 117.4, 113.6, 111.4, 93.8, 56.2, 36.0, 35.9, 31.6, 31.4, 30.4, 30.2, 26.8, 22.6, 22.5, 18.5, 18.4, 14.2, 14.1.

HRMS (ESI) for $C_{56}H_{57}IrN_3O_4$ $[M+H]^+$ calcd. 1028.3973, found 1028.3980

Enantiomeric excess established by HPLC analysis using a Chiralpak IM column, ee = 87% (HPLC: IM, 254 nm, n-hexane/isopropanol = 80:20, flow rate 1.0 mL/min, 40 °C, t_r (major) = 10.4 min, t_r (minor) = 12.6 min.)

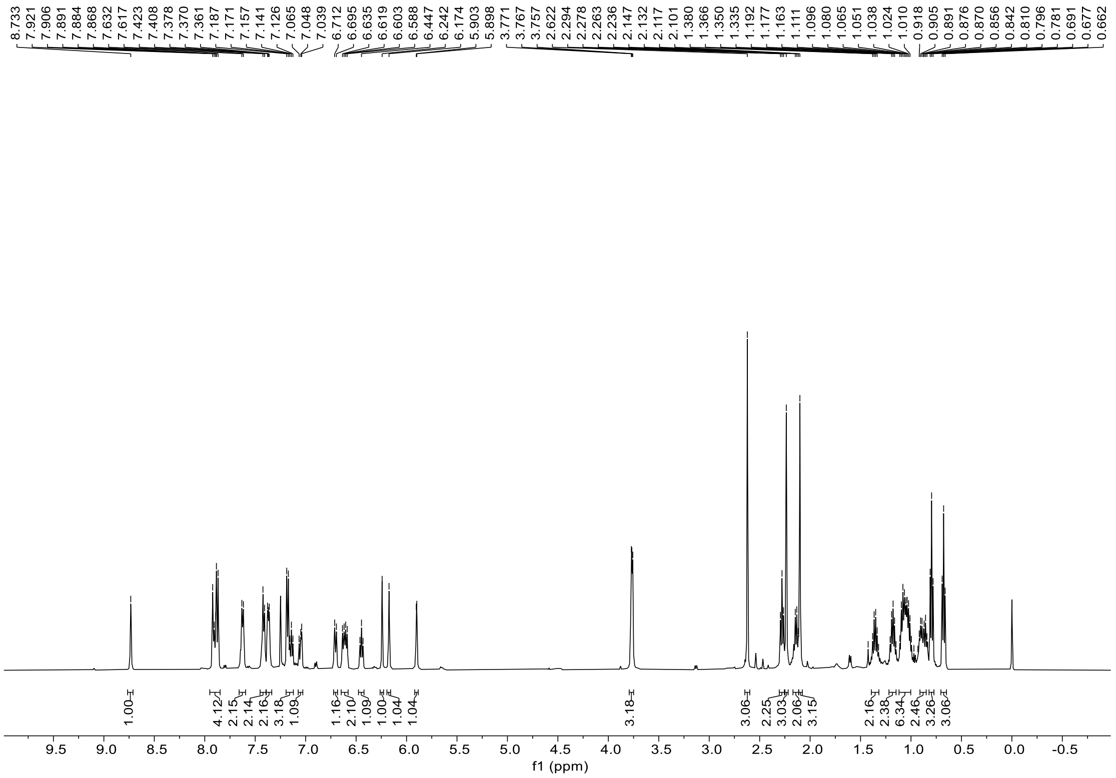

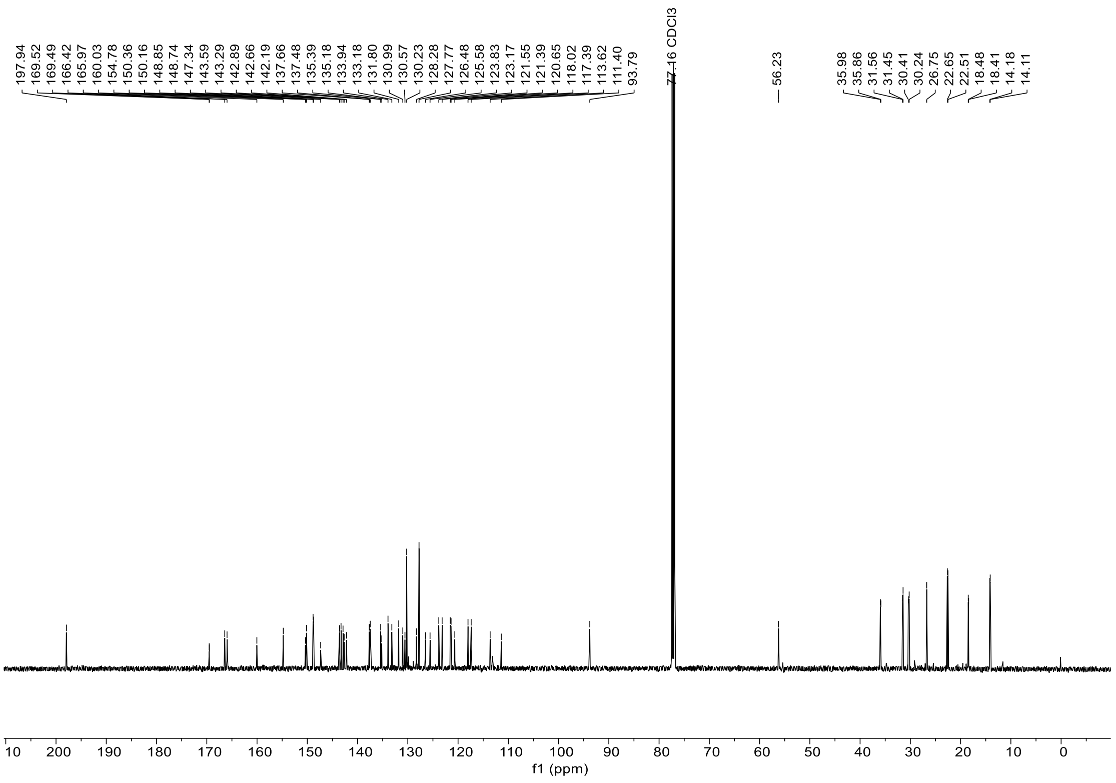

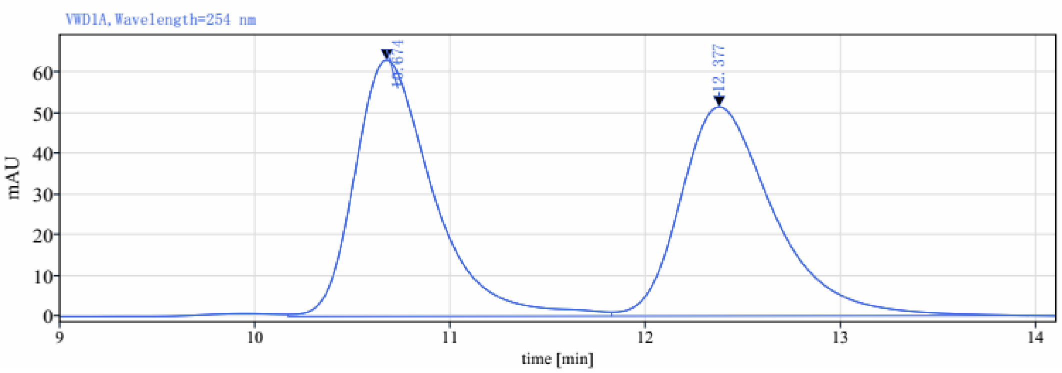

Rettime [min]	Type	Width [min]	Area	Height	Area%
10.674	VM m	1.66	1761.35	63.09	50.68
12.377	MM m	2.14	1714.12	51.47	49.32

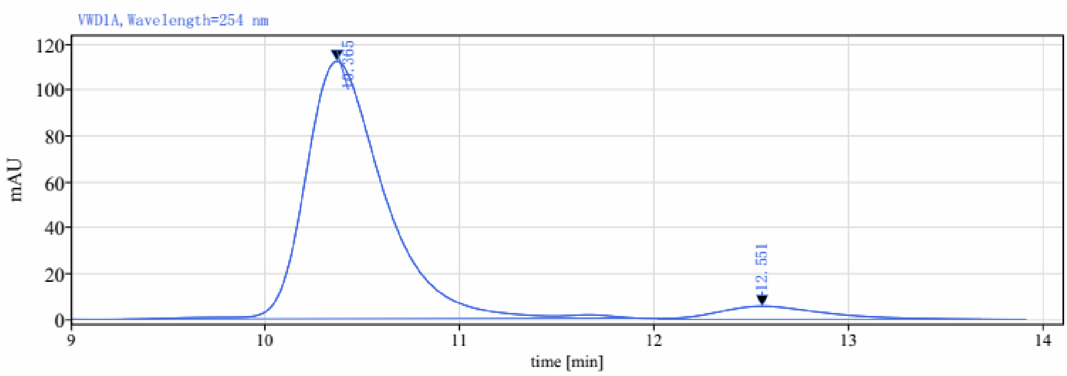

Rettime [min]	Type	Width [min]	Area	Height	Area%
10.365	MM m	2.65	3154.37	112.26	93.65
12.551	VM m	1.66	213.83	5.69	6.35

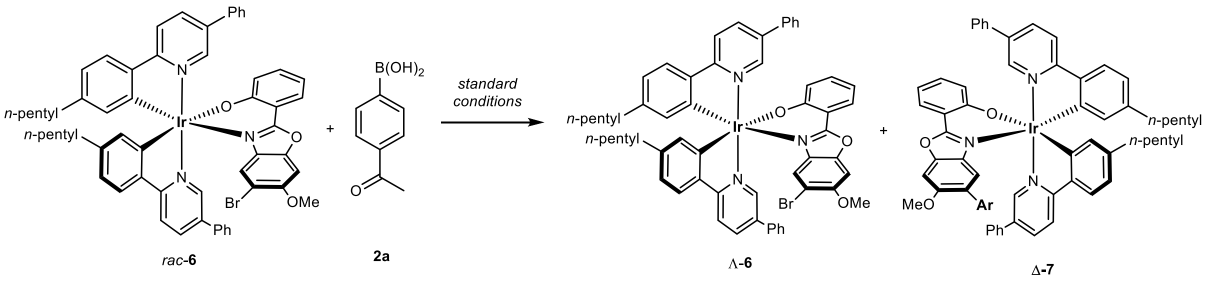

According to the general procedure, the reaction was carried out at 35 °C for 1 days to give the recovered Λ-**6** as dark red solid (22.2 mg, 40% yield) and Δ-**7** as a dark red solid (23.6 mg, 41% yield).

Purification conditions: petroleum ether/EtOAc = 8:1 to 2:1.

R_f (Λ-**6**) = 0.6 in petroleum ether/EtOAc (2:1).

R_f (Δ-**7**) = 0.5 in petroleum ether/EtOAc (2:1).

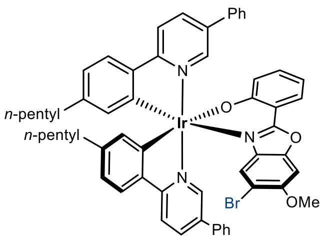

Analytical data of Λ-**6:**

^{1}H NMR (500 MHz, Chloroform-*d*) δ 9.21 (d, *J* = 2.0 Hz, 1H), 8.36 (d, *J* = 2.0 Hz, 1H), 7.87 –

7.71 (m, 4H), 7.65 (d, J = 8.5 Hz, 1H), 7.46 – 7.40 (m, 2H), 7.34 – 7.25 (m, 8H), 7.22 – 7.16 (m, 3H), 6.87 – 6.81 (m, 2H), 6.76 (dd, J = 8.0, 1.5 Hz, 1H), 6.66 (dd, J = 8.0, 1.5 Hz, 1H), 6.47 – 6.41 (m, 2H), 6.33 (d, J = 1.5 Hz, 1H), 6.13 (d, J = 1.5 Hz, 1H), 3.75 (s, 3H), 2.41 – 2.25 (m, 4H), 1.57 – 1.44 (m, 2H), 1.41 – 1.33 (m, 2H), 1.20 – 1.07 (m, 8H), 0.76 – 0.69 (m, 6H).

^{13}C NMR (126 MHz, $CDCl_3$) δ 170.0, 167.7, 167.1, 160.5, 153.6, 151.2, 149.6, 148.0, 147.5, 144.6, 144.1, 142.3, 141.7, 136.9, 136.4, 135.3, 135.2, 134.9, 134.5, 134.1, 133.8, 133.6, 132.3, 129.2, 129.1, 128.6, 128.2, 128.1, 126.8, 126.7, 126.1, 124.4, 124.0, 122.9, 121.83, 121.79, 118.6, 117.8, 113.8, 111.3, 108.0, 94.3, 56.8, 36.1, 36.0, 31.8, 31.6, 31.0, 30.4, 22.63, 22.59, 14.11, 14.09.

HRMS (ESI) for $C_{58}H_{54}BrIrN_3O_3$[M+H]$^+$ calcd. 1112.2973, found 1112.2977

Enantiomeric excess established by HPLC analysis using a Chiralpak IM column, ee = 90% (HPLC: IM, 365 nm, n-hexane/isopropanol = 80:20, flow rate 1.0 mL/min, 40 ^{o}C, t_r (major) = 11.2 min, t_r (minor) = 8.3 min.)

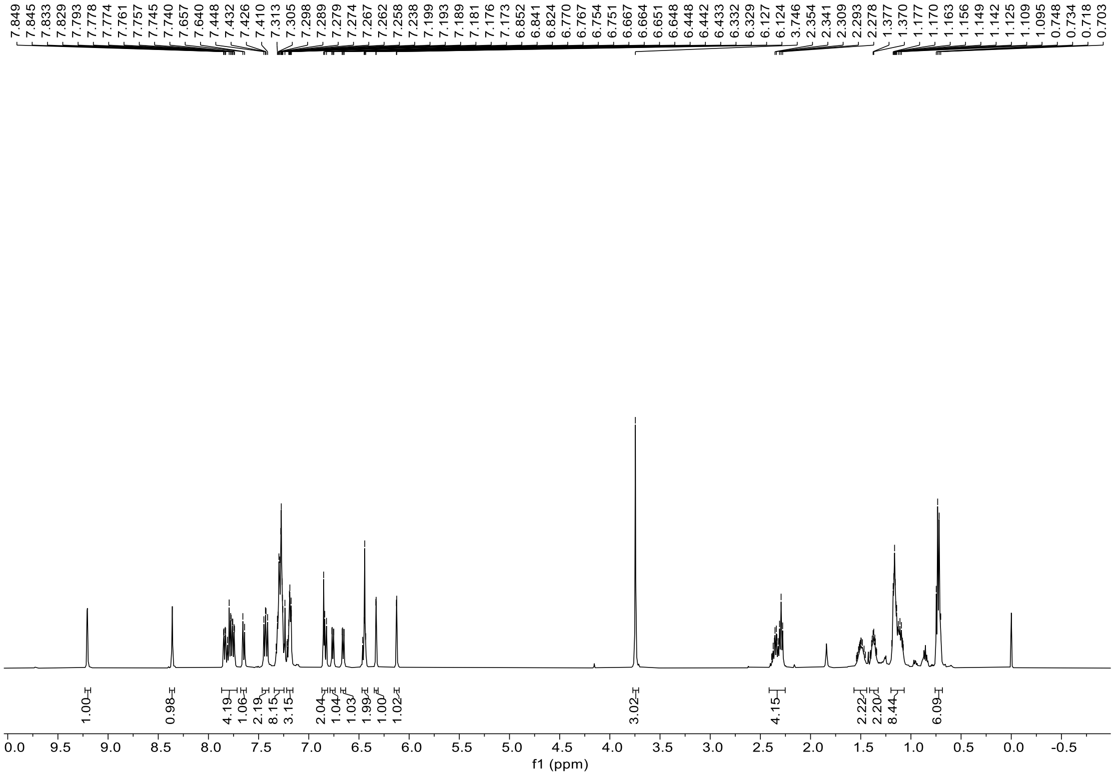

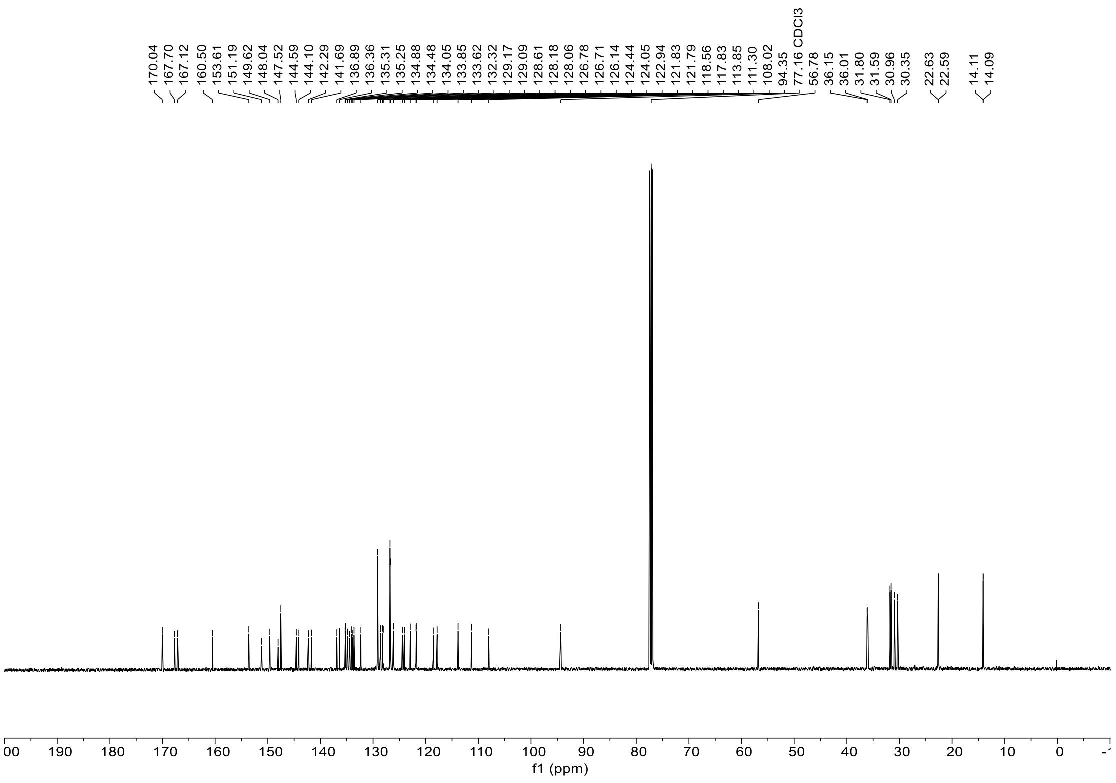

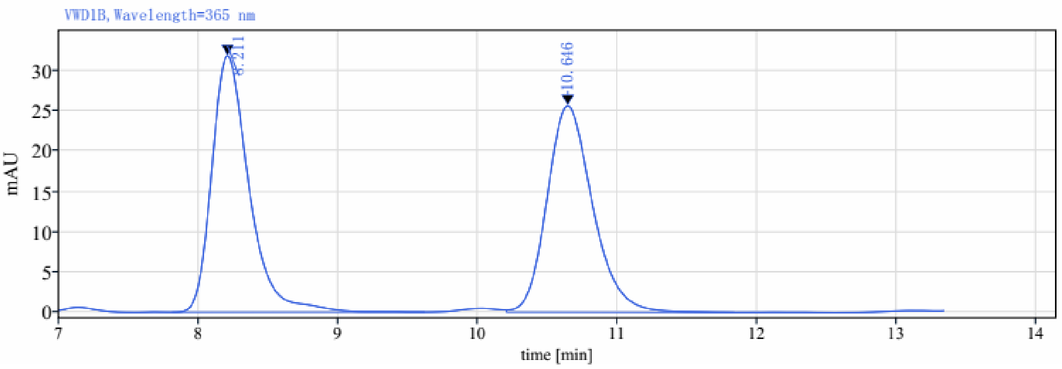

Retime [min]	Type	Width [min]	Area	Height	Area%
8.211	BB	1.81	587.32	31.70	50.32
10.646	VB	1.63	579.84	25.54	49.68

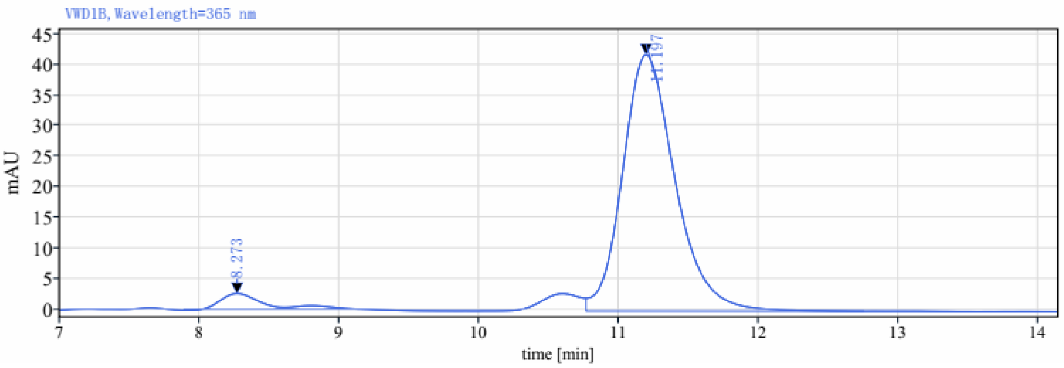

Rettime [min]	Type	Width [min]	Area	Height	Area%
8.273	MM m	1.25	56.93	2.56	4.94
11.197	VB	1.99	1095.82	41.86	95.06

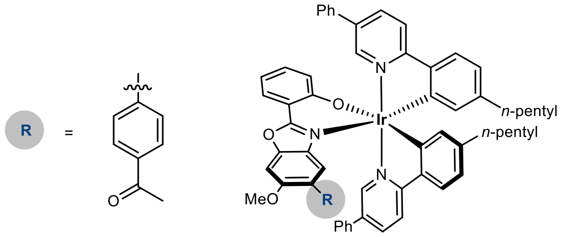

Analytical data of Δ-**7:**

^{1}H NMR (500 MHz, Chloroform-*d*) δ 9.29 – 9.23 (m, 1H), 8.42 (d, *J* = 2.5 Hz, 1H), 7.94 – 7.85 (m, 3H), 7.82 – 7.67 (m, 4H), 7.47 – 7.40 (m, 2H), 7.35 – 7.24 (m, 8H), 7.22 – 7.10 (m, 5H), 7.04 – 6.95 (m, 1H), 6.87 – 6.81 (m, 1H), 6.69 – 6.61 (m, 2H), 6.55 – 6.46 (m, 2H), 6.25 – 6.21 (m, 1H), 6.13 – 6.08 (m, 1H), 3.78 – 3.67 (m, 3H), 2.60 (s, 3H), 2.28 (t, *J* = 7.5 Hz, 2H), 2.19 – 2.06 (m, 2H), 1.41 – 1.31 (m, 2H), 1.16 – 0.94 (m, 8H), 0.90 – 0.83 (m, 2H), 0.73 (t, *J* = 7.0 Hz, 3H), 0.65 – 0.55 (m, 3H).

^{13}C NMR (126 MHz, $CDCl_3$) δ 197.8, 170.0, 167.8, 167.1, 160.2, 154.8, 151.4, 150.3, 148.5, 147.7, 147.6, 144.14, 144.08, 142.8, 142.3, 141.6, 136.8, 136.4, 135.4, 135.1, 135.0, 134.8, 134.2, 133.9, 133.8, 133.5, 132.2, 130.2, 129.1, 128.6, 128.2, 128.0, 127.8, 126.9, 126.8, 126.73, 126.71, 126.0, 124.3, 124.1, 121.8, 121.6, 120.4, 118.5, 117.8, 113.9, 111.7, 94.0, 56.2, 36.0, 35.9, 31.6, 31.5, 30.3, 30.0, 26.7, 22.6, 22.5, 14.1, 14.0.

HRMS (ESI) for $C_{66}H_{61}IrN_3O_4$ $[M+H]^+$ calcd.1152.4286, found 1152.4276

Enantiomeric excess established by HPLC analysis using a Chiralpak IM column, ee = 88% (HPLC: IM, 365 nm, *n*-hexane/isopropanol = 80:20, flow rate 1.0 mL/min, 40 °C, t_r (major) =

20.6 min, t_r (minor) = 13.3 min.)

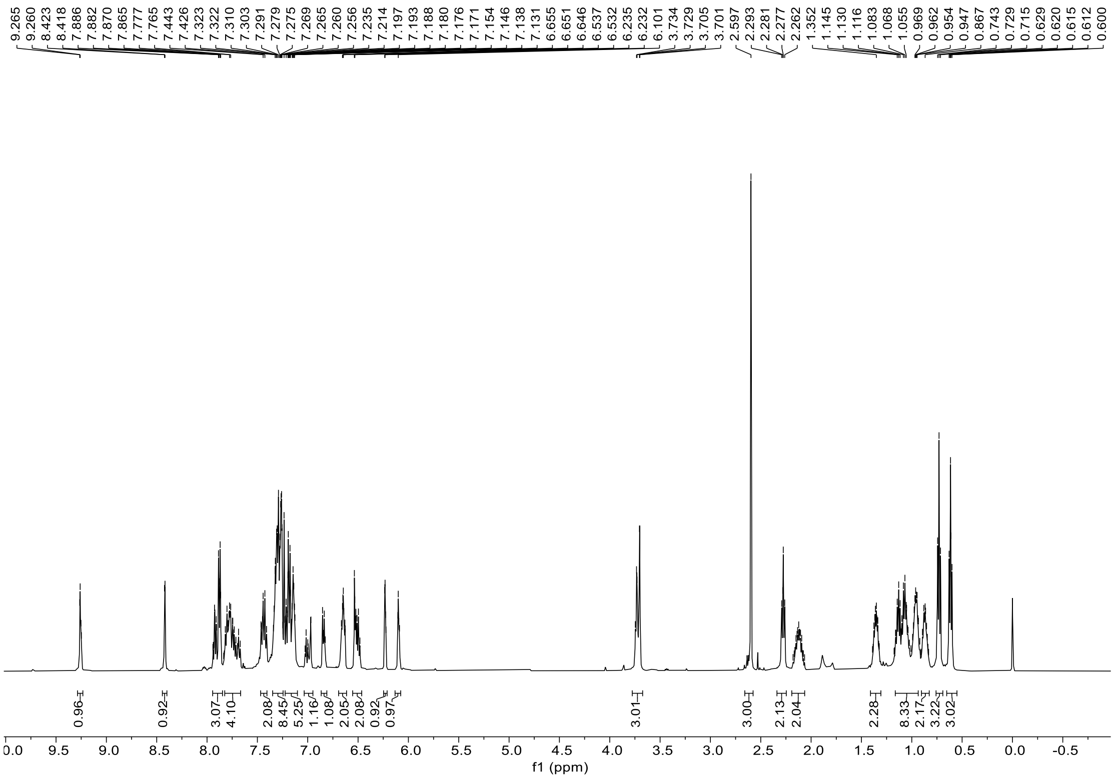

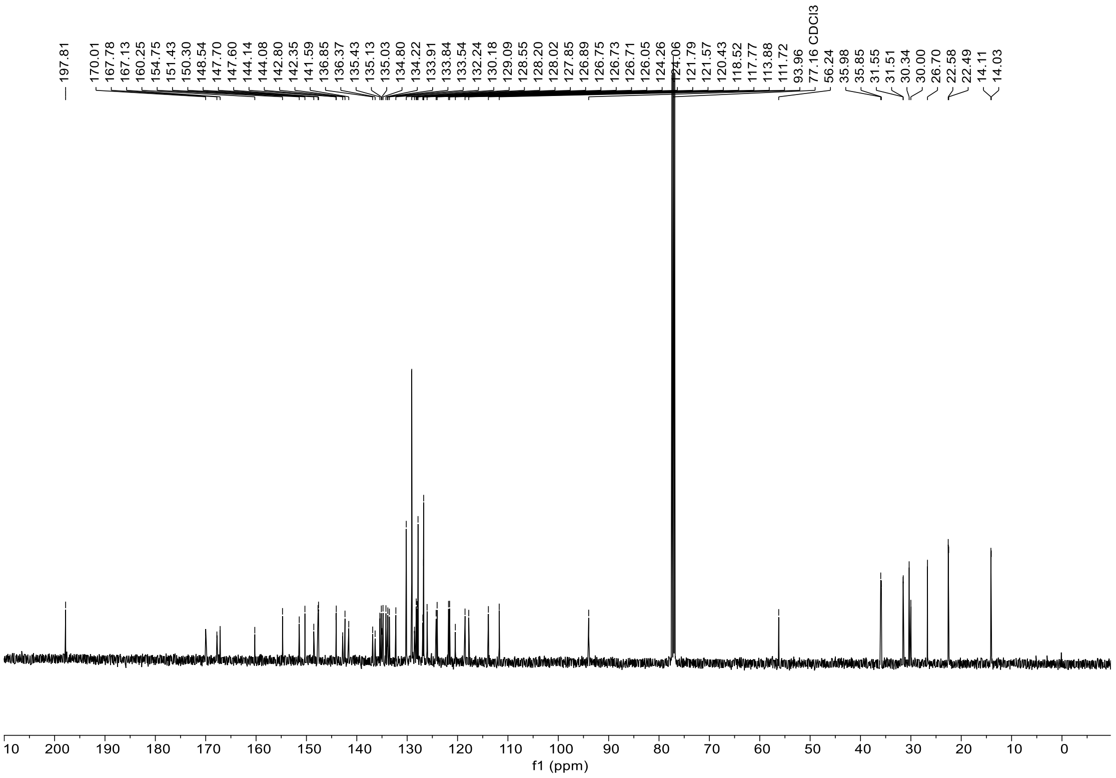

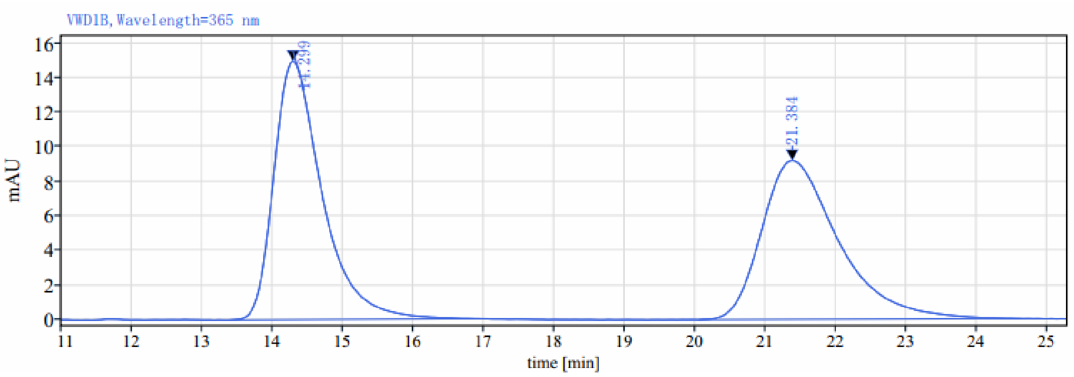

Rettime [min]	Type	Width [min]	Area	Height	Area%
14.299	BB	3.49	713.81	14.97	50.58
21.384	BB	4.57	697.47	9.21	49.42

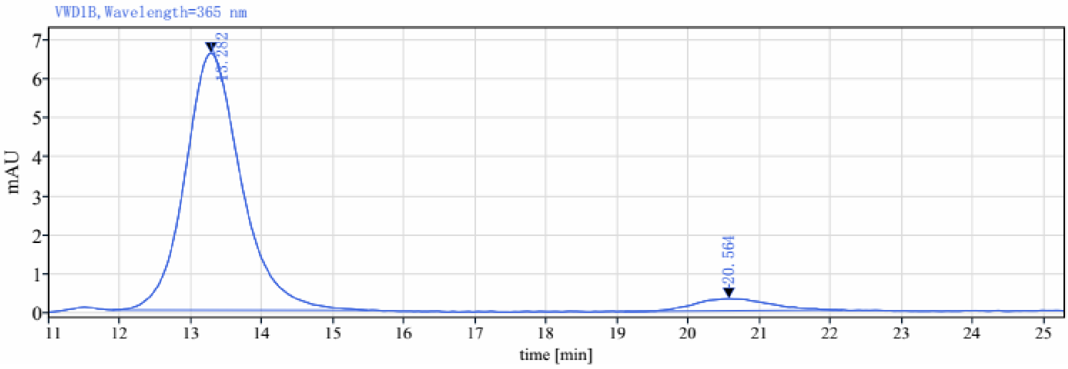

Rettime [min]	Type	Width [min]	Area	Height	Area%
13.282	BB	3.59	362.73	6.58	94.02
20.564	MM m	3.08	23.05	0.31	5.98

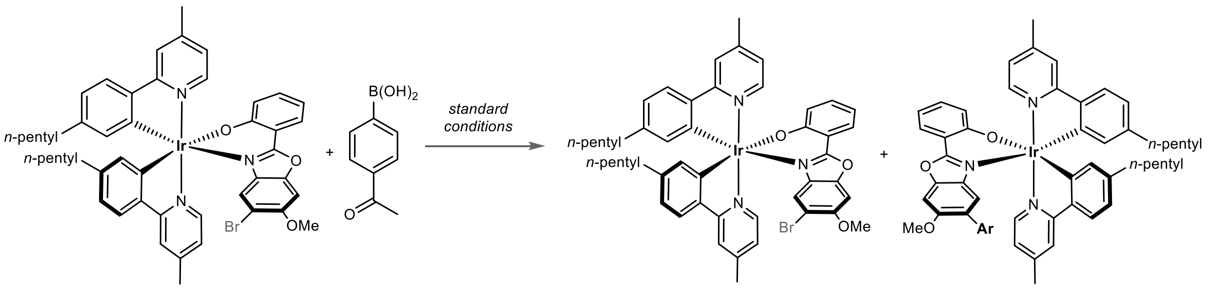

According to the general procedure, the reaction was carried out at 30 °C for 2.5 days to give the recovered Λ-**8** as dark red solid (15.3 mg, 31% yield) and Δ-**9** as a dark red solid (26.7 mg, 52% yield).

Purification conditions: petroleum ether/EtOAc = 10:1 to 4:1.

R_f (Λ-**8**) = 0.6 in petroleum ether/EtOAc (4:1).

R_f (Δ-**9**) = 0.4 in petroleum ether/EtOAc (4:1).

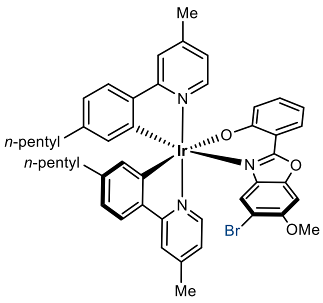

Analytical data of Λ-**8:**

^{1}H NMR (500 MHz, Chloroform-*d*) δ 8.62 (d, *J* = 6.0 Hz, 1H), 7.86 (d, *J* = 6.5 Hz, 2H), 7.60 (s, 1H), 7.53 (s, 1H), 7.47 (dd, *J* = 21.5, 8.0 Hz, 2H), 7.17 – 7.11 (m, 1H), 6.95 (s, 1H), 6.83 – 6.76 (m, 2H), 6.73 (d, *J* = 9.0 Hz, 1H), 6.69 – 6.61 (m, 2H), 6.41 (t, *J* = 7.5 Hz, 1H), 6.24 (s, 1H), 6.00 (d, *J* = 6.5 Hz, 2H), 3.84 (s, 3H), 2.47 (d, *J* = 7.5 Hz, 6H), 2.38 – 2.27 (m, 4H), 1.52 – 1.35 (m, 4H), 1.23 – 1.11 (m, 8H), 0.83 – 0.75 (m, 6H).

^{13}C NMR (126 MHz, $CDCl_3$) δ 169.5, 168.4, 168.0, 160.0, 153.5, 151.4, 149.5, 148.4, 148.2, 148.1, 148.0, 147.9, 144.3, 143.7, 142.7, 142.2, 135.5, 134.1, 133.1, 132.1, 128.4, 126.0, 124.2, 123.5, 123.3, 122.5, 122.2, 121.53, 121.51, 119.3, 118.7, 113.3, 110.4, 107.7, 94.1, 56.9, 36.1, 36.0, 31.8, 31.7, 31.1, 30.3, 22.6, 21.44, 21.41, 14.2.

HRMS (ESI) for $C_{48}H_{50}BrIrN_3O_3$ $[M+H]^+$ calcd.988.2660, found 988.2648

Enantiomeric excess established by HPLC analysis using a Chiralpak IK-3 column, ee = 97% (HPLC: IK-3, 254 nm, *n*-hexane/isopropanol = 80:20, flow rate 1.0 mL/min, 40 ^{o}C, t_r (major) = 6.5 min, t_r (minor) = 8.4 min.)

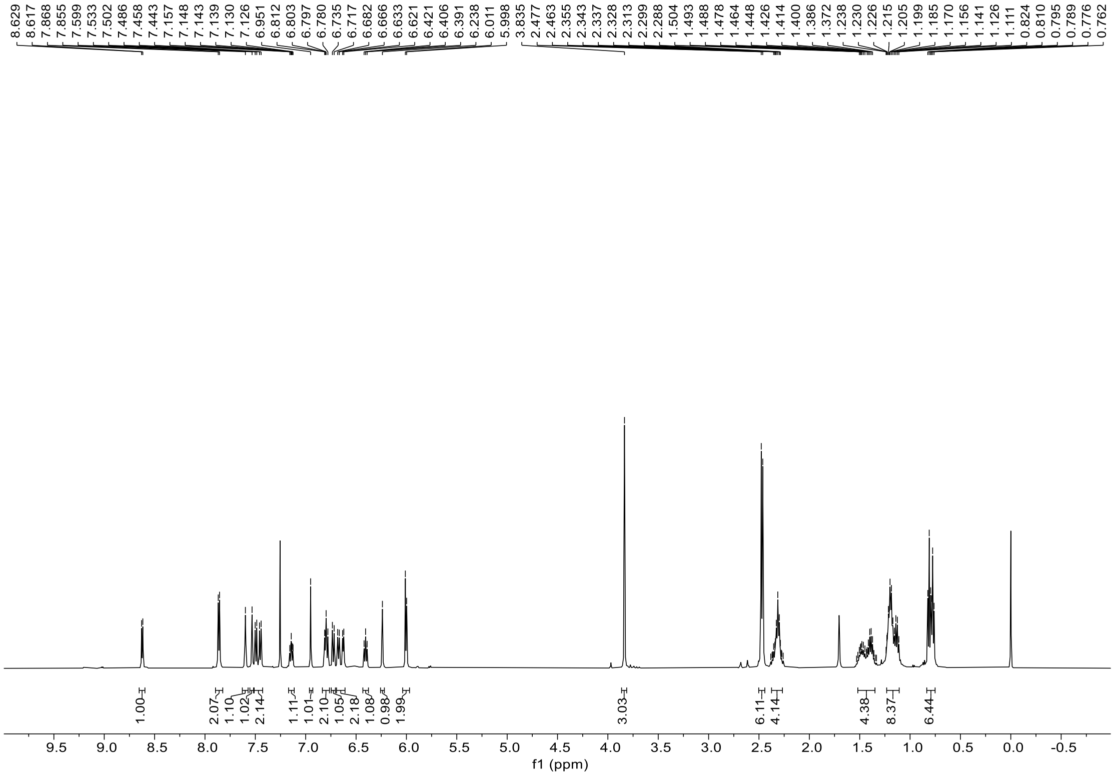
8.629 8.617 7.868 7.855 7.599 7.533 7.502 7.486 7.458 7.443 7.157 7.148 7.143 7.139 7.130 7.126 6.951 6.812 6.803 6.797 6.780 6.735 6.717 6.682 6.666 6.633 6.621 6.421 6.406 6.391 6.238 6.011 5.998 3.835 2.477 2.463 2.355 2.343 2.337 2.328 2.313 2.299 2.288 1.504 1.493 1.488 1.478 1.464 1.448 1.426 1.414 1.400 1.386 1.372 1.238 1.230 1.226 1.215 1.205 1.199 1.185 1.170 1.156 1.141 1.126 1.111 0.824 0.810 0.795 0.789 0.776 0.762
1.00 2.07 1.10 1.02 2.14 1.11 1.01 2.10 1.05 2.18 1.08 0.98 1.99 3.03 6.11 4.14 4.38 8.37 6.44
9.5 9.0 8.5 8.0 7.5 7.0 6.5 6.0 5.5 5.0 4.5 4.0 3.5 3.0 2.5 2.0 1.5 1.0 0.5 0.0 -0.5
f1 (ppm)

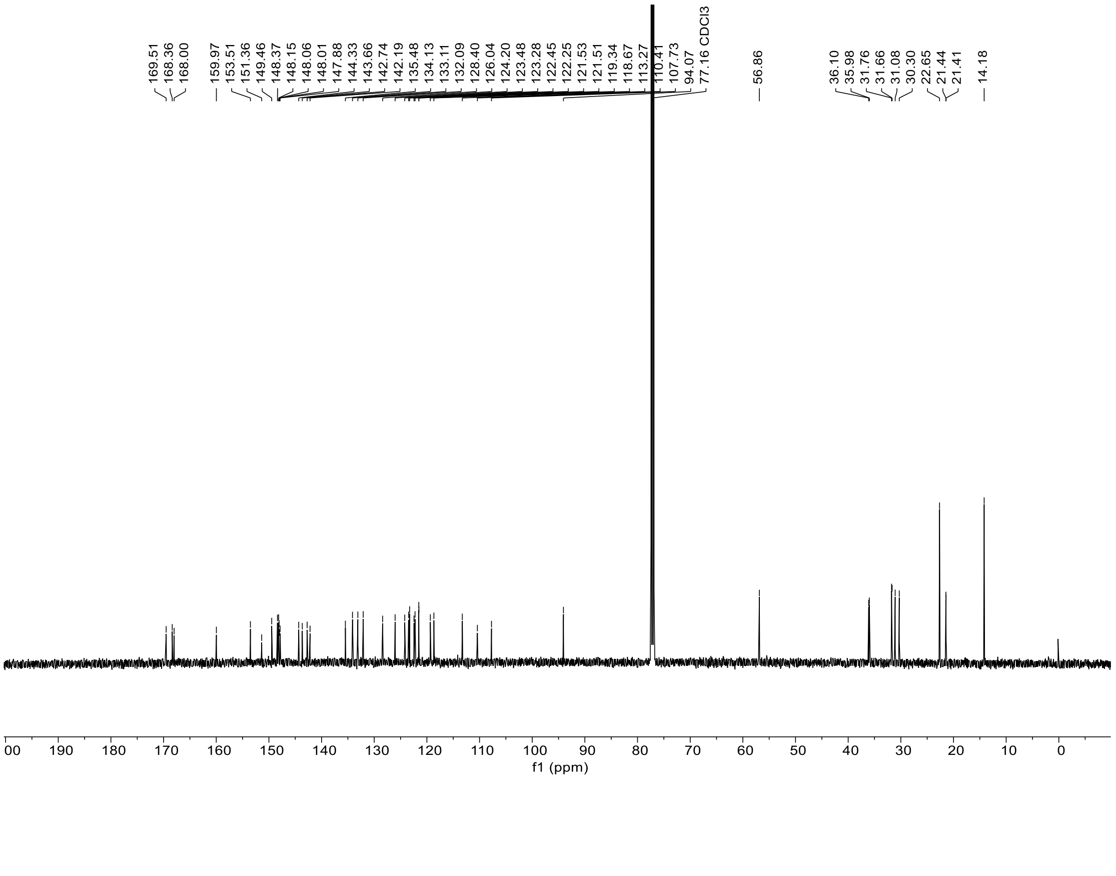
169.51 168.36 168.00 159.97 153.51 151.36 149.46 148.37 148.15 148.06 148.01 147.88 144.33 143.66 142.74 142.19 135.48 134.13 133.11 132.09 128.40 126.04 124.20 123.48 123.28 122.45 122.25 121.53 121.51 119.34 118.67 113.27 110.41 107.73 94.07 77.16 CDCl3 56.86 36.10 35.98 31.76 31.66 31.08 30.30 22.65 21.44 21.41 14.18
00 190 180 170 160 150 140 130 120 110 100 90 80 70 60 50 40 30 20 10 0
f1 (ppm)

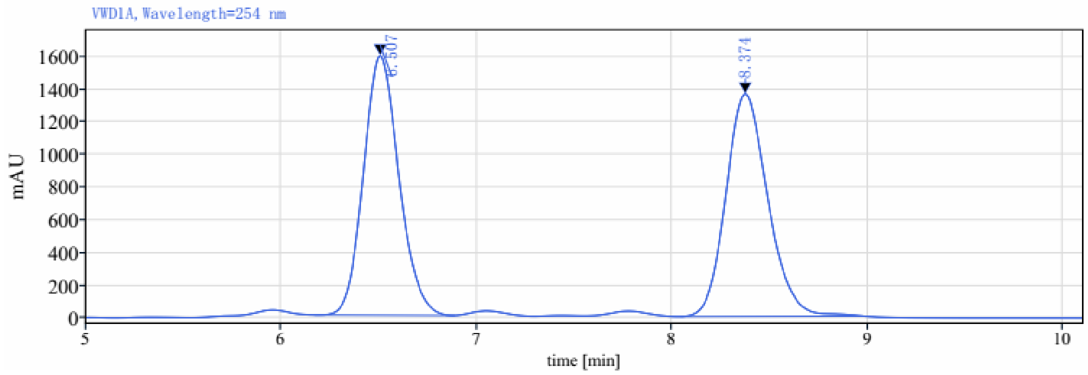

Rettime [min]	Type	Width [min]	Area	Height	Area%
6.507	MM m	0.72	20051.38	1586.88	49.71
8.374	MM m	0.94	20288.20	1361.83	50.29

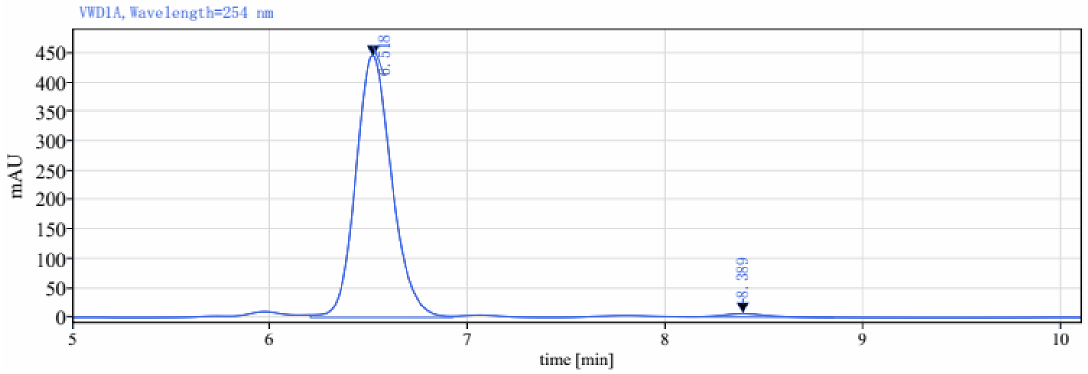

Rettime [min]	Type	Width [min]	Area	Height	Area%
6.518	MM m	0.72	5640.67	443.98	98.59
8.389	MM m	0.75	80.48	5.44	1.41

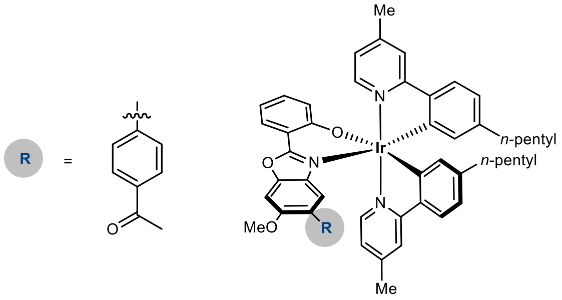

Analytical data of Δ-**9:**

^{1}H NMR (500 MHz, Chloroform-*d*) δ 8.67 (d, *J* = 6.0 Hz, 1H), 7.99 – 7.82 (m, 4H), 7.56 (d, *J* = 14.5 Hz, 2H), 7.44 (dd, *J* = 20.5, 8.0 Hz, 2H), 7.20 – 7.12 (m, 3H), 7.06 – 7.01 (m, 1H), 6.82 (d, *J* = 6.0 Hz, 1H), 6.74 (d, *J* = 9.0 Hz, 1H), 6.68 – 6.58 (m, 3H), 6.43 (t, *J* = 7.5 Hz, 1H), 6.19

(d, J = 17.0 Hz, 2H), 5.96 (s, 1H), 3.77 (s, 3H), 2.61 (s, 3H), 2.48 (s, 3H), 2.43 (s, 3H), 2.30 (t, J = 7.5 Hz, 2H), 2.16 – 2.07 (m, 2H), 1.43 – 1.33 (m, 2H), 1.22 – 1.16 (m, 2H), 1.14 – 1.07 (m, 2H), 1.05 – 0.94 (m, 4H), 0.93 – 0.85 (m, 2H), 0.80 (t, J = 7.5 Hz, 3H), 0.69 (t, J = 7.0 Hz, 3H). ^{13}C NMR (126 MHz, $CDCl_3$) δ 197.9, 169.3, 168.4, 168.0, 159.7, 154.7, 151.5, 150.1, 148.53, 148.46, 148.3, 148.1, 147.8, 143.9, 143.6, 142.74, 142.71, 142.1, 135.4, 135.3, 134.0, 133.1, 132.0, 130.3, 128.4, 127.8, 126.4, 125.8, 124.0, 123.5, 122.4, 122.3, 121.53, 121.45, 120.7, 119.3, 118.6, 113.3, 110.8, 93.7, 56.3, 36.0, 35.9, 31.58, 31.55, 30.34, 30.31, 26.7, 22.6, 22.5, 21.43, 21.37, 14.2, 14.1.

HRMS (ESI) for $C_{56}H_{57}BrIrN_3O_4$ $[M+H]^+$ calcd.1028.3973, found 1028.3963

Enantiomeric excess established by HPLC analysis using a Chiralpak IK-3 column, ee = 66% (HPLC: IK-3, 254 nm, *n*-hexane/isopropanol = 80:20, flow rate 1.0 mL/min, 40 °C, t_r (major) = 15.3 min, t_r (minor) = 13.6 min.)

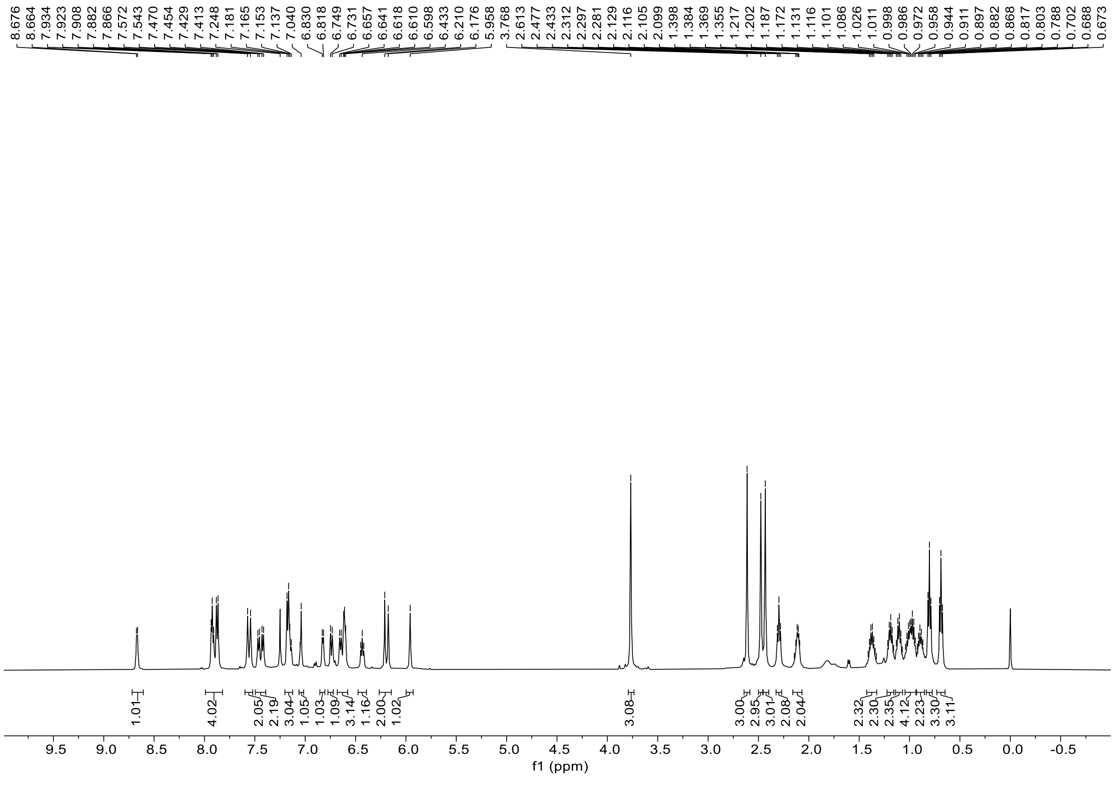

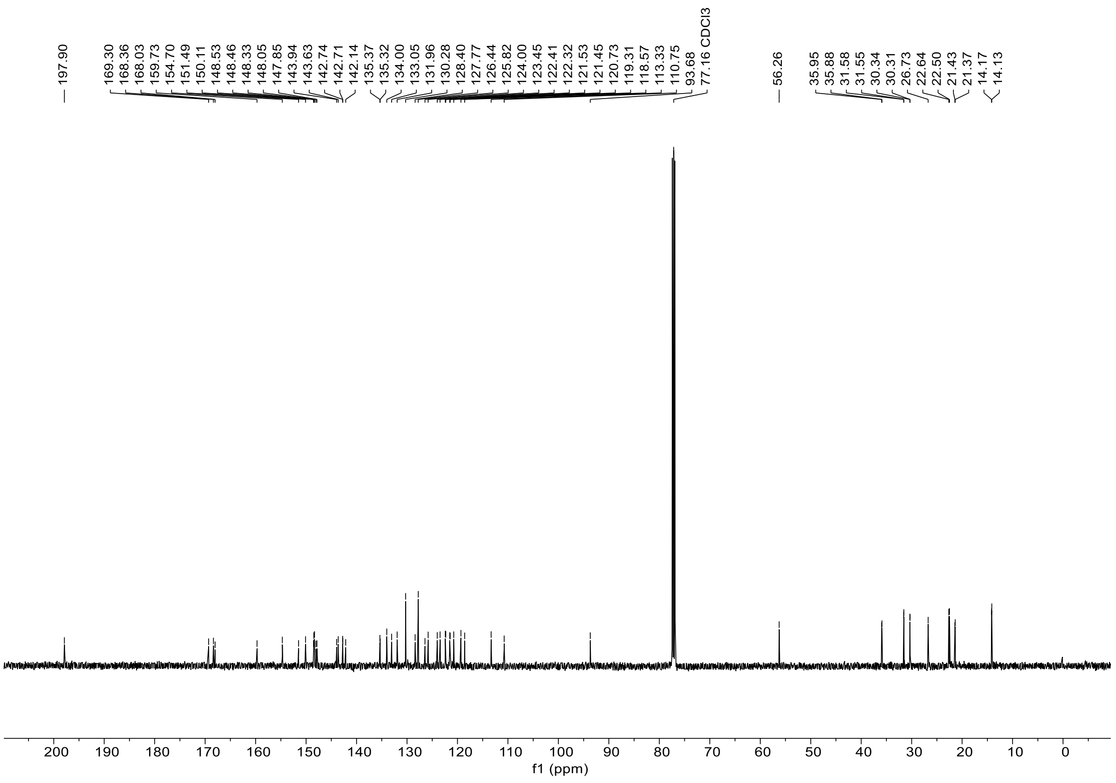

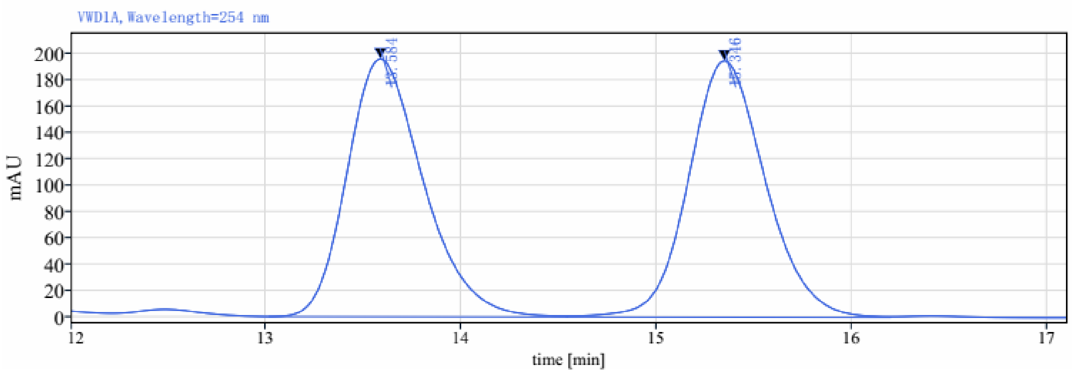

Rettime [min]	Type	Width [min]	Area	Height	Area%
13.584	BV	1.53	5337.29	196.55	49.94
15.346	VV	1.66	5349.68	195.36	50.06

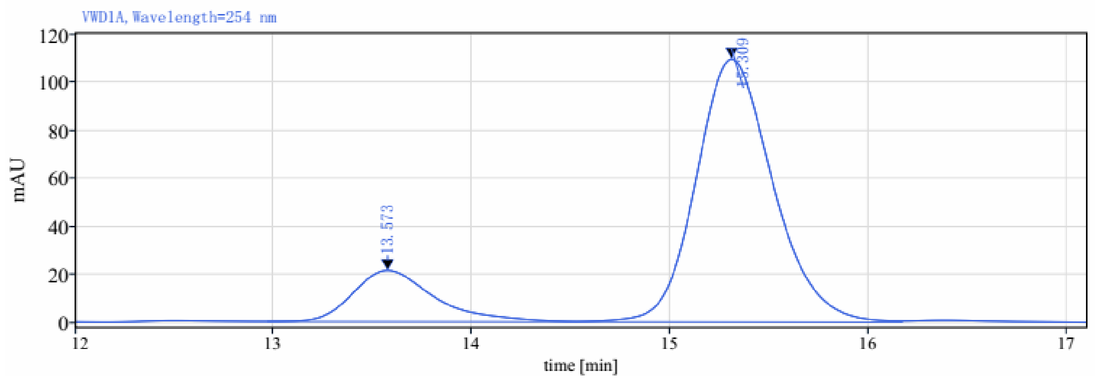

Rettime [min]	Type	Width [min]	Area	Height	Area%
13.573	BV	1.56	606.99	21.27	17.10
15.309	VV	1.65	2943.08	109.25	82.90

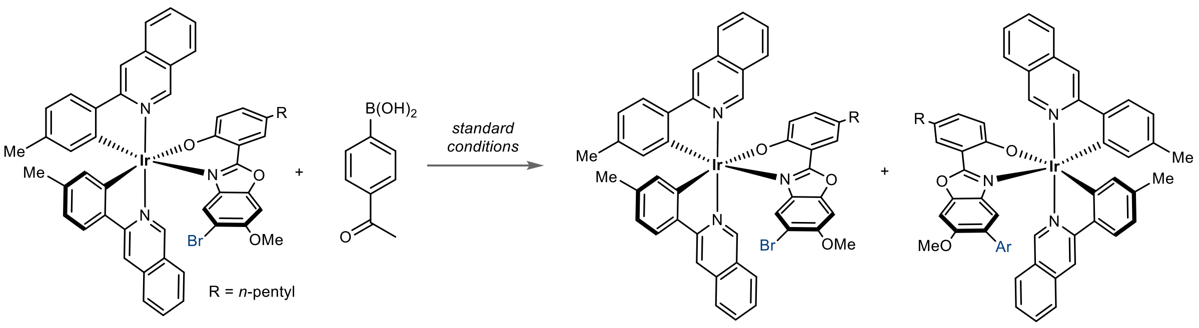

According to the general procedure, the reaction was carried out at 30 °C for 4 days to give the recovered Λ-**10** as orange red solid (21.9 mg, 43% yield) and Δ-**11** as orange red solid (21.6mg, 41% yield).

Purification conditions: petroleum ether/EtOAc = 10:1 to 2:1.

R_f (Λ-**10**) = 0.6 in petroleum ether/EtOAc (3:1).

R_f (Δ-**11**) = 0.4 in petroleum ether/EtOAc (3:1).

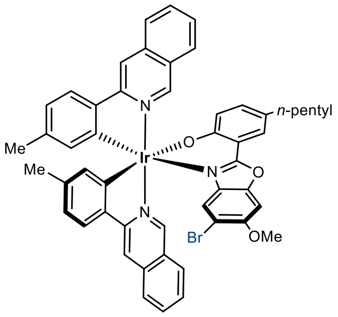

Analytical data of Λ-**10:**

^{1}H NMR (500 MHz, Chloroform-*d*) δ 9.62 (s, 1H), 8.78 (s, 1H), 8.10 (s, 1H), 8.06 (s, 1H), 7.81 (d, *J* = 8.5 Hz, 2H), 7.75 (d, *J* = 8.0 Hz, 1H), 7.63 – 7.53 (m, 6H), 7.41 (t, *J* = 7.5 Hz, 1H), 7.35 (t, *J* = 7.5 Hz, 1H), 6.98 – 6.93 (m, 2H), 6.82 (d, *J* = 8.0 Hz, 1H), 6.72 – 6.67 (m, 2H), 6.26 (s, 1H), 6.22 (s, 1H), 5.96 (s, 1H), 3.85 – 3.78 (m, 3H), 2.44 – 2.39 (m, 2H), 2.03 (s, 3H), 1.96 (s, 3H), 1.55 – 1.48 (m, 2H), 1.31 – 1.24 (m, 4H), 0.87 – 0.84 (m, 3H).

^{13}C NMR (126 MHz, $CDCl_3$) δ 168.2, 162.0, 161.6, 160.6, 153.5, 152.12, 152.06, 149.6, 149.3, 146.3, 142.4, 141.7, 138.7, 138.3, 136.6, 136.5, 135.4, 134.9, 134.4, 132.9, 131.7, 131.6, 128.2, 127.6, 127.5, 127.4, 126.8, 126.6, 126.51, 126.49, 126.3, 125.9, 123.4, 123.2, 123.1, 123.0, 122.7, 113.8, 113.3, 109.8, 107.8, 94.2, 56.8, 35.0, 31.6, 31.4, 22.7, 21.9, 21.7, 14.2.

HRMS (ESI) for $C_{51}H_{44}BrIrN_3O_3$ $[M+H]^+$ calcd.1018.2190, found 1018.2165

Enantiomeric excess established by HPLC analysis using a Chiralpak IK-3 column, ee = 92% (HPLC: IK-3, 254 nm, *n*-hexane/isopropanol = 80:20, flow rate 1.0 mL/min, 40 °C, t_r (major) = 12.3 min, t_r (minor) = 14.3 min.)

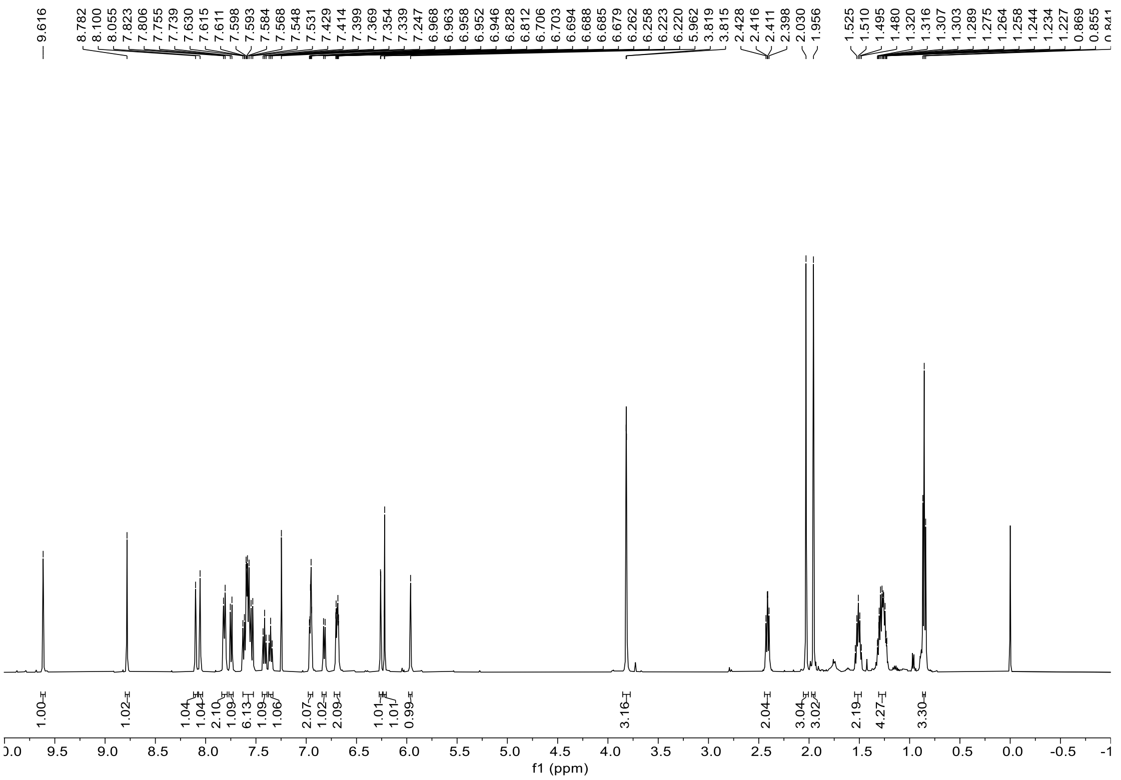

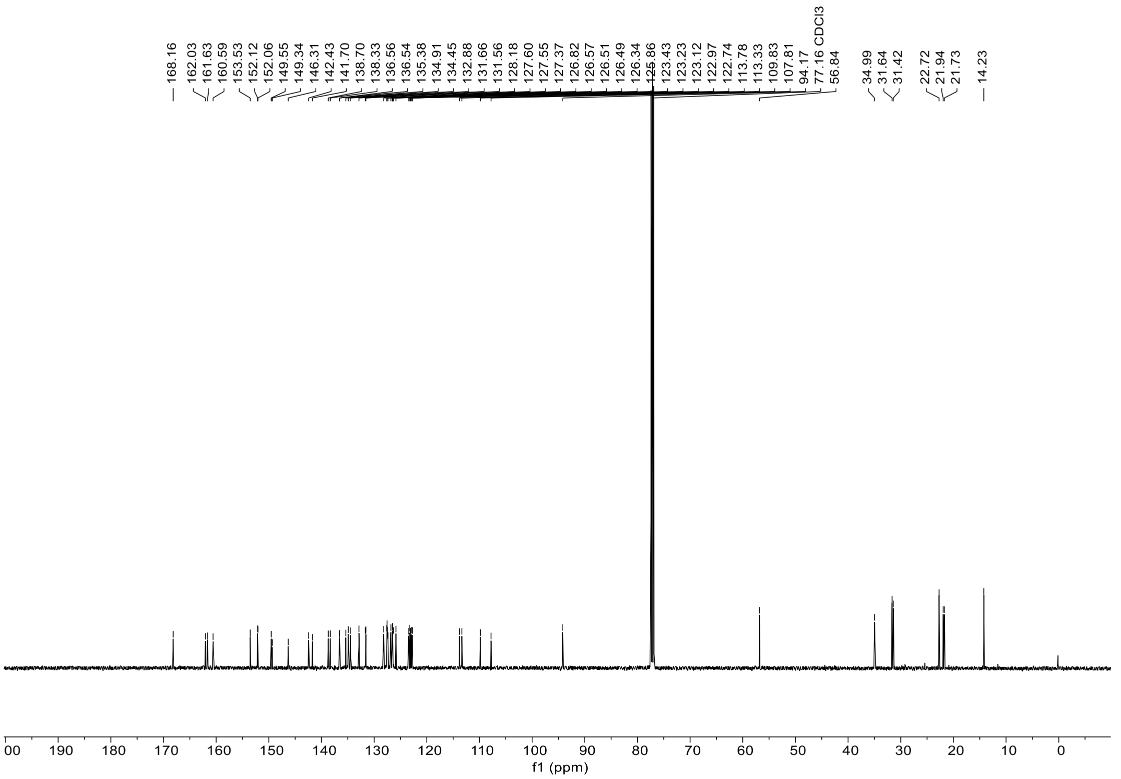

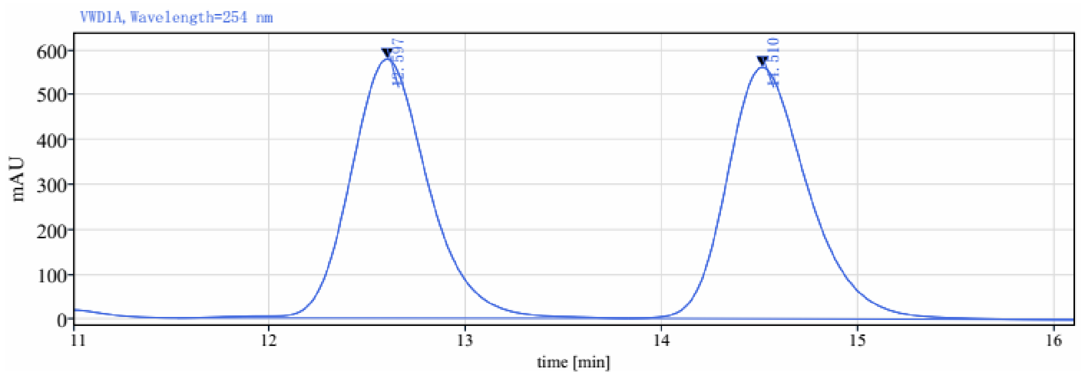

Rettime [min]	Type	Width [min]	Area	Height	Area%
12.597	BB	2.30	15914.48	576.34	49.73
14.510	BB	2.36	16087.92	559.34	50.27

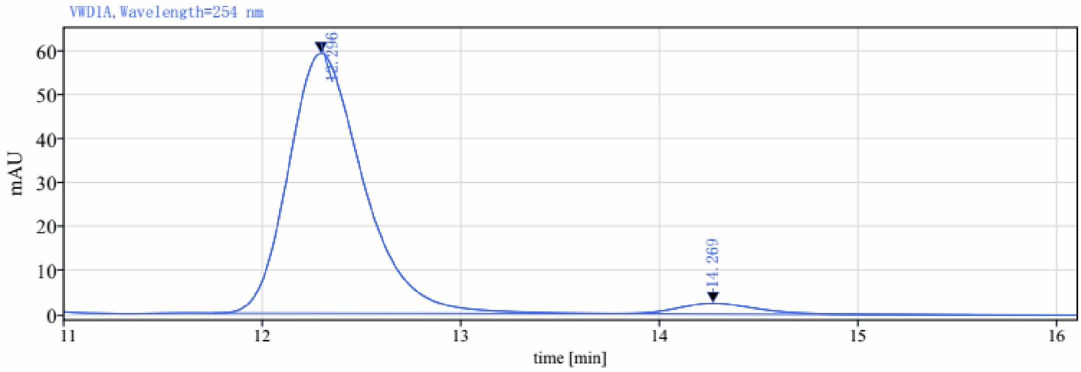

Rettime [min]	Type	Width [min]	Area	Height	Area%
12.296	BB	2.01	1543.73	59.03	95.99
14.269	BB	1.79	64.56	2.38	4.01

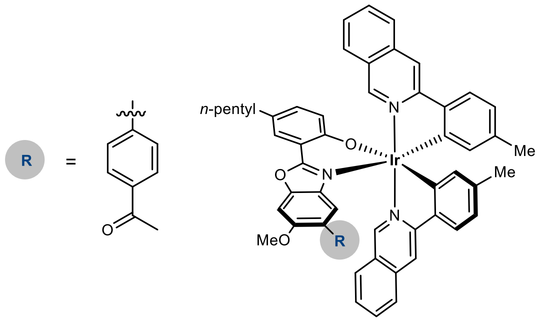

Analytical data of Δ-**11:**

^{1}H NMR (500 MHz, Chloroform-*d*) δ 9.67 (s, 1H), 8.86 (s, 1H), 8.08 (s, 1H), 8.00 (s, 1H), 7.88 (d, *J* = 8.5 Hz, 2H), 7.83 – 7.72 (m, 3H), 7.65 (d, *J* = 2.5 Hz, 1H), 7.61 – 7.54 (m, 4H), 7.50 (d, *J* = 8.0 Hz, 1H), 7.42 (t, *J* = 7.5 Hz, 1H), 7.38 – 7.33 (m, 1H), 7.24 – 7.18 (m, 2H), 7.07 (s, 1H),

6.96 (dd, J = 9.0, 2.5 Hz, 1H), 6.71 – 6.63 (m, 3H), 6.42 (s, 1H), 6.25 (d, J = 1.5 Hz, 1H), 5.91 (d, J = 2.0 Hz, 1H), 3.78 (s, 3H), 2.61 (s, 3H), 2.48 – 2.41 (m, 2H), 1.94 (s, 3H), 1.91 (s, 3H), 1.56 – 1.49 (m, 2H), 1.32 – 1.24 (m, 4H), 0.87 (t, J = 6.5 Hz, 3H).

^{13}C NMR (126 MHz, $CDCl_3$) δ 198.1, 168.0, 162.1, 161.6, 160.5, 154.8, 152.3, 152.26, 150.2, 149.5, 146.7, 142.7, 142.5, 141.6, 138.4, 138.3, 136.53, 136.5, 135.4, 135.2, 134.9, 134.4, 132.8, 131.7, 131.6, 130.2, 128.2, 127.73, 127.68, 127.6, 127.5, 127.4, 126.9, 126.6, 126.5, 126.4, 126.3, 125.6, 123.3, 123.1, 122.9, 122.7, 120.6, 113.7, 113.3, 110.4, 93.8, 56.3, 35.0, 31.6, 31.5, 26.8, 22.7, 21.9, 21.7, 14.2.

HRMS (ESI) for $C_{59}H_{51}IrN_3O_4$ $[M+H]^+$ calcd.1058.3504, found 1058.3509

Enantiomeric excess established by HPLC analysis using a Chiralpak IK-3 column, ee = 94% (HPLC: IK-3, 254 nm, *n*-hexane/isopropanol = 80:20, flow rate 1.0 mL/min, 40 °C, t_r (major) = 34.8 min, t_r (minor) = 38.7 min.)

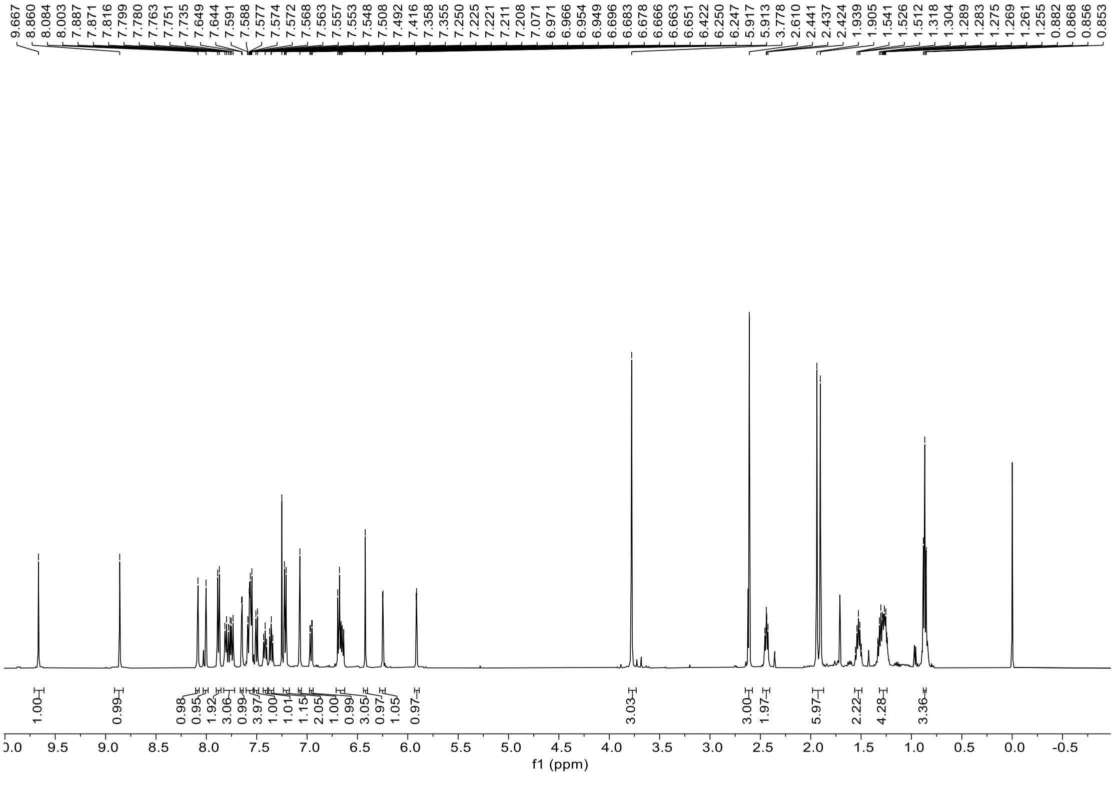

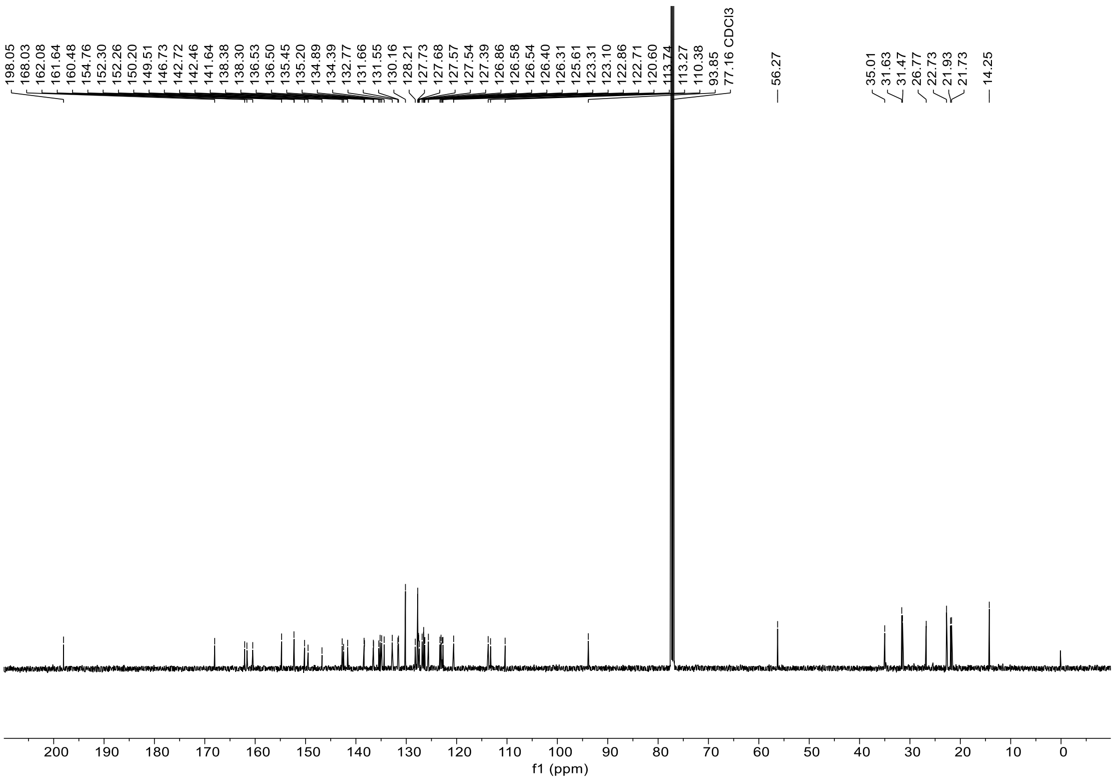

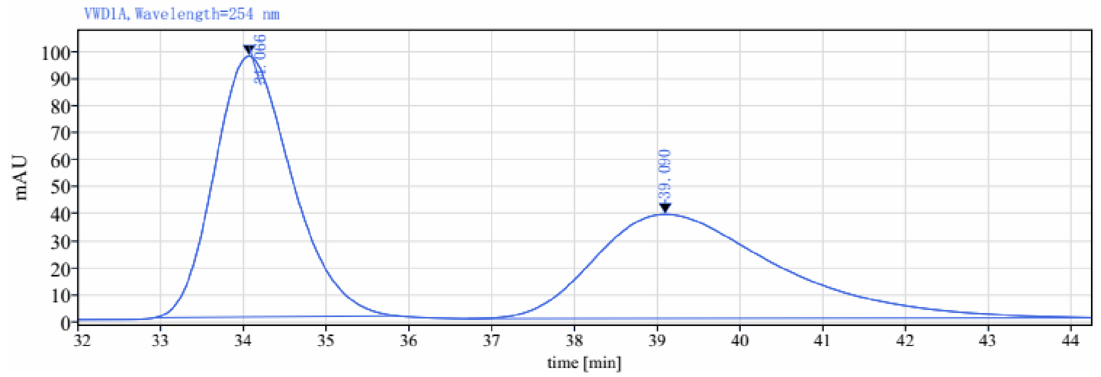

Rettime [min]	Type	Width [min]	Area	Height	Area%
34.066	MM m	2.94	6273.39	96.52	51.41
39.090	MM m	7.62	5928.64	38.55	48.59

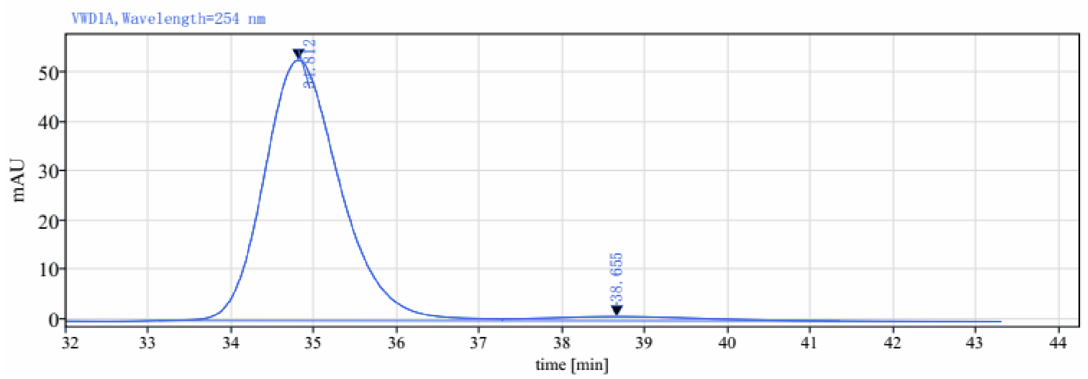

Rettime [min]	Type	Width [min]	Area	Height	Area%
34.812	MM m	4.15	3374.87	52.84	96.81
38.655	MM m	4.08	111.25	0.83	3.19

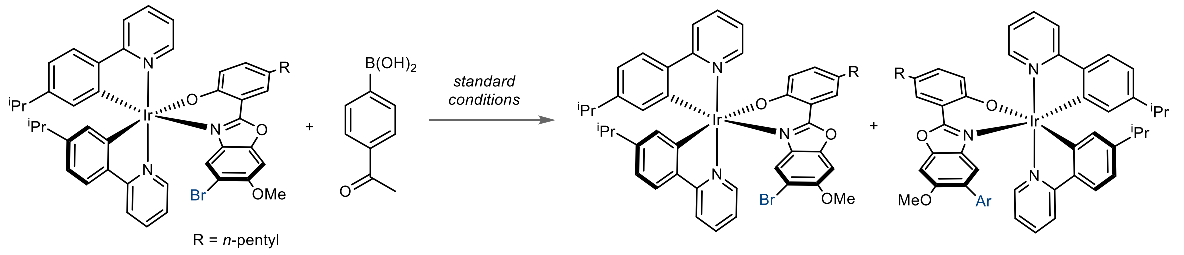

rac-**12** **2a** Λ-**12** Δ-**13**

According to the general procedure, the reaction was carried out at 30 °C for 4 days to give the recovered Λ-**12** as yellow solid (20.5 mg, 42% yield) and Δ-**13** as yellow solid (23.8 mg, 47% yield).

Purification conditions: petroleum ether/EtOAc = 10:1 to 4:1.

R_f (Λ-**12**) = 0.6 in petroleum ether/EtOAc (4:1).

R_f (Δ-**13**) = 0.5 in petroleum ether/EtOAc (4:1).

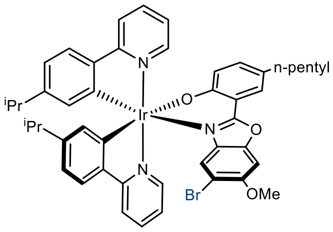

Analytical data of Λ-**12:**

^{1}H NMR (500 MHz, Chloroform-*d*) δ 8.83 (d, *J* = 5.5 Hz, 1H), 8.06 (d, *J* = 6.0 Hz, 1H), 7.78 – 7.63 (m, 3H), 7.59 – 7.41 (m, 4H), 7.06 – 6.99 (m, 1H), 6.99 – 6.92 (m, 2H), 6.85 (d, *J* = 8.0

Hz, 1H), 6.80 – 6.66 (m, 3H), 6.26 (s, 1H), 6.01 (s, 1H), 5.90 (s, 1H), 3.81 (s, 3H), 2.62 – 2.44 (m, 4H), 1.58 (p, *J* = 7.5 Hz, 2H), 1.38 – 1.29 (m, 4H), 1.06 (t, *J* = 6.5 Hz, 6H), 1.02 – 0.98 (m, 6H), 0.92 – 0.87 (m, 3H).

^{13}C NMR (126 MHz, $CDCl_3$) δ 169.0, 168.6, 167.9, 160.2, 153.4, 151.5, 150.3, 149.6, 149.4, 149.0, 148.9, 148.2, 142.9, 142.3, 136.7, 136.5, 135.4, 134.3, 132.2, 130.1, 127.4, 126.8, 125.7, 124.5, 123.8, 123.0, 121.2, 121.0, 119.4, 119.3, 118.5, 117.9, 109.4, 107.7, 94.1, 56.8, 35.1, 34.0, 33.9, 31.8, 31.5, 24.4, 23.9, 23.8, 23.7, 22.8, 14.3.

HRMS (ESI) for $C_{47}H_{48}BrIrN_3O_3$ $[M+H]^+$ calcd.974.2503, found 974.2496

Enantiomeric excess established by HPLC analysis using a Chiralpak IK-3 column, ee = 97% (HPLC: IK-3, 254 nm, *n*-hexane/isopropanol = 80:20, flow rate 1.0 mL/min, 40 ^{o}C, t_r (major) = 5.5 min, t_r (minor) = 6.3 min.)

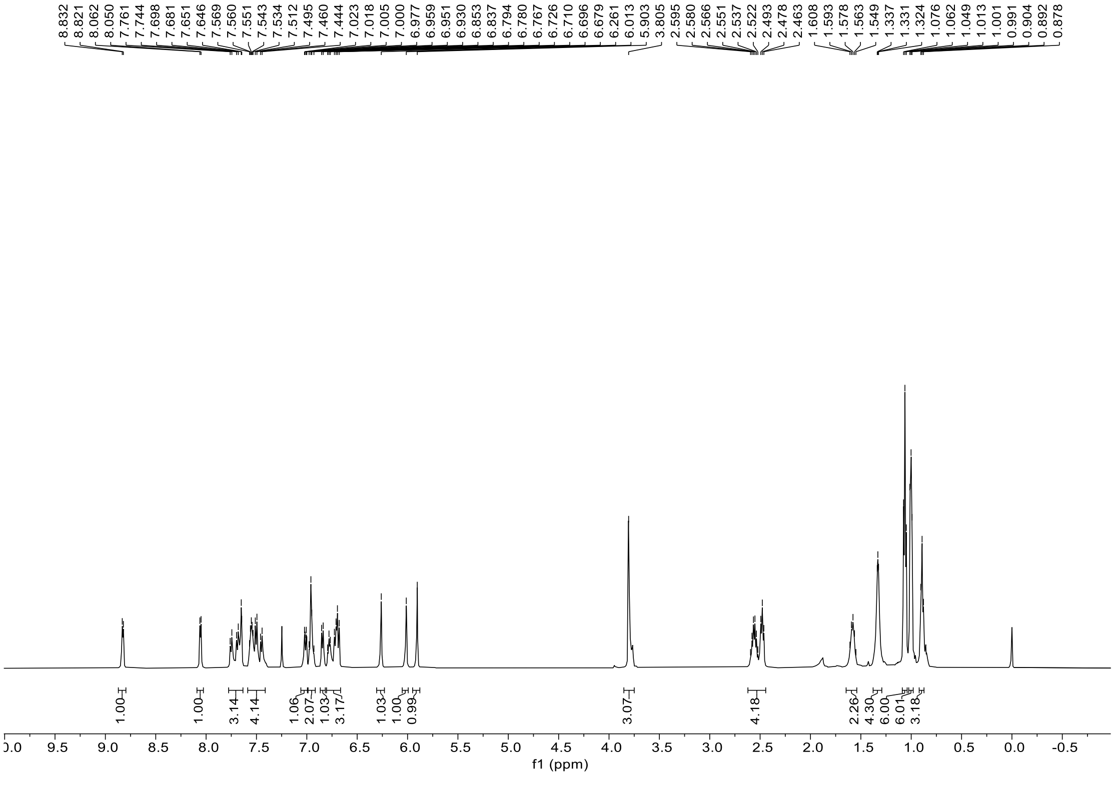

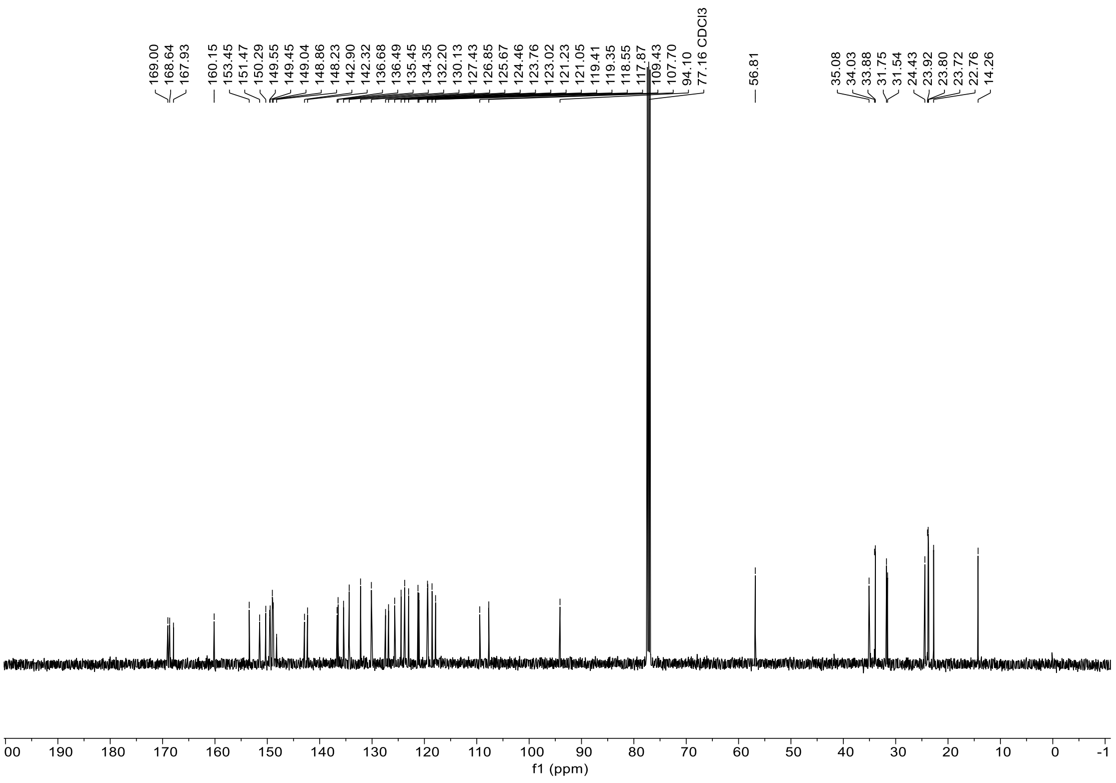

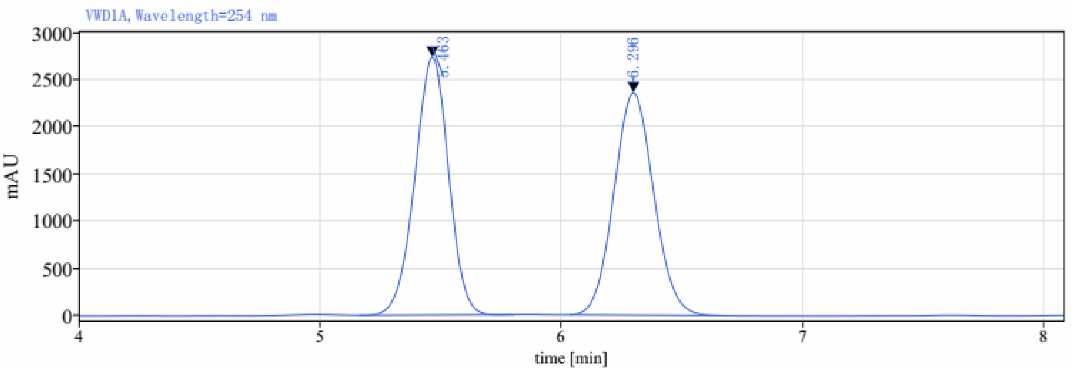

Rettime [min]	Type	Width [min]	Area	Height	Area%
5.463	MM m	0.64	26544.61	2729.41	49.53
6.296	MM m	0.63	27050.81	2354.70	50.47

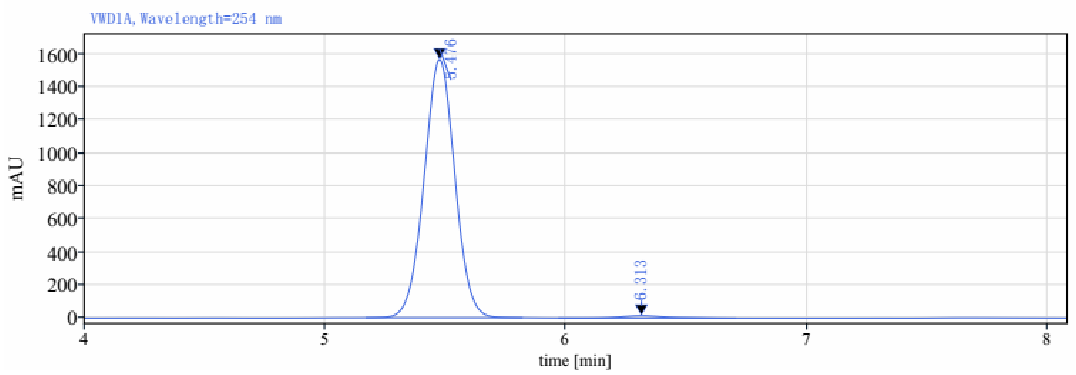

Rettime [min]	Type	Width [min]	Area	Height	Area%
5.476	MM m	0.65	13923.12	1565.34	98.67
6.313	MM m	0.74	187.23	14.38	1.33

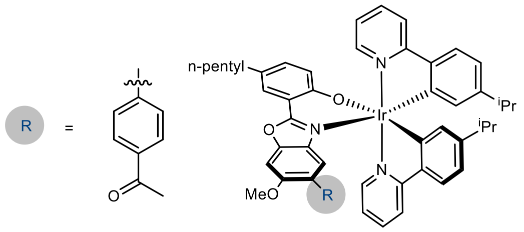

Analytical data of Δ-**13:**

^{1}H NMR (500 MHz, Chloroform-*d*) δ 8.90 (d, *J* = 5.5 Hz, 1H), 8.11 (d, *J* = 6.0 Hz, 1H), 7.88 (d, *J* = 8.0 Hz, 2H), 7.79 – 7.69 (m, 3H), 7.63 – 7.49 (m, 3H), 7.43 (d, *J* = 8.0 Hz, 1H), 7.15 (d, *J* = 8.0 Hz, 2H), 7.10 – 7.06 (m, 1H), 7.04 – 6.98 (m, 2H), 6.78 (t, *J* = 7.0 Hz, 1H), 6.72 – 6.61 (m, 3H), 6.17 (s, 1H), 6.04 (s, 1H), 5.96 (s, 1H), 3.76 (d, *J* = 2.5 Hz, 3H), 2.62 (s, 3H), 2.56 – 2.45 (m, 3H), 2.31 (p, *J* = 7.0 Hz, 1H), 1.60 (p, *J* = 7.5 Hz, 2H), 1.38 – 1.29 (m, 4H), 1.02 – 0.96 (m, 6H), 0.90 (t, *J* = 6.5 Hz, 3H), 0.69 (d, *J* = 7.0 Hz, 3H), 0.56 (d, *J* = 7.0 Hz, 3H).

^{13}C NMR (126 MHz, $CDCl_3$) δ 198.0, 169.0, 168.7, 167.5, 159.9, 154.7, 151.6, 150.1, 149.9, 149.6, 149.1, 149.0, 148.4, 142.9, 142.7, 142.4, 136.7, 136.5, 135.4, 135.3, 134.3, 132.2, 130.4, 130.0, 127.8, 127.7, 126.9, 126.6, 125.2, 124.2, 123.8, 121.21, 121.16, 120.5, 119.48, 119.45, 118.5, 117.8, 109.8, 93.7, 56.2, 35.1, 33.9, 33.6, 31.8, 31.6, 26.8, 23.9, 23.7, 23.0, 22.8, 14.3.

HRMS (ESI) for $C_{55}H_{55}IrN_3O_4$ $[M+H]^+$ calcd.1014.3817, found 1014.3809

Enantiomeric excess established by HPLC analysis using a Chiralpak IM column, ee = 85% (HPLC: IM, 254 nm, *n*-hexane/isopropanol = 80:20, flow rate 1.0 mL/min, 40 °C, t_r (major) = 11.2 min, t_r (minor) = 9.1 min.)

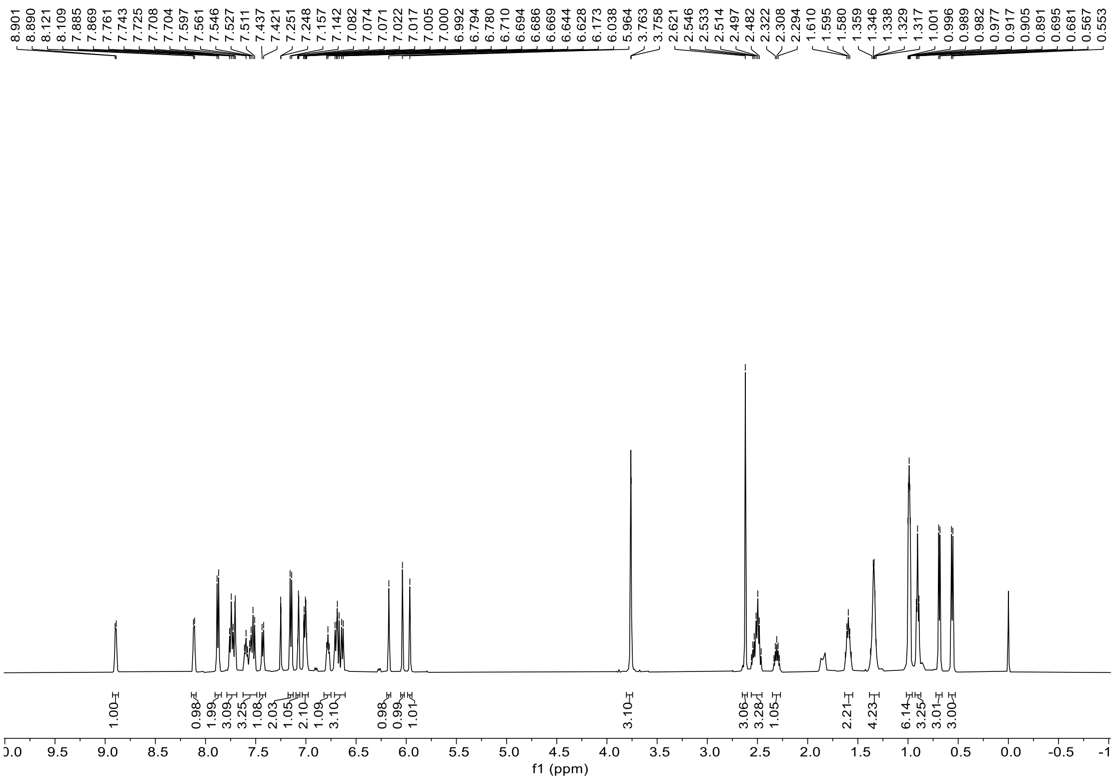

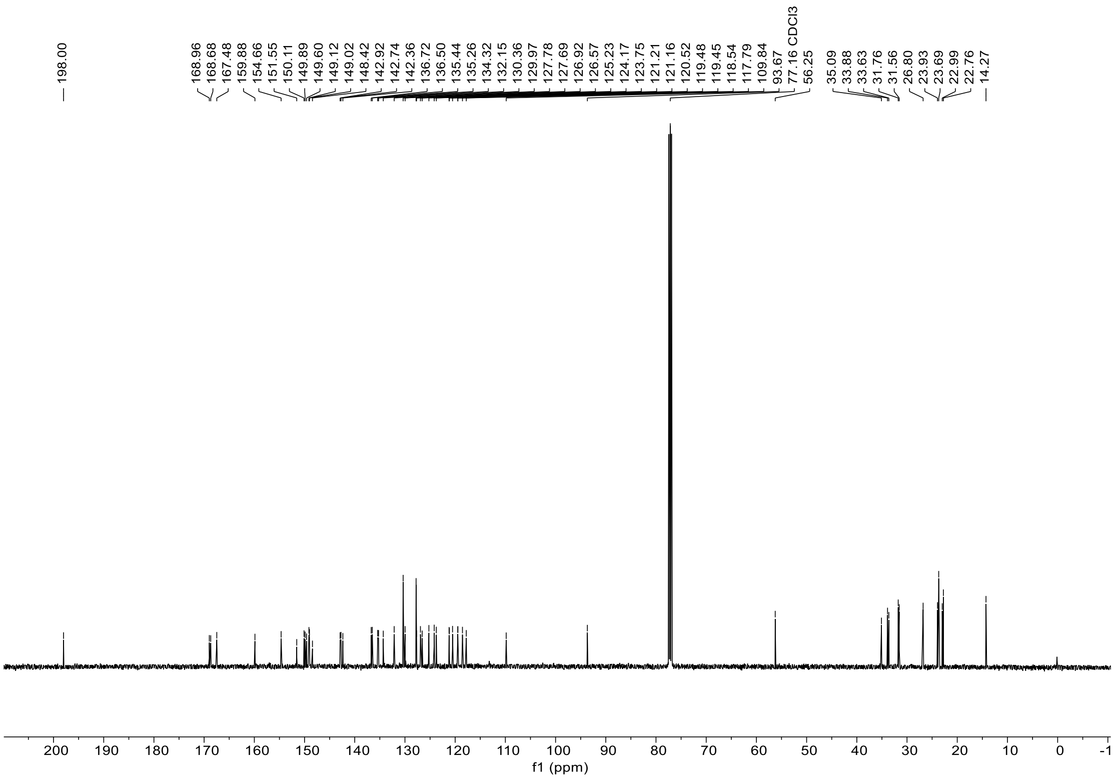

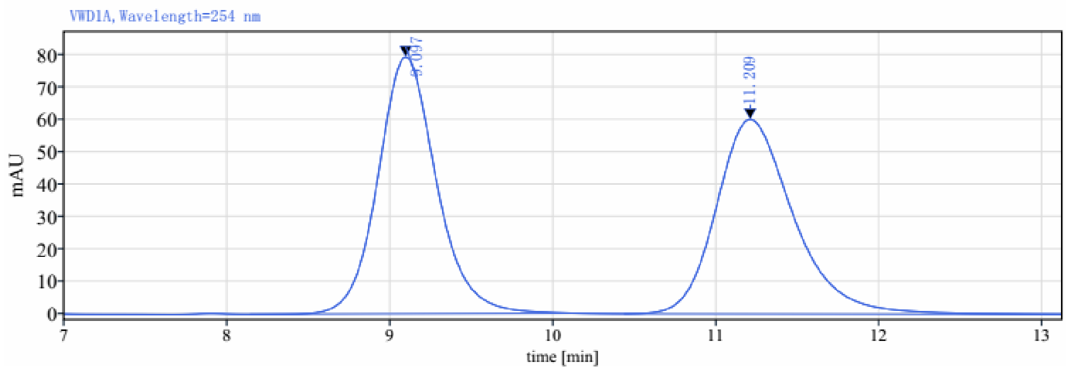

Rettime [min]	Type	Width [min]	Area	Height	Area%
9.097	MM m	1.98	1978.07	79.36	49.98
11.209	BB	3.33	1979.29	60.10	50.02

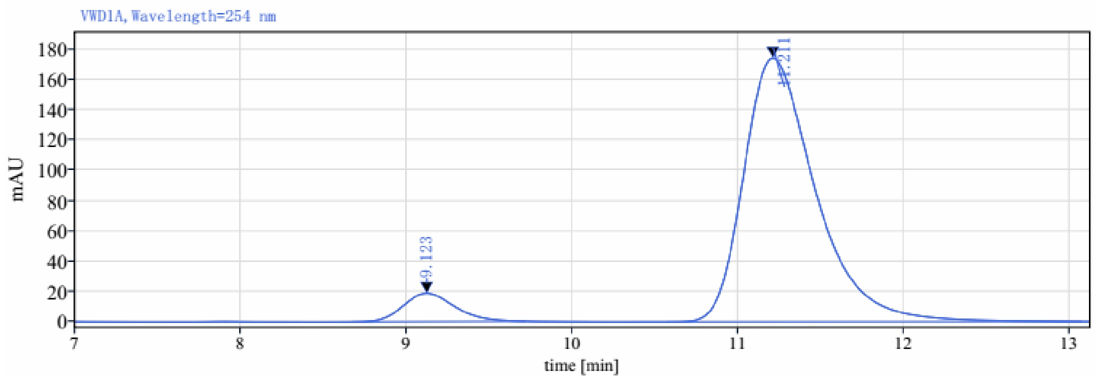

Rettime [min]	Type	Width [min]	Area	Height	Area%
9.123	MM m	1.05	406.33	18.73	7.18
11.211	BM m	2.50	5249.66	174.24	92.82

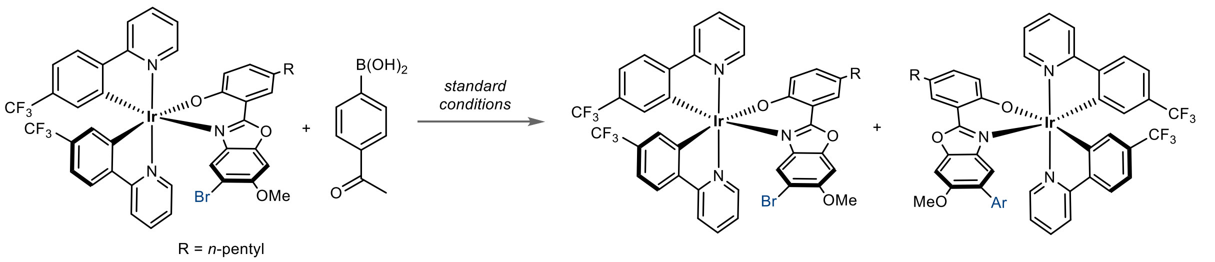

rac-**14** **2a** Λ-**14** Δ-**15**

According to the general procedure, the reaction was carried out at 30 ºC for 5 days to give the recovered Λ-**14** as yellow solid (23.1 mg, 45% yield) and Δ-**15** as yellow solid (25.6 mg, 48% yield).

Purification conditions: petroleum ether/EtOAc = 10:1 to 4:1.

R_f (Λ-**14**) = 0.6 in petroleum ether/EtOAc (4:1).

R_f (Δ-**15**) = 0.4 in petroleum ether/EtOAc (4:1).

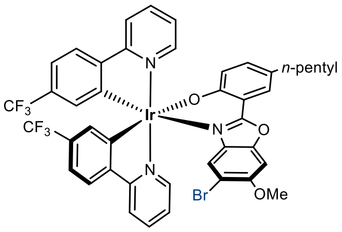

Analytical data of Λ-**14:**

^{1}H NMR (500 MHz, Chloroform-*d*) δ 8.87 (dd, *J* = 6.0, 1.5 Hz, 1H), 8.10 (dd, *J* = 6.0, 1.5 Hz, 1H), 7.90 – 7.80 (m, 2H), 7.71 – 7.63 (m, 4H), 7.58 (d, *J* = 8.0 Hz, 1H), 7.24 – 7.21 (m, 1H), 7.16 – 7.11 (m, 1H), 7.08 – 7.02 (m, 2H), 6.99 (s, 1H), 6.97 – 6.92 (m, 1H), 6.67 (d, *J* = 8.5 Hz, 1H), 6.63 (d, *J* = 2.0 Hz, 1H), 6.32 (d, *J* = 1.5 Hz, 1H), 5.91 (s, 1H), 3.82 (s, 3H), 2.49 (t, *J* = 8.0 Hz, 2H), 1.58 (p, *J* = 7.5 Hz, 2H), 1.37 – 1.29 (m, 4H), 0.89 (t, *J* = 7.0 Hz, 3H).

^{19}F NMR (471 MHz, $CDCl_3$) δ -62.54, -62.61.

^{13}C NMR (126 MHz, $CDCl_3$) δ 167.6, 167.3, 166.9, 160.3, 153.8, 150.6, 149.4, 148.8, 148.2, 148.0, 137.7, 137.5, 134.9, 130.8, 130.2, 129.8, 128.2, 127.7, 126.9, 125.5, 124.5, 124.2, 123.8, 123.3, 123.2, 122.4, 120.1, 119.4, 118.9, 118.5, 109.2, 108.1, 94.4, 56.9, 35.0, 31.7, 31.5, 22.7, 14.2.

HRMS (ESI) for $C_{43}H_{34}BrF_6IrN_3O_3$ $[M+H]^+$ calcd.1026.1312, found 1026.1301

Enantiomeric excess established by HPLC analysis using a Chiralpak IK-3 column, ee = 89% (HPLC: IK-3, 254 nm, *n*-hexane/isopropanol = 80:20, flow rate 1.0 mL/min, 40 °C, t_r (major) = 6.2 min, t_r (minor) = 7.9 min.)

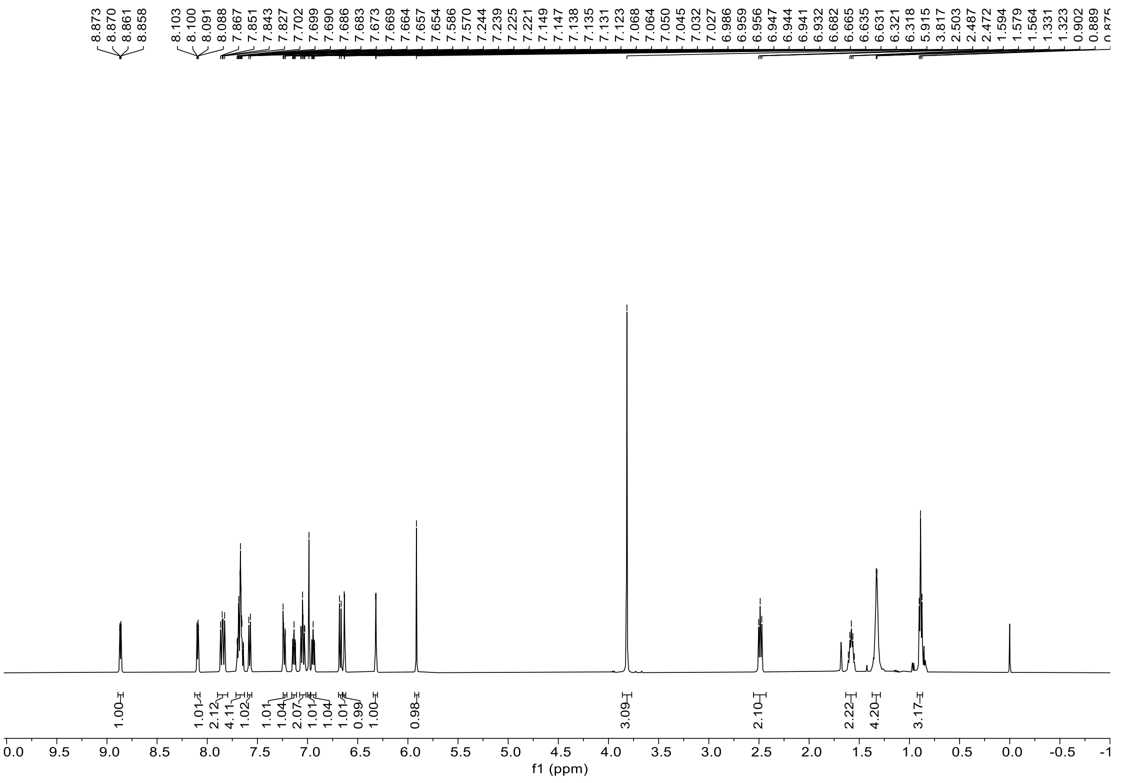

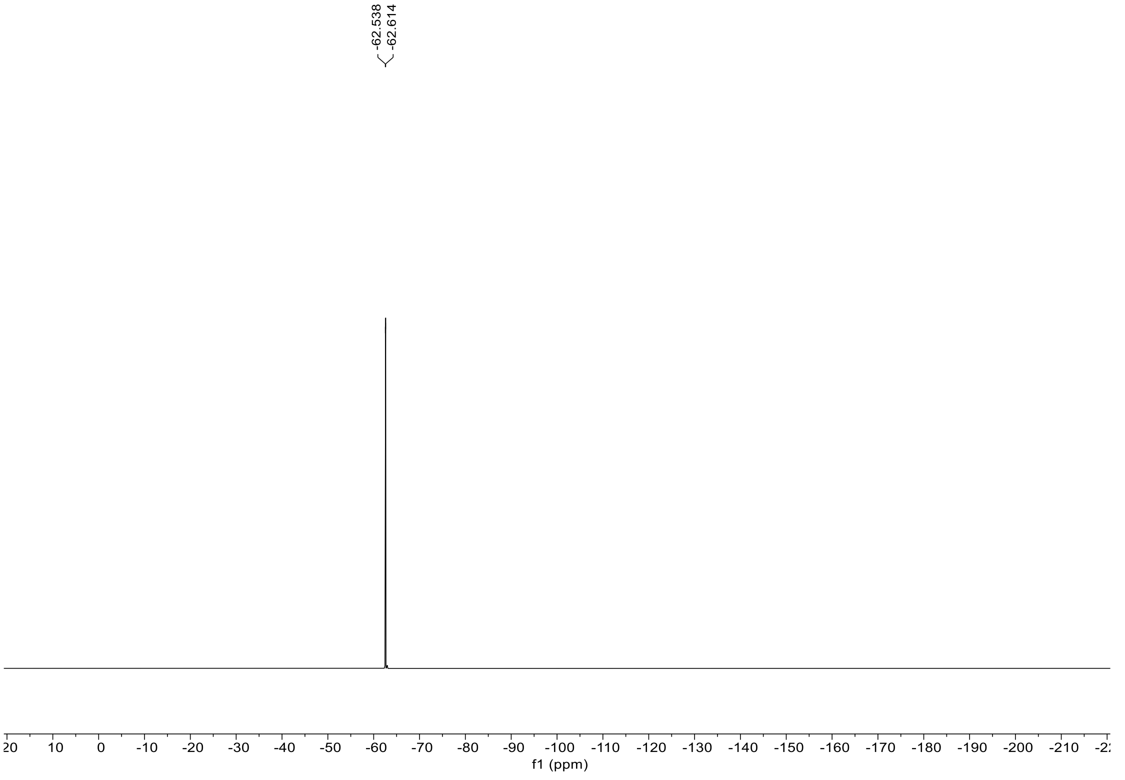

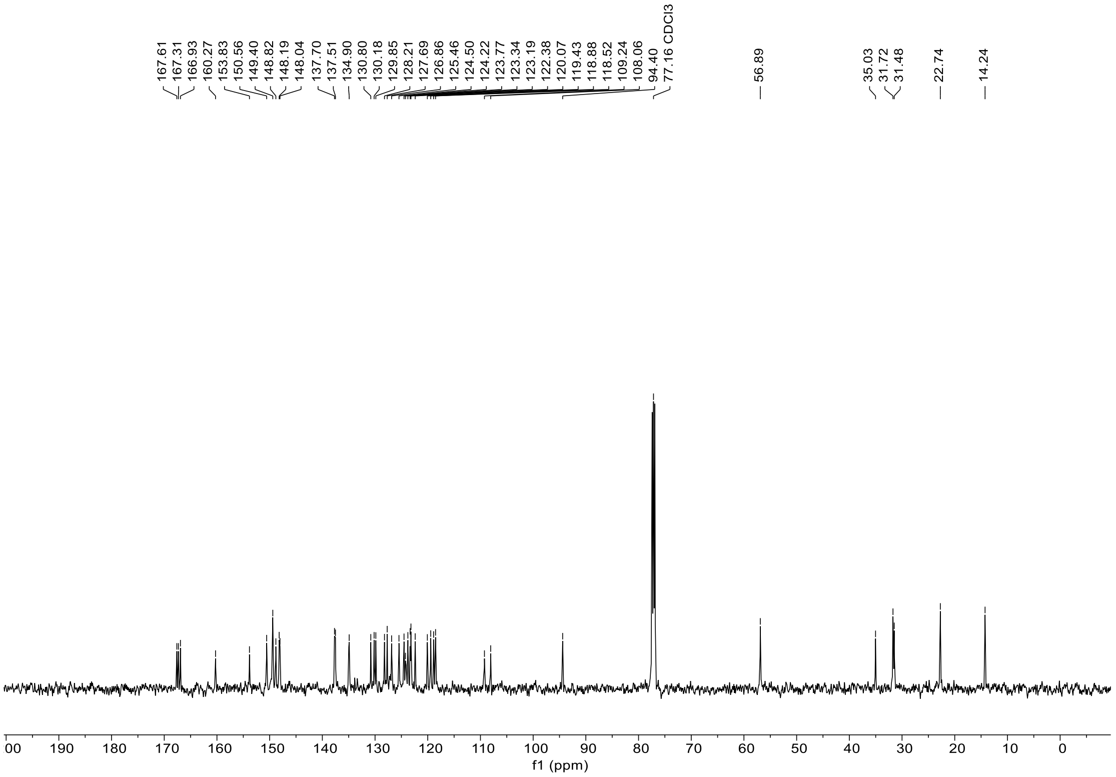

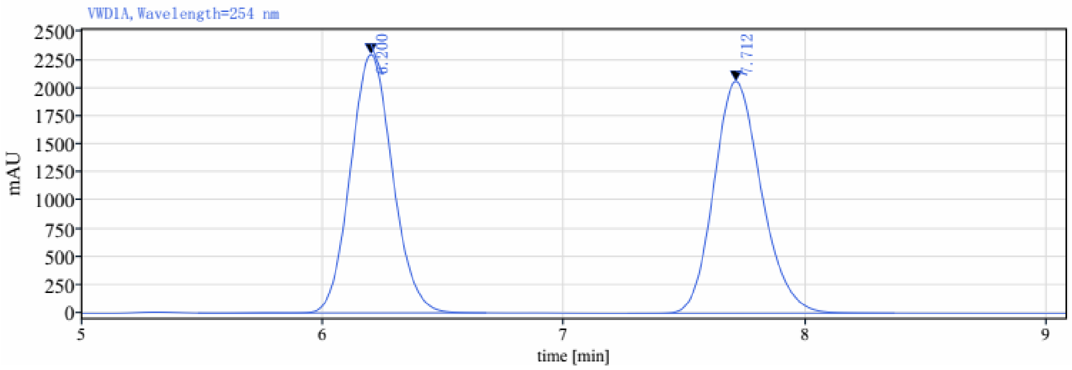

Rettime [min]	Type	Width [min]	Area	Height	Area%
6.200	MM m	1.20	27851.69	2293.97	49.78
7.712	MM m	1.11	28100.37	2057.80	50.22

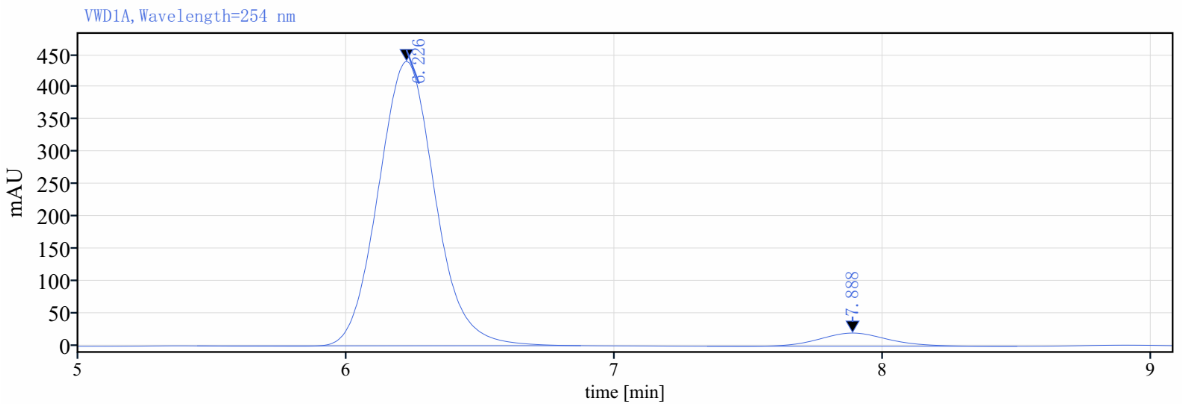

Rettime [min]	Type	Width [min]	Area	Height	Area%
6.226	MM m	1.43	6444.48	439.51	94.70
7.888	MM m	1.16	360.82	20.41	5.30

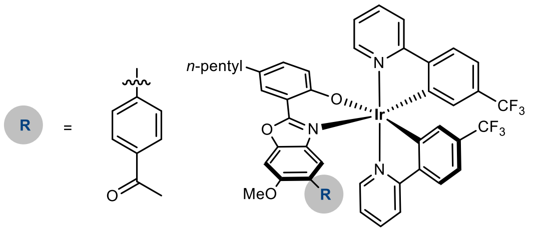

Analytical data of Δ-**15:**

^{1}H NMR (500 MHz, Chloroform-*d*) δ 8.94 (d, *J* = 5.5 Hz, 1H), 8.19 (d, *J* = 6.0 Hz, 1H), 7.92 – 7.78 (m, 4H), 7.74 – 7.64 (m, 3H), 7.63 – 7.54 (m, 2H), 7.19 – 7.14 (m, 1H), 7.14 – 7.06 (m, 4H), 7.05 – 6.97 (m, 3H), 6.69 – 6.60 (m, 2H), 6.26 (s, 1H), 6.01 – 5.95 (m, 1H), 3.79 (s, 3H), 2.62 (s, 3H), 2.51 (t, *J* = 8.0 Hz, 2H), 1.63 – 1.58 (m, 2H), 1.40 – 1.31 (m, 4H), 0.91 (t, *J* = 6.5 Hz, 3H).

^{19}F NMR (471 MHz, $CDCl_3$) δ -62.58, -62.60.

^{13}C NMR (126 MHz, $CDCl_3$) δ 198.0, 167.4, 166.9, 160.1, 155.0, 150.7, 150.1, 149.5, 149.4, 148.9, 148.6, 148.0, 142.4, 137.7, 137.5, 135.7, 134.8, 130.5, 130.2, 129.8, 129.7, 128.3, 127.9, 127.5, 127.1, 126.9, 125.1, 124.2, 124.0, 123.7, 123.2, 120.0, 119.8, 119.4, 118.9, 118.3, 109.7, 94.0, 56.3, 35.0, 31.7, 31.5, 26.7, 22.7, 14.2.

HRMS (ESI) for $C_{51}H_{41}F_6IrN_3O_4$ $[M+H]^+$ calcd.1066.2625, found 1066.2613

Enantiomeric excess established by HPLC analysis using a Chiralpak IK-3 column, ee = 90% (HPLC: IK-3, 254 nm, *n*-hexane/isopropanol = 80:20, flow rate 1.0 mL/min, 40 °C, t_r (major)

= 13.9 min, t_r (minor) = 12.0 min.)

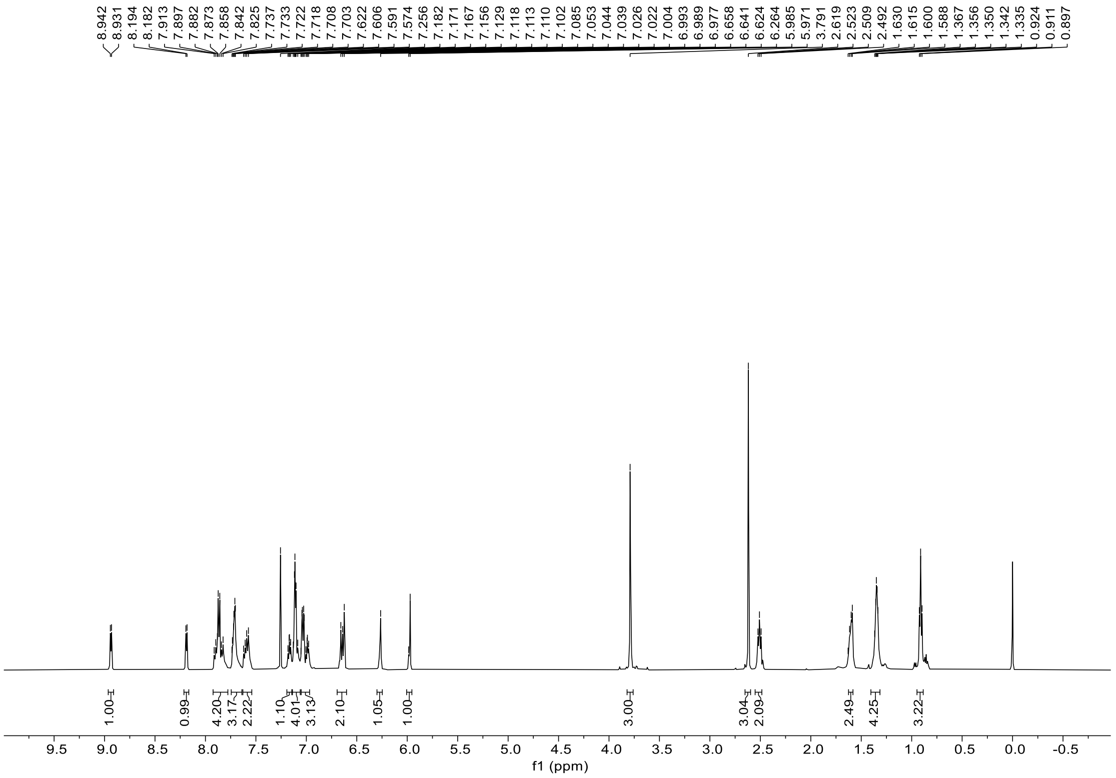

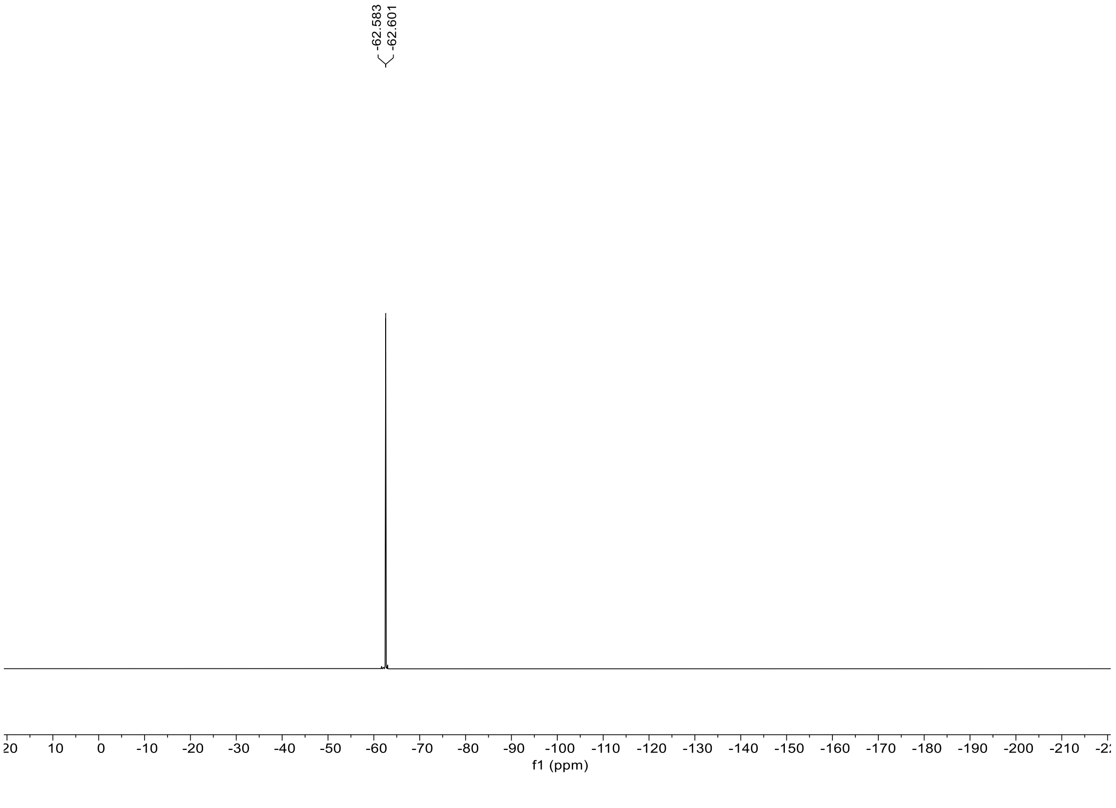

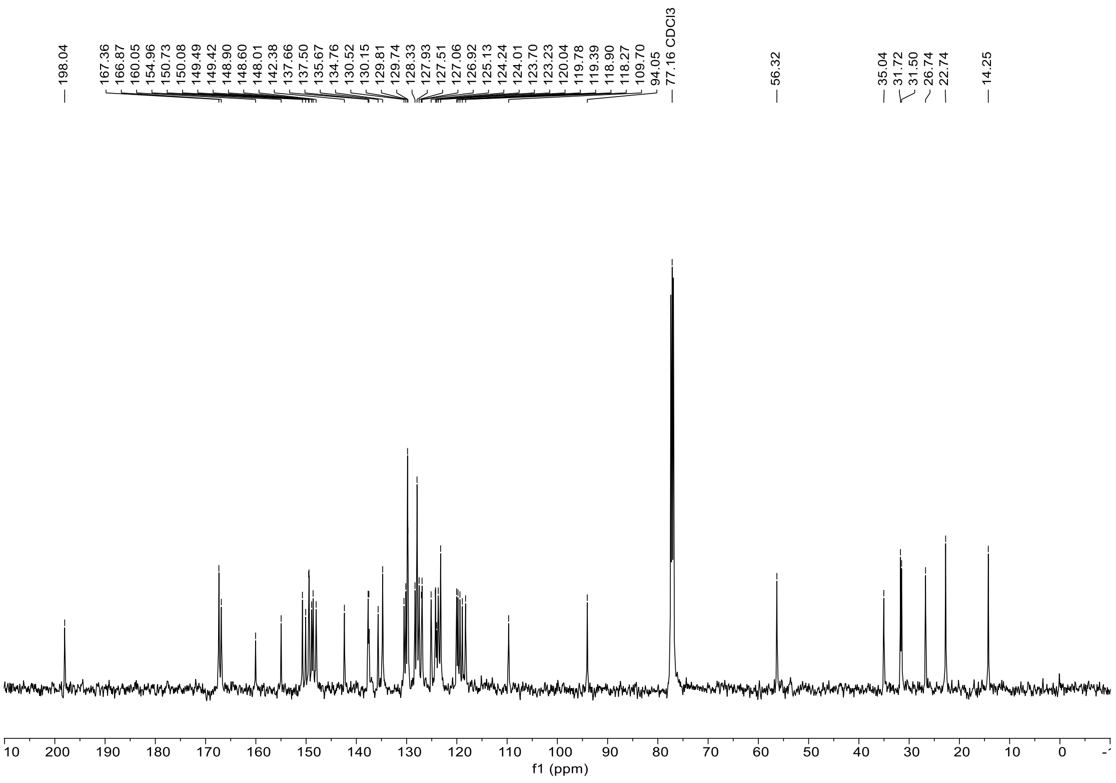

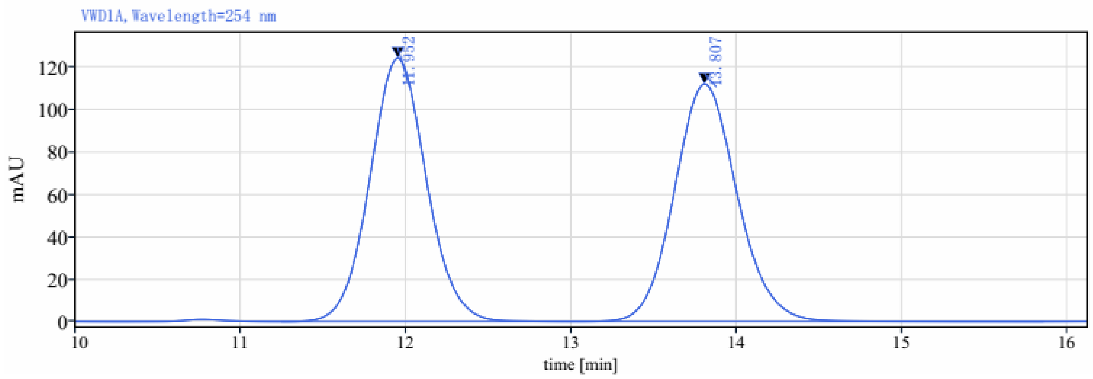

Rettime [min]	Type	Width [min]	Area	Height	Area%
11.952	BB	1.78	2953.88	123.96	49.92
13.807	BB	2.83	2963.00	111.74	50.08

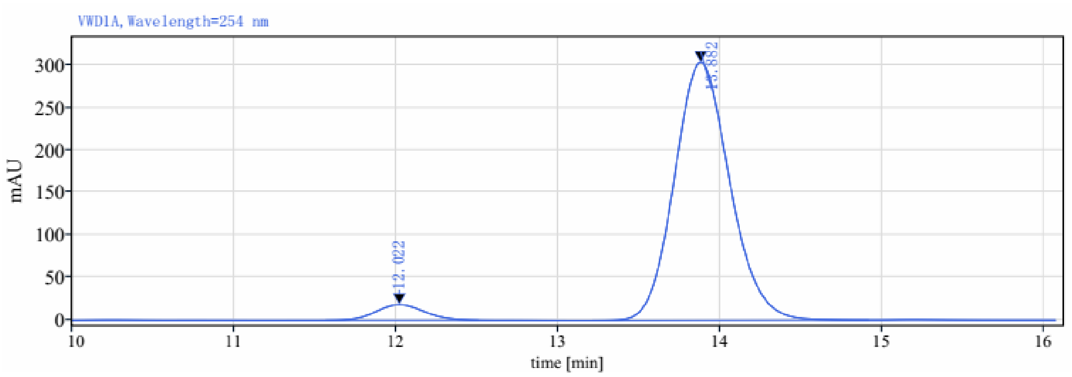

Rettime [min]	Type	Width [min]	Area	Height	Area%
12.022	MM m	0.94	379.60	18.38	4.99
13.882	BB	1.71	7234.41	304.25	95.01

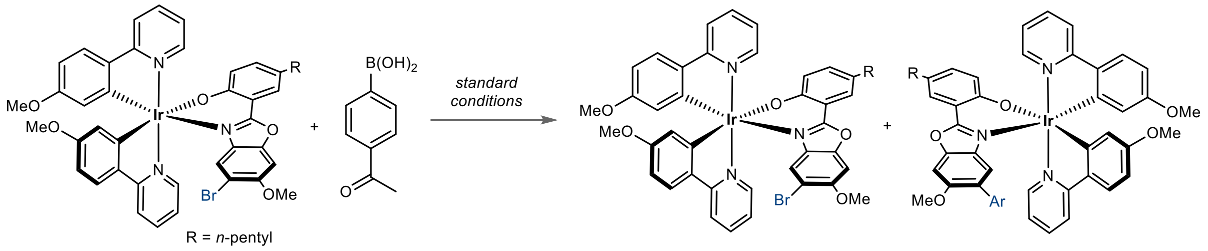

According to the general procedure, the reaction was carried out at 30 °C for 3 days to give the recovered Λ-**16** as yellow solid (17.3 mg, 37% yield) and Δ-**17** as yellow solid (23.6 mg, 48% yield).

Purification conditions: petroleum ether/EtOAc = 6:1 to 1:1.

R_f (Λ-**16**) = 0.5 in petroleum ether/EtOAc (2:1).

R_f (Δ-**17**) = 0.3 in petroleum ether/EtOAc (2:1).

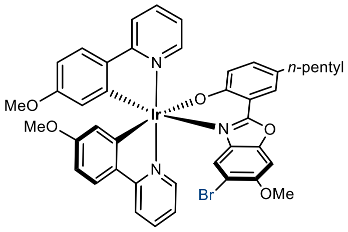

Analytical data of Λ-**16:**

^{1}H NMR (500 MHz, Chloroform-*d*) δ 8.79 (d, *J* = 5.5 Hz, 1H), 7.98 (d, *J* = 6.0 Hz, 1H), 7.68

(d, *J* = 8.5 Hz, 1H), 7.66 – 7.61 (m, 2H), 7.59 – 7.51 (m, 3H), 7.48 (dd, *J* = 8.5, 1.5 Hz, 1H), 7.01 (dd, *J* = 9.0, 2.5 Hz, 1H), 6.98 (s, 1H), 6.95 – 6.90 (m, 1H), 6.76 – 6.71 (m, 1H), 6.69 (d, *J* = 9.0 Hz, 1H), 6.61 – 6.56 (m, 1H), 6.46 – 6.40 (m, 1H), 6.16 (s, 1H), 5.98 (d, *J* = 2.5 Hz, 1H), 5.71 (d, *J* = 2.5 Hz, 1H), 3.83 (s, 3H), 3.61 (s, 3H), 3.53 (s, 3H), 2.47 (t, *J* = 8.0 Hz, 2H), 1.58 (q, *J* = 7.5 Hz, 2H), 1.38 – 1.30 (m, 4H), 0.89 (t, *J* = 6.5 Hz, 3H).

^{13}C NMR (126 MHz, $CDCl_3$) δ 168.3, 168.0, 167.8, 160.4, 160.0, 153.4, 153.3, 150.3, 149.3, 148.8, 148.7, 138.4, 137.6, 136.7, 136.4, 135.3, 134.3, 127.4, 126.7, 125.9, 125.5, 125.3, 123.0, 120.6, 120.4, 119.2, 118.2, 117.5, 116.8, 109.3, 107.6, 106.8, 106.5, 94.1, 56.8, 54.8, 54.5, 34.9, 31.6, 31.4, 22.6, 14.1.

HRMS (ESI) for $C_{43}H_{40}BrN_3O_5Ir$ $[M+H]^+$ calcd.950.1775, found 950.1743

Enantiomeric excess established by HPLC analysis using a Chiralpak IK-3 column, ee = 94% (HPLC: IK-3, 254 nm, *n*-hexane/isopropanol = 80:20, flow rate 1.0 mL/min, 40 °C, t_r (major) = 16.9 min, t_r (minor) = 26.2 min.)

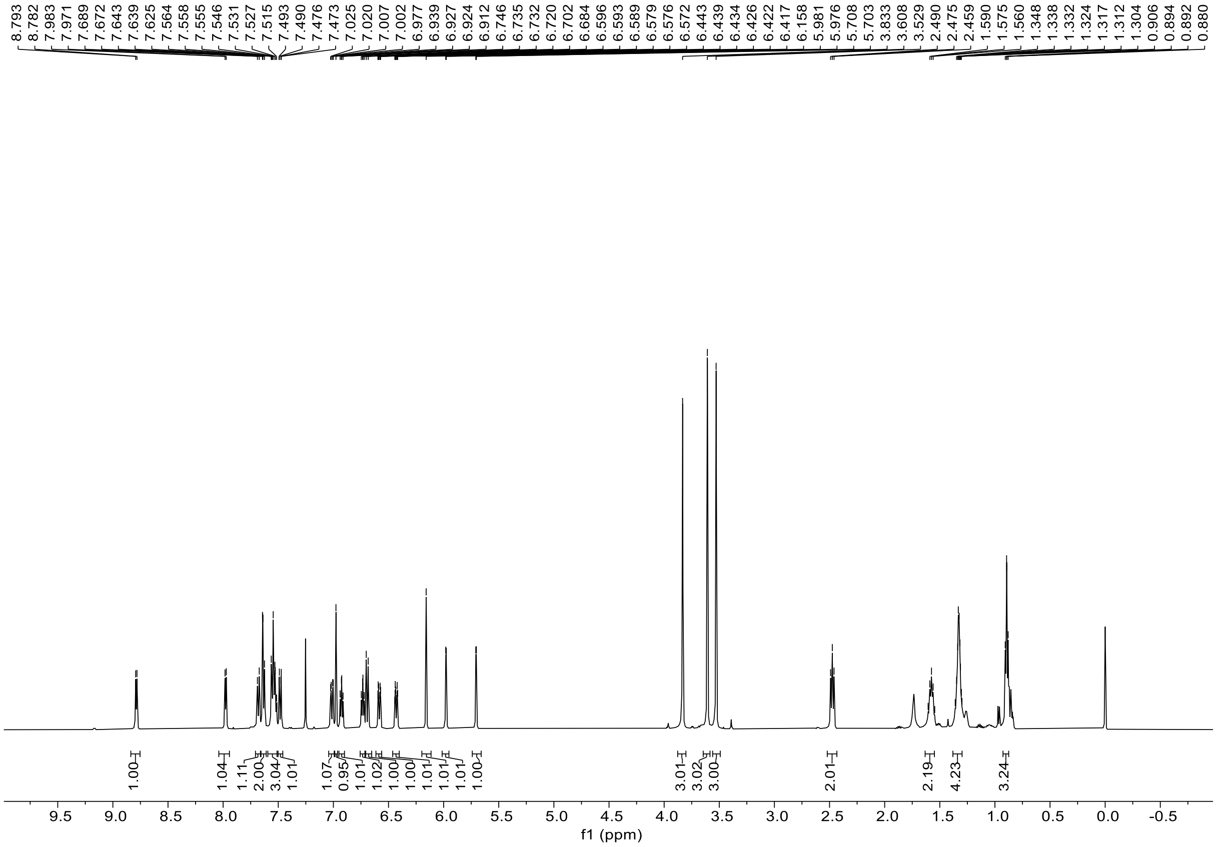

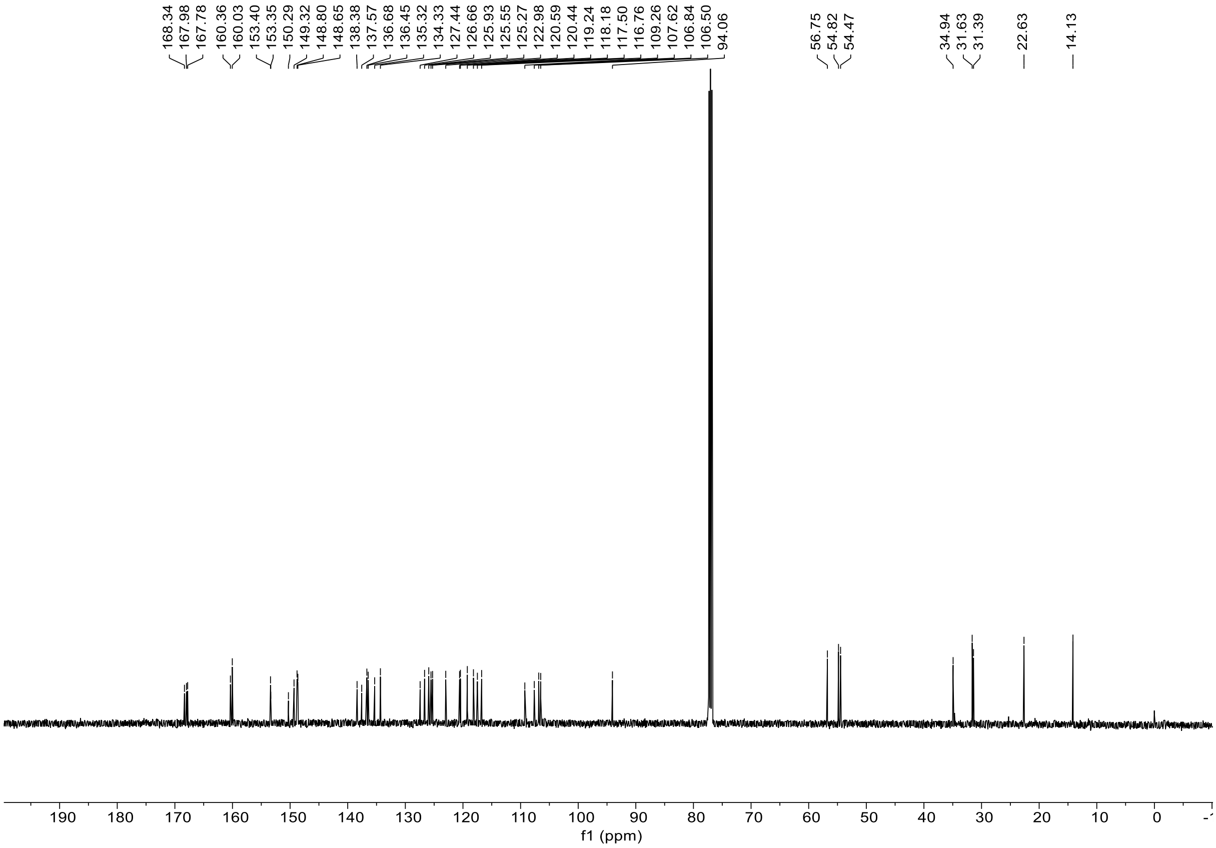

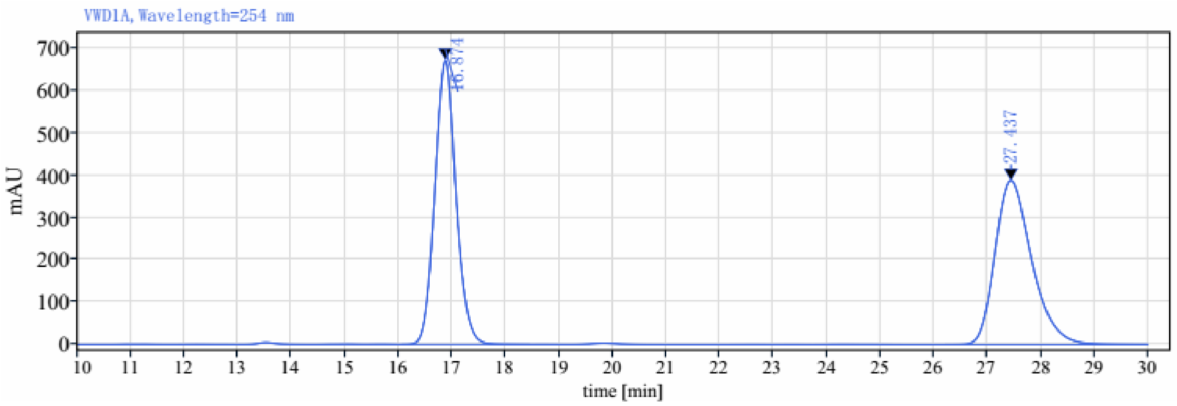

Rettime [min]	Type	Width [min]	Area	Height	Area%
16.874	BB	2.39	18434.45	671.88	49.98
27.437	BBA	3.79	18446.46	387.52	50.02

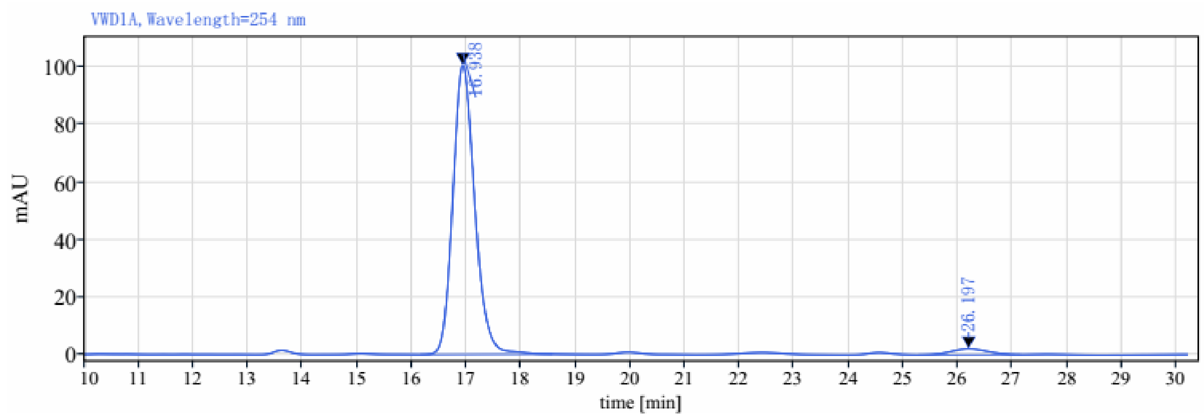

Rettime [min]	Type	Width [min]	Area	Height	Area%
16.938	BM m	2.43	2821.40	100.88	96.77
26.197	BB	1.87	94.27	2.06	3.23

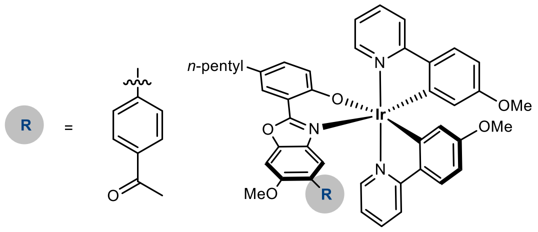

Analytical data of Δ-**17:**

^{1}H NMR (500 MHz, Chloroform-*d*) δ 8.81 (d, *J* = 6.0 Hz, 1H), 8.05 (d, *J* = 5.5 Hz, 1H), 7.88 (d, *J* = 8.0 Hz, 2H), 7.72 – 7.68 (m, 1H), 7.68 – 7.63 (m, 1H), 7.59 (d, *J* = 8.0 Hz, 1H), 7.56 – 7.48 (m, 2H), 7.47 – 7.42 (m, 2H), 7.23 (d, *J* = 8.0 Hz, 2H), 7.08 (s, 1H), 7.04 – 6.99 (m, 1H), 6.92 (t, *J* = 6.5 Hz, 1H), 6.76 – 6.68 (m, 2H), 6.43 – 6.33 (m, 3H), 6.00 – 5.93 (m, 1H), 5.66 (s, 1H), 3.78 (s, 3H), 3.50 (s, 3H), 3.43 (s, 3H), 2.61 (s, 3H), 2.50 (t, *J* = 8.0 Hz, 2H), 1.60 (p, *J* = 7.5 Hz, 2H), 1.41 – 1.30 (m, 4H), 0.90 (t, *J* = 6.5 Hz, 3H).

^{13}C NMR (126 MHz, $CDCl_3$) δ 197.8, 168.4, 168.0, 167.6, 160.1, 160.0, 159.8, 154.6, 153.5, 150.8, 150.0, 148.9, 148.8, 142.7, 138.3, 137.5, 136.6, 136.4, 135.3, 135.2, 134.2, 130.0, 127.7, 127.5, 126.7, 126.4, 125.8, 125.4, 125.3, 120.6, 120.5, 120.4, 119.0, 118.2, 117.4, 116.7, 109.7, 106.8, 106.2, 93.7, 56.2, 54.5, 54.4, 35.0, 31.6, 31.4, 26.7, 22.6, 14.2.

HRMS (ESI) for $C_{51}H_{47}N_3O_6Ir$ $[M+H]^+$ calcd.990.3089, found 990.3101

Enantiomeric excess established by HPLC analysis using a Chiralpak IK-3 column, ee = 72%

(HPLC: IK-3, 254 nm, *n*-hexane/isopropanol = 60:40, flow rate 1.0 mL/min, 40 ^{o}C, t_r (major) = 18.4 min, t_r (minor) = 13.8 min.)

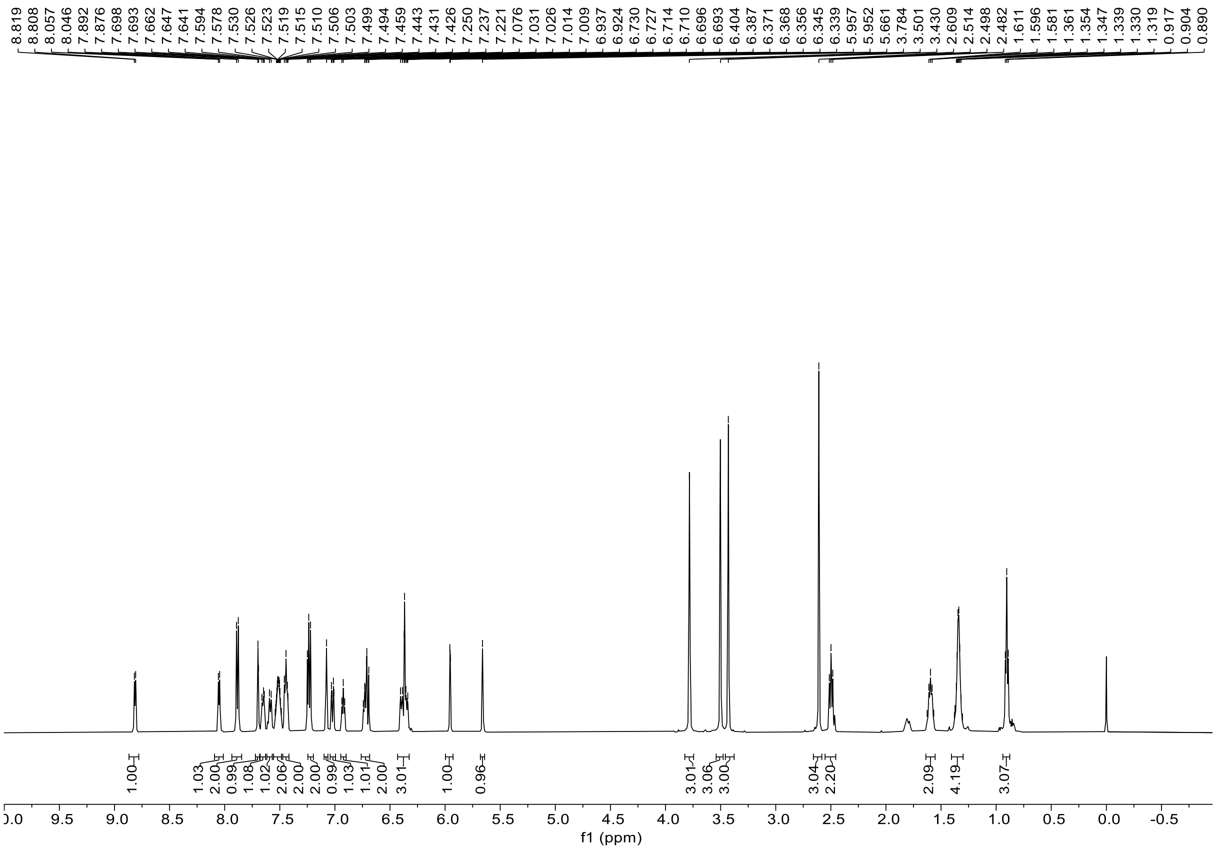

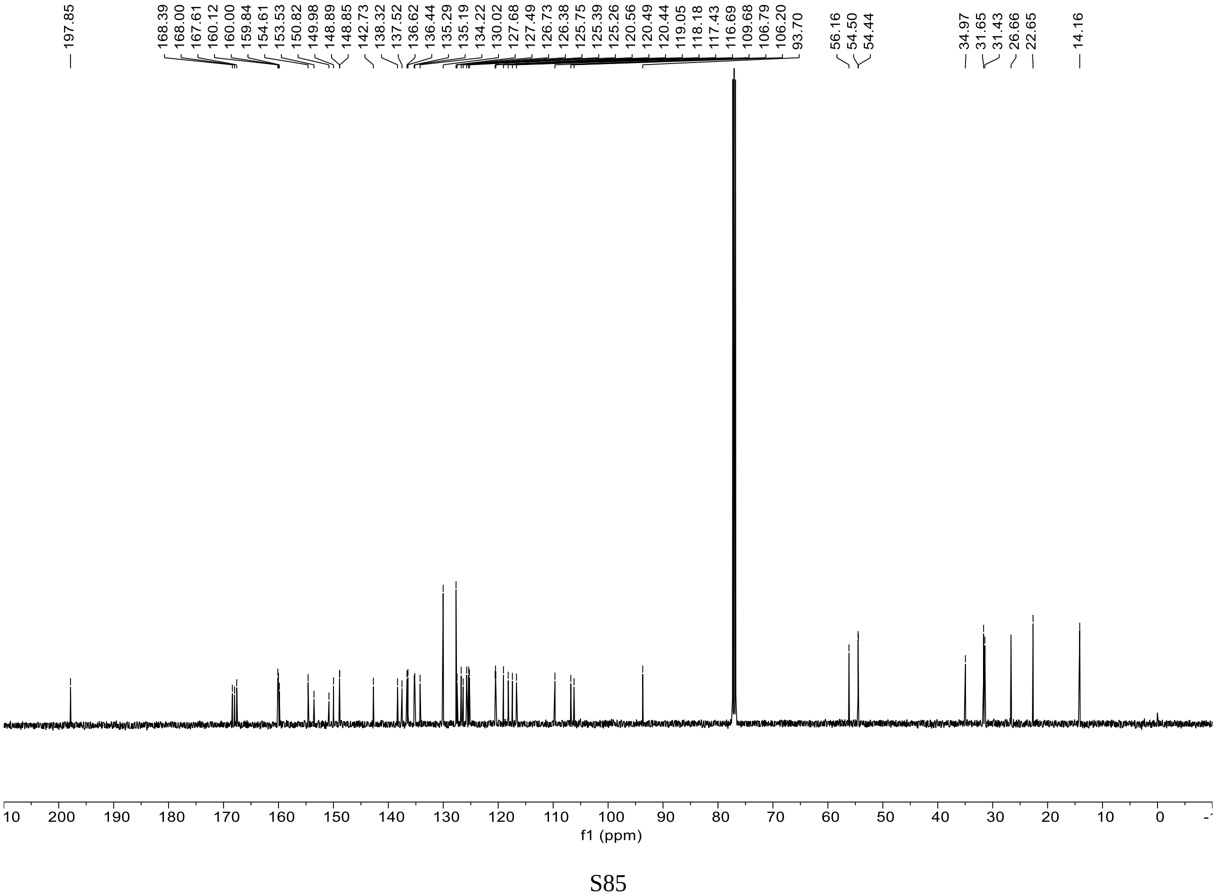

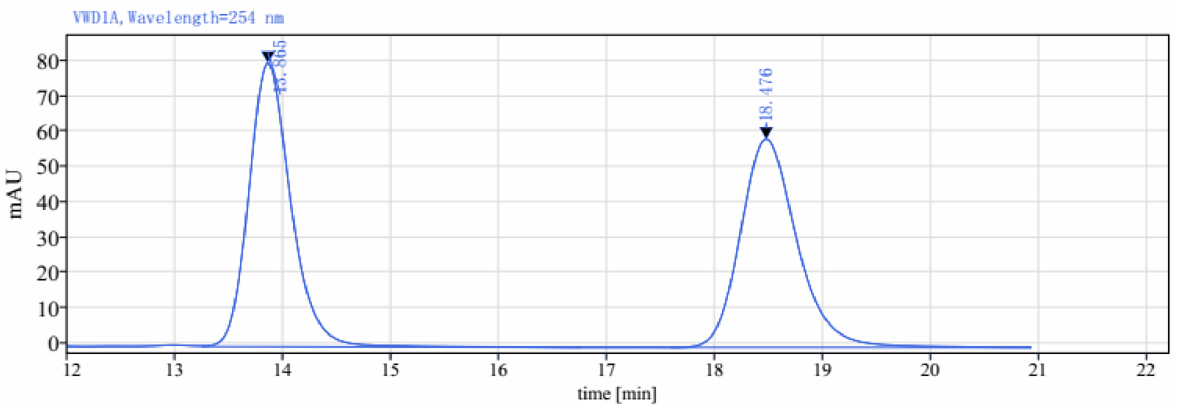

Rettime [min]	Type	Width [min]	Area	Height	Area%
13.865	VB	3.51	2170.28	80.19	50.26
18.476	BBA	3.34	2147.55	58.94	49.74

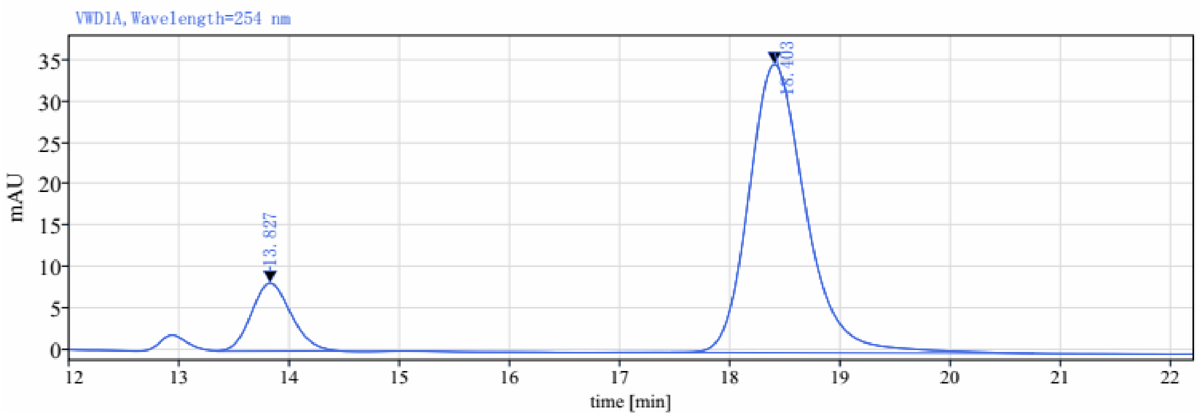

Rettime [min]	Type	Width [min]	Area	Height	Area%
13.827	VM m	1.11	206.28	8.23	14.14
18.403	BM m	3.29	1252.60	34.94	85.86

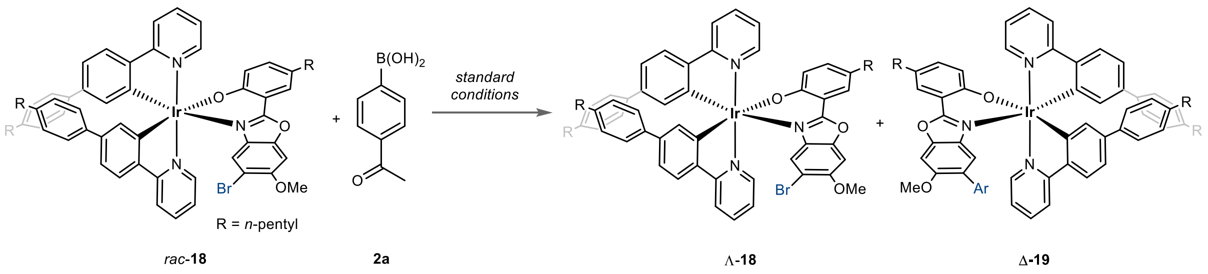

According to the general procedure, the reaction was carried out at 30 ºC for 4.5 days to give

the recovered Λ-**18** as dark red solid (23.6 mg, 40% yield) and Δ-**19** as dark red solid (26.1 mg, 43% yield).

Purification conditions: petroleum ether/DCM/EtOAc = 10:1:1 to 4:1:1.

R_f (Λ-**18**) = 0.4 in petroleum ether/ DCM/EtOAc (4:1:1).

R_f (Δ-**19**) = 0.2 in petroleum ether/EtOAc (4:1:1).

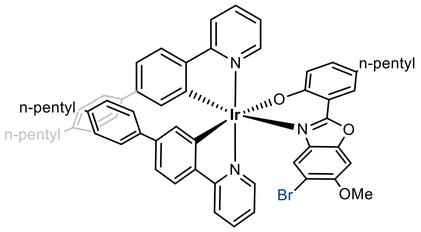

Analytical data of Λ-**18:**

^{1}H NMR (500 MHz, Chloroform-*d*) δ 8.88 (d, *J* = 5.5 Hz, 1H), 8.07 (d, *J* = 6.0 Hz, 1H), 7.80 (d, *J* = 8.0 Hz, 1H), 7.73 (d, *J* = 8.5 Hz, 1H), 7.67 (d, *J* = 2.5 Hz, 1H), 7.61 – 7.52 (m, 4H), 7.27 (d, *J* = 8.0 Hz, 2H), 7.22 (d, *J* = 8.0 Hz, 2H), 7.16 (dd, *J* = 8.0, 2.0 Hz, 1H), 7.10 – 6.98 (m, 7H), 6.95 (s, 1H), 6.79 (t, *J* = 6.5 Hz, 1H), 6.72 (d, *J* = 9.0 Hz, 1H), 6.67 (s, 1H), 6.42 (d, *J* = 2.0 Hz, 1H), 6.17 (s, 1H), 3.78 (s, 3H), 2.58 – 2.46 (m, 6H), 1.65 – 1.53 (m, 6H), 1.35 – 1.27 (m, 12H), 0.91 – 0.84 (m, 9H).

^{13}C NMR (126 MHz, $CDCl_3$) δ 168.6, 168.2, 168.0, 160.2, 153.5, 151.4, 149.4, 149.1, 149.0, 148.6, 144.2, 143.7, 142.1, 141.8, 141.6, 141.2, 139.4, 139.2, 136.9, 136.7, 135.4, 134.5, 132.4, 130.0, 128.5, 127.5, 127.4, 127.0, 126.8, 125.8, 124.8, 124.2, 123.2, 121.8, 121.5, 120.8, 120.5, 119.0, 118.4, 109.4, 107.8, 94.2, 56.8, 35.71, 35.68, 35.1, 31.76, 31.75, 31.7, 31.5, 31.3, 31.2, 22.8, 22.7, 14.3, 14.17, 14.16.

HRMS (ESI) for $C_{63}H_{64}IrBrN_3O_3$ $[M+H]^+$ calcd.1182.3755, found 1182.3729

Enantiomeric excess established by HPLC analysis using a Chiralpak IM column, ee = 93% (HPLC: IM, 254 nm, *n*-hexane/isopropanol = 80:20, flow rate 1.0 mL/min, 40 ^{o}C, t_r (major) = 7.1 min, t_r (minor) = 8.6 min.)

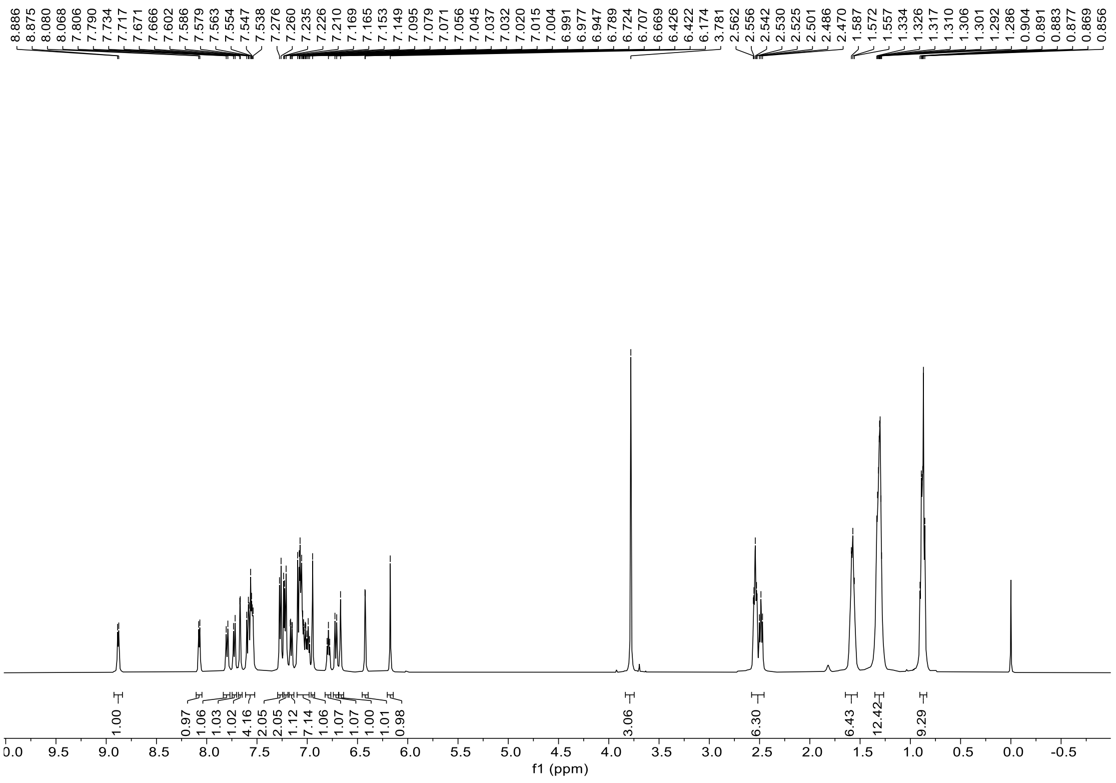

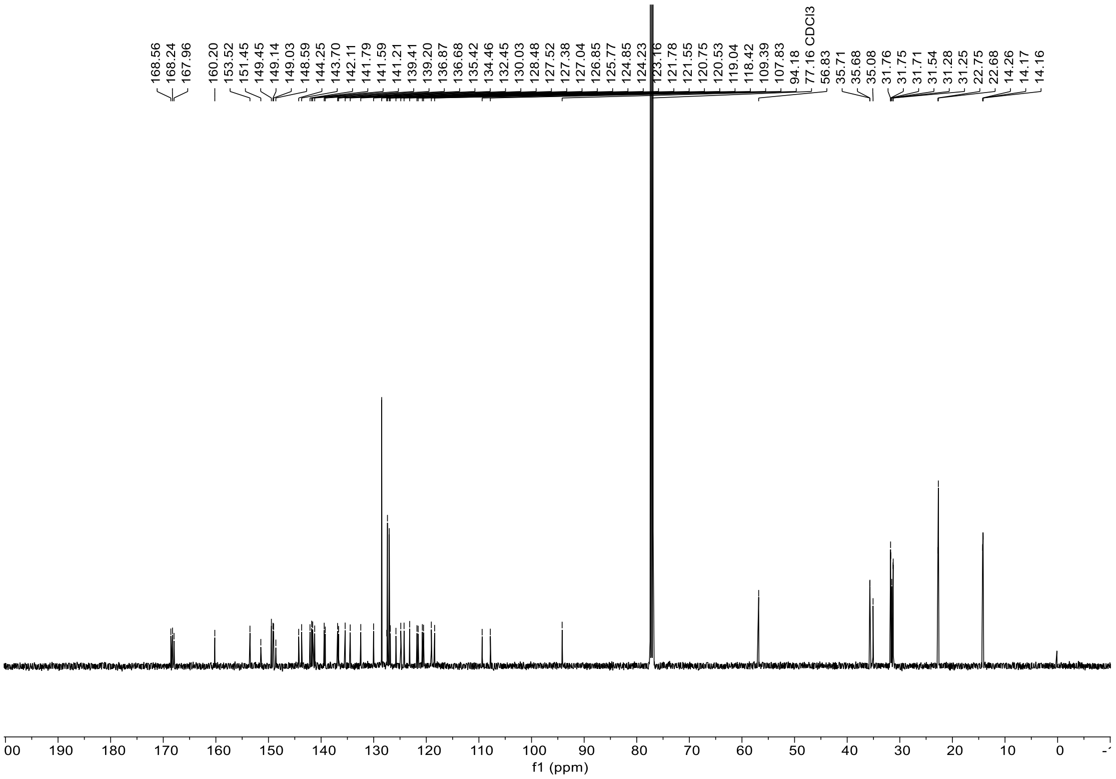

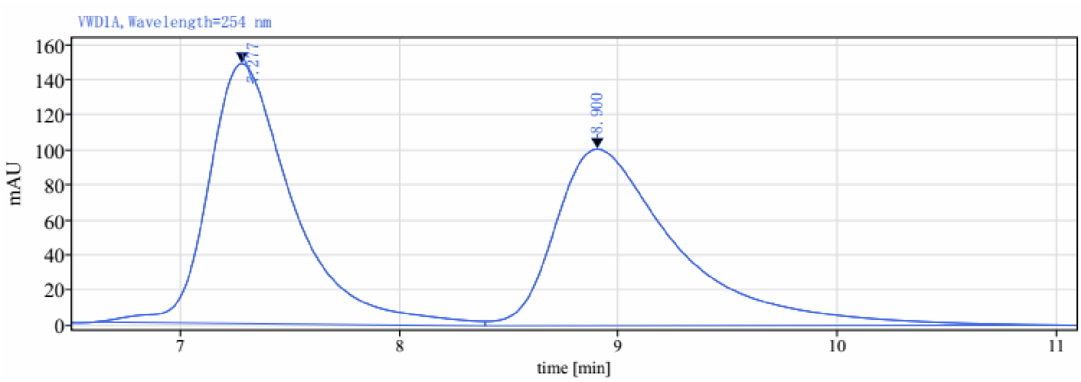

Rettime [min]	Type	Width [min]	Area	Height	Area%
7.277	MM m	1.78	3977.09	148.35	50.56
8.900	VM m	2.62	3888.97	100.84	49.44

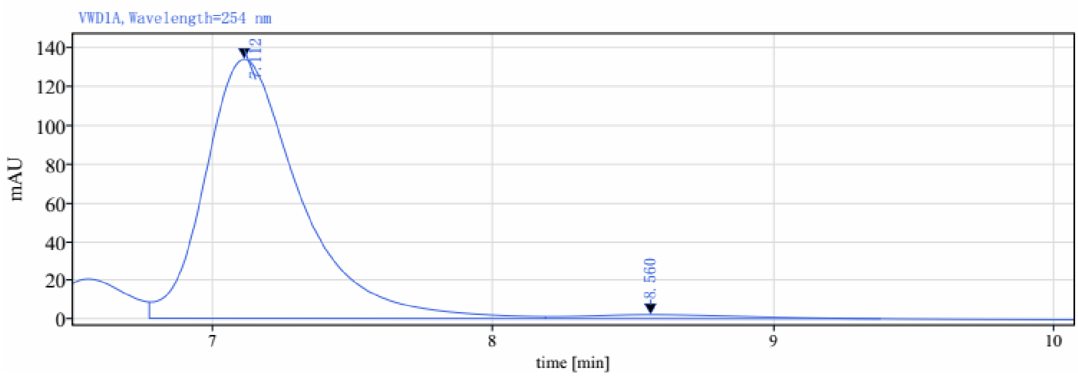

Rettime [min]	Type	Width [min]	Area	Height	Area%
7.112	MM m	1.41	3164.20	133.86	97.23
8.560	MM m	1.19	90.03	2.19	2.77

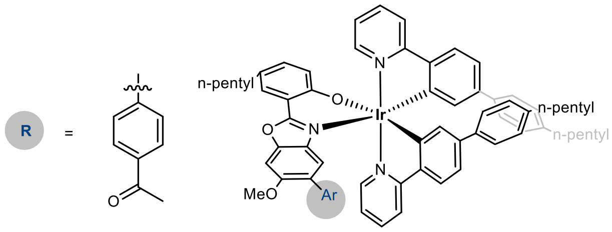

Analytical data of Δ-**19:**

^{1}H NMR (500 MHz, Chloroform-*d*) δ 8.91 (d, *J* = 5.5 Hz, 1H), 8.23 – 8.15 (m, 1H), 7.80 (d, *J* = 8.0 Hz, 1H), 7.75 – 7.68 (m, 2H), 7.63 – 7.56 (m, 4H), 7.53 – 7.47 (m, 2H), 7.19 (d, *J* = 7.9 Hz, 2H), 7.13 – 7.10 (m, 2H), 7.09 – 6.97 (m, 10H), 6.94 (dd, *J* = 8.0, 2.0 Hz, 1H), 6.86 (t, *J* =

6.5 Hz, 1H), 6.74 (d, *J* = 8.5 Hz, 1H), 6.70 – 6.68 (m, 1H), 6.38 (d, *J* = 1.5 Hz, 1H), 6.33 (s, 1H), 3.80 – 3.74 (m, 3H), 2.56 – 2.49 (m, 6H), 2.44 (s, 3H), 1.63 – 1.52 (m, 6H), 1.36 – 1.27 (m, 12H), 0.92 – 0.85 (m, 9H).

^{13}C NMR (126 MHz, $CDCl_3$) δ 197.9, 168.7, 168.2, 167.4, 160.0, 154.7, 151.3, 150.1, 149.3, 148.9, 144.3, 143.6, 142.7, 141.9, 141.6, 141.4, 141.3, 139.2, 138.7, 136.8, 136.7, 135.24, 135.21, 134.4, 131.8, 130.0, 128.5, 128.4, 127.8, 127.7, 127.04, 126.98, 126.9, 126.8, 125.4, 124.7, 124.2, 121.8, 121.6, 120.8, 120.6, 120.4, 119.1, 118.4, 109.9, 93.8, 56.3, 35.7, 35.6, 35.1, 31.8, 31.7, 31.6, 31.31, 31.26, 26.6, 22.8, 22.68, 22.66, 14.3, 14.17, 14.15.

HRMS (ESI) for $C_{71}H_{71}IrN_3O_4$ $[M+H]^+$ calcd.1222.5069, found 1222.5075

Enantiomeric excess established by HPLC analysis using a Chiralpak IM column, ee = 85% (HPLC: IM, 254 nm, *n*-hexane/isopropanol = 80:20, flow rate 1.0 mL/min, 40 °C, t_r (major) = 15.9 min, t_r (minor) = 12.9 min.)

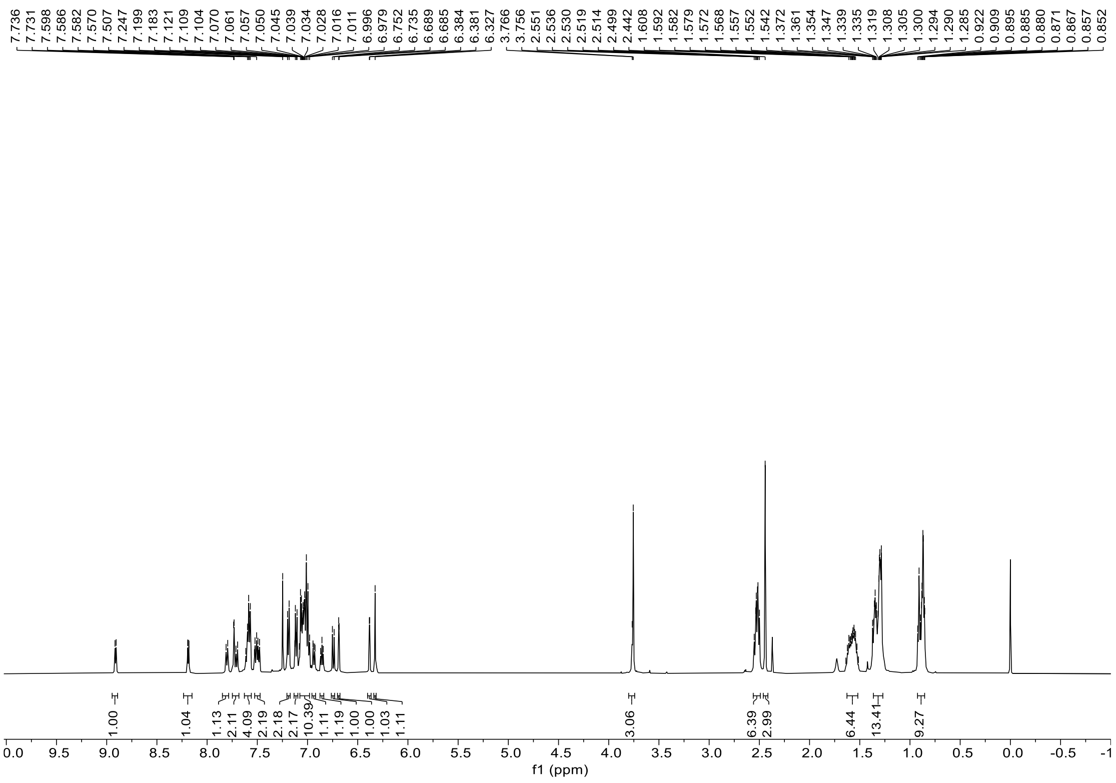

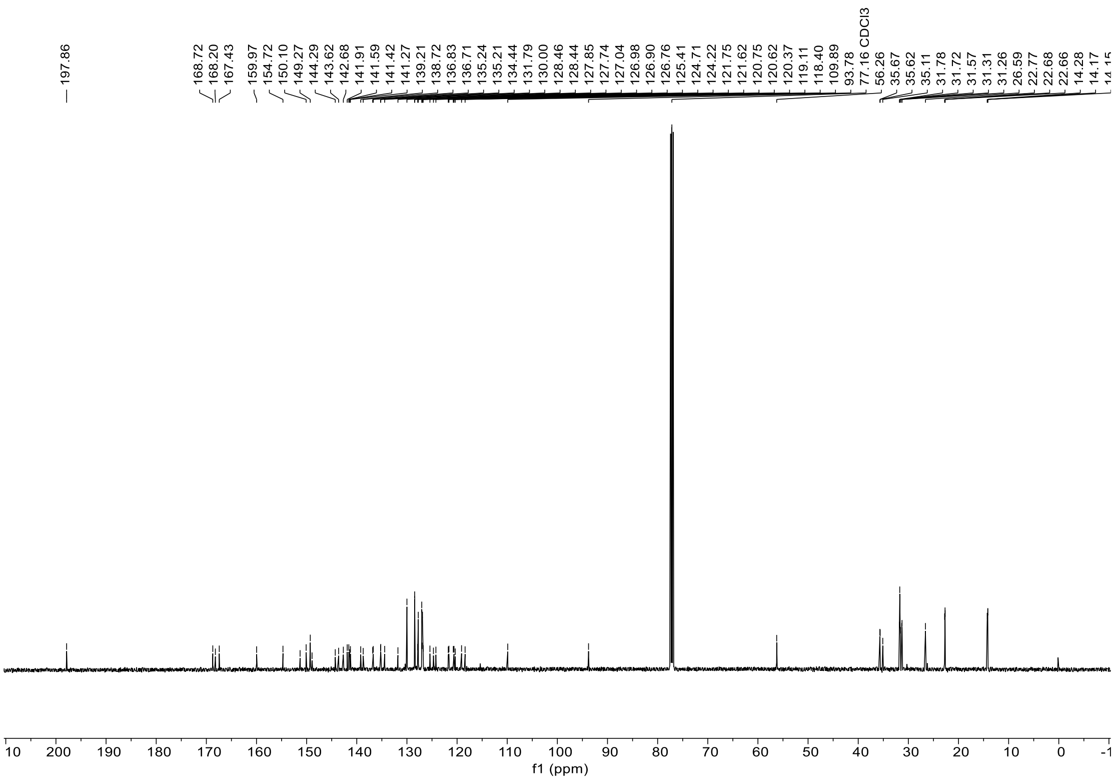

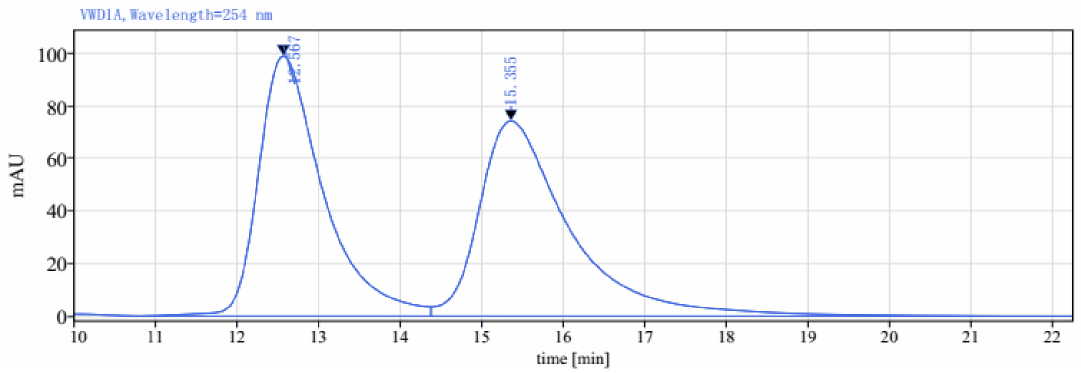

Rettime [min]	Type	Width [min]	Area	Height	Area%
12.567	BV	3.53	5443.59	98.97	48.84
15.355	VB	6.97	5701.62	74.28	51.16

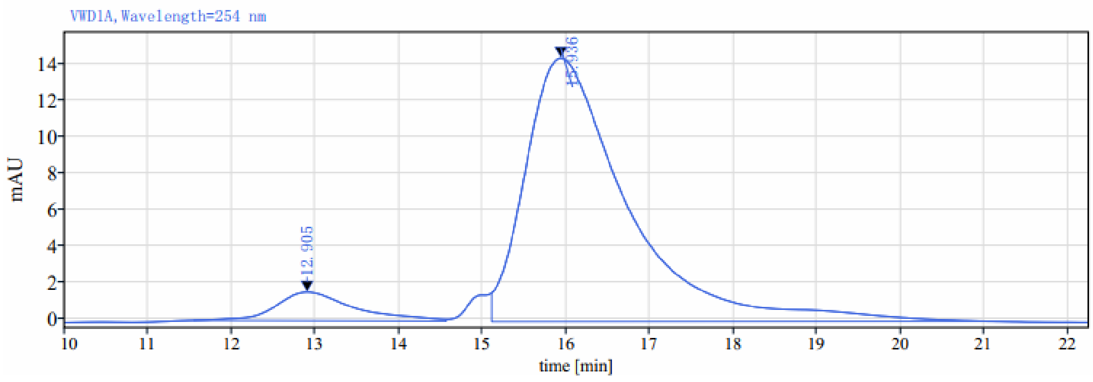

Rettime [min]	Type	Width [min]	Area	Height	Area%
12.905	MM m	3.02	100.52	1.55	7.60
15.936	MM m	5.95	1222.09	14.47	92.40

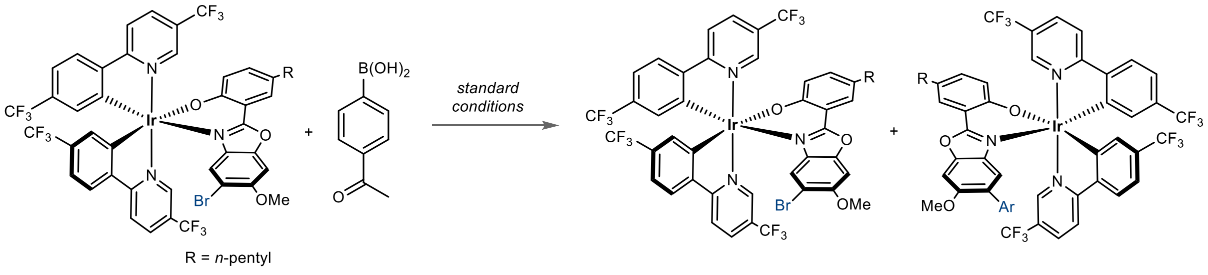

According to the general procedure, the reaction was carried out at 30 °C for 4.5 days to give the recovered Λ-**20** as red solid (22.6 mg, 39% yield) and Δ-**21** as red solid (27.0 mg, 45% yield).

Purification conditions: petroleum ether/EtOAc = 10:1 to 5:1.

R_f (Λ-**20**) = 0.5 in petroleum ether/EtOAc (5:1).

R_f (Δ-**21**) = 0.3 in petroleum ether/EtOAc (5:1).

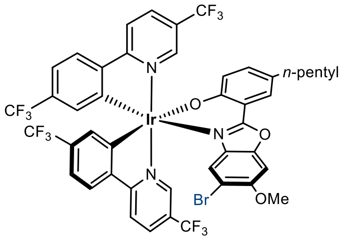

Analytical data of Λ-**20:**

^{1}H NMR (500 MHz, $CDCl_3$) δ 9.23 (s, 1H), 8.33 (s, 1H), 8.03 (d, J = 8.5 Hz, 2H), 7.97 – 7.91

(m, 2H), 7.82 (d, J = 8.0 Hz, 1H), 7.70 (d, J = 8.0 Hz, 1H), 7.61 (d, J = 2.5 Hz, 1H), 7.31 (dd, J = 8.5, 2.0 Hz, 1H), 7.15 (dd, J = 8.5, 2.0 Hz, 1H), 7.08 – 7.01 (m, 2H), 6.70 (d, J = 2.0 Hz, 1H), 6.62 (d, J = 9.0 Hz, 1H), 6.29 (d, J = 2.0 Hz, 1H), 5.98 (s, 1H), 3.87 (s, 3H), 2.48 (t, J = 7.5 Hz, 2H), 1.60 – 1.53 (m, 2H), 1.36 – 1.28 (m, 4H), 0.89 (t, J = 7.0 Hz, 3H).

^{19}F NMR (471 MHz, $CDCl_3$) δ -62.44, -62.62, -62.96, -62.99.

^{13}C NMR (126 MHz, $CDCl_3$) δ 170.9, 170.7, 167.7, 161.0, 154.2, 151.6, 149.9, 149.7, 147.0, 146.7, 146.2, 135.6, 135.3, 134.9, 134.2, 132.1, 131.6, 130.4, 129.5, 127.7, 127.0, 126.2, 126.0, 125.9, 125.4, 124.8, 123.8, 122.4, 121.7, 119.8, 119.5, 119.3, 119.1, 110.0, 108.6, 94.8, 56.9, 34.9, 31.5, 31.4, 22.7, 14.2.

HRMS (ESI) for $C_{45}H_{32}IrBrF_{12}N_3O_3$ $[M+H]^+$ calcd.1162.1059, found 1162.1048

Enantiomeric excess established by HPLC analysis using a Chiralpak IA-3 column, ee = 99% (HPLC: IA-3, 254 nm, *n*-hexane/isopropanol = 90:10, flow rate 1.0 mL/min, 40 °C, t_r (major) = 9.9 min, t_r (minor) = 9.0 min.)

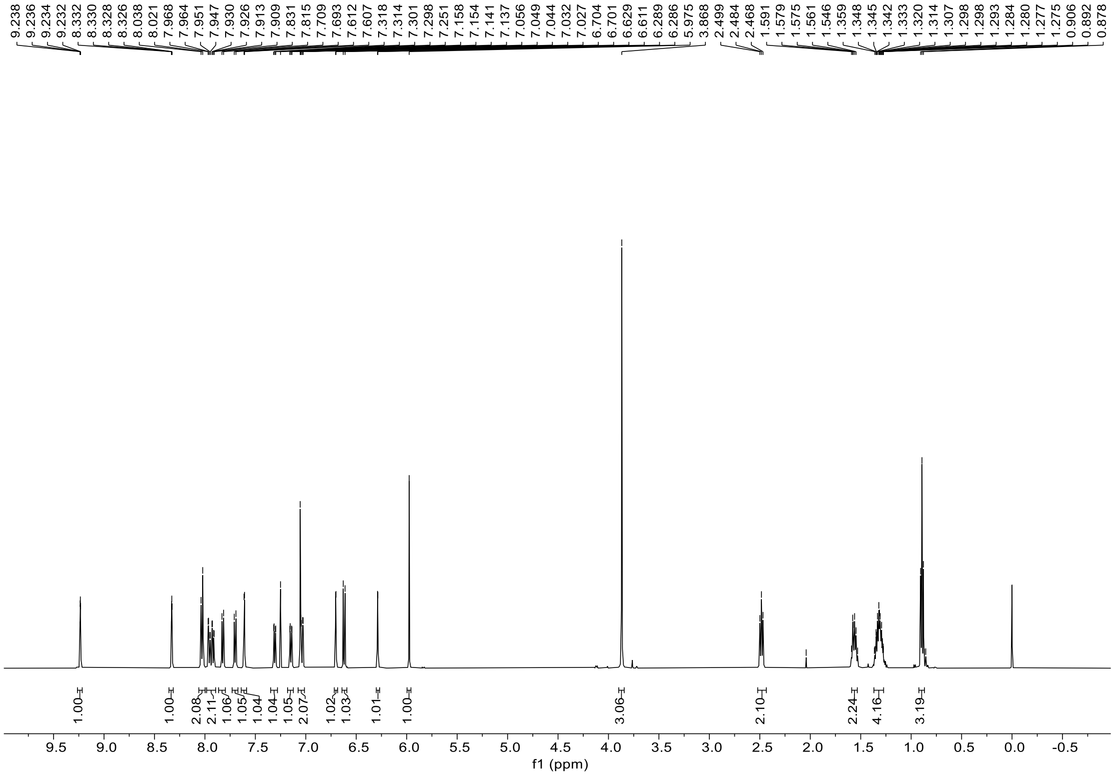

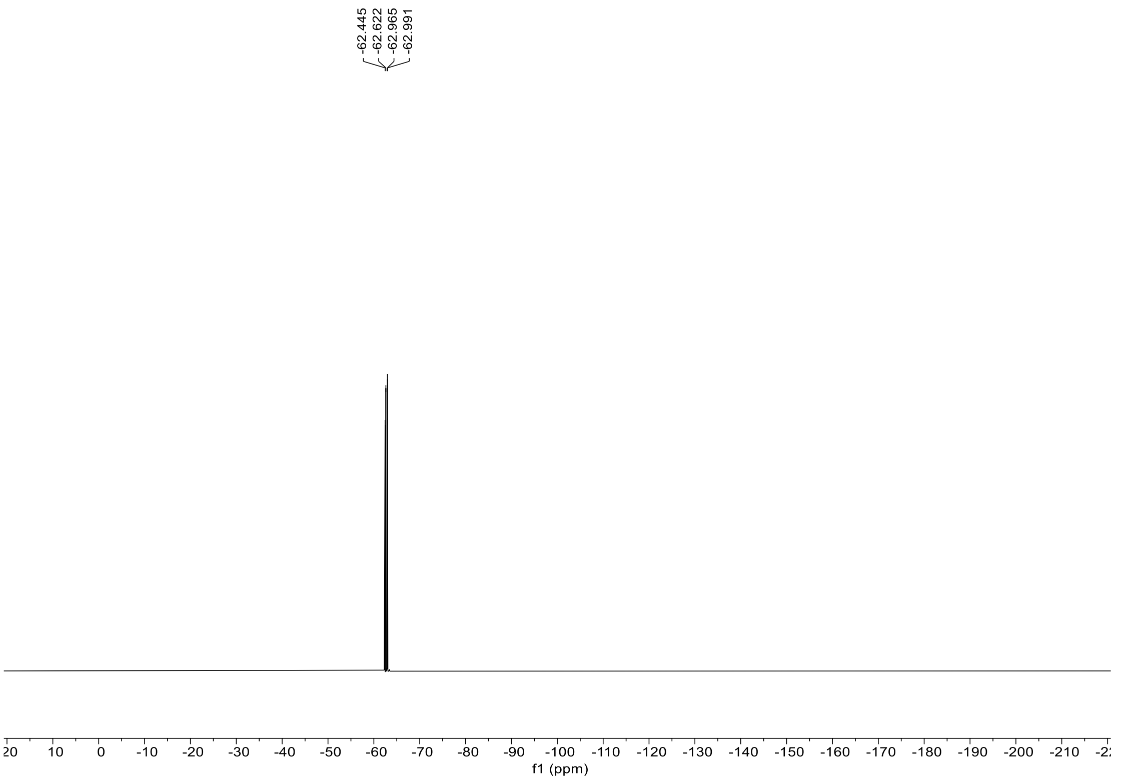
-62.445
-62.622
-62.965
-62.991
f1 (ppm)

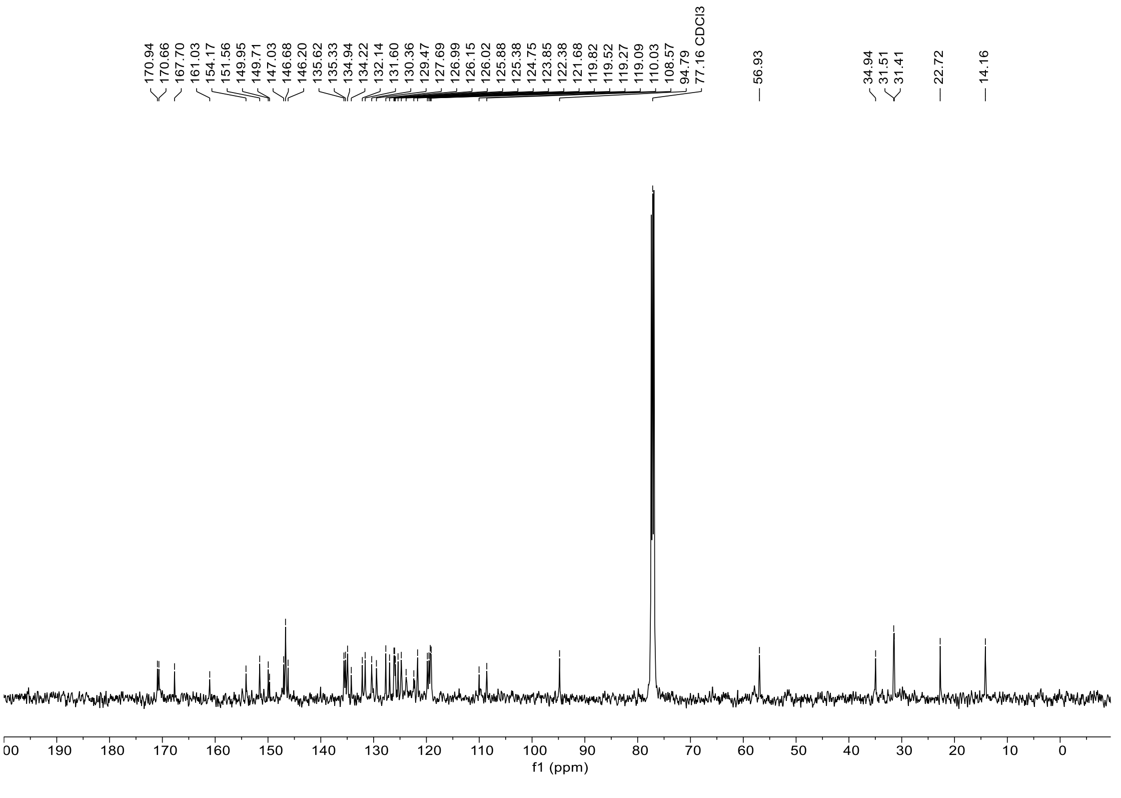
170.94
170.66
167.70
161.03
154.17
151.56
149.95
149.71
147.03
146.68
146.20
135.62
135.33
134.94
134.22
132.14
131.60
130.36
129.47
127.69
126.99
126.15
126.02
125.88
125.38
124.75
123.85
122.38
121.68
119.82
119.52
119.27
119.09
110.03
108.57
94.79
77.16 CDCl3
56.93
34.94
31.51
31.41
22.72
14.16
f1 (ppm)

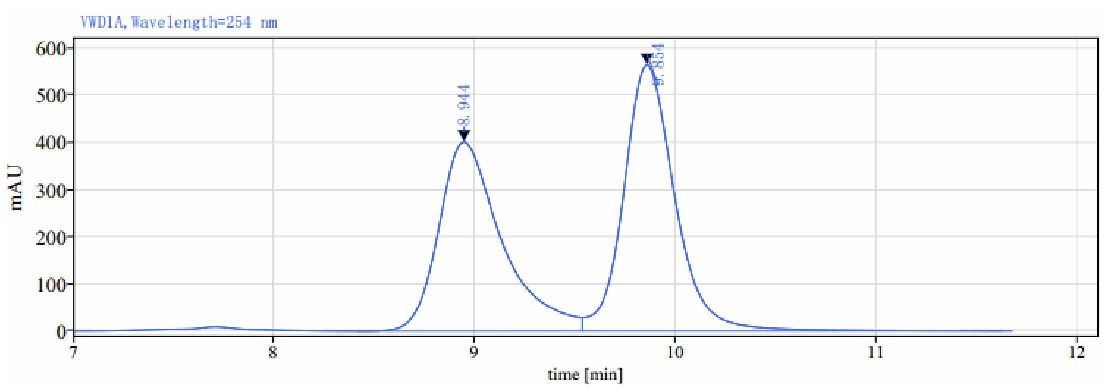

Rettime [min]	Type	Width [min]	Area	Height	Area%
8.944	BV	1.12	8784.54	401.29	46.74
9.854	VB	2.09	10008.67	564.09	53.26

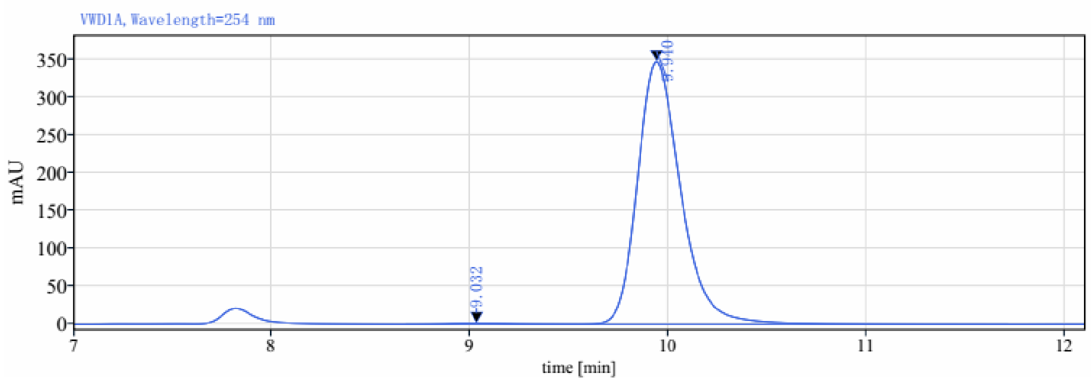

Rettime [min]	Type	Width [min]	Area	Height	Area%
9.032	BV	0.87	28.92	1.38	0.55
9.940	VB	2.17	5265.31	346.59	99.45

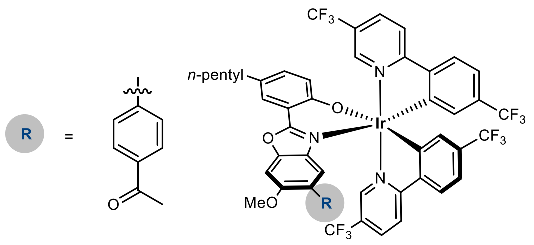

Analytical data of Δ-**21:**

^{1}H NMR (500 MHz, Chloroform-*d*) δ 9.31 (s, 1H), 8.41 (s, 1H), 8.03 – 7.87 (m, 6H), 7.71 – 7.64 (m, 3H), 7.18 – 7.08 (m, 5H), 7.03 (dd, *J* = 8.5, 2.5 Hz, 1H), 6.71 (s, 1H), 6.59 (d, *J* = 8.5 Hz, 1H), 6.24 (s, 1H), 5.95 (s, 1H), 3.81 (s, 3H), 2.63 (s, 3H), 2.50 (t, *J* = 7.5 Hz, 2H), 1.62 –

1.55 (m, 2H), 1.39 – 1.28 (m, 4H), 0.91 (t, *J* = 7.0 Hz, 3H).

^{19}F NMR (471 MHz, $CDCl_3$) δ -62.37, -62.56, -62.87, -63.00.

^{13}C NMR (126 MHz, $CDCl_3$) δ 198.0, 170.9, 170.5, 167.3, 160.8, 155.3, 151.6, 150.3, 150.2, 147.1, 146.8, 146.7, 146.2, 142.3, 135.8, 135.5, 135.3, 134.9, 133.9, 131.8, 131.5, 130.2, 129.8, 128.0, 127.6, 127.5, 127.1, 126.0, 125.7, 125.3, 124.3, 123.8, 123.7, 122.4, 122.2, 119.8, 119.5, 119.2, 119.0, 118.8, 110.6, 94.4, 56.3, 34.9, 31.5, 31.4, 26.7, 22.7, 14.1.

HRMS (ESI) for $C_{53}H_{39}IrF_{12}N_3O_4$ $[M+H]^+$ calcd.1202.2373, found 1202.2353

Enantiomeric excess established by HPLC analysis using a Chiralpak IK-3 column, ee = 86% (HPLC: IK-3, 254 nm, *n*-hexane/isopropanol = 90:10, flow rate 1.0 mL/min, 40 °C, t_r (major) = 11.2 min, t_r (minor) = 10.3 min.)

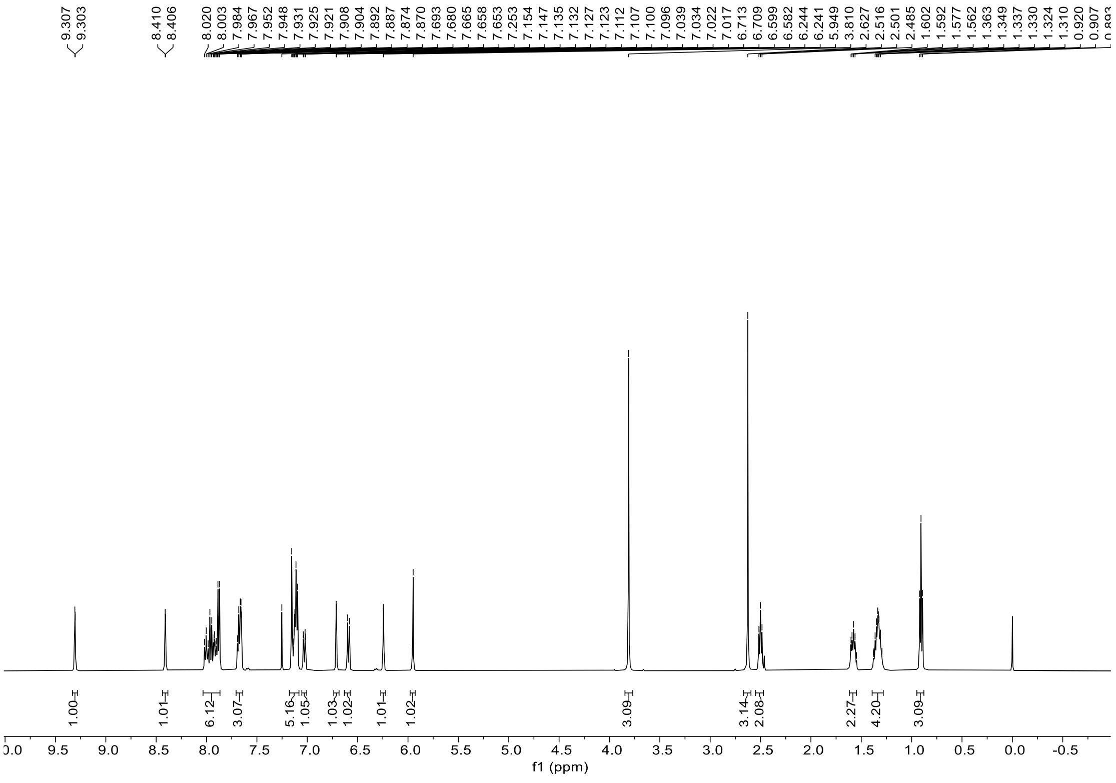

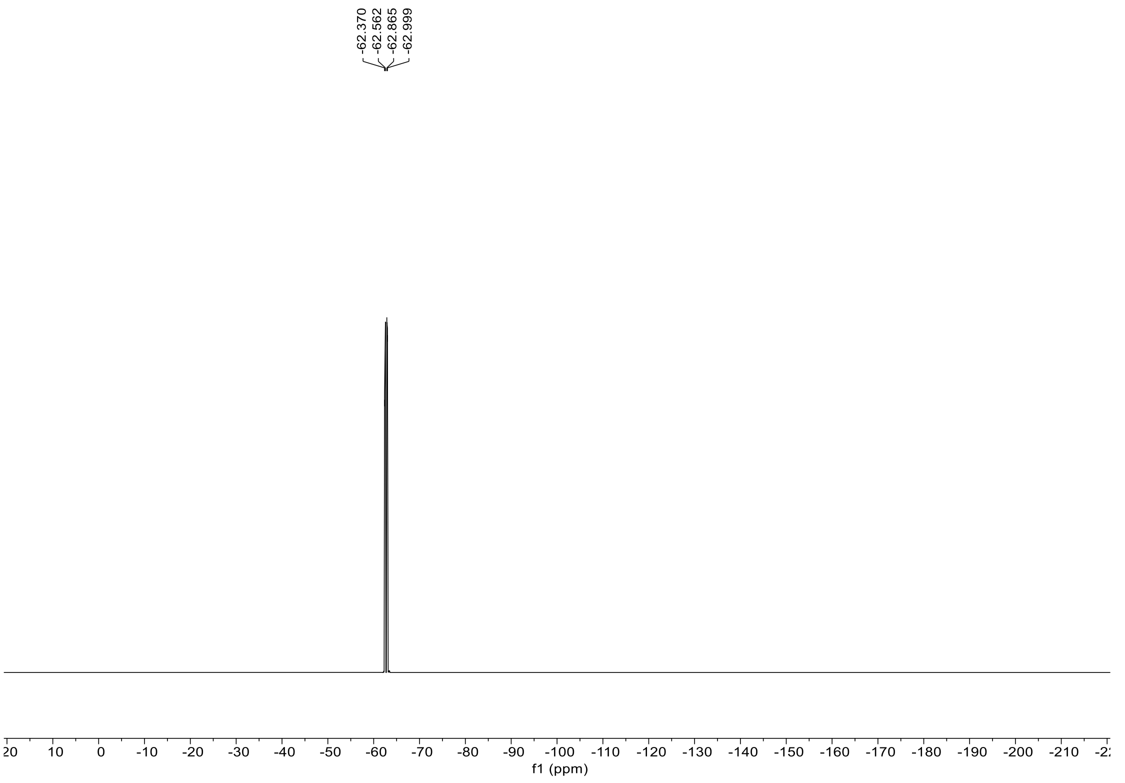
-62.370
-62.562
-62.865
-62.999
20 10 0 -10 -20 -30 -40 -50 -60 -70 -80 -90 -100 -110 -120 -130 -140 -150 -160 -170 -180 -190 -200 -210
f1 (ppm)

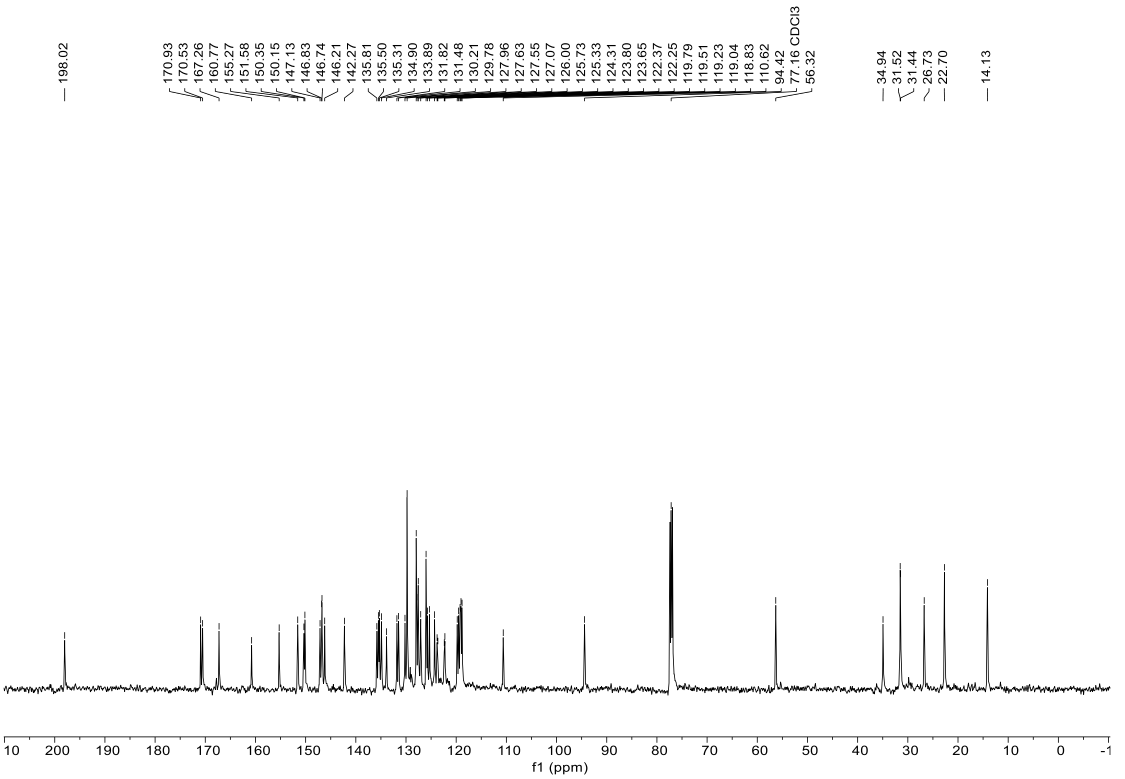
198.02
170.93
170.53
167.26
160.77
155.27
151.58
150.35
150.15
147.13
146.83
146.74
146.21
142.27
135.81
135.50
135.31
134.90
133.89
131.82
131.48
130.21
129.78
127.96
127.63
127.55
127.07
126.00
125.73
125.33
124.31
123.80
123.65
122.37
122.25
119.79
119.51
119.23
119.04
118.83
110.62
94.42
77.16 CDCl3
56.32
34.94
31.52
31.44
26.73
22.70
14.13
200 190 180 170 160 150 140 130 120 110 100 90 80 70 60 50 40 30 20 10 0
f1 (ppm)

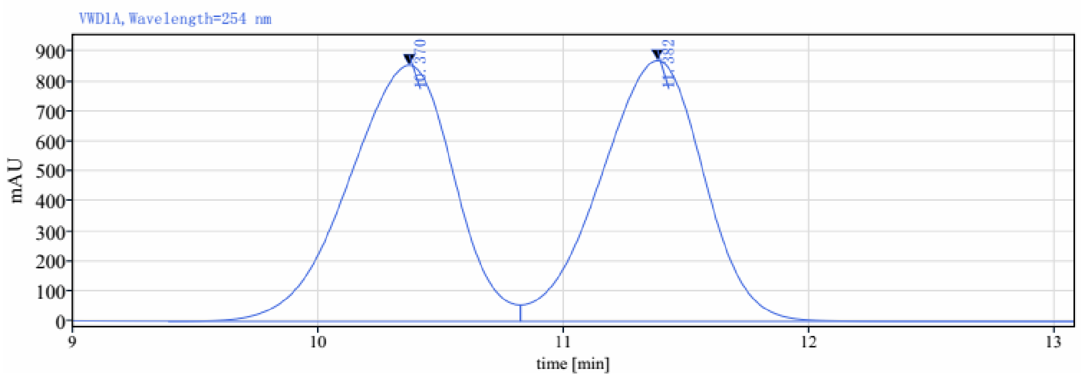

Rettime [min]	Type	Width [min]	Area	Height	Area%
10.370	VV	1.43	25455.93	853.89	49.94
11.382	VB	2.52	25513.62	869.80	50.06

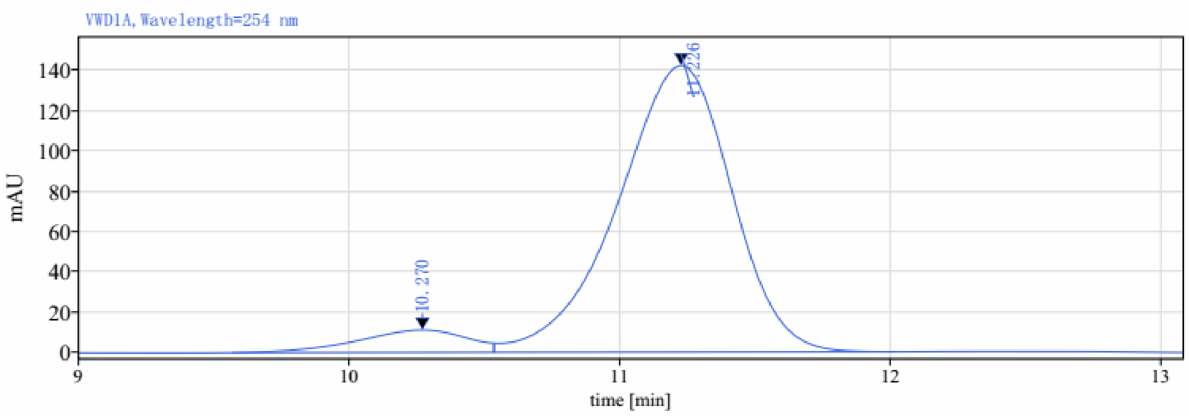

Rettime [min]	Type	Width [min]	Area	Height	Area%
10.270	MM m	0.97	308.14	11.01	6.91
11.226	MB m	1.48	4149.19	141.96	93.09

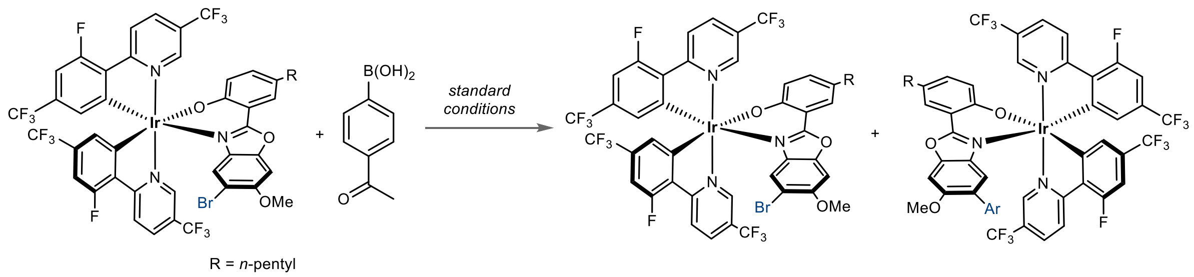

According to the general procedure, the reaction was carried out at 30 ºC for 4 days to give the

recovered Λ-**22** as red solid (22.1 mg, 37% yield) and Δ-**23** as red solid (27.2 mg, 44% yield).

Purification conditions: petroleum ether/EtOAc = 10:1 to 5:1.

R_f (Λ-**22**) = 0.5 in petroleum ether/EtOAc (5:1).

R_f (Δ-**23**) = 0.3 in petroleum ether/EtOAc (5:1).

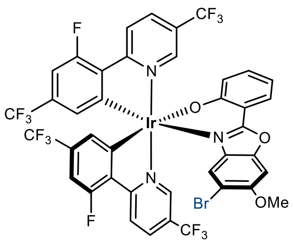

Analytical data of Λ-**22:**

^{1}H NMR (500 MHz, Chloroform-*d*) δ 9.21 (s, 1H), 8.54 (dd, *J* = 8.5, 3.0 Hz, 1H), 8.49 (dd, *J* = 8.5, 2.5 Hz, 1H), 8.36 (s, 1H), 8.01 (dd, *J* = 8.5, 2.0 Hz, 2H), 7.83 (dd, *J* = 8.0, 2.0 Hz, 1H), 7.25 – 7.20 (m, 1H), 7.09 – 7.03 (m, 2H), 6.93 (dd, *J* = 12.5, 1.5 Hz, 1H), 6.72 (d, *J* = 8.5 Hz, 1H), 6.61 – 6.53 (m, 1H), 6.50 (d, *J* = 1.5 Hz, 1H), 6.07 (s, 1H), 6.05 (s, 1H), 3.89 (s, 3H).

^{19}F NMR (471 MHz, $CDCl_3$) δ -62.70, -62.78, -63.20, -63.23, -109.98, -111.15.

^{13}C NMR (126 MHz, $CDCl_3$) δ 168.9, 168.3, 168.0, 161.7, 161.0, 160.9, 154.4, 154.0, 152.8, 149.7, 146.6, 136.0, 135.7, 134.9, 134.8, 134.1, 133.8, 133.7, 133.1, 128.5, 126.2, 126.1, 125.8, 125.0, 124.3, 123.8, 123.2, 122.9, 122.8, 122.1, 122.0, 121.5, 115.6, 110.5, 108.9, 107.7, 107.0, 94.9, 57.0.

HRMS (ESI) for $C_{40}H_{20}IrBrF_{14}N_3O_3$ $[M+H]^+$ calcd.1128.0088, found 1128.0076

Enantiomeric excess established by HPLC analysis using a Chiralpak OD-3 column, ee = 94% (HPLC: OD-3, 254 nm, *n*-hexane/isopropanol = 99:1, flow rate 1.0 mL/min, 40 ^{o}C, t_r (major) = 14.3 min, t_r (minor) = 11.5 min.)

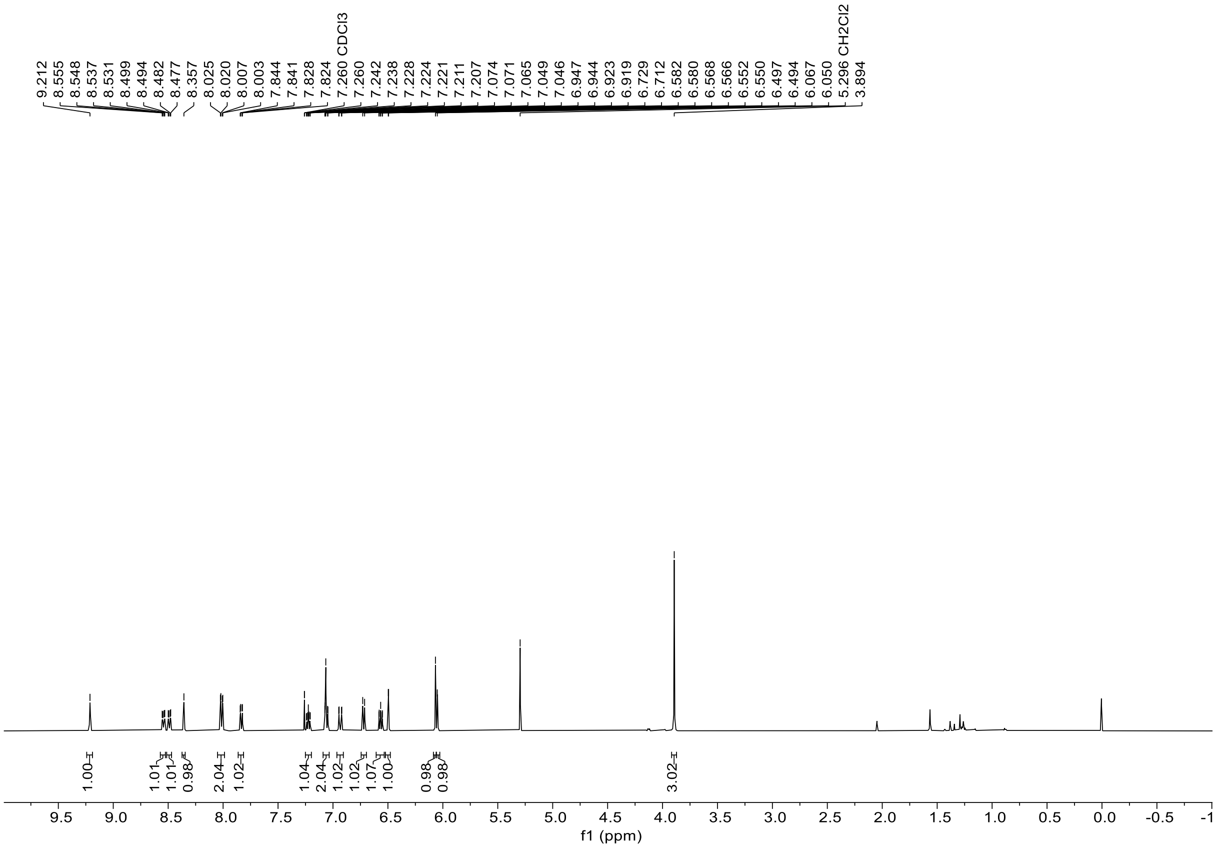
9.212
8.555
8.548
8.537
8.531
8.499
8.494
8.482
8.477
8.357
8.025
8.020
8.007
8.003
7.844
7.841
7.828
7.824
7.260 CDCl3
7.260
7.242
7.238
7.228
7.224
7.221
7.211
7.207
7.074
7.071
7.065
7.049
7.046
6.947
6.944
6.923
6.919
6.729
6.712
6.582
6.580
6.568
6.566
6.552
6.550
6.497
6.494
6.067
6.050
5.296 CH2Cl2
3.894
1.00
1.01
1.01
0.98
2.04
1.02
1.04
2.04
1.02
1.02
1.07
1.00
0.98
0.98
3.02
9.5
9.0
8.5
8.0
7.5
7.0
6.5
6.0
5.5
5.0
4.5
4.0
3.5
3.0
2.5
2.0
1.5
1.0
0.5
0.0
-0.5
f1 (ppm)

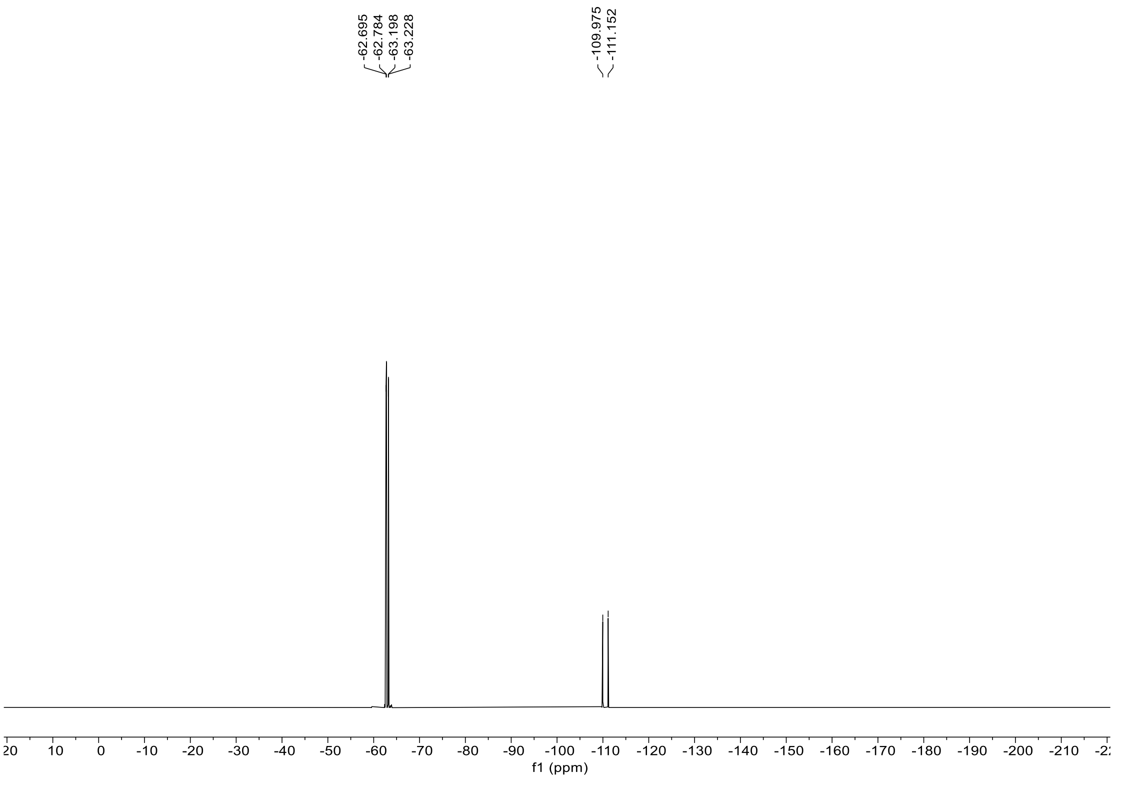
-62.695
-62.784
-63.198
-63.228
-109.975
-111.152
10
0
-10
-20
-30
-40
-50
-60
-70
-80
-90
-100
-110
-120
-130
-140
-150
-160
-170
-180
-190
-200
-210
f1 (ppm)

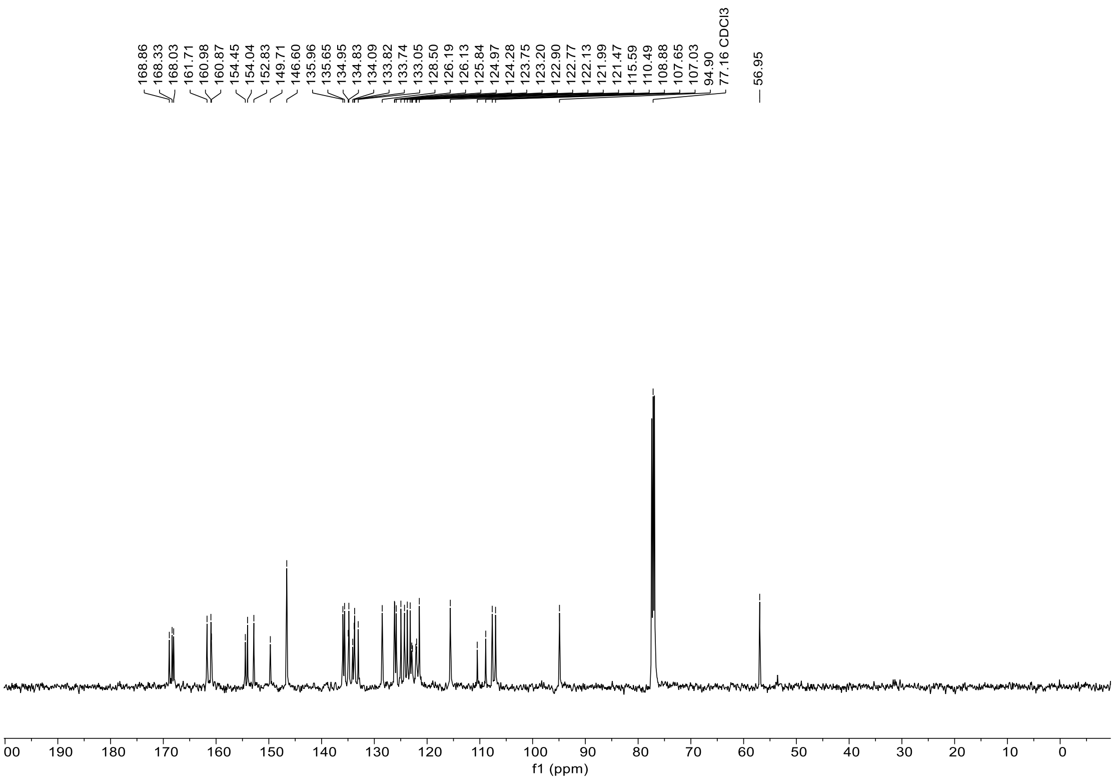

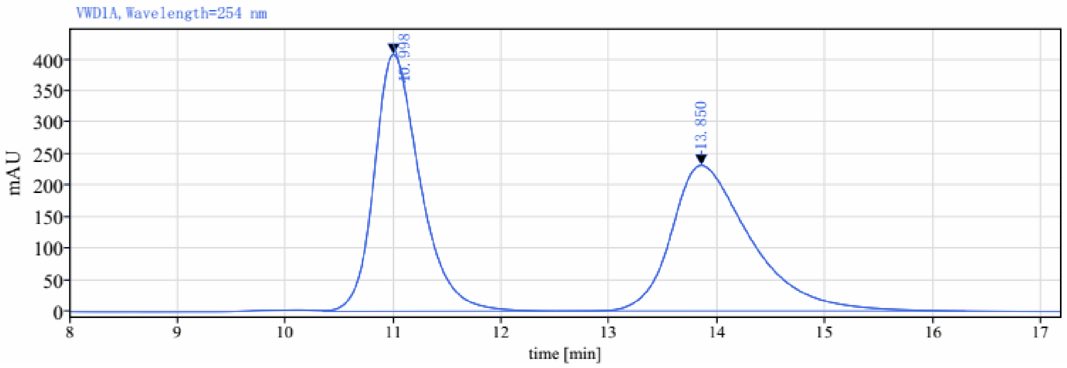

Rettime [min]	Type	Width [min]	Area	Height	Area%
10.998	VB	2.17	12022.59	407.12	50.41
13.850	BM m	3.78	11827.99	230.73	49.59

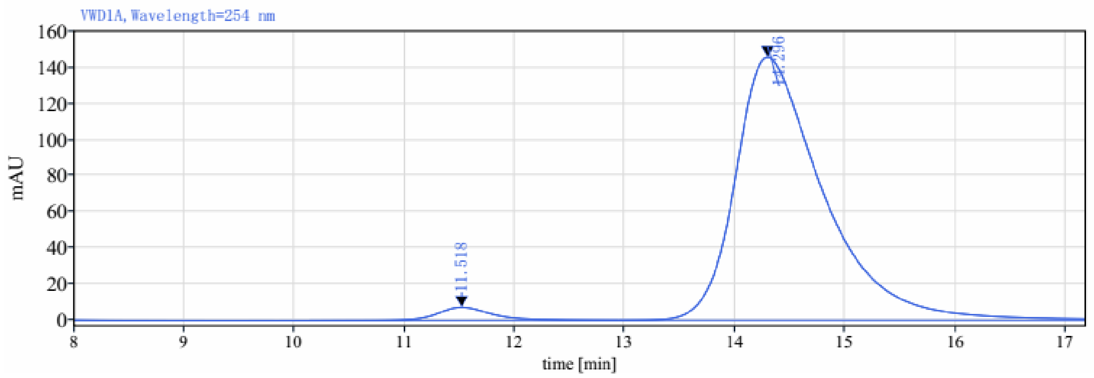

Rettime [min]	Type	Width [min]	Area	Height	Area%
11.518	BB	2.80	246.98	7.21	2.94
14.296	BB	7.60	8154.51	145.81	97.06

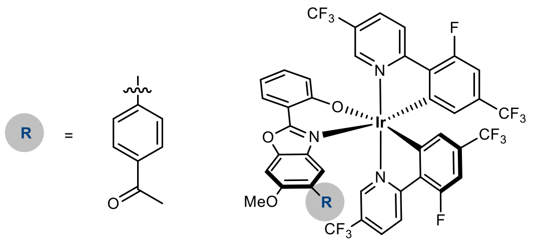

Analytical data of Δ-**23:**

^{1}H NMR (500 MHz, Chloroform-*d*) δ 9.27 (d, *J* = 2.0 Hz, 1H), 8.52 (dd, *J* = 9.0, 3.0 Hz, 1H), 8.48 – 8.42 (m, 2H), 8.03 – 7.97 (m, 2H), 7.91 (dd, *J* = 8.5, 2.0 Hz, 2H), 7.87 (dd, *J* = 8.0, 2.0 Hz, 1H), 7.24 – 7.19 (m, 1H), 7.17 – 7.12 (m, 3H), 6.91 (dd, *J* = 12.5, 2.0 Hz, 1H), 6.81 (dd, *J* = 12.0, 1.5 Hz, 1H), 6.72 – 6.68 (m, 1H), 6.62 – 6.57 (m, 1H), 6.46 (d, *J* = 1.5 Hz, 1H), 6.01 (d, *J* = 1.5 Hz, 1H), 5.99 – 5.96 (m, 1H), 3.83 (s, 3H), 2.64 (s, 3H).

^{19}F NMR (471 MHz, $CDCl_3$) δ -62.62, -62.73, -63.16, -63.23, -110.13, -111.19.

^{13}C NMR (126 MHz, $CDCl_3$) δ 197.9, 168.7, 168.4, 168.0, 161.6, 160.9, 160.6, 155.5, 154.1, 153.3, 150.3, 146.8, 146.7, 142.2, 136.0, 135.6, 135.0, 134.7, 134.1, 133.4, 133.0, 129.6, 128.5, 128.1, 126.2, 126.0, 125.8, 124.7, 124.2, 123.7, 123.1, 122.9, 122.7, 122.1, 122.0, 118.8, 115.7, 111.1, 107.7, 106.7, 94.5, 56.4, 26.8.

HRMS (ESI) for $C_{48}H_{27}IrF_{14}N_3O_4$ $[M+H]^+$ calcd.1168.1402, found 1168.1394

Enantiomeric excess established by HPLC analysis using a Chiralpak OD-3 column, ee = 85% (HPLC: OD-3, 365 nm, *n*-hexane/isopropanol = 98:2, flow rate 1.0 mL/min, 40 °C, t_r (major) = 19.8 min, t_r (minor) = 15.7 min.)

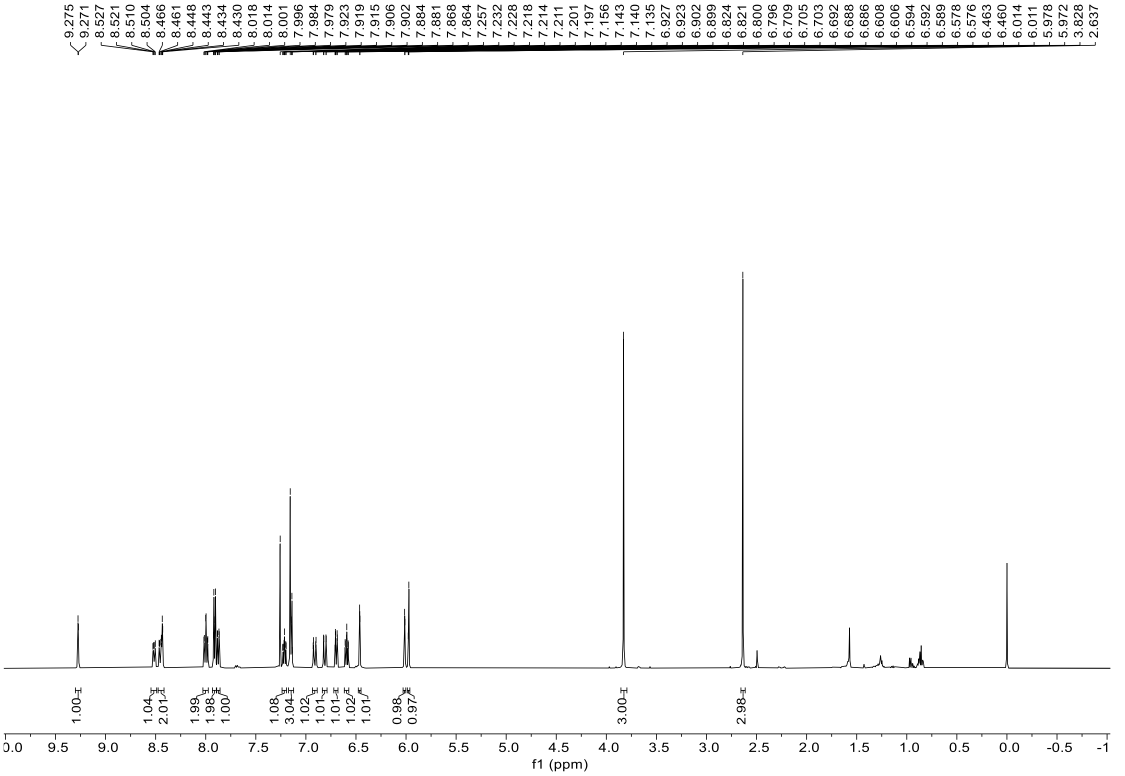

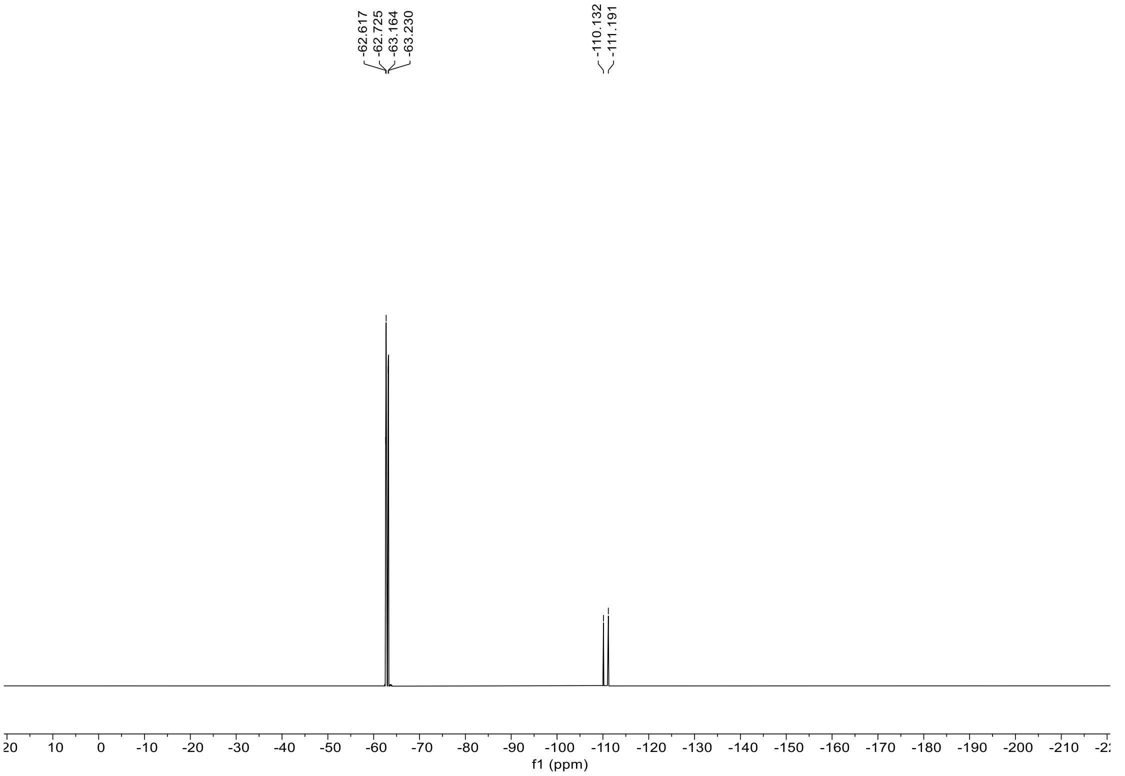

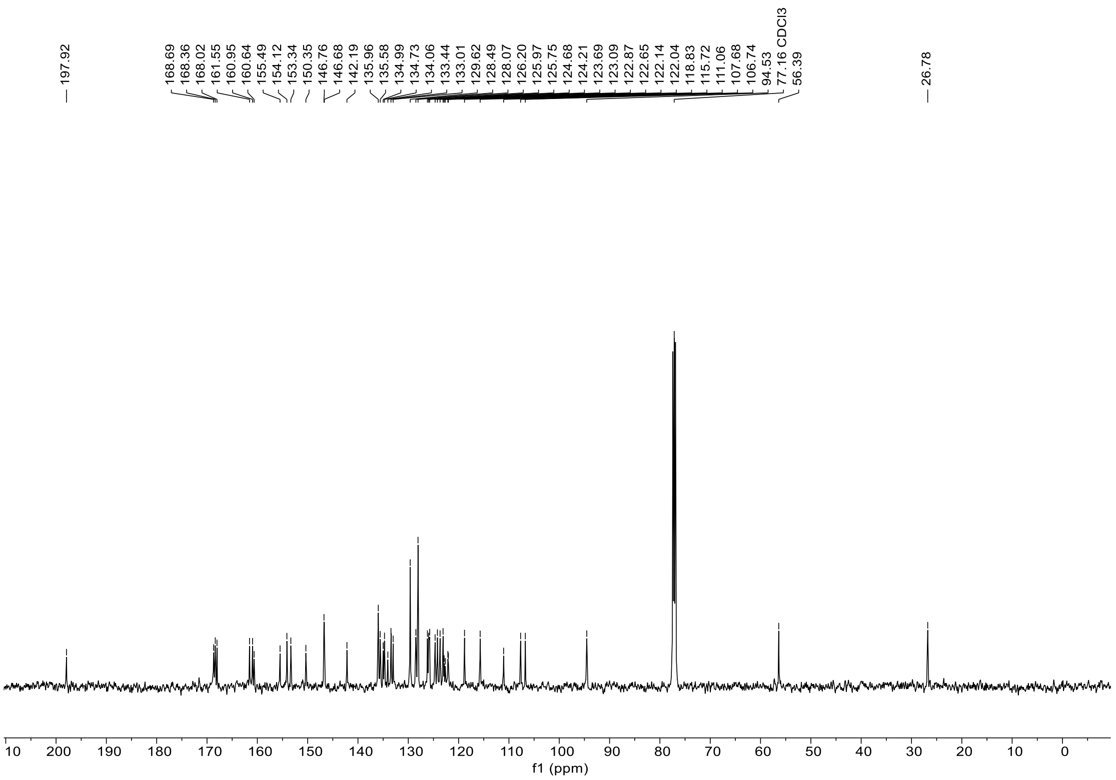

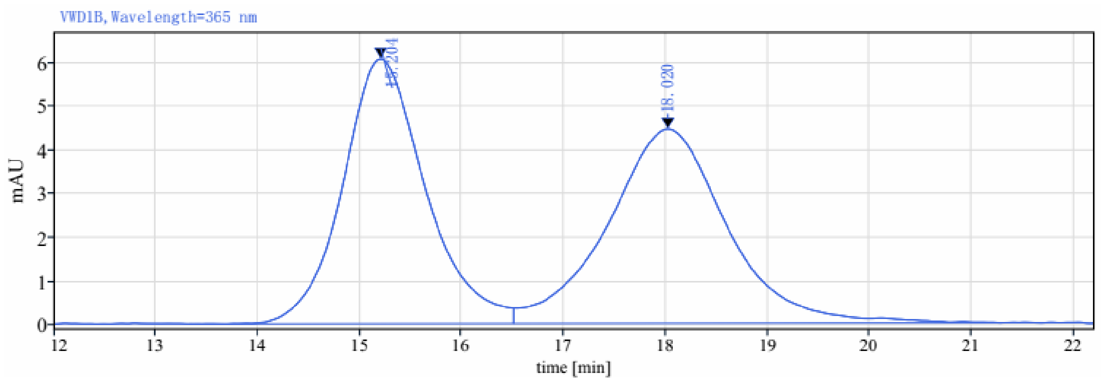

Rettime [min]	Type	Width [min]	Area	Height	Area%
15.204	MM m	3.18	341.44	6.04	48.71
18.020	MM m	4.78	359.58	4.43	51.29

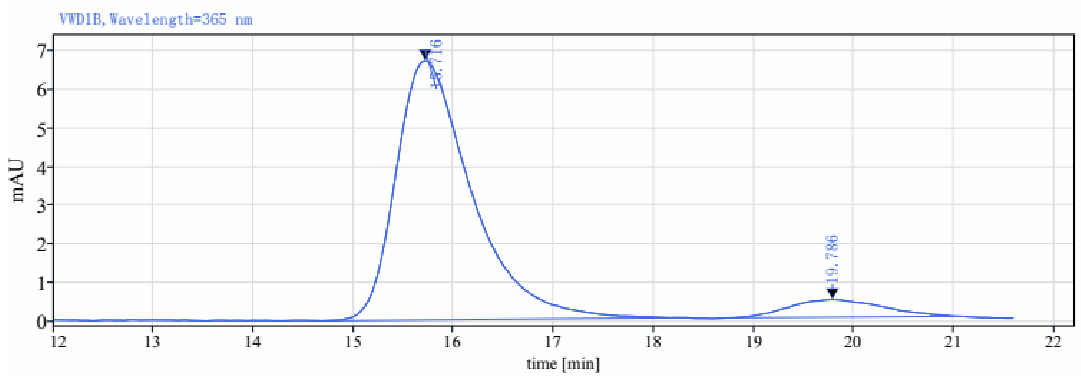

Rettime [min]	Type	Width [min]	Area	Height	Area%
15.716	BM m	3.46	352.74	6.70	92.30
19.786	MM m	2.21	29.44	0.46	7.70

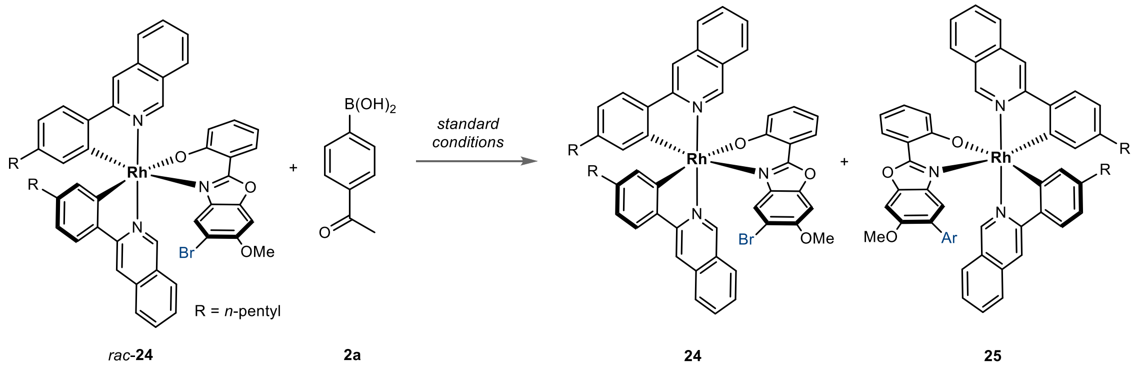

According to the general procedure, the reaction was carried out at 30 °C for 3 days to give the recovered **24** as yellow solid (24.2 mg, 50% yield) and **25** as yellow solid (19.0 mg, 38% yield). Purification conditions: petroleum ether/EtOAc/Et_3N = 50:10:1 to 50:30:1.

R_f (**24**) = 0.5 in petroleum ether/EtOAc (50:25:1).

R_f (**25**) = 0.3 in petroleum ether/EtOAc (50:25:1).

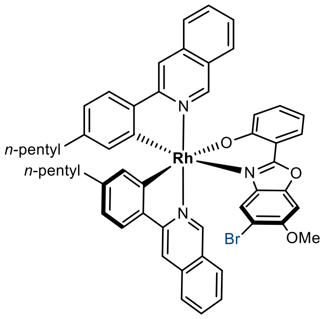

Analytical data of **24:**

^1H NMR (500 MHz, Chloroform-*d*) δ 9.58 (s, 1H), 8.73 (s, 1H), 8.05 (d, *J* = 8.5 Hz, 2H), 7.84 (dd, *J* = 8.0, 2.0 Hz, 1H), 7.77 (q, *J* = 7.5 Hz, 3H), 7.66 (d, *J* = 8.0 Hz, 1H), 7.62 – 7.49 (m, 4H), 7.41 (t, *J* = 7.5 Hz, 1H), 7.33 (t, *J* = 7.5 Hz, 1H), 7.12 – 7.04 (m, 1H), 6.93 (s, 1H), 6.87 (dd, *J* = 8.0, 1.5 Hz, 1H), 6.78 (d, *J* = 8.5 Hz, 1H), 6.74 – 6.67 (m, 1H), 6.35 (t, *J* = 7.5 Hz, 1H), 6.20 (d, *J* = 8.5 Hz, 2H), 5.99 (s, 1H), 3.78 (s, 3H), 2.32 – 2.16 (m, 4H), 1.44 – 1.24 (m, 4H), 1.16 – 0.96 (m, 8H), 0.75 – 0.60 (m, 6H).

^{13}C NMR (126 MHz, $CDCl_3$) δ 171.3, 167.2, 167.0, 166.5, 166.2, 163.9, 158.9, 158.4, 153.4, 153.3, 152.7, 149.4, 143.4, 143.0, 141.9, 141.3, 136.5, 135.5, 134.7, 133.7, 133.0, 131.63, 131.57, 129.0, 128.2, 127.6, 127.2, 126.8, 126.76, 126.5, 125.8, 123.4, 123.2, 122.92, 122.88, 122.7, 114.3, 113.9, 112.5, 110.1, 107.8, 94.2, 56.8, 36.0, 35.9, 31.48, 31.46, 30.8, 30.1, 22.53, 22.51, 14.08, 14.06.

HRMS (ESI) for $C_{54}H_{50}BrN_3O_3Rh$ $[M+H]^+$ calcd.970.2085, found 970.2076;

Enantiomeric excess established by HPLC analysis using a Chiralpak IK-3 column, ee = 0 (HPLC: IK-3, 254 nm, *n*-hexane/isopropanol = 80:20, flow rate 1.0 mL/min, 40 °C, t_r (major) = 9.3 min, t_r (minor) = 10.8 min.)

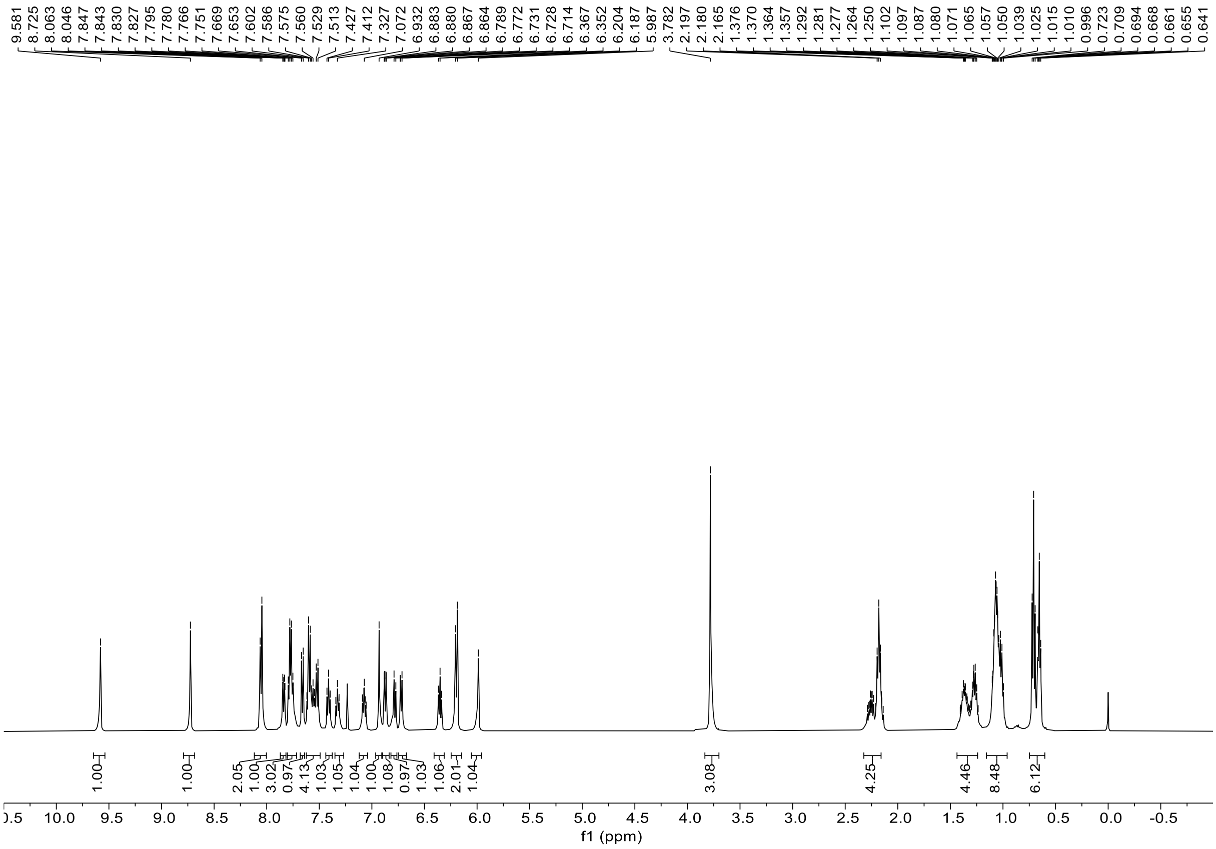

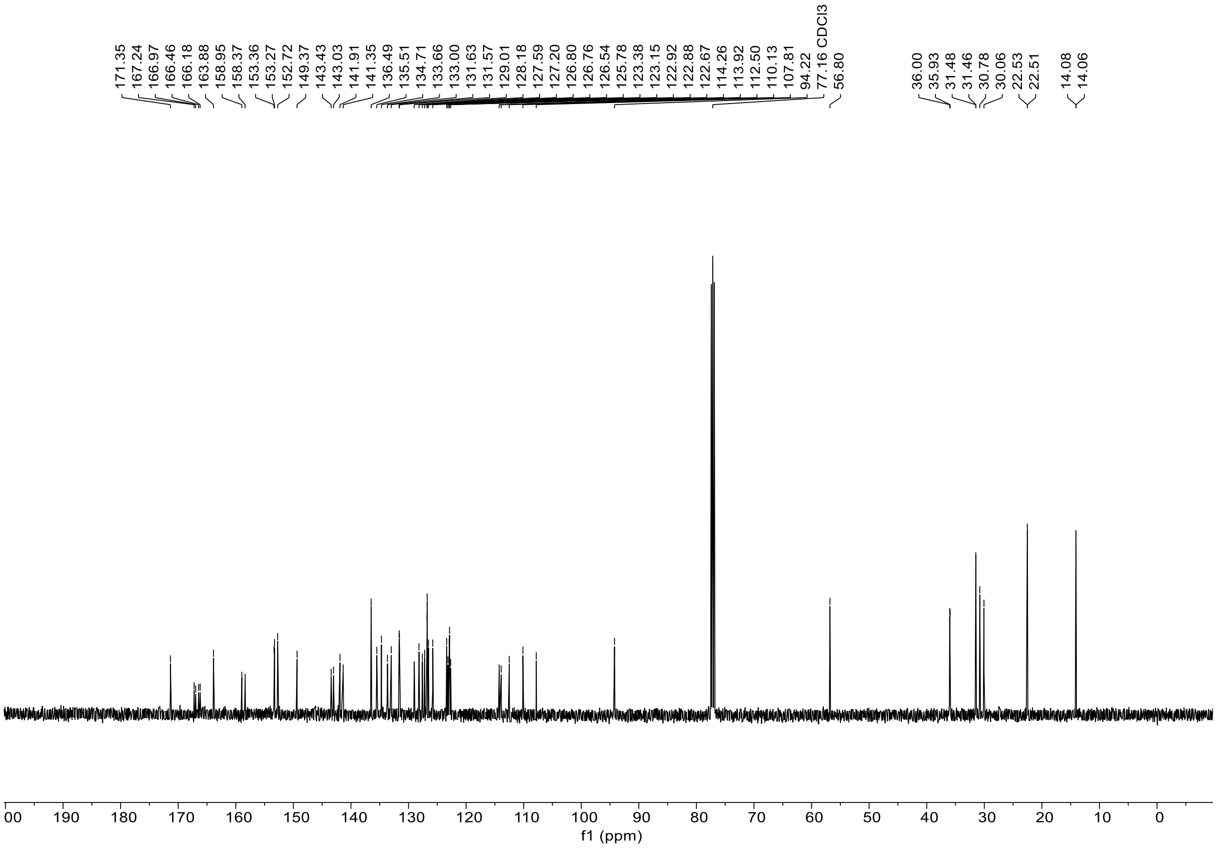

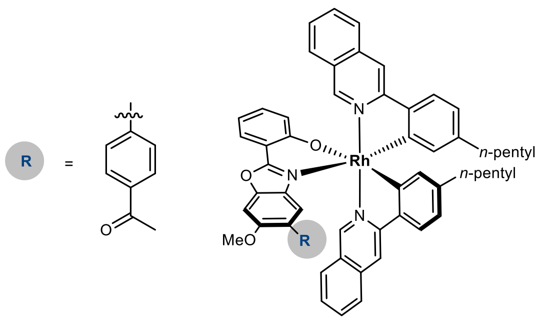

Analytical data of **25:**

^{1}H NMR (500 MHz, Chloroform-*d*) δ 9.67 (s, 1H), 8.83 (s, 1H), 8.11 (s, 2H), 7.98 – 7.78 (m, 6H), 7.70 – 7.57 (m, 5H), 7.51 – 7.45 (m, 1H), 7.42 – 7.36 (m, 1H), 7.25 (dd, *J* = 8.4, 2.1 Hz, 2H), 7.11 (s, 2H), 6.86 – 6.74 (m, 3H), 6.49 – 6.36 (m, 2H), 6.20 (s, 1H), 5.99 (d, *J* = 3.5 Hz, 1H), 3.80 (s, 3H), 2.65 (s, 3H), 2.26 – 2.18 (m, 2H), 2.16 – 2.06 (m, 2H), 1.35 – 1.28 (m, 2H), 1.15 – 1.08 (m, 2H), 1.05 – 0.86 (m, 8H), 0.75 (t, *J* = 7.0 Hz, 3H), 0.64 (t, *J* = 7.0 Hz, 3H).

^{13}C NMR (126 MHz, $CDCl_3$) δ 197.9, 171.1, 167.4, 167.1, 166.9, 166.6, 163.6, 159.0, 158.4, 154.6, 153.4, 152.9, 150.1, 143.1, 143.0, 142.8, 142.0, 141.3, 136.5, 135.4, 135.2, 134.6, 133.6, 132.9, 131.7, 131.6, 130.3, 128.9, 128.3, 127.8, 127.6, 127.25, 127.18, 126.8, 126.7, 126.5, 125.5, 123.3, 123.0, 122.6, 120.4, 114.2, 113.8, 112.6, 110.6, 93.8, 56.3, 35.91, 35.87, 31.4,

31.3, 30.3, 30.1, 26.7, 22.5, 22.4, 14.1, 14.0.

HRMS (ESI) for $C_{62}H_{57}N_3O_4Rh$ $[M+H]^+$ calcd.1010.3399, found 1010.3384;

Enantiomeric excess established by HPLC analysis using a Chiralpak IM column, ee = 0 (HPLC: IM, 254 nm, *n*-hexane/isopropanol = 80:20, flow rate 1.0 mL/min, 40 °C, t_r (major) = 17.4 min, t_r (minor) = 19.4 min.)

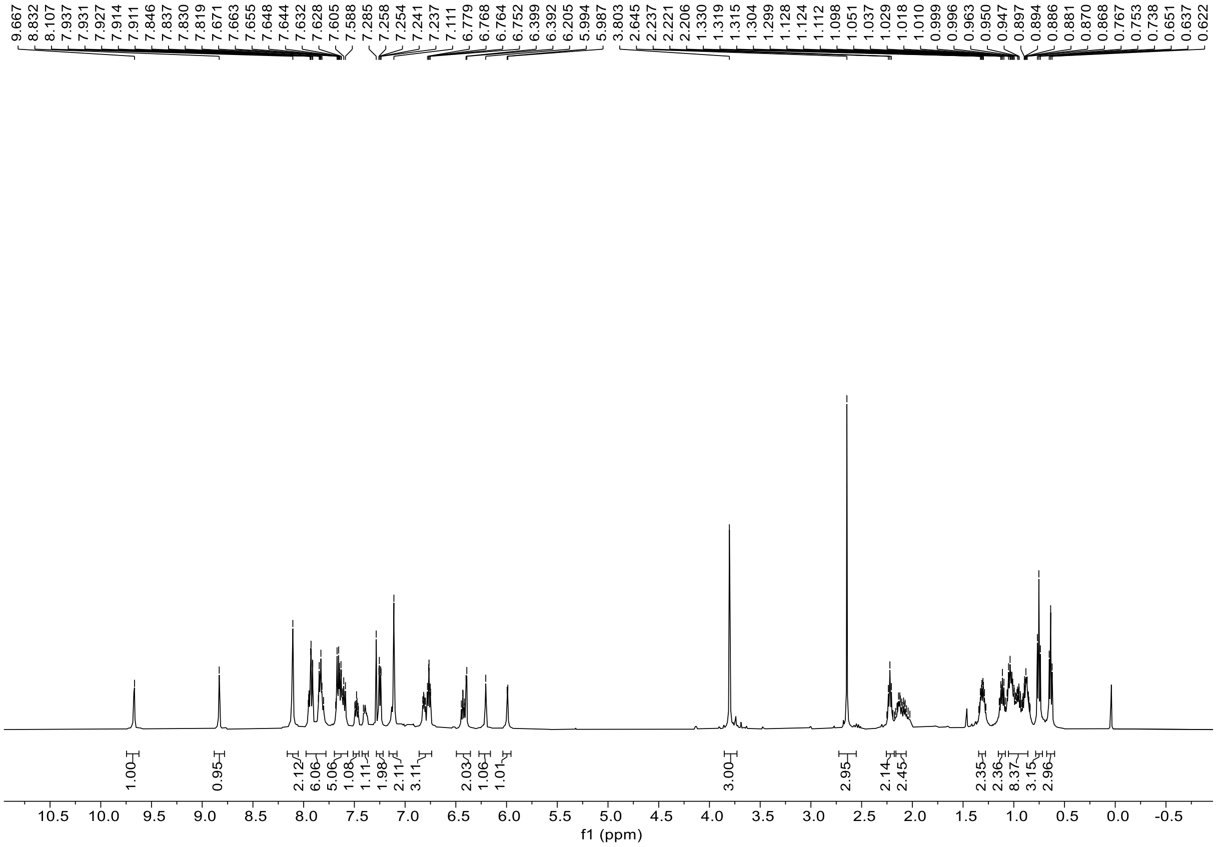

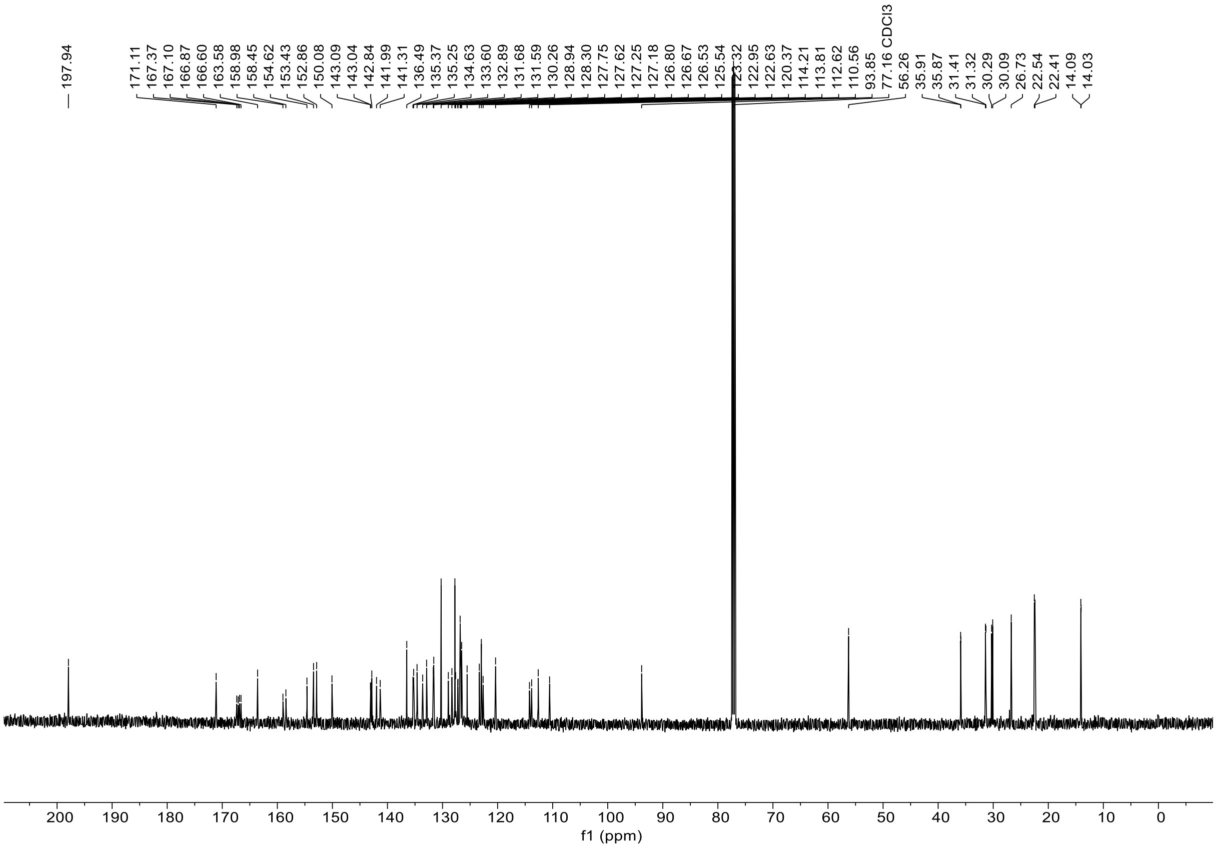

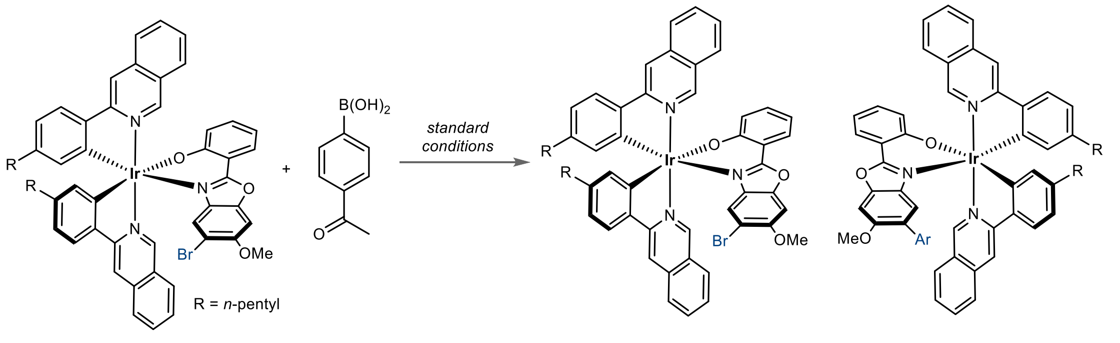

rac-**28** **2a** Λ-**28** Δ-**29**

According to the general procedure, the reaction was carried out at 30 ºC for 3.5 days to give the recovered Λ-**28** as orange solid (25.9 mg, 49% yield) and Δ-**29** as orange solid (24.2 mg, 44% yield).

Purification conditions: petroleum ether/EtOAc = 10:1 to 2:1.

R_f (Λ-**28**) = 0.6 in petroleum ether/EtOAc (2:1).

R_f (Δ-**29**) = 0.4 in petroleum ether/EtOAc (2:1).

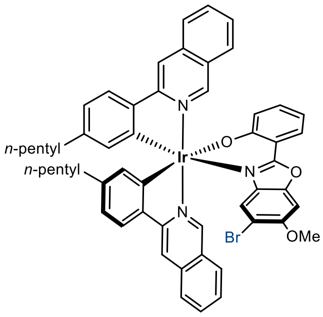

Analytical data of Λ-**28:**

^{1}H NMR (500 MHz, $CDCl_3$) δ 9.60 (d, *J* = 7.2 Hz, 1H), 8.80 (d, *J* = 4.0 Hz, 1H), 8.07 (dd, *J* = 15.6, 4.0 Hz, 2H), 7.86 (dt, *J* = 8.0, 2.4 Hz, 1H), 7.83 – 7.74 (m, 3H), 7.63 (dd, *J* = 8.0, 2.4 Hz, 1H), 7.61 – 7.51 (m, 4H), 7.44 – 7.38 (m, 1H), 7.35 (t, *J* = 7.2 Hz, 1H), 7.12 (ddd, *J* = 10.8, 5.6, 2.1 Hz, 1H), 6.96 – 6.91 (m, 1H), 6.84 – 6.74 (m, 2H), 6.70 (dt, *J* = 8.5, 2.5 Hz, 1H), 6.39 (dd, *J* = 8.3, 6.7 Hz, 1H), 6.18 (dd, *J* = 13.1, 4.8 Hz, 2H), 5.94 (t, *J* = 4.5 Hz, 1H), 3.86 – 3.76 (m, 3H), 2.30 – 2.15 (m, 4H), 1.42 – 1.26 (m, 4H), 1.15 – 1.01 (m, 8H), 0.70 (dtd, *J* = 21.8, 7.1, 2.4 Hz, 6H).

^{13}C NMR (126 MHz, $CDCl_3$) δ 169.5, 162.1, 161.7, 160.3, 153.6, 152.0, 151.9, 149.5, 149.1, 145.6, 143.7, 143.1, 142.4, 141.9, 136.6, 136.5, 135.3, 134.1, 133.4, 132.1, 131.7, 131.6, 128.5, 128.1, 127.6, 127.5, 127.4, 126.55, 126.45, 126.4, 126.0, 123.4, 123.2, 122.7, 122.1, 122.0, 113.8, 113.6, 113.3, 110.7, 108.0, 94.1, 56.8, 36.0, 35.9, 31.6, 31.57, 30.9, 30.2, 22.6, 14.1.

HRMS (ESI) for $C_{54}H_{50}IrBrN_3O_3$ $[M+H]^+$ calcd.1060.2660, found 1060.2656

Enantiomeric excess established by HPLC analysis using a Chiralpak IK-3 column, ee = 92% (HPLC: IK-3, 254 nm, *n*-hexane/isopropanol = 80:20, flow rate 1.0 mL/min, 40 ^{o}C, t_r (major) = 8.9 min, t_r (minor) = 10.2 min.)

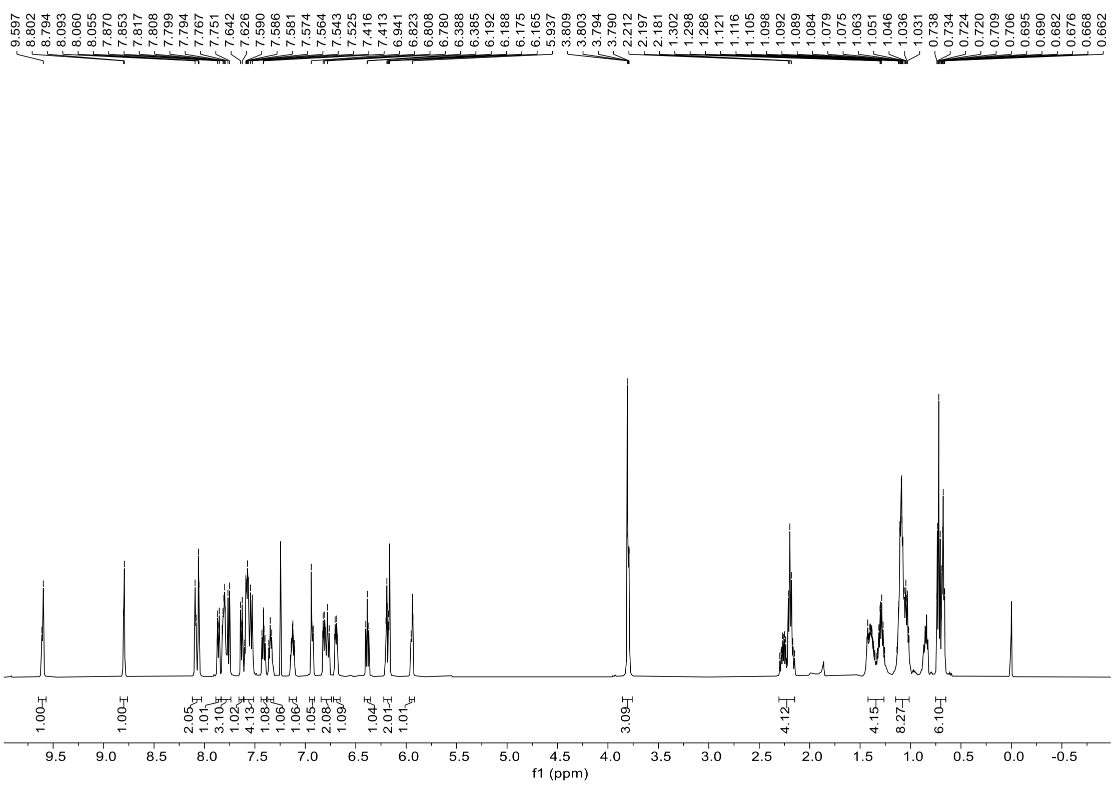
9.597
8.802
8.794
8.093
8.060
8.055
7.870
7.853
7.817
7.808
7.799
7.794
7.767
7.751
7.642
7.626
7.590
7.586
7.581
7.574
7.564
7.543
7.525
7.416
7.413
6.941
6.823
6.808
6.780
6.388
6.385
6.192
6.188
6.175
6.165
5.937
3.809
3.803
3.794
3.790
2.212
2.197
2.181
1.302
1.298
1.286
1.121
1.116
1.105
1.098
1.092
1.089
1.084
1.079
1.075
1.063
1.051
1.046
1.036
1.031
0.738
0.734
0.724
0.720
0.709
0.706
0.695
0.690
0.682
0.676
0.668
0.662
1.00
1.00
2.05
1.01
3.10
1.02
4.13
1.08
1.06
1.06
1.05
2.08
1.09
1.04
2.01
1.01
3.09
4.12
4.15
8.27
6.10
9.5
9.0
8.5
8.0
7.5
7.0
6.5
6.0
5.5
5.0
4.5
4.0
3.5
3.0
2.5
2.0
1.5
1.0
0.5
0.0
-0.5
f1 (ppm)

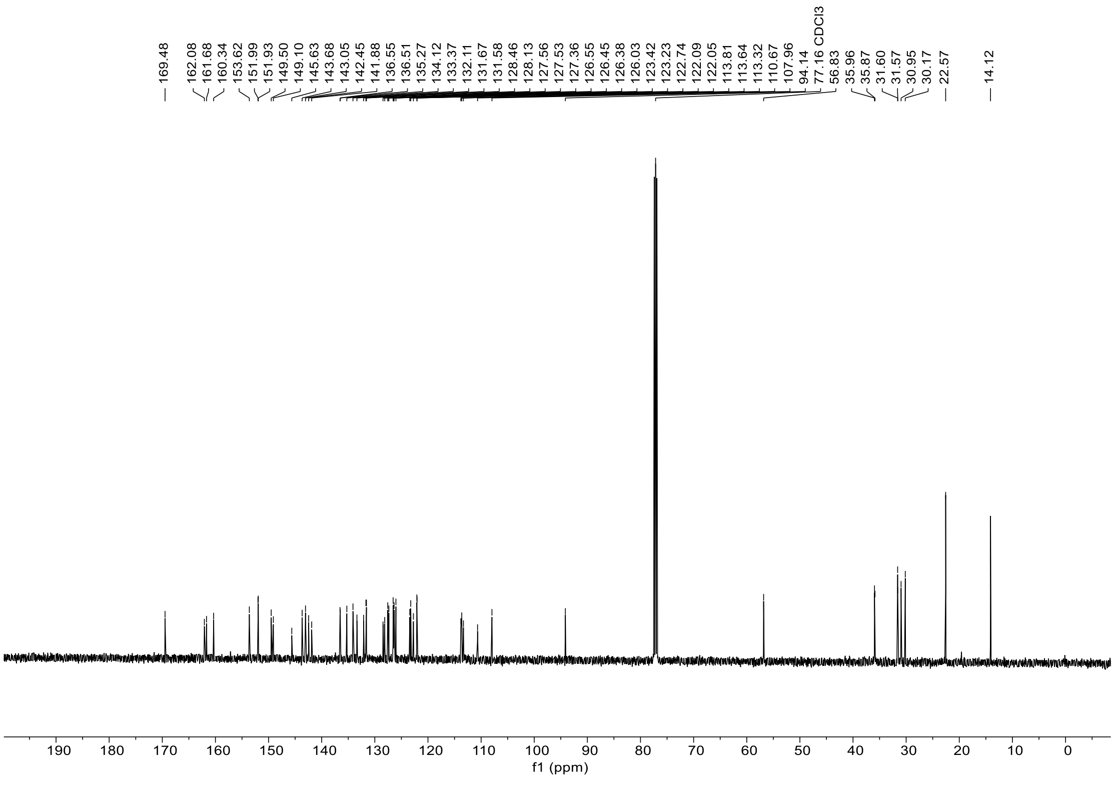
169.48
162.08
161.68
160.34
153.62
151.99
151.93
149.50
149.10
145.63
143.68
143.05
142.45
141.88
136.55
136.51
135.27
134.12
133.37
132.11
131.67
131.58
128.46
128.13
127.56
127.53
127.36
126.55
126.45
126.38
126.03
123.42
123.23
122.74
122.09
122.05
113.81
113.64
113.32
110.67
107.96
94.14
77.16 CDCl3
56.83
35.96
35.87
31.60
31.57
30.95
30.17
22.57
14.12
190
180
170
160
150
140
130
120
110
100
90
80
70
60
50
40
30
20
10
0
f1 (ppm)

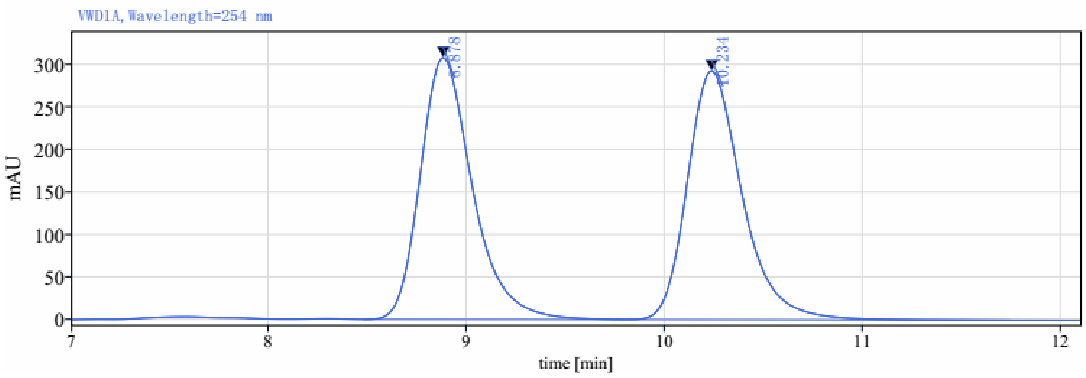

Rettime [min]	Type	Width [min]	Area	Height	Area%
8.878	BB	1.33	5644.43	307.62	50.05
10.234	BBA	1.94	5633.67	292.31	49.95

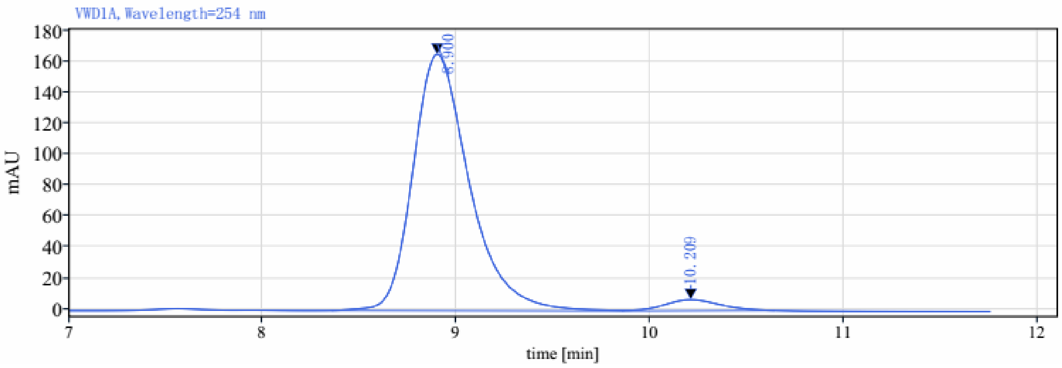

Rettime [min]	Type	Width [min]	Area	Height	Area%
8.900	MM m	1.51	3360.05	166.04	95.91
10.209	VM m	0.78	143.17	7.14	4.09

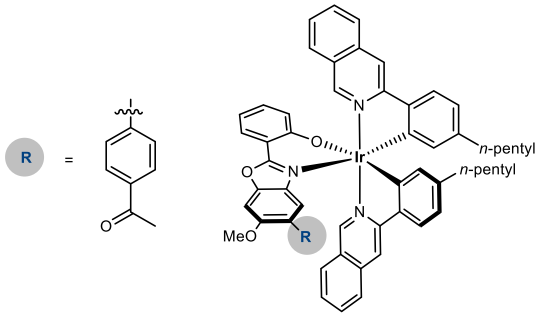

Analytical data of Δ-**29:**

^{1}H NMR (500 MHz, $CDCl_3$) δ 9.65 (s, 1H), 8.86 (s, 1H), 8.08 (d, *J* = 8.0 Hz, 2H), 7.92 (dd, *J* = 8.5, 2.0 Hz, 1H), 7.88 (d, *J* = 8.0 Hz, 2H), 7.84 – 7.77 (m, 3H), 7.62 – 7.54 (m, 5H), 7.43 (t, *J* = 7.5 Hz, 1H), 7.35 (t, *J* = 7.5 Hz, 1H), 7.19 (d, *J* = 8.0 Hz, 2H), 7.12 (d, *J* = 7.0 Hz, 1H), 7.07

(s, 1H), 6.77 (d, J = 8.5 Hz, 1H), 6.70 – 6.64 (m, 2H), 6.42 (t, J = 7.5 Hz, 1H), 6.36 (s, 1H), 6.14 (d, J = 1.5 Hz, 1H), 5.89 (d, J = 1.5 Hz, 1H), 3.77 (s, 3H), 2.61 (s, 3H), 2.19 (t, J = 7.7 Hz, 2H), 2.11 – 2.00 (m, 2H), 1.29 (dt, J = 8.2, 4.1 Hz, 2H), 1.12 – 1.06 (m, 2H), 1.03 – 0.82 (m, 8H), 0.72 (t, J = 7.2 Hz, 3H), 0.61 (t, J = 7.2 Hz, 3H).

^{13}C NMR (126 MHz, $CDCl_3$) δ 198.0, 169.2, 162.1, 161.7, 160.1, 154.9, 152.2, 152.1, 150.2, 149.3, 146.0, 143.3, 143.1, 142.6, 142.5, 141.9, 136.53, 136.5, 135.4, 135.1, 134.0, 133.4, 132.0, 131.7, 131.6, 130.3, 128.4, 128.2, 127.8, 127.55, 127.53, 127.4, 126.7, 126.6, 126.4, 125.7, 123.3, 122.7, 122.1, 122.0, 120.7, 113.8, 113.2, 111.1, 93.8, 56.3, 35.84, 35.79, 31.5, 31.4, 30.4, 30.2, 26.7, 22.6, 22.4, 14.11, 14.07.

HRMS (ESI) for $C_{62}H_{57}IrN_3O_4$ $[M+H]^+$ calcd.1100.3973, found 1100.3979

Enantiomeric excess established by HPLC analysis using a Chiralpak IK-3 column, ee = 94% (HPLC: IK-3, 254 nm, *n*-hexane/isopropanol = 80:20, flow rate 1.0 mL/min, 40 °C, t_r (major) = 20.8 min, t_r (minor) = 23.3 min.)

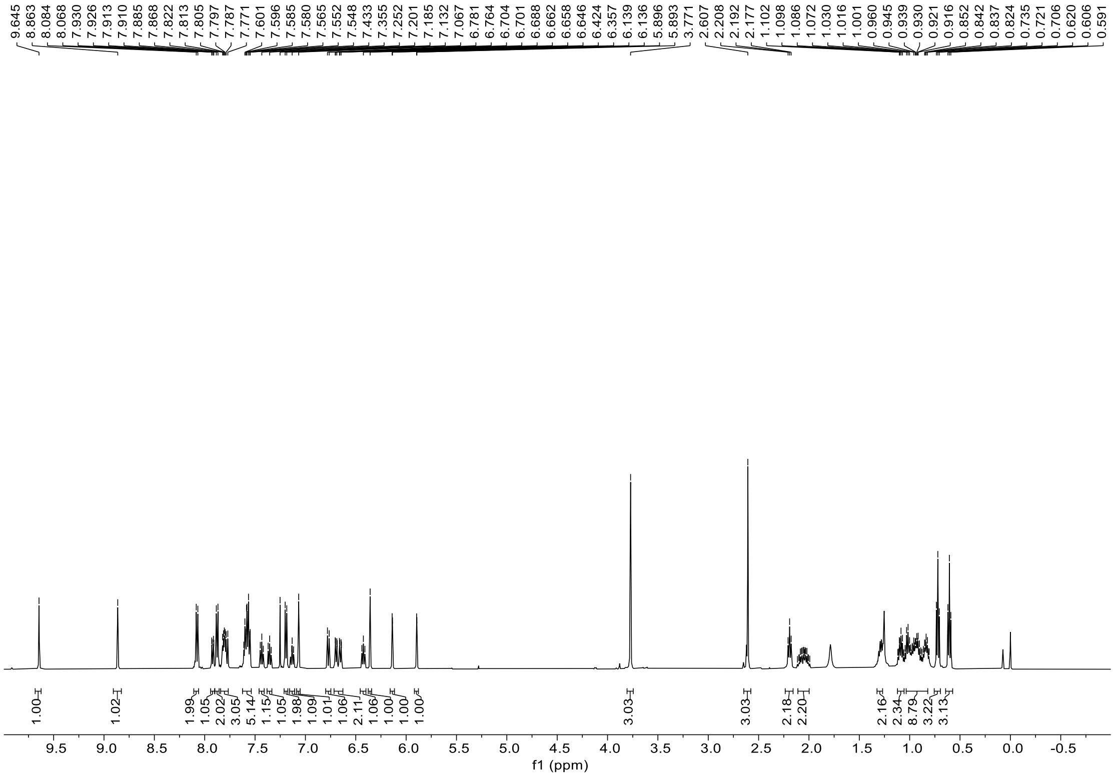

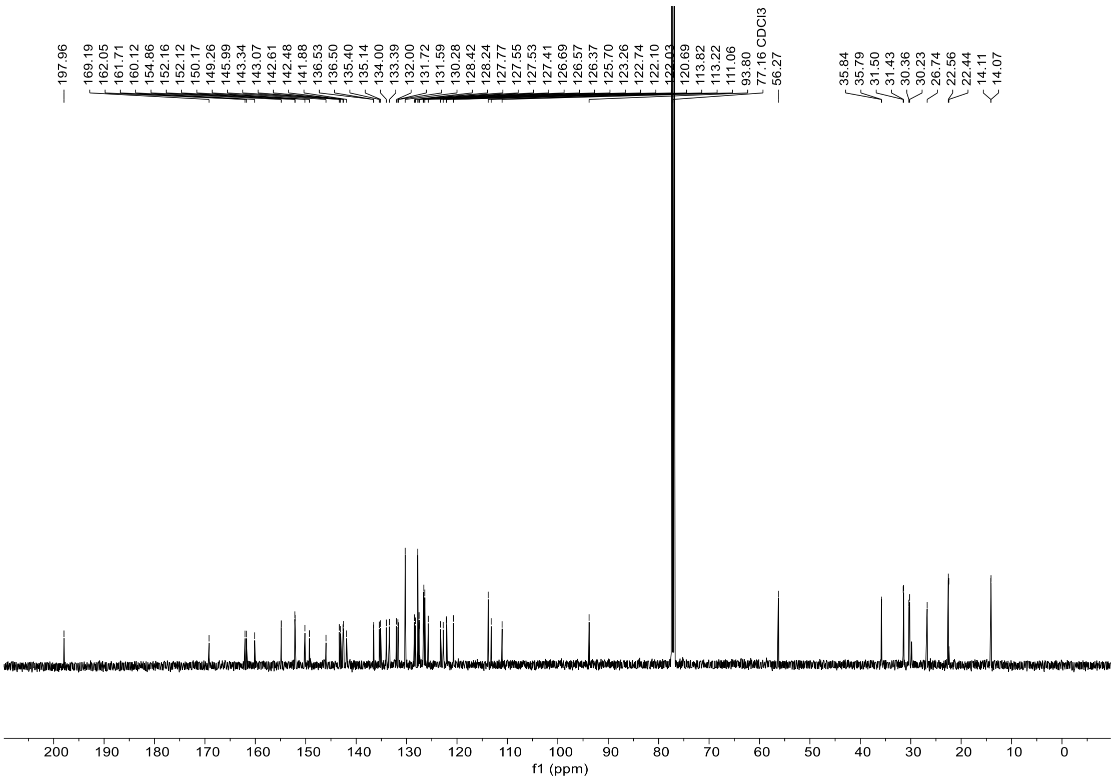

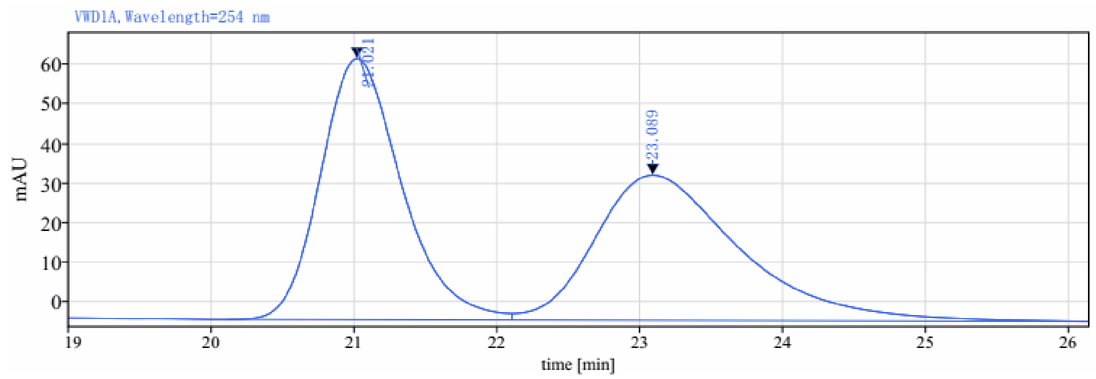

Rettime [min]	Type	Width [min]	Area	Height	Area%
21.021	BM m	1.99	2599.90	65.81	50.19
23.089	MM m	4.01	2579.89	36.61	49.81

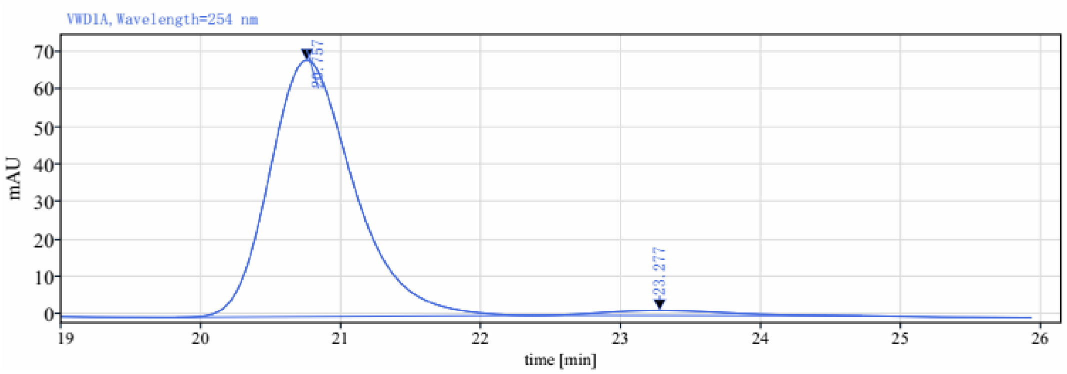

Rettime [min]	Type	Width [min]	Area	Height	Area%
20.757	BB	2.55	2853.37	68.30	97.17
23.277	BM m	2.34	83.05	1.36	2.83

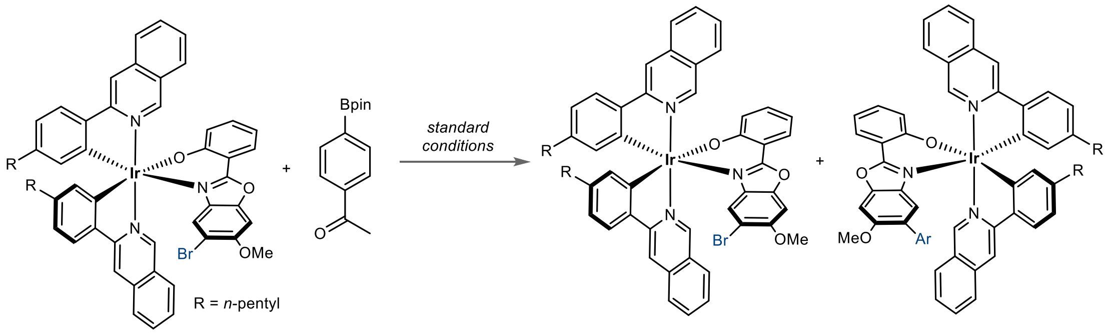

According to the general procedure, the reaction was carried out at 30 ºC for 4 days to give the recovered Λ-**28** as orange solid (22.7 mg, 43% yield) and Δ-**29** as orange solid (24.0 mg, 44% yield).

Purification conditions: petroleum ether/EtOAc = 10:1 to 2:1.

R_f (Λ-**28**) = 0.6 in petroleum ether/EtOAc (2:1).

R_f (Δ-**29**) = 0.4 in petroleum ether/EtOAc (2:1).

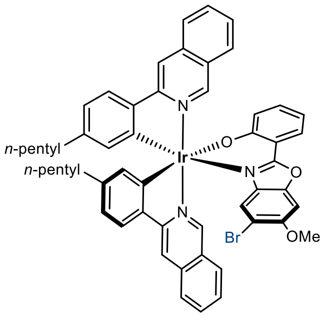

Enantiomeric excess established by HPLC analysis using a Chiralpak IK-3 column, ee = 89% (HPLC: IK-3, 254 nm, *n*-hexane/isopropanol = 80:20, flow rate 1.0 mL/min, 40 ºC, t_r (major)

= 8.9 min, t_r (minor) = 10.4 min.)

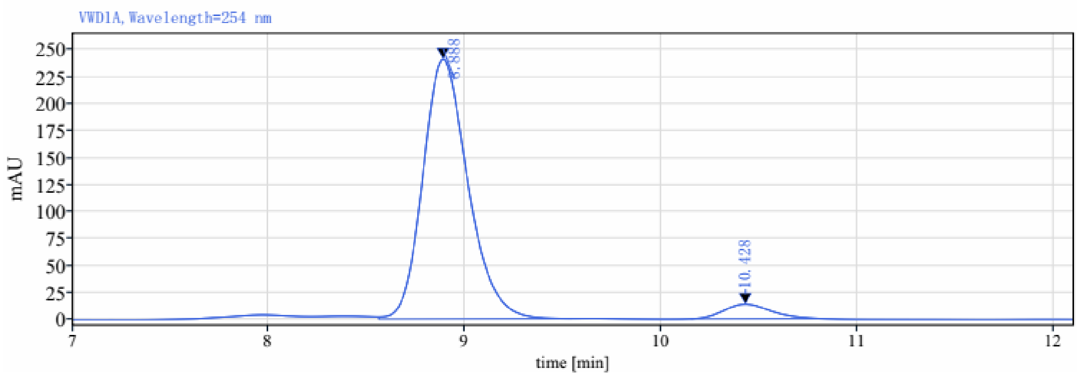

Rettime [min]	Type	Width [min]	Area	Height	Area%
8.888	VB	0.99	3847.87	240.84	94.46
10.428	MM m	0.67	225.54	13.39	5.54

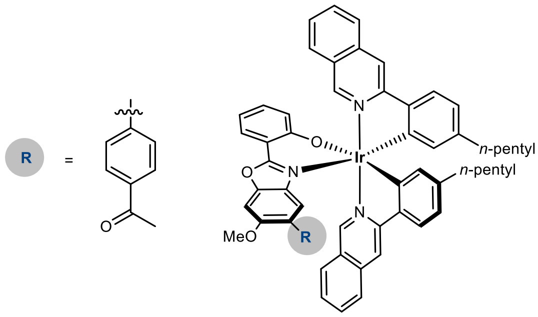

Enantiomeric excess established by HPLC analysis using a Chiralpak IK-3 column, ee = 94% (HPLC: IK-3, 254 nm, *n*-hexane/isopropanol = 80:20, flow rate 1.0 mL/min, 40 °C, t_r (major) = 21.3 min, t_r (minor) = 22.8 min.)

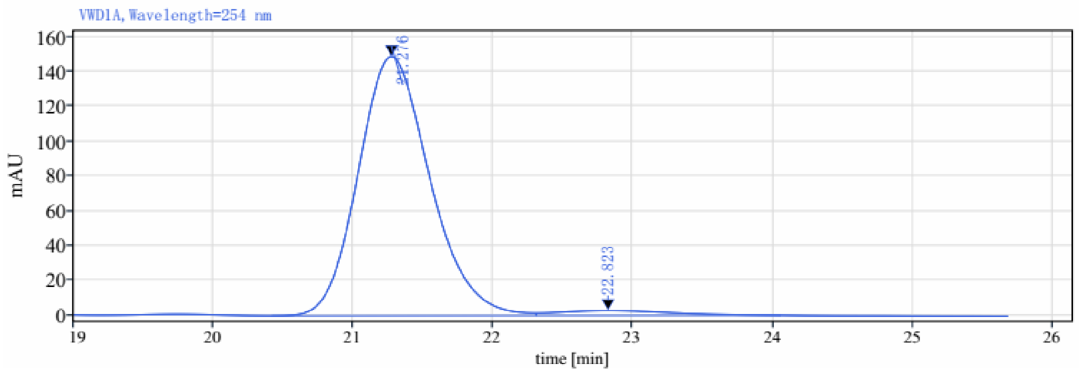

Rettime [min]	Type	Width [min]	Area	Height	Area%
21.276	BM m	1.89	5295.50	148.90	97.08
22.823	MM m	1.75	159.09	2.86	2.92

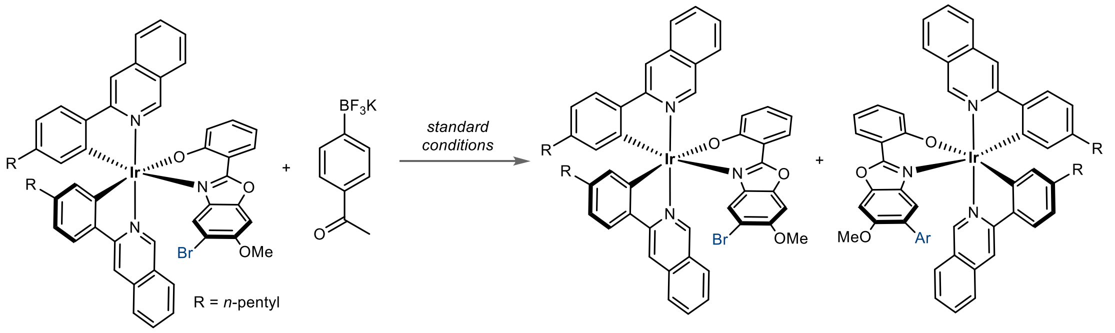

According to the general procedure, the reaction was carried out at 40 °C for 2.5 days to give the recovered Λ-**28** as orange solid (21.0 mg, 40% yield) and Δ-**29** as orange solid (21.4 mg, 39% yield).

Purification conditions: petroleum ether/EtOAc = 10:1 to 2:1.

R_f (Λ-**28**) = 0.6 in petroleum ether/EtOAc (2:1).

R_f (Δ-**29**) = 0.4 in petroleum ether/EtOAc (2:1).

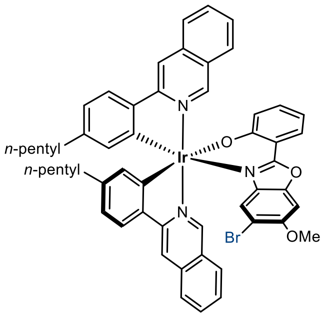

Enantiomeric excess established by HPLC analysis using a Chiralpak IK-3 column, ee = 92% (HPLC: IK-3, 254 nm, *n*-hexane/isopropanol = 80:20, flow rate 1.0 mL/min, 40 °C, t_r (major)

= 8.9 min, t_r (minor) = 10.4 min.)

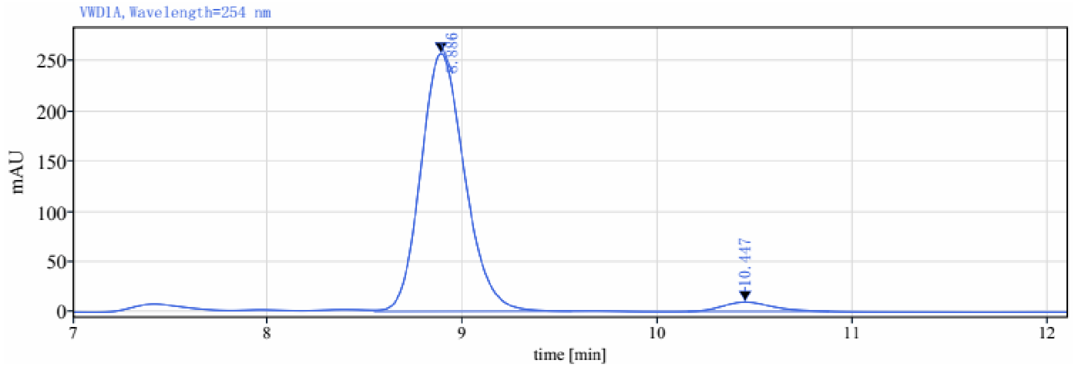

Rettime [min]	Type	Width [min]	Area	Height	Area%
8.886	VB	1.01	4024.48	257.42	96.11
10.447	MM m	0.73	162.96	9.40	3.89

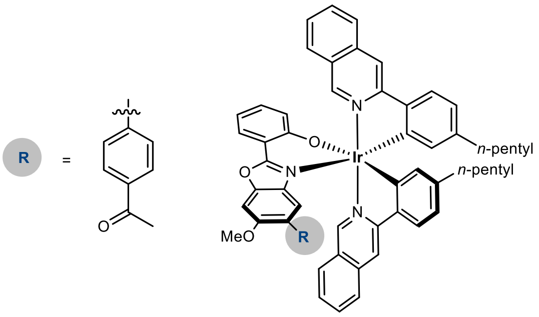

Enantiomeric excess established by HPLC analysis using a Chiralpak IK-3 column, ee = 90% (HPLC: IK-3, 254 nm, *n*-hexane/isopropanol = 80:20, flow rate 1.0 mL/min, 40 °C, t_r (major) = 21.5 min, t_r (minor) = 22.3 min.)

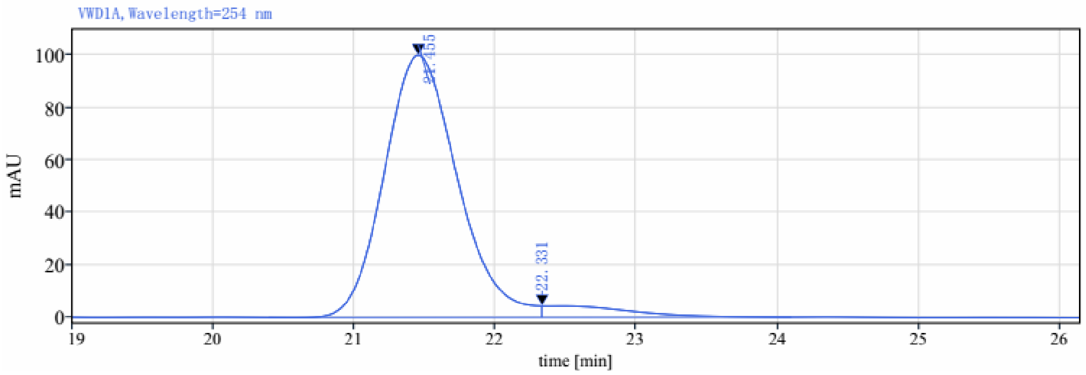

Rettime [min]	Type	Width [min]	Area	Height	Area%
21.455	MM m	1.93	3602.60	99.94	95.11
22.331	MM m	1.77	185.19	4.31	4.89

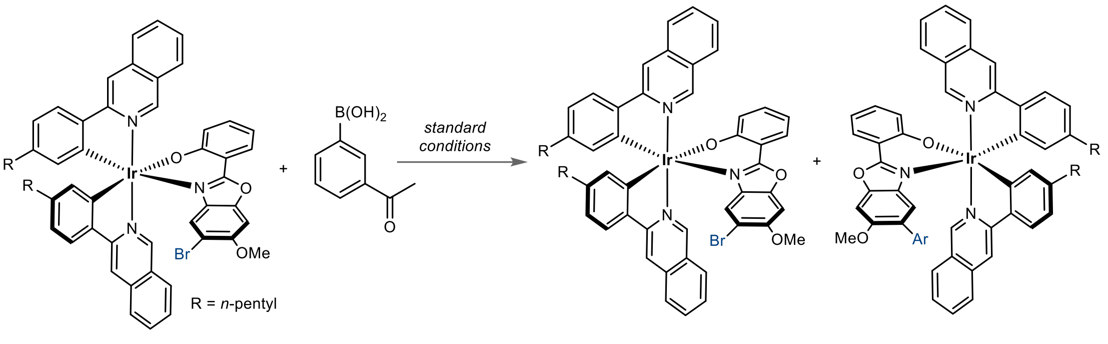

rac-**28** **2d** Λ-**28** Δ-**30**

According to the general procedure, the reaction was carried out at 30 ºC for 3 days to give the recovered Λ-**28** as orange solid (22.8 mg, 43% yield) and Δ-**30** as orange solid (26.3 mg, 48% yield).

Purification conditions: petroleum ether/EtOAc = 10:1 to 2:1.

R_f (Λ-**28**) = 0.6 in petroleum ether/EtOAc (2:1).

R_f (Δ-**30**) = 0.4 in petroleum ether/EtOAc (2:1).

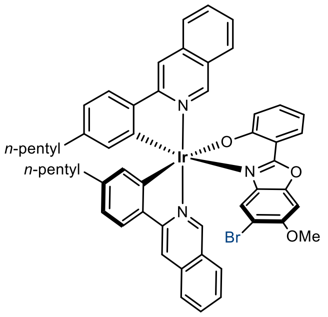

Enantiomeric excess established by HPLC analysis using a Chiralpak IK-3 column, ee = 92% (HPLC: IK-3, 254 nm, *n*-hexane/isopropanol = 80:20, flow rate 1.0 mL/min, 40 ºC, t_r (major) = 8.9 min, t_r (minor) = 10.1 min.)

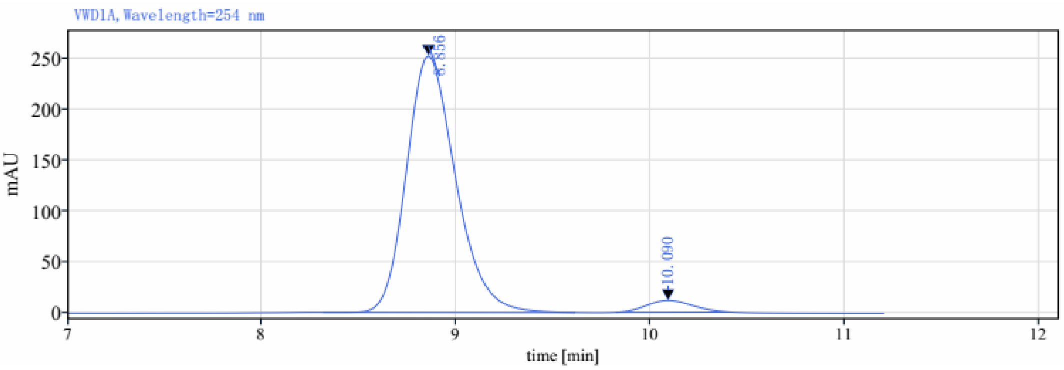

Rettime [min]	Type	Width [min]	Area	Height	Area%
8.856	MM m	1.30	4447.36	251.83	95.93
10.090	MM m	0.59	188.90	11.47	4.07

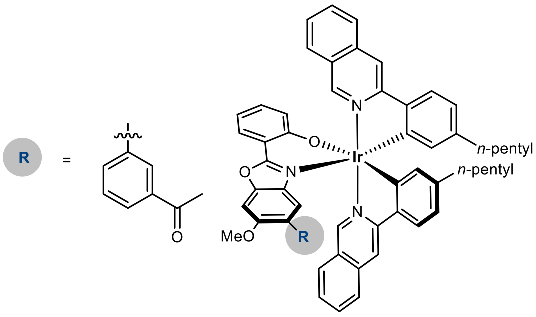

Analytical data of Δ-**30:**

^{1}H NMR (500 MHz, $CDCl_3$) δ 9.70 – 9.62 (m, 1H), 8.86 (s, 1H), 8.16 (s, 1H), 8.07 (d, *J* = 4.5 Hz, 1H), 7.96 – 7.91 (m, 1H), 7.90 – 7.83 (m, 2H), 7.83 – 7.76 (m, 3H), 7.66 – 7.53 (m, 5H), 7.47 – 7.33 (m, 3H), 7.19 (d, *J* = 7.5 Hz, 1H), 7.16 – 7.10 (m, 1H), 7.07 (s, 1H), 6.81 – 6.74 (m, 1H), 6.69 (d, *J* = 8.0 Hz, 1H), 6.51 (d, *J* = 8.0 Hz, 1H), 6.43 (t, *J* = 7.5 Hz, 1H), 6.25 – 6.20 (m, 1H), 6.10 (s, 1H), 5.92 (s, 1H), 3.77 (s, 3H), 2.63 (s, 3H), 2.19 (t, *J* = 7.5 Hz, 2H), 2.05 – 1.89 (m, 2H), 1.34 – 1.23 (m, 2H), 1.14 – 0.99 (m, 4H), 0.94 – 0.77 (m, 6H), 0.72 (t, *J* = 7.0 Hz, 3H), 0.60 (t, *J* = 7.5 Hz, 3H).

^{13}C NMR (126 MHz, $CDCl_3$) δ 198.2, 169.3, 162.1, 161.8, 160.0, 154.7, 152.14, 152.07, 150.0, 149.3, 146.1, 143.3, 143.0, 142.6, 141.8, 138.2, 136.6, 136.56, 136.5, 135.0, 134.9, 134.0, 133.3, 132.1, 131.7, 131.6, 130.1, 128.4, 128.2, 128.1, 127.5, 127.4, 127.0, 126.9, 126.55, 126.45, 126.3, 125.8, 123.3, 122.7, 122.0, 121.9, 120.7, 113.7, 113.3, 111.2, 93.7, 56.3, 35.9, 35.7, 31.5, 31.4, 30.4, 30.2, 26.9, 22.6, 22.4, 14.12, 14.07.

HRMS (ESI) for $C_{62}H_{57}IrN_3O_4$ $[M+H]^+$ calcd.1100.3973, found 1100.3967

Enantiomeric excess established by HPLC analysis using a Chiralpak IK-3 column, ee = 90% (HPLC: IK-3, 254 nm, *n*-hexane/isopropanol = 80:20, flow rate 1.0 mL/min, 40 ^{o}C, t_r (major) = 16.4 min, t_r (minor) = 20.7 min.)

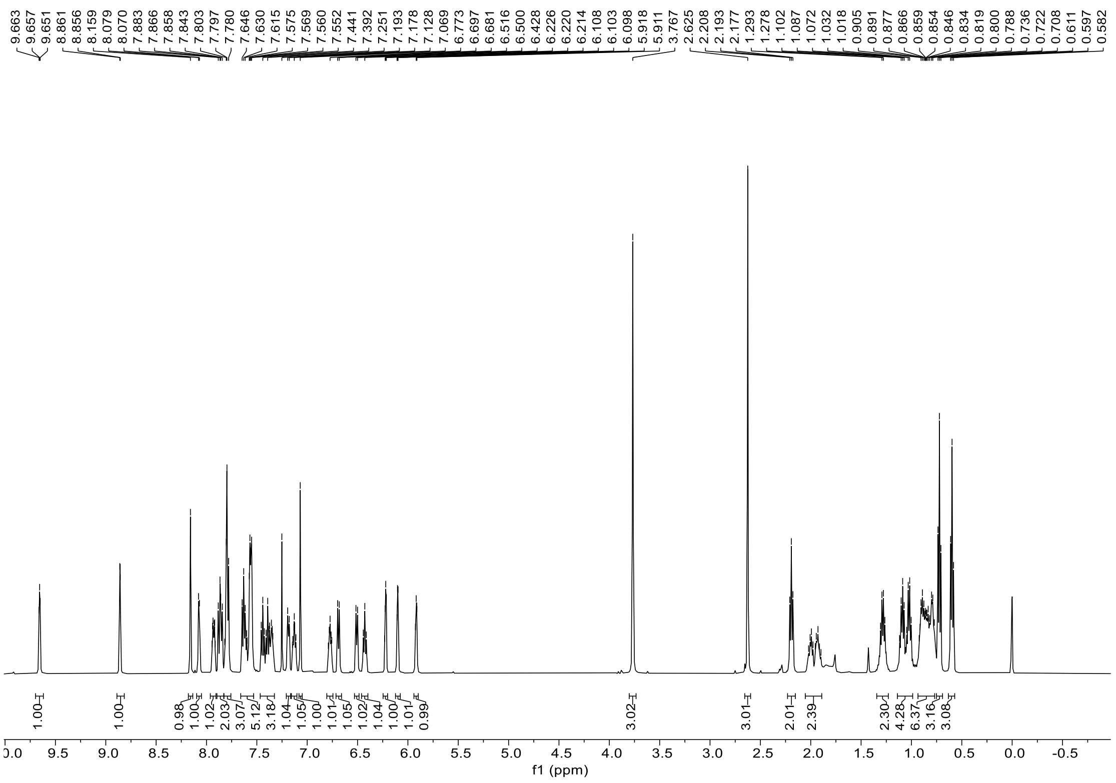

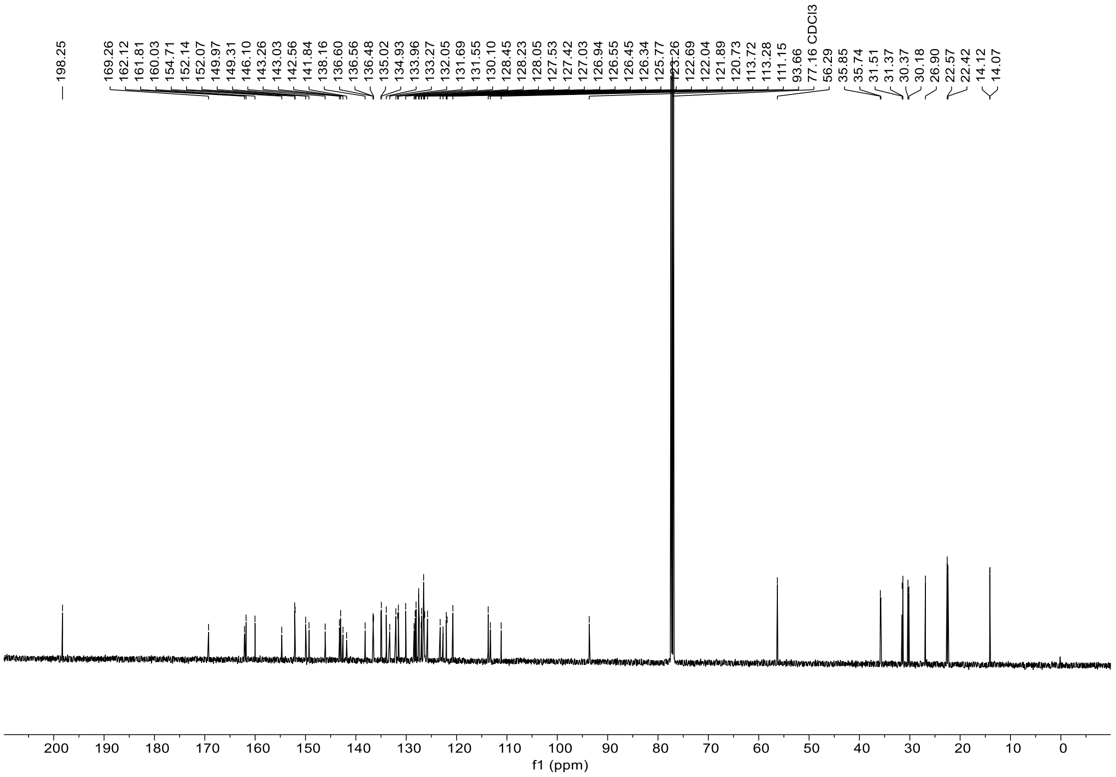

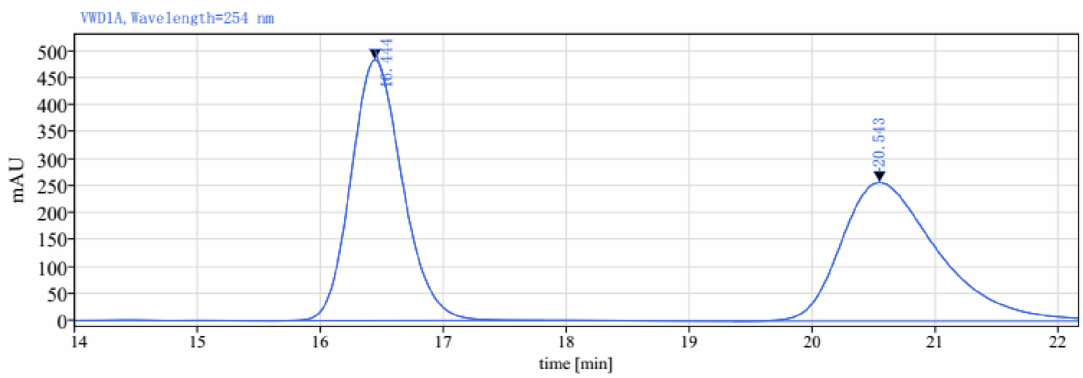

Rettime [min]	Type	Width [min]	Area	Height	Area%
16.444	BB	2.25	14011.84	482.98	49.80
20.543	BBA	5.17	14126.44	256.72	50.20

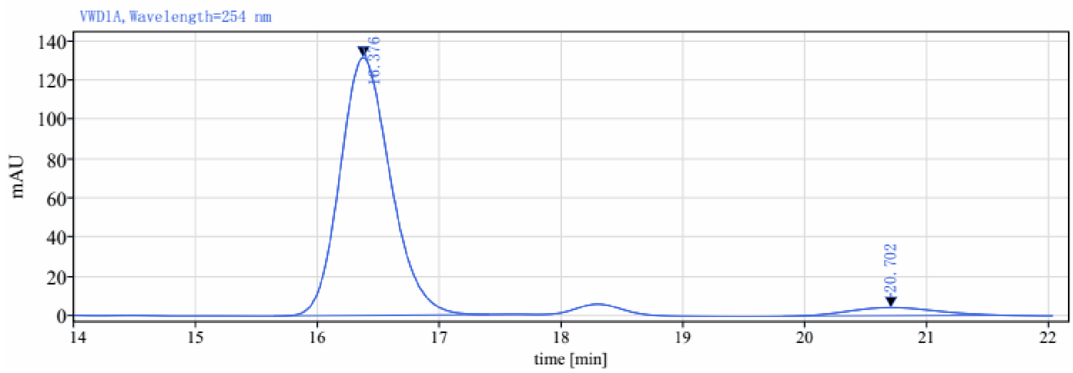

Rettime [min]	Type	Width [min]	Area	Height	Area%
16.376	BB	1.76	3776.85	131.42	94.91
20.702	MM m	1.80	202.61	4.04	5.09

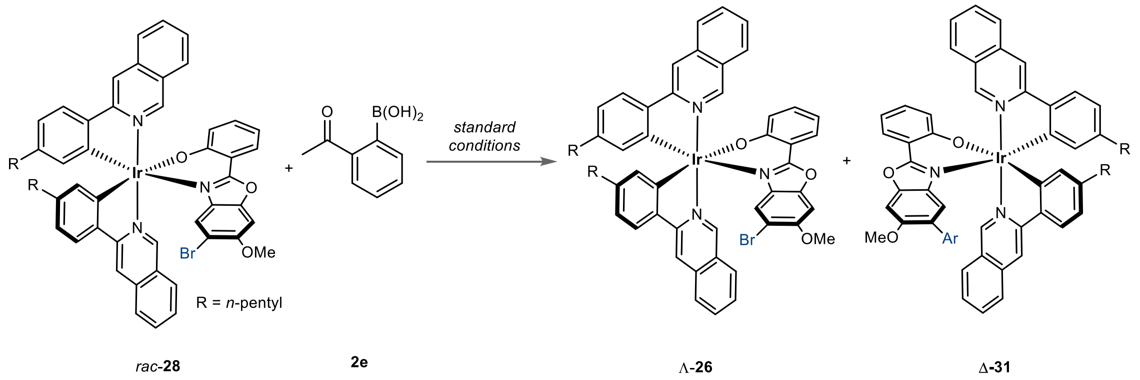

According to the general procedure, the reaction was carried out at 40 ºC for 1 days to give the

recovered Λ-**28** as orange solid (24.6 mg, 45% yield) and Δ-**31** as orange solid (23.5 mg, 43% yield).

Purification conditions: petroleum ether/EtOAc = 10:1 to 2:1.

R_f (Λ-**28**) = 0.6 in petroleum ether/EtOAc (2:1).

R_f (Δ-**31**) = 0.5 in petroleum ether/EtOAc (2:1).

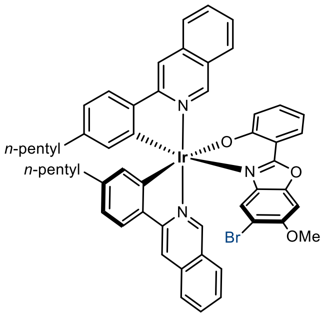

Enantiomeric excess established by HPLC analysis using a Chiralpak IK-3 column, ee = 87% (HPLC: IK-3, 254 nm, *n*-hexane/isopropanol = 80:20, flow rate 1.0 mL/min, 40 °C, t_r (major) = 8.8 min, t_r (minor) = 10.0 min.)

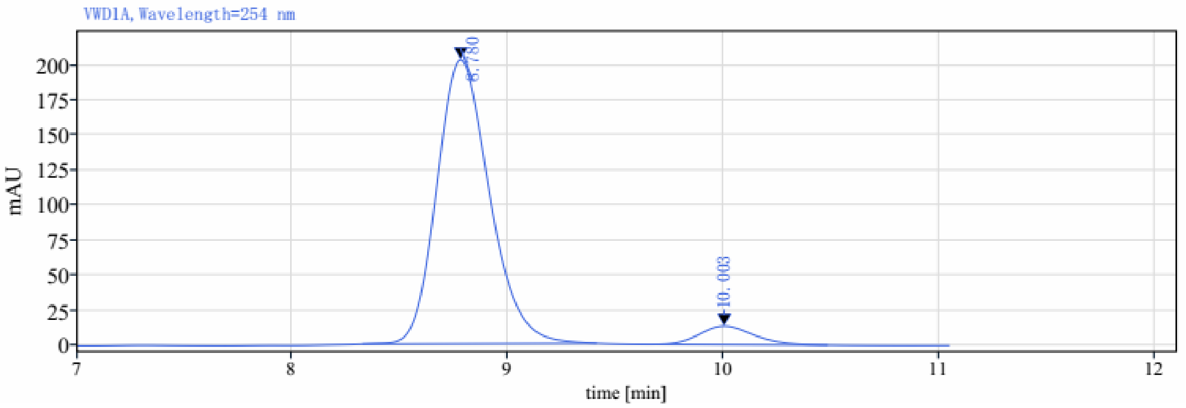

Rettime [min]	Type	Width [min]	Area	Height	Area%
8.780	MM m	1.09	3436.39	202.56	93.74
10.003	MM m	0.80	229.57	13.18	6.26

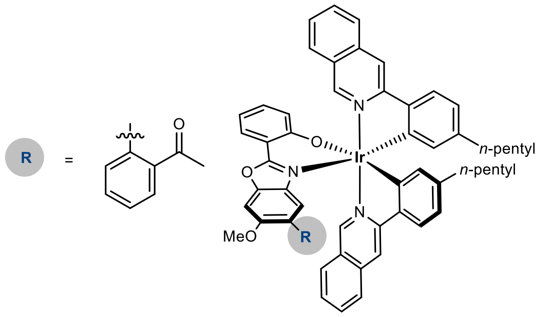

Analytical data of Δ-**31:**

^{1}H NMR (500 MHz, Tetrachloroethane-d_2, 110 °C) δ 9.66 (s, 1H), 9.00 (s, 1H), 8.14 (s, 1H), 8.04 (s, 1H), 7.93 (dd, J = 8.0, 2.0 Hz, 1H), 7.88 (t, J = 8.5 Hz, 2H), 7.77 (d, J = 8.0 Hz, 1H), 7.68 – 7.57 (m, 5H), 7.54 – 7.34 (m, 5H), 7.23 – 7.17 (m, 1H), 7.01 (s, 1H), 6.88 – 6.78 (m, 3H), 6.54 – 6.43 (m, 2H), 6.25 (s, 1H), 6.15 (s, 1H), 6.00 (s, 1H), 3.68 (s, 3H), 2.36 – 2.26 (m, 2H), 2.01 – 1.97 (m, 3H), 1.37 (p, J = 7.5 Hz, 2H), 1.18 – 0.85 (m, 12H), 0.77 (t, J = 7.0 Hz, 3H), 0.66 (t, J = 7.0 Hz, 3H).

^{13}C NMR (126 MHz, $C_2D_2Cl_4$, 110 °C) δ 200.0, 169.7, 162.1, 161.7, 160.3, 154.2, 152.6, 152.3, 149.9, 148.9, 145.7, 142.8, 142.4, 142.3, 142.1, 139.8, 136.4, 136.3, 134.8, 133.6, 133.0, 132.0, 131.8, 131.3, 131.2, 130.3, 128.4, 128.0, 127.8, 127.5, 127.2, 127.0, 126.7, 126.2, 126.12, 126.1, 126.0, 125.2, 122.9, 122.6, 121.5, 120.2, 113.3, 113.2, 112.6, 111.2, 93.4, 55.7, 35.3, 35.1, 31.1, 29.4, 29.2, 28.2, 22.0, 21.8, 13.5, 13.4.

HRMS (ESI) for $C_{62}H_{57}IrN_3O_4$ $[M+H]^+$ calcd.1100.3973, found 1100.3978

Enantiomeric excess established by HPLC analysis using a Chiralpak IK-3 column, ee = 90% (HPLC: IK-3, 254 nm, *n*-hexane/isopropanol = 80:20, flow rate 1.0 mL/min, 40 ^{o}C, t_r (major) = 16.2 min, t_r (minor) = 13.7 min.)

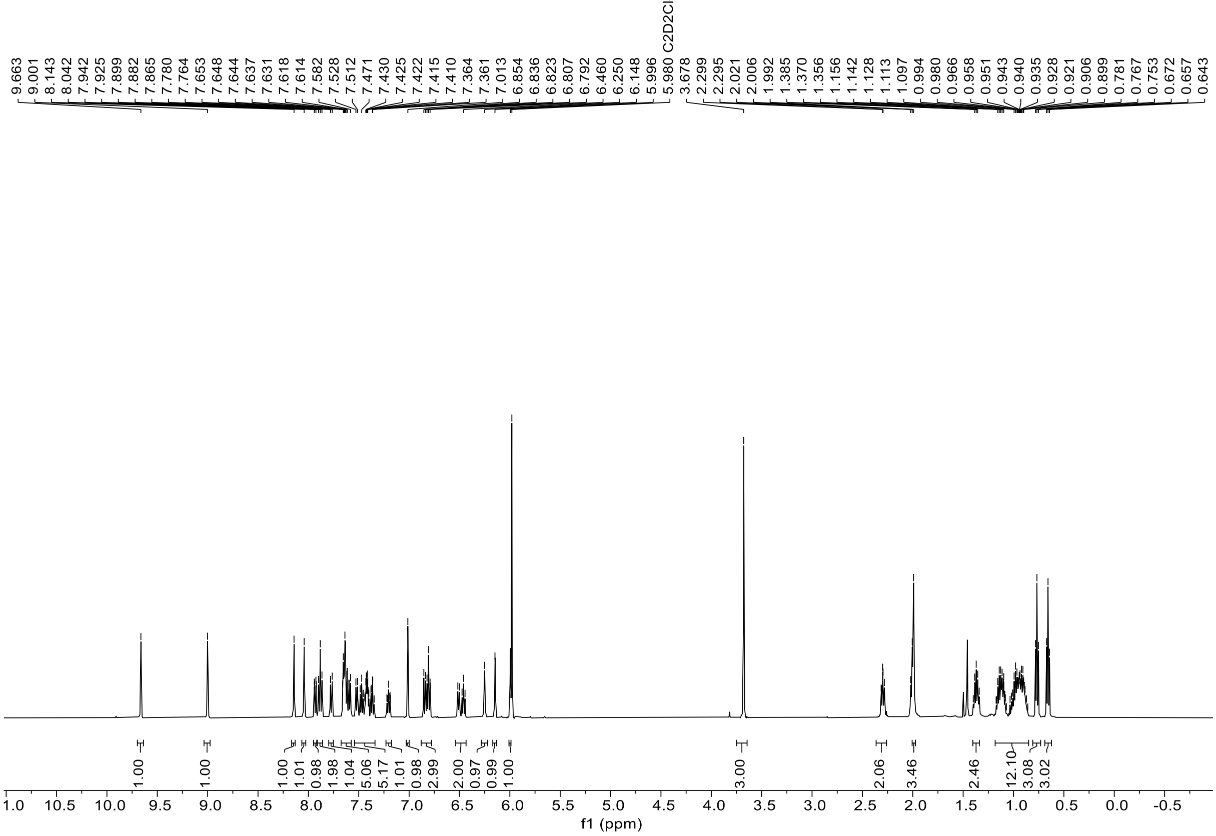
9.663
9.001
8.143
8.042
7.942
7.925
7.899
7.882
7.865
7.780
7.764
7.653
7.648
7.644
7.637
7.631
7.618
7.614
7.582
7.528
7.512
7.471
7.430
7.425
7.422
7.415
7.410
7.364
7.361
7.013
6.854
6.836
6.823
6.807
6.792
6.460
6.250
6.148
5.996
5.980 C2D2Cl4
3.678
2.299
2.295
2.021
2.006
1.992
1.385
1.370
1.356
1.156
1.142
1.128
1.113
1.097
0.994
0.980
0.966
0.958
0.951
0.943
0.940
0.935
0.928
0.921
0.906
0.899
0.781
0.767
0.753
0.672
0.657
0.643
1.00
1.00
1.00
1.01
0.98
1.98
1.04
5.06
5.17
1.01
0.98
2.99
2.00
0.97
0.99
1.00
3.00
2.06
3.46
2.46
12.10
3.08
3.02
1.0 10.5 10.0 9.5 9.0 8.5 8.0 7.5 7.0 6.5 6.0 5.5 5.0 4.5 4.0 3.5 3.0 2.5 2.0 1.5 1.0 0.5 0.0 -0.5
f1 (ppm)

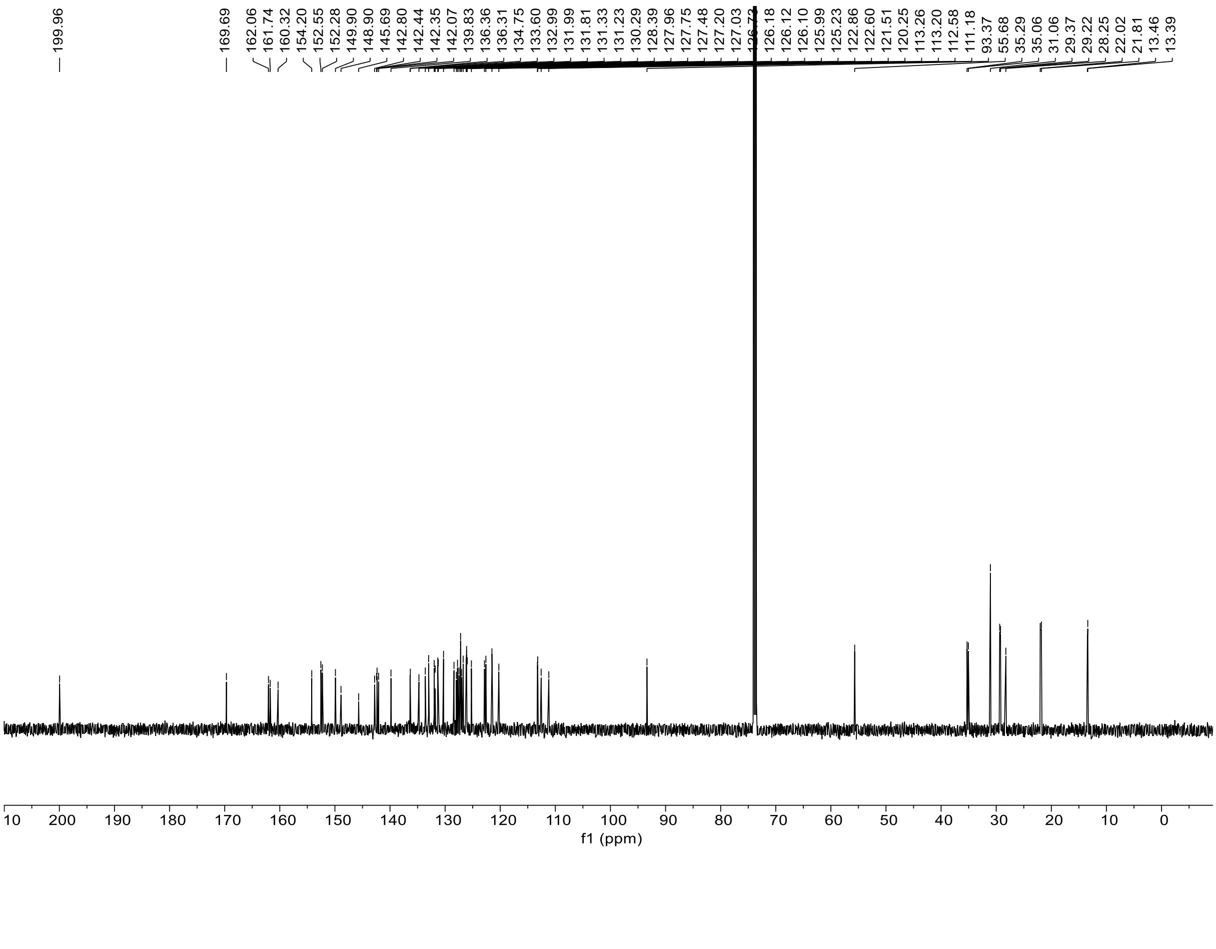
199.96
169.69
162.06
161.74
160.32
154.20
152.55
152.28
149.90
148.90
145.69
142.80
142.44
142.35
142.07
139.83
136.36
136.31
134.75
133.60
132.99
131.99
131.81
131.33
131.23
130.29
128.39
127.96
127.75
127.48
127.20
127.03
126.18
126.12
126.10
125.99
125.23
122.86
122.60
121.51
120.25
113.26
113.20
112.58
111.18
93.37
55.68
35.29
35.06
31.06
29.37
29.22
28.25
22.02
21.81
13.46
13.39
10 200 190 180 170 160 150 140 130 120 110 100 90 80 70 60 50 40 30 20 10 0
f1 (ppm)

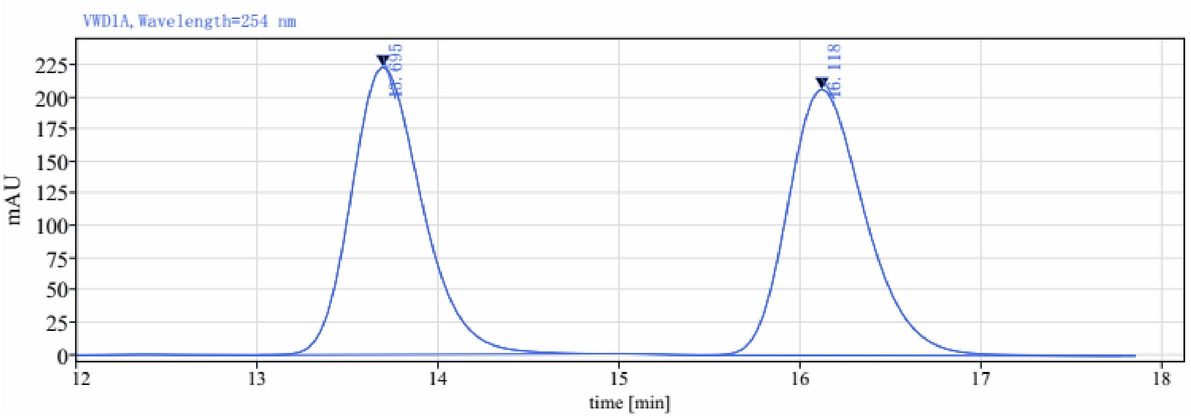

Rettime [min]	Type	Width [min]	Area	Height	Area%
13.695	BB	1.88	6083.04	223.25	49.76
16.118	BBA	2.29	6142.49	206.50	50.24

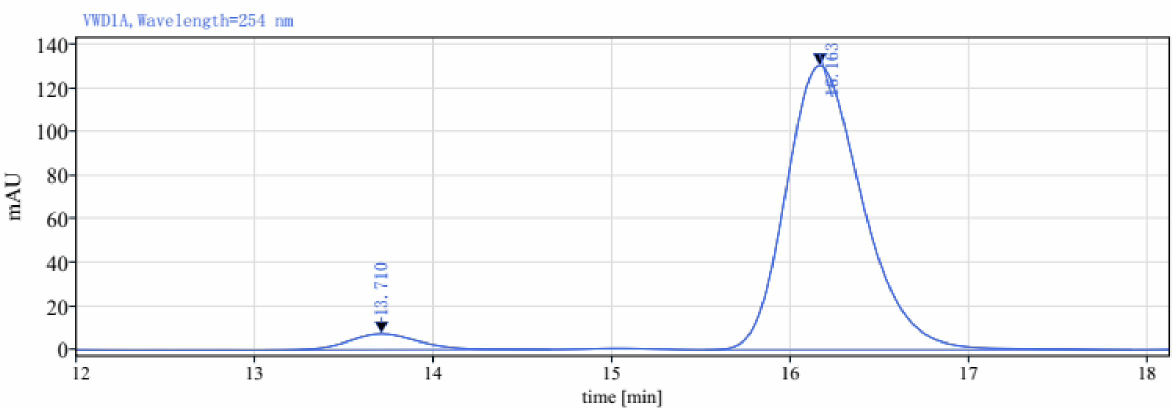

Rettime [min]	Type	Width [min]	Area	Height	Area%
13.710	BB	1.60	203.86	7.45	4.98
16.163	BB	2.39	3893.65	130.63	95.02

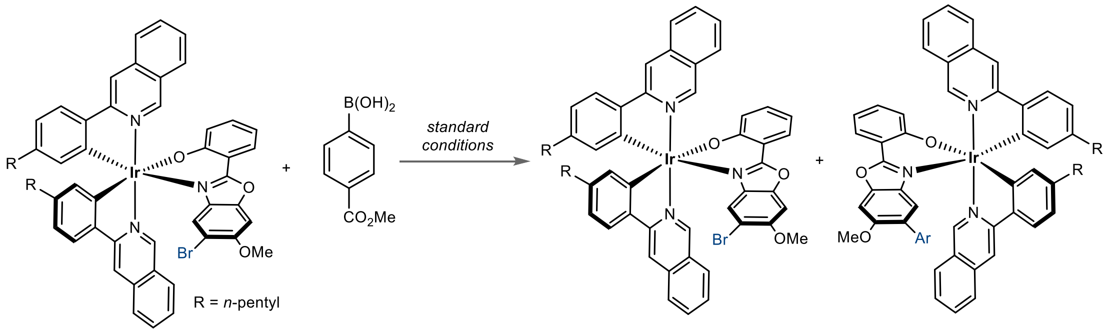

rac-**28** **2e** Λ-**28** Δ-**32**

According to the general procedure, the reaction was carried out at 30 ^{o}C for 4 days to give the recovered Λ-**28** as orange solid (21.7 mg, 41% yield) and Δ-**32** as orange solid (21.8 mg, 39% yield).

Purification conditions: petroleum ether/EtOAc = 10:1 to 2:1.

R_f (Λ-**28**) = 0.6 in petroleum ether/EtOAc (2:1).

R_f (Δ-**32**) = 0.5 in petroleum ether/EtOAc (2:1).

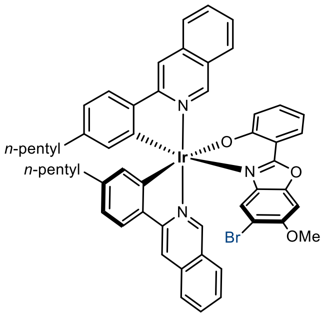

Enantiomeric excess established by HPLC analysis using a Chiralpak IK-3 column, ee = 91% (HPLC: IK-3, 254 nm, *n*-hexane/isopropanol = 80:20, flow rate 1.0 mL/min, 40 ^{o}C, t_r (major) = 8.8 min, t_r (minor) = 10.0 min.)

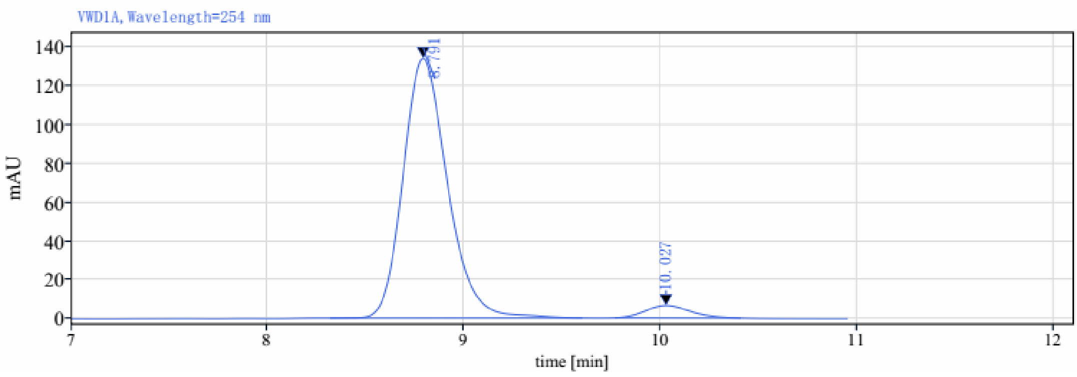

Rettime [min]	Type	Width [min]	Area	Height	Area%
8.791	MM m	1.28	2099.51	133.87	95.36
10.027	MM m	0.64	102.11	6.40	4.64

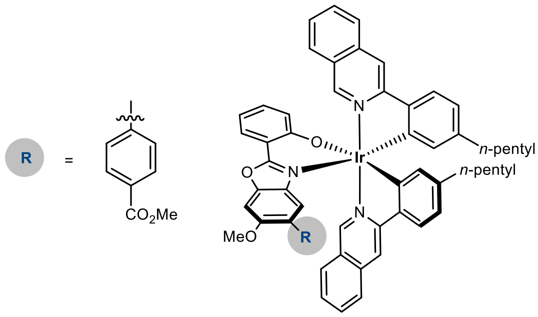

Analytical data of Δ-**32:**

^{1}H NMR (500 MHz, CDCl$_3$) δ 9.66 (d, *J* = 2.0 Hz, 1H), 8.87 (s, 1H), 8.07 (d, *J* = 4.0 Hz, 2H), 7.96 – 7.91 (m, 3H), 7.86 – 7.74 (m, 3H), 7.63 – 7.54 (m, 5H), 7.43 (t, *J* = 7.5 Hz, 1H), 7.35 (td, *J* = 7.5, 2.5 Hz, 1H), 7.17 (dd, *J* = 8.5, 1.5 Hz, 2H), 7.12 (ddd, *J* = 8.5, 7.0, 2.0 Hz, 1H), 7.06 (s, 1H), 6.76 (dd, *J* = 8.5, 2.5 Hz, 1H), 6.71 – 6.64 (m, 2H), 6.42 (t, *J* = 7.5 Hz, 1H), 6.36 – 6.32 (m, 1H), 6.13 (d, *J* = 2.0 Hz, 1H), 5.89 (d, *J* = 2.5 Hz, 1H), 3.93 (s, 3H), 3.77 (d, *J* = 2.0 Hz, 3H), 2.18 (t, *J* = 7.5 Hz, 2H), 2.11 – 1.99 (m, 2H), 1.31 – 1.24 (m, 2H), 1.12 – 1.05 (m, 2H), 1.03 – 0.83 (m, 8H), 0.72 (t, *J* = 7.0 Hz, 3H), 0.61 (t, *J* = 7.0 Hz, 3H).

^{13}C NMR (126 MHz, CDCl$_3$) δ 169.2, 167.3, 162.1, 161.8, 160.1, 154.8, 152.2, 152.1, 150.1, 149.2, 146.0, 143.4, 143.0, 142.43, 142.37, 141.8, 136.53, 136.49, 135.1, 134.0, 133.3, 132.0, 131.7, 131.6, 130.1, 129.0, 128.44, 128.36, 128.2, 127.54, 127.51, 127.4, 126.8, 126.5, 126.4, 126.3, 125.7, 123.2, 122.7, 122.0, 120.7, 113.8, 113.7, 113.2, 111.2, 93.8, 56.3, 52.2, 35.84, 35.77, 31.5, 31.4, 30.3, 30.2, 22.6, 22.4, 14.13, 14.08.

HRMS (ESI) for $C_{62}H_{57}IrN_3O_5$ $[M+H]^+$ calcd.1116.3922, found 1116.3923

Enantiomeric excess established by HPLC analysis using a Chiralpak IK-3 column, ee = 95% (HPLC: IK-3, 254 nm, *n*-hexane/isopropanol = 90:10, flow rate 1.0 mL/min, 40 ^{o}C, t_r (major) = 33.5 min, t_r (minor) = 33.7 min.)

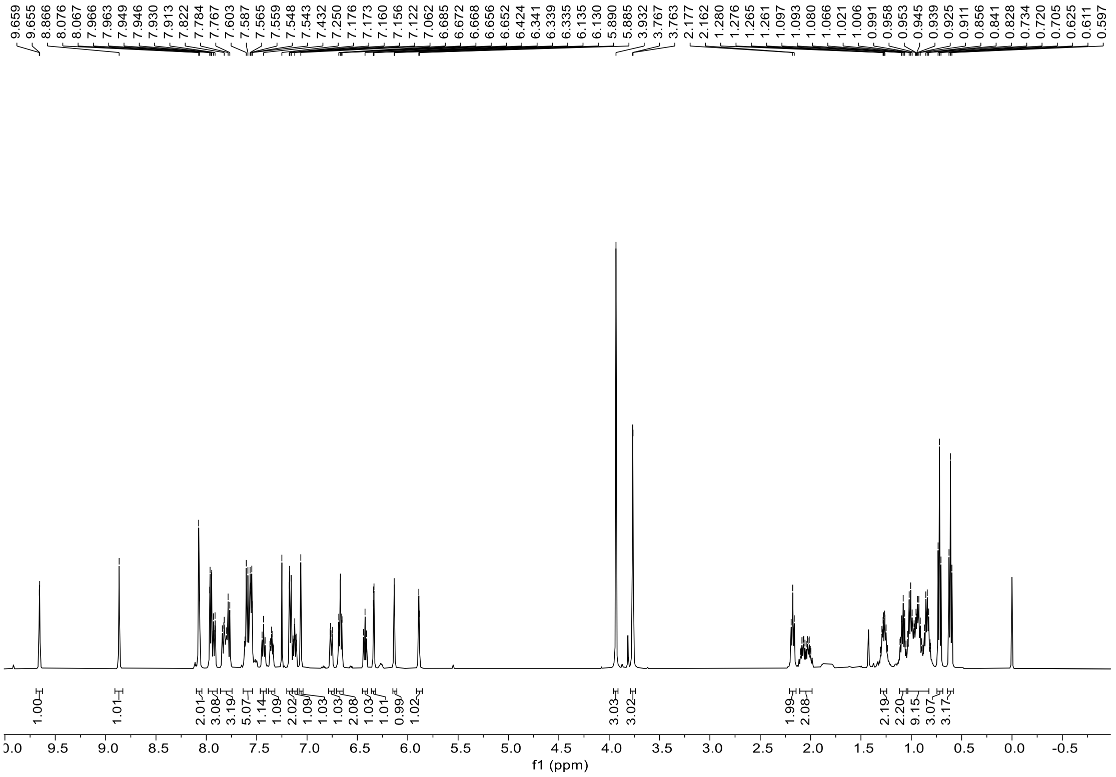

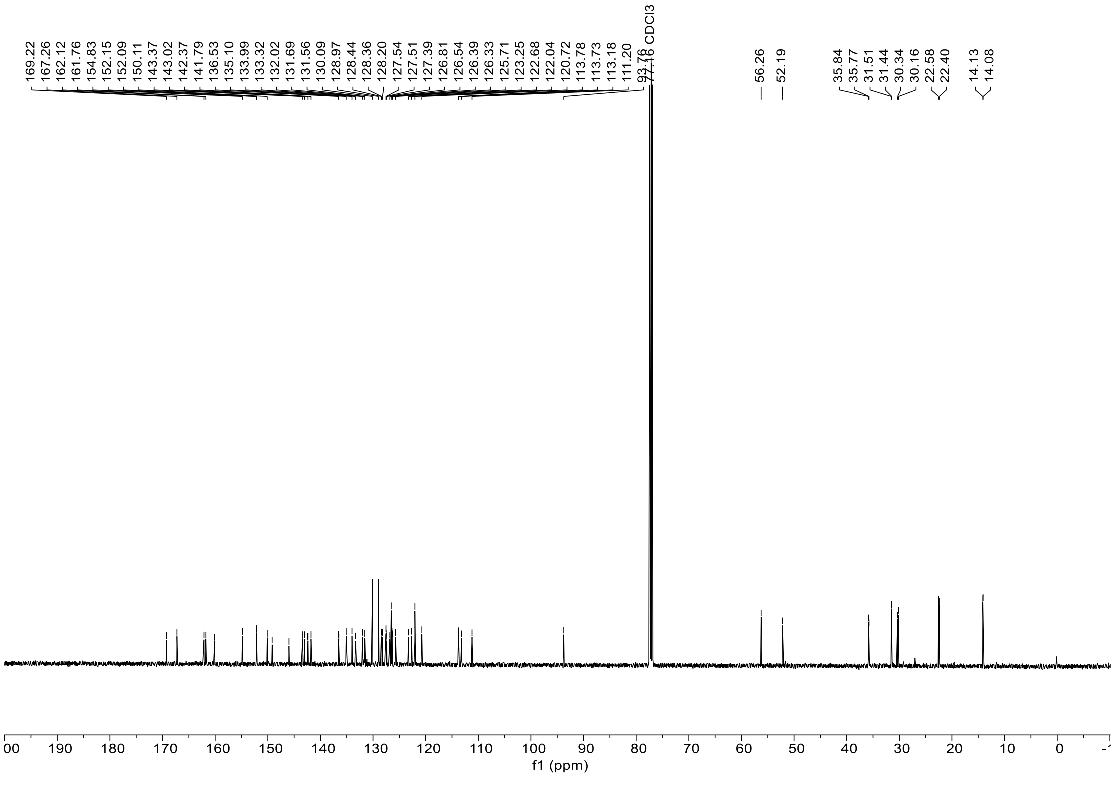

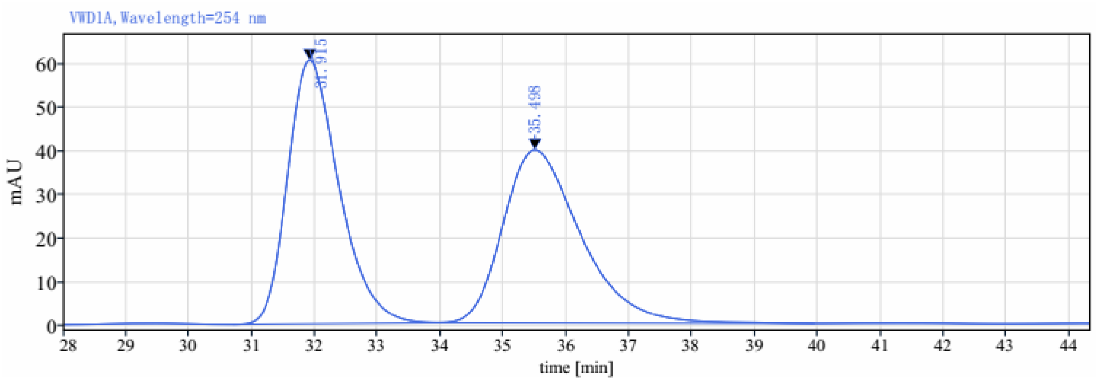

Rettime [min]	Type	Width [min]	Area	Height	Area%
31.915	BB	3.26	3419.08	60.30	50.17
35.498	BB	5.99	3396.44	39.56	49.83

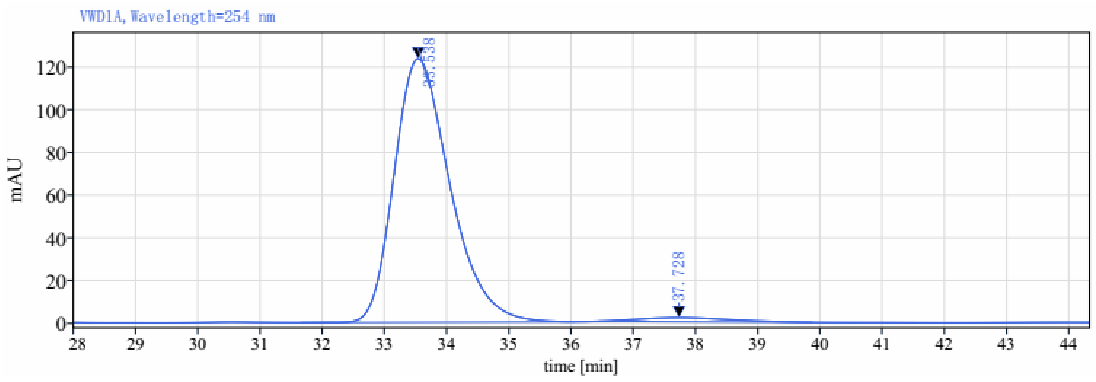

Rettime [min]	Type	Width [min]	Area	Height	Area%
33.538	BB	4.40	7724.03	124.05	97.67
37.728	MM m	3.43	184.35	1.84	2.33

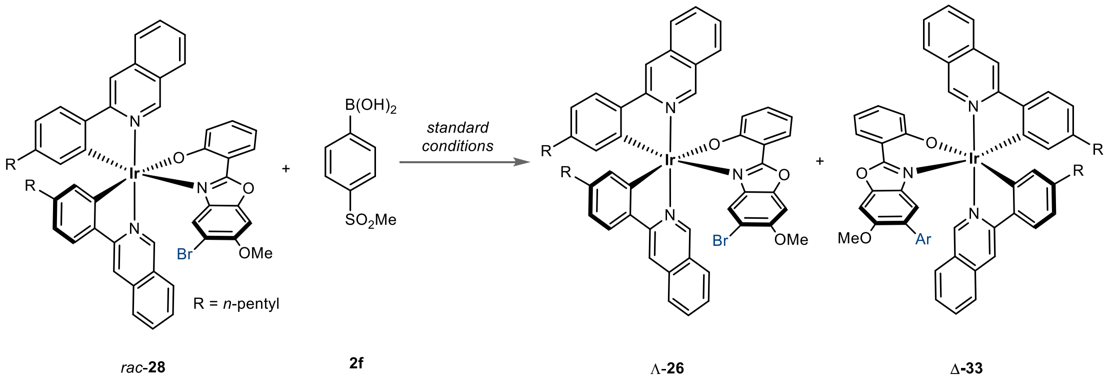

According to the general procedure, the reaction was carried out at 30 °C for 3 days to give the

recovered Λ-**28** as orange solid (22.2 mg, 42% yield) and Δ-**33** as orange solid (26.6 mg, 47% yield).

Purification conditions: petroleum ether/EtOAc = 10:1 to 1:1.

R_f (Λ-**28**) = 0.6 in petroleum ether/EtOAc (2:1).

R_f (Δ-**33**) = 0.2 in petroleum ether/EtOAc (2:1).

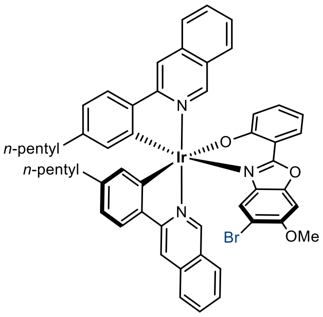

Enantiomeric excess established by HPLC analysis using a Chiralpak IK-3 column, ee = 97% (HPLC: IK-3, 254 nm, *n*-hexane/isopropanol = 80:20, flow rate 1.0 mL/min, 40 ^oC, t_r (major) = 8.9 min, t_r (minor) = 10.4 min.)

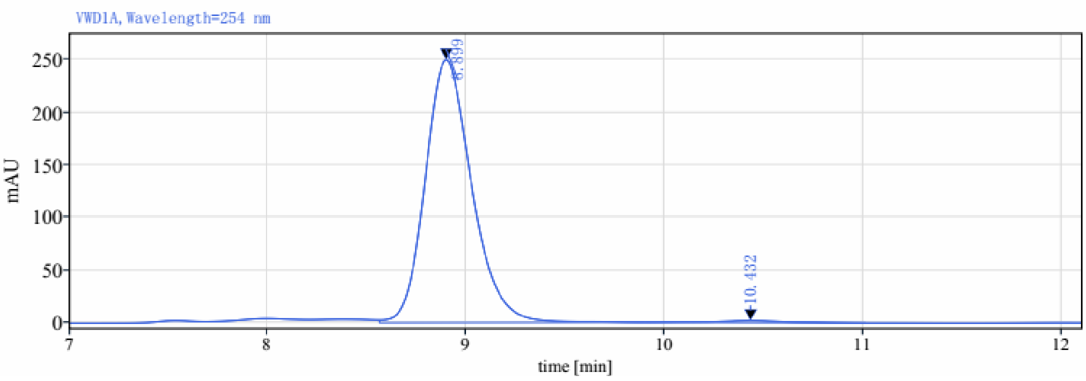

Rettime [min]	Type	Width [min]	Area	Height	Area%
8.899	VB	1.34	4071.82	249.67	98.95
10.432	BB	1.11	43.13	1.88	1.05

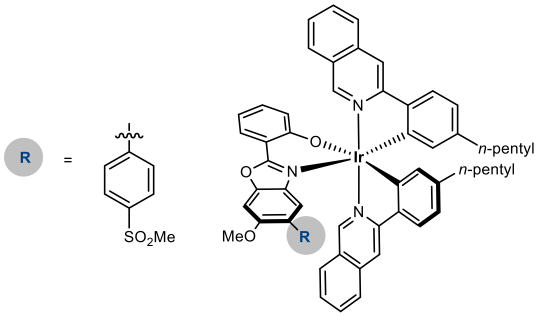

Analytical data of Δ-**33:**

^{1}H NMR (500 MHz, Chloroform-*d*) δ 9.66 (s, 1H), 8.85 (s, 1H), 8.08 (s, 2H), 7.91 (d, *J* = 8.0 Hz, 1H), 7.86 – 7.75 (m, 5H), 7.63 – 7.52 (m, 5H), 7.43 (d, *J* = 7.5 Hz, 1H), 7.34 (d, *J* = 7.5 Hz, 1H), 7.29 (d, *J* = 8.0 Hz, 2H), 7.15 – 7.10 (m, 1H), 7.08 (s, 1H), 6.76 (d, *J* = 8.5 Hz, 1H), 6.66 (dd, *J* = 20.0, 8.0 Hz, 2H), 6.41 (t, *J* = 7.5 Hz, 1H), 6.37 (s, 1H), 6.14 (s, 1H), 5.88 (s, 1H), 3.78 (s, 3H), 3.05 (s, 3H), 2.23 – 2.15 (m, 2H), 2.11 – 2.02 (m, 2H), 1.32 – 1.23 (m, 2H), 1.11 – 0.80 (m, 10H), 0.72 (t, *J* = 7.0 Hz, 3H), 0.59 (t, *J* = 7.0 Hz, 3H).

^{13}C NMR (126 MHz, $CDCl_3$) δ 169.6, 162.2, 161.7, 160.4, 154.7, 152.12, 152.08, 150.4, 149.2, 146.3, 143.4, 143.2, 143.1, 142.6, 141.7, 138.5, 136.53, 136.51, 135.3, 134.1, 133.4, 131.9, 131.8, 131.6, 131.0, 128.4, 128.2, 127.5, 127.45, 127.4, 126.7, 126.62, 126.58, 126.4, 126.3, 125.9, 125.7, 123.2, 122.7, 122.1, 122.0, 120.8, 113.8, 113.7, 113.2, 111.0, 93.9, 56.3, 44.7, 35.8, 35.7, 31.5, 31.3, 30.4, 30.1, 22.6, 22.4, 14.11, 14.06.

HRMS (ESI) for $C_{61}H_{57}IrN_3O_5S$ $[M+H]^+$ calcd.1136.3643, found 1136.3642

Enantiomeric excess established by HPLC analysis using a Chiralpak IM column, ee = 92% (HPLC: IM, 254 nm, *n*-hexane/isopropanol = 50:50, flow rate 1.0 mL/min, 40 ^{o}C, t_r (major) = 16.9 min, t_r (minor) = 23.4 min.)

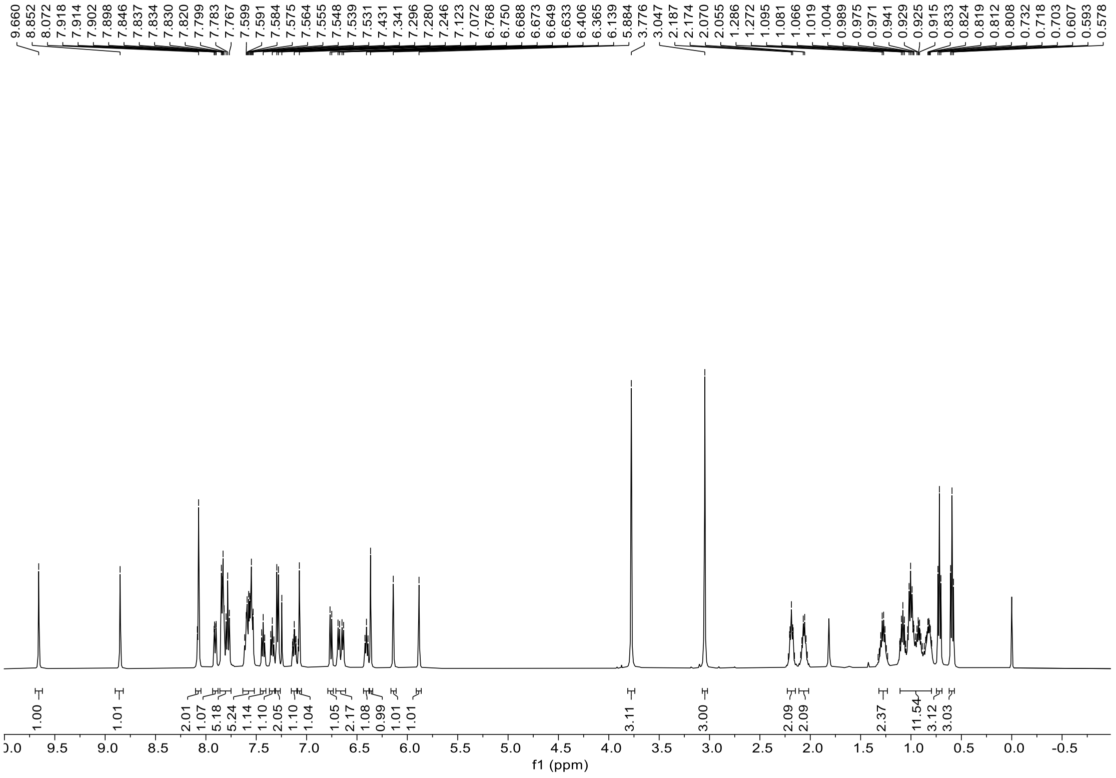

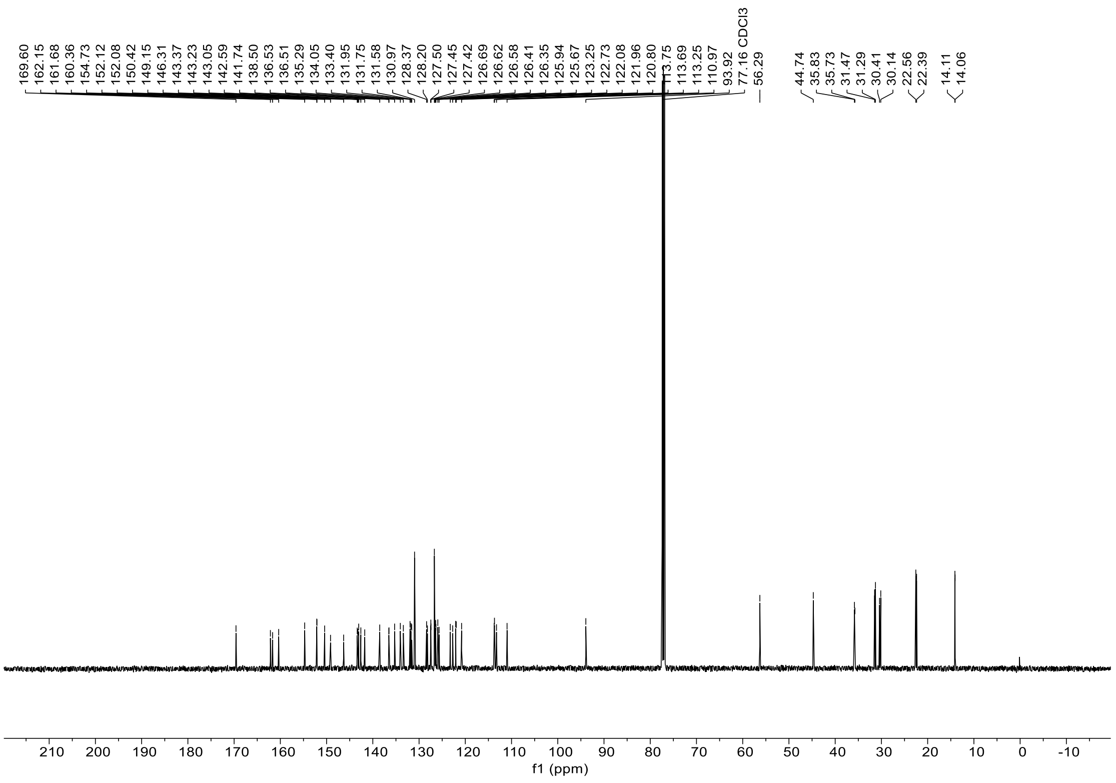

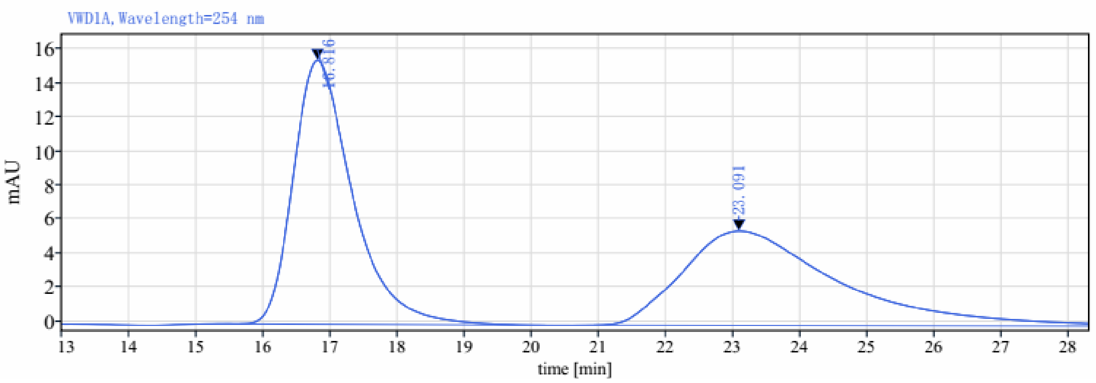

Rettime [min]	Type	Width [min]	Area	Height	Area%
16.816	BB	4.56	952.69	15.53	51.53
23.091	BBA	9.19	895.98	5.56	48.47

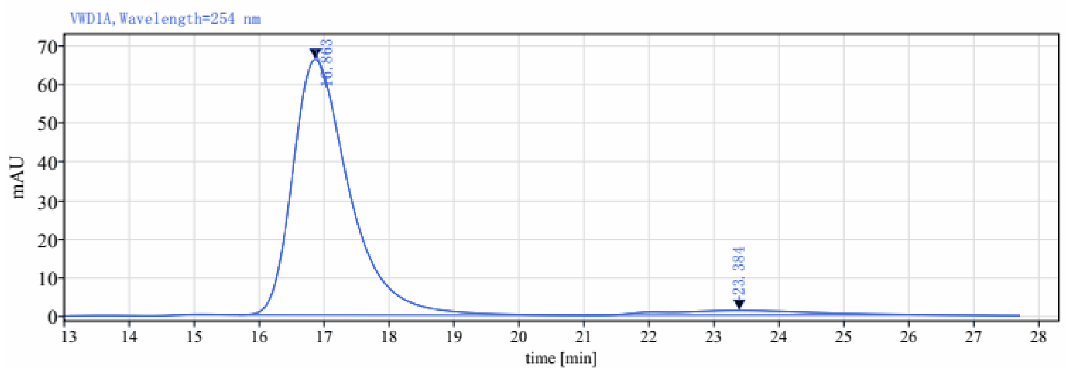

Rettime [min]	Type	Width [min]	Area	Height	Area%
16.863	BB	5.40	3983.17	65.96	95.89
23.384	MM m	5.06	170.86	1.12	4.11

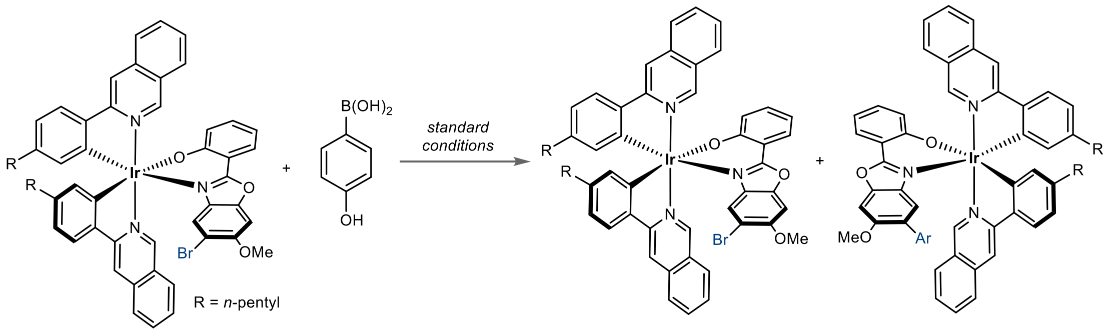

According to the general procedure, the reaction was carried out at 30 ºC for 5 days to give the recovered Λ-**28** as orange solid (21.6 mg, 41% yield) and Δ-**34** as orange solid (21.0 mg, 39% yield).

Purification conditions: petroleum ether/EtOAc = 10:1 to 1:1.

R_f (Λ-**28**) = 0.6 in petroleum ether/EtOAc (2:1).

R_f (Δ-**34**) = 0.3 in petroleum ether/EtOAc (2:1).

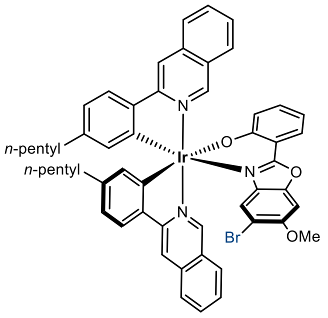

Enantiomeric excess established by HPLC analysis using a Chiralpak IK-3 column, ee = 85%

(HPLC: IK-3, 254 nm, *n*-hexane/isopropanol = 80:20, flow rate 1.0 mL/min, 40 °C, t_r (major) = 8.9 min, t_r (minor) = 10.1 min.)

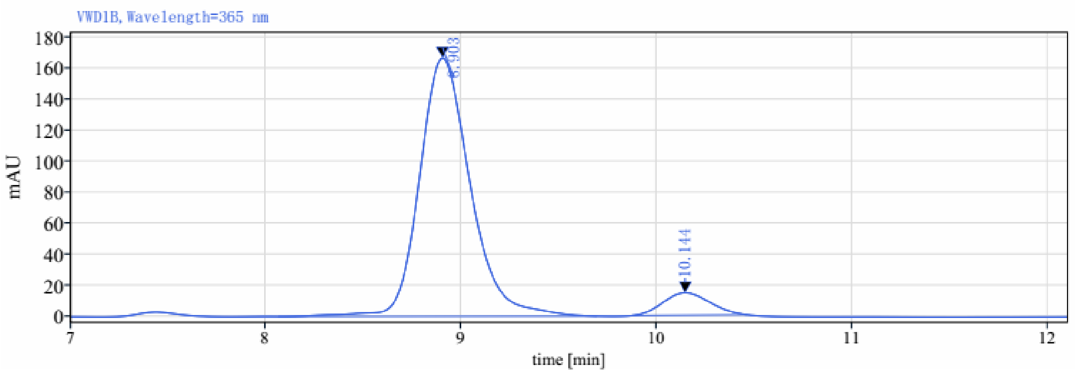

Rettime [min]	Type	Width [min]	Area	Height	Area%
8.903	MM m	1.63	3007.30	166.59	92.61
10.144	MM m	0.59	240.14	14.42	7.39

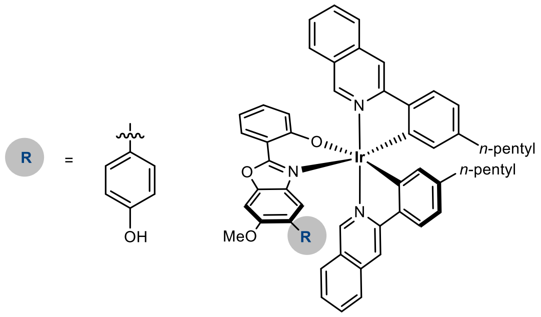

Analytical data of Δ-**34:**

^{1}H NMR (500 MHz, Chloroform-*d*) δ 9.65 (s, 1H), 8.89 (s, 1H), 8.03 (d, *J* = 5.0 Hz, 2H), 7.95 – 7.90 (m, 1H), 7.76 (d, *J* = 7.5 Hz, 2H), 7.68 (d, *J* = 8.5 Hz, 1H), 7.57 – 7.50 (m, 5H), 7.36 (d, *J* = 7.5 Hz, 1H), 7.31 (d, *J* = 7.5 Hz, 1H), 7.12 – 7.08 (m, 1H), 7.02 (s, 1H), 6.87 (d, *J* = 8.5 Hz, 2H), 6.78 (d, *J* = 8.5 Hz, 1H), 6.66 – 6.58 (m, 4H), 6.45 (t, *J* = 7.5 Hz, 1H), 6.35 (s, 1H), 6.13 (s, 1H), 6.06 (s, 1H), 5.88 (s, 1H), 3.74 (s, 3H), 2.14 (d, *J* = 8.0 Hz, 2H), 2.09 – 1.99 (m, 2H), 1.26 – 1.19 (m, 2H), 1.07 – 0.80 (m, 10H), 0.68 (t, *J* = 7.0 Hz, 3H), 0.58 (t, *J* = 7.5 Hz, 3H).

^{13}C NMR (126 MHz, $CDCl_3$) δ 168.6, 162.2, 161.8, 159.9, 155.2, 154.9, 152.3, 149.3, 149.1, 145.6, 143.3, 143.0, 142.4, 141.9, 136.5, 134.9, 133.9, 133.4, 132.1, 131.6, 131.5, 131.2, 129.3, 128.6, 128.5, 128.2, 128.0, 127.8, 127.6, 127.5, 127.4, 126.52, 126.45, 126.3, 126.27, 125.2, 123.2, 122.7, 122.1, 122.06, 120.3, 114.7, 114.2, 113.7, 113.1, 111.8, 93.6, 56.2, 35.83, 35.81, 31.5, 31.4, 30.3, 30.2, 22.5, 22.4, 14.1.

HRMS (ESI) for $C_{60}H_{55}IrN_3O_4$ $[M+H]^+$ calcd.1074.3817, found 1074.3827

Enantiomeric excess established by HPLC analysis using a Chiralpak IK-3 column, ee = 85% (HPLC: IK-3, 254 nm, *n*-hexane/isopropanol = 80:20, flow rate 1.0 mL/min, 40 °C, t_r (major) = 16.0 min, t_r (minor) = 18.7 min.)

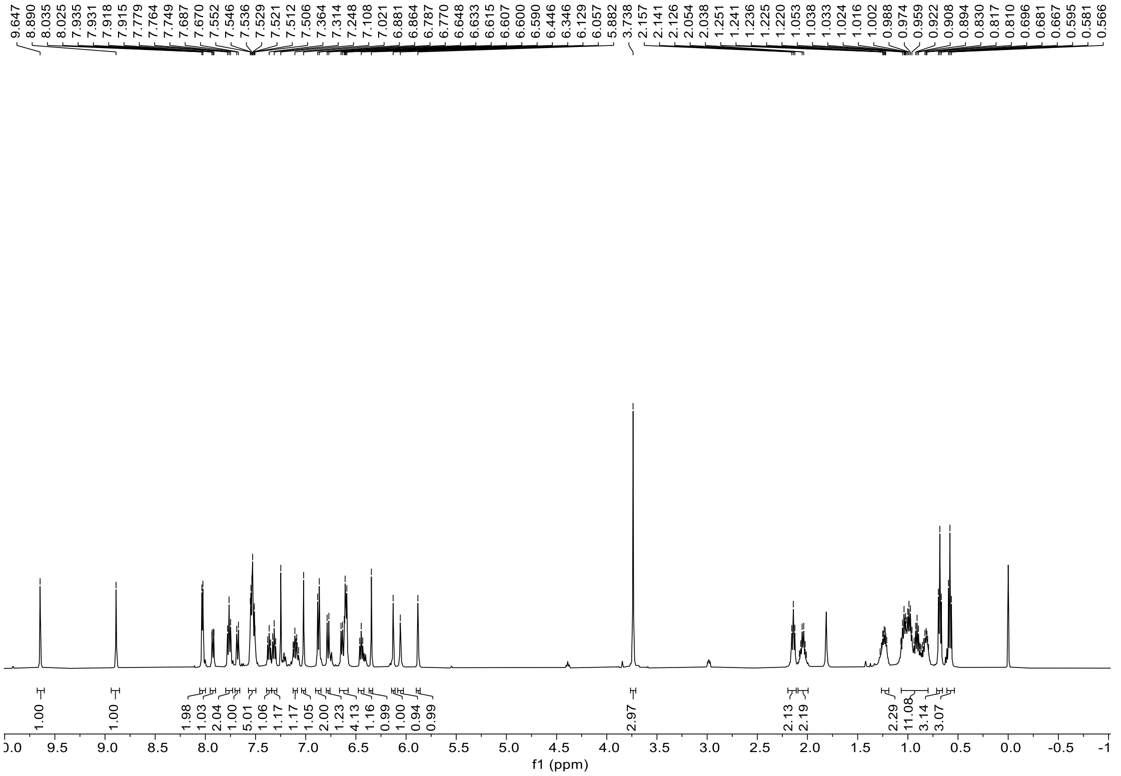

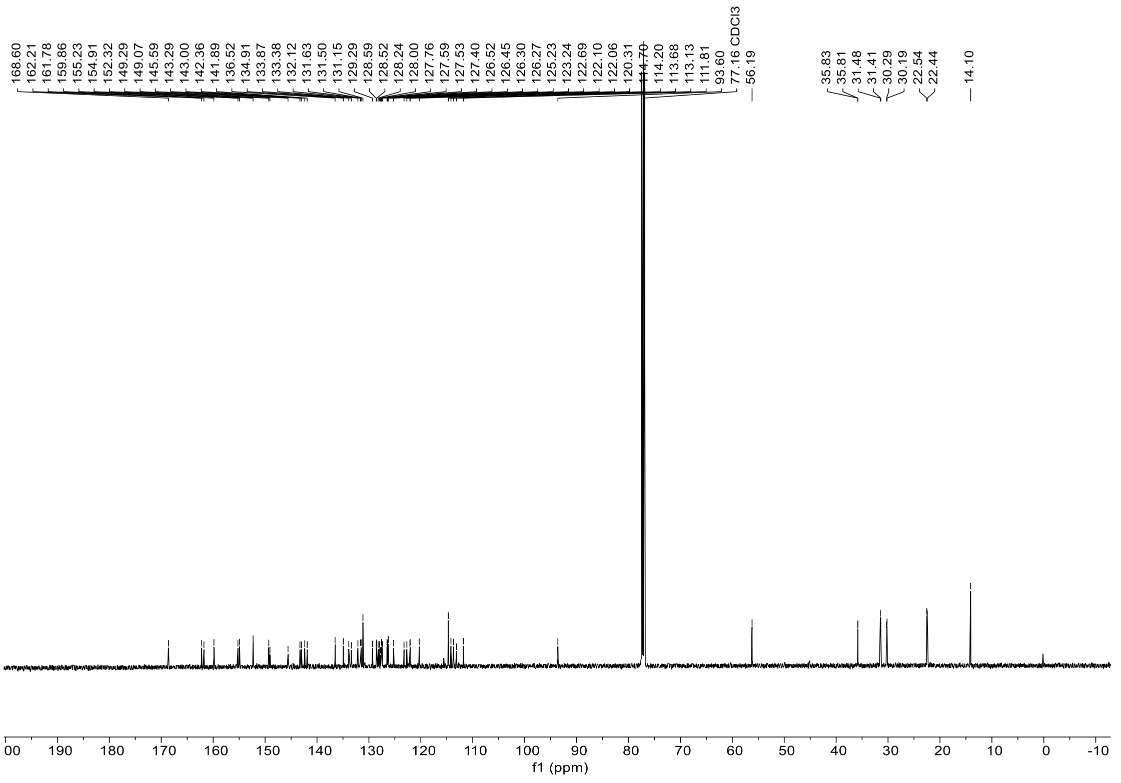

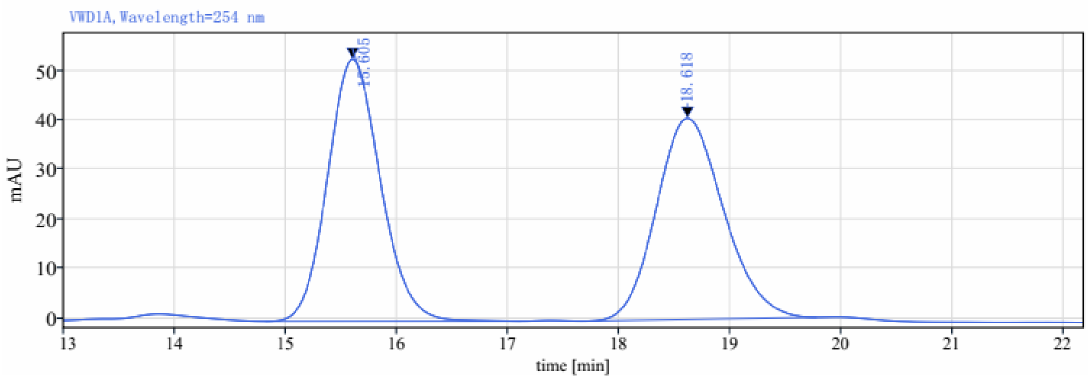

Rettime [min]	Type	Width [min]	Area	Height	Area%
15.605	BB	2.30	1750.51	53.02	50.61
18.618	BB	2.09	1708.25	40.67	49.39

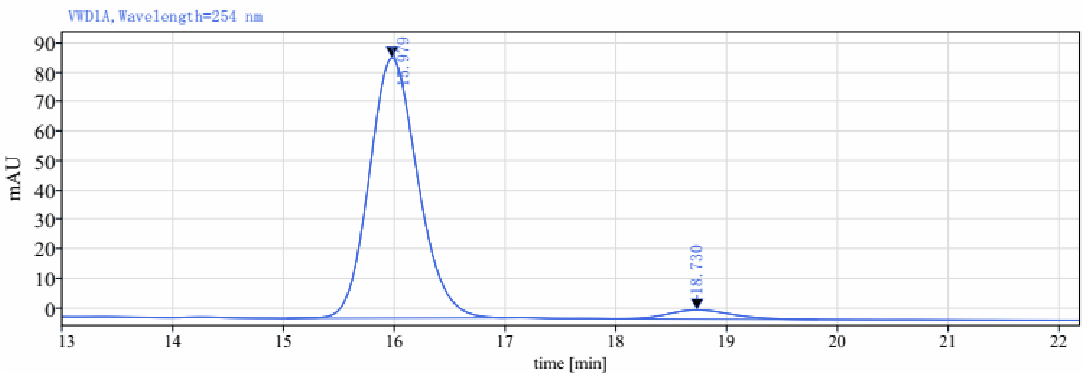

Rettime [min]	Type	Width [min]	Area	Height	Area%
15.979	BB	1.97	2683.04	88.32	95.70
18.730	BM m	1.80	120.58	3.06	4.30

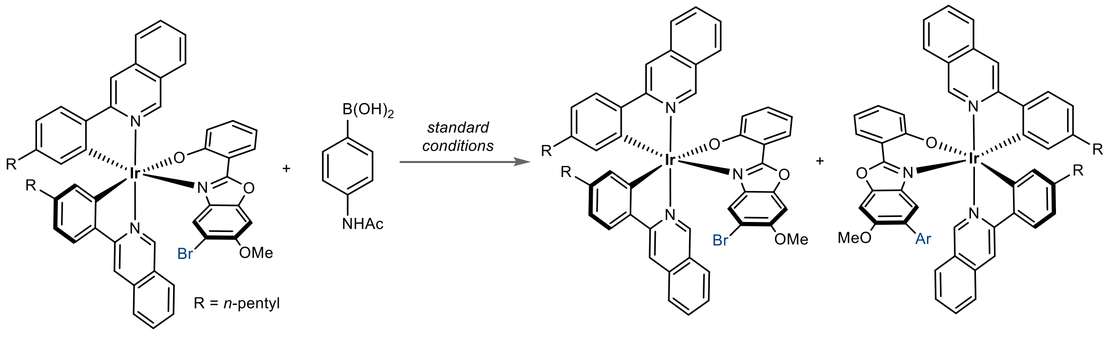

According to the general procedure, the reaction was carried out at 30 ºC for 4 days to give the

recovered Λ-**28** as orange solid (23.6 mg, 45% yield) and Δ-**35** as orange solid (25.0 mg, 45% yield).

Purification conditions: petroleum ether/EtOAc = 10:1 to 1:1.

R_f (Λ-**28**) = 0.6 in petroleum ether/EtOAc (2:1).

R_f (Δ-**35**) = 0.2 in petroleum ether/EtOAc (2:1).

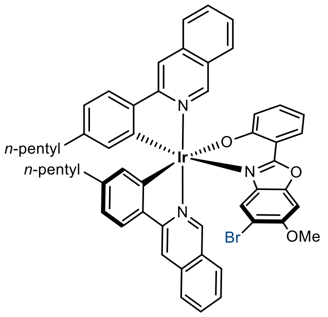

Enantiomeric excess established by HPLC analysis using a Chiralpak IK-3 column, ee = 94% (HPLC: IK-3, 254 nm, *n*-hexane/isopropanol = 80:20, flow rate 1.0 mL/min, 40 °C, t_r (major) = 8.9 min, t_r (minor) = 10.1 min.)

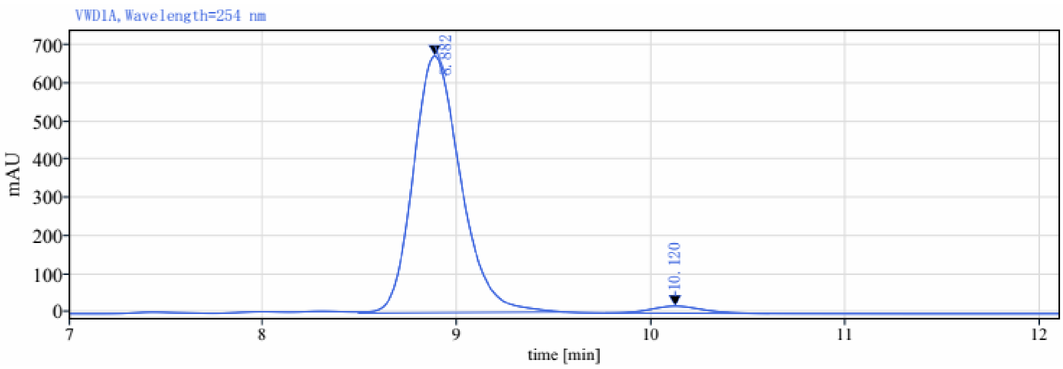

Rettime [min]	Type	Width [min]	Area	Height	Area%
8.882	VM m	1.06	11381.41	671.37	97.22
10.120	MM m	0.85	326.02	17.76	2.78

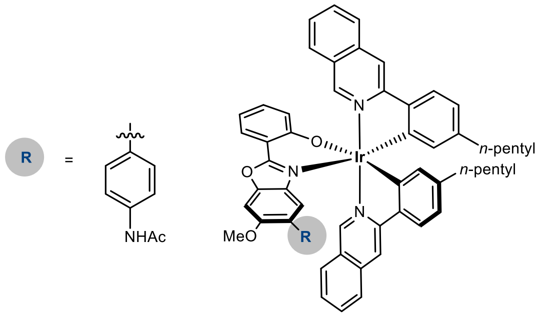

Analytical data of Δ-**35:**

^{1}H NMR (500 MHz, Chloroform-*d*) δ 9.63 (s, 1H), 8.87 (s, 1H), 8.00 (s, 1H), 7.98 – 7.91 (m, 2H), 7.75 (s, 1H), 7.69 (q, *J* = 7.5 Hz, 3H), 7.54 – 7.43 (m, 5H), 7.37 (d, *J* = 7.5 Hz, 1H), 7.31 (d, *J* = 8.5 Hz, 2H), 7.22 (t, *J* = 7.5 Hz, 1H), 7.14 (t, *J* = 8.0 Hz, 1H), 7.02 (s, 1H), 6.92 (d, *J* = 8.5 Hz, 2H), 6.78 (d, *J* = 8.5 Hz, 1H), 6.66 (d, *J* = 8.0 Hz, 1H), 6.60 (d, *J* = 8.0 Hz, 1H), 6.45 (t, *J* = 7.5 Hz, 1H), 6.38 (s, 1H), 6.11 (s, 1H), 5.89 (s, 1H), 3.71 (s, 3H), 2.15 (t, *J* = 7.5 Hz, 2H), 2.10 – 1.97 (m, 5H), 1.83 (s, 1H), 1.29 – 1.22 (m, 2H), 1.09 – 0.86 (m, 6H), 0.93 – 0.79 (m, 4H), 0.70 (t, *J* = 7.0 Hz, 3H), 0.58 (t, *J* = 7.0 Hz, 3H).

^{13}C NMR (126 MHz, $CDCl_3$) δ 169.0, 168.5, 162.1, 161.8, 159.9, 154.9, 152.22, 152.15, 149.5, 149.2, 145.7, 143.3, 143.0, 142.3, 141.9, 137.1, 136.48, 136.46, 135.0, 133.8, 133.4, 133.0, 132.0, 131.6, 131.4, 130.4, 128.6, 128.1, 127.6, 127.5, 127.4, 127.3, 126.5, 126.4, 126.3, 125.3, 123.3, 122.8, 122.1, 120.3, 118.9, 114.0, 113.8, 113.2, 111.6, 93.7, 56.2, 35.8, 35.7, 31.5, 31.3, 30.21, 30.18, 24.5, 22.5, 22.4, 14.1, 14.08.

HRMS (ESI) for $C_{62}H_{57}IrN_4O_4$ $[M+H]^+$ calcd.1115.4082, found 1115.4081

Enantiomeric excess established by HPLC analysis using a Chiralpak IK-3 column, ee = 93% (HPLC: IK-3, 254 nm, *n*-hexane/isopropanol = 80:20, flow rate 1.0 mL/min, 40 ^oC, t_r (major) = 25.9 min, t_r (minor) = 31.7 min.)

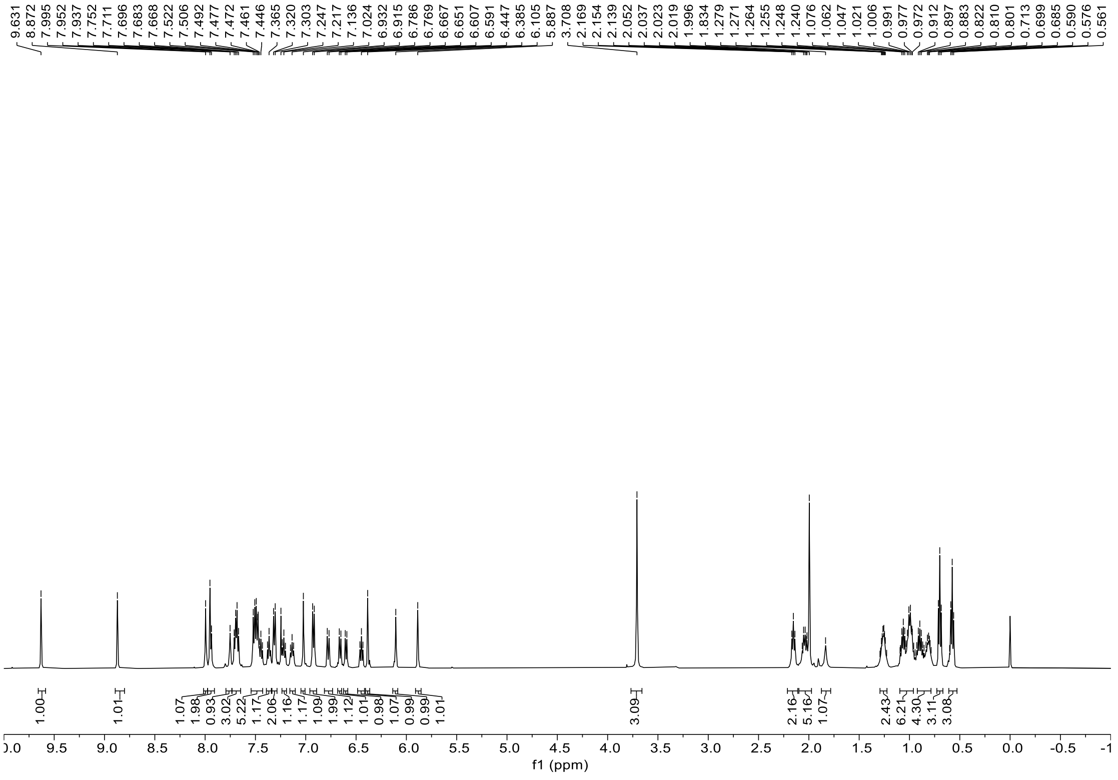

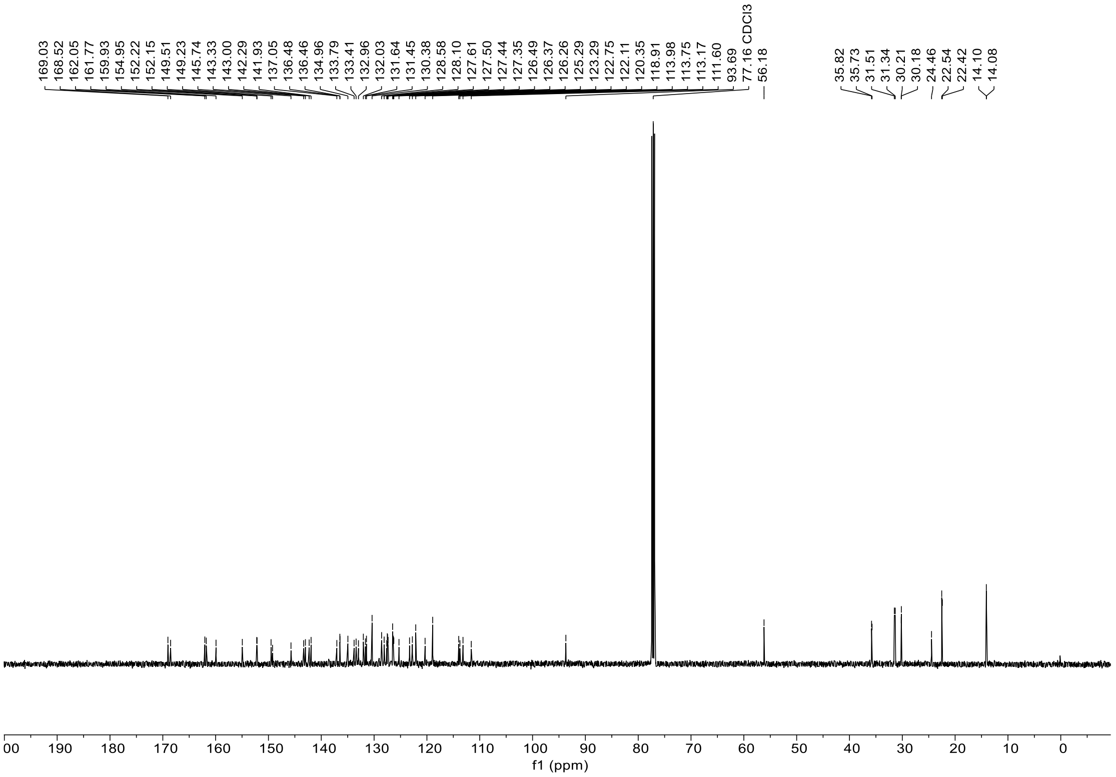

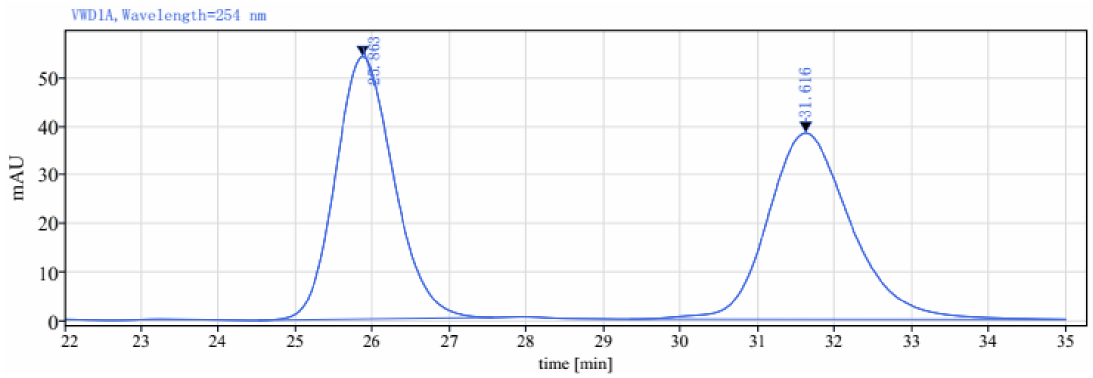

Rettime [min]	Type	Width [min]	Area	Height	Area%
25.863	BB	3.05	2848.40	54.05	49.68
31.616	BBA	5.67	2885.45	38.41	50.32

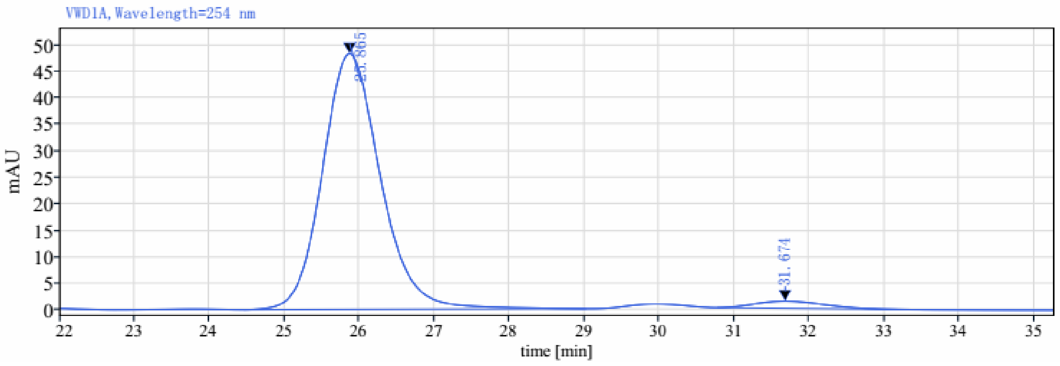

Rettime [min]	Type	Width [min]	Area	Height	Area%
25.865	BB	4.57	2597.09	48.21	96.72
31.674	BB	2.60	88.18	1.37	3.28

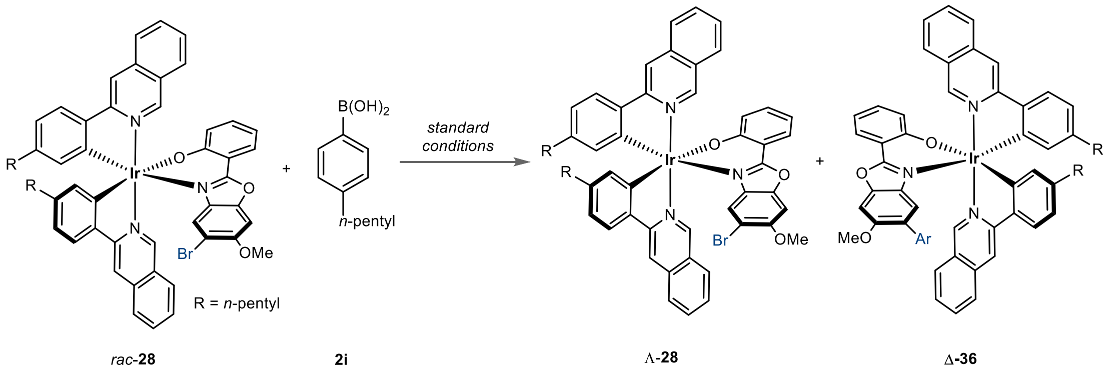

According to the general procedure, the reaction was carried out at 30 °C for 4.5 days to give the recovered Λ-**28** as orange solid (22.6 mg, 42% yield) and Δ-**36** as orange solid (22.1 mg, 40% yield).

Purification conditions: petroleum ether/EtOAc = 10:1 to 3:1.

R_f (Λ-**28**) = 0.4 in petroleum ether/EtOAc (3:1).

R_f (Δ-**36**) = 0.5 in petroleum ether/EtOAc (3:1).

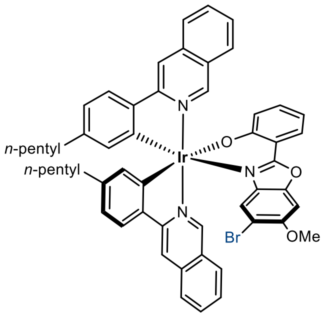

Enantiomeric excess established by HPLC analysis using a Chiralpak IK-3 column, ee = 93%

(HPLC: IK-3, 254 nm, *n*-hexane/isopropanol = 80:20, flow rate 1.0 mL/min, 40 °C, t_r (major) = 8.8 min, t_r (minor) = 10.0 min.)

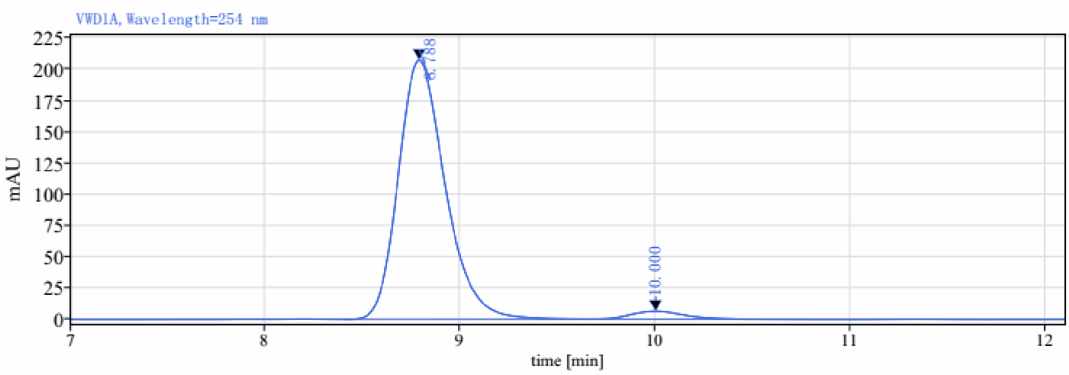

Rettime [min]	Type	Width [min]	Area	Height	Area%
8.788	VM m	1.26	3470.66	207.55	96.62
10.000	MM m	0.83	121.53	6.30	3.38

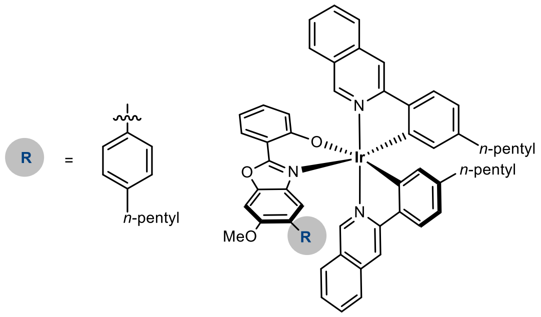

Analytical data of Δ-**36:**

^{1}H NMR (500 MHz, $CDCl_3$) δ 9.64 (s, 1H), 8.88 (s, 1H), 8.08 (d, *J* = 7.5 Hz, 2H), 7.91 (dd, *J* = 8.0, 2.0 Hz, 1H), 7.82 (t, *J* = 9.0 Hz, 2H), 7.76 (d, *J* = 8.0 Hz, 1H), 7.62 – 7.53 (m, 5H), 7.42 (t, *J* = 7.5 Hz, 1H), 7.35 (t, *J* = 7.5 Hz, 1H), 7.11 (dd, *J* = 17.5, 7.5 Hz, 3H), 7.03 (s, 1H), 7.00 (d, *J* = 8.0 Hz, 2H), 6.76 (d, *J* = 8.5 Hz, 1H), 6.69 (dd, *J* = 8.0, 1.5 Hz, 1H), 6.63 (dd, *J* = 8.0, 1.5 Hz, 1H), 6.41 (t, *J* = 7.5 Hz, 1H), 69.31 (s, 1H), 6.14 (s, 1H), 5.90 (s, 1H), 3.76 (s, 3H), 2.60 (t, *J* = 7.5 Hz, 2H), 2.19 (t, *J* = 7.5 Hz, 2H), 2.05 (td, *J* = 7.5, 5.0 Hz, 2H), 1.64 (t, *J* = 7.5 Hz, 2H), 1.39 – 1.35 (m, 4H), 1.31 – 1.26 (m, 2H), 1.13 – 1.06 (m, 2H), 1.05 – 0.89 (m, 9H), 0.88 – 0.83 (m, 2H), 0.73 (t, *J* = 7.5 Hz, 3H), 0.62 (t, *J* = 7.5 Hz, 3H).

^{13}C NMR (126 MHz, $CDCl_3$) δ 169.3, 162.2, 161.9, 159.9, 154.8, 152.2, 152.1, 149.54, 149.47, 146.2, 143.2, 142.9, 142.4, 141.8, 141.5, 136.53, 136.47, 134.9, 134.7, 134.0, 133.1, 132.1, 131.6, 131.5, 129.9, 128.4, 128.2, 128.0, 127.7, 127.6, 127.5, 127.4, 126.5, 126.44, 126.36,

126.3, 125.8, 123.2, 122.7, 122.0, 121.9, 120.6, 113.7, 113.5, 113.1, 111.4, 93.5, 56.2, 35.9, 35.8, 31.7, 31.53, 31.49, 31.3, 30.3, 30.2, 22.8, 22.6, 22.5, 14.2, 14.14, 14.12.

HRMS (ESI) for $C_{65}H_{65}IrN_3O_3$ $[M+H]^+$ calcd.1128.4650, found 1128.4657

Enantiomeric excess established by HPLC analysis using a Chiralpak IM column, ee = 93% (HPLC: IM, 254 nm, *n*-hexane/isopropanol = 95:5, flow rate 1.0 mL/min, 40 °C, t_r (major) = 17.8 min, t_r (minor) = 22.3 min.)

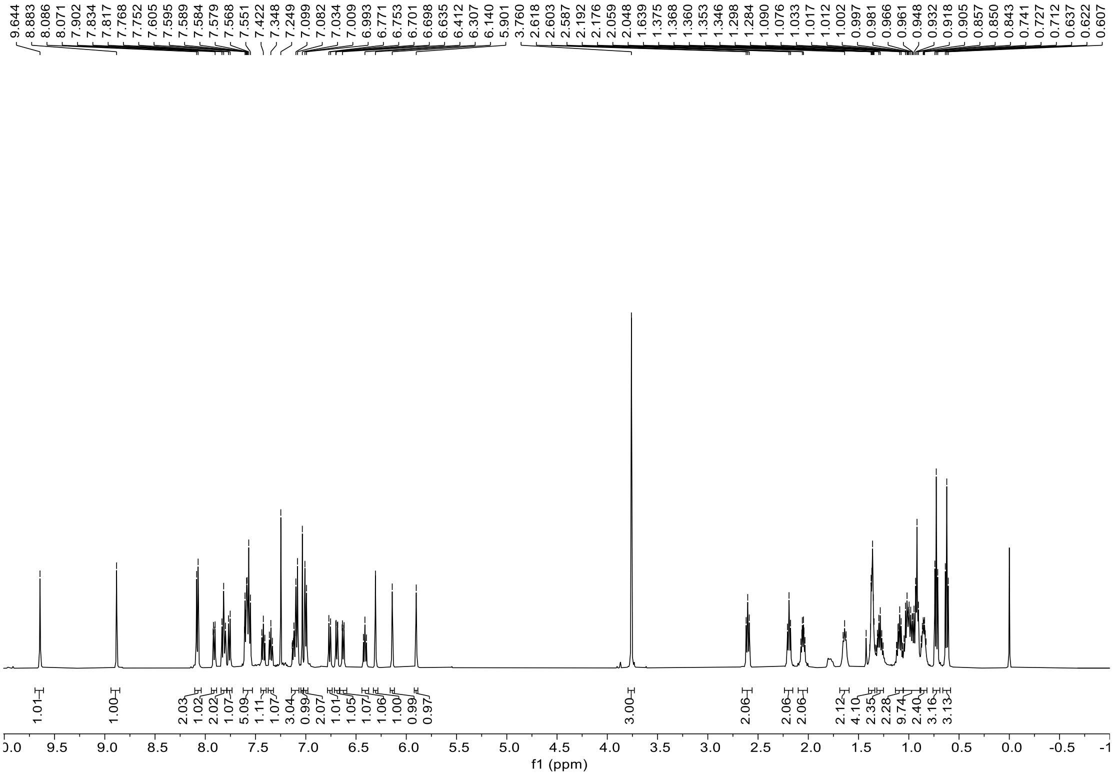

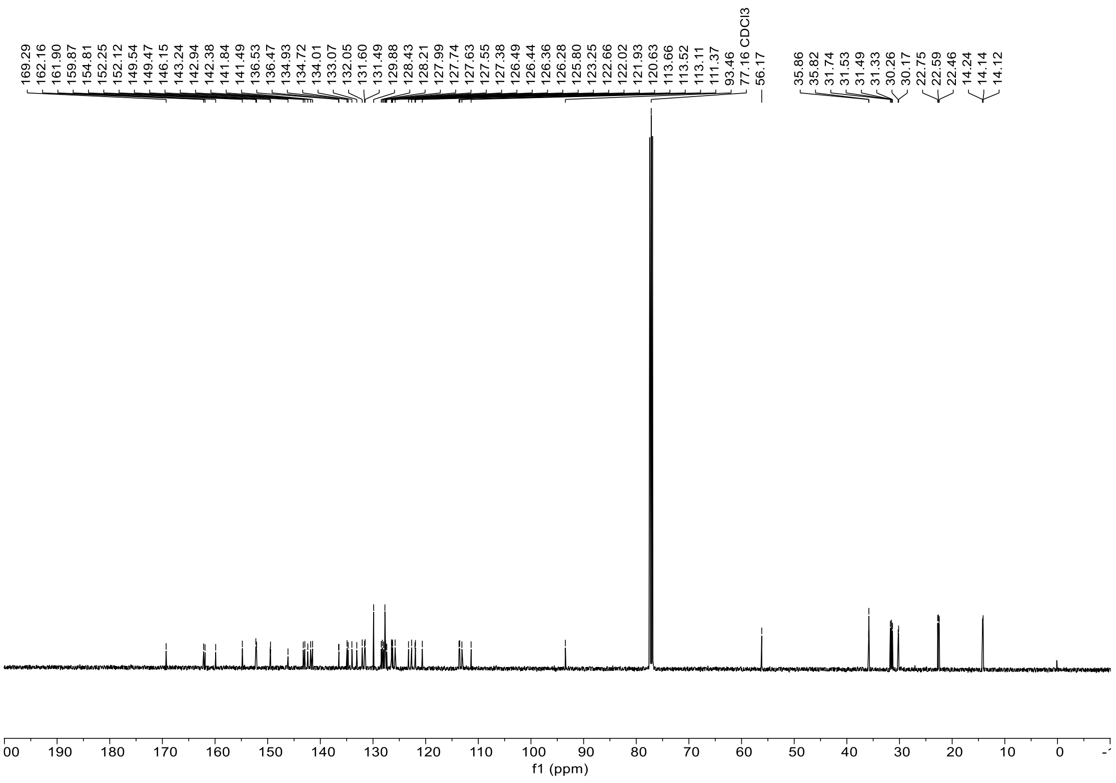

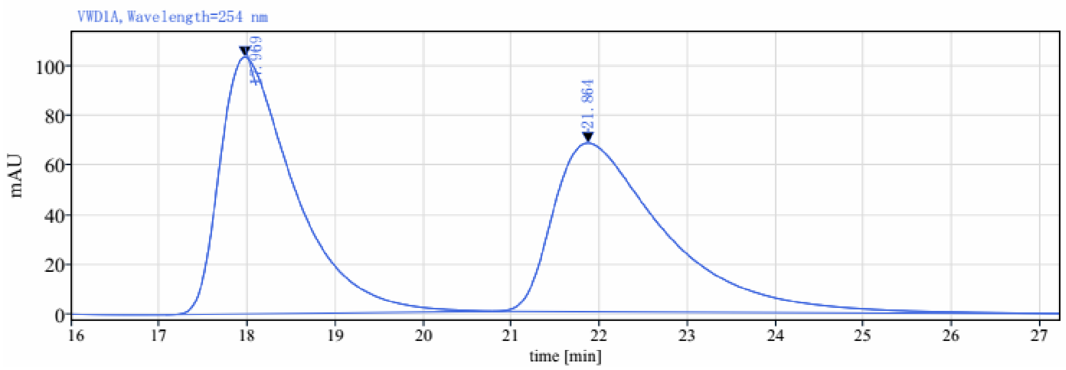

Rettime [min]	Type	Width [min]	Area	Height	Area%
17.969	BB	3.71	6050.03	102.90	50.33
21.864	BB	7.31	5971.28	67.52	49.67

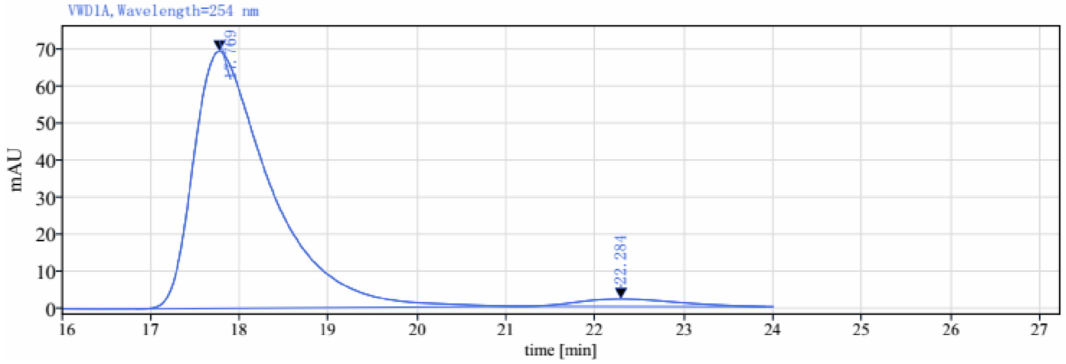

Rettime [min]	Type	Width [min]	Area	Height	Area%
17.769	BB	4.32	4192.18	69.71	96.47
22.284	BBA	2.77	153.47	2.00	3.53

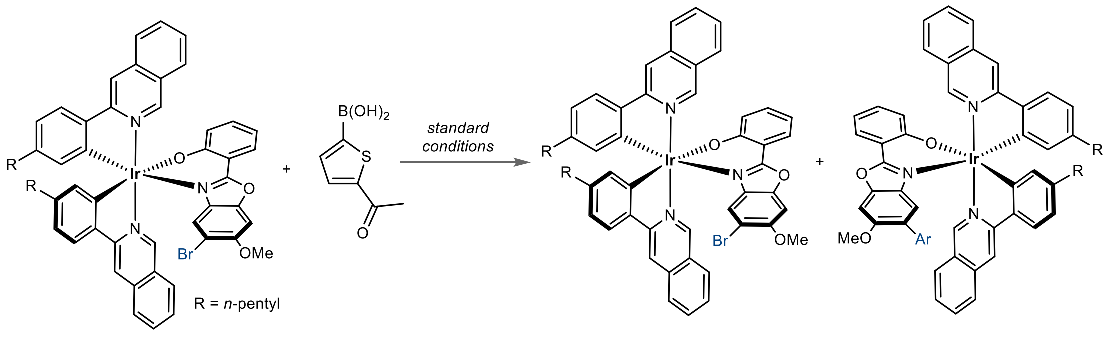

rac-**28** **2j** Λ-**28** Δ-**37**

According to the general procedure, the reaction was carried out at 30 °C for 6 days to give the recovered Λ-**28** as orange solid (23.2 mg, 43% yield) and Δ-**37** as orange solid (23.2 mg, 42% yield).

Purification conditions: petroleum ether/EtOAc = 10:1 to 2:1.

R_f (Λ-**28**) = 0.6 in petroleum ether/EtOAc (2:1).

R_f (Δ-**37**) = 0.4 in petroleum ether/EtOAc (2:1).

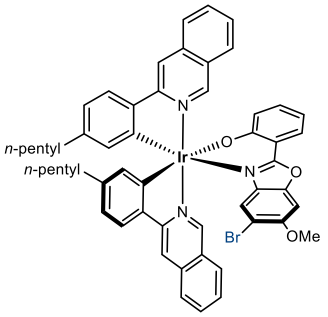

Enantiomeric excess established by HPLC analysis using a Chiralpak IK-3 column, ee = 93%

(HPLC: IK-3, 254 nm, *n*-hexane/isopropanol = 80:20, flow rate 1.0 mL/min, 40 °C, t_r (major) = 8.9 min, t_r (minor) = 10.1 min.)

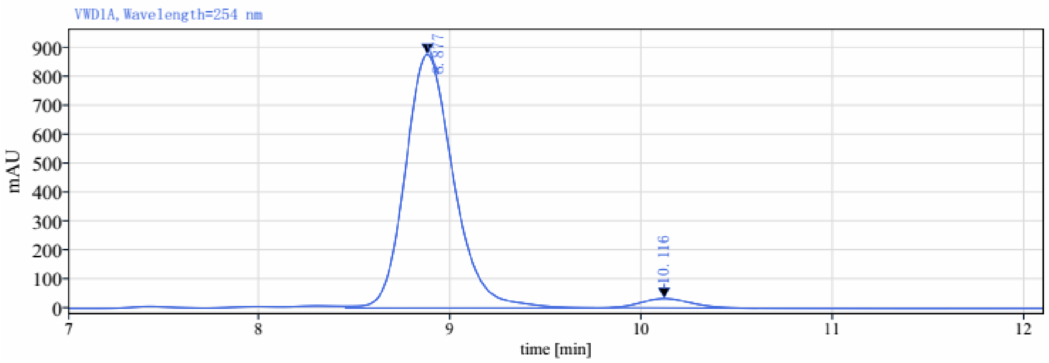

Rettime [min]	Type	Width [min]	Area	Height	Area%
8.877	VB	1.31	15331.55	875.06	96.40
10.116	MM m	0.77	572.88	31.95	3.60

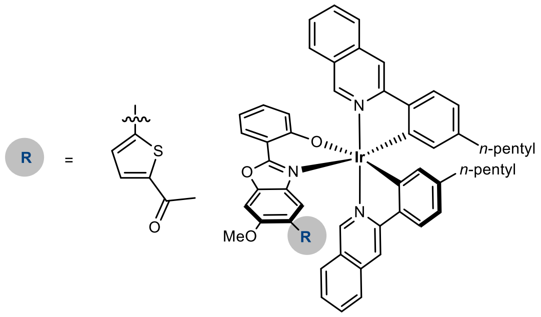

Analytical data of Δ-**37:**

^{1}H NMR (500 MHz, $CDCl_3$) δ 9.64 (s, 1H), 8.85 (s, 1H), 8.11 (d, *J* = 4.5 Hz, 2H), 7.90 (d, *J* = 8.0 Hz, 1H), 7.83 (d, *J* = 8.5 Hz, 2H), 7.78 (d, *J* = 8.5 Hz, 1H), 7.71 (d, *J* = 8.0 Hz, 1H), 7.63 – 7.55 (m, 4H), 7.52 (d, *J* = 4.0 Hz, 1H), 7.44 (t, *J* = 7.5 Hz, 1H), 7.36 (t, *J* = 7.5 Hz, 1H), 7.12 (t, *J* = 8.0 Hz, 1H), 7.08 (s, 1H), 6.94 (s, 1H), 6.82 (d, *J* = 8.0 Hz, 1H), 6.76 – 6.67 (m, 3H), 6.41 (t, *J* = 7.5 Hz, 1H), 6.22 (s, 1H), 5.88 (s, 1H), 3.93 (s, 3H), 2.55 (s, 3H), 2.27 – 2.16 (m, 4H), 1.34 – 1.22 (m, 4H), 1.13 – 0.92 (m, 8H), 0.74 (t, *J* = 7.0 Hz, 3H), 0.63 (t, *J* = 7.0 Hz, 3H).
^{13}C NMR (126 MHz, $CDCl_3$) δ 191.3, 169.7, 162.1, 161.6, 160.5, 154.4, 152.12, 152.05, 150.4, 149.1, 146.8, 146.6, 143.5, 143.1, 143.0, 142.5, 141.7, 136.5, 135.5, 134.1, 133.4, 131.9, 131.7, 131.6, 128.4, 128.2, 127.6, 127.5, 127.4, 126.8, 126.6, 126.5, 126.4, 126.0, 123.5, 122.7, 122.1, 121.8, 119.8, 118.0, 113.8, 113.7, 113.3, 110.9, 94.0, 56.3, 35.9, 35.8, 31.44, 31.38, 30.6, 30.2, 26.9, 22.6, 22.5, 14.2, 14.1.

HRMS (ESI) for $C_{60}H_{55}IrN_3O_4S$ $[M+H]^+$ calcd.1106.3537, found 1106.3547

Enantiomeric excess established by HPLC analysis using a Chiralpak IK-3 column, ee = 95% (HPLC: IK-3, 254 nm, *n*-hexane/isopropanol = 70:30, flow rate 1.0 mL/min, 40 °C, t_r (major) = 18.2 min, t_r (minor) = 23.9 min.)

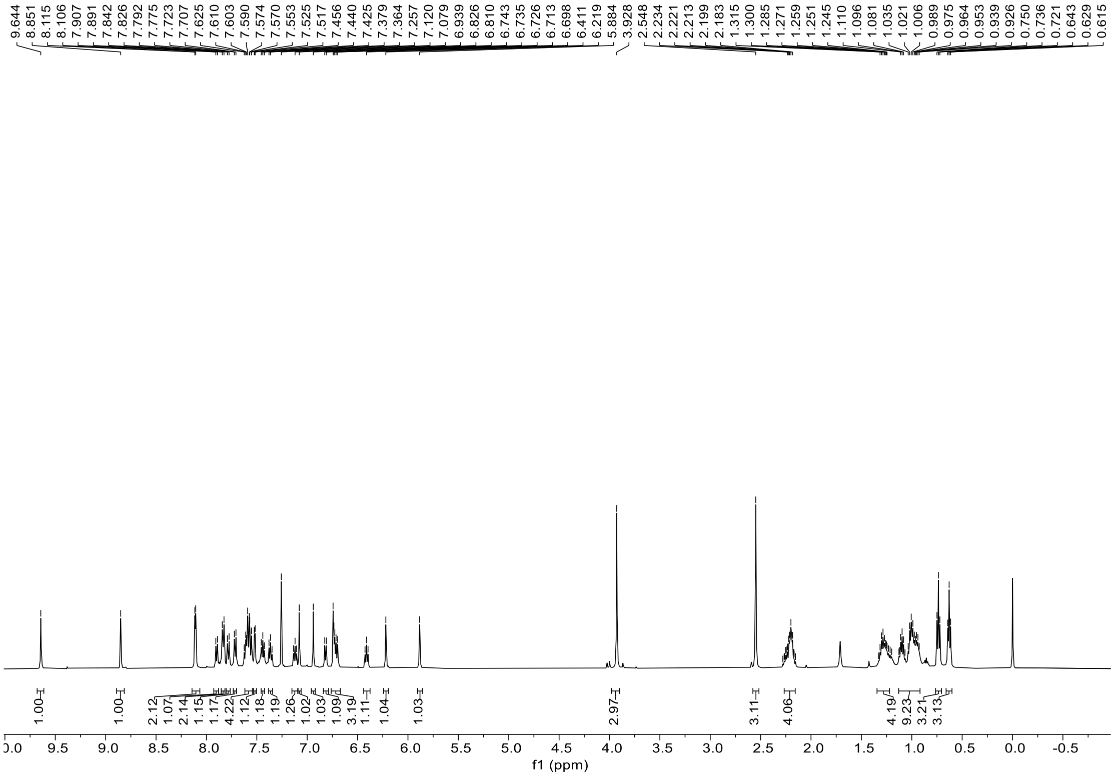

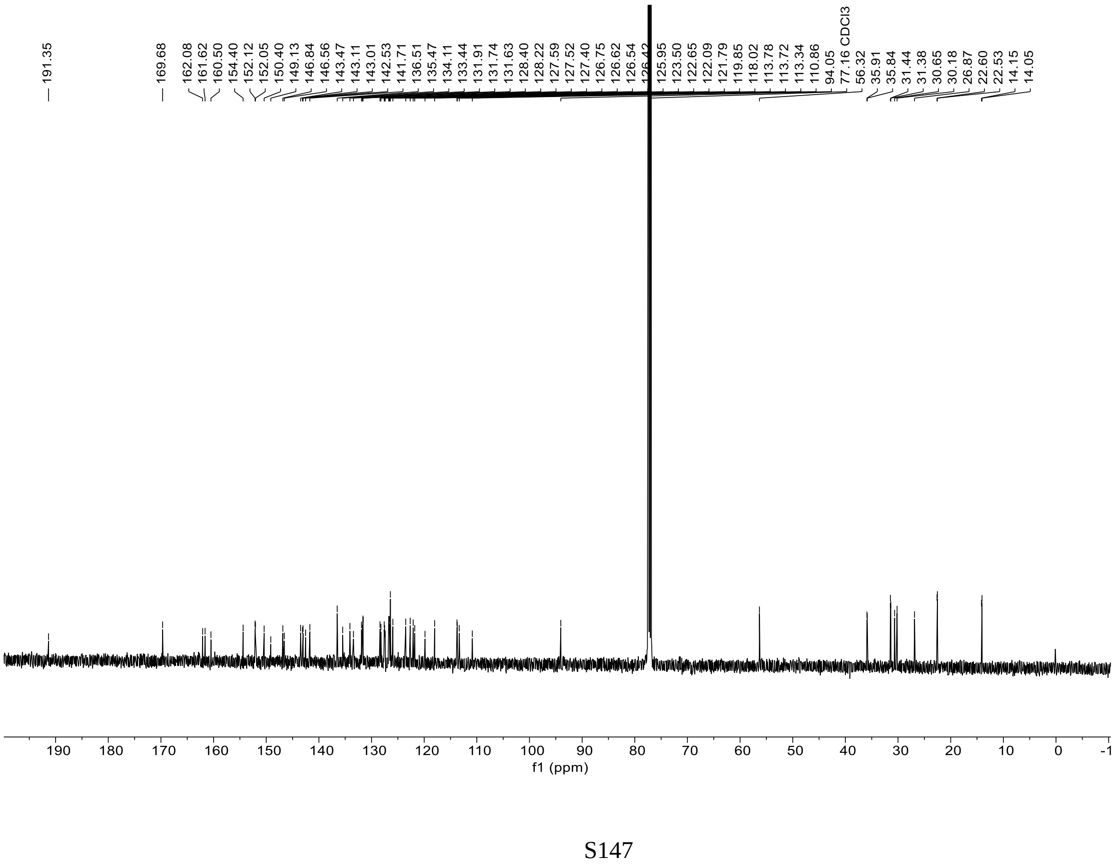

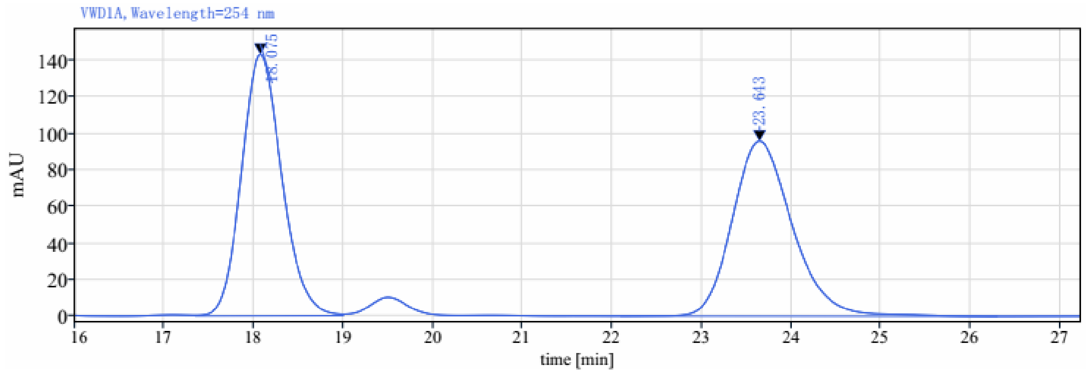

Rettime [min]	Type	Width [min]	Area	Height	Area%
18.075	VV	1.65	4465.63	142.59	50.05
23.643	BB	4.21	4457.46	95.44	49.95

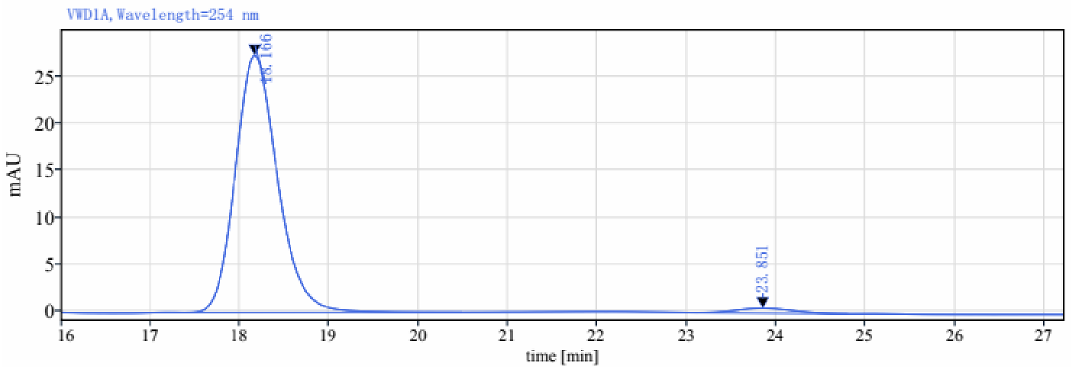

Rettime [min]	Type	Width [min]	Area	Height	Area%
18.166	BB	3.09	899.16	27.30	97.58
23.851	BB	1.71	22.33	0.52	2.42

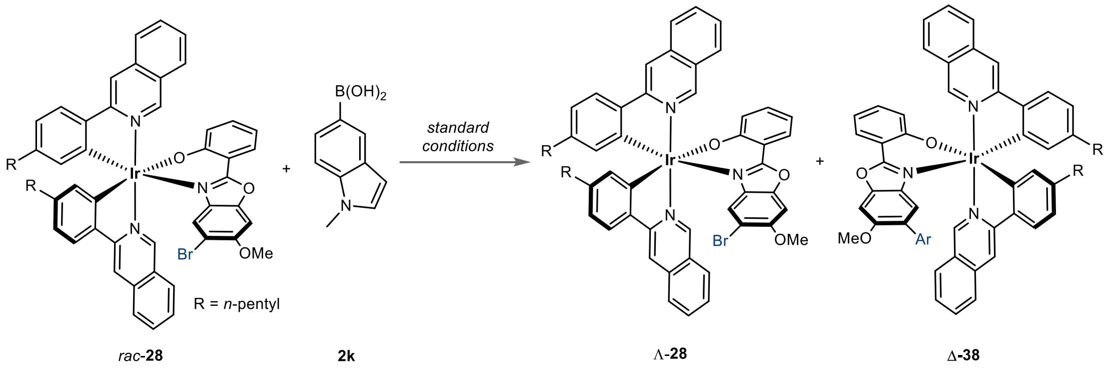

According to the general procedure, the reaction was carried out at 30 ºC for 2.5 days to give the recovered Λ-**28** as orange solid (22.3 mg, 42% yield) and Δ-**38** as orange solid (23.5 mg,

42% yield).

Purification conditions: petroleum ether/EtOAc = 10:1 to 2:1.

R_f (Λ-**28**) = 0.6 in petroleum ether/EtOAc (2:1).

R_f (Δ-**38**) = 0.5 in petroleum ether/EtOAc (2:1).

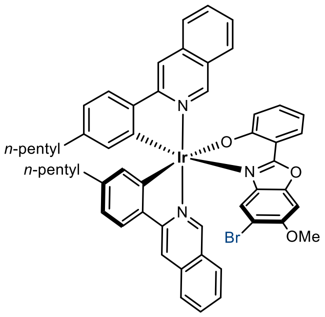

Enantiomeric excess established by HPLC analysis using a Chiralpak IK-3 column, ee = 93% (HPLC: IK-3, 254 nm, *n*-hexane/isopropanol = 80:20, flow rate 1.0 mL/min, 40 °C, t_r (major) = 8.8 min, t_r (minor) = 10.0 min.)

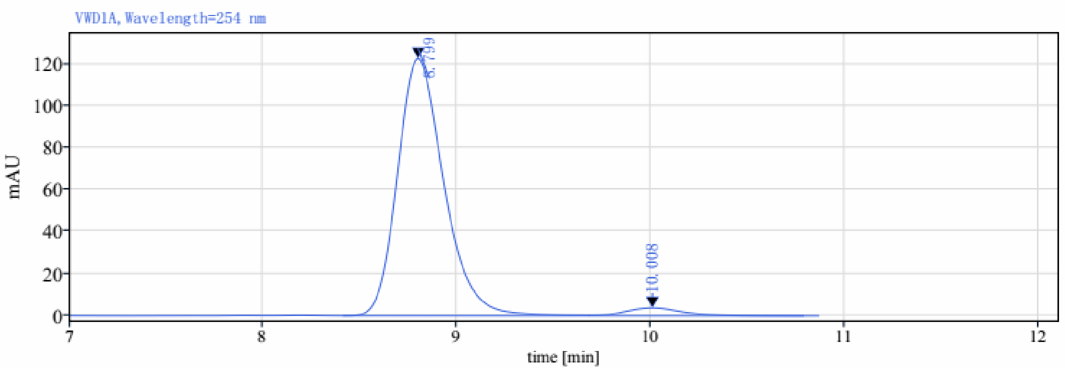

Rettime [min]	Type	Width [min]	Area	Height	Area%
8.799	BV	1.27	2043.61	122.73	96.35
10.008	VBA	1.11	77.53	3.81	3.65

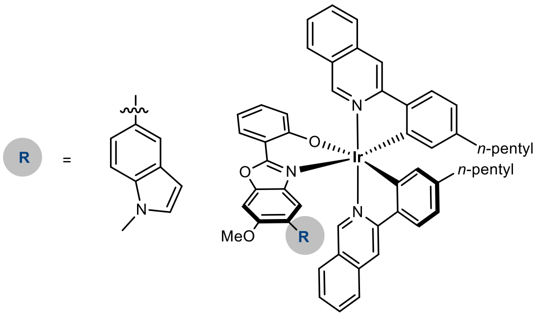

Analytical data of Δ-**38:**

^{1}H NMR (500 MHz, $CDCl_3$) δ 9.67 (s, 1H), 8.92 (s, 1H), 8.05 (d, *J* = 11.5 Hz, 2H), 7.92 (dd, *J*

= 8.0, 2.0 Hz, 1H), 7.79 – 7.75 (m, 3H), 7.60 – 7.52 (m, 5H), 7.40 (t, *J* = 7.5 Hz, 1H), 7.34 (t, *J* = 7.5 Hz, 1H), 7.24(s, 1H) 7.19 (d, *J* = 8.5 Hz, 1H), 7.12 – 7.09 (m, 1H), 7.07 – 7.01 (m, 3H), 6.75 (d, *J* = 8.5 Hz, 1H), 6.69 – 6.63 (m, 2H), 6.51 (d, *J* = 3.0 Hz, 1H), 6.41 (d, *J* = 4.0 Hz, 2H), 6.15 (d, *J* = 2.0 Hz, 1H), 5.91 (d, *J* = 1.5 Hz, 1H), 3.77 (s, 3H), 3.75 (s, 3H), 2.19 (t, *J* = 7.5 Hz, 2H), 2.02 (q, *J* = 7.0 Hz, 2H), 1.32 – 1.25 (m, 2H), 1.12 – 1.00 (m, 4H), 0.89 – 0.82 (m, 4H), 0.80 – 0.75 (m, 2H), 0.72 (t, *J* = 7.0 Hz, 3H), 0.58 (t, *J* = 7.0 Hz, 3H).

^{13}C NMR (126 MHz, $CDCl_3$) δ 169.4, 162.2, 162.0, 159.9, 155.0, 152.4, 152.1, 149.7, 149.2, 146.2, 143.2, 142.9, 142.4, 141.9, 136.6, 136.5, 136.0, 134.9, 134.0, 133.0, 132.1, 131.5, 131.4, 129.5, 128.8, 128.7, 128.4, 128.3, 128.2, 127.7, 127.5, 127.4, 126.5, 126.38, 126.36, 126.2, 125.7, 124.1, 123.3, 122.6, 122.5, 122.0, 121.9, 120.9, 113.6, 113.5, 113.1, 111.6, 108.1, 101.4, 93.6, 56.2, 35.9, 35.8, 33.0, 31.54, 31.5, 30.14, 30.11, 22.6, 22.3, 14.12, 14.06.

HRMS (ESI) for $C_{63}H_{58}IrN_4O_3$ $[M+H]^+$ calcd.1111.4133, found 1111.4133

Enantiomeric excess established by HPLC analysis using a Chiralpak IM column, ee = 94% (HPLC: IM, 254 nm, *n*-hexane/isopropanol = 90:10, flow rate 1.0 mL/min, 40 °C, t_r (major) = 23.9 min, t_r (minor) = 29.3 min.)

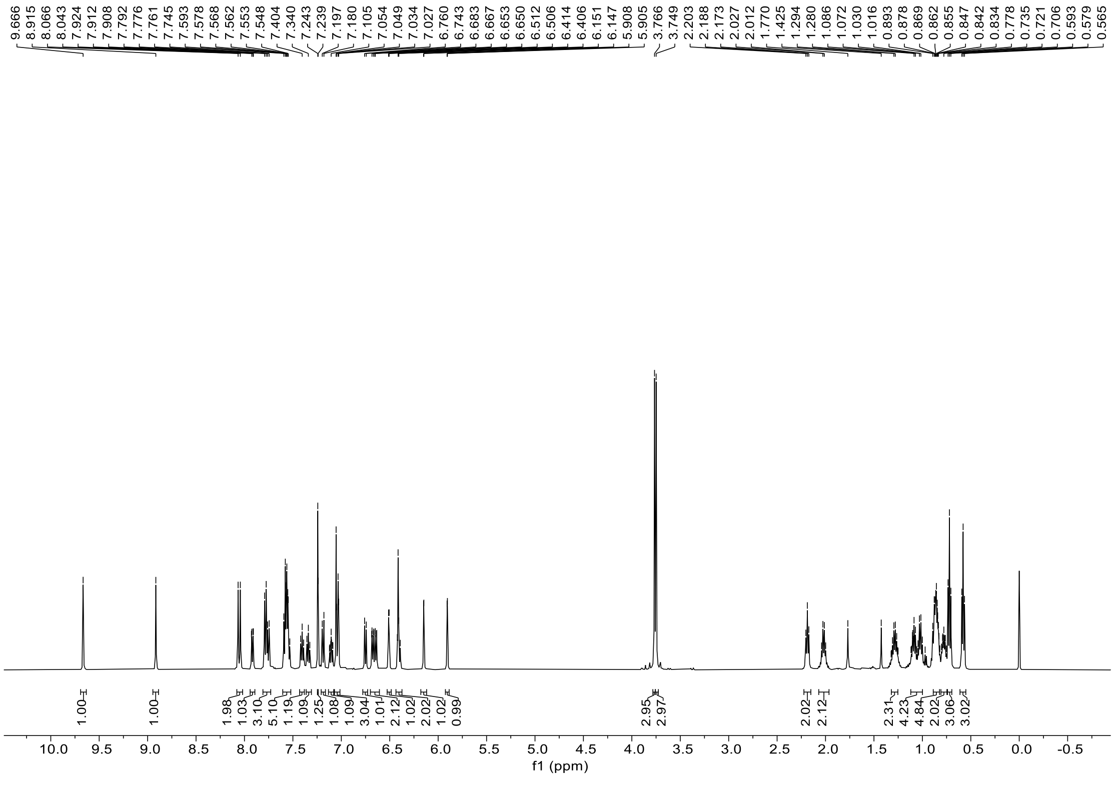

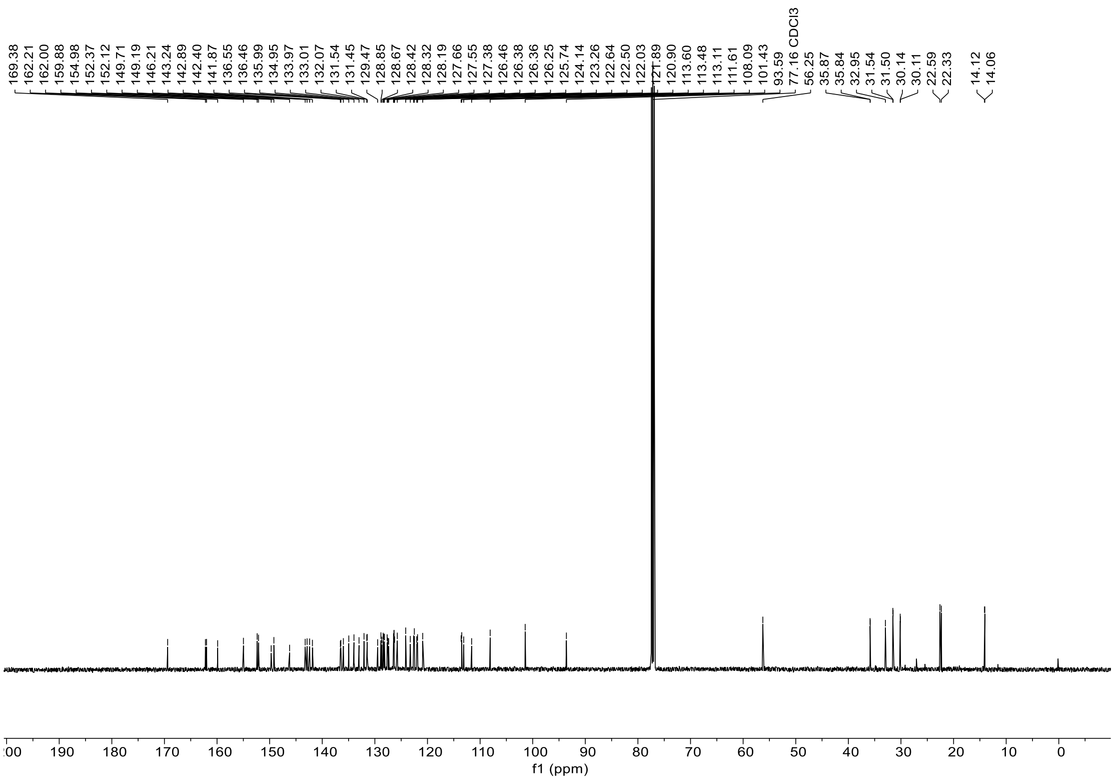

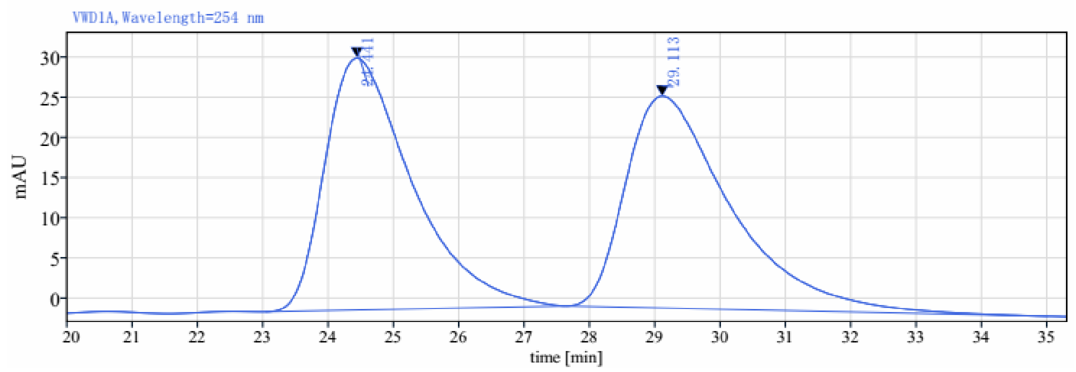

Rettime [min]	Type	Width [min]	Area	Height	Area%
24.441	BB	4.59	2916.50	31.40	49.68
29.113	BBA	7.37	2954.32	26.39	50.32

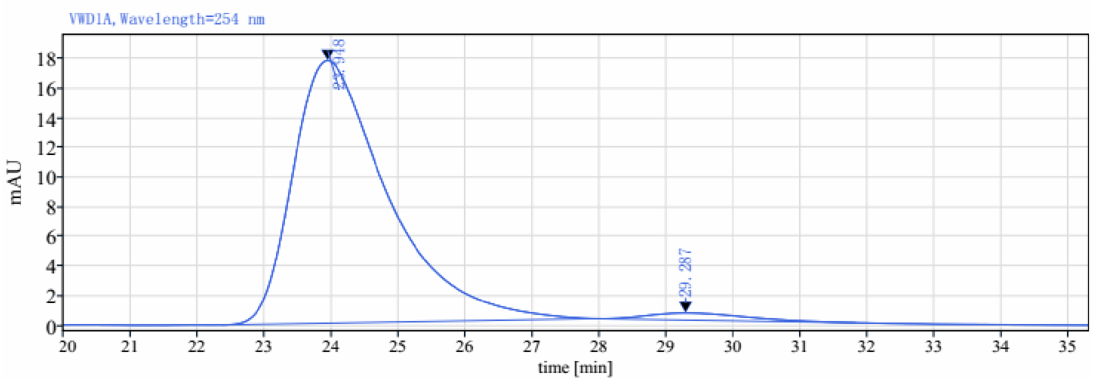

Rettime [min]	Type	Width [min]	Area	Height	Area%
23.948	BM m	5.53	1744.71	17.71	97.30
29.287	MM m	3.92	48.41	0.47	2.70

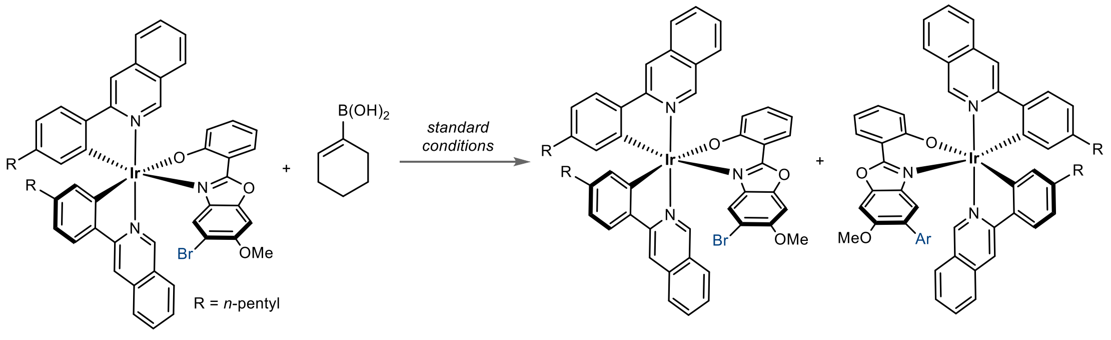

According to the general procedure, the reaction was carried out at 30 ºC for 3 days to give the recovered Λ-**28** as orange solid (18.1 mg, 34% yield) and Δ-**39** as orange solid (19.9 mg, 37% yield).

Purification conditions: petroleum ether/EtOAc = 10:1 to 3:1.

R_f (Λ-**28**) = 0.5 in petroleum ether/EtOAc (3:1).

R_f (Δ-**39**) = 0.6 in petroleum ether/EtOAc (3:1).

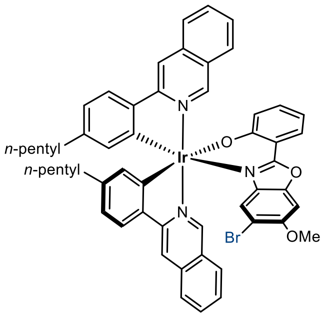

Enantiomeric excess established by HPLC analysis using a Chiralpak IK-3 column, ee = 90% (HPLC: IK-3, 254 nm, *n*-hexane/isopropanol = 80:20, flow rate 1.0 mL/min, 40 ºC, t_r (major) = 8.9 min, t_r (minor) = 10.0 min.)

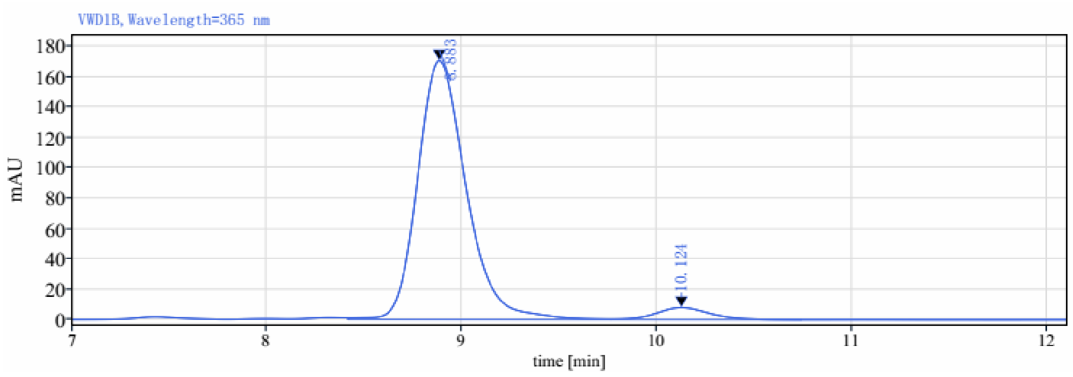

Rettime [min]	Type	Width [min]	Area	Height	Area%
8.883	VV	1.35	2951.35	170.54	95.18
10.124	VB	0.98	149.60	7.92	4.82

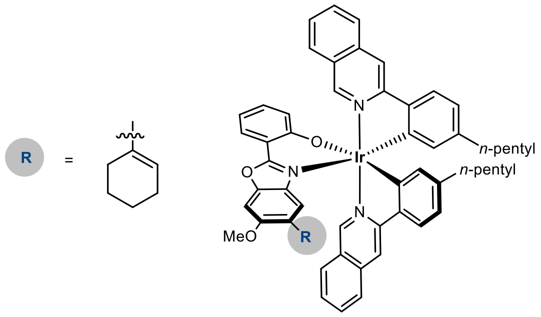

Analytical data of Δ-**39:**

^{1}H NMR (500 MHz, Chloroform-*d*) δ 9.65 (s, 1H), 8.89 (s, 1H), 8.10 (s, 1H), 8.07 (s, 1H), 7.89 – 7.80 (m, 3H), 7.77 (d, *J* = 8.0 Hz, 1H), 7.63 – 7.55 (m, 5H), 7.42 (t, *J* = 7.5 Hz, 1H), 7.36 (t, *J* = 7.5 Hz, 1H), 7.11 – 7.04 (m, 1H), 6.91 (s, 1H), 6.75 – 6.66 (m, 3H), 6.39 (t, *J* = 7.5 Hz, 1H), 6.19 (s, 1H), 5.97 (s, 1H), 5.90 (s, 1H), 5.27 – 5.17 (m, 1H), 3.75 (s, 3H), 2.24 – 2.07 (m, 7H), 2.01 – 1.90 (m, 1H), 1.67 – 1.56 (m, 4H), 1.34 – 1.25 (m, 4H), 1.13 – 1.01 (m, 8H), 0.70 (dt, *J* = 25.5, 7.0 Hz, 6H).

^{13}C NMR (126 MHz, $CDCl_3$) δ 169.3, 162.2, 162.0, 159.8, 155.1, 152.4, 152.0, 149.6, 149.2, 146.5, 143.2, 142.9, 142.6, 141.9, 136.6, 136.5, 136.4, 134.5, 134.0, 132.9, 132.1, 131.6, 131.5, 131.3, 128.4, 128.2, 127.61, 127.57, 127.4, 127.0, 126.5, 126.4, 126.35, 126.27, 125.7, 123.2, 122.7, 121.9, 121.4, 119.6, 113.6, 113.4, 113.0, 111.6, 93.0, 56.1, 35.9, 35.88, 31.7, 31.6, 30.6,

30.1, 28.8, 25.7, 23.1, 22.6, 22.5, 22.3, 14.13, 14.09.

HRMS (ESI) for $C_{60}H_{59}IrN_3O_3$ $[M+H]^+$ calcd.1062.4180, found 1062.4180

Enantiomeric excess established by HPLC analysis using a Chiralpak IM column, ee = 82% (HPLC: IM, 254 nm, *n*-hexane/isopropanol = 90:10, flow rate 1.0 mL/min, 40 °C, t_r (major) = 9.6 min, t_r (minor) = 11.1 min.)

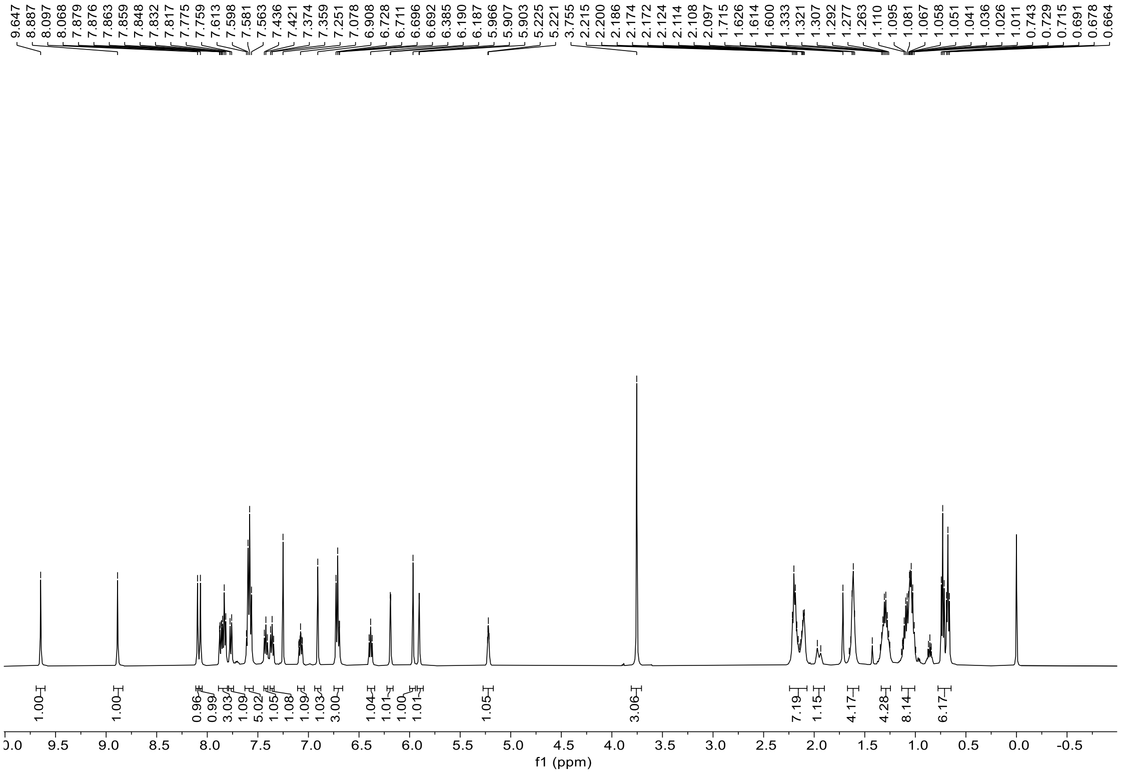

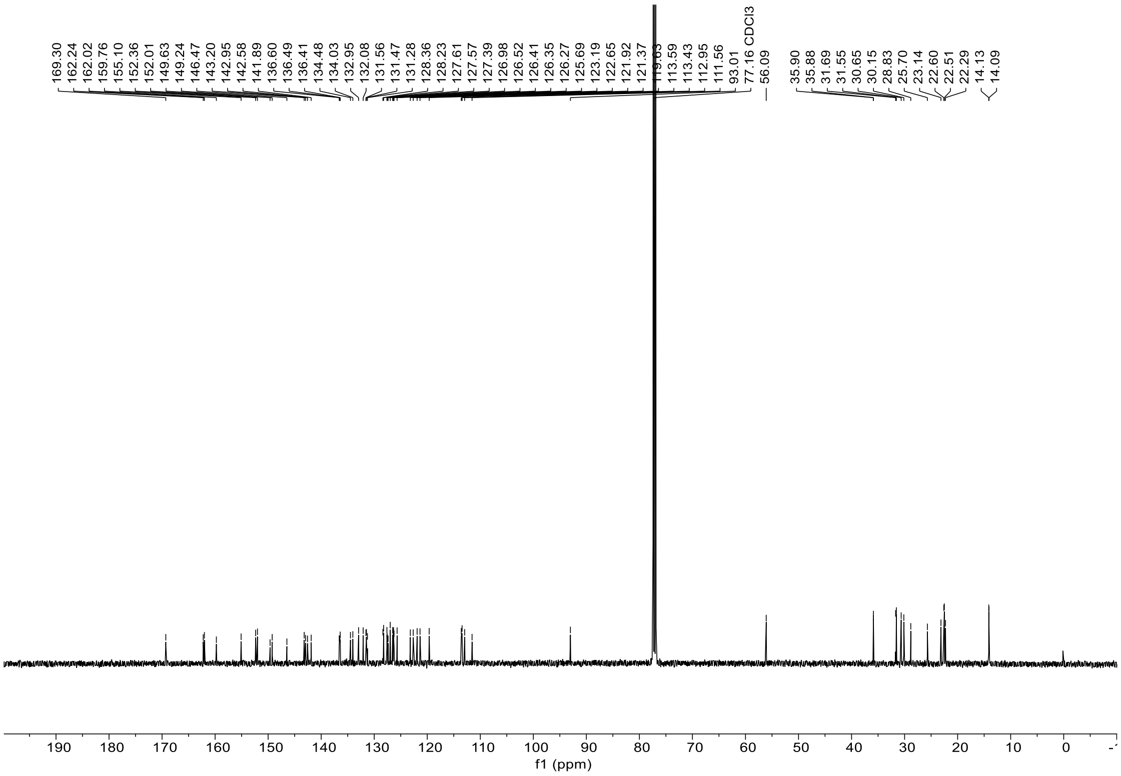

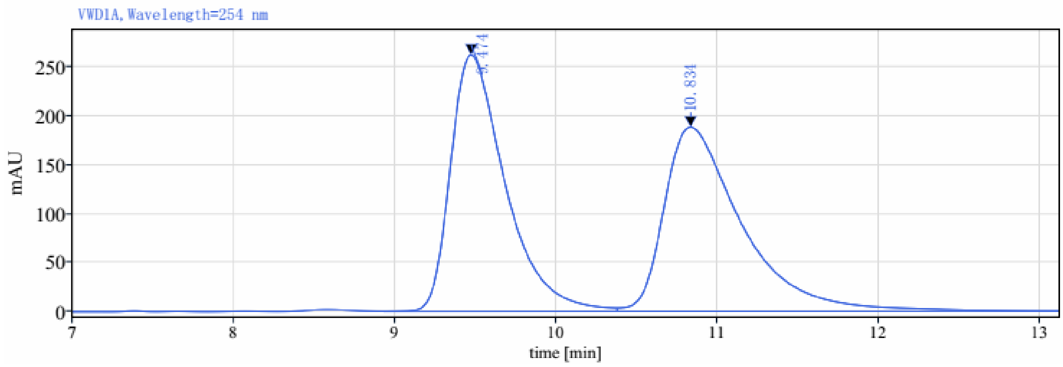

Rettime [min]	Type	Width [min]	Area	Height	Area%
9.474	VV	1.45	6196.92	261.92	49.51
10.834	VBA	3.11	6319.69	188.08	50.49

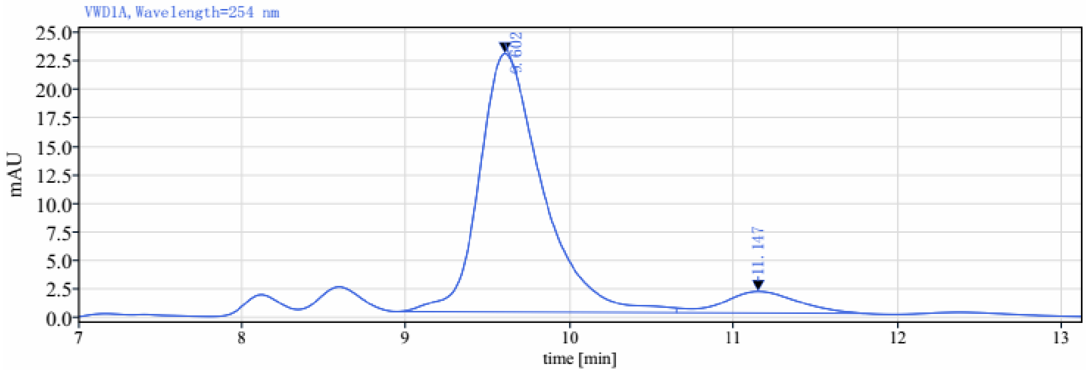

Rettime [min]	Type	Width [min]	Area	Height	Area%
9.602	MM m	1.73	607.33	22.67	91.05
11.147	MM m	1.14	59.71	1.89	8.95

5. Synthetic Transformations

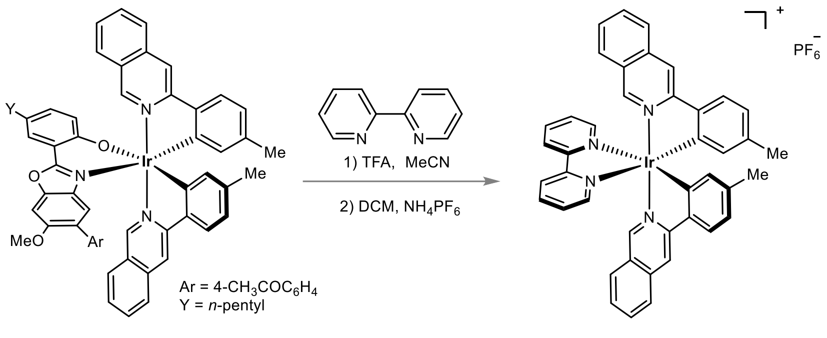

Δ-**11**, 94% ee **40**, 90% yield

To a flame-dried 25 mL round bottom flask was added Δ-**11** (0.02 mmol), acetonitrile (10 mL) was then added and the reaction sparged with N_2 for 5 min followed by the addition of trifluoroacetic acid (0.1 mmol). The solution was then stirred for 0.5 h. After cooling to room temperature, the acetonitrile was removed under vacuum. The yellow solid from the prior step was dissolved in DCM (10 mL), followed by the addition of NH_4PF_6 (0.2 mmol). After stirring for 30 min, the compound Δ-**40** was purified via flash column chromatography (16.8mg, 90% yield, red solid).

Purification conditions: DCM/MeOH = 50:1 to 20:1, R_f (Δ-**40**) = 0.6 in DCM/MeOH (20:1).

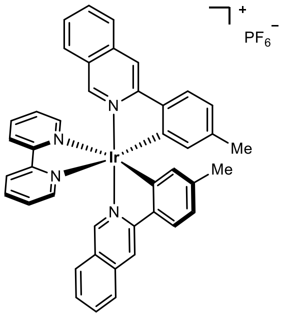

Analytical data of Δ-**40:**

^{1}H NMR (500 MHz, DMSO-d_6) δ 8.88 (d, J = 8.0 Hz, 2H), 8.69 (s, 2H), 8.52 (s, 2H), 8.30 – 8.23 (m, 2H), 8.09 – 8.01 (m, 4H), 7.93 (dd, J = 15.5, 8.0 Hz, 4H), 7.85 – 7.80 (m, 2H), 7.75 – 7.69 (m, 2H), 7.62 – 7.55 (m, 2H), 6.88 (dd, J = 8.0, 1.5 Hz, 2H), 5.99 (d, J = 1.5 Hz, 2H), 1.97 (s, 6H).

^{31}P NMR (202 MHz, DMSO) δ -133.61, -137.12, -140.63, -144.15, -147.67, -151.17, -154.68.

^{19}F NMR (471 MHz, DMSO) δ -69.32, -70.83.

^{13}C NMR (126 MHz, DMSO) δ 159.5, 155.5, 152.4, 149.6, 148.9, 141.1, 139.6, 138.4, 136.0, 133.0, 131.9, 128.5, 128.2, 127.5, 127.3, 126.5, 125.1, 123.8, 123.6, 115.2, 21.4.

HRMS (ESI) for $C_{42}H_{32}IrN_4$ $[M]^+$ calcd. 785.2256, found 785.2260

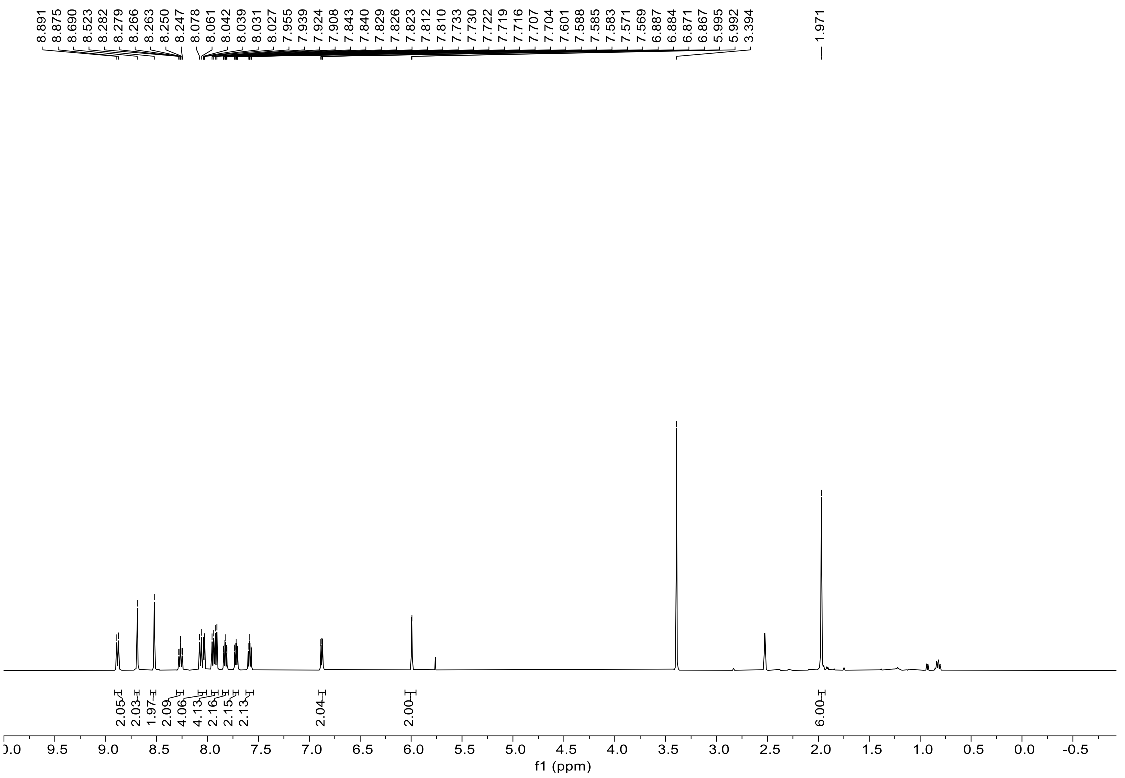

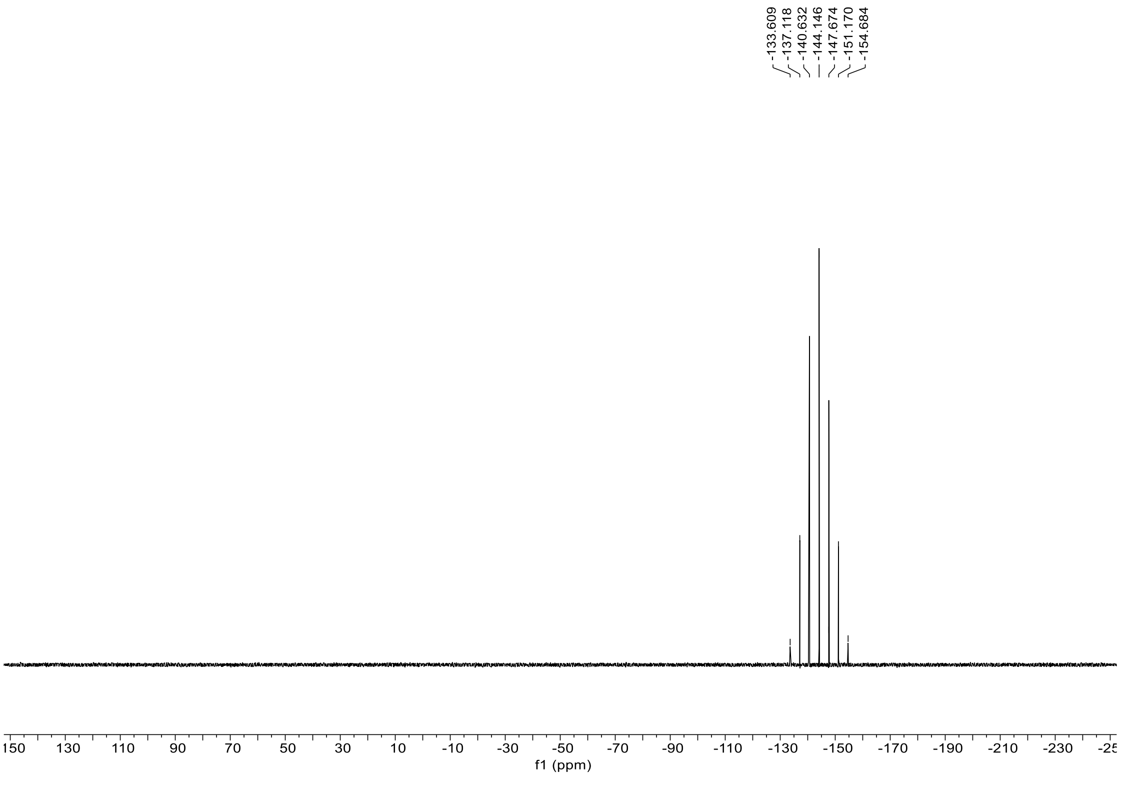

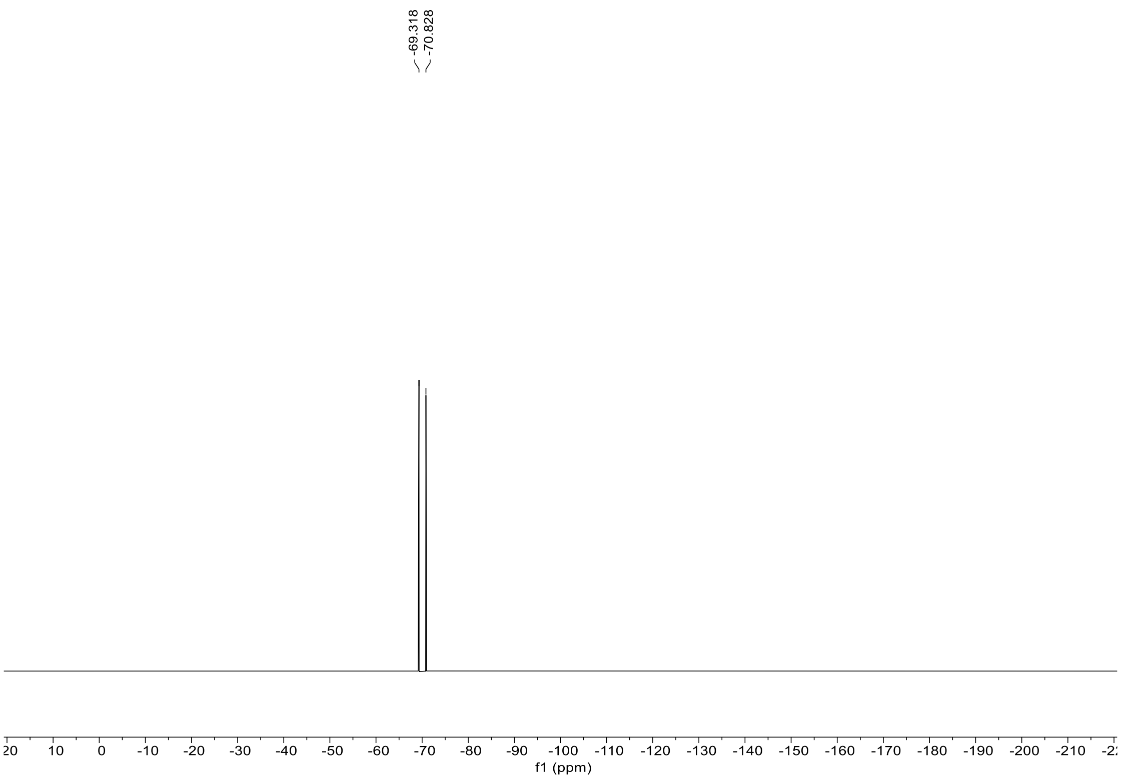
-69.318
-70.828
f1 (ppm)

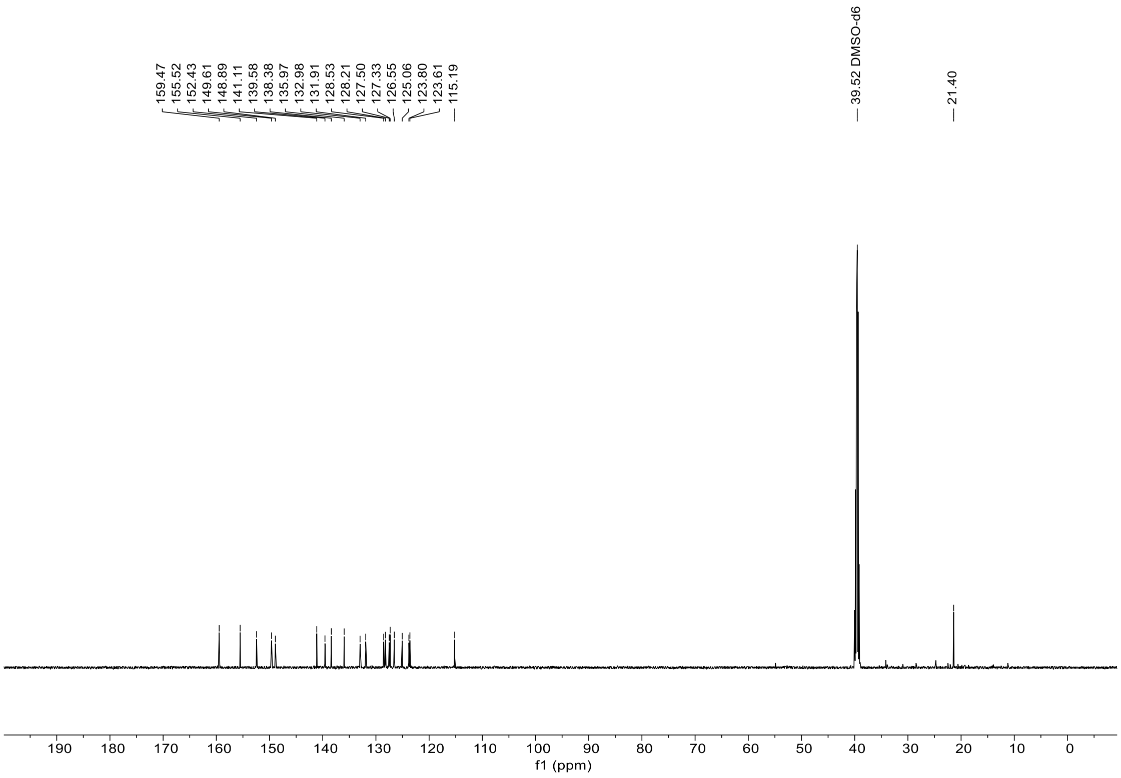
159.47
155.52
152.43
149.61
148.89
141.11
139.58
138.38
135.97
132.98
131.91
128.53
128.21
127.50
127.33
126.55
125.06
123.80
123.61
115.19
39.52 DMSO-d6
21.40
f1 (ppm)

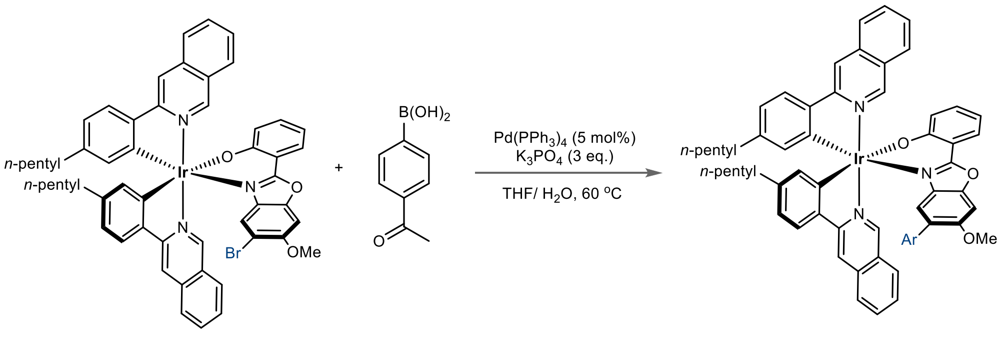

Λ-**28** **41**

A dried 10 mL Schlenk tube was charged with the Λ-**28** (0.02 mmol, 1.0 equiv.), (4-acetylphenyl)boronic acid (0.04 mmol, 2.0 equiv.), $Pd(PPh_3)_4$ (5 mol%), K_3PO_4 (0.06 mmol, 3.0 equiv.) and THF/H_2O (v/v, 9:1, 0.5 mL) in glovebox. The mixture was stirred at 60°C for 12 h. After achieving appropriate conversion, the mixture was concentrated under vacuum to give a residue, which was purified by column chromatography on silica gel to give the **41** (20.0 mg, 92% yield, >99% ee, orange solid).

Purification conditions: petroleum ether/EtOAc = 10:1 to 2:1, R_f (Λ-**41**) = 0.5 in petroleum ether/EtOAc (2:1).

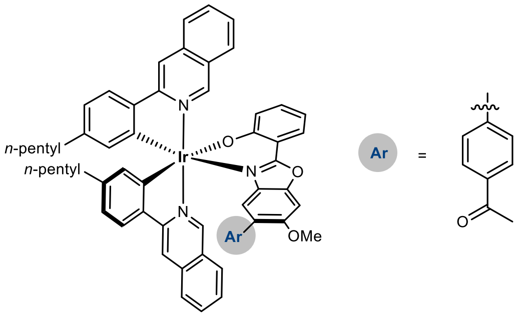

Analytical data of Λ-**41:**

Enantiomeric excess established by HPLC analysis using a Chiralpak IK-3 column, ee > 99% (HPLC: IK-3, 254 nm, *n*-hexane/isopropanol = 80:20, flow rate 1.0 mL/min, 40 °C, t_r (major) = 23.2 min.)

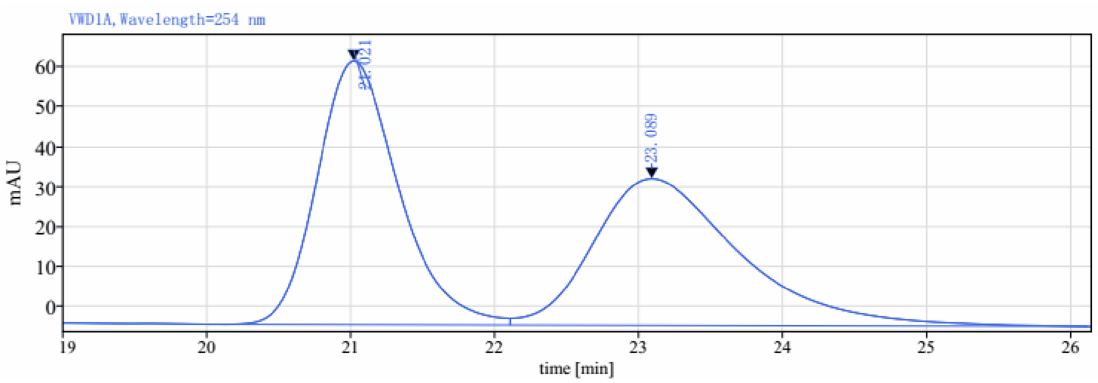

Rettime [min]	Type	Width [min]	Area	Height	Area%
21.021	BM m	1.99	2599.90	65.81	50.19
23.089	MM m	4.01	2579.89	36.61	49.81

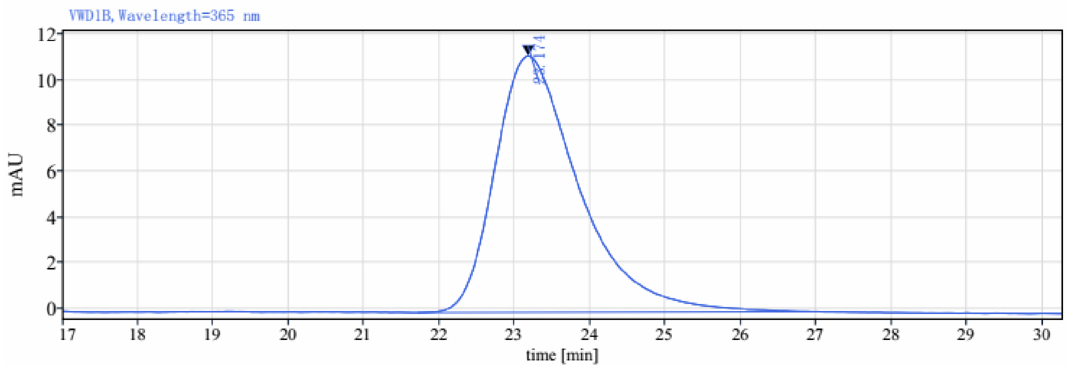

Rettime [min]	Type	Width [min]	Area	Height	Area%
23.174	BB	5.24	874.47	11.25	100.00

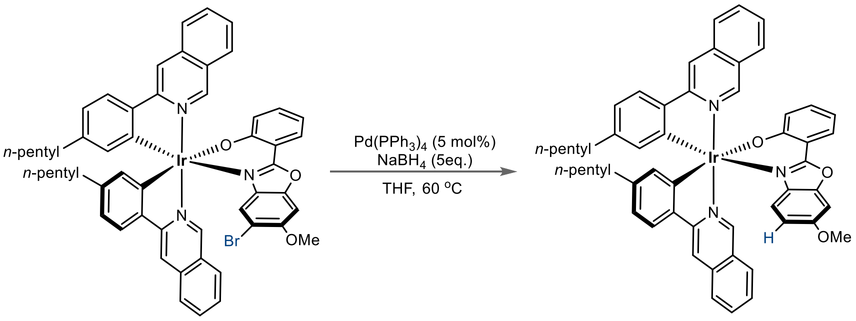

Λ-**28** **42**

A dried 10 mL Schlenk tube was charged with the Λ-**28** (0.02 mmol, 1.0 equiv.), $Pd(PPh_3)_4$ (5 mol%), $NaBH_4$ (0.10 mmol, 5 equiv.) and THF (0.5 mL) in glovebox. The mixture was stirred at 60°C for 3 h. After achieving appropriate conversion, the mixture was concentrated under

vacuum to give a residue, which was purified by flash column chromatography on silica gel to give the **42** (14.4 mg, 75% yield, 99% ee, orange solid).

Purification conditions: petroleum ether/EtOAc = 10:1 to 3:1.

R_f (Λ-**42**) = 0.5 in petroleum ether/EtOAc (3:1).

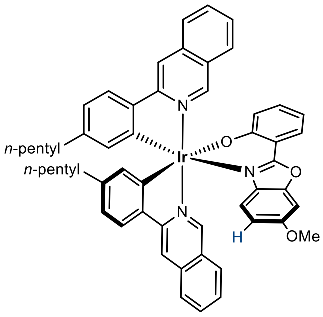

Analytical data of Λ-**42:**

^{1}H NMR (500 MHz, $CDCl_3$) δ 9.64 (s, 1H), 8.83 (s, 1H), 8.05 (s, 1H), 8.02 (s, 1H), 7.92 – 7.86 (m, 1H), 7.80 – 7.70 (m, 3H), 7.61 – 7.46 (m, 5H), 7.38 (t, *J* = 7.5 Hz, 1H), 7.33 – 7.26 (m, 1H), 7.16 – 7.07 (m, 1H), 6.94 (d, *J* = 2.5 Hz, 1H), 6.82 – 6.75 (m, 1H), 6.75 – 6.66 (m, 2H), 6.42 – 6.34 (m, 2H), 6.24 – 6.19 (m, 1H), 6.08 (t, *J* = 8.0 Hz, 1H), 5.95 (d, *J* = 8.5 Hz, 1H), 3.70 (s, 3H), 2.31 – 2.13 (m, 4H), 1.37 – 1.25 (m, 4H), 1.14 – 0.99 (m, 8H), 0.76 – 0.66 (m, 6H).

^{13}C NMR (126 MHz, $CDCl_3$) δ 169.5, 162.2, 161.8, 159.9, 157.6, 152.2, 152.1, 150.4, 149.5, 146.6, 143.2, 142.9, 142.6, 141.8, 136.5, 136.4, 135.0, 134.3, 133.1, 132.1, 131.6, 131.5, 128.5, 128.1, 127.54, 127.47, 127.4, 126.5, 126.45, 126.4, 126.3, 125.8, 123.3, 122.7, 122.0, 121.5, 119.3, 113.7, 113.5, 113.1, 111.9, 111.3, 95.4, 55.9, 35.9, 35.7, 31.5, 31.2, 30.9, 30.1, 22.6, 22.5, 14.11, 14.09.

HRMS (ESI) for $C_{54}H_{51}IrN_3O_3$ $[M+H]^+$ calcd.982.3554, found 982.3556

Enantiomeric excess established by HPLC analysis using a Chiralpak IK-3 column, ee = 99% (HPLC: IK-3, 254 nm, *n*-hexane/isopropanol = 80:20, flow rate 1.0 mL/min, 40 ^oC, t_r (major) = 7.5 min, t_r (minor) = 8.2 min.)

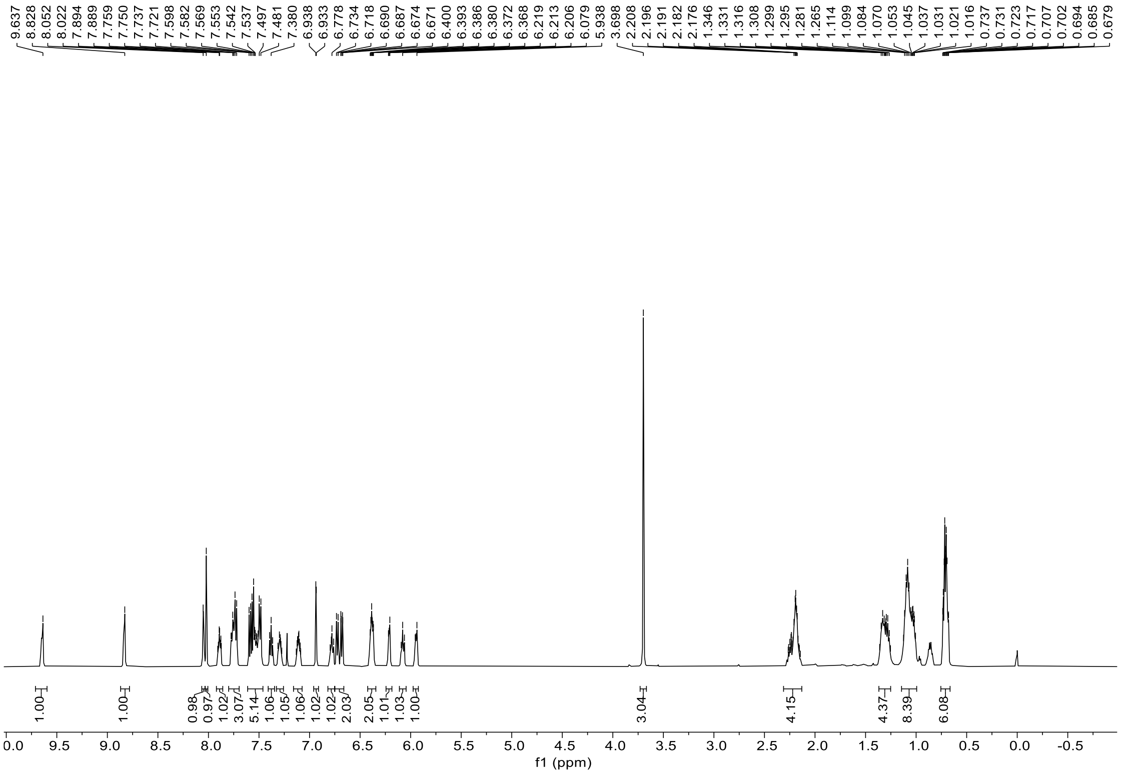
9.637
8.828
8.052
8.022
7.894
7.889
7.759
7.750
7.737
7.721
7.598
7.582
7.569
7.553
7.542
7.537
7.497
7.481
7.380
6.938
6.933
6.778
6.734
6.718
6.690
6.687
6.674
6.671
6.400
6.393
6.386
6.380
6.372
6.368
6.219
6.213
6.206
6.079
5.938
3.698
2.208
2.196
2.191
2.182
2.176
1.346
1.331
1.316
1.308
1.299
1.295
1.281
1.265
1.114
1.099
1.084
1.070
1.053
1.045
1.037
1.031
1.021
1.016
0.737
0.731
0.723
0.717
0.707
0.702
0.694
0.685
0.679
1.00
1.00
0.98
0.97
1.02
3.07
5.14
1.06
1.05
1.06
1.02
1.02
2.03
2.05
1.01
1.03
1.00
3.04
4.15
4.37
8.39
6.08
f1 (ppm)

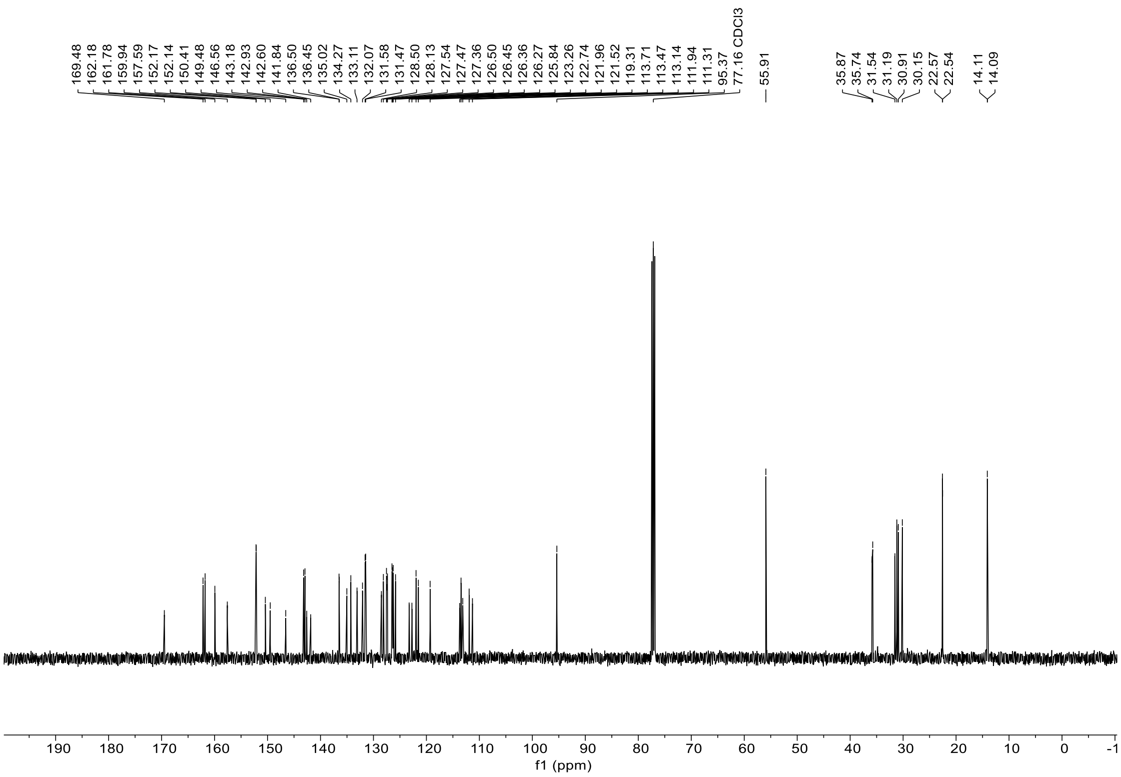
169.48
162.18
161.78
159.94
157.59
152.17
152.14
150.41
149.48
146.56
143.18
142.93
142.60
141.84
136.50
136.45
135.02
134.27
133.11
132.07
131.58
131.47
128.50
128.13
127.54
127.47
127.36
126.50
126.45
126.36
126.27
125.84
123.26
122.74
121.96
121.52
119.31
113.71
113.47
113.14
111.94
111.31
95.37
77.16 CDCl3
55.91
35.87
35.74
31.54
31.19
30.91
30.15
22.57
22.54
14.11
14.09
f1 (ppm)

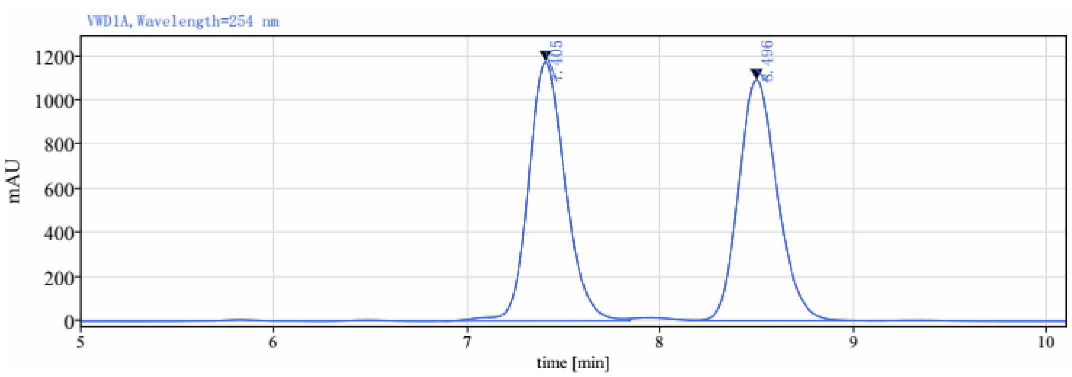

Rettime [min]	Type	Width [min]	Area	Height	Area%
7.405	MM m	0.96	15539.33	1172.88	50.59
8.496	MM m	1.14	15175.79	1089.88	49.41

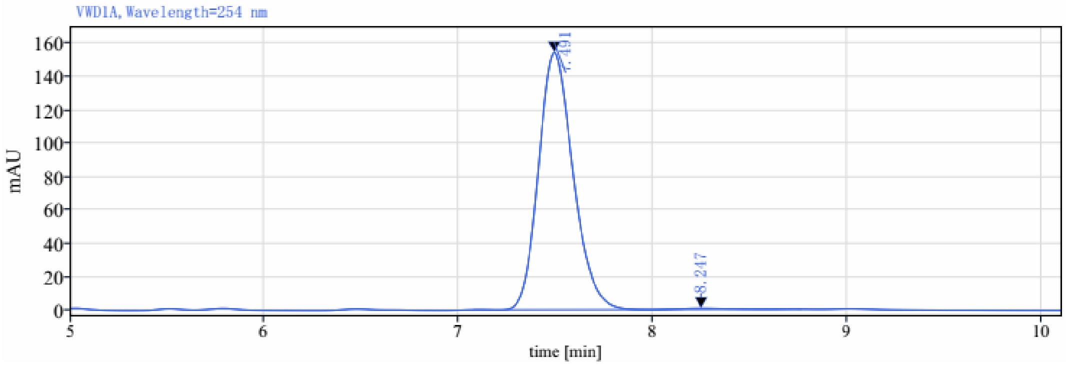

Rettime [min]	Type	Width [min]	Area	Height	Area%
7.491	MM m	0.86	1908.57	154.12	99.51
8.247	MM m	0.83	9.38	0.57	0.49

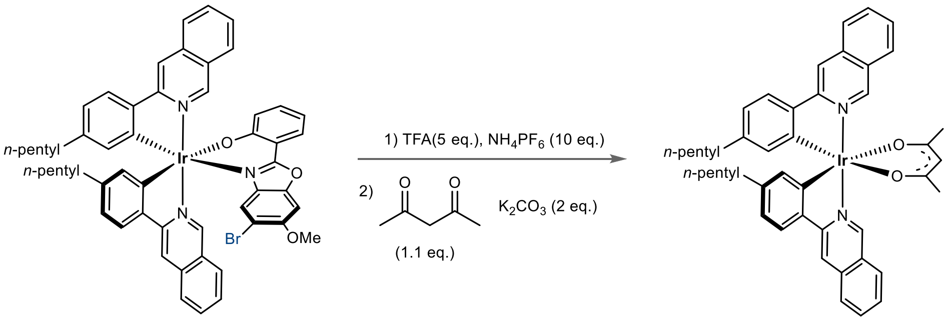

A solution of Λ-**28** (0.02 mmol) and NH_4PF_6 (0.2 mmol) in acetonitrile (5 mL) was sparged with N_2 for 5 min. Subsequently, trifluoroacetic acid (0.1 mmol) was added. The solution was

stirred for 0.5 h at room temperature, and then the acetonitrile was removed under reduced pressure. A flash chromatography column was run with a gradient from (DCM/MeCN = 100:1) to (DCM/MeCN = 10:1). The yellow solid from the prior step was dissolved in DCM (10 mL), followed by the addition of acetylacetone (0.022 mmol) and K_2CO_3 (0.2 mmol). After stirring for 30 min, the compound **43** was purified via flash column chromatography (petroleum ether/EtOAc = 10:1) (15.9 mg, 93% yield, 96% ee, red solid).

Purification conditions: petroleum ether/EtOAc = 10:1 to 5:1.

R_f (Λ-**43**) = 0.5 in petroleum ether/EtOAc (5:1).

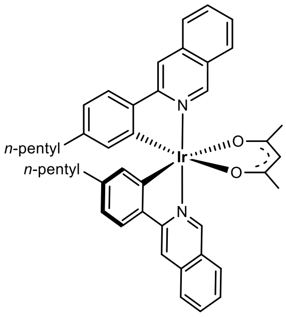

Analytical data of Λ-**43:**

^{1}H NMR (500 MHz, Chloroform-*d*) δ 9.28 – 9.20 (m, 2H), 8.09 (s, 2H), 7.92 – 7.84 (m, 4H), 7.69 – 7.62 (m, 2H), 7.56 (t, *J* = 7.0 Hz, 2H), 7.51 (t, *J* = 7.5 Hz, 2H), 6.69 – 6.60 (m, 2H), 6.05 – 5.94 (m, 2H), 5.27 – 5.17 (m, 1H), 2.19 (t, *J* =8.0 Hz, 4H), 1.85 – 1.76 (m, 6H), 1.37 – 1.25 (m, 4H), 1.15 – 1.01 (m, 8H), 0.72 (q, *J* = 7.5 Hz, 6H).

^{13}C NMR (126 MHz, $CDCl_3$) δ 184.7, 162.1, 151.3, 145.57, 145.55, 142.7, 142.25, 142.21, 136.8, 133.51, 133.49, 131.6, 127.8, 127.4, 126.69, 126.66, 126.44, 126.42, 122.7, 122.6, 121.5, 121.48, 113.2, 113.1, 100.64, 100.61, 35.9, 35.8, 31.63, 31.6, 30.32, 30.31, 29.08, 29.07, 22.58, 22.56, 14.13, 14.1.

HRMS (ESI) for $C_{45}H_{48}IrN_2O_2$ $[M+H]^+$ calcd.841.3340, found 841.3336

Enantiomeric excess established by HPLC analysis using a Chiralpak AD-3 column, ee = 96% (HPLC: AD-3, 254 nm, *n*-hexane/isopropanol = 90:10, flow rate 1.0 mL/min, 40 °C, t_r (major) = 7.7 min, t_r (minor) = 6.0 min.)

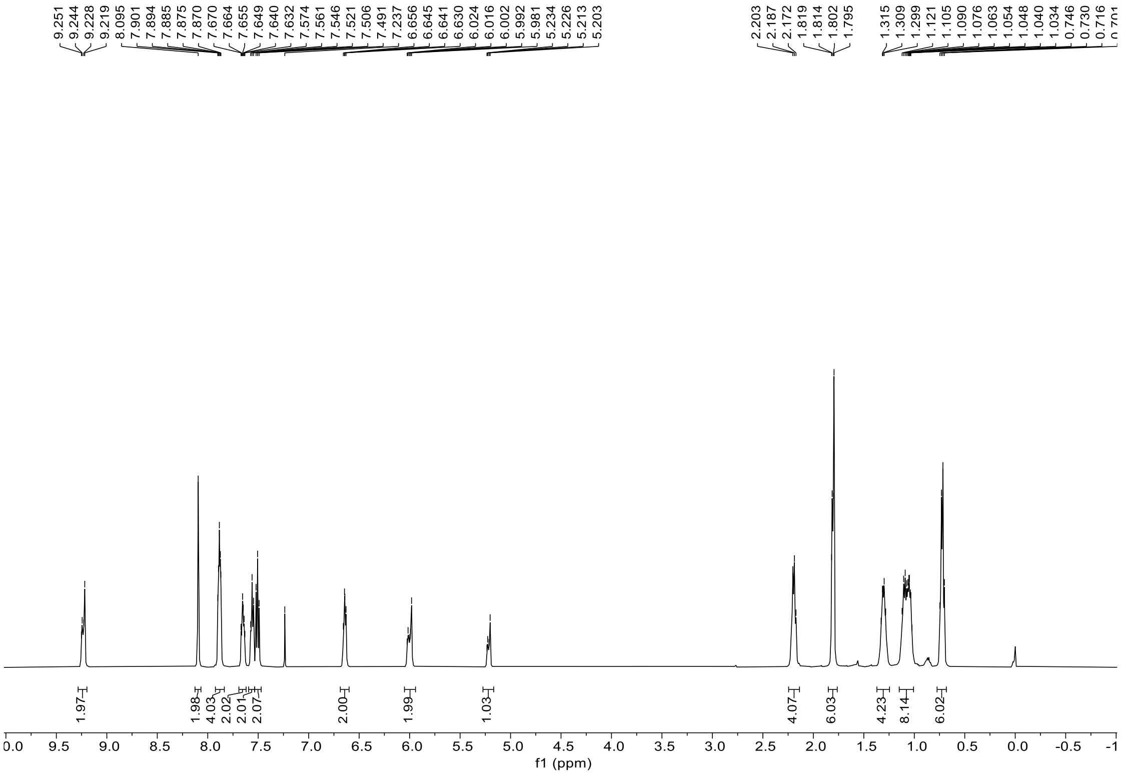
9.251
9.244
9.228
9.219
8.095
7.901
7.894
7.885
7.875
7.870
7.670
7.664
7.655
7.649
7.640
7.632
7.574
7.561
7.546
7.521
7.506
7.491
7.237
6.656
6.645
6.641
6.630
6.024
6.016
6.002
5.992
5.981
5.234
5.226
5.213
5.203
2.203
2.187
2.172
1.819
1.814
1.802
1.795
1.315
1.309
1.299
1.121
1.105
1.090
1.076
1.063
1.054
1.048
1.040
1.034
0.746
0.730
0.716
1.97
1.98
4.03
2.02
2.01
2.07
2.00
1.99
1.03
4.07
6.03
4.23
8.14
6.02
f1 (ppm)

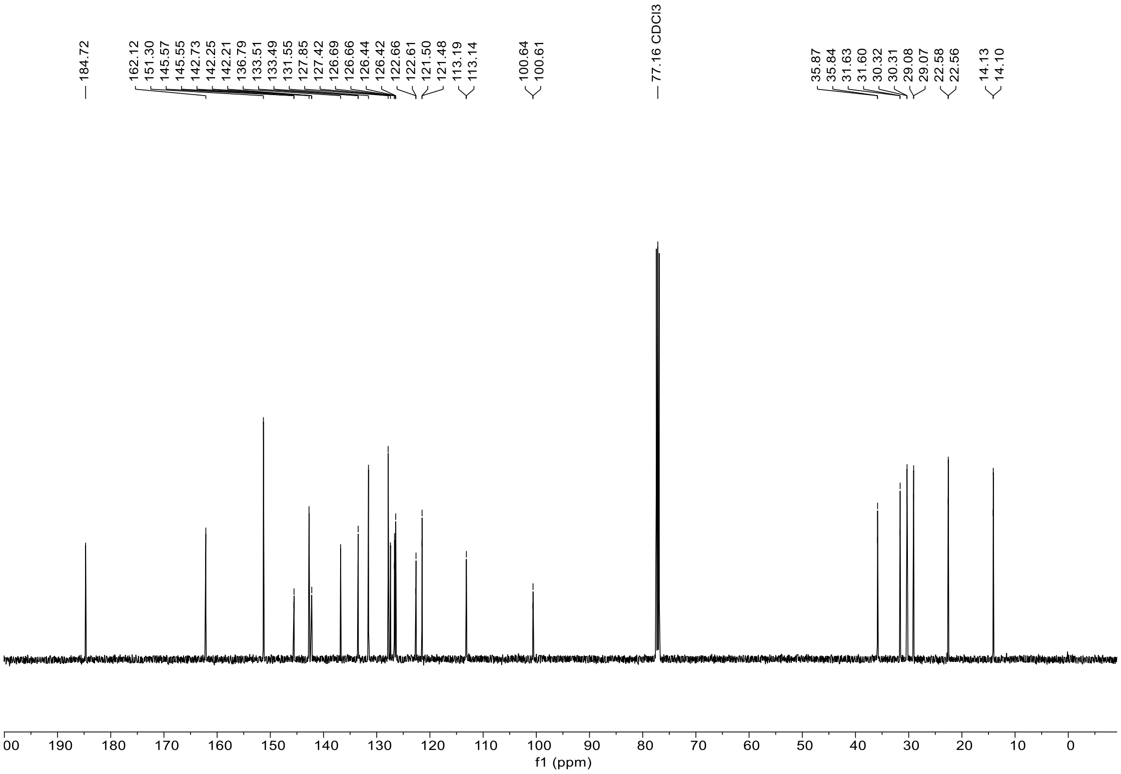
184.72
162.12
151.30
145.57
145.55
142.73
142.25
142.21
136.79
133.51
133.49
131.55
127.85
127.42
126.69
126.66
126.44
126.42
122.66
122.61
121.50
121.48
113.19
113.14
100.64
100.61
77.16 CDCl3
35.87
35.84
31.63
31.60
30.32
30.31
29.08
29.07
22.58
22.56
14.13
14.10
f1 (ppm)

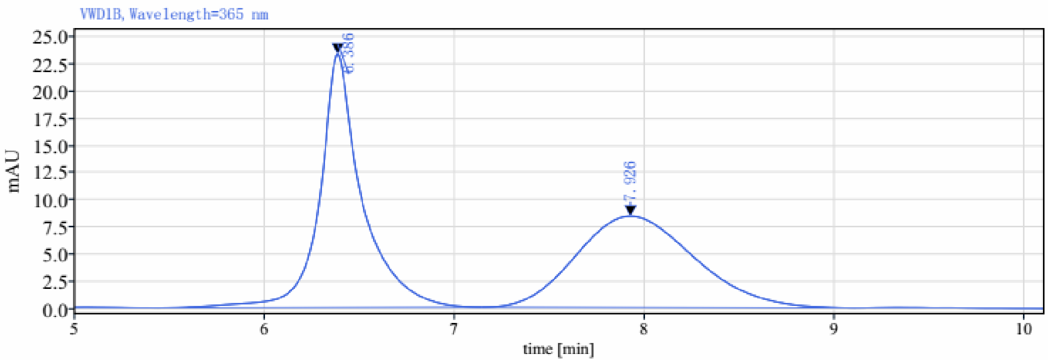

Rettime [min]	Type	Width [min]	Area	Height	Area%
6.386	BB	1.70	361.95	23.31	49.95
7.926	BB	1.97	362.69	8.40	50.05

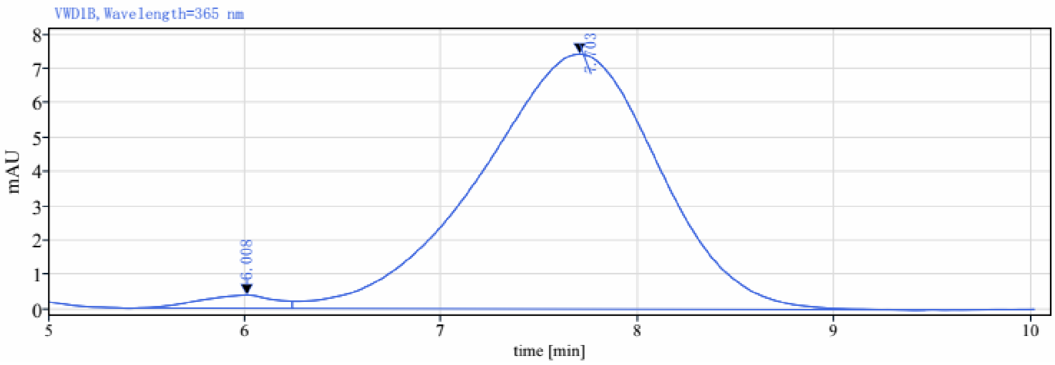

Rettime [min]	Type	Width [min]	Area	Height	Area%
6.008	MM m	0.78	10.15	0.38	2.12
7.703	MM m	3.22	469.26	7.42	97.88

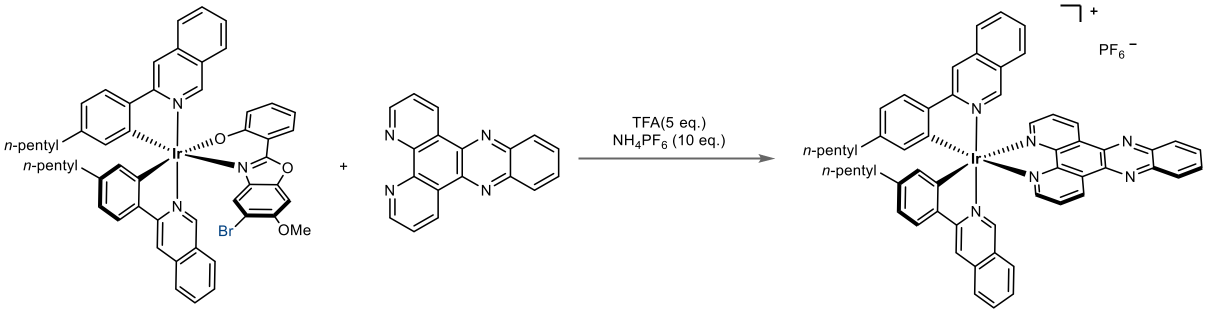

Λ-**28** **44**

A solution of Λ-**28** (0.02 mmol) and NH_4PF_6 (0.2 mmol) in acetonitrile (5 mL) was sparged with N_2 for 5 min. Subsequently, trifluoroacetic acid (0.1 mmol) was added. The solution was

stirred for 0.5 h at room temperature, and then the acetonitrile was removed under reduced pressure. The yellow solid from the prior step was dissolved in DCM (10 mL), followed by the addition of dipyrido[3,2-*a*:2',3'-*c*]phenazine (0.22 mmol) and NH_4PF_6 (0.2 mmol). After stirring for 30 min, the compound **44** was purified via flash column chromatography (CH_2Cl_2/MeOH = 100:1) (23.1 mg, 97% yield, red solid).

Purification conditions: DCM/MeOH = 100:1 to 30:1, R_f (Λ-**44**) = 0.5 in DCM/MeOH (30:1).

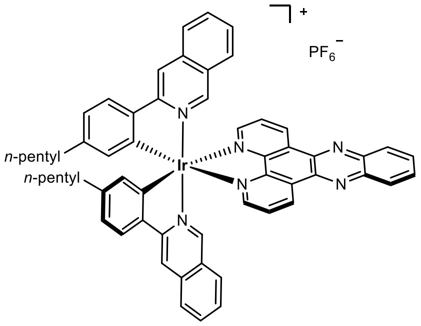

Analytical data of **44:**

^{1}H NMR (500 MHz, Methylene Chloride-d_2) δ 9.90 (d, J = 8.0 Hz, 2H), 8.59 (d, J = 5.5 Hz, 2H), 8.50 (d, J = 7.5 Hz, 2H), 8.34 (s, 2H), 8.26 (s, 2H), 8.14 – 7.82 (m, 8H), 7.71 (t, J = 7.5 Hz, 2H), 7.60 – 7.38 (m, 4H), 7.02 (d, J = 8.0 Hz, 2H), 6.22 (s, 2H), 2.37 (t, J = 8.0 Hz, 4H), 1.55 – 1.36 (m, 4H), 1.15 (s, 8H), 0.78 (d, J = 7.0 Hz, 6H).

^{31}P NMR (202 MHz, CD_2Cl_2) δ -133.94, -137.46, -140.97, -144.48, -147.99, -151.50, -155.01.

^{19}F NMR (471 MHz, CD_2Cl_2) δ -72.37, -73.88.

^{13}C NMR (126 MHz, CD_2Cl_2) δ 160.8, 152.5, 152.3, 149.8, 147.5, 145.3, 143.5, 141.6, 139.9, 137.0, 136.2, 133.3, 132.9, 132.0, 131.8, 130.2, 128.5, 128.1, 127.9, 127.7, 127.0, 124.2, 123.9, 115.7, 36.0, 31.8, 30.8, 22.7, 14.2.

HRMS (ESI) for $C_{58}H_{50}IrN_6$ $[M]^+$ calcd.1023.3726, found 1023.3740

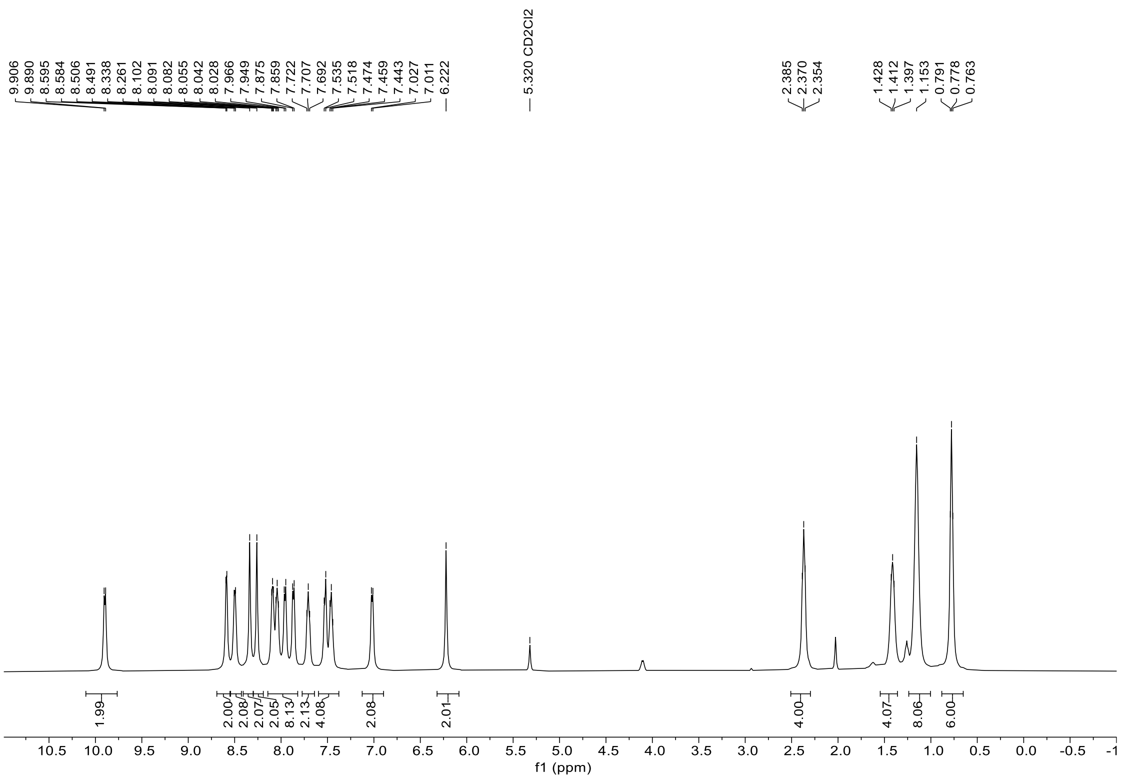
9.906
9.890
8.595
8.584
8.506
8.491
8.338
8.261
8.102
8.091
8.082
8.055
8.042
8.028
7.966
7.949
7.875
7.859
7.722
7.707
7.692
7.535
7.518
7.474
7.459
7.443
7.027
7.011
6.222
5.320 CD2Cl2
2.385
2.370
2.354
1.428
1.412
1.397
1.153
0.791
0.778
0.763
1.99
2.00
2.08
2.07
2.05
8.13
2.13
4.08
2.08
2.01
4.00
4.07
8.06
6.00
10.5 10.0 9.5 9.0 8.5 8.0 7.5 7.0 6.5 6.0 5.5 5.0 4.5 4.0 3.5 3.0 2.5 2.0 1.5 1.0 0.5 0.0 -0.5
f1 (ppm)

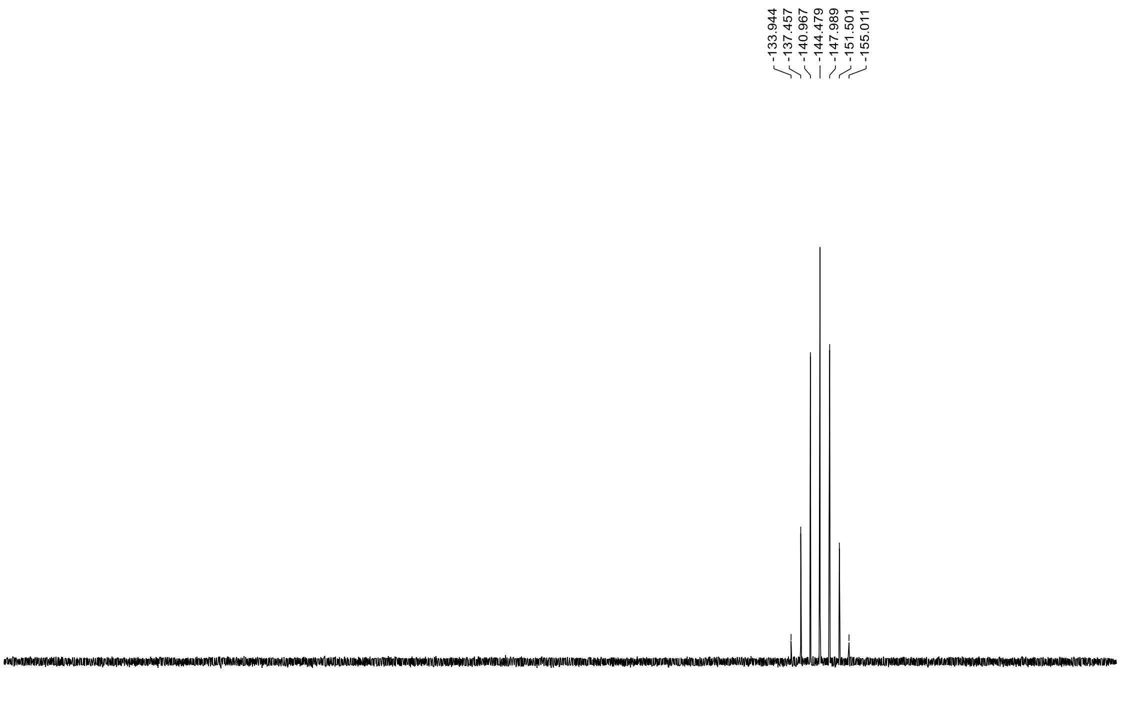
-133.944
-137.457
-140.967
-144.479
-147.989
-151.501
-155.011
150 130 110 90 70 50 30 10 -10 -30 -50 -70 -90 -110 -130 -150 -170 -190 -210 -230
f1 (ppm)

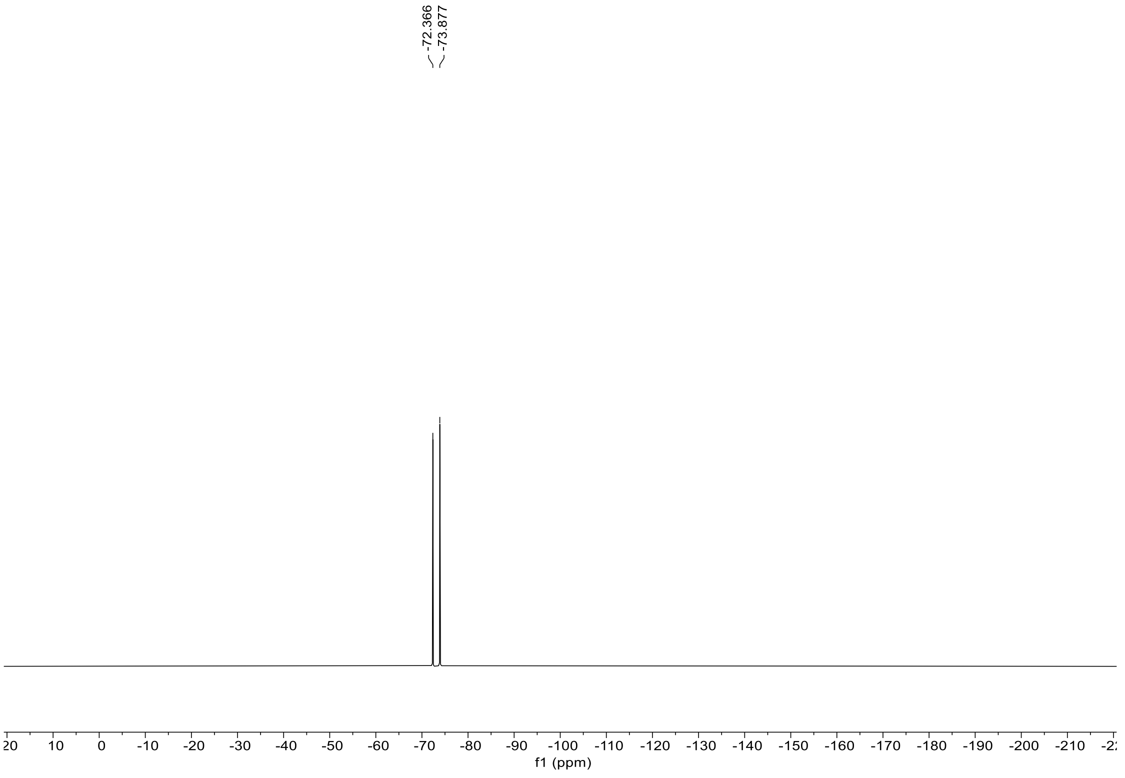

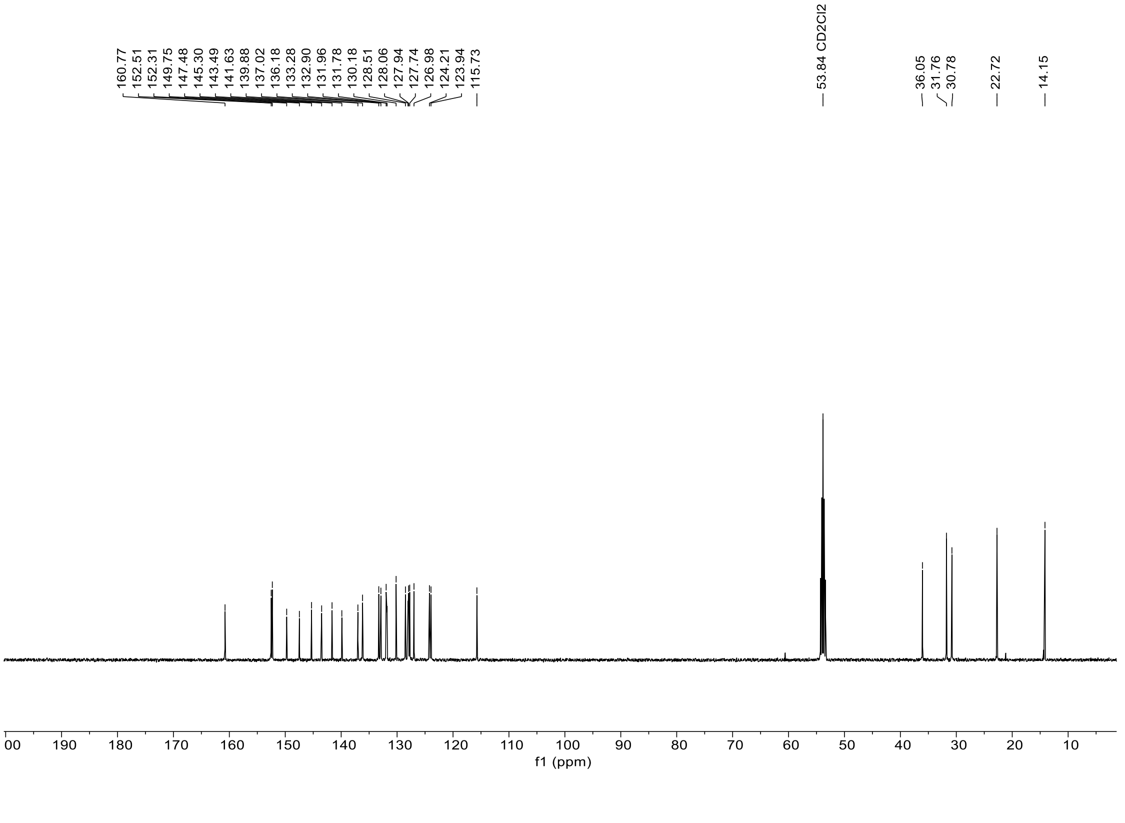

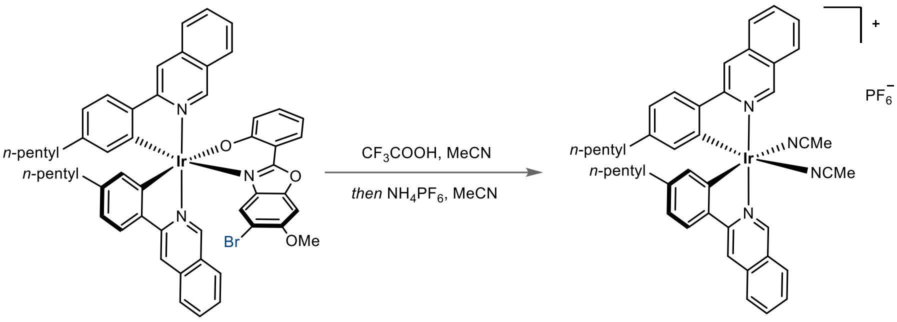

Λ-28 45

The compound **45** *was prepared according to the modified procedure*[11]

To a solution of **Λ-28** (0.02 mmol, 1.0 equiv.) dissolved in MeCN (5 mL), CF_3COOH (0.1 mmol, 5.0 equiv.) was gradually added in a dropwise manner. The reaction mixture was maintained at 25 °C and stirred for 30 minutes. Subsequently, the volatile components were removed under vacuum using a rotary evaporator. The resulting yellow oil was then dissolved in 10 ml of CH_3CN. NH_4PF_6 (0.4 mmol, 20 equiv.) was added in a single portion, and the mixture was stirred at room temperature for an additional 30 minutes. Following this, the MeCN was removed under vacuum. Finally, the compound **45** was purified through flash column chromatography (90% yield, yellow solid).

Purification conditions: DCM/MeCN = 100:1 to 10:1, R_f (Λ-**45**) = 0.5 in DCM/MeCN (20:1).

^{1}H NMR (500 MHz, Acetonitrile-d_3) δ 9.81 (s, 2H), 8.32 (s, 2H), 8.26 – 8.20 (m, 2H), 8.05 – 7.99 (m, 2H), 7.85 – 7.79 (m, 2H), 7.73 – 7.67 (m, 2H), 7.54 (d, *J* = 8.0 Hz, 2H), 6.63 (dd, *J* = 8.0, 1.5 Hz, 2H), 5.77 (d, *J* = 2.0 Hz, 2H), 2.07 (t, *J* = 7.5 Hz, 4H), 1.18 – 1.08 (m, 4H), 1.02 – 0.94 (m, 4H), 0.94 – 0.86 (m, 4H), 0.64 (t, *J* = 7.5 Hz, 6H).

^{13}C NMR (126 MHz, CD_3CN) δ 160.7, 155.6, 144.2, 143.04, 142.99, 137.8, 134.0, 132.1, 129.1, 128.8, 127.7, 124.2, 124.0, 115.9, 36.1, 31.9, 31.1, 23.0, 14.3.

^{31}P NMR (202 MHz, CD_3CN) δ -134.04, -137.53, -141.02, -144.52, -147.97, -148.01, -151.50, -154.99.

^{19}F NMR (471 MHz, CD_3CN) δ -71.86, -73.36.

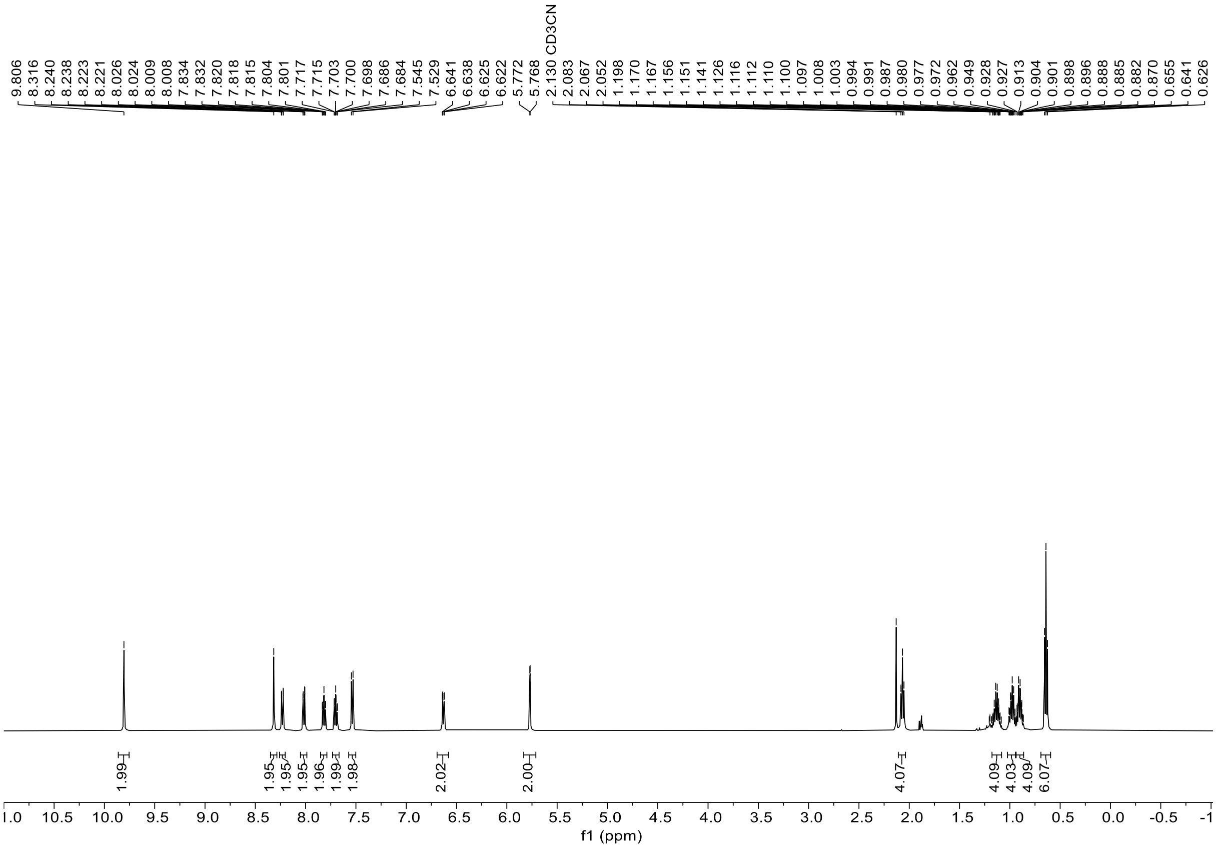
9.806
8.316
8.240
8.238
8.223
8.221
8.026
8.024
8.009
8.008
7.834
7.832
7.820
7.818
7.815
7.804
7.801
7.717
7.715
7.703
7.700
7.698
7.686
7.684
7.545
7.529
6.641
6.638
6.625
6.622
5.772
5.768
2.130 CD3CN
2.083
2.067
2.052
1.198
1.170
1.167
1.156
1.151
1.141
1.126
1.116
1.112
1.110
1.100
1.097
1.008
1.003
0.994
0.991
0.987
0.980
0.977
0.972
0.962
0.949
0.928
0.927
0.913
0.904
0.901
0.898
0.896
0.888
0.885
0.882
0.870
0.655
0.641
0.626
1.99
1.95
1.95
1.95
1.96
1.99
1.98
2.02
2.00
4.07
4.09
4.03
4.09
6.07
f1 (ppm)

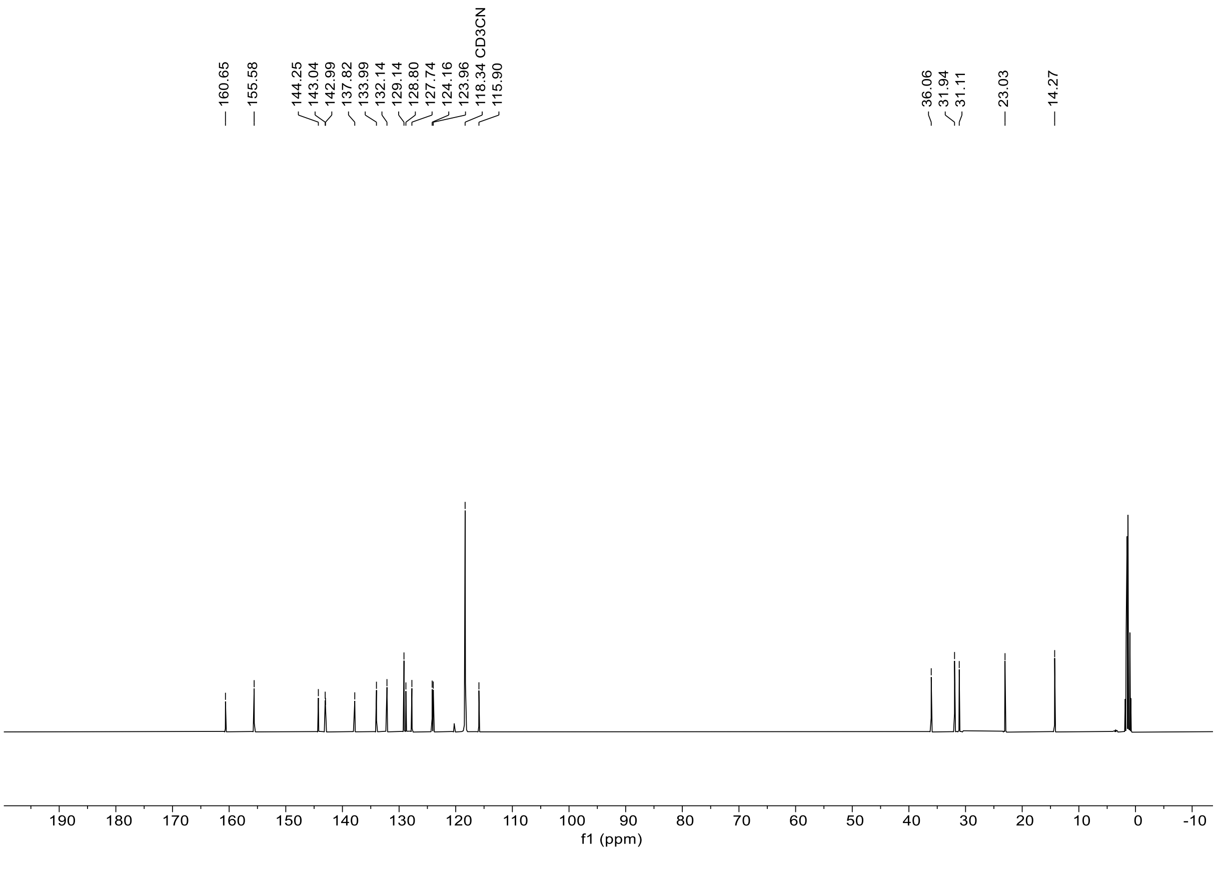
160.65
155.58
144.25
143.04
142.99
137.82
133.99
132.14
129.14
128.80
127.74
124.16
123.96
118.34 CD3CN
115.90
36.06
31.94
31.11
23.03
14.27
f1 (ppm)

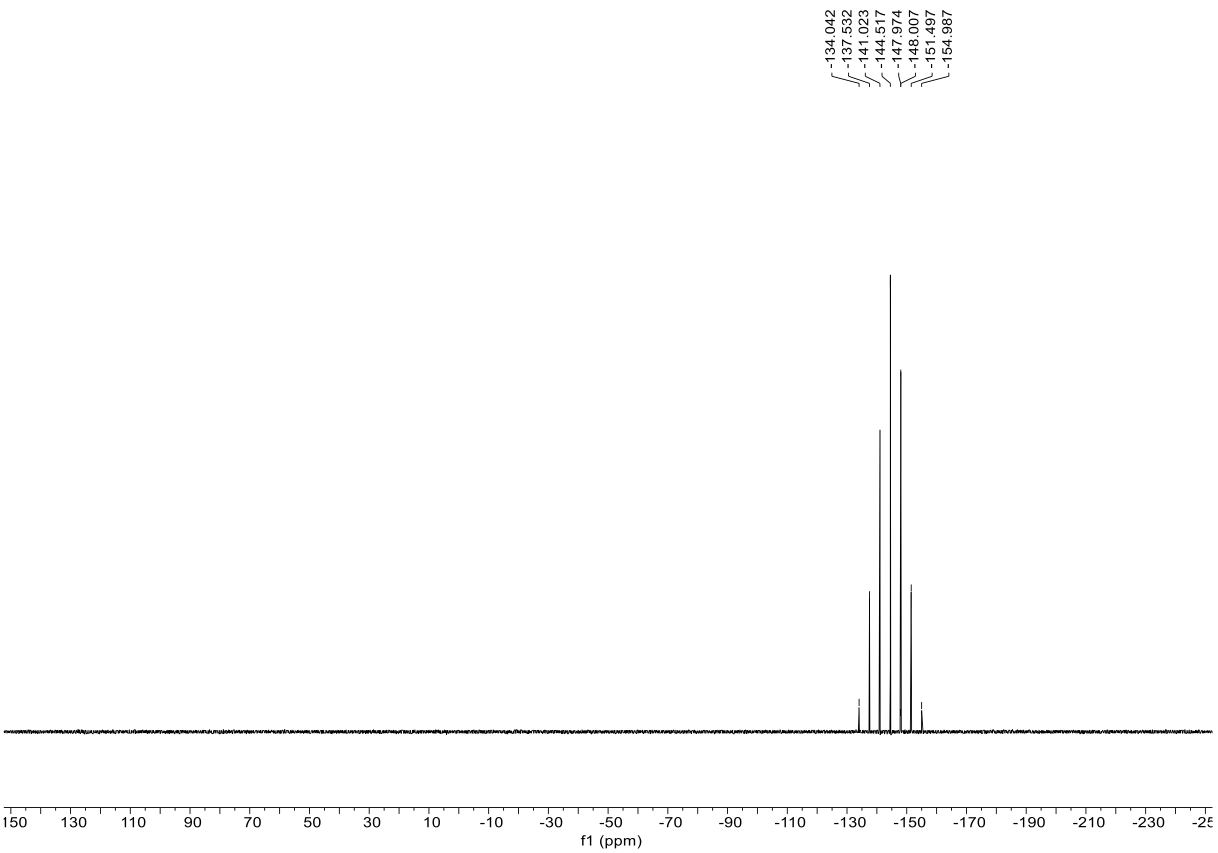
-134.042
-137.532
-141.023
-144.517
-147.974
-148.007
-151.497
-154.987
150 130 110 90 70 50 30 10 -10 -30 -50 -70 -90 -110 -130 -150 -170 -190 -210 -230
f1 (ppm)

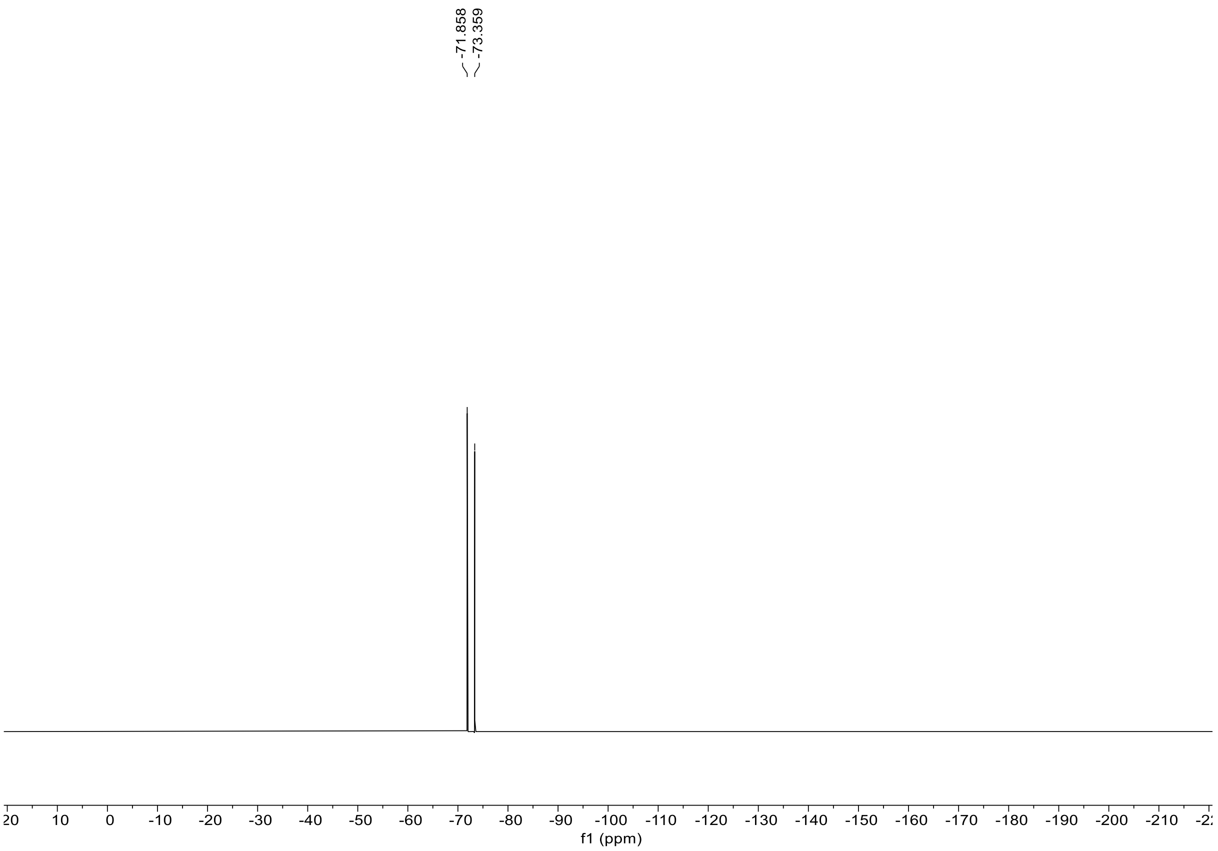
-71.858
-73.359
20 10 0 -10 -20 -30 -40 -50 -60 -70 -80 -90 -100 -110 -120 -130 -140 -150 -160 -170 -180 -190 -200 -210
f1 (ppm)

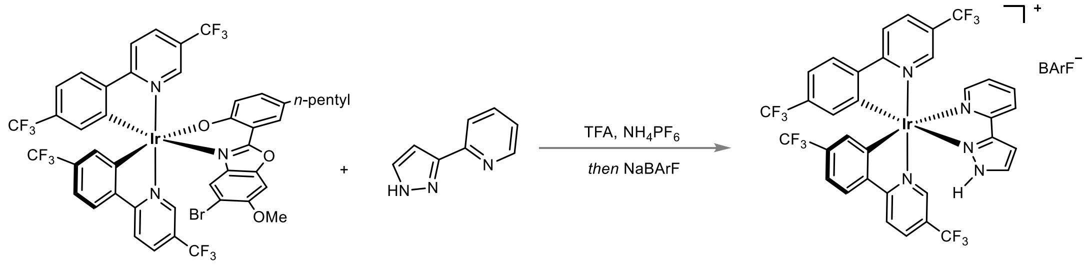

Λ-**20** **46**

The compound ***46*** *was prepared according to the reported procedure*[12].

To a flame-dried 25 mL round bottom flask were added 2-(1H-pyrazol-3-yl)pyridine (1.3 equiv. 0.026 mmol), ammonium hexafluorophosphate (10 equiv., 0.2 mmol), and **Λ-20** (0.02 mmol). Acetonitrile (10 mL) was then added and the reaction sparged with N_2 for 5 min followed by the addition of trifluoroacetic acid (10 equiv., 0.2 mmol). The solution was then heated to 60 °C under N_2 for 5 h. After cooling to room temperature, the acetonitrile was removed under vacuum. A short flash chromatography column was run with a gradient from 1:1 petroleum ether/EtOAc to EtOAc. The resulting yellow solid was carried further without purification. The yellow solid from the prior step was dissolved in DCM (10 mL), followed by the addition of NaBArF (1.1 equiv., 0.022 mmol). After stirring for 30 min, the reaction was extracted with H_2O (5×5 mL). The DCM was subsequently dried with Na_2SO_4, filtered, and the DCM removed under vacuum. The compound **46** was purified via flash column chromatography, CH_2Cl_2 eluant, and isolated as a bright yellow-green powder in 90% yield.

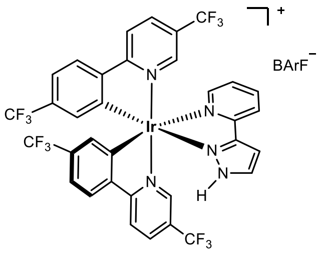

Analytical data of **46:**

^{1}H NMR (400 MHz, DMSO-d_6) δ 14.23 (s, 1H), 8.73 (d, *J* = 8.8 Hz, 2H), 8.61 – 8.46 (m, 3H), 8.38 – 8.20 (m, 4H), 7.77 (s, 1H), 7.74 – 7.54 (m, 15H), 7.46 – 7.37 (m, 2H), 6.35 (d, *J* = 2.0 Hz, 1H), 6.26 (d, *J* = 1.6 Hz, 1H).

The spectroscopic properties were consistent with the data available in the literature[12]

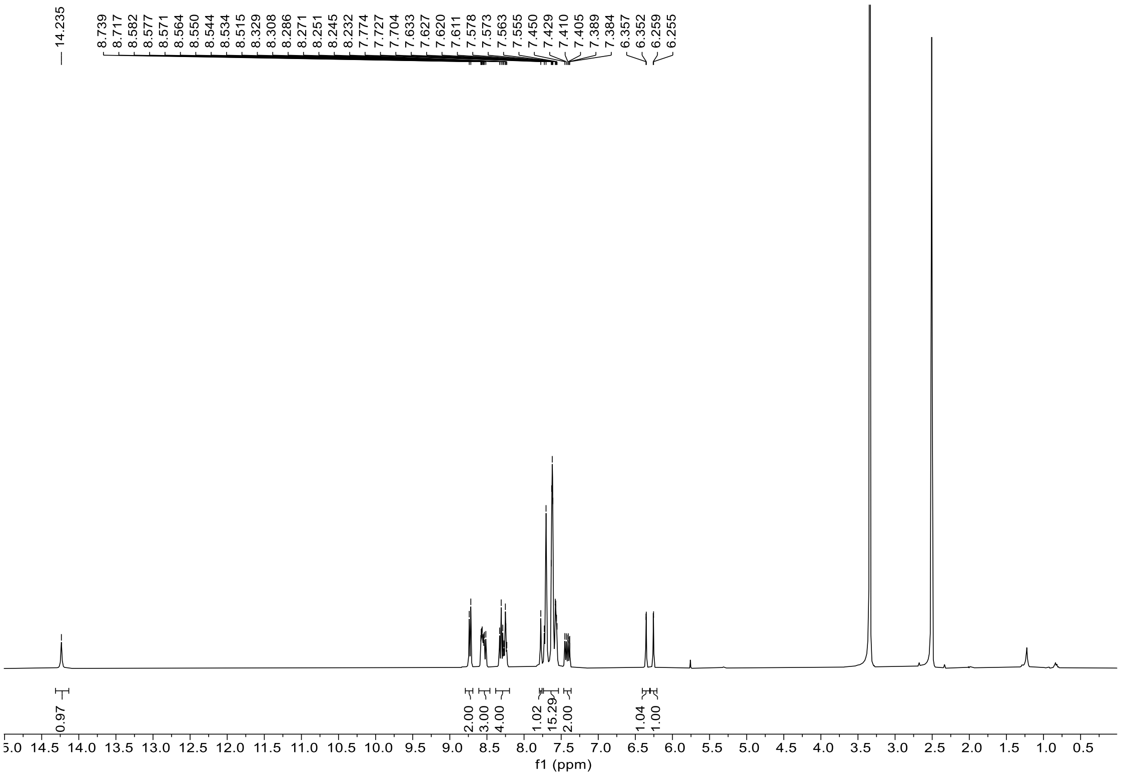

14.235
8.739
8.717
8.582
8.577
8.571
8.564
8.550
8.544
8.534
8.515
8.329
8.308
8.286
8.271
8.251
8.245
8.232
7.774
7.727
7.704
7.633
7.627
7.620
7.611
7.578
7.573
7.563
7.555
7.450
7.429
7.410
7.405
7.389
7.384
6.357
6.352
6.259
6.255
0.97
2.00
3.00
4.00
1.02
15.29
2.00
1.04
1.00
f1 (ppm)

6. High Temperature NMR Experiment of Compound 31

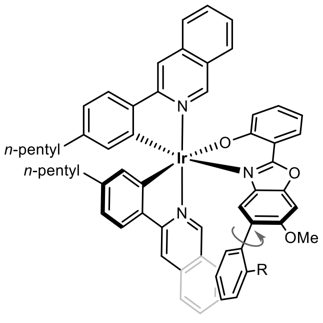

R = Acetyl

The compound **31** simultaneously possesses stable metal-centered chirality and low-rotation-energy-barrier biaryl axial chirality. Therefore, relatively disordered NMR spectra are observed in the NMR experiment at 298k. When the NMR experimental temperature is increased, the biaryl axial chirality with a low rotation energy barrier rotates rapidly, so the clear NMR spectrum can be obtained in the NMR experiment at 383K.

^{1}H NMR spectrum of **31** (500 MHz, $C_2D_2Cl_4$)

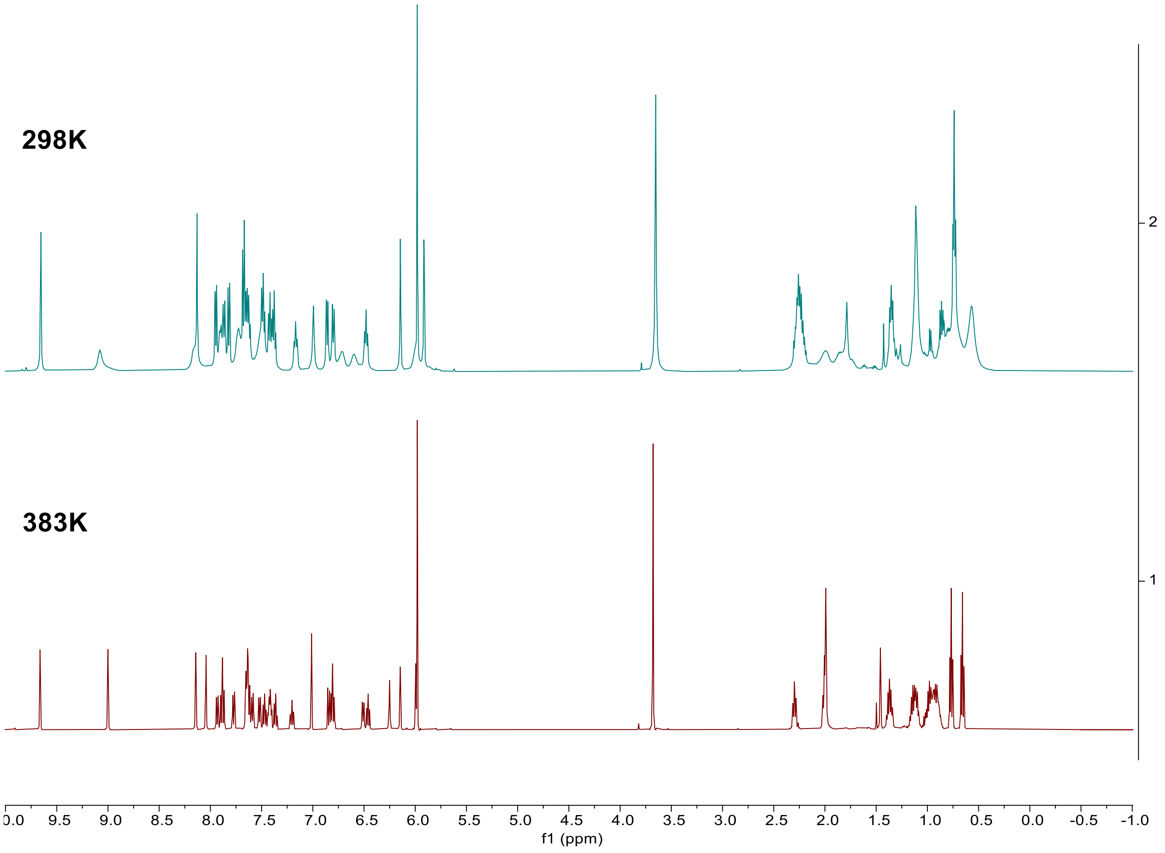

^{13}C NMR spectrum of **31** (126 MHz, $C_2D_2Cl_4$)

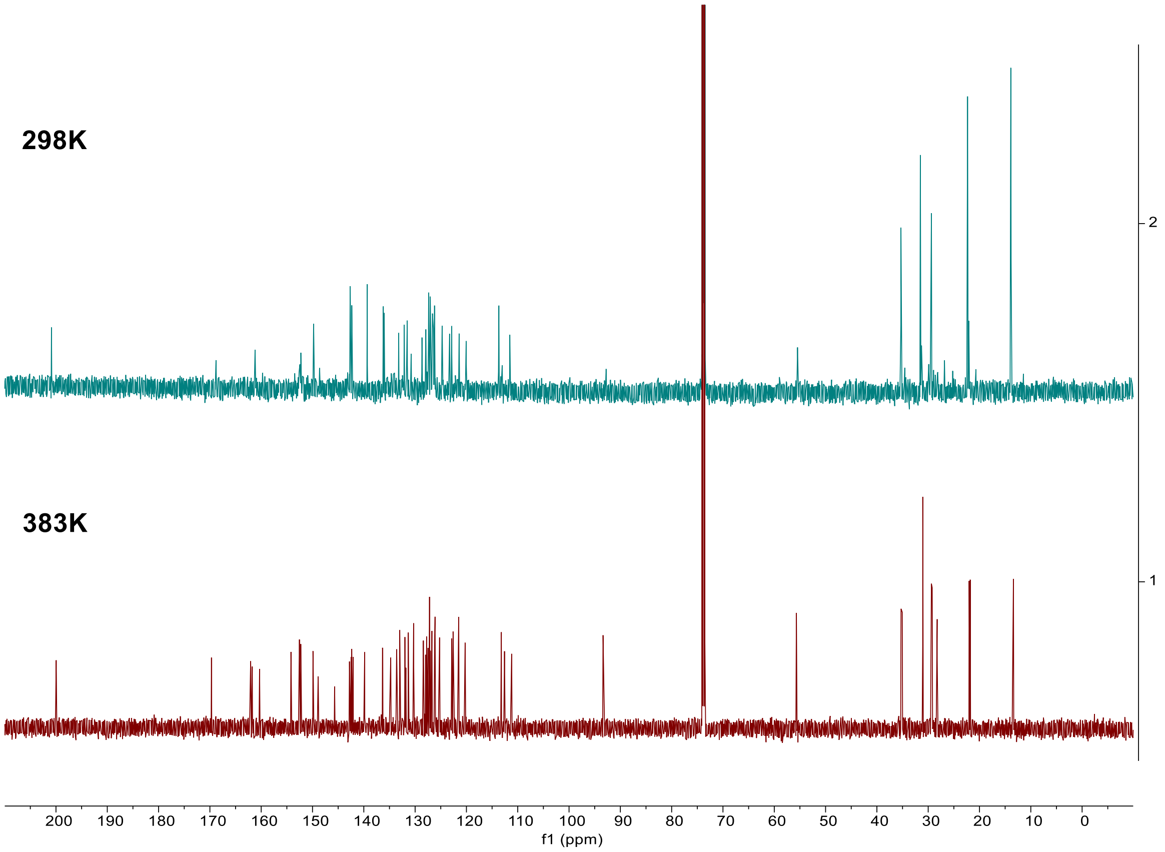

7. Study of Coordination Stability of Rh-25

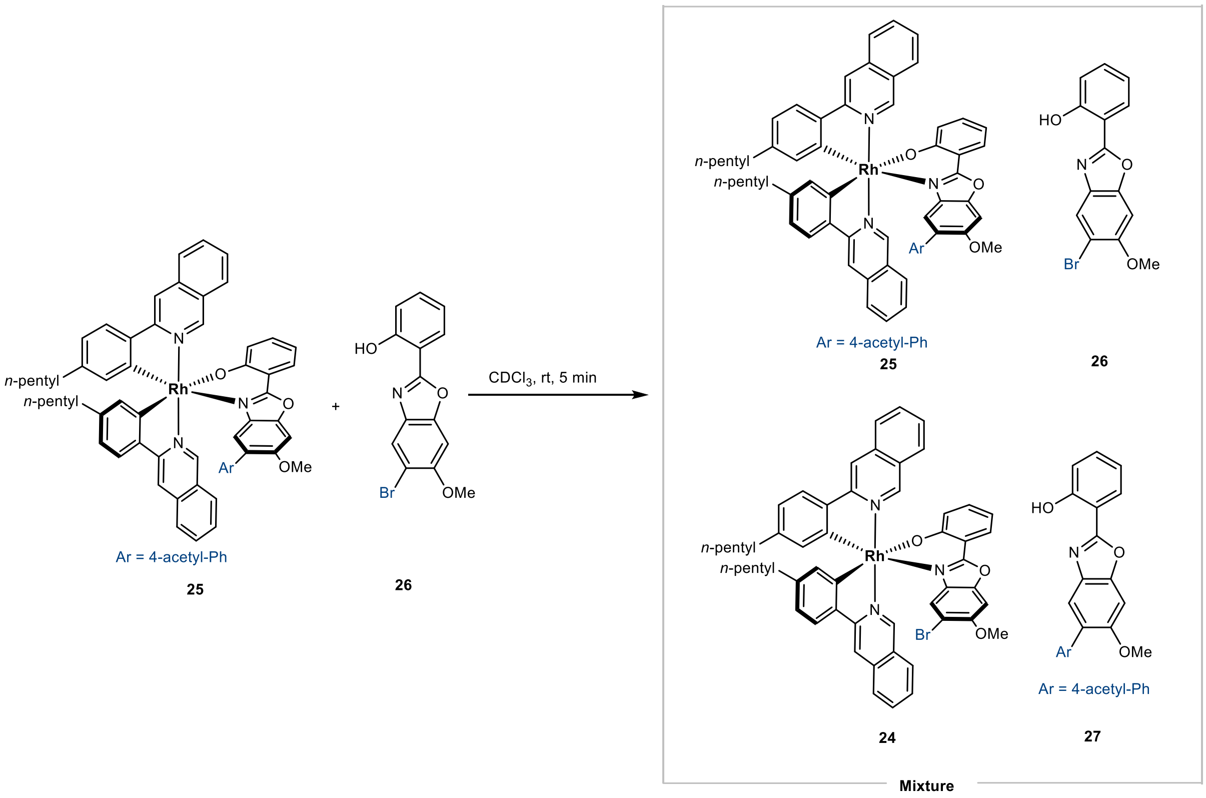

The compound **Rh-25** (0.5 equiv.) was added to the NMR tube containing compound **26** (0.015 mmol, 0.6 mL $CDCl_3$), and left at room temperature for 5 minutes before conducting the NMR experiment. The characteristic peaks of compounds **Rh-25**, **26**, **Rh-24**, and **27** were simultaneously observed in the ^1H NMR spectrum. The experimental results indicate that compound **Rh-25** can undergo rapid ligand exchange.

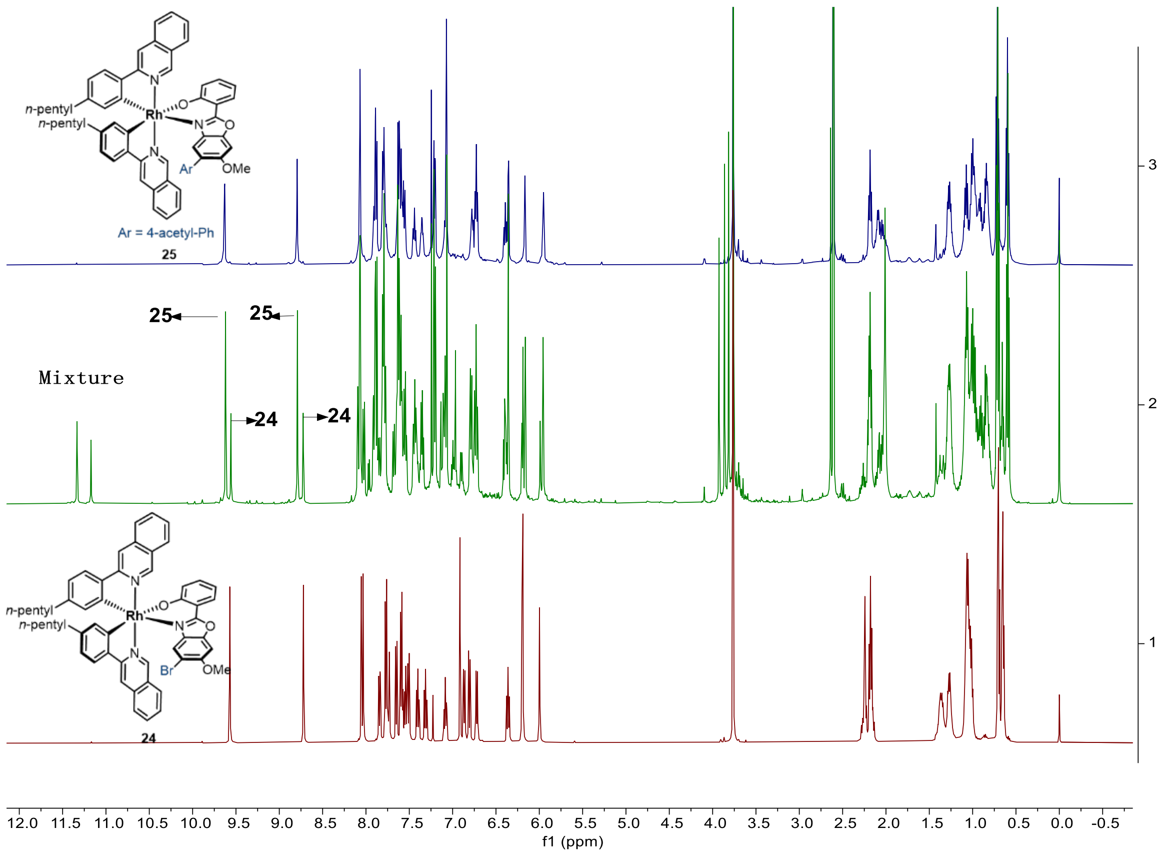
n-pentyl
n-pentyl
Rh
Ar
OMe
Ar = 4-acetyl-Ph
25
Mixture
25
25
24
24
Br
OMe
24
12.0 11.5 11.0 10.5 10.0 9.5 9.0 8.5 8.0 7.5 7.0 6.5 6.0 5.5 5.0 4.5 4.0 3.5 3.0 2.5 2.0 1.5 1.0 0.5 0.0 -0.5
f1 (ppm)

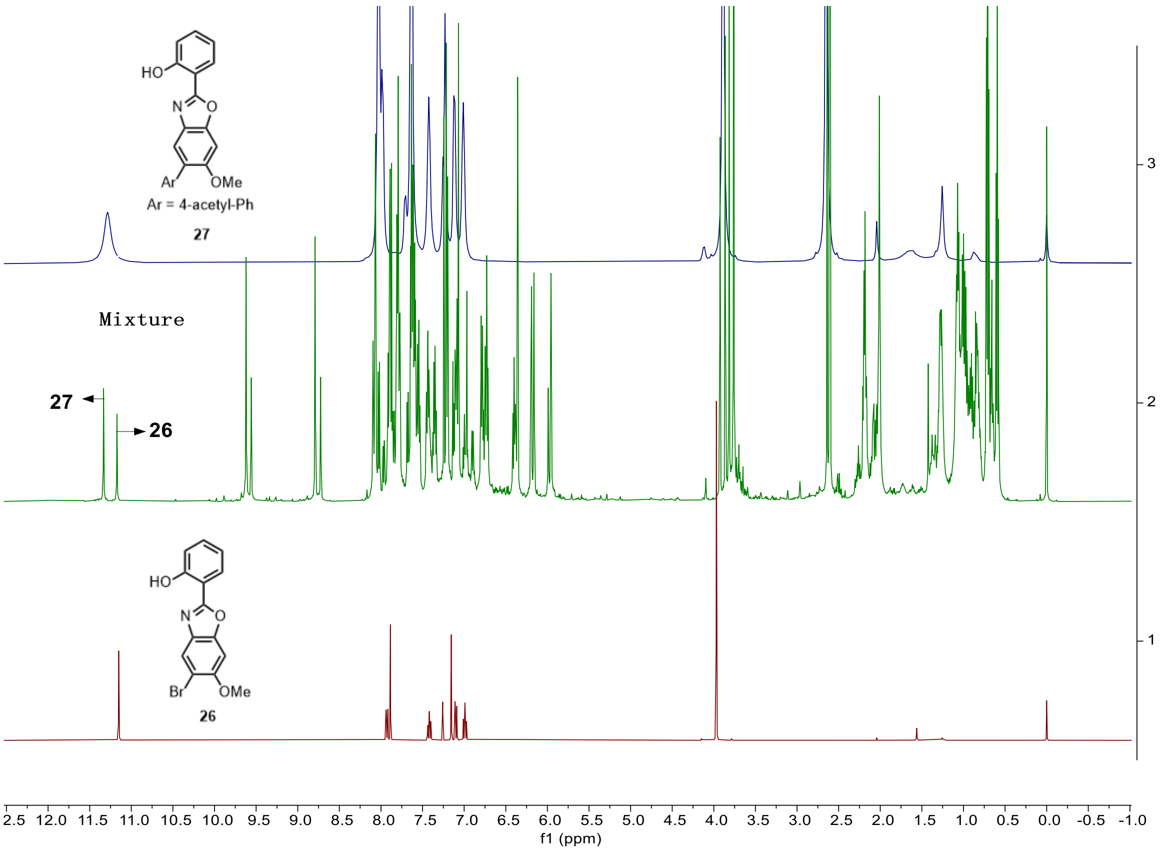
HO
Ar
OMe
Ar = 4-acetyl-Ph
27
Mixture
27
26
HO
Br
OMe
26
2.5 12.0 11.5 11.0 10.5 10.0 9.5 9.0 8.5 8.0 7.5 7.0 6.5 6.0 5.5 5.0 4.5 4.0 3.5 3.0 2.5 2.0 1.5 1.0 0.5 0.0 -0.5 -1.0
f1 (ppm)

8. Kinetic Experiments

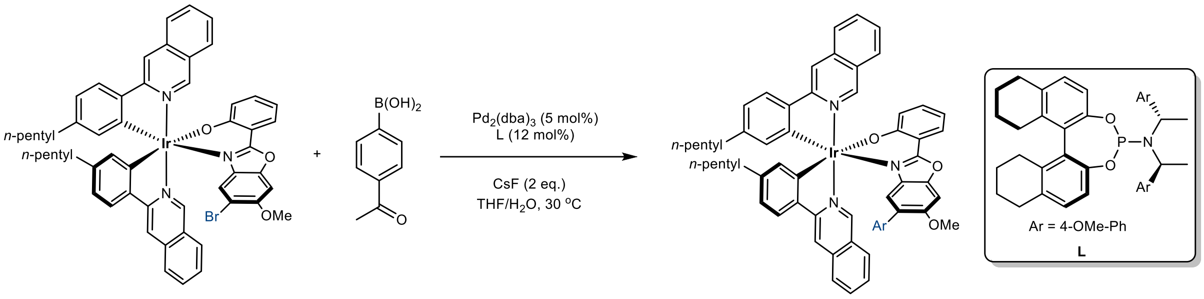

General procedure for kinetic experiments:

A dried 4 mL vial was charged with the **28** (0.003 mmol – 0.012 mmol, 0.3 – 1.2 equiv.), arylboronic acid **2a** (0.003 mmol – 0.012 mmol, 0.3 – 1.2 equiv.), **cat.** (3 – 12 mol%), CsF (0.02 mmol, 3.04 mg, 2 equiv.) and THF/H_2O (v/v, 9:1, 0.4 mL, 0.025 M) in glovebox. The mixture was stirred at 30°C for 12 h, and aliquots of the reaction mixture (20 μL) was added to isopropanol (980 μL), and the reaction conversion rate was monitored by HPLC peak area of product **29** (HPLC: IK-3, 254 nm, *n*-hexane/isopropanol = 70:30, flow rate 1.0 mL/min, 40 °C, injection volume 20 μL.)

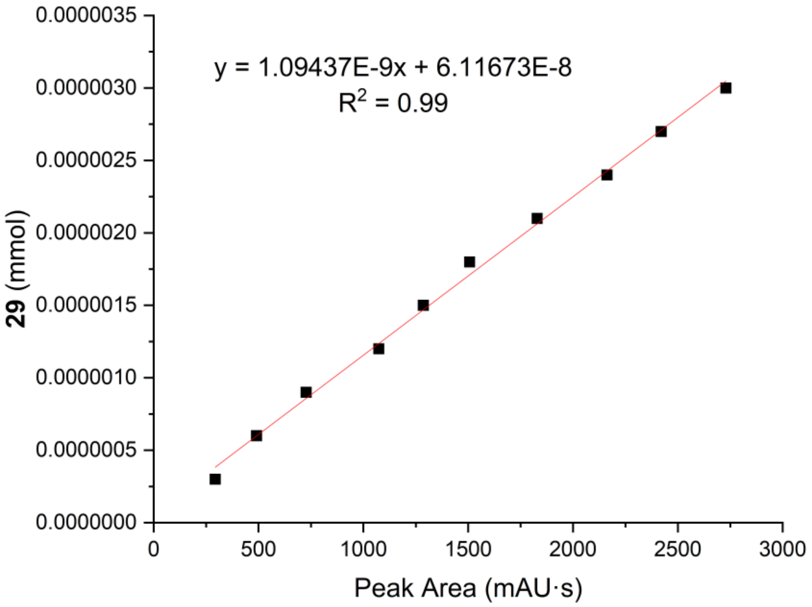

Figure S5. Calibration curve for **29**

*The zero-order dependence of the reaction rate with respect to the concentration of **28***

A dried 4 mL vial was charged with the **28** (0.003 mmol – 0.012 mmol, 0.3 – 1.2 equiv.), arylboronic acid **2a** (0.02 mmol, 2 equiv.), Pd2dba3 (5 mol%), L9 (12 mol%), CsF (0.02 mmol, 3.04 mg, 2 equiv.) and THF/H_2O (v/v, 9:1, 0.4 mL, 0.025 M) in glovebox. The mixture was stirred at 30°C for 12 h, and aliquots of the reaction mixture (20 μL) was added to isopropanol (980 μL), and the reaction conversion rate was monitored by HPLC peak area of product **29** (HPLC: IK-3, 254 nm, *n*-hexane/isopropanol = 70:30, flow rate 1.0 mL/min, 40 °C, injection volume 20 μL.)

The concentrations of **28** used: 0.0075 M, 0.015 M, 0.0225 M, 0.03 M.

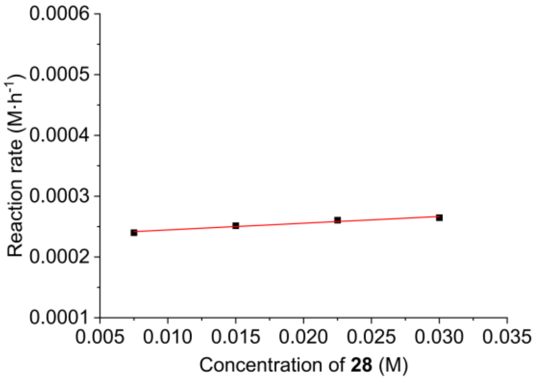

**28** (M)	Peak Area of **29** (mAU·s)	Reaction rate ($M \cdot h^{-1}$)
0.0075	997	2.4005E-4
0.0150	1047	2.5145E-4
0.0225	1087	2.6057E-4
0.0300	1105	2.6467E-4

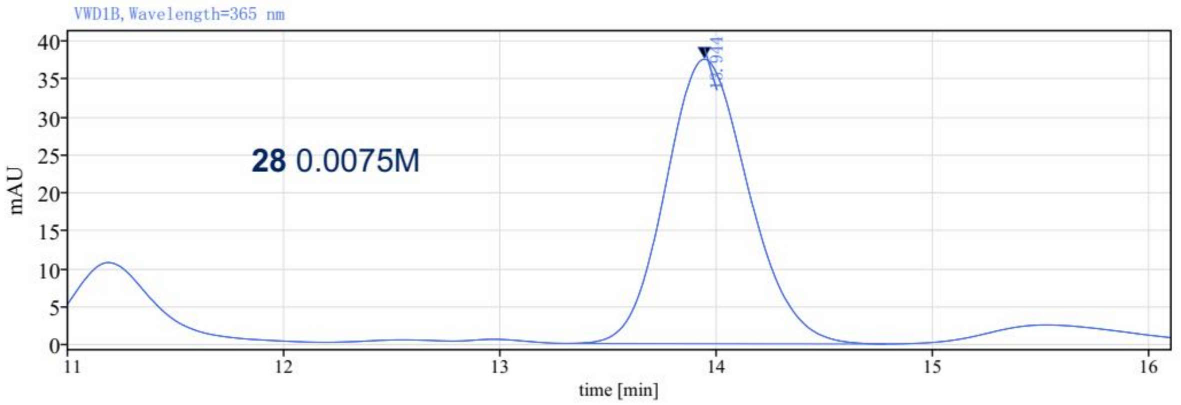

Rettime [min]	Type	Width [min]	Area	Height	Area%
13.944	BB	1.47	997.24	37.50	100.00

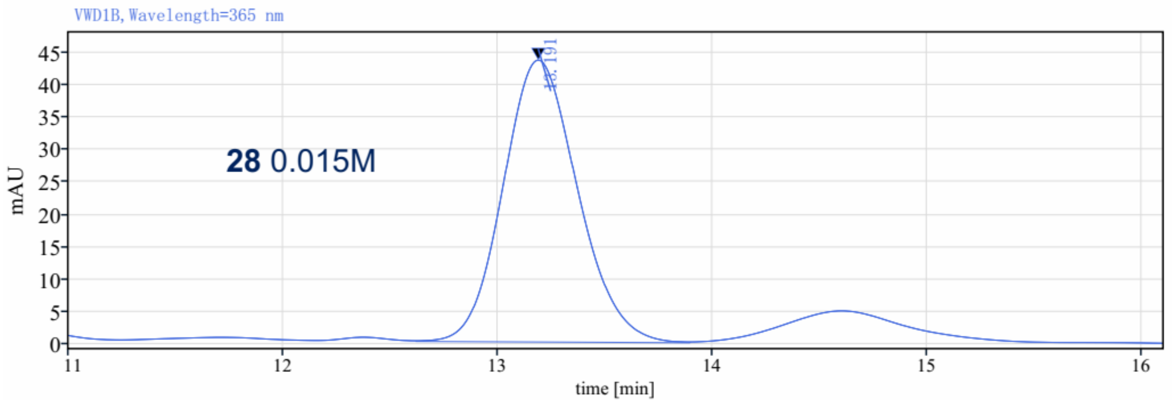

Rettime [min]	Type	Width [min]	Area	Height	Area%
13.191	VV	1.28	1047.01	43.41	100.00

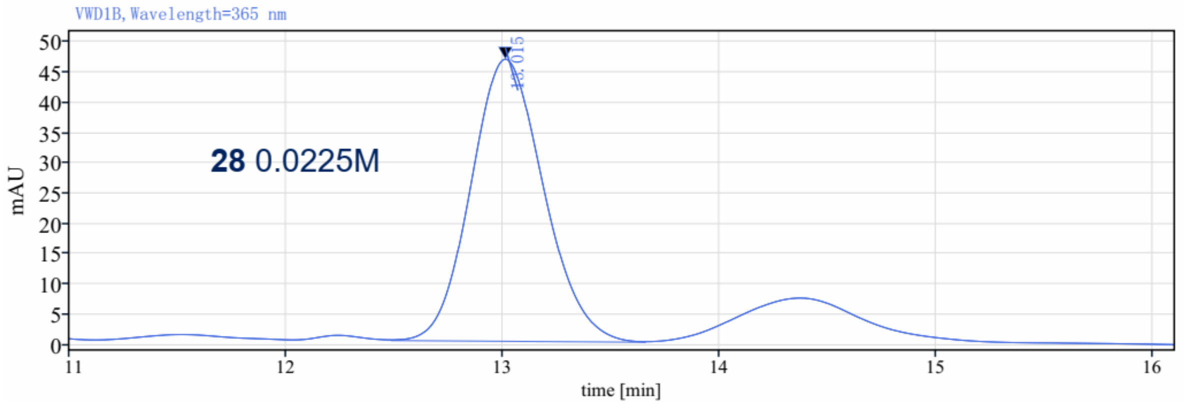

Rettime [min]	Type	Width [min]	Area	Height	Area%
13.015	VV	1.17	1087.04	46.63	100.00

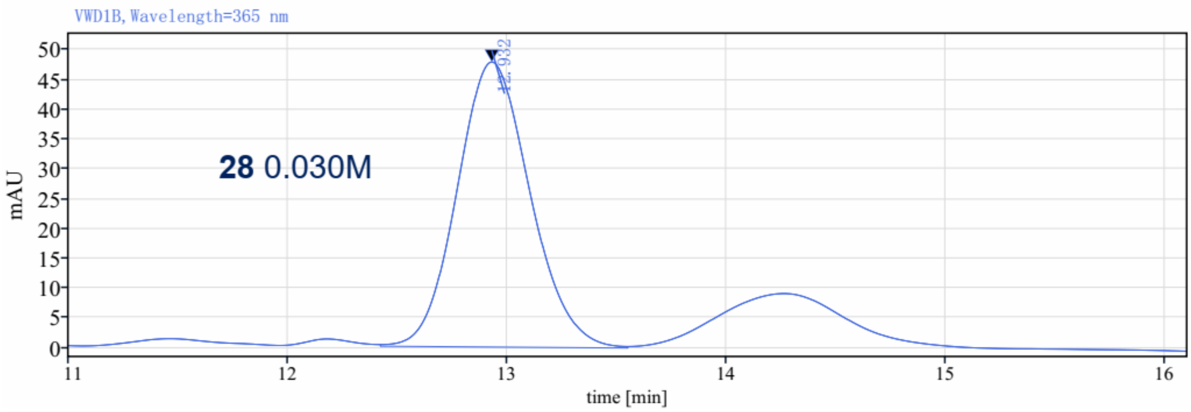

Rettime [min]	Type	Width [min]	Area	Height	Area%
12.932	VV	1.13	1105.59	47.89	100.00

The zero-order dependence of the reaction rate with respect to the concentration of ***2a***

A dried 4 mL vial was charged with the **28** (0.01 mmol, 1 equiv.), arylboronic acid **2a** (0.003 mmol – 0.012 mmol, 0.3 – 1.2 equiv.), Pd2dba3 (5 mol%), L9 (12 mol%), CsF (0.02 mmol, 3.04 mg, 2 equiv.) and THF/H_2O (v/v, 9:1, 0.4 mL, 0.025 M) in glovebox. The mixture was stirred at 30°C for 12 h, and aliquots of the reaction mixture (20 μL) was added to isopropanol (980 μL), and the reaction conversion rate was monitored by HPLC peak area of product **29** (HPLC: IK-3, 254 nm, *n*-hexane/isopropanol = 70:30, flow rate 1.0 mL/min, 40 ^{o}C, injection volume 20 μL.)

The concentrations of **2a** used: 0.0075 M, 0.015 M, 0.0225 M, 0.03 M.

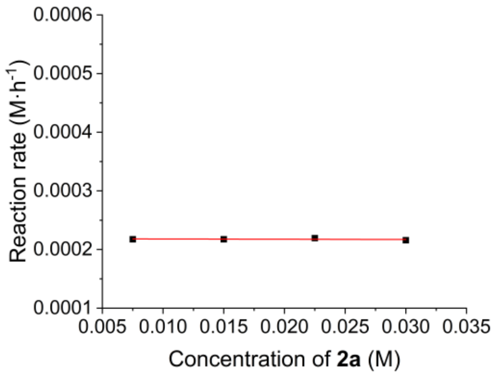

**2a** (M)	Peak Area of **29** (mAU·s)	Reaction rate ($M{\cdot}h^{-1}$)
0.0075	898	2.1748E-4
0.0150	898	2.1748E-4
0.0225	906	2.1937E-4
0.0300	891	2.1588E-4

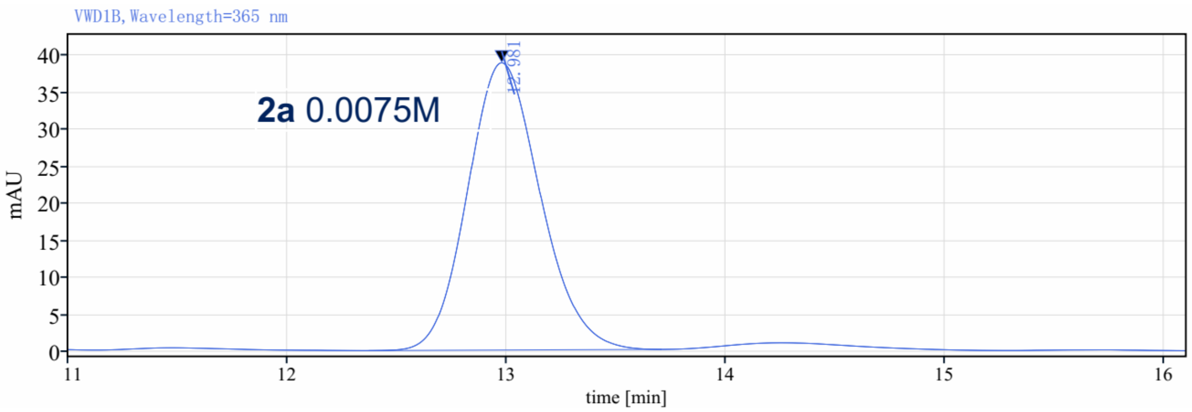

Rettime [min]	Type	Width [min]	Area	Height	Area%
12.981	BB	1.34	897.62	38.87	100.00

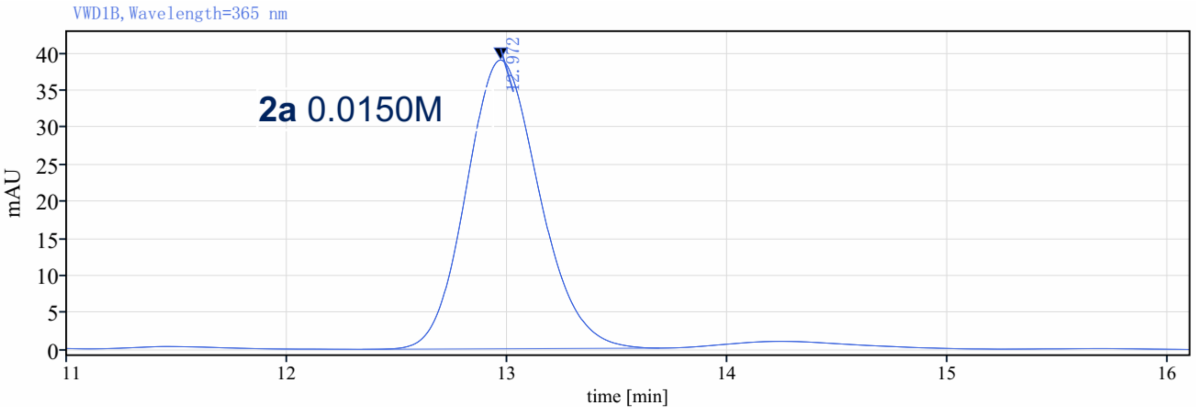

Rettime [min]	Type	Width [min]	Area	Height	Area%
12.972	BB	1.35	897.63	38.91	100.00

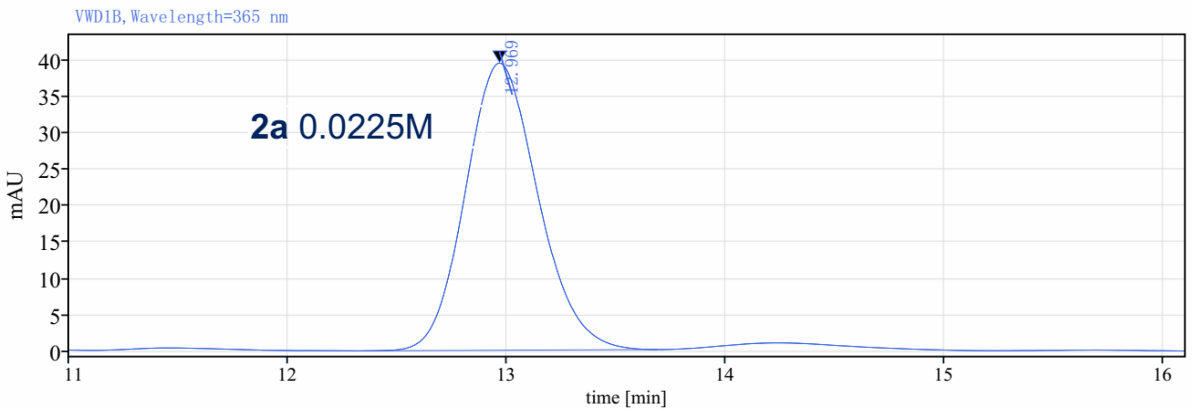

Rettime [min]	Type	Width [min]	Area	Height	Area%
12.969	BB	1.34	905.98	39.32	100.00

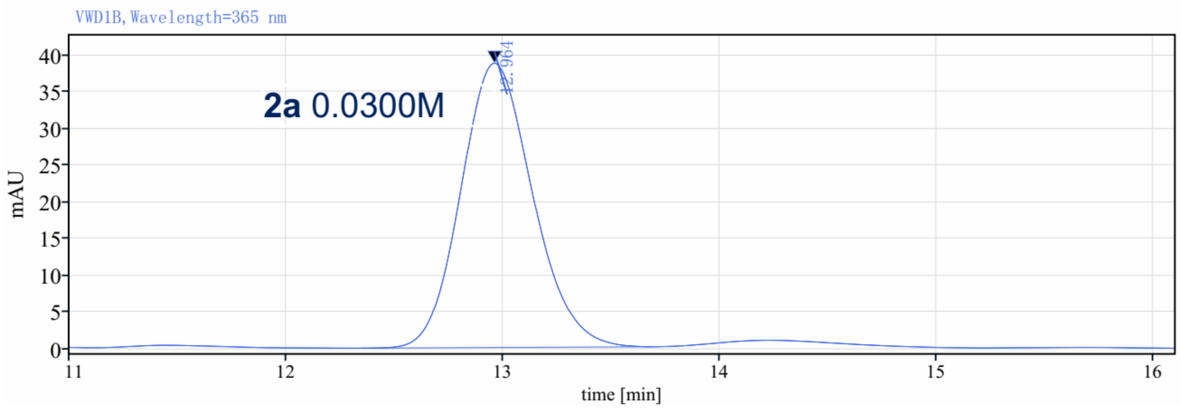

Rettime [min]	Type	Width [min]	Area	Height	Area%
12.964	BB	1.34	891.47	38.73	100.00

The first-order dependence of the reaction rate with respect to the concentration of ***cat.***

A dried 4 mL vial was charged with the **28** (0.01 mmol, 1 equiv.), arylboronic acid **2a** (0.01 mmol, 1 equiv.), **cat.** (3 – 12 mol%,), CsF (0.02 mmol, 3.04 mg, 2 equiv.) and THF/H_2O (v/v, 9:1, 0.4 mL, 0.025 M) in glovebox. The mixture was stirred at 30°C for 12 h, and aliquots of the reaction mixture (20 μL) was added to isopropanol (980 μL), and the reaction conversion rate was monitored by HPLC peak area of product **29** (HPLC: IK-3, 254 nm, *n*-hexane/isopropanol = 70:30, flow rate 1.0 mL/min, 40 °C, injection volume 20 μL.)

The concentrations of **cat.** used: 0.00075 M, 0.0015 M, 0.00225 M, 0.003 M.

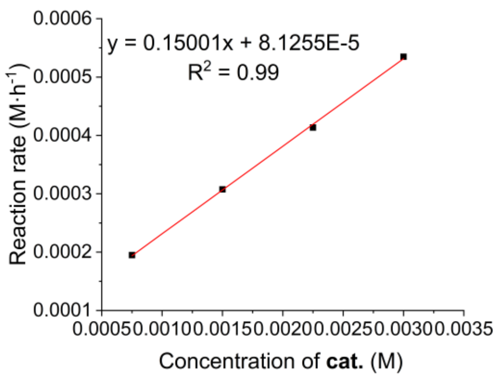

**cat.** (M)	Peak Area of **29** (mAU·s)	Reaction rate ($M \cdot h^{-1}$)
0.00075	799	1.9491E-4
0.00150	1293	3.0754E-4
0.00225	1758	4.1356E-4
0.00300	2290	5.3485E-4

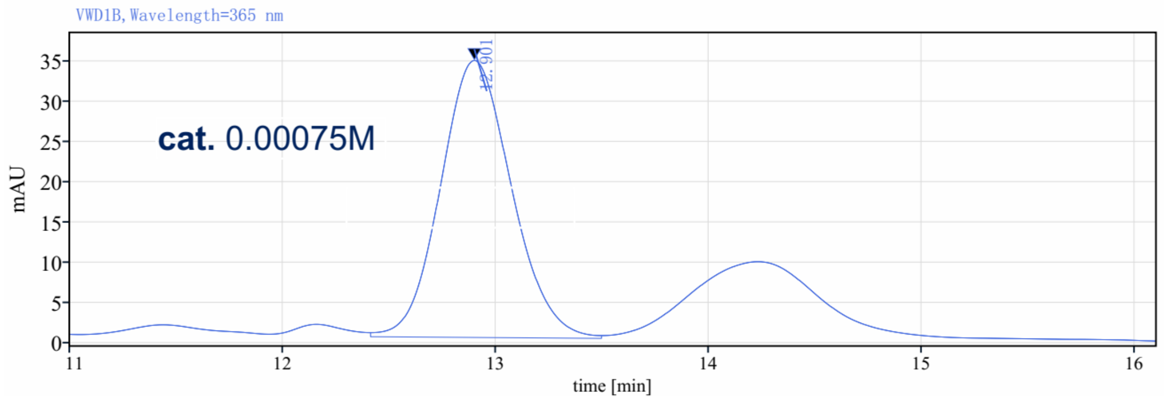

Rettime [min]	Type	Width [min]	Area	Height	Area%
12.901	VV	1.08	798.96	34.51	100.00

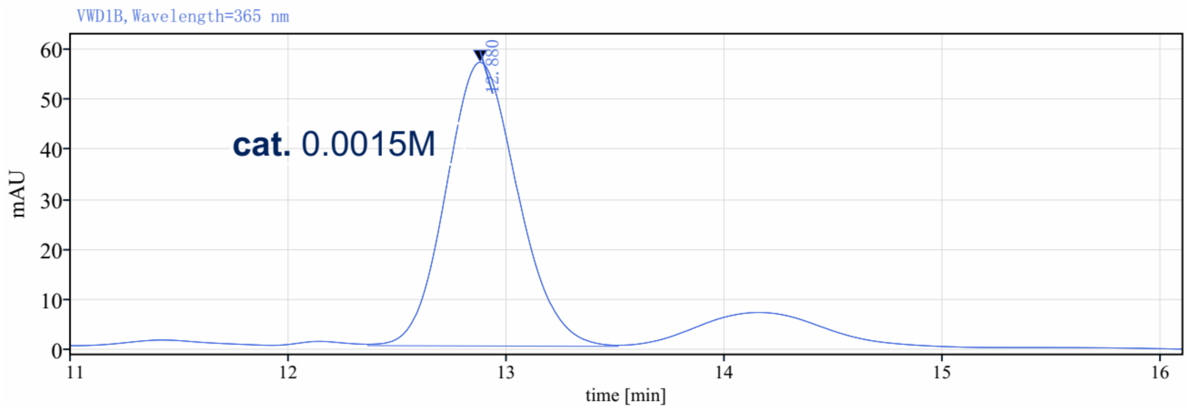

Rettime [min]	Type	Width [min]	Area	Height	Area%
12.880	VV	1.15	1293.23	56.64	100.00

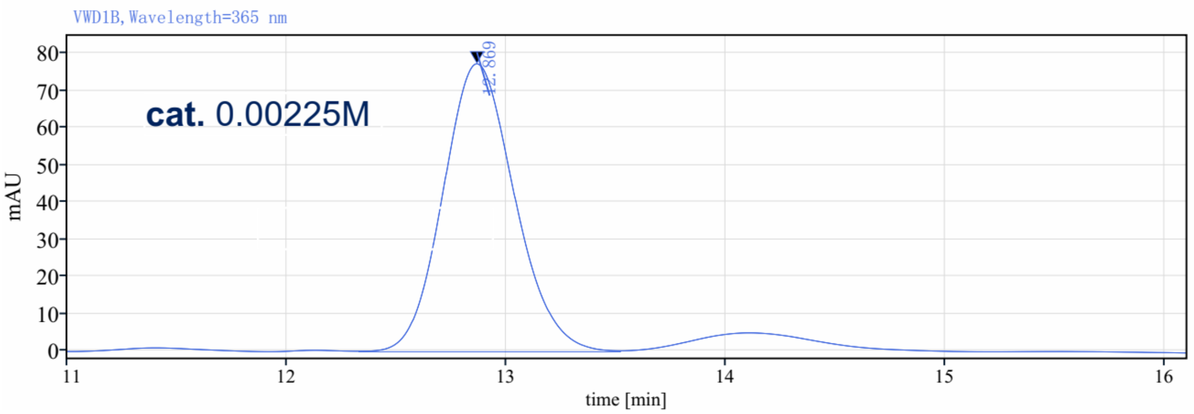

Rettime [min]	Type	Width [min]	Area	Height	Area%
12.869	VV	1.19	1757.51	77.56	100.00

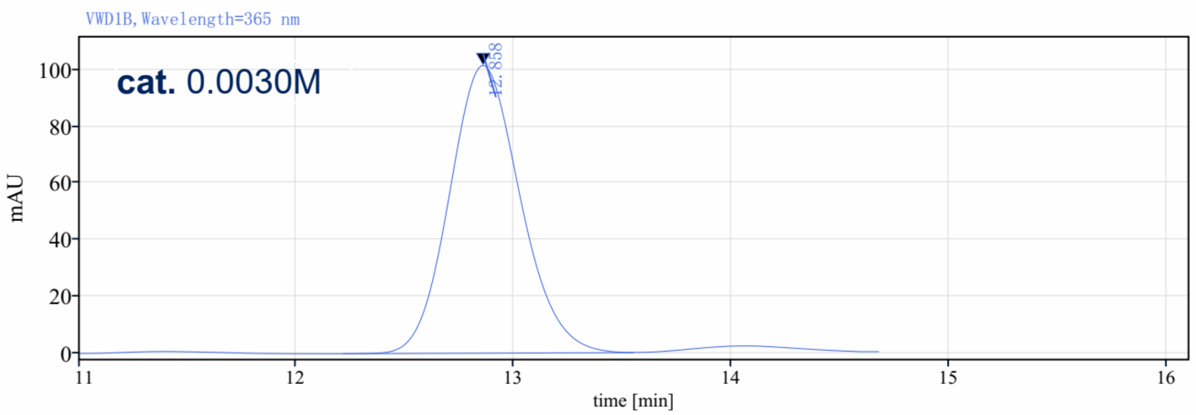

Rettime [min]	Type	Width [min]	Area	Height	Area%
12.858	BV	1.34	2293.67	101.61	100.00

9. Circular Dichroism

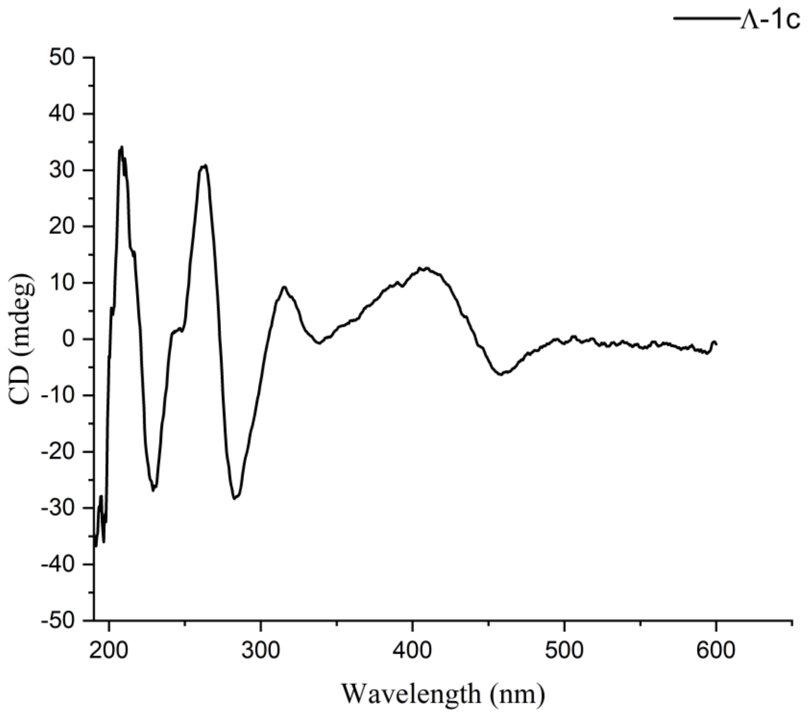

Figure S6. CD spectrum of **Λ-1c** recorded in MeCN (0.000025 mol/L).

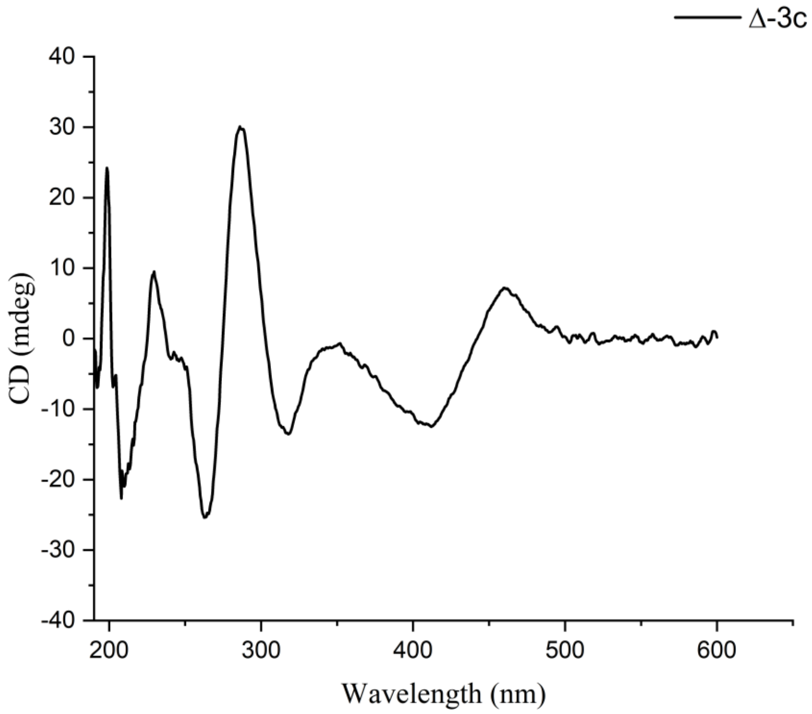

Figure S7. CD spectrum of **Δ-3c** recorded in MeCN (0.000025 mol/L)

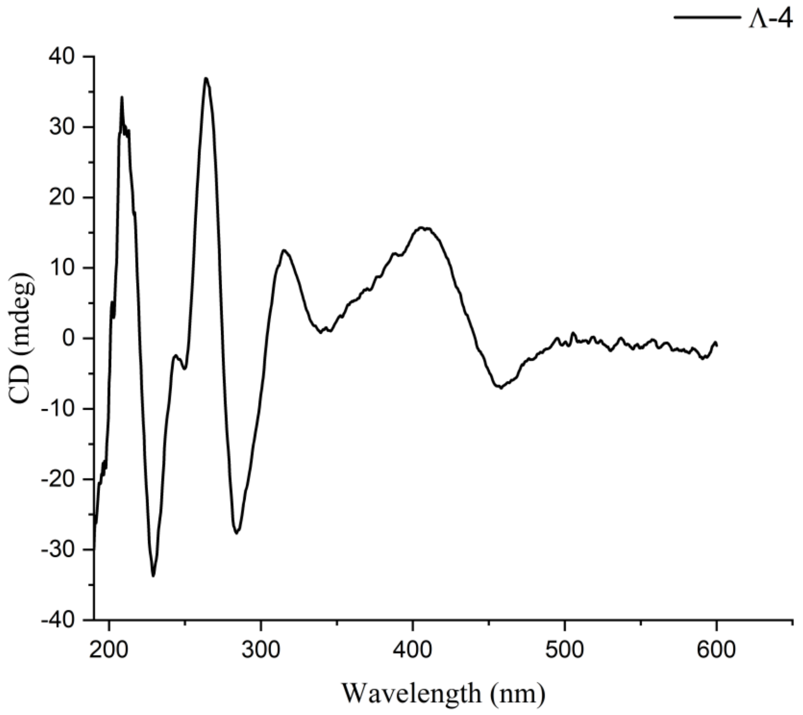

Figure S8. CD spectrum of **Λ-4** recorded in MeCN (0.000025 mol/L)

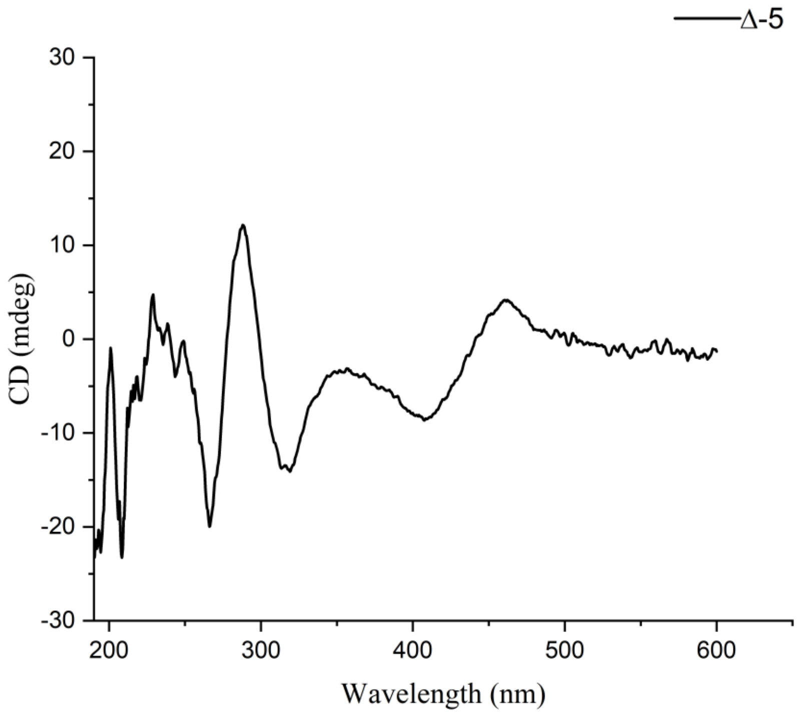

Figure S9. CD spectrum of **Δ-5** recorded in MeCN (0.000025 mol/L)

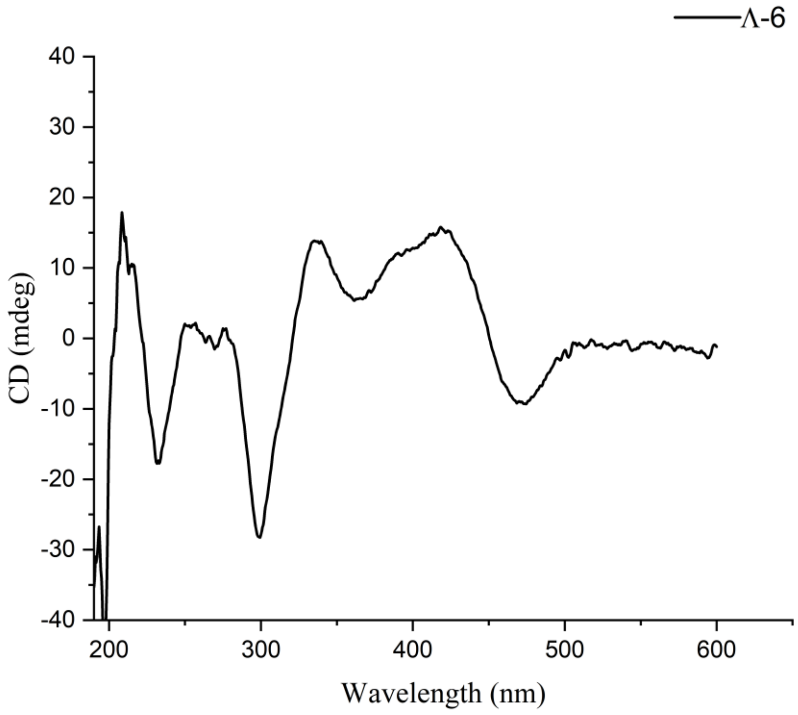

Figure S10. CD spectrum of **Λ-6** recorded in MeCN (0.000025 mol/L)

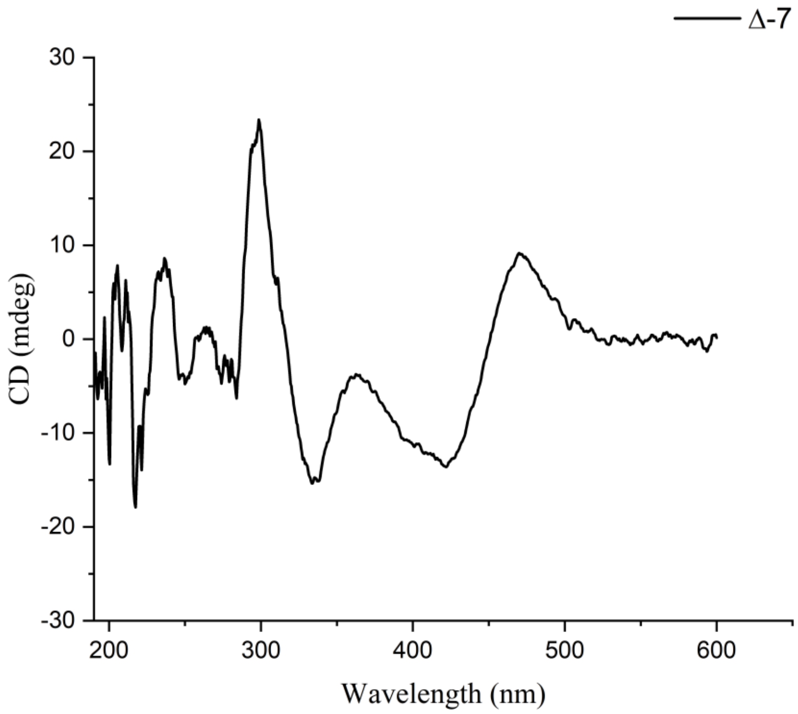

Figure S11. CD spectrum of **Δ-7** recorded in MeCN (0.000025 mol/L)

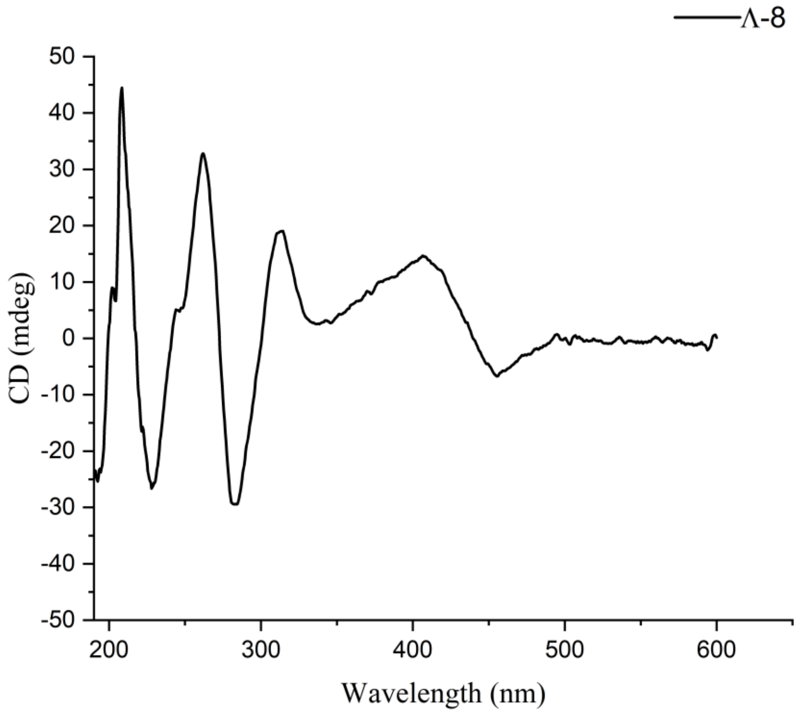

Figure S12. CD spectrum of **Λ-8** recorded in MeCN (0.000025 mol/L)

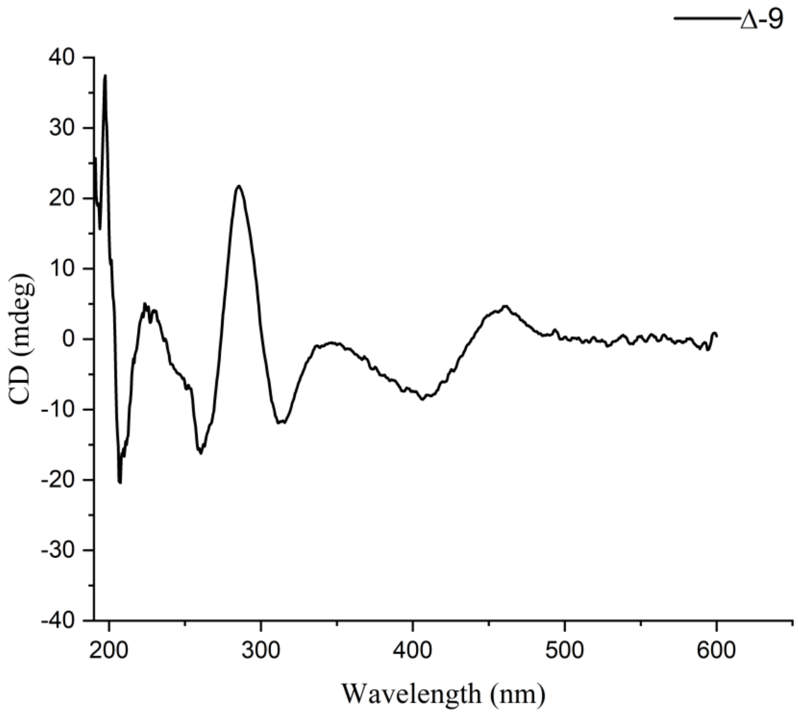

Figure S13. CD spectrum of **Δ-9** recorded in MeCN (0.000025 mol/L)

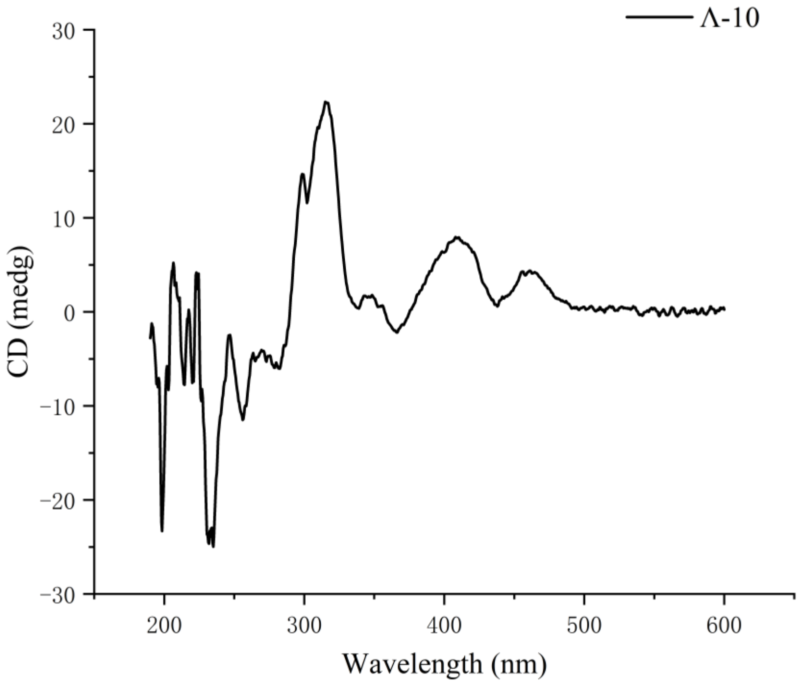

Figure S14. CD spectrum of **Λ-10** recorded in MeCN (0.000025 mol/L).

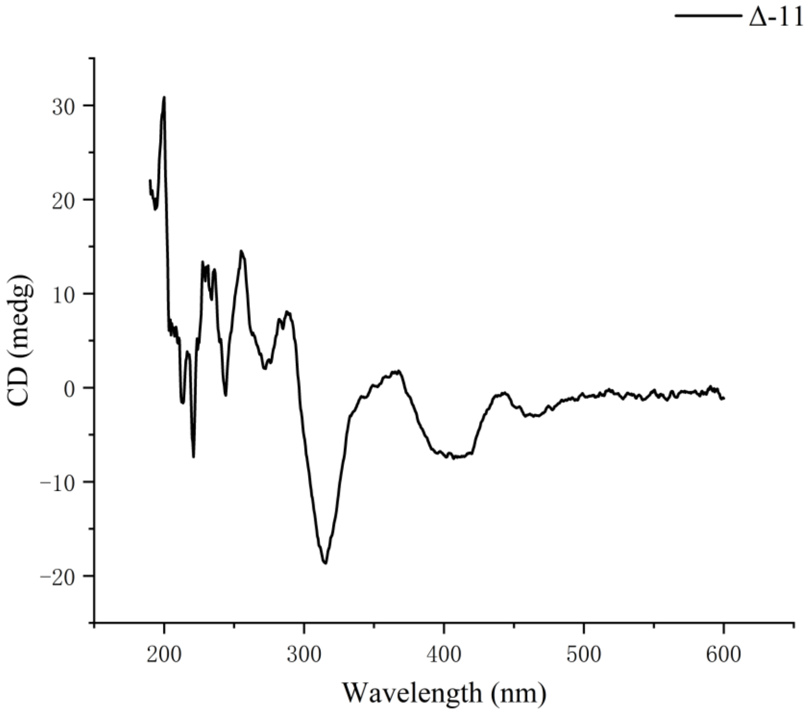

Figure S15. CD spectrum of **Λ-11** recorded in MeCN (0.000025 mol/L)

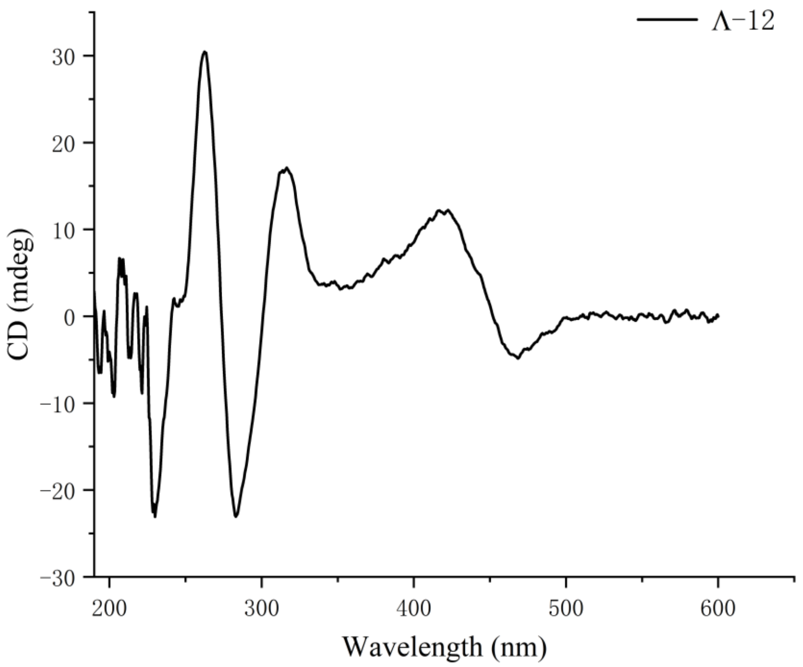

Figure S16. CD spectrum of **Λ-12** recorded in MeCN (0.000025 mol/L)

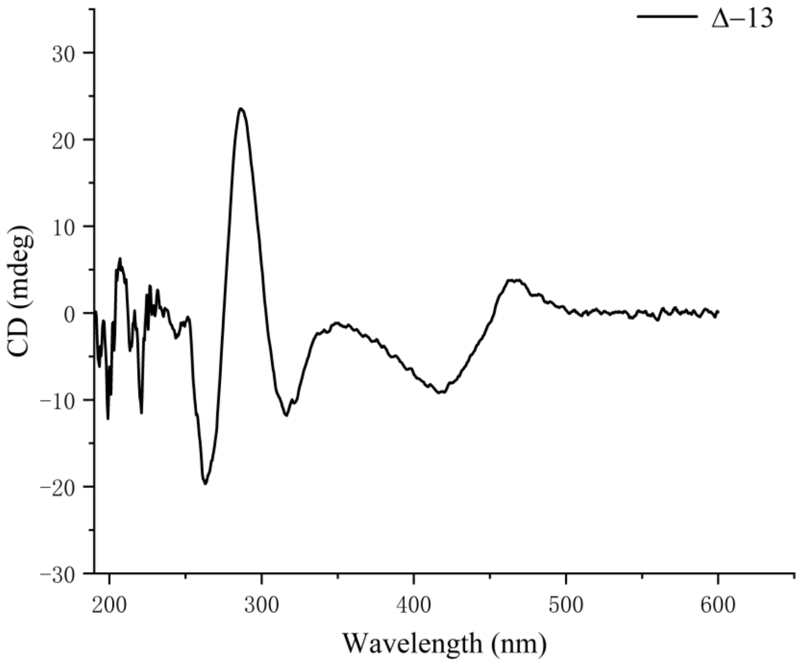

Figure S17. CD spectrum of **Δ-13** recorded in MeCN (0.000025 mol/L)

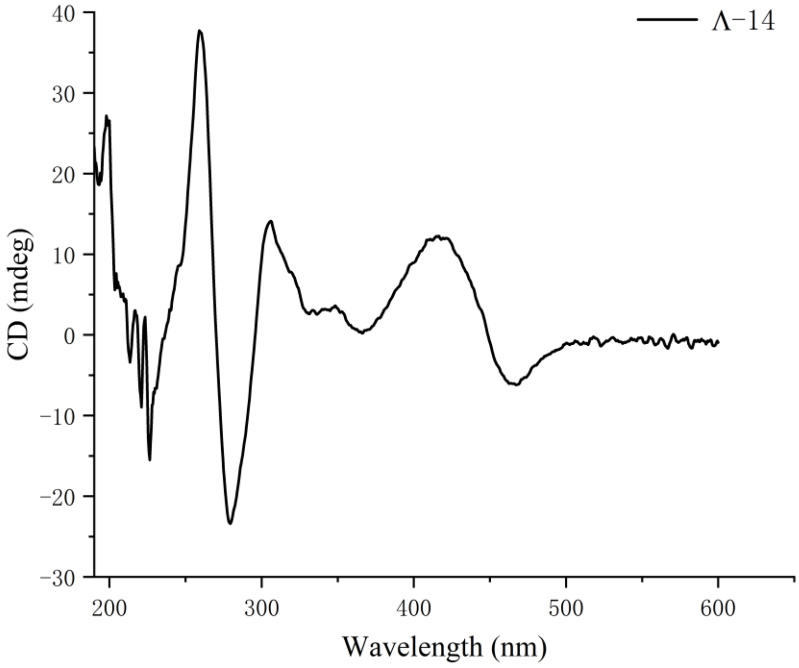

Figure S18. CD spectrum of **Λ-14** recorded in MeCN (0.000025 mol/L)

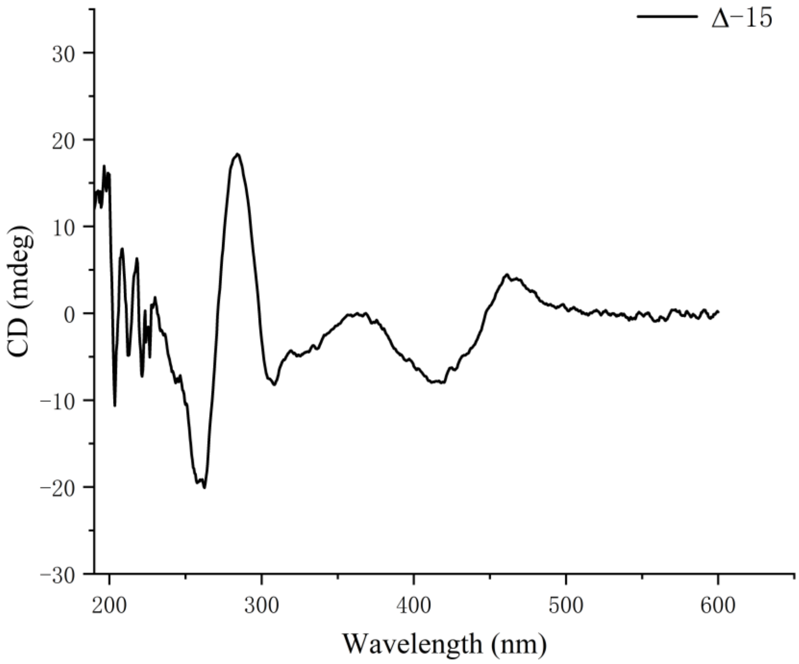

Figure S19. CD spectrum of **Δ-15** recorded in MeCN (0.000025 mol/L)

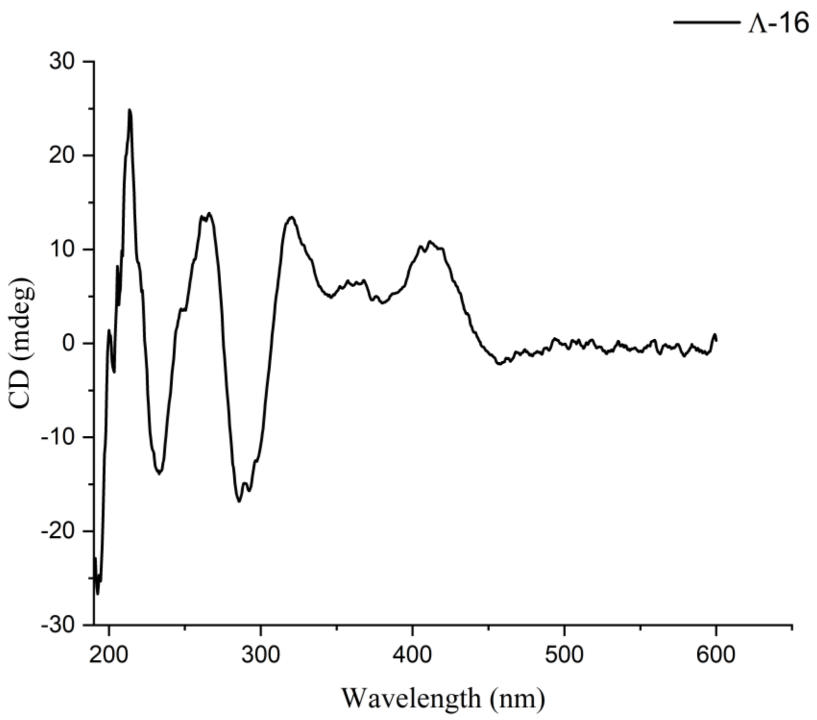

Figure S20. CD spectrum of **Λ-16** recorded in MeCN (0.000025 mol/L)

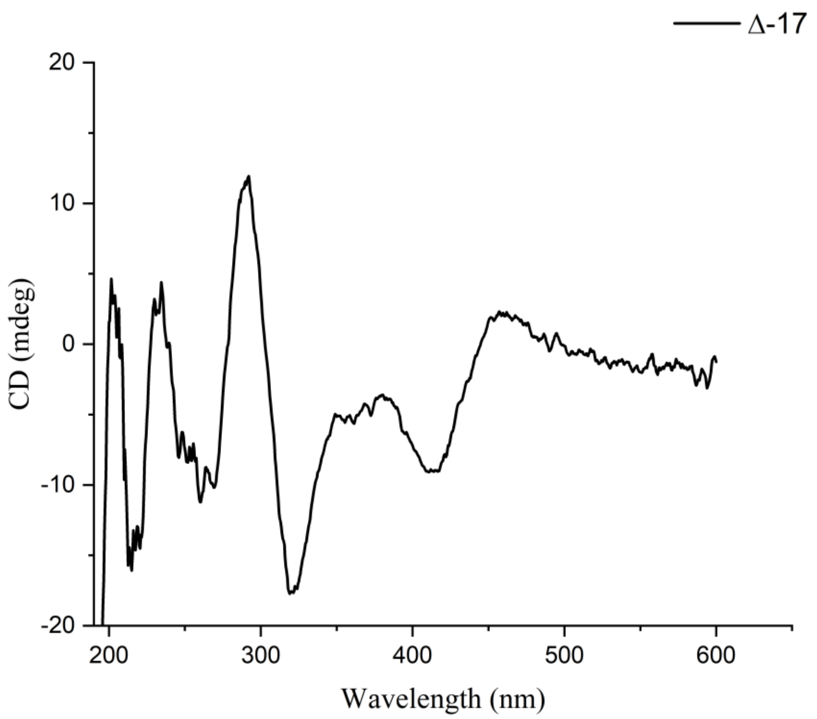

Figure S21. CD spectrum of **Δ-17** recorded in MeCN (0.000025 mol/L)

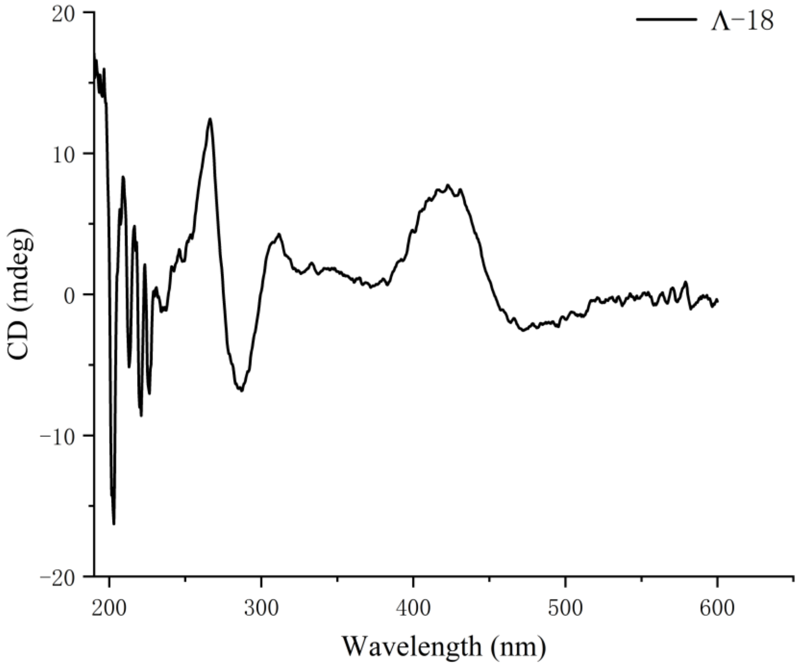

Figure S22. CD spectrum of **Λ-18** recorded in MeCN (0.000025 mol/L)

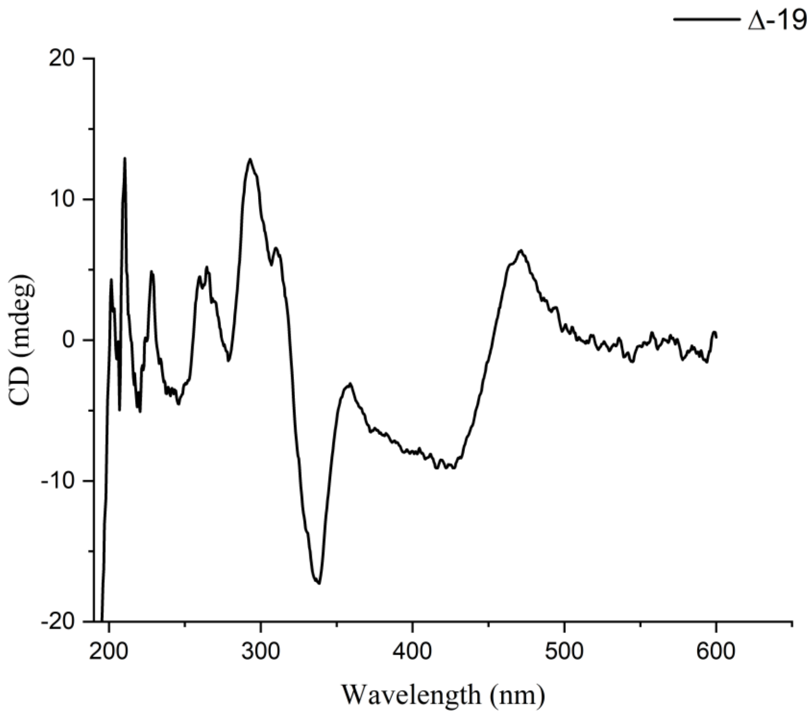

Figure S23. CD spectrum of **Δ-19** recorded in MeCN (0.000025 mol/L)

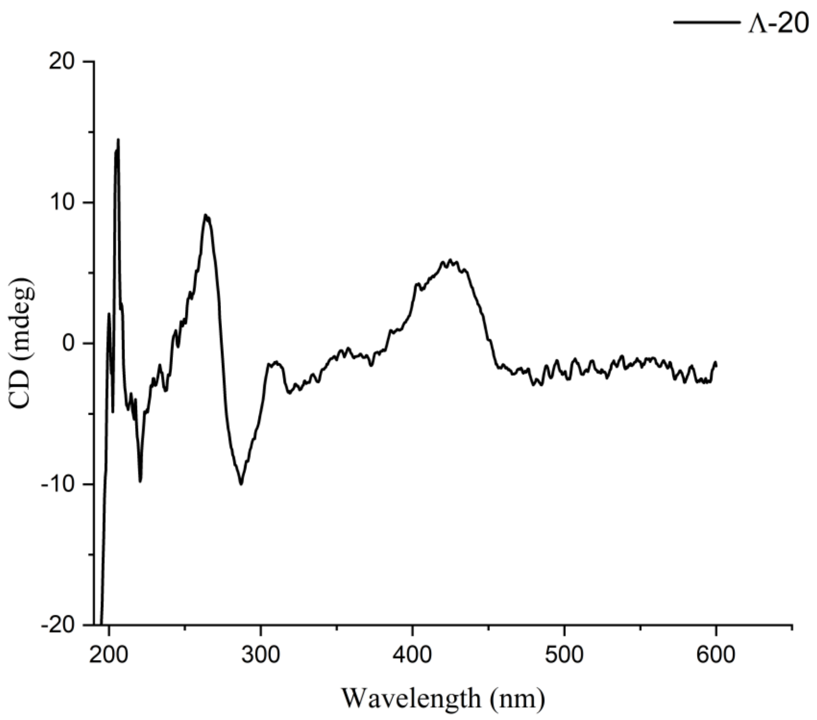

Figure S24. CD spectrum of **Λ-20** recorded in MeCN (0.000025 mol/L)

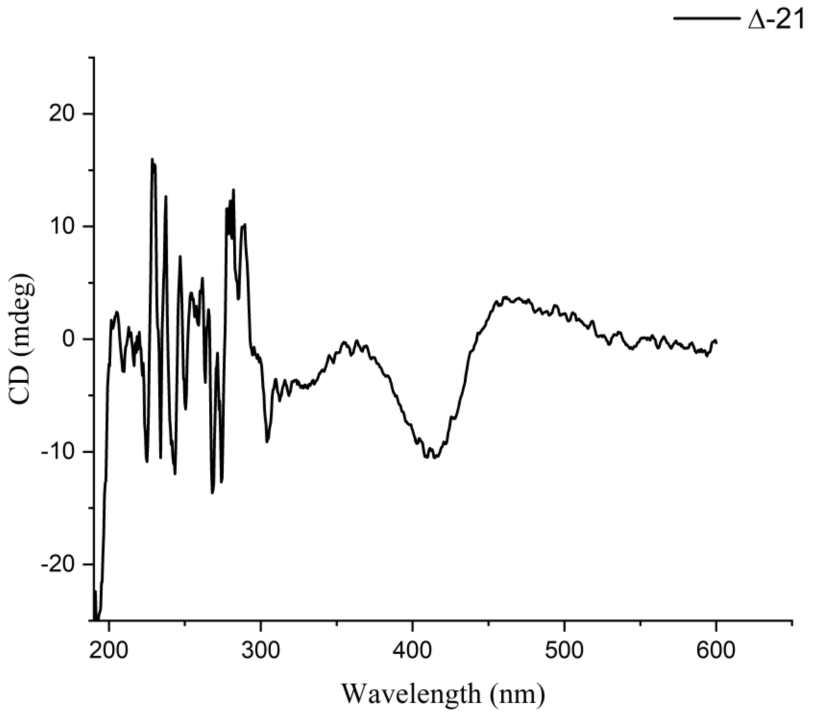

Figure S25. CD spectrum of **Δ-21** recorded in MeCN (0.000025 mol/L)

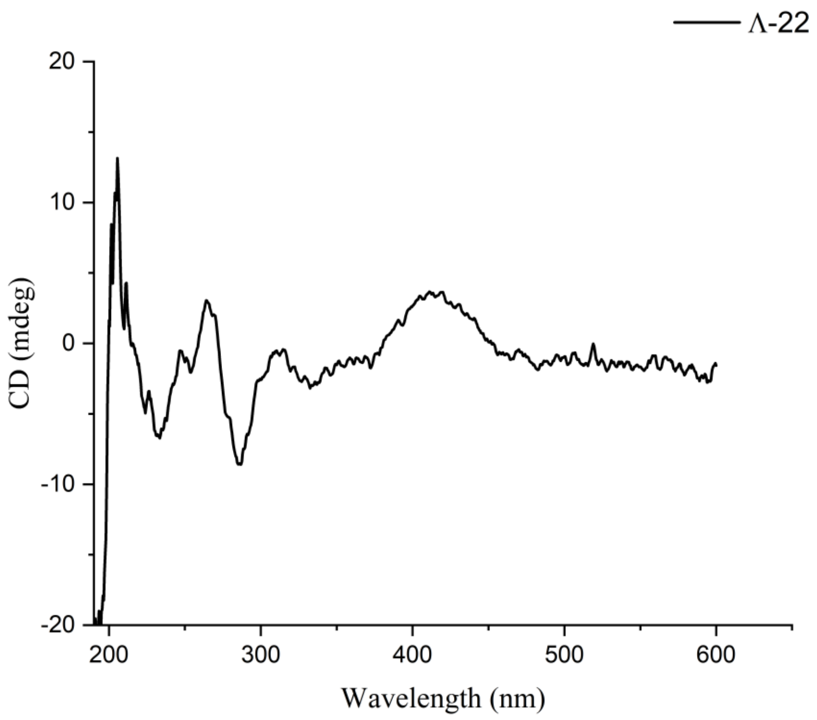

Figure S26. CD spectrum of **Λ-22** recorded in MeCN (0.000025 mol/L)

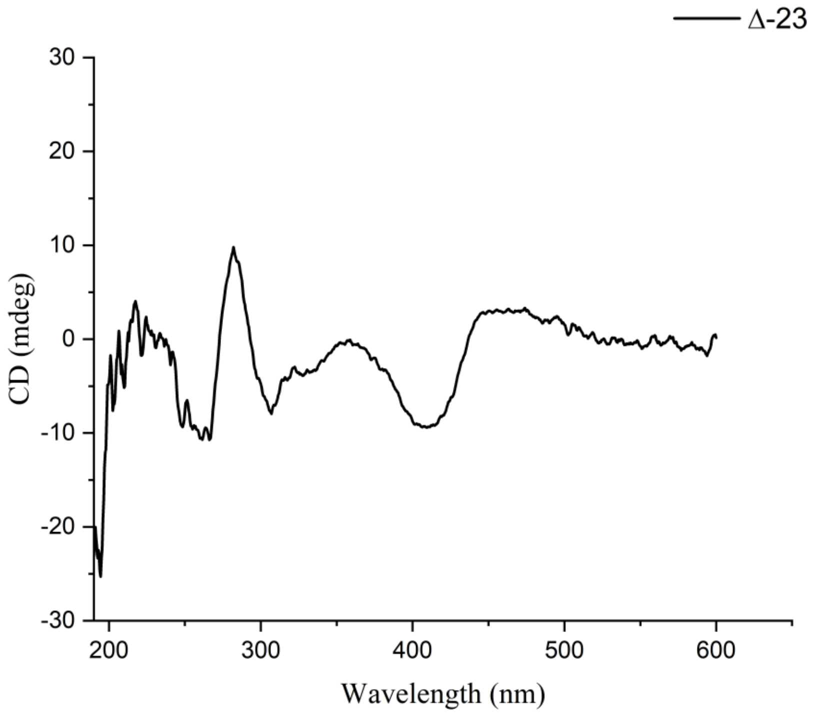

Figure S27. CD spectrum of **Δ-23** recorded in MeCN (0.000025 mol/L)

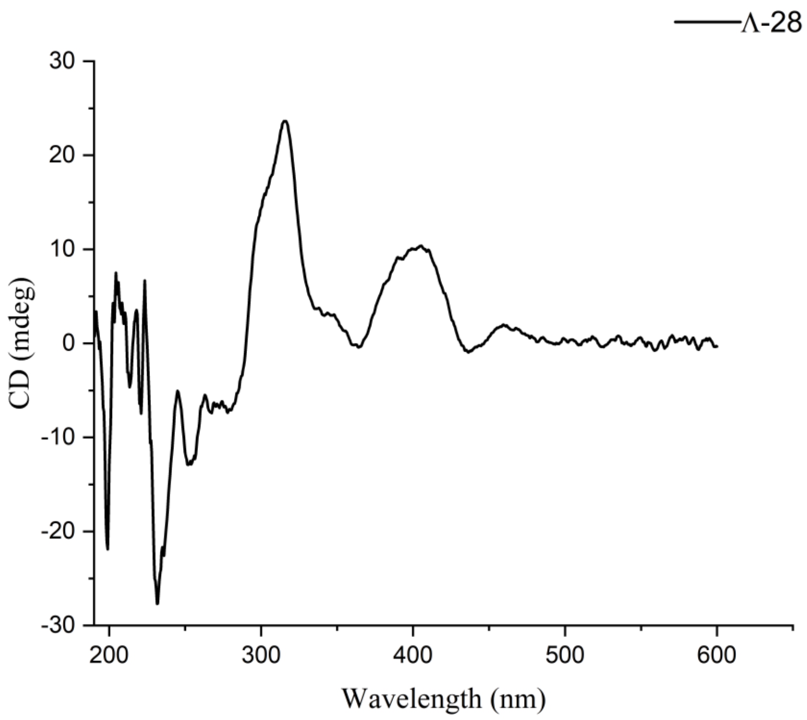

Figure S28. CD spectrum of **Λ-28** recorded in MeCN (0.000025 mol/L)

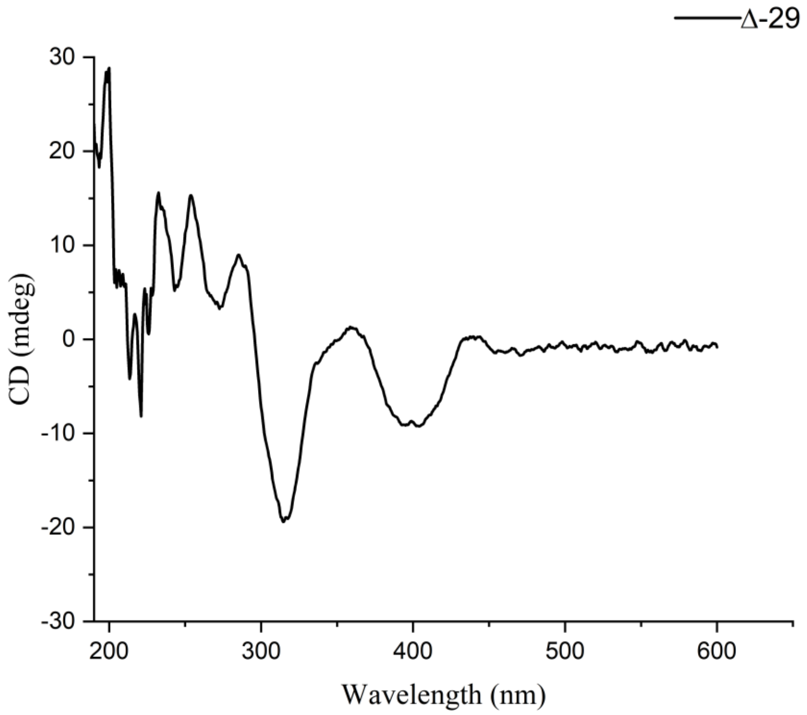

Figure S29. CD spectrum of **Δ-29** recorded in MeCN (0.000025 mol/L)

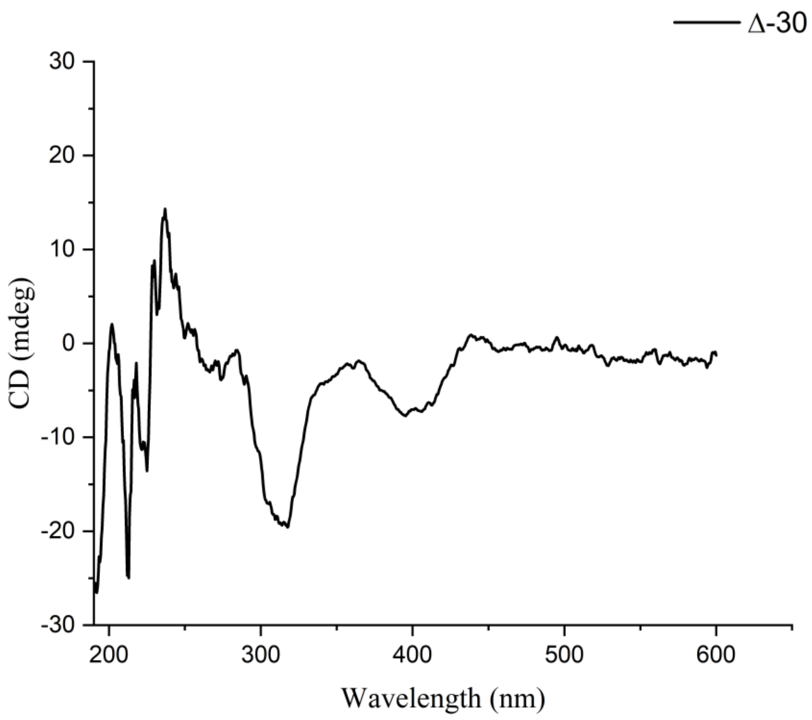

Figure S30. CD spectrum of **Δ-30** recorded in MeCN (0.000025 mol/L)

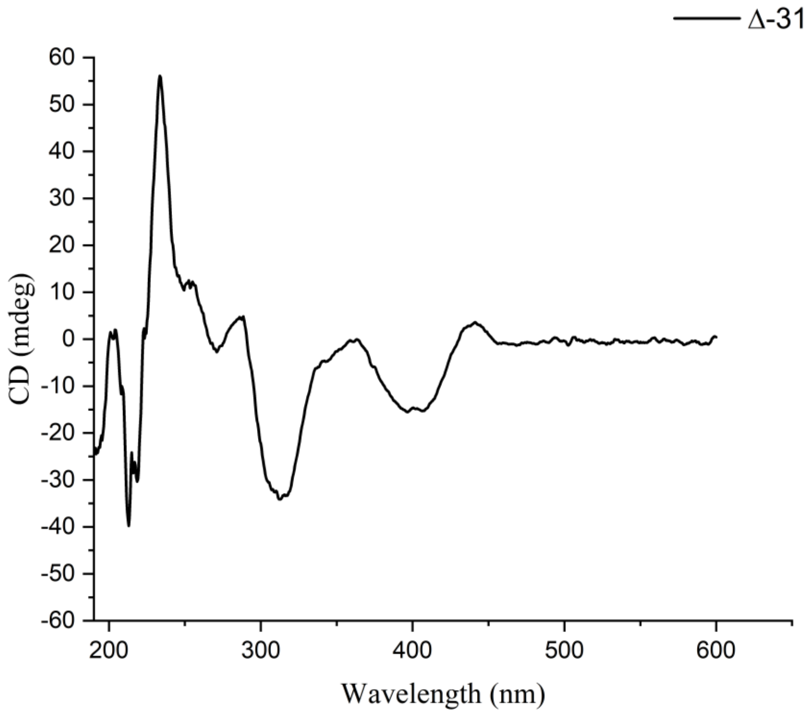

Figure S31. CD spectrum of **Δ-31** recorded in MeCN (0.000025 mol/L)

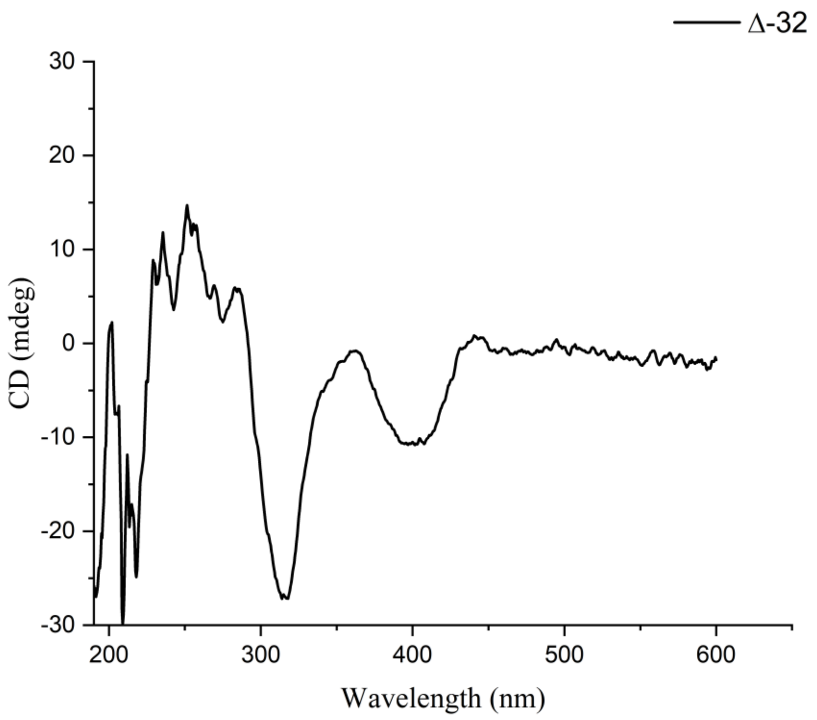

Figure S32. CD spectrum of **Δ-32** recorded in MeCN (0.000025 mol/L)

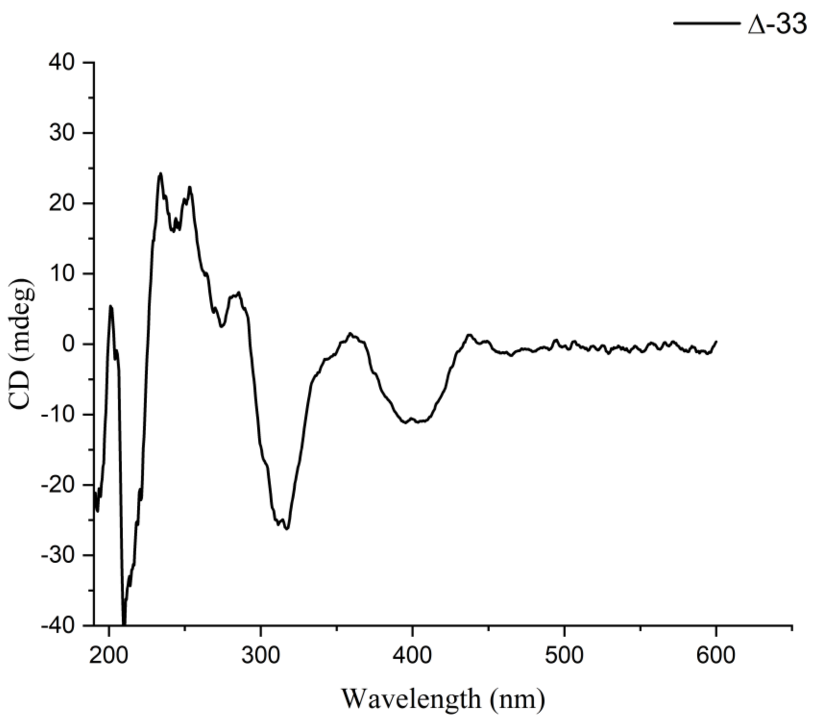

Figure S33. CD spectrum of **Δ-33** recorded in MeCN (0.000025 mol/L)

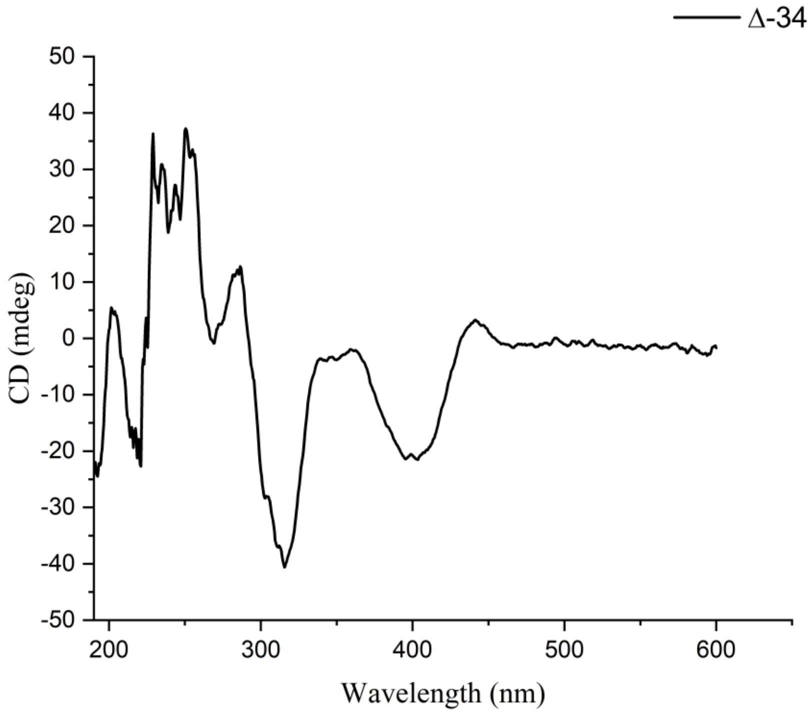

Figure S34. CD spectrum of **Δ-34** recorded in MeCN (0.000025 mol/L)

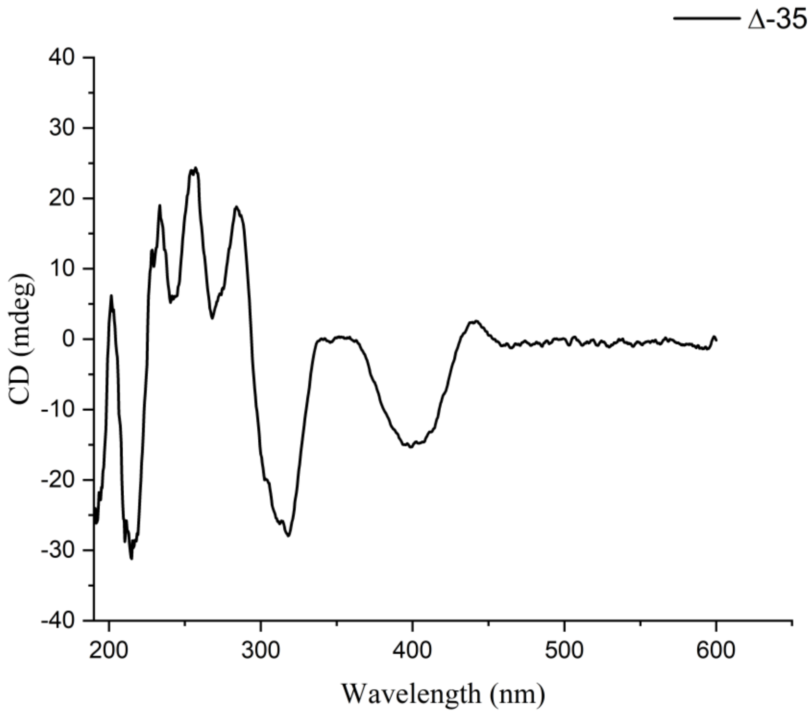

Figure S35. CD spectrum of **Δ-35** recorded in MeCN (0.000025 mol/L)

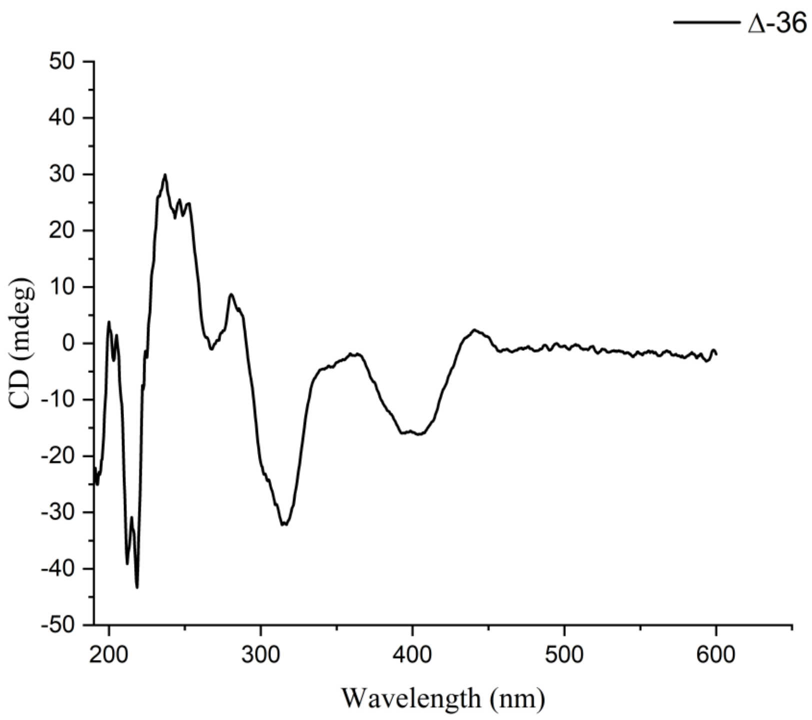

Figure S36. CD spectrum of **Δ-36** recorded in MeCN (0.000025 mol/L)

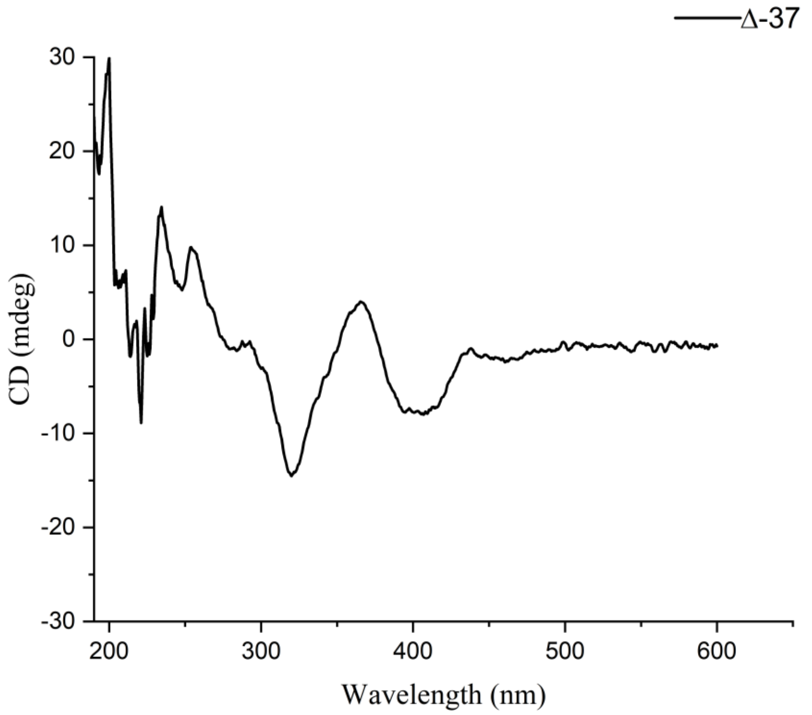

Figure S37. CD spectrum of **Δ-37** recorded in MeCN (0.000025 mol/L)

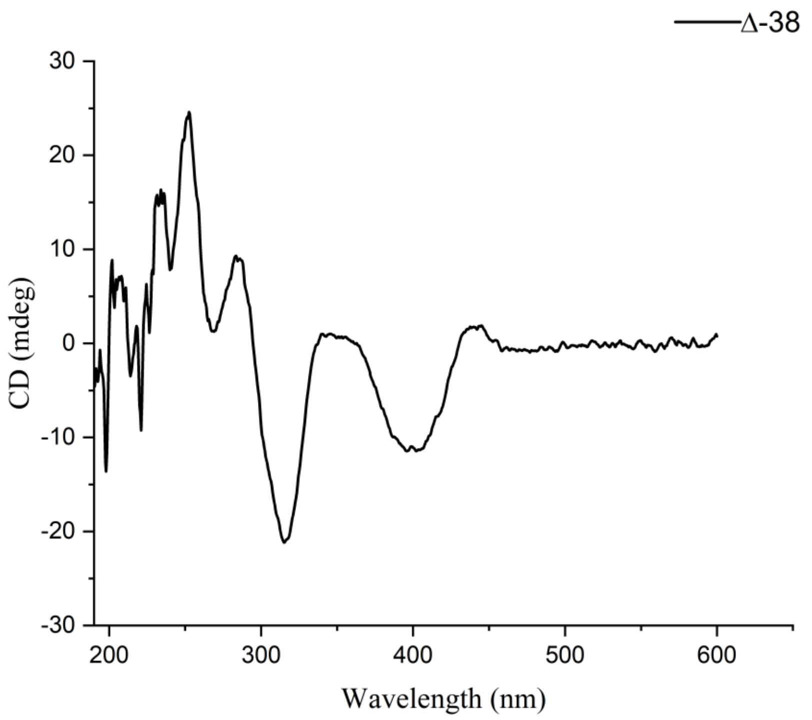

Figure S38. CD spectrum of **Δ-38** recorded in MeCN (0.000025 mol/L)

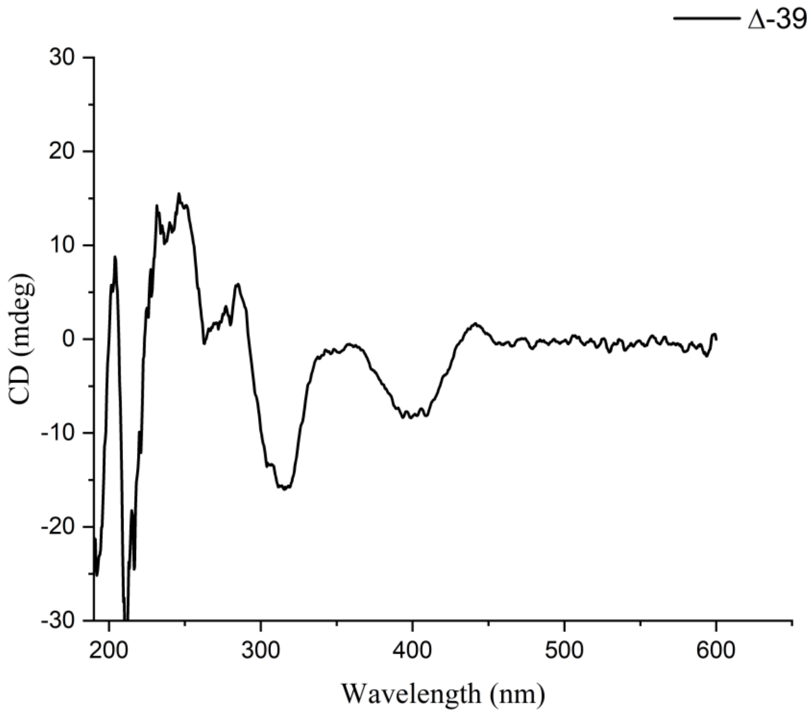

Figure S39. CD spectrum of **Δ-39** recorded in MeCN (0.000025 mol/L)

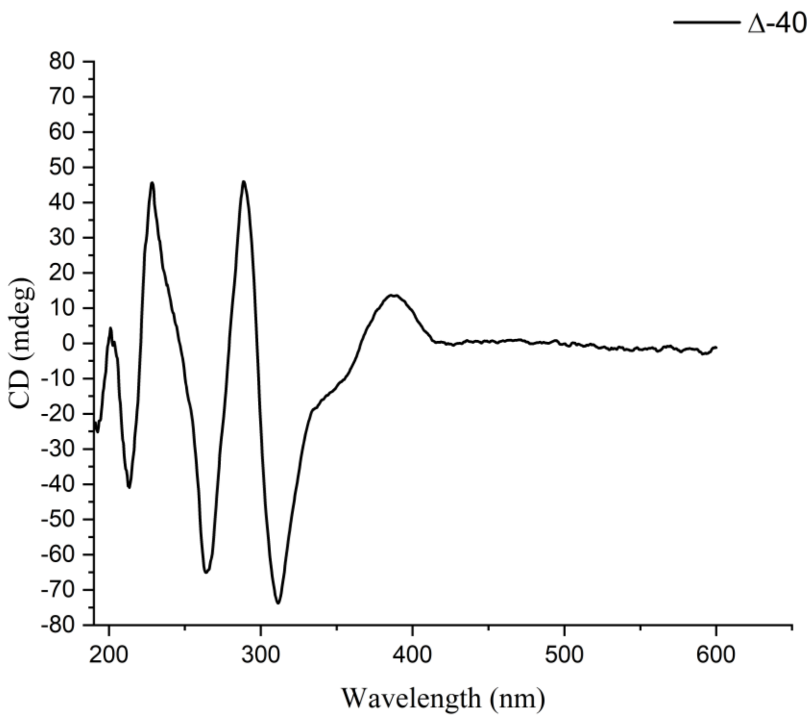

Figure S40. CD spectrum of **Δ-40** recorded in MeCN (0.000015 mol/L)

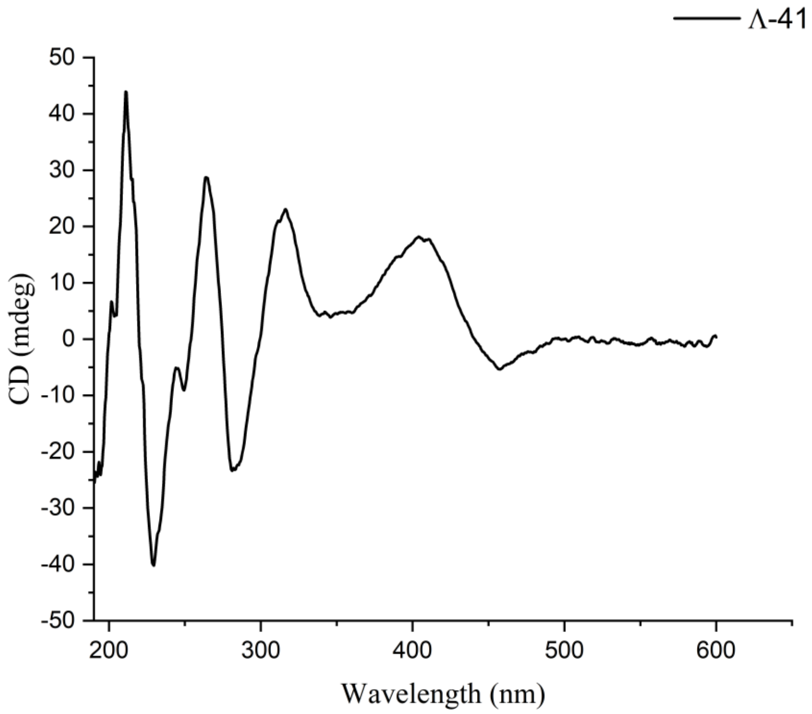

Figure S41. CD spectrum of **Λ-41** recorded in MeCN (0.000025 mol/L)

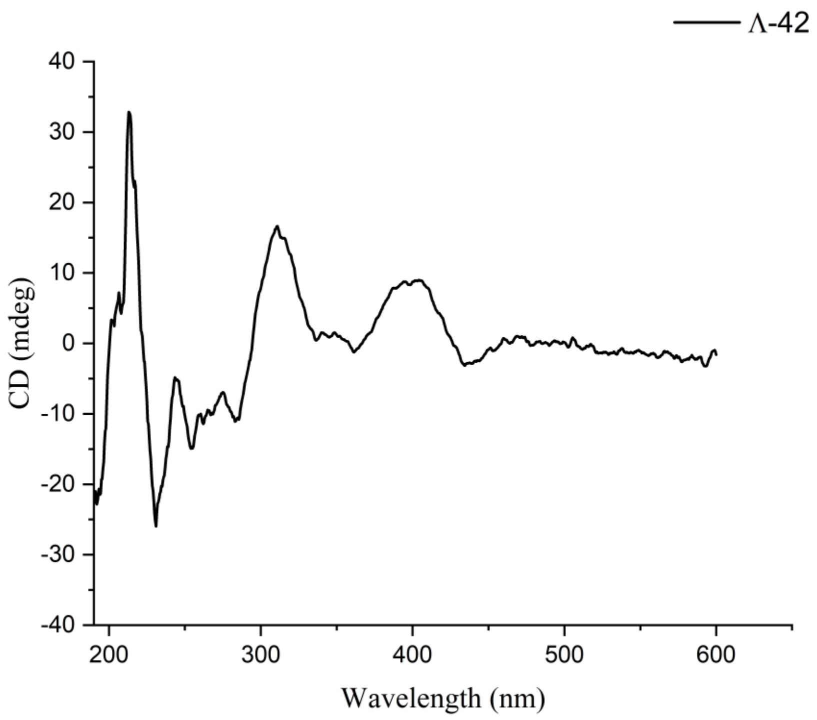

Figure S42. CD spectrum of **Λ-42** recorded in MeCN (0.000025 mol/L)

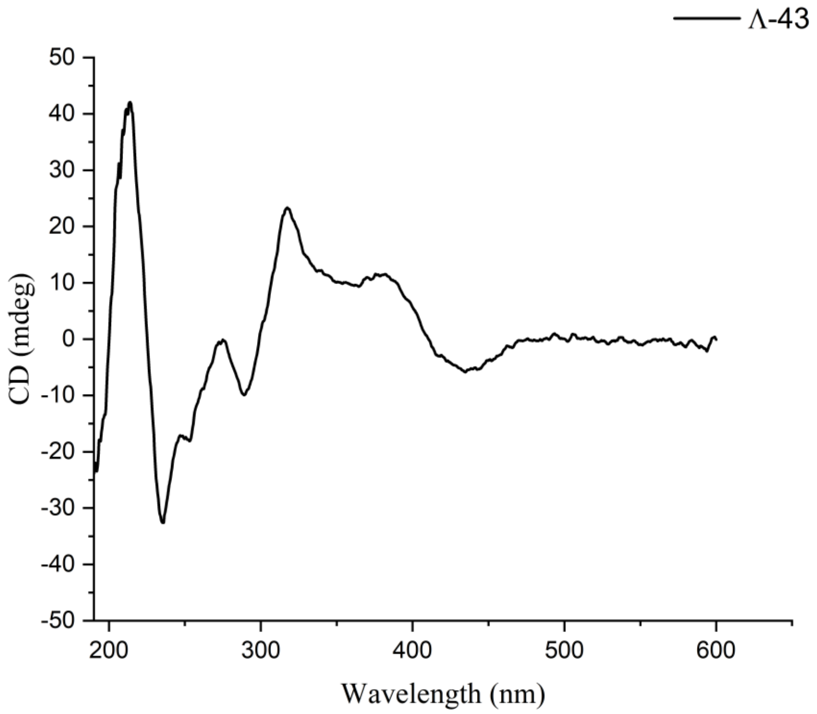

Figure S43. CD spectrum of **Λ-43** recorded in MeCN (0.000010 mol/L)

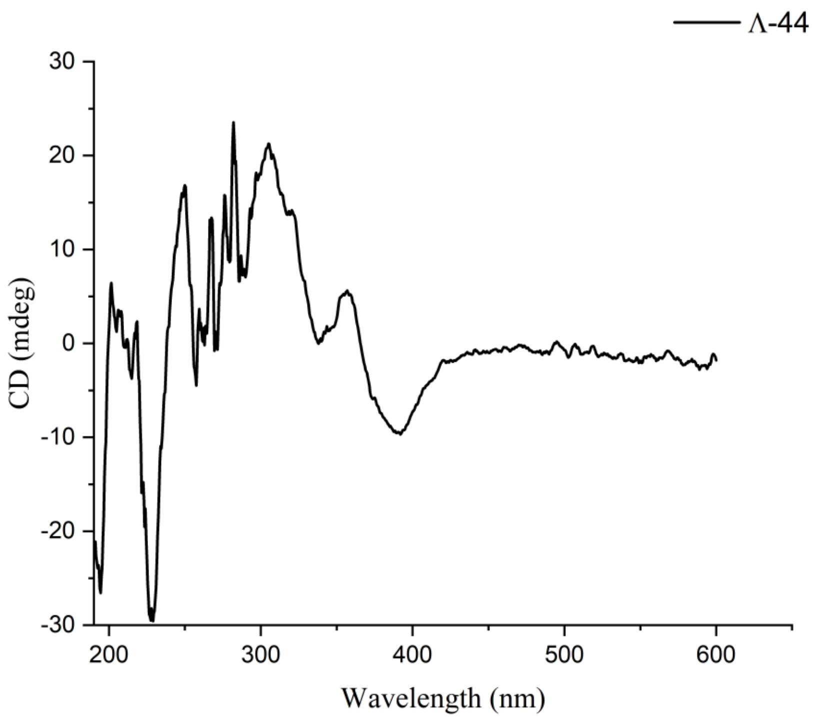

Figure S44. CD spectrum of **Λ-44** recorded in MeCN (0.000010 mol/L)

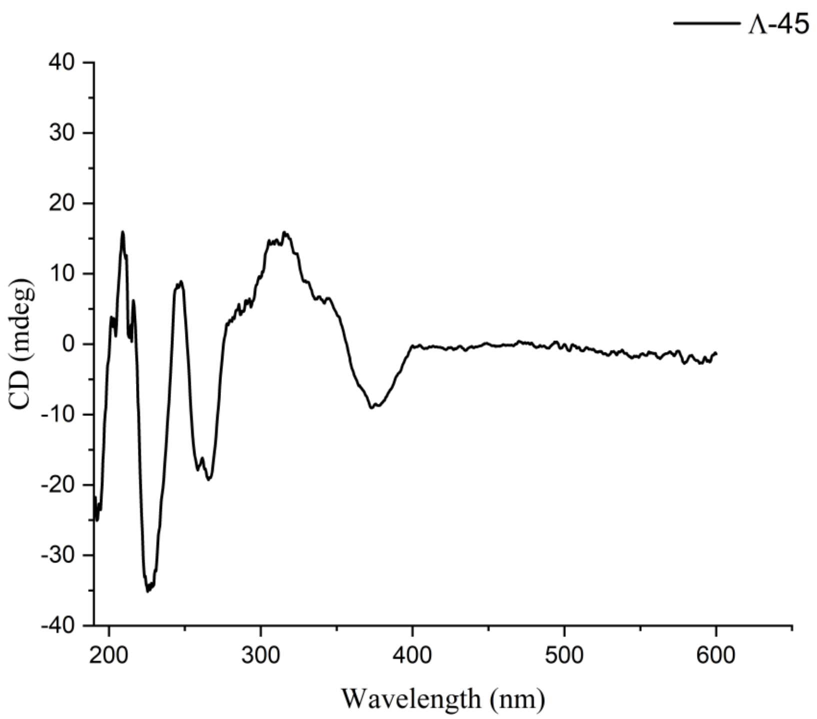

Figure S45. CD spectrum of **Λ-45** recorded in MeCN (0.000025 mol/L)

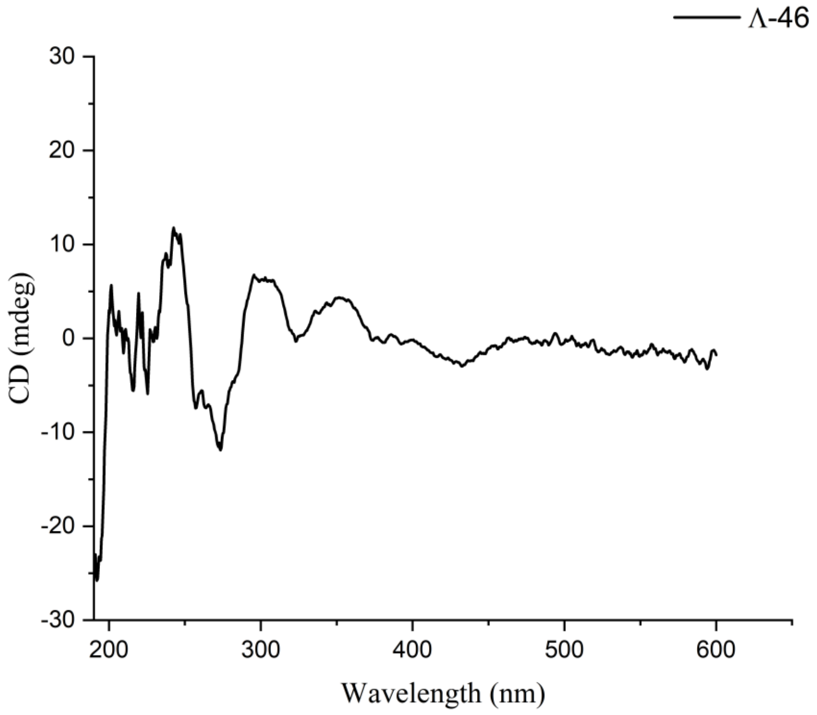

Figure S46. CD spectrum of **Λ-46** recorded in MeCN (0.000025 mol/L)

10. Attempting to Enhance Enantiopurity of Product via Recrystallization

Placed **Λ-28** (10 mg, 94% ee) in a flask. Added the chosen solvent (THF, EtOAc, or DCM) and stirred the mixture to ensure complete dissolution of **Λ-28**. Subsequently, petroleum ether was gradually introduced until solid precipitation was observed. The precipitate was then allowed to settle, followed by centrifugation, ultimately yielding recrystallized **Λ-28**. Enantiomeric excess established by HPLC analysis. (IK-3, 254 nm, *n*-hexane/isopropanol = 80:20, flow rate 1.0 mL/min, 40 ^{o}C, t_r (major) = 8.9 min, t_r (minor) = 10.2 min.)

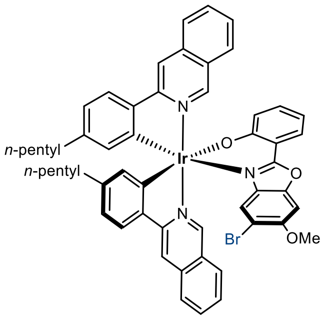

Λ-**28** (94% ee)

Table S3. Recrystallization experiments of **Λ-28**

Solvent	First Recrystallization	Second Recrystallization
THF/petroleum ether	95% ee	95% ee
EtOAc/petroleum ether	94% ee	94% ee
DCM/petroleum ether	94% ee	95% ee

11. Configuration Stability Test of Iridium complex

The **Λ-28** (0.5 mg) was placed in a 2.5 ml glass vial, Added the chosen solvent (THF/ H_2O, ^{i}PrOH or MeCN) and heated to 60°C, and maintained at this temperature for 6 hours. The ee value was subsequently determined. Enantiomeric excess established by HPLC analysis. (IK-3, 254 nm, *n*-hexane/isopropanol = 80:20, flow rate 1.0 mL/min, 40 ºC, t_r (major) = 8.9 min, t_r (minor) = 10.2 min.)

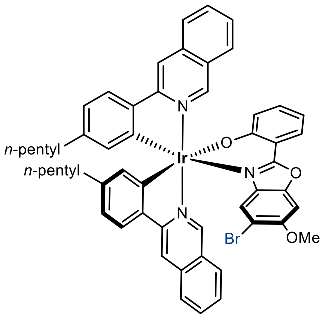

Λ-**28** (94% ee)

Table S4. Configuration Stability Test of **Λ-28**

Solvent	25 ºC (6 h)	60 ºC (6 h)
THF/H_2O (9/1)	94% ee	94% ee
^{i}PrOH	94% ee	94% ee
MeCN	94% ee	94% ee

12. Single Crystal X-Ray Diffraction

Single crystals of Δ-**40** suitable for X-ray diffraction were obtained by slow diffusion from a solution of Δ-**40** (10 mg) in CH_2Cl_2 (1 mL) layered with *n*-hexane (1 mL) at room temperature for several days in a vial.

X-Ray Crystallographic Data of Product **40** (CCDC: 2349911):

(Displacement ellipsoids are drawn at 30% probability level)

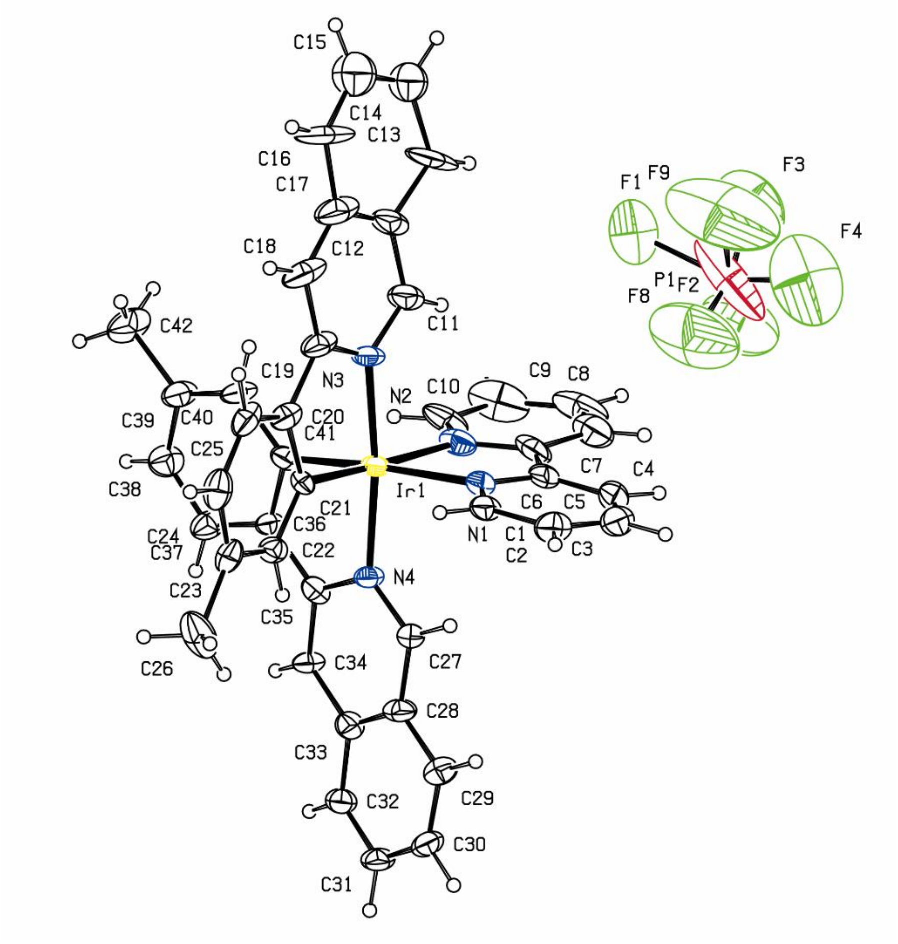

Crystal data and structure refinement for product **40**.

Identification code	a	
Empirical formula	C84 H64 F18 Ir2 N8 P3	
Formula weight	2004.74	
Temperature	120(2) K	
Wavelength	1.54178 Å	
Crystal system	Tetragonal	
Space group	$P4_12_12$	
Unit cell dimensions	a = 16.1441(3) Å	α= 90°.

	b = 16.1441(3) Å	β= 90°.
	c = 36.0222(8) Å	γ = 90°.
Volume	9388.5(4) $Å^3$	
Z	4	
Density (calculated)	1.418 Mg/m^3	
Absorption coefficient	6.577 mm^{-1}	
F(000)	3940	
Crystal size	0.160 x 0.140 x 0.120 mm^3	
Theta range for data collection	3.677 to 68.623°.	
Index ranges	-19<=h<=19, -19<=k<=13, -43<=l<=43	
Reflections collected	61293	
Independent reflections	8671 [R(int) = 0.0517]	
Completeness to theta = 67.679°	99.8 %	
Absorption correction	Semi-empirical from equivalents	
Refinement method	Full-matrix least-squares on F^2	
Data / restraints / parameters	8671 / 6 / 513	
Goodness-of-fit on F^2	1.120	
Final R indices [I>2sigma(I)]	R1 = 0.0498, wR2 = 0.1335	
R indices (all data)	R1 = 0.0531, wR2 = 0.1358	
Absolute structure parameter	0.048(19)	
Extinction coefficient	0.00066(5)	
Largest diff. peak and hole	1.056 and -1.090 e.$Å^{-3}$	

13. Reference

1. Kim, J. Y. *et al.* Rhodium-catalyzed intermolecular amidation of arenes with sulfonyl azides via chelation-assisted C–H bond activation. *J. Am. Chem. Soc.* **134**, 9110–9113 (2012).
2. Yu, X., Tang, J., Jin, X., Yamamoto, Y. & Bao, M. Manganese-catalyzed C−H cyanation of arenes with N-cyano-N-(4-methoxy)phenyl-p-toluenesulfonamide. *Asian J. Org. Chem.* **7**, 550–553 (2018).
3. Hou, Q. *et al.* Synthesis and photoluminescence behavior of π-conjugated 3-substituted isoquinoline derivatives. *Curr. Org. Chem.* **24**, 1517–1526.
4. Yamashita, K., Fujiwara, Y. & Hamashima, Y. Amide-ligand-promoted silver-catalyzed C–H fluorination via radical/polar crossover. *J. Org. Chem.* **88**, 1865–1874 (2023).
5. Sakai, K., Oisaki, K. & Kanai, M. A germanium catalyst accelerates the photoredox α-C(sp3)–H alkylation of primary amines. *Org. Lett.* **24**, 3325–3330 (2022).
6. Zou, G. *et al.* Cyclometalated platinum(II) metallomesogens based on half-disc-shaped β-diketonate ligands with hexacatenar: crystal structures, mesophase properties, and semiconductor devices. *Inorg. Chem.* **61**, 11702–11714 (2022).
7. Chen, L.-A. *et al.* Asymmetric catalysis with an inert chiral-at-metal iridium complex. *J. Am. Chem. Soc.* **135**, 10598–10601 (2013).
8. Subramanyam, C. & Bell, M. R. Tetrahydro 2-saccharinylmerthyl aryl carboxylates. U.S. Patent 5, 306, 818 (1994)
9. Yu, X., Scheller, D., Rademacher, O. & Wolff, T. Selectivity in the photodimerization of 6-alkylcoumarins. *J. Org. Chem.* **68**, 7386–7399 (2003).

10. Welin, E. R., Le, C., Arias-Rotondo, D. M., McCusker, J. K. & MacMillan, D. W. C. Photosensitized, energy transfer-mediated organometallic catalysis through electronically excited nickel(II). *Science* **355**, 380–385 (2017).

11. Ma, J., Zhang, X., Huang, X., Luo, S. & Meggers, E. Preparation of chiral-at-metal catalysts and their use in asymmetric photoredox chemistry. *Nat. Protoc.* **13**, 605–632 (2018).

12. Swords, W. B. *et al.* Highly enantioselective 6π photoelectrocyclizations engineered by hydrogen bonding. *J. Am. Chem. Soc.* **145**, 27045–27053 (2023).
